# Conceptualizing patient-centered care for substance use disorder treatment: findings from a systematic scoping review

**DOI:** 10.1186/s13011-019-0227-0

**Published:** 2019-09-11

**Authors:** Kirsten Marchand, Scott Beaumont, Jordan Westfall, Scott MacDonald, Scott Harrison, David C. Marsh, Martin T. Schechter, Eugenia Oviedo-Joekes

**Affiliations:** 1 School of Population and Public Health, University of British Columbia, 2206 East Mall, Vancouver, BC V6T 1Z3 Canada; 2 Centre for Health Evaluation & Outcome Sciences, Providence Health Care, St. Paul’s Hospital, 575- 1081 Burrard St, Vancouver, BC V6Z 1Y6 Canada; 3 Canadian Association for Safe Supply, 46 East Hastings St, Vancouver, BC V6A 1N1 Canada; 4 Providence Health Care, Providence Crosstown Clinic, 84 West Hastings St, Vancouver, BC V6B 1G6 Canada; 5 Northern Ontario School of Medicine, 935 Ramsey Lake Road, Sudbury, ON P3E 2C6 Canada

**Keywords:** Patient-centered care, Client-centered care, Substance-related disorders, Scoping review, Directed content analysis

## Abstract

**Background:**

Despite ongoing efforts aimed to improve treatment engagement for people with substance-related disorders, evidence shows modest rates of utilization as well as client-perceived barriers to care. Patient-centered care (PCC) is one widely recognized approach that has been recommended as an evidence-based practice to improve the quality of substance use disorder treatment. PCC includes four core principles: a holistic and individualized focus to care, shared decision-making and enhanced therapeutic alliance.

**Aims:**

This scoping review aimed to explore which PCC principles have been described and how they have defined and measured among people with substance-related disorders.

**Methods:**

Following the iterative stages of the Arksey and O’Malley scoping review methodology, empirical (from Medline, Embase, PsycINFO, CINAHL and ISI Web of Science) and grey literature references were eligible if they focused on people accessing treatment for substance-related disorders and described PCC. Two reviewers independently screened the title/abstract and full-texts of references. Descriptive analyses and a directed content analysis were performed on extracted data.

**Findings:**

One-hundred and forty-nine references met inclusion from the 2951 de-duplicated references screened. Therapeutic alliance was the most frequent principle of PCC described by references (72%); this was consistently defined by characteristics of empathy and non-judgment. Shared decision-making was identified in 36% of references and was primarily defined by client and provider strategies of negotiation in the treatment planning process. Individualized care was described by 30% of references and included individualized assessment and treatment delivery efforts. Holistic care was identified in 23% of references; it included an integrated delivery of substance use, health and psychosocial services via comprehensive care settings or coordination. Substance use and treatment engagement outcomes were most frequently described, regardless of PCC principle.

**Conclusions:**

This review represents a necessary first step to explore how PCC has been defined and measured for people accessing substance use disorder treatment. The directed content analysis revealed population and context-specific evidence regarding the defining characteristics of PCC-principles that can be used to further support the implementation of PCC.

## Background

Substance-related disorders are increasingly considered multifactorial health conditions that require evidence-based and public health responses [1]. Substantial efforts have been made to expand the availability of pharmacological, psychosocial and community-based treatments [2, 3]. In addition, practice-based frameworks, such as trauma-informed and culturally competent, responsive and appropriate care, have also been developed [4–6]. In spite of these efforts, global estimates suggest that one out of every six people [7] or less [2, 8] in need of substance use disorder treatment receives it. This treatment gap poses a significant public health concern given that treatment engagement (i.e., retention, adherence) is positively associated with improvements in substance use, health and social functioning [2, 9, 10].

To understand this gap, a growing body of research has focused on treatment process barriers and facilitators from the perspectives of people using substances. Select recent evidence from across populations and settings reveals similarities in peoples’ experiences. For example, structural barriers include the costs and convenience of treatment [11, 12], societal stigma [11–13] and the attitudes and behaviours of health care providers [12, 14]. Research has also shown that people’s preferred treatment goals and outcomes are often incongruent with those of the health care system [15–17]. Additionally, evidence suggests that people want more opportunities to be involved in the substance use disorder treatment planning process [18, 19].

This body of research reveals opportunities to improve the quality of substance use disorder treatment. Examples of existing frameworks include trauma-informed and culturally competent, responsive, and appropriate care. These frameworks emphasize respect for client diversity, an empowerment of people using substances and provider understanding of the varied impact that trauma and ethnicity/culture have on treatment expectations and experiences [4, 5, 20]. In addition, there are emerging interests in the design of patient-centered approaches for substance use disorder treatment (see for example [21–25]). Patient-centered care (PCC) has been widely recommended to strengthen the quality of health care [4, 26, 27] as it can be universally applied across treatments, settings and providers. This framework challenges traditional approaches to treatment by prioritizing the unique needs of each client and seeking a greater balance in power between the client and provider. In the last two decades, the health and social sciences have expanded the conceptualization of PCC. Although this varies slightly between disciplines and settings, the principles of PCC most frequently include the integration of a holistic or bio-psycho-social approach; an individualized focus on clients’ unique needs, goals and preferences; shared power and responsibility between the client and health care provider as with collaborative care or shared decision-making; and a therapeutic alliance [28–31].

Such varying conceptualizations of PCC have posed challenges to its implementation and measurement of its outcomes [30]. Consequently, an important first step toward designing, implementing and evaluating PCC approaches in substance use disorder treatment is a broad exploration of its orientation and conceptualization in this field. To our knowledge, no such reviews exist, although specific principles of PCC have been empirically studied (see for example [22, 24, 32]). Therefore, the aim of the present scoping review was to systematically explore how the principles of PCC have been defined in substance use disorder treatment. Specifically, this study asked:

1. Which PCC principles have been described in substance use disorder treatment settings?
2. How have these PCC principles been conceptualized?
3. What outcomes of PCC principles have been empirically described?

## Methods

A scoping review was deemed the most appropriate and feasible synthesis methodology to capture the breadth of existing evidence. This review followed the classic Arksey and O’Malley framework [33, 34] and best practices for conducting and reporting scoping reviews [35, 36] (Additional file 1). The review’s protocol was registered with Open Science Framework (https://osf.io/5swvd/). Full methodological details are available elsewhere [31] and summarized below.

The search strategy (Additional file 2) was developed as a broad framing of the population (people with substance-related disorders), concept (patient-centered care) and context (health care settings delivering substance use disorder treatment). It was developed in English in Medline (Ovid), refined through extensive consultations with a Health Sciences Librarian and clinical experts (authors SM and SH) and was peer-reviewed. The empirical search for primary studies and previous reviews was conducted in Medline (Ovid), Embase (Ovid), PsycINFO, CINAHL, and ISI Web of Science. The search for grey literature reports and clinical practice guidelines was done in British Columbia Guidelines and Protocols Databases, CPG Infobase, the Registered Nurses’ Association Clinical Practice Guidelines Program, Des Libris, National Guideline Clearinghouse and TRIP.

Two independent reviewers (author KM and SB) selected references through a two-stage screening process. In the first stage, the reviewers screened the de-duplicated titles/abstracts (85% agreement) according to criteria one through three below. In the second stage, titles/abstracts meeting these initial criteria underwent full-text review (93% agreement) based on the full list of eligibility criteria. Empirical and grey literature references were eligible, if they:

1. Included people with substance-related disorders, including tobacco, alcohol, cannabis, stimulants, opioids or had dual diagnoses.
2. Described patient-centered care (i.e., holistic care, individualized care, shared decision-making, therapeutic alliance), trauma-informed care and/or culturally safe care.
3. Were set in a health care context that delivered substance use disorder treatment. This included inpatient (e.g., hospital, residential treatment) or outpatient (e.g., emergency department, primary care, community-based program) settings. This excluded criminal justice settings and self-help models.
4. Were published between 1 January 1960 and 1 July 2018 in English, French, Spanish, Italian or Portuguese.
5. Provided an operational or conceptual definition of the patient-centered care approach.
6. Empirical quantitative references observed at least one patient outcome (e.g., substance use, health) or treatment process outcome (e.g., treatment engagement, treatment satisfaction).

Study screening (including de-duplication) and data extraction was done in DistillerSR [37]. The data charting form (Additional file 3) was used to capture the characteristics of each reference and to identify which principle(s) of PCC were described (objective 1). This form was piloted with the first five empirical and grey literature references. Author KM led data extraction and author SB checked extraction of the PCC principles (94% agreement).

A descriptive overview (including tabular and graphical summaries) of extracted data was completed. In addition, a directed content analysis [38, 39] was performed on the defining characteristics of PCC principles and their outcomes (objectives 2 and 3). This systematic method is particularly beneficial when there exists theories or frameworks (i.e., for patient-centered care) that can guide coding and analysis and can be used to determine patterns and relationships between the content coded [38, 39]. For this analysis, a coding guide (Additional file 4) was developed to identify categories (e.g., therapeutic alliance), sub-categories (e.g., defining characteristics, outcomes associated with) and codes for the defining characteristics (e.g., non-judgment) and outcomes (e.g., frequency of substance use) of each PCC-principle. This guide was developed in iterative stages through consultation with the team’s knowledge users and the initial data extraction process; these codes and categories were then broadly operationalized according to existing PCC frameworks. Directed content analysis also allows new evidence to emerge via open codes, which are used to label content that is unique to predetermined codes [38, 39]. Open coding was used within each of the broader categories (e.g., therapeutic alliance/defining characteristics) and also to identify antecedents to PCC.

Author KM led initial coding; after which, the categories, subcategories, codes and content were reviewed with the team for trustworthiness and for further analysis (e.g., integration, collapsing, or expanding categories). Since the content coded across categories was not mutually exclusive (i.e., a reference could have more than one principle of PCC or more than one category of outcomes coded), data reported are the number of references (i.e., sources) coded at each category, instead of the number of times each reference is coded. The directed content analysis was carried out in NVivo (version 11 for Mac).

## Results

### Descriptive results

**Fig. 1:**
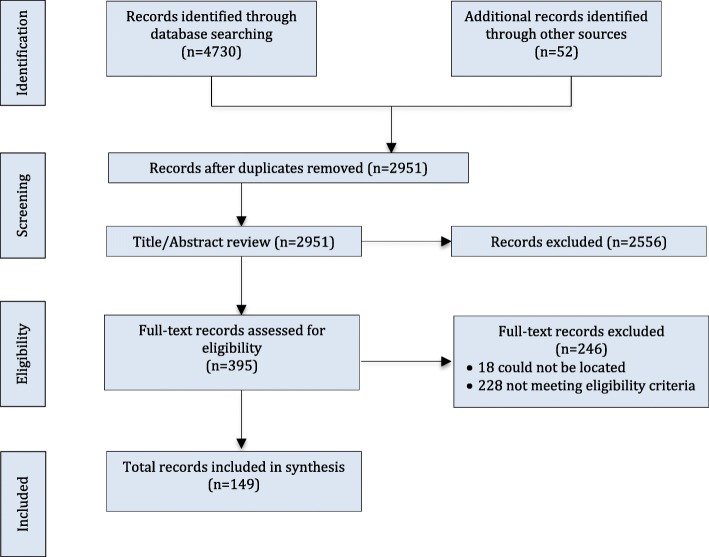
Flow diagram for scoping review process

After de-duplication, 2951 unique references underwent title/abstract screening. Of these, 395 were assessed during the full-text review, with 149 references included (Fig. 1; Additional files 5 and 6 for Excluded References and Detailed Extracted Data). Table 1 provides the characteristics of the eligible references. Approximately 50% of the references were empirical quantitative papers published in the last decade, over two-thirds were based in North America and all but three of the references were published in English. The targeted population was primarily adults receiving substance use disorder treatment. Nearly two-thirds of the references were in outpatient settings and delivered psychosocial treatments. Regarding the principles of PCC, 63 (42.3%) references described more than one PCC principle, and therapeutic alliance was the most frequently described (*n* = 107; 71.8%).

**Table 1:** Extracted characteristics of eligible publications, including the target population, concept and context

Publication Characteristics	Number of references	Percentage of total references
	(*n* = 149)	
Publication Year:		
< 2000	17	11.4
2000–2004	12	8.1
2005–2009	44	29.5
2010–2014	35	23.5
2015-Present	41	27.5
Publication Type:		
Empirical Quantitative Study	74	49.7
Empirical Qualitative Study	25	16.8
Empirical Mixed-Methods	3	2.0
Empirical Review	5	3.4
Report	25	16.8
Clinical Practice Guideline	17	11.4
Publication Location:		
Africa	1	0.7
Asia	2	1.3
Australia	8	5.4
Europe	37	24.8
North America	100	67.1
South America	1	0.7
Publication Language:		
English	146	98.0
French	3	2.0
Population Sampled or Targeted:		
Adult clients with substance-related disorders	96	64.4
Youth clients with substance-related disorders	21	14.1
Health care providers delivering substance use disorder treatment	27	18.1
Both clients and health care providers	5	3.4
Primary Substance Used or Targeted:		
Alcohol	23	15.4
Cannabis	7	4.7
Opioids	17	11.4
Stimulants	4	2.7
Tobacco	13	8.7
Poly-substance ^a^	6	4.0
Dual diagnosis ^b^	19	12.8
People receiving addiction treatment in general ^c^	60	40.3
Health Care Setting: ^d^		
Inpatient	28	18.8
Outpatient	99	66.4
Inpatient & Outpatient	22	14.8
Type of Addiction Treatment: ^e^		
Pharmacological	7	4.7
Psychosocial	99	66.4
Psychosocial & Pharmacological	33	22.1
Not specified	10	6.7
Patient-Centered Care Principles (not mutually exclusive categories):		
Holistic care	35	23.5
Individualized care	46	30.9
Shared decision-making	54	36.2
Therapeutic alliance	109	73.1
Trauma-informed care	9	6.0
Culturally-safe care	8	5.4
More than one principle described	63	42.3
All four PCC principles described	7	4.7

^a^Poly-substance use included references that targeted people using more than one substance category (e.g., alcohol, opioids and stimulants) or people using injection drugs (e.g., opioids or stimulants)

^b^Dual diagnosis included references that targeted people with diagnoses for substance-related disorders and mental health conditions (e.g., post-traumatic stress disorder and opioid use)

^c^Not a targeted substance category included references that were primarily based on convenience samples of people receiving inpatient or outpatient treatment for substance use. Therefore the samples were a mix of people with problematic licit and illicit substance use

^d^Inpatient settings included hospitals or residential addiction-specific treatment centers. Outpatient settings included general primary care or addiction specific outpatient programs (e.g., opioid agonist treatment clinics)

^e^Pharmacological treatment included any medication-based substitute interventions (e.g., methadone maintenance treatment, nicotine replacement therapy). Psychosocial treatment included any behavioural treatments (e.g., cognitive behavioural therapy, contingency management, strengths-based treatment). When a combination of behavioural and medication-assisted interventions was used, the reference was classified as using a combined approach. For the 10 references where the type of treatment was not specified, 4 references were guidelines written about general approaches for the delivery of addiction treatment, and therefore, could be considered applicable to both psychosocial and pharmacological interventions. The remaining 6 references generally described addiction treatment as delivered in residential settings or primary care based settings, without specifying the particular treatments delivered

### Directed content analysis of the defining characteristics of PCC principles

Tables 2, 3, 4, 5, 6 and 7 summarize the results of the directed content analysis of defining characteristics for each PCC principle, and include representative examples of the content coded. For holistic care (Table 2), the sub-categories converged in their aim to provide *“wraparound services that meet clients' needs at a given point in time”* [44]. For most of these references, this included an integrated delivery (*n* = 25) or the coordination (*n* = 15) of additional health (e.g., primary care, specialist care, nutrition, exercise) and psychosocial supports (e.g., housing, financial, legal, family) within a substance use disorder treatment setting. Gender-responsive services (*n* = 9) described an integrated approach, wherein women’s health, substance use and psychosocial treatment needs were comprehensively addressed. Finally, four references described the delivery of substance use disorder treatment within a primary care or hospital setting.

**Table 2:** Directed content analysis of the defining characteristics of holistic care

Defining Characteristics ^a^	Representative Example of Content Coded
Integrated delivery of physical health, mental health or psychosocial supports within addiction treatment setting (*n* = 25) ^b^	“Other interventions designed to improve the potential for a successful outcome included educational sessions about the harmful effects of smoking and the benefits of stopping, stress management, the value of developing a support network, improving nutrition and avoiding significant weight gain after stopping smoking, the importance of a safe and regular exercise program, and understanding the potential role of spirituality.” [40]
Coordination of health or psychosocial services as part of addiction treatment (n = 15) ^c^	“If a woman was involved with many service providers, the ICF [Integrated Care Facilitator], with the woman’s permission, would maintain contact with those providers to ensure that all providers understood her needs in a similar way and that services were coordinated.” [41]
Adapting a gender-responsive approach to the delivery of health, substance use, and psychosocial treatment (n = 9) ^d^	“It allows clinicians to treat addiction as the primary problem while also addressing the complexity of issues that women bring to treatment: genetic predispositions, health consequences, shame, isolation, histories of abuse, or a combination of these.” [42]
Integrated delivery of addiction treatment as part of a primary care or hospital setting for other health or psychosocial needs (n = 4) ^e^	“NRT [Nicotine Replacement Therapy] was available to participants at no cost during hospitalization.[…] A variety of group meetings were held according to a preset time schedule which was announced at the unit. The degree to which patients participated in the meetings differed depending on the length of their hospital stay.” [43]

^a^A total of 35 references defined holistic care. Coded categories were not mutually exclusive such that a reference might have defined the principle of patient-centered care at more than one category. Bracketed numbers represent the number of unique references coded at each category

^b^References coded at this category [20, 25, 40–62]

^c^References coded at this category [41, 44, 45, 47, 50, 51, 56, 63–70]

^d^References coded at this category [41, 42, 44, 45, 50, 55, 65, 69, 71]

^e^References coded at this category [43, 54, 67, 70]

**Table 3:** Directed content analysis of the defining characteristics of individualized care

Defining Characteristics ^a^	Representative Example of Content Coded
Individualized assessment and treatment planning (n = 29) ^b^	“Needs assessment and treatment planning activities are necessary to match patients to appropriate treatments. […] Similarly, care plans must include provisions for monitoring the client’s progress after the index episode of treatment, given that posttreatment relapse is so common.” [69]
Delivery of treatment according to patient needs and preferences (n = 24) ^c^	“The participants in this residential program used as much medication as was necessary to suppress nicotine withdrawal symptoms which often was more than what is typically prescribed.” [40]
Treatment adapted to clients’ barriers and assets (*n* = 11) ^d^	“A typical call included discussion of the reasons the participant sought and discontinued treatment; the participant’s current intentions regarding alcohol and drug use with a focus on increasing motivation to achieve or maintain abstinence; the participant’s thoughts about what might be most helpful at this time; and troubleshooting practical barriers to treatment.” [72]

^a^A total of 46 references defined individualized care. Coded categories were not mutually exclusive such that a reference might have defined the principle of patient-centered care at more than one category. Bracketed numbers represent the number of unique references coded at each category

^b^References coded at this category [20, 40, 42, 43, 45, 47, 50, 52, 64, 69, 72–89]

^c^References coded at this category [25, 40, 43, 46, 47, 50, 52–57, 63, 67, 71, 75–77, 84, 87, 90–93]

^d^References coded at this category [20, 45, 52–54, 64, 67, 72, 74, 86, 88]

**Table 4:** Directed content analysis of the defining characteristics of shared decision-making

Defining Characteristics ^a^	Representative Example of Content Coded
Client and provider dialogue to reach a mutual decision (n = 31) ^b^	“The form of NRT [Nicotine Replacement Therapy] selected is a joint decision made by the client and advisor, and is based on the client’s individual smoking habits and feelings as well as any contraindications.” [76]
Autonomous decision-making (*n* = 17) ^c^	“Participants appreciated the practitioners’ active listening skills. For example, one client noted that her request to not use tablets or patches for smoking cessation was recognised by the practitioners as the topic was not broached again in consultations.” [94]

^a^A total of 54 references defined shared decision-making. Coded categories were not mutually exclusive such that a reference might have defined the principle of patient-centered care at more than one category. Bracketed numbers represent the number of unique references coded at each category

^b^References coded at this category [20, 22, 25, 40, 41, 45, 47, 52, 59, 61, 63, 68, 69, 73–81, 84, 90, 93–99]

^c^References coded at this category [20, 23, 45, 51, 59, 61, 64, 71, 72, 75–78, 80, 90, 94, 98]

**Table 5:** Directed content analysis of the defining characteristics of therapeutic alliance

Defining Characteristics ^a^	Representative Example of Content Coded
Non-judgmental, respectful and accepting (*n* = 37) ^b^	“A major theme discussed by patients was the importance of building supportive relationships. Patients expressed a desire to work with staff who possessed qualities such as empathy, understanding, trust, respect and expertise and described feeling accepted in these relationships. Patients who perceived staff to be nonjudgmental in their approach described that this reduced their feelings of shame.” [51]
Empathy, understanding, warmth, kindness, supportive (*n* = 32) ^c^	“The nurse engages in caring relationships with patients with the purpose of helping them to handle a complex and intricate health problem in a dignified manner, acknowledging the therapeutic effects of feeling being understood as a patient.” [43]

^a^A total of 109 references defined therapeutic alliance. Coded categories were not mutually exclusive such that a reference might have defined the principle of patient-centered care at more than one category. Bracketed numbers represent the number of unique references coded at each category

^b^References coded at this category [45, 48, 51, 57, 59, 61–66, 71, 76, 77, 79, 83, 89, 91, 93, 94, 96, 99, 103, 104, 118–128]

^c^References coded at this category [41, 43, 45, 48, 51, 57, 61, 63–66, 71, 74, 89–91, 96, 103, 118–121, 123, 125–132]

**Table 6:** Directed content analysis of the defining characteristics of trauma-informed care

Defining Characteristics ^a^	Representative Example of Content Coded
Trauma-informed framework (*n* = 6) ^**b**^	“SAMHSA outlines a “four R” perspective for the elements that are required to create this shift in organizational culture: (1) realizing the prevalence of trauma, (2) recognizing how trauma affects all individuals involved with the organization (clients, families and team members), (3) responding by putting this knowledge into practice, and (4) actively resisting retraumatization.” [133]
Understanding the effects of trauma (*n* = 3) ^**c**^	“Taking into account the impact of trauma on the lives, development, and drug use of people. This does not necessarily require disclosure of trauma.” [59]
Avoiding re-traumatization (n = 1) ^**d**^	“We should make great efforts to do nothing that could be retraumatizing, such as exercising authority and/or control, asking intrusive questions, being unpredictable, or using shaming language/ techniques.” [79]

^a^A total of 9 references defined trauma-informed care. Coded categories were not mutually exclusive such that a reference might have defined the principle of patient-centered care at more than one category. Bracketed numbers represent the number of unique references coded at each category

^b^References coded at this category [41, 42, 58, 133–135]

^c^References coded at this category [44, 59, 79]

^d^References coded at this category [79]

**Table 7:** Directed content analysis of the defining characteristics of culturally-safe care

Defining Characteristics ^a^	Representative Example of Content Coded
Adapting care plans to meet culture-specific preferences (n = 7)	“Akeela House developed a model that incorporated traditional Alaska Native cultural lifestyles into the therapeutic community treatment approach. This was termed a “Spirit Camp Model” and consisted of four major elements: (1) spirit groups, (2) cultural awareness activities, (3) urban orientation, and (4) individual counseling. To implement these components, additional Alaska Native counselors were hired.” [136]
Inquiring about health and healing practices of the client (n = 2)	“The nurse engages with Charlie to prioritize his needs. He/she discusses his living situation and how he sees the future. The nurse does an assessment in keeping with the principles of cultural safety and cultural competence – he/she begins by asking Charlie if there is anything that he/she should know about him (e.g. beliefs about health and healing practices) to assist with his treatment plan and before making referrals etc.” [59]
Reflecting on personal beliefs, assumptions and biases (n = 2)	“The concept of cultural safety takes critical inquiry a step further by requiring nurses to reflect on issues of racialization, institutionalized discrimination, culturalism, and health and health-care inequities.” [59]

^a^A total of 8 references defined culturally-safe care. Coded categories were not mutually exclusive such that a reference might have defined the principle of patient-centered care at more than one category. Bracketed numbers represent the number of unique references coded at each category

^b^References coded at this category [6, 40, 45, 47, 136–138]

^c^References coded at this category [59, 138]

^d^References coded at this category [45, 59]

Individualized care (Table 3) was defined by health care providers’ efforts to understand clients’ unique needs, preferences, and expectations. The first of such efforts was the use of individualized assessments and treatment plans, both at entry and throughout treatment (*n* = 29). Eight of these references (8/26 = 30.8%) used a specific tool (e.g., Goals of Treatment Questionnaire [73]) in these assessments. More frequently, a general process of assessment was described, whereby health care providers took time to understand the *“main problems to be addressed, what actions and resources were needed, who is responsible and timeframes for action and review”* [74]. The second defining sub-category described efforts to deliver treatment by *“fit* [ting] *services to the individual, based on an ongoing assessment of that person’s needs and level of functioning”* [75] (*n* = 24). This often included presenting clients with a range of treatment options that responded to those assessed needs and preferences. Examples of treatment options included group or individual counseling, medication options (when there was more than one), the schedule and frequency of visits, and location of visits. This was sometimes referred to as “treatment-matching” (*n* = 9) or “as-needed dosing” (*n* = 3).

Shared decision-making (Table 4) was defined in the first sub-category by activities or strategies whereby the client and provider engaged in dialogue to reach a mutual decision on the best course of treatment (*n* = 31), including choice of the intervention, its frequency, duration, and follow-up plans. Here, health care providers elicited clients’ preferences and needs, presented information on the available treatment options, and then the client and provider *“negotiated dialogues towards a mutually agreed upon destination”* [95]. In the second sub-category, decision-making was referred to as an autonomous and client-led approach (*n* = 17). Clients were described as having responsibility and control over their treatment decisions, including the frequency of counseling (e.g. [20, 23]), choice of medications or behavioural interventions, and transition plans (e.g. [76–78]). These two defining categories were similar in their empowering view of clients as an *“integral partner, rather than passive or compliant recipient, of a treatment program”* [75]. They diverged in the extent of autonomy that the client had and in their emphasis on the dialogue process.

Therapeutic alliance was defined by relationships that were non-judgmental, respectful and accepting (*n* = 37) and/or as empathic, understanding, warm and kind (*n* = 32). While these defining categories reflected distinct relational qualities, there was substantial overlap between them (*n* = 26 references coded at both), as shown in the representative examples in Table 3. In addition to these characteristics, 56 (52.3%) references defined therapeutic alliance according to empirically-based measures, such as the widely used Working Alliance Inventory. Among these references, therapeutic alliance was commonly explored as a predictor or mediating variable of substance use and treatment engagement outcomes.

Trauma-informed care was defined according to existing theoretical and clinical practice frameworks (*n* = 6), such as Seeking Safety (*n* = 2) and Harris & Fallot’s trauma theory (n = 2). Open coding captured additional defining features, including understanding the effects of trauma (*n* = 3) and avoiding re-traumatization (*n* = 1). Defining characteristics of culturally-safe care included open codes for adapting care plans according to culturally-relevant preferences of clients (*n* = 7), inquiring about the health and healing beliefs of clients (n = 2), and health care providers’ reflection of their personal beliefs and biases (n = 2).

### Directed content analysis of outcomes of PCC principles

**Fig. 2:**
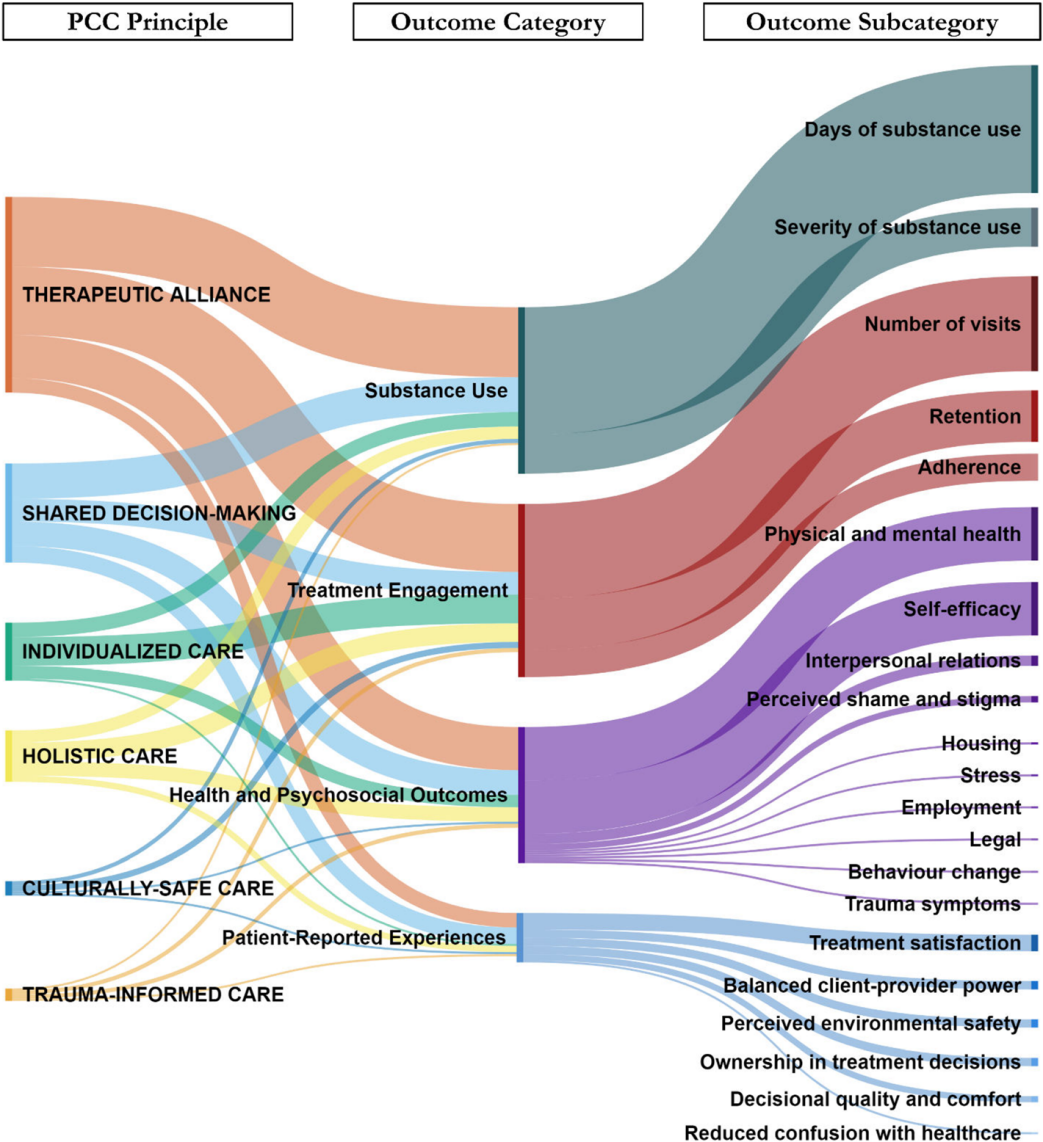
Directed content analysis of outcomes of patient-centered care principles. a) Among publications reporting outcomes of the patient-centered care principles, the Sankey diagram presents the general outcome category and sub-category and the relative number of times it was coded within each patientcentered care principle. b) Outcome categories and sub-categories are not mutually exclusive. A publication could have described more than one (e.g., Substance Use and Treatment Engagement). If a publication operationalized more than one principle and/or outcome, each principle received a link to each general outcome category. Additional space seen in the general outcome category nodes and their flows to sub-outcomes is from publications that studied multiple suboutcomes under one outcome category since each principle did not receive an additional link to a general outcome category for each sub-outcome studied in that category

A total of 103 (69%) references were identified as describing or exploring at least one of the predetermined categories of outcomes. The sankey diagram (Fig. 2) displays nodes for each principle of PCC, as well as the categories and sub-categories of coded outcomes. The width of each flow represents, among those references that identified outcomes, the relative distribution of which PCC principles were studied or described, and in association with which outcome categories and sub-categories. As shown in Fig. 2, therapeutic alliance and shared decision-making contributed a higher number of references with identified outcomes. Within each of the PCC principles, substance use (*n* = 52/103; 50.5%) and treatment engagement outcomes (*n* = 50/103; 48.5%) were the most frequently coded categories, followed by health and psychosocial outcomes (*n* = 40/103; 38.8%), and patient-reported experiences (*n* = 17/103; 16.5%). Within each of the broader outcome categories, the most frequent subcategories included the number of days of substance use, number of visits or sessions attended, physical and mental health symptoms, perceived self-efficacy, and treatment satisfaction.

### Directed content analysis of antecedents to PCC

During the directed content analysis, open codes were used to distinguish antecedents contributing to, or strengthening the implementation of PCC from those that were described as outcomes. Since this was not part of the original review design, these factors were analyzed inductively and based on their within-category content. A total of 75 (50.3%) references were coded for describing such contributors; the emergent categories and sub-categories are displayed in Table 8. Organizational values, policies, and procedures (*n* = 42; 56.0%) clustered around six features. These included the skills and training of providers (e.g., case management, multicultural competence) and environments that were safe, stable, and social. Clinical approaches (*n* = 49; 65.3%) also emerged as contributing to therapeutic alliance (*n* = 43; e.g., communication style, building trust), shared decision-making (*n* = 30; e.g., appropriate information sharing; empowering approach), or individualized care (*n* = 6; e.g., encouraging client input).

**Table 8:** Directed content analysis of antecedents to patient-centered care

Categories (n references coded)	Open codes (n references coded)
1. Organizational Values, Policies and Procedures (n = 42)	1.1 Health care provider skills and training (e.g., case management, motivational interviewing, transtheoretical change model) (*n* = 18)
	1.2 Creating preferred environments that are safe, stable and social (n = 11)
	1.3 Inter-professional care teams (n = 9)
	1.4 Simplifying the logistics and continuity of access to health care providers (*n* = 9)
	1.5 A system that is rooted in the values of harm reduction and the social determinants of health (n = 6)
	1.6 Comprehensive assessment and screening procedures (n = 3)
2. Clinical Approaches that Strengthen Therapeutic Alliance (n = 43)	2.1 Open communication and active listening (*n* = 28)
	2.2 Investing time to build trust (*n* = 20)
	2.3 Affirming client’s ability to succeed in their goals (n = 17)
	2.4 Adopting an individualized approach (*n* = 8)
	2.5 Collaborating with clients (n = 7)
	2.6 Taking a holistic view (*n* = 7)
3. Clinical Approaches that Support Shared Decision-making (n = 30)	3.1 Sharing information in a manner appropriate for the client (*n* = 21)
	3.2 Empowering clients as experts in treatment need and building capacity for self-responsibility (*n* = 13)
	3.3 Establishing respectful relationship with clients (n = 4)
	3.4 Being flexible in approaches offered (n = 2)
4. Clinical Approaches that Support Individualized Care (n = 6)	4.1 Encouraging clients’ input and preferences (n = 3)
	4.2 Establishing caring relationship with clients (n = 2)
	4.3 Offering a flexible continuum of care (n = 2)

## Discussion

To our knowledge, this scoping review is the first to undertake a systematic synthesis of PCC in substance use disorder treatment settings. To strengthen the breadth and specificity of this review, existing frameworks of PCC from other disciplines were used to guide the search strategy and data charting methods [31], and the directed content analysis allowed population and context specific nuances to be identified. The findings suggested that few references had examined all four principles of PCC, although 42% described more than one PCC principle. The most frequent principle identified was therapeutic alliance and the most frequent outcomes measured included substance use and treatment engagement. The findings contribute evidence that can be used to support a comprehensive and evidence-based conceptualization of PCC with implications for its implementation and evaluation.

The first objective was to determine which PCC principles have been described in substance use disorder treatment settings, and the results revealed that therapeutic alliance was the most frequently described principle. The first plausible explanation for this is the longstanding tradition of therapeutic alliance in psychotherapeutic research and practice [100]. In the present review, two-thirds of the references offered primarily psychosocial treatments (e.g., cognitive behavioral therapy) for substance-related disorders. In this discipline, therapeutic alliance receives significant attention given its importance in predicting counseling outcomes [32, 100]. In the references that described therapeutic alliance, over 50% were empirical quantitative papers and conceptualized therapeutic alliance according to client, provider, or observer-rated empirical measures, such as the Working Alliance Inventory (WAI). Thus, this longstanding tradition to examine the extent of therapeutic alliance likely contributed to the high number of references in the present review that described this PCC-principle.

Our search also yielded references that delivered additional treatments (e.g., pharmacological treatments alone or combined with psychosocial) in alternative settings (e.g., residential detoxification programs, harm reduction services), which provided an opportunity to determine that non-judgment, respect, empathy, and understanding were also common characteristics of therapeutic alliance. While respect has been described in broader conceptual analyses of PCC [29, 101], information regarding why these attributes were important among people with substance use strengthens its interpretation in this context. Examples of these reasons included clients’ safety [79, 102]; to gain clients’ trust [45, 63, 103]; and to reduce stigma [63, 96, 104]. These defining attributes are especially salient when considering that experiences of stigma are common among people with substance-related disorders [105–107] and have been identified as barriers to treatment [12, 14].

These relational characteristics also intersected with shared decision-making, such that the analysis of antecedents to PCC revealed that respectful and understanding relationships promoted shared decision-making. The reciprocal was also found, whereby collaborative approaches strengthened therapeutic alliance. These antecedents give more depth to our finding that the defining characteristics of shared decision-making denoted an underlying philosophy of respect towards clients as “integral … rather than passive” partners in the treatment process. The first defining characteristic emphasized a joint decision-making process. This category primarily described a process of dialogue and discussion that granted clients a more active role in the decision-making process and facilitated the health care provider’s understanding of clients’ needs and expectations. This view of shared decision-making resembles those of the broader PCC-frameworks that have conceptualized this principle as “sharing power and responsibility” [28], “finding common ground” [108] and also more recent proposals for the clinical practice of shared decision-making [109]. However, the second defining characteristic emphasized a fully autonomous decision-making process, which is more closely aligned with other frameworks’ notion of “empowering care” [29, 110]. Those existing frameworks describe autonomy, self-confidence and self-determination as core characteristics of this principle. While, there is evidence that increasingly recognizes clients’ preferences to be more actively involved in substance use disorder treatment decision-making [22], further work might explore the circumstances under which a deliberative or autonomous decision-making process is more suitable from the client and health care provider’s perspective.

The integration of shared decision-making practices often presumed an individualized care approach, such that the process of dialogue involved discussion of clients’ unique needs, circumstances, traditions and preferences [29]. One study to highlight is that of Joosten et al. who developed and tested the effectiveness of a shared decision-making intervention in an inpatient treatment setting [73, 80, 81]. Their intervention relied on an individualized assessment of clients’ needs and goals (via the Camberwell Assessment of Need). The client and clinician then discussed their independent ranking of the priority of these goals and adapted treatment accordingly. In this review, individualized care did not always include shared decision-making however; it was also described by several treatment matching approaches, such as as-needed-dosing [40, 46, 90]. Regardless of the specific design chosen, these findings imply that comprehensive assessments and flexibility in service design and delivery (at a clinical and organizational level) supported individualized care.

Thus, individualized needs assessment and treatment delivery overlapped with holistic, trauma-informed and culturally competent, responsive, and appropriate care with respect to their common goal to provide comprehensive and flexible care, adapted to client-identified needs and values. Under ideal circumstances, such consideration would be facilitated by an assessment of clients’ bio-psycho-social needs [47], which are often inextricable from their cultural context and the pervasive impacts of structural and interpersonal trauma [20]. In the present review, the defining characteristics of these principles included specific practices adopted by health care providers (e.g., comprehensive needs assessments, avoiding re-traumatization). However, the inductive analysis of antecedents to PCC revealed that both the system (e.g., a vision of shared governance; safety and stability of treatment setting; flexibility of service provision) and the health care provider (e.g., communication style) play a conjoint role in the successful implementation of PCC principles. For instance, a physician’s endeavor to adopt shared decision-making practices in the prescription of opioid substitution treatment will require a health care system that has implemented evidence-based treatment options and policies that support client-provider collaboration (e.g., flexibility around dosing schedules, frequency of visits, etc). Thus, a good starting point for moving PCC into the realm of evidence-based practice in substance use disorder treatment is a consideration of potential barriers to its implementation from the client, provider and system’s perspectives [111].

While factors supporting PCC have been relatively consistent across broader concept analyses [29, 30, 112], there has been less agreement on what consequences or outcomes of PCC can be expected and thus, measured. Examples of such outcomes have included consultation processes (e.g., communication skills, quality of care, treatment satisfaction) [29, 113], health behaviours (e.g., service utilization, adherence to treatment plans) [113, 114], health outcomes [29] and patient-reported outcomes [112]. It has also been proposed that some of these outcomes are likely more intermediate (i.e., perceived quality of care, satisfaction, consultation process outcomes), while others more distal (i.e., health behaviours and health outcomes) [113].

In the present review, substance use and treatment engagement outcomes were the most frequently investigated, regardless of PCC principle. This might have been influenced by the high frequency of references exploring therapeutic alliance, half of which related the WAI with the number of days of substance use or number of counseling sessions. However, it might also reflect a common assumption that the goal of any substance use disorder treatment is to reduce the severity of use [115]. A continued emphasis on substance use outcomes neglects that the stated goal of PCC is to improve the treatment process [26, 27]. It is also not congruent with prior research demonstrating that clients’ goals extend to other domains (e.g., health, housing, family relationships) [116] and emerging recommendations to integrate patient-centered or patient-reported measures in substance use disorder treatment [117]. Thus, future PCC research in substance use disorder treatment will be strengthened through choices of measures that reflect these goals.

This review was a necessary first step towards conceptualizing PCC for substance use disorder treatment. Although scoping reviews typically take a broad framing of the population, concept and context [33, 34], this resulted in a high number of false positives and posed several challenges to the synthesis and to teasing apart potential differences in treatment type and setting. While efforts were made to overcome these challenges (i.e., substantial resources were devoted to reaching adequate inter-rater agreement and carrying out the directed content analysis), there are further limitations to bear in mind. First, we were unable to carry out a comprehensive grey literature search in international search databases, other than TRIP, and thus, might not have adequately captured grey literature reports of the implementation of PCC in settings outside of North America. In addition, our search strategy was developed in English and eligibility was limited to references published in English, French, Spanish, Italian or Portuguese. This might have influenced the comprehensiveness and international breadth of the search and thus, inflating the number of references from North America.

## Conclusions

The present scoping review synthesized existing empirical and grey literature as a necessary first step to explore which PCC principles have been described and how they have defined and measured for people engaging in substance use disorder treatment. The directed content analysis revealed population and context-specific evidence regarding the defining characteristics. These results can be used to support the implementation and evaluation of PCC. The results also identify future directions for research, including potential measures of PCC and its associated outcomes.

## Supplementary information

**Additional file 1.** Prisma-ScR checklist. (PDF 82 kb)

Additional file 2. Sample Ovid Medline search terms. (PDF 470 kb)

Additional file 3. Data extraction form. (PDF 287 kb)

Additional file 4. Directed Content Analysis Coding Guide. (PDF 140 kb)

Additional file 5. Excluded references. (PDF 2429 kb)

Additional file 6. Detailed extracted data. (XLSX 33 kb)

## Data Availability

The datasets used and/or analysed during the current study are available from the corresponding author on reasonable request.

## Abbreviations

PCC: Patient-centered care
WAI: Working alliance inventory

## References

[1] United Nations Office on Drugs and Crime. Our joint commitment to effectively addressing and countering the world drug problem. United Nations General Assembly on the World Drug Problem2016.

[2] Substance Abuse and Mental Health Services Administration. Chapter 4, Early Intervention, Treatment, And Management of Substance Use Disorders. Washington (DC): US Department of Health and Human Services; 2016.

[3] European Drug Monitoring Centre for Drugs and Drug Addiction. Health and Social Responses to Drug Problems: A European Guide. Luxembourg: Publications Office of the European Union; 2017.

[4] Bassuk EL, Latta RE, Sember R, Raja S, Richard M (2017). Universal Design for Underserved Populations: person-centered, recovery-oriented and trauma informed. J Health Care Poor Underserved.

[5] Gainsbury SM (2017). Cultural competence in the treatment of addictions: theory, practice and evidence. Clin Psychol Psychother.

[6] Steinka-Fry KT, Tanner-Smith EE, Dakof GA, Henderson C (2017). Culturally sensitive substance use treatment for racial/ethnic minority youth: a meta-analytic review. J Subst Abus Treat.

[7] United Nations Office on Drugs and Crime (2017). International standards for the treatment of drug use disorders.

[8] Pirie T (2015). & National Treatment Indicators Working Group. National Treatment Indicators Report: 2012–2013 data.

[9] Zhang Z, Friedmann PD, Gerstein DR (2003). Does retention matter? Treatment duration and improvement in drug use. Addiction..

[10] Simpson DD, Joe GW, Rowan-Szal GA (1997). Drug abuse treatment retention and process effects on follow-up outcomes. Drug Alcohol Depend.

[11] Muthulingam D, Bia J, Madden LM, Farnum SO, Barry DT, Altice FL (2019). Using nominal group technique to identify barriers, facilitators, and preferences among patients seeking treatment for opioid use disorder: a needs assessment for decision making support. J Subst Abus Treat.

[12] Matthews AK, Cesario J, Ruiz R, Ross N, King A (2017). A qualitative study of the barriers to and facilitators of smoking cessation among lesbian, gay, bisexual, and transgender smokers who are interested in quitting. LGBT Health.

[13] Hewell VM, Vasquez AR, Rivkin ID. Systemic and individual factors in the buprenorphine treatment-seeking process: a qualitative study. Subst Abuse Treat Prev Policy. 2017;12(3):1–10. 10.1186/s13011-016-0085-y PMC5237159 28086837

[14] Reisinger HS, Schwartz RP, Mitchell SG, Peterson JA, Kelly SM, O'Grady KE (2009). Premature discharge from methadone treatment: patient perspectives. J Psychoactive Drugs.

[15] Hodgins DC, Leigh G, Milne R, Gerrish R (1997). Drinking goal selection in behavioral self-management treatment of chronic alcoholics. Addict Behav.

[16] Joosten EAG, De Weert-Van Oene GH, Sensky T, Van Der Staak CPF, De Jong CAJ (2011). Treatment goals in addiction healthcare: the perspectives of patients and clinicians. Int J Soc Psychiatry.

[17] Alves P, Sales C, Ashworth M (2017). Does outcome measurement of treatment for substance use disorder reflect the personal concerns of patients? A scoping review of measures recommended in Europe. Drug Alcohol Depend.

[18] Friedrichs A, Silkens A, Reimer J, Kraus L, Scherbaum N, Piontek D (2018). Role preferences of patients with alcohol use disorders. Addict Behav.

[19] Marchand K, Palis H, Peng D, Fikowski J, Harrison S, Spittal P (2015). The role of gender in factors associated with addiction treatment satisfaction among long-term opioid users. J Addict Med.

[20] Registered Nurses Association of Ontario. Engaging clients who use substances. Ontario: Registered Nurses’ Association of Ontario; 2015.

[21] Marchand K, Oviedo-Joekes E (2017). Prioritizing the patient in patient-centered addictions treatment. Addiction..

[22] Friedrichs A, Spies M, Härter M, Buchholz A (2016). Patient preferences and Shared Decision Making in the treatment of substance use disorders: A systematic review of the literature. PLoS One.

[23] Schwartz RP, Kelly SM, Mitchell SG, Gryczynski J, O'Grady KE, Gandhi D (2017). Patient-centered methadone treatment: a randomized clinical trial. Addiction..

[24] Barrio P, Gual A (2016). Patient-centered care interventions for the management of alcohol use disorders: a systematic review of randomized controlled trials. Patient Prefer Adherence..

[25] DiClemente CC, Norwood AEQ, Gregory WH, Travaglini L, Graydon MM, Corno CM (2016). Consumer-centered, collaborative, and comprehensive care: the Core essentials of recovery-oriented system of care. J Addict Nurs.

[26] Institute of Medicine (IOM) (2001). Crossing the Quality Chasm: A New Health System for the 21st Century.

[27] Beaulieu MD (2013). President’s message: toward a patient-centred health care system. Can Fam Physician.

[28] Mead N, Bower P (2000). Patient-centredness: a conceptual framework and review of the empirical literature. Soc Sci Med.

[29] Morgan S, Yoder LH (2012). A concept analysis of person-centered care. J Holist Nurs.

[30] Scholl I, Zill JM, Harter M, Dirmaier J (2014). An integrative model of patient-centeredness - a systematic review and concept analysis. PLoS One.

[31] Marchand K, Beaumont S, Westfall J, MacDonald S, Harrison S, Marsh DC (2018). Patient-centred care for addiction treatment: a scoping review protocol. BMJ Open.

[32] Meier PS, Barrowclough C, Donmall MC (2005). The role of the therapeutic alliance in the treatment of substance misuse: a critical review of the literature. Addiction..

[33] Arksey H, O'Malley L (2005). Scoping studies: towards a methodological framework. Int J Soc Res Methodol.

[34] Colquhoun HL, Levac D, O'Brien KK, Straus S, Tricco AC, Perrier L (2014). Scoping reviews: time for clarity in definition, methods, and reporting. J Clin Epidemiol.

[35] Tricco Andrea C., Lillie Erin, Zarin Wasifa, O'Brien Kelly K., Colquhoun Heather, Levac Danielle, Moher David, Peters Micah D.J., Horsley Tanya, Weeks Laura, Hempel Susanne, Akl Elie A., Chang Christine, McGowan Jessie, Stewart Lesley, Hartling Lisa, Aldcroft Adrian, Wilson Michael G., Garritty Chantelle, Lewin Simon, Godfrey Christina M., Macdonald Marilyn T., Langlois Etienne V., Soares-Weiser Karla, Moriarty Jo, Clifford Tammy, Tunçalp Özge, Straus Sharon E. (2018). PRISMA Extension for Scoping Reviews (PRISMA-ScR): Checklist and Explanation. Annals of Internal Medicine.

[36] Joanna Briggs Institute. The Joanna Briggs Institute Reviewers’ Manual 2015: Methodology for JBI scoping reviews. Australia: The Joanna Briggs Institute; 2015.

[37] Partners E (2016). DistillerSR: systematic review and literature review software.

[38] Hsieh H-F, Shannon SE (2005). Three approaches to qualitative content analysis. Qual Health Res.

[39] Cavanagh S (1997). Content analysis: concepts, methods and applications. Nurs Res.

[40] Hodgkin JE, Sachs DP, Swan GE, Jack LM, Titus BL, Waldron SJ (2013). Outcomes from a patient-centered residential treatment plan for tobacco dependence. Mayo Clin Proc.

[41] Finkelstein N, Markoff LS (2004). The women embracing life and living (WELL) project: using the relational model to develop integrated systems of care for women with alcohol/drug use and mental health disorders with histories of violence. Alcohol Treat Q.

[42] Covington SS (2008). Women and addiction: a trauma-informed approach. J Psychoactive Drugs.

[43] Jonsdottir H, Jonsdottir R, Geirsdottir T, Sigridur Sveinsdottir K, Sigurdardottir T (2004). Multicomponent individualized smoking cessation intervention for patients with lung disease. J Adv Nurs.

[44] Zweben JE, Moses Y, Cohen JB, Price G, Chapman W, Lamb J (2015). Enhancing Family Protective Factors in Residential Treatment for Substance Use Disorders. Child Welfare.

[45] Registered Nurses Association of Ontario. Supporting Clients on Methadone Maintenance Treatment. Ontario: Registered Nurses’ Association of Ontario; 2009.

[46] Brands B, Blake J, Marsh D (2003). Impact of methadone program philosophy changes on early treatment outcomes. J Addict Dis.

[47] Cacchione PZ, Eible L, Gill LRL, Pluege SF (2016). Person-centered Care for Older Adults with Serious Mental Illness and Substance Misuse within a program of all-inclusive Care for the Elderly. J Gerontol Nurs.

[48] Krampe H, Ehrenreich H (2012). Therapeutic alliance and multiple psychotherapy in the context of therapist rotation: experiences with OLITA. Neurol Psychiatry Brain Res.

[49] Krampe H, Stawicki S, Hoehe MR, Ehrenreich H (2007). Outpatient long-term intensive therapy for alcoholics (OLITA): a successful biopsychosocial approach to the treatment of alcoholism. Dialogues Clin Neurosci.

[50] Timko C, Schultz NR, Britt J, Cucciare MA (2016). Transitioning from detoxification to substance use disorder treatment: facilitators and barriers. J Subst Abus Treat.

[51] McCallum SL, Mikocka-Walus AA, Gaughwin MD, Andrews JM, Turnbull DA (2016). ‘I’m a sick person, not a bad person’: patient experiences of treatments for alcohol use disorders. Health expectations.

[52] Abou-Saleh MT, Tarter RE, Salloum IM. Substance use disorders. Person Centered Psychiatry. 2016;325:325–43.

[53] Blankertz L, Magura S, Staines GL, Madison EM, Spinelli M, Horowitz E (2004). A new work placement model for unemployed methadone maintenance patients. Subst Use Misuse..

[54] Browne AJ, Shultis JD, Thio-Watts M (1999). Solution-focused approaches to tobacco reduction with disadvantaged prenatal clients. J Community Health Nurs.

[55] Tanney MR, Lowenstein V (1997). One-stop shopping: description of a model program to provide primary care to substance-abusing women and their children. J Pediatr Healthcare.

[56] Shin HC, Marsh JC, Cao DC, Andrews CM (2011). Client-provider relationship in comprehensive substance abuse treatment: differences in residential and nonresidential settings. J Subst Abus Treat.

[57] Borge L, Rossberg JI, Sverdrup S (2013). Cognitive milieu therapy and physical activity: experiences of mastery and learning among patients with dual diagnosis. J Psychiatr Ment Health Nurs.

[58] British Columbia Centre on Substance Use (2018). Treatment of Opioid Use Disorder During Pregnancy-Guideline Supplement.

[59] Registered Nurses Association of Ontario (2018). Implementing Supervised Injection Services.

[60] Norrish ME, Jooste K (2001). Nursing care of the patient undergoing alcohol detoxification. Curationis..

[61] Korthuis PT, Gregg J, Rogers WE, McCarty D, Nicolaidis C, Boverman J (2010). Patients’ reasons for choosing office-based buprenorphine: preference for patient-centered care. J Addict Med.

[62] Banazadeh N, Kheradmand A, Abedi H (2009). Opiate dependents’ experiences of the therapeutic relationship in methadone centers; a qualitative study. Addict Health.

[63] Fox AD, Masyukova M, Cunningham CO (2016). Optimizing psychosocial support during office-based buprenorphine treatment in primary care: Patients’ experiences and preferences. Subst Abus.

[64] Yang JA, Ashtari N, Kyupelyan L, Runyan AM (2016). Treatment of an older adult with borderline personality disorder and prescription opioid abuse. Clin Case Stud.

[65] Lefebvre L, Midmer D, Boyd JA, Ordean A, Graves L, Kahan M (2010). Participant perception of an integrated program for substance abuse in pregnancy. J Obstetr Gynecol Neonatal Nurs.

[66] Hillen P, Cree VE, Jain S (2014). Facilitating Recovery from Drug and Alcohol Problems — Reflections on Interviews with Service Users in Scotland. Practice.

[67] Nelson EC, Lazar J (2015). Mollie's story: a case and a place that exemplify person-centered care. J Ambul Care Manage.

[68] Ahrens MP (1998). A model for dual disorder treatment in acute psychiatry in a VA population. J Subst Abus Treat.

[69] Alexander JA, Nahra TA, Lemak CH, Pollack H, Campbell CI (2008). Tailored treatment in the outpatient substance abuse treatment sector: 1995-2005. J Subst Abus Treat.

[70] Yarborough BJH, Chi FW, Green CA, Hinman A, Mertens J, Beck A (2018). Patient and System Characteristics Associated with Performance on the HEDIS Measures of Alcohol and Other Drug Treatment Initiation and Engagement. J Addict Med.

[71] Lafave L, Desportes L, McBride C (2008). Treatment outcomes and perceived benefits: a qualitative and quantitative assessment of a women's substance abuse treatment program. Women Ther.

[72] Van Horn DHA, Drapkin M, Lynch KG, Rennert L, Goodman JD, Thomas T (2015). Treatment choices and subsequent attendance by substance-dependent patients who disengage from intensive outpatient treatment. Addict Res Theory.

[73] Joosten EAG, De Jong CAJ, de Weert-van Oene GH, Sensky T, van der Staak CPF (2011). Shared decision-making: increases autonomy in substance-dependent patients. Subst Use Misuse.

[74] Lawn S, Pols RG, Battersby MW (2009). Working effectively with patients with comorbid mental illness and substance abuse: a case study using a structured motivational behavioural approach. BMJ Case Rep.

[75] Miller SD, Mee-Lee D, Plum W, Hubble MA, Lebow JL, Lebow JL (2005). Making treatment count: client-directed, outcome-informed clinical work with problem drinkers. Handbook of clinical family therapy.

[76] Owens C, Springett J (2006). The Roy Castle fag ends stop smoking service: a successful client-led approach to smoking cessation. J Smok Cessat.

[77] Pichot T (2001). Co-creating solutions for substance abuse. J Sys Ther.

[78] Campbell BK, Fuller BE, Lee ES, Woelfel T, Robinson J, McCarty D (2009). Facilitating outpatient treatment entry following detoxification for injection drug use: a multisite test of three interventions. Psychol Addict Behav.

[79] Vakharia SP, Little J (2017). Starting where the client is: harm reduction guidelines for clinical social work practice. Clin Soc Work J.

[80] Joosten EAG, De Jong CAJ, De Weert-van Oene GH, Sensky T, van der Staak CPF (2009). Shared decision-making reduces drug use and psychiatric severity in substance-dependent patients. Psychother Psychosom.

[81] Joosten EAG, de Weert GH, Sensky T, van der Staak CPF, de Jong CAJ (2008). Effect of shared decision-making on therapeutic alliance in addiction health care. Patient Prefer Adherence.

[82] Stankiewicz Murphy L, Oros MT, Dorsey SG (2014). The Baltimore buprenorphine initiative: understanding the role of buprenorphine in addressing heroin addiction in an urban-based community. J Addict Nurs.

[83] Cermak TL (2015). Clinical approach to the heavy Cannabis user in the age of medical marijuana. J Psychoactive Drugs.

[84] Hammer G. Trust and relationship as cornerstones of successful psychotherapy. Harm Reduction Psychother. 2002;106:106–35.

[85] Diamond GM, Liddle HA, Hogue A, Dakof GA (1999). Alliance-building interventions with adolescents in family therapy: A process study. Psychotherapy.

[86] Blonigen DM, Timko C, Jacob T, Moos RH (2015). Patient-centered feedback on the results of personality testing increases early engagement in residential substance use disorder treatment: a pilot randomized controlled trial. Addict Sci Clin Pract.

[87] Bradley KA, Bobb JF, Ludman EJ, Chavez LJ, Saxon AJ, Merrill JO (2018). Alcohol-related nurse Care Management in Primary Care: a randomized clinical trial. JAMA Intern Med.

[88] Registered Nurses Association of Ontario (2017). Integrating Tobacco Interventions into Daily Practice.

[89] Newman CF (1997). Establishing and maintaining a therapeutic alliance with substance abuse patients: a cognitive therapy approach. NIDA Res Monogr.

[90] Van Bilsen HP, Van Emst AJ (1986). Heroin addiction and motivational milieu therapy. Int J Addict.

[91] Mena JA, Ampadu GG, Prochaska JO (2017). The influence of engagement and satisfaction on smoking cessation interventions: a qualitative study. Subst Use Misuse.

[92] Mericle AA, Casaletto K, Knoblach D, Brooks AC, Carise D (2009). Barriers to implementing individualized substance Abuse treatment: qualitative findings from the CASPAR replication studies. J Drug Issues.

[93] Bobb JF, Lee AK, Lapham GT, Oliver M, Ludman E, Achtmeyer C (2017). Evaluation of a pilot implementation to integrate alcohol-related care within primary care. Int J Environ Res Public Health.

[94] O’Sullivan GA, Hanlon C, Dentry T, Morris T, Banting L (2018). A qualitative exploration of the client experience of inter-professional practice in the delivery of ActivePlus: a combined smoking cessation and physical activity intervention. BMC Health Serv Res.

[95] Ness O, Borg M, Semb R, Karlsson B. “Walking alongside:” Collaborative practices in mental health and substance use care. Int J Ment Health Sys. 2014;8:1–8. 10.1186/1752-4458-8-55 PMC4276107 25540670

[96] Redko C, Rapp RC, Elms C, Snyder M, Carlson RG (2007). Understanding the working alliance between persons with substance abuse problems and strengths-based case managers. J Psychoactive Drugs.

[97] Sohler NL, Li X, Kunins HV, Sacajiu G, Giovanniello A, Whitley S (2010). Home- versus office-based buprenorphine inductions for opioid-dependent patients. J Subst Abus Treat.

[98] Andersson HW, Otterholt E, Grawe RW (2017). Patient satisfaction with treatments and outcomes in residential addiction institutions. Nord Stud Alcohol Drugs.

[99] Woolhouse S, Brown JB, Thind A (2014). ‘Meeting people where they’re at’: experiences of family physicians engaging women who use illicit drugs. Ann Fam Med.

[100] Luborsky L, McLellan AT, Woody GE, O'Brien CP, Auerbach A (1985). Therapist success and its determinants. Arch Gen Psychiatry.

[101] Slater L (2006). Person-centredness: a concept analysis. Contemp Nurse.

[102] Lafave L, Desportes L, McBride C (2009). Treatment outcomes and perceived benefits: a qualitative and quantitative assessment of a women's substance abuse treatment program. Women Ther.

[103] Abrahamsson A, Springett J, Karlsson L, Håkansson A, Ottosson T (2005). Some lessons from Swedish midwives’ experiences of approaching women smokers in antenatal care. Midwifery..

[104] Morel A (2010). Place of psychotherapy in therapeutic accompaniment in addictology. Theory Pract Psychotropes.

[105] Yang LH, Wong LY, Grivel MM, Hasin DS (2017). Stigma and substance use disorders: an international phenomenon. Curr Opin Psychiatry.

[106] Marchand K, Palis H, Oviedo-Joekes E (2016). Patient perceptions of prejudice and discrimination by health care providers and its relationship with mental disorders: results from the 2012 Canadian community health-mental health survey data. Community Ment Health J.

[107] van Boekel LC, Brouwers EP, van Weeghel J, Garretsen HF (2013). Stigma among health professionals towards patients with substance use disorders and its consequences for healthcare delivery: systematic review. Drug Alcohol Depend.

[108] Stewart M, Brown JB, Weston WW, Freeman TR (2003). Patient-Centred medicine: transforming the clinical method.

[109] Elwyn G, Frosch D, Thomson R, Joseph-Williams N, Lloyd A, Kinnersley P (2012). Shared decision making: a model for clinical practice. J Gen Intern Med.

[110] McMillan SS, Kendall E, Sav A, King MA, Whitty JA, Kelly F (2013). Patient-centered approaches to health care: a systematic review of randomized controlled trials. Med Care Res Rev.

[111] Damschroder LJ, Hagedorn HJ (2011). A guiding framework and approach for implementation research in substance use disorders treatment. Psychol Addict Behav.

[112] Santana MJ, Manalili K, Jolley RJ, Zelinsky S, Quan H, Lu M (2018). How to practice person-centred care: a conceptual framework. Health Expect.

[113] Rathert C, Wyrwich Md Fau - Boren SA, Boren SA. Patient-centered care and outcomes: a systematic review of the literature. Med Care Res Rev. 2013;70(4):351–79. 10.1177/1077558712465774 23169897

[114] Dwamena F, Holmes-Rovner M, Gaulden CM, Jorgenson S, Sadigh G, Sikorskii A (2012). Interventions for providers to promote a patient-centred approach in clinical consultations. Cochrane Database Syst Rev.

[115] Tiffany ST, Friedman L, Greenfield SF, Hasin DS, Jackson R (2012). Beyond drug use: a systematic consideration of other outcomes in evaluations of treatments for substance use disorders. Addiction..

[116] Neale J, Tompkins C, Wheeler C, Finch E, Marsden J, Mitcheson L (2015). “You’re all going to hate the word ‘recovery’ by the end of this”: service users’ views of measuring addiction recovery. Drugs.

[117] Trujols J, Portella MJ, Iraurgi I, Campins MJ, Sinol N, de Los Cobos JP (2013). Patient-reported outcome measures: are they patient-generated, patient-centred or patient-valued?. J Ment Health.

[118] Godlaski TM, Butler L, Heron M, Debord S, Cauvin L (2009). A qualitative exploration of engagement among rural women entering substance user treatment. Subst Use Misuse.

[119] Allen RS, Olson BD (2016). The what and why of effective substance abuse treatment. Int J Ment Heal Addict.

[120] Kavanagh DJ, Connolly JM (2007). Motivational interviewing. Clinical handbook of co-existing mental health and drug and alcohol problems.

[121] Bryant-Jefferies R. A person centred approach to understanding and helping people with a dual diagnosis. Dual diagnosis nursing. 2006;240:240–52.

[122] McMahon J (2009). Hard to reach and impossible to help: working at the rough end of social care. J Soc Work Pract.

[123] Rance J, Treloar C (2015). “We are people too”: consumer participation and the potential transformation of therapeutic relations within drug treatment. Int J Drug Policy.

[124] Rance J, Treloar C, Ethos Study Group (2014). ‘Not just methadone Tracy’: transformations in service-user identity following the introduction of hepatitis C treatment into Australian opiate substitution settings. Addiction.

[125] Chorlton E, Smith I, Jones SA (2015). Understanding how people who use illicit drugs and alcohol experience relationships with psychiatric inpatient staff. Soc Psychiatry Psychiatr Epidemiol.

[126] Guichenez P (2009). Establishment of a therapeutic alliance in smoking cessation within the framework of behavioral and cognitive therapies. Rev Mal Respir.

[127] Brown RL, Pfeifer JM, Gjerde CL, Seibert CS, Haq CL (2004). Teaching patient-centered tobacco intervention to first-year medical students. J Gen Intern Med.

[128] Luborsky L, Barber JP, Siqueland L, McLellan AT, Woody G (1997). Establishing a therapeutic alliance with substance abusers. NIDA Res Monogr.

[129] Ness O, Kvello O, Borg M, Semb R, Davidson L (2017). “Sorting things out together”: Young adults’ experiences of collaborative practices in mental health and substance use care. Am J Psychiatr Rehabil.

[130] Van Hout MC, Bingham T (2014). A qualitative study of prescribing doctor experiences of methadone maintenance treatment. Int J Ment Heal Addict.

[131] Moyers TB, Miller WR (2013). Is low therapist empathy toxic?. Psychol Addict Behav.

[132] Miller WR, Benefield RG, Tonigan JS (1993). Enhancing motivation for change in problem drinking: a controlled comparison of two therapist styles. J Consult Clin Psychol.

[133] Marcellus L (2014). Supporting women with substance use issues: trauma-informed care as a foundation for practice in the NICU. Neonatal Netw.

[134] Gatz M, Brown V, Hennigan K, Rechberger E, O'Keefe M, Rose T (2007). Effectiveness of an integrated, trauma-informed approach to treating women with co-occurring disorders and histories of trauma: the Los Angeles site experience. J Community Psychol.

[135] Shier ML, Turpin A (2017). A multi-dimensional conceptual framework for trauma-informed practice in addictions programming. J Soc Serv Res.

[136] Fisher DG, Lankford BA, Galea RP (1996). Therapeutic community retention among Alaska natives: Akeela house. J Subst Abus Treat.

[137] Matthews AK, Conrad M, Kuhns L, Vargas M, King AC (2013). Project exhale: preliminary evaluation of a tailored smoking cessation treatment for HIV-positive African American smokers. AIDS Patient Care STDs.

[138] Johnson TM, Fenton BJ, Kracht BR, Weiner MF, Guggenheim FG (1988). Providing culturally sensitive care: intervention by a consultation-liaison team. Hosp Community Psychiatry.

