# Supplementary material for: Conceptualizing patient-centered care for substance use disorder treatment: findings from a systematic scoping review
Source: Subst Abuse Treat Prev Policy. 2019 Sep 11;14:37. doi: 10.1186/s13011-019-0227-0 (PMC6739978; doi:10.1186/s13011-019-0227-0)
Supplement: Supplementary file 2 — Sample Ovid Medline search terms. (PDF 470 kb) [file 13011_2019_227_MOESM2_ESM.pdf]

Appendix A

Database-Specific Search Strategies

Table A1. Medline (Ovid) search strategy

Search term/line number	Conceptual term of interest	Other keywords/synonyms and notes	Search term entered into Ovid-Medline	Number of hits
1	Substance-Related Disorders	exp substance-related disorders/ or exp alcohol-related disorders/ or exp amphetamine-related disorders/ or exp cocaine-related disorders/ or exp drug overdose/ or exp heroin dependence/ or exp inhalant abuse/ or exp marijuana abuse/ or exp neonatal abstinence syndrome/ or exp opioid-related disorders/ or exp phencyclidine abuse/ or exp psychoses, substance-induced/ or exp substance abuse, intravenous/ or exp substance abuse, oral/ or exp substance withdrawal syndrome/ or exp "tobacco use disorder"/	exp Substance-Related Disorders	258462
2	Street Drugs	exp street drugs/ or exp crack cocaine/	exp Street Drugs	11263
3	Substance abuser	substance abusing	substance abus*	49532
4	Substance dependent	Substance dependency	substance dependen*	2720
5	Substance misuse	Substance misusers, substance misusing	substance misus*	2150
6	Problematic substance use	problematic substance users, people with problematic substance use, problematic substance using	Problematic substance adj2 (use* or usage or using)	254

7	People who inject drugs	PWID	(people who inject drug* or PWID)	1740
8	Injection drug use	IDU, injection drug users	injection drug "(use or user or usage or using)" or IDU	2794
9	People who use drugs	PWUD	"people who use drugs" or PWUD	343
10	Illicit substance use	Illicit substance using, illicit substance use, illegal substance users, illegal substance using, illegal substance use, street substance users, street substance using, street substance use	(illicit or street or illegal) adj2 (drug or substance) adj2 (use* or usage or using)	5848
11	opioid dependence	opioid dependent, opioid dependency, opioid addiction, opioid abuse, opioid misuse, opioid problem	opioid adj2 (addict* or abus* or dependen* or misus* or problem* or disorder*)	15537
12	opiate dependence	opiate dependent, opiate dependency, opiate addiction, opiate abuse, opiate misuse, opiate problem	opiate adj2 (addict* or abus* or dependen* or misus* or problem* or disorder*)	3475
13	narcotic dependence	narcotic dependent, narcotic dependency, narcotic addiction, narcotic abuse, narcotic misuse, narcotic problem	narcotic adj2 (addict* or abus* or dependen* or misus* or problem* or disorder*)	1279
14	heroin	None	heroin	17865
15	stimulant use disorder		"stimulant use disorder"	40
16	exp crack cocaine	MeSH exploded; this is a separate Mesh from cocaine-related disorders and reflects addiction to crack cocaine	exp crack cocaine	1343

17	exp cocaine smoking	Mesh exploded; this is a separate Mesh from cocaine-related disorders and crack cocaine	exp cocaine smoking	4
18	cocaine dependence	cocaine dependent, cocaine dependency, cocaine addiction, cocaine abuse, cocaine dependence, cocaine misuse, cocaine problem	((cocaine or crack) adj2 (addict* or abus* or dependen* or misus* or problem* or disorder*))	11806
19	amphetamine dependence	amphetamine dependent, dependency, addiction, abuse, dependence, misuse, problem; crystal methamphetamine…; crystal meth….;	(amphetamine or crystal methamphetamine or crystal meth) adj1 (addict* or abus* or dependen* or misus* or problem* or disorder*)	592
20	alcohol dependence	alcohol dependent, alcohol dependency, alcohol addiction, alcohol abuse, alcohol miuse, alcohol problem	alcohol adj2 (addict* or abus* or dependen* or misus* or problem* or disorder*)	50104
21	cannabis dependence	cannabis dependent, cannabis dependency, cannabis abuse, cannabis addiction	cannabis adj2 (addict* or abus* or dependen* or disorder*)	1917
22	tobacco dependence	tobacco or nicotine dependent, tobacco or nicotine dependency, smoking cessation	(tobacco or nicotine or smok*) adj2 (dependen* or disorder* or cessation)	45598
23	Diagnosis, Dual (Psychiatry)	MeSH unexploded	Diagnosis, Dual (Psychiatry)	3372
24		(all problematic substance use related terms)	1 or 2 or 3 or 4 or 5 or 6 or 7 or 8 or 9 or	337412

			10 or 11 or 12 or 13 or 14 or 15 or 16 or 17 or 18 or 19 or 20 or 21 or 22 or 23	
25	exp Patient-Centered Care	MeSH Exploded includes all sub-categories	exp Patient-Centered Care	16653
26	Patient centered care	patient centered treatment, patient centered therapy, Client centered care, client centered treatment, client centered therapy, Person-Centered Care, person centered treatment, person centered therapy	((patient or client or person) adj1 cent?red adj1 (care or treatment* or therap*))	20713
27	Patient focused care	Client focused care, person focused care	((patient or client or person) adj1 focus?ed adj1 (care or treatment* or therap*))	425
28	Patient participation in treatment planning, process, decisions	Patient/client autonomy in decision making/care/practice/treatment/planning Patient/client involvement in decision making/care/practice/treatment/planning Patient/client control in decision making/care/practice/treatment/planning Patient/client empowerment in decision making/care/practice/treatment/planning	(patient or client) adj1 (autonom* or involve* or control or empower*) adj1 (decision making or care or practic* or treatment* or plan*)	126
29	Collaborative care	Collaborative practices, collaborative treatment, collaborative care planning	collaborative adj1 (care or practic* or treatment* or plan*)	3803
30	Shared decision-making	shared decision making, joint decision making	(shared or joint or collaborative) adj2 decision making	6279
31	Therapeutic alliance	No mesh term, as a subject heading crossed with substance related disorders it results in 153 hits.	therapeutic alliance	9027

32	Relational practice		relational practic*	44
33	Enhanced patient-provider relationship	physician/doctor/nurse/professional/provider-patient/client alliance/empowerment/support/enhance	(physician or doctor or nurse or professional or provider) adj1 (patient or client) adj1 (enhanc* or alliance* or empower* or support*)	304
34	Enhanced patient-provider communication	physician/doctor/nurse/professional/provider-patient/client communication	(physician or doctor or nurse or professional or provider) adj1 (patient or client) adj1 communicat*	4230
35	Holistic Nursing	holistic nursing care	Holistic Nursing/	3135
36	Trauma informed care	trauma-informed, trauma informed, trauma centered with care, approach, treatment or therapy	trauma adj1 (cent?red or informed) adj1 (care or approach or treatment* or therap*)	263
37	Culturally safe care	culturally sensitive care	cultural* adj1 (safe or sensitive) adj1 (care or approach or treatment* or therap*)	395
39	Family Systems Nursing		Family Systems Nursing	64
39	Expert patient program	expert patient programme	expert patient program*	27

40		(All patient-centered care related terms)	25 or 26 or 27 or 28 or 29 or 30 or 31 or 32 or 33 or 34 or 35 or 36 or 37 or 38 or 39	40281
41			**24 and 40**	**896**

Table A2. Embase (Ovid) search strategy

**Search term/line number**	**Conceptual term of interest**	**Other keywords/synonyms and notes**	**Search term entered into Ovid-Embase**	**Number of hits**
1	Substance-Related Disorders	exp substance-related disorders/ or exp alcohol-related disorders/ or exp amphetamine-related disorders/ or exp cocaine-related disorders/ or exp drug overdose/ or exp heroin dependence/ or exp inhalant abuse/ or exp marijuana abuse/ or exp neonatal abstinence syndrome/ or exp opioid-related disorders/ or exp phencyclidine abuse/ or exp psychoses, substance-induced/ or exp substance abuse, intravenous/ or exp substance abuse, oral/ or exp substance withdrawal syndrome/ or exp "tobacco use disorder"/	exp Drug Dependence/	217291
2	Street Drugs	exp street drugs/ or exp crack cocaine/	exp Street drug/	3375
3	Substance abuser	substance abusing	substance abus*	65379
4	Substance dependent	Substance dependency	substance dependen*	3853
5	Substance misuse	Substance misusers, substance misusing	substance misus*	3235

6	Problematic substance use	problematic substance users, people with problematic substance use, problematic substance using	Problematic substance adj2 (use* or usage or using)	321
7	People who inject drugs	PWID	(people who inject drug* or PWID)	2225
8	Injection drug use	IDU, injection drug users	injection drug "(use or user or usage or using)" or IDU	3877
9	People who use drugs	PWUD	"people who use drugs" or PWUD	443
10	Illicit substance use	Illicit substance using, illicit substance use, illegal substance users, illegal substance using, illegal substance use, street substance users, street substance using, street substance use	(illicit or street or illegal) adj2 (drug or substance) adj2 (use* or usage or using)	7836
11	opioid dependence	opioid dependent, opioid dependency, opioid addiction, opioid abuse, opioid misuse, opioid problem	opioid adj2 (addict* or abus* or dependen* or misus* or problem* or disorder*)	10903
12	opiate dependence	opiate dependent, opiate dependency, opiate addiction, opiate abuse, opiate misuse, opiate problem	opiate adj2 (addict* or abus* or dependen* or misus* or problem* or disorder*)	17604
13	narcotic dependence	narcotic dependent, narcotic dependency, narcotic addiction, narcotic abuse, narcotic misuse, narcotic problem	narcotic adj2 (addict* or abus* or dependen* or misus* or problem* or disorder*)	2539

14	heroin	None	heroin	21066
15	stimulant use disorder		"stimulant use disorder"	62
16	exp crack cocaine	MeSH exploded; this is a separate Mesh from cocaine-related disorders and reflects addiction to crack cocaine	exp crack cocaine	52870
17	exp cocaine smoking	Mesh exploded; this is a separate Mesh from cocaine-related disorders and crack cocaine	"cocaine smoking"	126
18	cocaine dependence	cocaine dependent, cocaine dependency, cocaine addiction, cocaine abuse, cocaine dependence, cocaine misuse, cocaine problem	((cocaine or crack) adj2 (addict* or abus* or dependen* or misus* or problem* or disorder*))	16798
19	amphetamine dependence	amphetamine dependent, dependency, addiction, abuse, dependence, misuse, problem; crystal methamphetamine…; crystal meth….;	(amphetamine or crystal methamphetamine or crystal meth) adj1 (addict* or abus* or dependen* or misus* or problem* or disorder*)	1007
20	alcohol dependence	alcohol dependent, alcohol dependency, alcohol addiction, alcohol abuse, alcohol miuse, alcohol problem	alcohol adj2 (addict* or abus* or dependen* or misus* or problem* or disorder*)	78110
21	cannabis dependence	cannabis dependent, cannabis dependency, cannabis abuse, cannabis addiction	cannabis adj2 (addict* or abus* or	9686

			dependen* or disorder*)	
22	tobacco dependence	tobacco or nicotine dependent, tobacco or nicotine dependency, smoking cessation	(tobacco or nicotine or smok*) adj2 (dependen* or disorder* or cessation)	72203
23	Diagnosis, Dual (Psychiatry)		dual diagnosis	2515
24		(All problematic substance use related terms)	1 or 2 or 3 or 4 or 5 or 6 or 7 or 8 or 9 or 10 or 11 or 12 or 13 or 14 or 15 or 16 or 17 or 18 or 19 or 20 or 21 or 22 or 23	402117
25	exp Patient-Centered Care		patient-centered care	4909
26	Patient centered care	patient centered treatment, patient centered therapy, Client centered care, client centered treatment, client centered therapy, Person-Centered Care, person centered treatment, person centered therapy	((patient or client or person) adj1 cent?red adj1 (care or treatment* or therap*))	9174
27	Patient focused care	Client focused care, person focused care	((patient or client or person) adj1 focus?ed adj1 (care or treatment* or therap*))	516
28	Patient participation in	Patient/client autonomy in decision making/care/practice/treatment/planning Patient/client involvement in decision	(patient or client) adj1 (autonom* or involve* or control or empower*) adj1	157

	treatment planning, process, decisions	making/care/practice/treatment/planning Patient/client control in decision making/care/practice/treatment/planning Patient/client empowerment in decision making/care/practice/treatment/planning	(decision making or care or practic* or treatment* or plan*)	
29	Collaborative care	Collaborative practices, collaborative treatment, collaborative care planning	collaborative adj1 (care or practic* or treatment* or plan*)	4994
30	Shared decision-making	shared decision making, joint decision making	(shared or joint or collaborative) adj2 decision making	9413
31	Therapeutic alliance		therapeutic alliance	3116
32	Relational practice		relational practic*	47
33	Enhanced patient-provider relationship	physician/doctor/nurse/professional/provider-patient/client alliance/empowerment/support/enhance	(physician or doctor or nurse or professional or provider) adj1 (patient or client) adj1 (enhanc* or alliance* or empower* or support*)	371
34	Enhanced patient-provider communication	physician/doctor/nurse/professional/provider-patient/client communication	(physician or doctor or nurse or professional or provider) adj1 (patient or client) adj1 communicat*	6051
35	Holistic Nursing	holistic nursing care	Holistic Nursing /	3117

36	Trauma informed care	trauma-informed, trauma informed, trauma centered with care, approach, treatment or therapy	trauma adj1 (cent?red or informed) adj1 (care or approach or treatment* or therap*)	294
37	Culturally safe care	culturally sensitive care	cultural* adj1 (safe or sensitive) adj1 (care or approach or treatment* or therap*)	479
39	Family Systems Nursing		Family Systems Nursing	58
39	Expert patient program	expert patient programme	expert patient program*	42
40		(All patient-centered care related terms)	25 or 26 or 27 or 28 or 29 or 30 or 31 or 32 or 33 or 34 or 35 or 36 or 37 or 38 or 39	36068
41			**24 and 40**	1072

Table A3. PsycINFO (EBSCO) search strategy

Search term/line number	Conceptual term of interest	Other keywords/synonyms and notes	Search term entered into PsycINFO (EBSCO)	Number of hits
1	Substance-Related Disorders	exp substance-related disorders/ or exp alcohol-related disorders/ or exp amphetamine-related disorders/ or exp cocaine-related disorders/ or exp drug overdose/ or exp heroin dependence/ or exp inhalant abuse/ or exp marijuana abuse/ or exp neonatal abstinence syndrome/ or exp opioid-related disorders/ or exp phencyclidine abuse/ or exp psychoses, substance-induced/ or exp substance abuse, intravenous/ or exp substance abuse, oral/ or exp substance withdrawal syndrome/ or exp "tobacco use disorder"/	DE "Substance Use Disorder"	5661
2	Street Drugs	exp street drugs/ or exp crack cocaine/	street drug*	3700
3	Substance abuser	substance abusing	substance abus*	9890
4	Substance dependent	Substance dependency	substance dependen*	3955
5	Substance misuse	Substance misusers, substance misusing	substance misus*	7881

6	Problematic substance use	problematic substance users, people with problematic substance use, problematic substance using	problematic substance n2 (use* or usage or using)	353
7	People who inject drugs	PWID	(people who inject drug* or PWID)	903
8	Injection drug use	IDU, injection drug users	injection drug "(use or user or usage or using)" or IDU	2128
9	People who use drugs	PWUD	"people who use drugs" or PWUD	245
10	Illicit substance use	Illicit substance using, illicit substance use, illegal substance users, illegal substance using, illegal substance use, street substance users, street substance using, street substance use	(illicit or street or illegal) n2 (drug or substance) n2 (use* or usage or using)	6874
11	opioid dependence	opioid dependent, opioid dependency, opioid addiction, opioid abuse, opioid misuse, opioid problem	opioid n2 (addict* or abus* or dependen* or misus* or problem* or disorder*)	7697
12	opiate dependence	opiate dependent, opiate dependency, opiate addiction, opiate abuse, opiate misuse, opiate problem	opiate n2 (addict* or abus* or dependen* or misus* or problem* or disorder*)	2872
13	narcotic dependence	narcotic dependent, narcotic dependency, narcotic addiction, narcotic abuse, narcotic misuse, narcotic problem	narcotic n2 (addict* or abus* or dependen* or misus* or problem* or disorder*)	965

14	heroin	None	heroin	9769
15	stimulant use disorder		"stimulant use disorder"	41
16	exp crack cocaine	MeSH exploded; this is a separate Mesh from cocaine-related disorders and reflects addiction to crack cocaine	DE "Crack Cocaine"	959
17	exp cocaine smoking	Mesh exploded; this is a separate Mesh from cocaine-related disorders and crack cocaine	"cocaine smoking"	43
18	cocaine dependence	cocaine dependent, cocaine dependency, cocaine addiction, cocaine abuse, cocaine dependence, cocaine misuse, cocaine problem	((cocaine or crack) n2 (addict* or abus* or dependen* or misus* or problem* or disorder*))	8439
19	amphetamine dependence	amphetamine dependent, dependency, addiction, abuse, dependence, misuse, problem; crystal methamphetamine…; crystal meth….;	(amphetamine or crystal methamphetamine or crystal meth) n1 (addict* or abus* or dependen* or misus* or problem* or disorder*)	1921
20	alcohol dependence	alcohol dependent, alcohol dependency, alcohol addiction, alcohol abuse, alcohol miuse, alcohol problem	alcohol n2 (addict* or abus* or dependen* or misus* or problem* or disorder*)	65796
21	cannabis dependence	cannabis dependent, cannabis dependency, cannabis abuse, cannabis addiction	cannabis n2 (addict* or abus* or dependen* or disorder*)	2196

22	tobacco dependence	tobacco or nicotine dependent, tobacco or nicotine dependency, smoking cessation	(tobacco or nicotine or smok*) n2 (dependen* or disorder* or cessation)	22659
23	Diagnosis, Dual (Psychiatry)		DE "Dual Diagnosis"	1971
24		(All problematic substance use related terms)	1 or 2 or 3 or 4 or 5 or 6 or 7 or 8 or 9 or 10 or 11 or 12 or 13 or 14 or 15 or 16 or 17 or 18 or 19 or 20 or 21 or 22 or 23	172693
25	exp Patient-Centered Care		DE "Client Centered Therapy"	3092
26	Patient centered care	patient centered treatment, patient centered therapy, Client centered care, client centered treatment, client centered therapy, Person-Centered Care, person centered treatment, person centered therapy	((patient or client or person) n1 cent?#red n1 (care or treatment* or therap*))	11577
27	Patient focused care	Client focused care, person focused care	((patient or client or person) n1 focus#ed n1 (care or treatment* or therap*))	129
28	Patient participation in treatment planning, process, decisions	Patient/client autonomy in decision making/care/practice/treatment/planning Patient/client involvement in decision making/care/practice/treatment/planning Patient/client control in decision	(patient or client) n1 (autonom* or involve* or control or empower*) n1 (decision making or	757

		making/care/practice/treatment/planning Patient/client empowerment in decision making/care/practice/treatment/planning	care or practic* or treatment* or plan*)	
29	Collaborative care	Collaborative practices, collaborative treatment, collaborative care planning	collaborative (care or practic* or treatment* or plan*)	3665
30	Shared decision-making	shared decision making, joint decision making	(shared or joint or collaborative) n2 decision making	3010
31	Therapeutic alliance		DE "Therapeutic Alliance"	4339
32	Relational practice		"relational practic*"	200
33	Enhanced patient-provider relationship	physician/doctor/nurse/professional/provider-patient/client alliance/empowerment/support/enhance	(physician or doctor or nurse or professional or provider) n1 (patient or client) n1 (enhanc* or alliance* or empower* or support*)	413
34	Enhanced patient-provider communication	physician/doctor/nurse/professional/provider-patient/client communication	(physician or doctor or nurse or professional or provider) n1 (patient or client) n1 communicat*	2804
35	Holistic Nursing	holistic nursing care	holistic nursing	299

36	Trauma informed care	trauma-informed, trauma informed, trauma centered with care, approach, treatment or therapy	trauma n1 (cent#red or informed) n1 (care or approach or treatment* or therap*)	511
37	Culturally safe care	culturally sensitive care	cultural* n1 (safe or sensitive) n1 (care or approach or treatment* or therap*)	808
39	Family Systems Nursing		"Family Systems Nursing"	58
39	Expert patient program	expert patient programme	"expert patient program*"	9
40		(All patient-centered care related terms)	S25 OR S26 OR S27 OR S28 OR S29 OR S30 OR S31 OR S32 OR S33 OR S34 OR S35 OR S36 OR S37 OR S38 OR S39	27380
41			**24 and 40**	943

Table A4. CINAHL (EBSCO) search strategy

Search term/line number	Conceptual term of interest	Other keywords/synonyms and notes	Search term entered into CINAHL (EBSCO)	Number of hits
1	Substance-Related Disorders	exp substance-related disorders/ or exp alcohol-related disorders/ or exp amphetamine-related disorders/ or exp cocaine-related disorders/ or exp drug overdose/ or exp heroin dependence/ or exp inhalant abuse/ or exp marijuana abuse/ or exp neonatal abstinence syndrome/ or exp opioid-related disorders/ or exp phencyclidine abuse/ or exp psychoses, substance-induced/ or exp substance abuse, intravenous/ or exp substance abuse, oral/ or exp substance withdrawal syndrome/ or exp "tobacco use disorder"/	(MH "Substance Use Disorders+")	133706
2	Street Drugs	exp street drugs/ or exp crack cocaine/	(MH "Street Drugs+")	4824
3	Substance abuser	substance abusing	substance abus*	45,878
4	Substance dependent	Substance dependency	substance dependen*	9939
5	Substance misuse	Substance misusers, substance misusing	substance misus*	1710

6	Problematic substance use	problematic substance users, people with problematic substance use, problematic substance using	problematic substance n2 (use* or usage or using)	227
7	People who inject drugs	PWID	(people who inject drug* or PWID)	1025
8	Injection drug use	IDU, injection drug users	injection drug "(use or user or usage or using)" or IDU	1588
9	People who use drugs	PWUD	"people who use drugs" or PWUD	208
10	Illicit substance use	Illicit substance using, illicit substance use, illegal substance users, illegal substance using, illegal substance use, street substance users, street substance using, street substance use	(illicit or street or illegal) n2 (drug or substance) n2 (use* or usage or using)	3780
11	opioid dependence	opioid dependent, opioid dependency, opioid addiction, opioid abuse, opioid misuse, opioid problem	opioid n2 (addict* or abus* or dependen* or misus* or problem* or disorder*)	4755
12	opiate dependence	opiate dependent, opiate dependency, opiate addiction, opiate abuse, opiate misuse, opiate problem	opiate n2 (addict* or abus* or dependen* or misus* or problem* or disorder*)	848
13	narcotic dependence	narcotic dependent, narcotic dependency, narcotic addiction, narcotic abuse, narcotic misuse, narcotic problem	narcotic n2 (addict* or abus* or dependen* or misus* or problem* or disorder*)	161

14	heroin	None	heroin	4940
15	stimulant use disorder		"stimulant use disorder"	20
16	exp crack cocaine	MeSH exploded; this is a separate Mesh from cocaine-related disorders and reflects addiction to crack cocaine	(MH "Crack Cocaine")	696
17	exp cocaine smoking	Mesh exploded; this is a separate Mesh from cocaine-related disorders and crack cocaine	"cocaine smoking"	23
18	cocaine dependence	cocaine dependent, cocaine dependency, cocaine addiction, cocaine abuse, cocaine dependence, cocaine misuse, cocaine problem	((cocaine or crack) n2 (addict* or abus* or dependen* or misus* or problem* or disorder*))	1763
19	amphetamine dependence	amphetamine dependent, dependency, addiction, abuse, dependence, misuse, problem; crystal methamphetamine…; crystal meth….;	(amphetamine or crystal methamphetamine or crystal meth) n1 (addict* or abus* or dependen* or misus* or problem* or disorder*)	138
20	alcohol dependence	alcohol dependent, alcohol dependency, alcohol addiction, alcohol abuse, alcohol miuse, alcohol problem	alcohol n2 (addict* or abus* or dependen* or misus* or problem* or disorder*)	24324
21	cannabis dependence	cannabis dependent, cannabis dependency, cannabis abuse, cannabis addiction	cannabis n2 (addict* or abus* or dependen* or disorder*)	1034

22	tobacco dependence	tobacco or nicotine dependent, tobacco or nicotine dependency, smoking cessation	(tobacco or nicotine or smok*) n2 (dependen* or disorder* or cessation)	24516
23	Diagnosis, Dual (Psychiatry)		(MH "Diagnosis, Dual (Psychiatry)")	2347
24		(All problematic substance use related terms)	1 or 2 or 3 or 4 or 5 or 6 or 7 or 8 or 9 or 10 or 11 or 12 or 13 or 14 or 15 or 16 or 17 or 18 or 19 or 20 or 21 or 22 or 23	165879
25	exp Patient-Centered Care		(MH "Patient Centered Care")	22967
26	Patient centered care	patient centered treatment, patient centered therapy, Client centered care, client centered treatment, client centered therapy, Person-Centered Care, person centered treatment, person centered therapy	((patient or client or person) n1 cent?#red n1 (care or treatment* or therap*))	24485
27	Patient focused care	Client focused care, person focused care	((patient or client or person) n1 focus#ed n1 (care or treatment* or therap*))	469
28	Patient participation in treatment planning, process, decisions	Patient/client autonomy in decision making/care/practice/treatment/planning Patient/client involvement in decision making/care/practice/treatment/planning Patient/client control in decision	(patient or client) n1 (autonom* or involve* or control or empower*) n1 (decision making or	1184

		making/care/practice/treatment/planning Patient/client empowerment in decision making/care/practice/treatment/planning	care or practic* or treatment* or plan*)	
29	Collaborative care	Collaborative practices, collaborative treatment, collaborative care planning	collaborative (care or practic* or treatment* or plan*)	6219
30	Shared decision-making	shared decision making, joint decision making	(shared or joint or collaborative) n2 decision making	3901
31	Therapeutic alliance		"therapeutic alliance"	930
32	Relational practice		"relational practic*"	68
33	Enhanced patient-provider relationship	physician/doctor/nurse/professional/provider-patient/client alliance/empowerment/support/enhance	(physician or doctor or nurse or professional or provider) n1 (patient or client) n1 (enhanc* or alliance* or empower* or support*)	598
34	Enhanced patient-provider communication	physician/doctor/nurse/professional/provider-patient/client communication	(physician or doctor or nurse or professional or provider) n1 (patient or client) n1 communicat*	2684
35	Holistic Nursing	holistic nursing care	(MH "Holistic Nursing")	2303

36	Trauma informed care	trauma-informed, trauma informed, trauma centered with care, approach, treatment or therapy	trauma n1 (cent#red or informed) n1 (care or approach or treatment* or therap*)	301
37	Culturally safe care	culturally sensitive care	cultural* n1 (safe or sensitive) n1 (care or approach or treatment* or therap*)	623
39	Family Systems Nursing		"Family Systems Nursing"	61
39	Expert patient program	expert patient programme	"expert patient program*"	26
40		(All patient-centered care related terms)	S25 OR S26 OR S27 OR S28 OR S29 OR S30 OR S31 OR S32 OR S33 OR S34 OR S35 OR S36 OR S37 OR S38 OR S39	41707
41			**24 or 40**	**940**

Table A5. ISI Web of Science search strategy

**Search term/line number**	**Conceptual term of interest**	**Other keywords/synonyms and notes**	**Search term entered into ISI Web of Science**	**Number of hits**
1	Substance-Related Disorders	exp substance-related disorders/ or exp alcohol-related disorders/ or exp amphetamine-related disorders/ or exp cocaine-related disorders/ or exp drug overdose/ or exp heroin dependence/ or exp inhalant abuse/ or exp marijuana abuse/ or exp neonatal abstinence syndrome/ or exp opioid-related disorders/ or exp phencyclidine abuse/ or exp psychoses, substance-induced/ or exp substance abuse, intravenous/ or exp substance abuse, oral/ or exp substance withdrawal syndrome/ or exp "tobacco use disorder"/	TS="substance-related disorders"	1217
2	Street Drugs	exp street drugs/ or exp crack cocaine/	TS="street drugs"	492
3	Substance abuser	substance abusing	TS="substance abus*"	34331
4	Substance dependent	Substance dependency	TS="substance dependen*"	3405

5	Substance misuse	Substance misusers, substance misusing	TS="substance misus*"	2721
6	Problematic substance use	problematic substance users, people with problematic substance use, problematic substance using	TS= ("Problematic substance" NEAR/2 (use* or usage or using))	278
7	People who inject drugs	PWID	TS= ("people who inject drug*" or PWID)	1802
8	Injection drug use	injection drug users	TS=("injection drug" NEAR/2 (use* or usage or using))	8264
9	Injection drug use	IDU	TS=IDU	2530
10	People who use drugs	PWUD	TS=("people who use drugs" or PWUD)	345
11	Illicit substance use	Illicit substance using, illicit substance use, illegal substance users, illegal substance using, illegal substance use, street substance users, street substance using, street substance use	TS=((illicit or street or illegal) NEAR/2 (drug or substance) NEAR/2 (use* or usage or using))	9159
12	opioid dependence	opioid dependent, opioid dependency, opioid addiction, opioid abuse, opioid misuse, opioid problem	TS=(opioid* NEAR/2 (addict* or abus* or dependen* or misus* or problem* or disorder*))	9854
13	opiate dependence	opiate dependent, opiate dependency, opiate addiction, opiate abuse, opiate misuse, opiate problem	TS=(opiate* NEAR/2 (addict* or abus* or dependen* or misus* or problem* or disorder*))	4886

14	narcotic dependence	narcotic dependent, narcotic dependency, narcotic addiction, narcotic abuse, narcotic misuse, narcotic problem	TS=(narcotic* NEAR/2 (addict* or abus* or dependen* or misus* or problem* or disorder*))	1727
15	heroin	None	TS=heroin	17783
16	stimulant use disorder		TS="stimulant use disorder"	42
17	exp crack cocaine	MeSH exploded; this is a separate Mesh from cocaine-related disorders and reflects addiction to crack cocaine	TS="crack cocaine"	2124
18	exp cocaine smoking	Mesh exploded; this is a separate Mesh from cocaine-related disorders and crack cocaine	TS="cocaine smoking"	107
19	cocaine dependence	cocaine dependent, cocaine dependency, cocaine addiction, cocaine abuse, cocaine dependence, cocaine misuse, cocaine problem	TS=((cocaine or crack) NEAR/2 (addict* or abus* or dependen* or misus* or problem* or disorder*))	17686
20	amphetamine dependence	amphetamine dependent, dependency, addiction, abuse, dependence, misuse, problem; crystal methamphetamine…; crystal meth….;	TS=((amphetamine or "crystal methamphetamine" or "crystal meth") NEAR/2 (addict* or abus* or dependen* or misus* or problem* or disorder*))	1293

21	alcohol dependence	alcohol dependent, alcohol dependency, alcohol addiction, alcohol abuse, alcohol miuse, alcohol problem	TS=(alcohol NEAR/2 (addict* or abus* or dependen* or misus* or problem* or disorder*))	55793
22	cannabis dependence	cannabis dependent, cannabis dependency, cannabis abuse, cannabis addiction	TS=((cannabis or marijuana) NEAR/2 (addict* or abus* or dependen* or disorder*))	3234
23	tobacco dependence	tobacco or nicotine dependent, tobacco or nicotine dependency, smoking cessation	TS=((tobacco or nicotine or smok*) NEAR/2 (dependen* or disorder* or cessation))	37802
24	Diagnosis, Dual (Psychiatry)		TS="Dual Diagnosis"	2517
25		(All problematic substance use related terms)	1 or 2 or 3 or 4 or 5 or 6 or 7 or 8 or 9 or 10 or 11 or 12 or 13 or 14 or 15 or 16 or 17 or 18 or 19 or 20 or 21 or 22 or 23 or 24	181173
26	Patient centered care	patient centered treatment, patient centered therapy, Client centered care, client centered treatment, client centered therapy, Person-Centered Care, person centered treatment, person centered therapy	TS=((patient or client or person) NEAR/1 cent?red NEAR/1 (care or treatment* or therap*))	5814
27	Patient focused care	Client focused care, person focused care	TS=((patient or client or person) NEAR/1 focus?ed NEAR/1	16

			(care or treatment* or therap*))	
28	Patient participation in treatment planning, process, decisions	Patient/client autonomy in decision making/care/practice/treatment/planning Patient/client involvement in decision making/care/practice/treatment/planning Patient/client control in decision making/care/practice/treatment/planning Patient/client empowerment in decision making/care/practice/treatment/planning	TS=((patient or client) NEAR/1 (autonom* or involve* or control or empower*) NEAR/1 (decision making or care or practic* or treatment* or plan*))	3085
29	Collaborative care	Collaborative practices, collaborative treatment, collaborative care planning	TS=(collaborative NEAR/1 (care or practic* or treatment* or plan*))	7722
30	Shared decision-making	shared decision making, joint decision making	TS=((shared or joint or collaborative) NEAR/2 decision making)	16868
31	Therapeutic alliance		TS=("therapeutic alliance")	3293
32	Relational practice		TS=("relational practic*")	221
33	Enhanced patient-provider relationship	physician/doctor/nurse/professional/provider-patient/client alliance/empowerment/support/enhance	TS=((physician or doctor or nurse or professional or provider) NEAR/1 (patient or client) NEAR/1 (enhanc* or alliance* or empower* or support*))	834

34	Enhanced patient-provider communication	physician/doctor/nurse/professional/provider-patient/client communication	TS=((physician or doctor or nurse or professional or provider) NEAR/1 ("patient communicat*"))	3212
34	Holistic Nursing	holistic nursing care	TS="Holistic nursing"	265
35	Trauma informed care	trauma-informed, trauma informed, trauma centered with care, approach, treatment or therapy	TS=(trauma NEAR/1 (cent?red or informed) NEAR/1 (care or approach or treatment* or therap*))	418
36	Culturally safe care	culturally sensitive care	TS=(cultural* NEAR/1 (safe or sensitive) NEAR/1 (care or approach or treatment* or therap*))	641
37	Family Systems Nursing		TS=("Family Systems Nursing")	52
38	Expert patient program	expert patient programme	TS=("expert patient program*")	26
39		(All patient-centered care related terms)	S26 OR S27 OR S28 OR S29 OR S30 OR S31 OR S32 OR S33 OR S34 OR S35 OR S36 OR S37 OR S38	40471
40			**25 and 39**	**882**
