# Supplementary material for: Conceptualizing patient-centered care for substance use disorder treatment: findings from a systematic scoping review
Source: Subst Abuse Treat Prev Policy. 2019 Sep 11;14:37. doi: 10.1186/s13011-019-0227-0 (PMC6739978; doi:10.1186/s13011-019-0227-0)
Supplement: Supplementary file 3 — Data extraction form. (PDF 287 kb) [file 13011_2019_227_MOESM3_ESM.pdf]

Appendix C

Data Extraction Forms

Table C1. Data extraction and charting for empirical and grey literature sources

**Questions**	**Response options**
**1. General document details that apply to all reference types**	
1.1 Reference Type	Empirical quantitative research, empirical qualitative research, empirical mixed-methods, empirical review, conference paper, report (including case report), guideline, book chapter
1.2 Publication Year	Year of publication
1.3 Country and Location	Country of publication (and location if provided)
1.4 Publication Language	Language of publication<br>Drop down with list of eligible languages
1.5 Research objective, purpose, aims	What was the research objective or specific question (PICO) to be tested?<br>Open text
1.6 Target sample	Would the target population of the study/intervention/health care setting be classified as primarily:<br>a) patients/clients/service users<br>b) health care providers<br>c) both
1.7 Main category of substance used or being treated	a) Tobacco<br>b) Cannabis<br>c) Alcohol<br>d) Opioids (Specify: illicit vs. misuse of prescribed opioids)<br>e) Stimulants (Specify: Cocaine vs. Amphetamine<br>f) Multi/Poly Substance (Specify: e.g., alcohol and opioids)<br>g) Not a targeted group, substance users in general (e.g., people accessing inpatient residential program)<br>h) Dual diagnosis (Specify: e.g., schizophrenia and cannabis)
1.8 Operational definition of patient-centered care	What was the operational definition of the patient-centered care approach or dimension(s) used?<br>Open text:
1.9 Specific Dimension of PCC	Which of the following dimensions/principles/components of PCC were studied?<br>a) Structured PCC framework integrating each of the classic dimensions of PCC.<br>b) Whole person/holistic care/comprehensive care

	c) Individualized/personalized care<br>d) Empowering care/shared decision making/collaborative care<br>e) Enhanced patient-provider relationship/therapeutic alliance/relational care<br>f) Trauma informed care<br>g) Culturally safe care
1.10 Context/setting	What health-oriented context was the PCC intervention apart of?<br>a) Hospital<br>b) Outpatient general health<br>c) Outpatient addition specific<br>d) Residential addiction<br>e) Other, specify:
1.11 Health professionals involved	What health professionals were involved?<br>a) Medical care<br>b) Nursing care<br>c) Case managers<br>d) Social work<br>e) Counselor/psychologist<br>f) Psychiatrist<br>g) Other, specify:
1.12 Type of addiction treatment offered	What type of addiction treatment was being offered?<br><br>Open text:
**2. Data Extraction Questions Specific to Empirical Quantitative and Mixed Methods Studies**	
2.1 Quantitative Study design	Observational, experimental, quasi-experimental?
2.2 Study hypotheses	Open text
2.3 Specific eligibility criteria	What were the study's inclusion and exclusion criteria?
2.4 Sample size	Open text
2.5 Study variables	1. Dependent variable of interest?<br>2. Secondary outcomes?<br>3. Independent variables?
2.6 Secondary outcomes	
2.7 Main results	What were the main results of the study?
2.8 Author's Limitations	What limitations did the authors describe?
2.9 Other limitations	What other limitations are there that the authors did not identify?

**3. Data Extraction Questions Specific to Qualitative Studies**	
3.1 Qualitative design	Was the study design phenomenological, grounded theory, etc.?
3.2 Specific eligibility criteria	What were the study's inclusion and exclusion criteria?
3.3 Sample size	Open text
3.4 Main findings	What were the main findings of the study?
3.5 Author's Limitations	What limitations did the authors describe?
3.6 Other Limitations	What other limitations are there that the authors did not identify?
**4. Data Extraction Questions Specific to Empirical Reviews**	
4.1 Study design	Was the study design: scoping review, systematic review, meta-analysis, report
4.2 Specific eligibility criteria	What were the study selection criteria? (e.g., RCTs only, specific populations, concepts, contexts of interest)
4.3 Interventions of included studies	What intervention(s) was measured, tested, or observed in the included studies?
4.4 Outcomes of included studies	What outcome(s) was measured, tested or observed in the included studies?
4.5 Main effects and conclusions	What were the main effects and conclusions of the review (according to each research question, each intervention or each outcome under review)
4.6 Study's limitations	What limitations did the authors describe?
4.7 Other limitations	What other limitations are they that the authors did not identify?
**5. Data Extraction Questions Specific to Guidelines (including empirical or grey literature or book chapters that are written like a clinical practice guideline)**	
5.1 Target audience	Is there a target audience specified for the guideline/report?<br>a) Policy/decision maker<br>b) Health care providers<br>c) Patient/client/family<br>d) Researcher<br>e) Other, specify:
5.2 Client/patient representation	Were patients/clients included in guideline committee?<br>a) Yes<br>b) No<br>c) Not specified
5.2 Interventions described	List and define all interventions described by the authors of the guideline.

5.3 PCC specific intervention	What patient-centered specific approaches were described?
5.4 Anticipated outcomes of PCC intervention	If applicable, were any outcomes reported (e.g., patient or provider satisfaction)?
5.5 Program evaluation	If available, results of any ongoing program evaluations?
