# Supplementary material for: Conceptualizing patient-centered care for substance use disorder treatment: findings from a systematic scoping review
Source: Subst Abuse Treat Prev Policy. 2019 Sep 11;14:37. doi: 10.1186/s13011-019-0227-0 (PMC6739978; doi:10.1186/s13011-019-0227-0)
Supplement: Supplementary file 4 — Directed Content Analysis Coding Guide. (PDF 140 kb) [file 13011_2019_227_MOESM4_ESM.pdf]

Reference characteristics:

(a) Reference type = quantitative study, qualitative study, mixed methods study, systematic review, report, guideline
(b) Country of publication
(c) Target population = Adult clients with problematic substance use; Youth clients with problematic substance use; Health care providers in substance use treatment settings; Both clients and health care providers
(d) Main substance category = Alcohol; Tobacco; Cannabis; Opioids; Stimulants; Poly-substance; Dual Diagnosis; People receiving addiction treatment in general
(e) Addiction treatment delivered = Pharmacological (includes medication assisted maintenance and detox); Psychosocial (includes education, counseling, etc); Pharmacological & Psychosocial (when a combination of those approaches was used); Not specified (sometimes references did specify the kind of addiction treatment)
(f) Setting = Inpatient; Outpatient; Inpatient & Outpatient

Table 1. Coding Guide for Objective 2

**Category**	**Subcategory**	**Code**	**Definition and Notes**
Therapeutic alliance (TA)	Defining characteristics of TA	Non-judgmental, respectful, dignity, accepting approach, minimized power differences	These characteristics reflect the attitudes or behaviours of the provider
		Anti-stigmatizing approach	More than just non-judgmental or non-discriminating, but an active approach at challenging stigma
		Trust	Trust may also be a condition or outcome of this and may appear in some of the other principles, depending on the context.
		Empathy, understanding, warmth, kindness, supportive approach	

		Bond	Added because it was defined in other frameworks of PCC.
		Time flexible, not being rushed	This could also be some of the factors contributing to developing TA. For now, we'll keep it here but might end up moving depending on the context given in the references to these attributes.
		Openness, active listening, conversational communication style	This could also be some of the factors contributing to developing TA. For now, we'll keep it here but might end up moving depending on the context given in the references to these attributes.<br><br>Need to really see how these aspects are framed/contextualized in the references, these codes might need to be split up and/or moved to factors contributing to.
		Open codes	To be used when the TA was defined in a way that is not synonymous with any of the above codes.
	Consequences or outcomes of TA	Frequency of substance use	e.g., number of days
		Severity or intensity of substance use	e.g., number of times per day, number of drinks per week, a standardized severity score
		Craving, withdrawal	
		Adherence	Adhering to treatment protocol or treatment plan; treatment completion
		Engagement	Initiated treatment, number of counseling

			sessions,
		Retention	Length of stay in treatment
		Patient-reported outcome measures	e.g., Health-related QoL, psychosocial outcomes, functionality,
		Patient-reported experiences	e.g., Treatment satisfaction
		Other dimensions of PCC as a consequence of TA	Open code at first
		Open codes	To be used when for outcomes that does not fall into any of the above codes.
	Factors contributing to increased TA	Open codes	Start with open codes since I don't have any a priori ideas for what this might be. There could be client and/or provider characteristics.
	Quantitative measure of TA	Measured with Working Alliance Inventory	Quant designs
		Measured with Helping Alliance Inventory	Quant designs
		Measured with other tool	Quant designs; if start to notice that there was one common other tool than create a code for it.
	Open Subcategories	Open codes	For anything not captured in the others.

Shared decision-making (SDM)	Defining characteristics of SDM	Encouragement and/or empowerment from provider for patient to make treatment decisions, sharing power	Empowerment is a tricky one because it can also be something that leads to SDM or is an inherent characteristic of SDM.<br>Be very thorough on capturing the context and making sure that is transparent
		Building client's capacity for self-management and self-care, helping client be aware that they're not powerless outside of treatment	This is distinct from empowerment within the actual treatment process itself. That should be captured above.
		Autonomous, independent, client-led decision-making	
		Collaborative decision-making, mutual agreement, mutual decision-making, client participation in, sharing responsibility	
		Sharing information in a manner that is appropriate for the client, allows them to make an informed decision	
		Discussions of client concerns, uncertainties, questions	
		Open codes	For characteristics that don't fit into any of the above codes.
	Consequences or outcomes of SDM	Frequency of substance use	e.g., number of days
		Severity or intensity of substance use	e.g., number of times per day, number of drinks per week, a standardized severity score

		Craving, withdrawal	
		Adherence	Adhering to treatment protocol or treatment plan; treatment completion
		Engagement	Initiated treatment, number of counseling sessions,
		Retention	Length of stay in treatment
		Patient-reported outcome measures	e.g., Health-related QoL, psychosocial outcomes, functionality,
		Patient-reported experiences	e.g., Treatment satisfaction
		Decisional quality; decisional comfort	Feeling informed, feeling that personal values are supported, perceived effectiveness of decision-making process
		Perceived empowerment outside of the treatment (e.g., in relationships, in feelings of increased confidence, accessing other health care)	
		Other dimensions of PCC as a consequence of SDM	Open code at first
		Open codes	For any consequences or outcomes or outcomes not captured above
	Factors contributing to SDM	Open codes	

	Quantitative measures of SDM	Measured by decision aid intervention	For quant references;
		Open codes	For other quant variables that were used for SDM
	Open Subcategories		
Individualized care (IC)	Defining characteristics of IC	Delivery of treatment according to patient needs and preferences from patient perspective	This code is tapping into our conceptualization of pcc-individualized care.
		Treatment matching	Sometimes from the health care provider's pov. Is this really patient-centered? This is for our discussion, worth pointing out/distinguishing.
		Intake assessments that integrate client's expected outcomes and goals for treatment or other efforts to understand client's expectations	
		Intake assessment of what treatment (e.g., what medication, what dose, what type of counseling) client's preferred	
		As-needed-dosing	
		Examine client's potential treatment barriers and providing support to overcome these barriers	

		Intermittent, mid-point, or ongoing evaluation of client outcomes, goals, and preferences	
		Transition or discharge planning at clients pace	
		Open codes	
	Consequences or outcomes of IC	Frequency of substance use	e.g., number of days
		Severity or intensity of substance use	e.g., number of times per day, number of drinks per week, a standardized severity score
		Adherence	Adhering to treatment protocol or treatment plan; treatment completion
		Craving, withdrawal	
		Engagement	Initiated treatment, number of counseling sessions,
		Retention	Length of stay in treatment
		Patient-reported outcome measures	e.g., Health-related QoL, psychosocial outcomes, functionality,
		Patient-reported experiences	e.g., Treatment satisfaction
		Other dimensions of PCC as a consequence of IC	Open code at first
		Open codes	For any qualitative consequences or outcomes or outcomes not captured above

	Factors contributing to IC	Open codes	
	Quantitative measures of IC	Open codes	
	Open Subcategories	Open codes	
Holistic care (HC)	Defining characteristics of HC	Direct provision of addiction treatment within a primary care, psychosocial treatment and/or case management type of care setting that is not addiction specific	Making clear that our definition of psychosocial and/or behavioural interventions includes individual and/or group therapy. Interventions may include any of the following: CBT, MI, MET, psychodynamic, etc)<br><br>Case management - employment support, housing support, legal support, family reconnection
		Direct provision of primary, psychosocial care and/or case management as part of an addiction treatment setting	
		Coordination or referral for primary, psychosocial care and/or case management or a continuum of care approach	
		Involvement of family, community and close others	
		Provision of childcare	

		Delivery of services or treatment that client's perceived spiritual treatment needs	
		Open codes	
	Consequences or outcomes of HC	Frequency of substance use	e.g., number of days
		Severity or intensity of substance use	e.g., number of times per day, number of drinks per week, a standardized severity score
		Adherence	Adhering to treatment protocol or treatment plan; treatment completion
		Engagement	Initiated treatment, number of counseling sessions,
		Retention	Length of stay in treatment
		Health outcomes, improvement in health unrelated to addiction, clinical and/or objective outcomes	Clinical, lab, observed, objective, not self-report
		Patient-reported outcome measures	e.g., Health-related QoL, psychosocial outcomes, functionality,
		Patient-reported experiences	e.g., Treatment satisfaction
		Other dimensions of PCC as a consequence of HC	Open code at first
		Open codes	For any qualitative consequences or outcomes or outcomes not captured above

	Factors contributing to HC	Open codes	
	Quantitative measures of HC	Open codes	
	Open Subcategories		
Trauma informed care (TIC)	Defining characteristics of TIC	TIC framework applied with a citation	
		Open Codes	Nuanced or unique way of expanding on their approach, etc.
	Consequences or outcomes of HC	Frequency of substance use	e.g., number of days
		Severity or intensity of substance use	e.g., number of times per day, number of drinks per week, a standardized severity score
		Adherence	Adhering to treatment protocol or treatment plan; treatment completion
		Engagement	Initiated treatment, number of counseling sessions,
		Retention	Length of stay in treatment
		Patient-reported outcome measures	e.g., Health-related QoL, psychosocial outcomes, functionality,
		Patient-reported experiences	e.g., Treatment satisfaction

		Other dimensions of PCC as a consequence of HC	Open code at first
	Factors contributing with TIC	Open codes	Might be systems/organizational/structural factors (e.g., provider training, redesigning structures)
	Quantitative measures of TIC	Open codes	
		Open sub-categories	
Culturally safe care (CSC)	Defining characteristics of CSC	Framework applied with a citation (including training, compliance monitoring)	
		Matching culture/ethnicity between provider and client	
		Open codes	For those references that include a nuanced or unique way of expanding on their approach
	Consequences or outcomes of CSC	Frequency of substance use	e.g., number of days
		Severity or intensity of substance use	e.g., number of times per day, number of drinks per week, a standardized severity score
		Adherence	Adhering to treatment protocol or treatment plan; treatment completion
		Engagement	Initiated treatment, number of counseling sessions,

		Retention	Length of stay in treatment, may need to add a qualifier for in this principle.
		Patient-reported outcome measures	e.g., Health-related QoL, psychosocial outcomes, functionality,
		Patient-reported experiences	e.g., Treatment satisfaction
		Other dimensions of PCC as a consequence of CSC	Open code at first
		Open codes	For any qualitative consequences or outcomes or outcomes not captured above;<br><br>For discussion of possible outcomes associated with CSC point out that this is usually relevant to the particular sub-group of interest.
	Factors contributing to CSC	Open codes	Might be systems/organizational/structural factors (e.g., provider training, redesigning structures)
	Quantitative measures of CSC	Open codes	
