# Supplementary material for: Conceptualizing patient-centered care for substance use disorder treatment: findings from a systematic scoping review
Source: Subst Abuse Treat Prev Policy. 2019 Sep 11;14:37. doi: 10.1186/s13011-019-0227-0 (PMC6739978; doi:10.1186/s13011-019-0227-0)
Supplement: Supplementary file 5 — Excluded references. (PDF 2429 kb) [file 13011_2019_227_MOESM5_ESM.pdf]

Result #	Reference	Exclusion Criteria
1	**Marianne S Matthias, Melvin T Donaldson, Agnes C Jensen, Erin E Krebs**. "I Was a Little Surprised": Qualitative Insights From Patients Enrolled in a 12-Month Trial Comparing Opioids With Nonopioid Medications for Chronic Musculoskeletal Pain.. *The journal of pain : official journal of the American Pain Society*. 2018//. #volume#:#pages#	Level 1, Form Title/abstract screen
2	**Yoong Mei Theng, Suzaily Wahab, Noor Alaudin Wahab, Hatta Sidi, Srijit Das**. Schizophrenia And Nicotine Dependence: What Psychopharmacological Treatment Options Are Available For The Duo Perturbationes?.. *Current drug targets*. 2017//. #volume#:#pages#	Level 1, Form Title/abstract screen
3	**Katie Schultz, Ciwang Teyra, Glenda Breiler, Tessa Evans-Campbell, Cynthia Pearson**. "They Gave Me Life": Motherhood and Recovery in a Tribal Community.. *Substance use & misuse*. 2018//. #volume#:1	Level 1, Form Title/abstract screen
4	**John B Correa, Karen O Brandon, Lauren R Meltzer, Hannah J Hoehn, Barbara Pineiro, Thomas H Brandon, Vani N Simmons**. Electronic cigarette use among patients with cancer: Reasons for use, beliefs, and patient-provider communication.. *Psycho-oncology*. 2018//. #volume#:#pages#	Level 1, Form Title/abstract screen
5	**Doyanne Darnell, Aaron Flaster, Karin Hendricks, Amanda Kerbrat, Katherine Anne Comtois**. Adolescent clinical populations and associations between trauma and behavioral and emotional problems.. *Psychological trauma : theory, research, practice and policy*. 2018//. #volume#:#pages#	Level 1, Form Title/abstract screen
6	**Julia Rozanova, Olga Morozova, Lyuba Azbel, Chethan Bachireddy, Jacob M Izenberg, Tetiana Kiriazova, Sergiy Dvoryak, Frederick L Altice**. Perceptions of Health-Related Community Reentry Challenges among Incarcerated Drug Users in Azerbaijan, Kyrgyzstan, and Ukraine.. *Journal of urban health : bulletin of the New York Academy of Medicine*. 2018//. #volume#:#pages#	Level 1, Form Title/abstract screen
8	**Nikki R Wooten, Jordan A Brittingham, Ronald O Pitner, Abbas S Tavakoli, Diana D Jeffery, K Sue Haddock**. Purchased Behavioral Health Care Received by Military Health System Beneficiaries in Civilian Medical Facilities, 2000-2014.. *Military medicine*. 2018//. #volume#:#pages#	Level 1, Form Title/abstract screen
9	**Elise Omaki, Renan Castillo, Karen Eden, Steve Davis, Eileen McDonald, Umbreen Murtaza, Andrea Gielen,My Healthy Choices Decision Aid Study Team**. Using m-health tools to reduce the misuse of opioid pain relievers.. *Injury prevention : journal of the International Society for Child and Adolescent Injury Prevention*. 2017//. #volume#:#pages#	Level 1, Form Title/abstract screen
10	**Donald N. Givler, Amy Givler**. Health Screening.. *#journal#*. 2017//. #volume#:#pages#	Level 1, Form Title/abstract screen
11	**Susie Rudge, Janet Denise Feigenbaum, Peter Fonagy**. Mechanisms of change in dialectical behaviour therapy and cognitive behaviour therapy for borderline personality disorder: a critical review of the literature.. *Journal of mental health (Abingdon, England)*. 2017//. #volume#:1	Level 1, Form Title/abstract screen
12	**Jane Murray Cramm, Anna Petra Nieboer**. Is "disease management" the answer to our problems? No! Population health management and (disease) prevention require "management of overall well-being".. *BMC health services research*. 2016//. 16:500	Level 1, Form Title/abstract screen
13	**Kelley A Saia, Davida Schiff, Elisha M Wachman, Pooja Mehta, Annmarie Vilkins, Michelle Sia, Jordana Price, Tirah Samura, Justin DeAngelis,**	Level 2, Form Full Text

	**Clark V Jackson, Sawyer F Emmer, Daniel Shaw, Sarah Bagley**. Caring for Pregnant Women with Opioid Use Disorder in the USA: Expanding and Improving Treatment.. *Current obstetrics and gynecology reports*. 2016//. 5:257	Screening
14	**Preeta Saxena, Christine E Grella, Nena P Messina**. Continuing Care and Trauma in Women Offenders' Substance Use, Psychiatric Status, and Self-Efficacy Outcomes.. *Women & criminal justice*. 2016//. 26:99	Level 1, Form Title/abstract screen
15	**Sofie L Champassak, Delwyn Catley, Sarah Finocchario-Kessler, Maghen Farris, Maniza Ehtesham, Rachel Schoor, Kathy Goggin**. Physician smoking cessation counseling and adherence to a clinical practice guideline.. *European journal for person centered healthcare*. 2014//. 2:477	Level 2, Form Full Text Screening
16	**Paul Crits-Christoph, Robert Gallop, Mary Beth Connolly Gibbons, Jaclyn S Sadicario, George Woody**. Measuring Outcome in the Treatment of Cocaine Dependence.. *Journal of alcoholism and drug dependence*. 2013//. 1:#pages#	Level 1, Form Title/abstract screen
17	**Camille S Wilson, Melanie E Bennett, Alan S Bellack**. Impact of Family History in Persons With Dual Diagnosis.. *Journal of dual diagnosis*. 2013//. 9:30	Level 1, Form Title/abstract screen
18	**Swedish Council on Health Technology Assessment**. . *#journal#*. 2012//. #volume#:#pages#	Level 1, Form Title/abstract screen
20	**R Fredericksen, P K Crane, J Tufano, J Ralston, S Schmidt, T Brown, D Layman, R D Harrington, S Dhanireddy, T Stone, W Lober, M M Kitahata, H M Crane**. Integrating a web-based, patient-administered assessment into primary care for HIV-infected adults.. *Journal of AIDS and HIV research (Online)*. 2012//. 4:47	Level 1, Form Title/abstract screen
21	**Shelly A Wiechelt, Brenda A Miller, Nancy J Smyth, Eugene Maguin**. ASSOCIATIONS BETWEEN POST-TRAUMATIC STRESS DISORDER SYMPTOMS AND ALCOHOL AND OTHER DRUG PROBLEMS: IMPLICATIONS FOR SOCIAL WORK PRACTICE.. *Practice (Birmingham, England)*. 2011//. 23:183	Level 1, Form Title/abstract screen
22	**Colleen Dell, Jennifer Kilty, Cathy Fillmore, Sheila Grantham, Tara Lyons, Sharon Clarke, Carol Hopkins**. Turtle Finding Fact Sheet: The Role of the Treatment Provider in Aboriginal Women's Healing from Illicit Drug Abuse.. *CES4Health.info*. 2010//. #volume#:#pages#	Level 2, Form Full Text Screening
24	**Aaron Hogue, Howard A Liddle**. Family-based treatment for adolescent substance abuse: controlled trials and new horizons in services research.. *Journal of family therapy*. 2009//. 31:126	Level 2, Form Full Text Screening
25	**Anthony C Tommasello**. Substance abuse and pharmacy practice: what the community pharmacist needs to know about drug abuse and dependence.. *Harm reduction journal*. 2004//. 1:3	Level 1, Form Title/abstract screen
26	**James L. Elmore**. Dissociative Spectrum Disorders in the Primary Care Setting.. *Primary care companion to the Journal of clinical psychiatry*. 2000//. 2:37	Level 1, Form Title/abstract screen
27	**Wiley D Jenkins, Alicia K Matthews, Angie Bailey, Whitney E Zahnd, Karriem S Watson, Georgia Mueller-Luckey, Yamile Molina, David Crumly, Julie Patera**. Rural areas are disproportionately impacted by smoking and lung cancer.. *Preventive medicine reports*. 2018//. 10:200	Level 1, Form Title/abstract screen
28	**Kamilla L Venner, Victoria Sanchez, Jacqueline Garcia, Robert L Williams, Andrew L Sussman**. Moving Away from the Tip of the Pyramid: Screening and Brief Intervention for Risky Alcohol and Opioid Use in	Level 1, Form Title/abstract screen

	Underserved Patients.. *Journal of the American Board of Family Medicine : JABFM*. 2018//. 31:243	
29	**Christine A Pace, Lisa A Uebelacker**. Addressing Unhealthy Substance Use in Primary Care.. *The Medical clinics of North America*. 2018//. 102:567	Level 1, Form Title/abstract screen
30	**M K A Hyder, J P Tripathy, J Kaur, P P Mandal, R Sharma, A M V Kumar, T Thamarangsi, R J Singh**. Tuberculosis-tobacco integration in the South-East Asia Region: policy analysis and implementation framework.. *The international journal of tuberculosis and lung disease : the official journal of the International Union against Tuberculosis and Lung Disease*. 2018//. 22:807	Level 1, Form Title/abstract screen
32	**Kathleen C Thomas, Hillary Owino, Sana Ansari, Leslie Adams, Julianne M Cyr, Bradley N Gaynes, Seth W Glickman**. Patient-Centered Values and Experiences with Emergency Department and Mental Health Crisis Care.. *Administration and policy in mental health*. 2018//. 45:611	Level 1, Form Title/abstract screen
33	**T V Agibalova, P V Tuchin, D I Shustov, O Zh Buzik**. [The therapeutic alliance as a main factor of building cooperation during the treatment of patients with opiate addiction].. *Zhurnal nevrologii i psikhiatrii imeni S.S. Korsakova*. 2014//. 114:57	Level 2, Form Full Text Screening
34	**Allison J Ober, Katherine E Watkins, Colleen M McCullough, Claude M Setodji, Karen Osilla, Sarah B Hunter**. Patient predictors of substance use disorder treatment initiation in primary care.. *Journal of substance abuse treatment*. 2018//. 90:64	Level 2, Form Full Text Screening
35	**Katherine E Watkins, Allison J Ober, Karen Lamp, Mimi Lind, Allison Diamant, Karen Chan Osilla, Keith Heinzerling, Sarah B Hunter, Harold Alan Pincus**. Implementing the Chronic Care Model for Opioid and Alcohol Use Disorders in Primary Care.. *Progress in community health partnerships : research, education, and action*. 2017//. 11:397	Level 1, Form Title/abstract screen
36	**Josephus Fm van den Heuvel, T Katrien Groenhof, Jan Hw Veerbeek, Wouter W van Solinge, A Titia Lely, Arie Franx, Mireille N Bekker**. eHealth as the Next-Generation Perinatal Care: An Overview of the Literature.. *Journal of medical Internet research*. 2018//. 20:e202	Level 1, Form Title/abstract screen
37	**Katherine Berry, Tom Palmer, Lynsey Gregg, Christine Barrowclough, Fiona Lobban**. Attachment and therapeutic alliance in psychological therapy for people with recent onset psychosis who use cannabis.. *Clinical psychology & psychotherapy*. 2018//. 25:440	Level 1, Form Title/abstract screen
38	**P Kushalnagar, Alina Engelman, G Sadler**. Deaf patient-provider communication and lung cancer screening: Health Information National Trends survey in American Sign Language (HINTS-ASL).. *Patient education and counseling*. 2018//. 101:1232	Level 1, Form Title/abstract screen
39	**Katelyn A Grayson-Sneed, Robert C Smith**. A research coding method to evaluate a smoking cessation model for training residents-A preliminary report.. *Patient education and counseling*. 2018//. 101:541	Level 1, Form Title/abstract screen
40	**Anke Friedrichs, Anna Silkens, Jens Reimer, Ludwig Kraus, Norbert Scherbaum, Daniela Piontek, Jeanette Rohrig, Jochen Hempleman, Martin Harter, Angela Buchholz**. Role preferences of patients with alcohol use disorders.. *Addictive behaviors*. 2018//. 84:248	Level 2, Form Full Text Screening
42	**Christian Schrader, Antoinette Lenton, Peter Gertonson, Alexander Rahimi**. Redeveloping Substance Abuse Treatment for Military Personnel.. *Current psychiatry reports*. 2018//. 20:45	Level 2, Form Full Text Screening

43	**Stephen R Holt, Joseph H Donroe, Dana A Cavallo, Jeanette M Tetrault**. Addressing discordant quantitative urine buprenorphine and norbuprenorphine levels: Case examples in opioid use disorder.. *Drug and alcohol dependence*. 2018//. 186:171	Level 2, Form Full Text Screening
44	**Brian E Sandoval, Jennifer Bell, Parinda Khatri, Patricia J Robinson**. Toward a Unified Integration Approach: Uniting Diverse Primary Care Strategies Under the Primary Care Behavioral Health (PCBH) Model.. *Journal of clinical psychology in medical settings*. 2018//. 25:187	Level 2, Form Full Text Screening
45	**Caroline Shulman, Briony F Hudson, Joseph Low, Nigel Hewett, Julian Daley, Peter Kennedy, Sarah Davis, Niamh Brophy, Diana Howard, Bella Vivat, Patrick Stone**. End-of-life care for homeless people: A qualitative analysis exploring the challenges to access and provision of palliative care.. *Palliative medicine*. 2018//. 32:36	Level 1, Form Title/abstract screen
46	**Gregory A Goldman, Robert J Gregory**. Preliminary relationships between adherence and outcome in dynamic deconstructive psychotherapy.. *Psychotherapy (Chicago, Ill.)*. 2009//. 46:480	Level 2, Form Full Text Screening
47	**Elizabeth E Epstein, Barbara S McCrady, Kevin A Hallgren, Ayorkor Gaba, Sharon Cook, Noelle Jensen, Thomas Hildebrandt, Cathryn Glanton Holzhauer, Mark D Litt**. Individual versus group female-specific cognitive behavior therapy for alcohol use disorder.. *Journal of substance abuse treatment*. 2018//. 88:27	Level 2, Form Full Text Screening
48	**Xinli Li, William Nylander, Tracy Smith, Soonhee Han, William Gunnar**. Risk Factors and Predictive Model Development of Thirty-Day Post-Operative Surgical Site Infection in the Veterans Administration Surgical Population.. *Surgical infections*. 2018//. 19:278	Level 1, Form Title/abstract screen
49	**Paula Wyndow, Roz Walker, Tracy Reibel**. A Novel Approach to Transforming Smoking Cessation Practice for Pregnant Aboriginal Women and Girls Living in the Pilbara.. *Healthcare (Basel, Switzerland)*. 2018//. 6:#pages#	Level 2, Form Full Text Screening
50	**Paul R King, Gregory P Beehler, Bonnie M Vest, Kerry Donnelly, Laura O Wray**. Qualitative exploration of traumatic brain injury-related beliefs among U.S. military veterans.. *Rehabilitation psychology*. 2018//. 63:121	Level 1, Form Title/abstract screen
51	**Abhijit Nadkarni**. Increasing access to psychosocial interventions for alcohol use disorders: Home based interventions.. *Indian journal of psychiatry*. 2018//. 60:S564	Level 1, Form Title/abstract screen
52	**Ju Long, Juntao Michael Yuan, Ron Kim Johnson**. A Shared Decision-Making Tool to Prevent Substance Abuse: Protocol for a Randomized Controlled Trial.. *JMIR research protocols*. 2018//. 7:e5	Level 2, Form Full Text Screening
53	**Georgia J Michlig, Ryan P Westergaard, Yukyan Lam, Azal Ahmadi, Gregory D Kirk, Andrew Genz, Jeanne Keruly, Heidi Hutton, Pamela J Surkan**. Avoidance, meaning and grief: psychosocial factors influencing engagement in HIV care.. *AIDS care*. 2018//. 30:511	Level 1, Form Title/abstract screen
54	**Kevin Selby, Gillian Bartlett-Esquilant, Jacques Cornuz**. Personalized cancer screening: helping primary care rise to the challenge.. *Public health reviews*. 2018//. 39:4	Level 1, Form Title/abstract screen
55	**Nadia Minian, Aliya Noormohamed, Laurie Zawertailo, Dolly Baliunas, Norman Giesbrecht, Bernard Le Foll, Jurgen Rehm, Andriy Samokhvalov, Peter L Selby**. A method for co-creation of an evidence-based patient workbook to address alcohol use when quitting smoking in primary care: a case study.. *Research involvement and engagement*. 2018//. 4:4	Level 1, Form Title/abstract screen

56	**Leontine van der Meer, Anna Petra Nieboer, Harry Finkenflugel, Jane Murray Cramm**. The importance of person-centred care and co-creation of care for the well-being and job satisfaction of professionals working with people with intellectual disabilities.. *Scandinavian journal of caring sciences*. 2018//. 32:76	Level 1, Form Title/abstract screen
57	**Leigh Chapman**. Lessons Learned After Losing my Brother to an Overdose: A Call to Action for Nurse Leaders.. *Nursing leadership (Toronto, Ont.)*. 2017//. 30:73	Level 2, Form Full Text Screening
58	**Craig Warlick, Kimber P Richter, Delwyn Catley, Byron J Gajewski, Laura E Martin, Laura M Mussulman**. Two brief valid measures of therapeutic alliance in counseling for tobacco dependence.. *Journal of substance abuse treatment*. 2018//. 86:60	Level 2, Form Full Text Screening
59	**Brett C Bade, Paul B Brasher, Branden W Luna, Gerard A Silvestri, Nichole T Tanner**. Reviewing Lung Cancer Screening: The Who, Where, When, Why, and How.. *Clinics in chest medicine*. 2018//. 39:31	Level 1, Form Title/abstract screen
60	**Lisa D Butler, Eugene Maguin, Janice Carello**. Retraumatization Mediates the Effect of Adverse Childhood Experiences on Clinical Training-Related Secondary Traumatic Stress Symptoms.. *Journal of trauma & dissociation : the official journal of the International Society for the Study of Dissociation (ISSD)*. 2018//. 19:25	Level 1, Form Title/abstract screen
61	**Stefano Omboni, Marina Caserini**. Effectiveness of pharmacist's intervention in the management of cardiovascular diseases.. *Open heart*. 2018//. 5:e000687	Level 1, Form Title/abstract screen
62	**Roger Lee Mendoza**. Is medical treatment of Alcohol Withdrawal Syndrome a Stag Hunt? Challenges and opportunities in managing risk and uncertainty in addiction cessation.. *Risk management and healthcare policy*. 2018//. 11:1	Level 1, Form Title/abstract screen
63	**Allison J Ober, Katherine E Watkins, Sarah B Hunter, Brett Ewing, Karen Lamp, Mimi Lind, Kirsten Becker, Keith Heinzerling, Karen C Osilla, Allison L Diamant, Claude M Setodji**. Assessing and improving organizational readiness to implement substance use disorder treatment in primary care: findings from the SUMMIT study.. *BMC family practice*. 2017//. 18:107	Level 1, Form Title/abstract screen
64	**S Ryan Greysen, James D Harrison, Sunil Kripalani, Eduard Vasilevskis, Edmondo Robinson, Joshua Metlay, Jeffery L Schnipper, David Meltzer, Neil Sehgal, Gregory W Ruhnke, Mark V Williams, Andrew D Auerbach**. Understanding patient-centred readmission factors: a multi-site, mixed-methods study.. *BMJ quality & safety*. 2017//. 26:33	Level 1, Form Title/abstract screen
65	**Fernea Moyo, Ella Archibald, Jason T Slyer**. Effectiveness of decision aids on smoking cessation in adult patients: a systematic review protocol.. *JBI database of systematic reviews and implementation reports*. 2017//. 15:2881	Level 2, Form Full Text Screening
66	**Thomas R Hickey, Paul D Kirwin, Elizabeth C Gardner, Jessica Feinleib**. Patient-Centered Perioperative Care for a Victim of Military Sexual Trauma.. *Military medicine*. 2017//. 182:e1807	Level 1, Form Title/abstract screen
67	**P Thomas, J M Thomas**. [Specificity of communication in general practice].. *Revue medicale de Bruxelles*. 2017//. 38:377	Level 1, Form Title/abstract screen
68	**Daniel R Witt, Gregory M Garrison, Cesar A Gonzalez, Terrence J Witt, Kurt B Angstman**. Six-Month Outcomes for Collaborative Care Management of Depression Among Smoking and Nonsmoking Patients.. *Health services research and managerial epidemiology*. 2017//. 4:2333392817721648	Level 1, Form Title/abstract screen

69	**Clara Iversen**. Now or never: smoking cessation discussions in the face of serious illness.. *Sociology of health & illness*. 2017//. 39:1330	Level 1, Form Title/abstract screen
70	**Dawn I Velligan, Martha Sajatovic, Ainslie Hatch, Pavel Kramata, John P Docherty**. Why do psychiatric patients stop antipsychotic medication? A systematic review of reasons for nonadherence to medication in patients with serious mental illness.. *Patient preference and adherence*. 2017//. 11:449	Level 1, Form Title/abstract screen
71	**Pablo Barrio, Lidia Teixidor, Lluisa Ortega, Merce Balcells, Eduard Vieta, Antoni Gual**. Patients' Knowledge and Attitudes Towards Regular Alcohol Urine Screening: A Survey Study.. *Journal of addiction medicine*. 2017//. 11:300	Level 1, Form Title/abstract screen
72	**Kirsten Marchand, Eugenia Oviedo-Joekes**. Prioritizing the patient in patient-centered addictions treatment.. *Addiction (Abingdon, England)*. 2017//. 112:466	Level 1, Form Title/abstract screen
73	**Torsten Kolind, Morten Hesse**. Patient-centred care-perhaps the future of substance abuse treatment.. *Addiction (Abingdon, England)*. 2017//. 112:465	Level 1, Form Title/abstract screen
74	**Emma Broglia, Abigail Millings, Michael Barkham**. Comparing counselling alone versus counselling supplemented with guided use of a well-being app for university students experiencing anxiety or depression (CASELOAD): protocol for a feasibility trial.. *Pilot and feasibility studies*. 2017//. 3:3	Level 1, Form Title/abstract screen
75	**Carol J Strike, Adrian Guta**. Patient-centred care and patient engagement to inform the use of psychosocial interventions with opioid substitution treatment: another path for Day & Mitcheson to follow.. *Addiction (Abingdon, England)*. 2017//. 112:1338	Level 1, Form Title/abstract screen
76	**Carolyn Y Fang, Carolyn J Heckman**. Informational and Support Needs of Patients with Head and Neck Cancer: Current Status and Emerging Issues.. *Cancers of the head & neck*. 2016//. 1:#pages#	Level 1, Form Title/abstract screen
77	**Sarah Masefield, Pippa Powell, Carlos Jimenez-Ruiz, Peter Hajek, Keir Lewis, Stefan Andreas, Philip Tonnesen, Onno van Schayck, Christina Gratziou, Bertrand Dautzenberg, Serena Tonstad, Thomas Hering, Stephano Nardini, Monica Fletcher**. Recommendations to improve smoking cessation outcomes from people with lung conditions who smoke.. *ERJ open research*. 2016//. 2:#pages#	Level 1, Form Title/abstract screen
79	**Udi E Ghitza**. Overlapping Mechanisms of Stress-Induced Relapse to Opioid Use Disorder and Chronic Pain: Clinical Implications.. *Frontiers in psychiatry*. 2016//. 7:80	Level 1, Form Title/abstract screen
80	**Michael B Steinberg, Daniel P Giovenco, Cristine D Delnevo**. Patient-physician communication regarding electronic cigarettes.. *Preventive medicine reports*. 2015//. 2:96	Level 1, Form Title/abstract screen
81	**Michael B Seidman, Robert D Vining, Stacie A Salsbury**. Collaborative care for a patient with complex low back pain and long-term tobacco use: a case report.. *The Journal of the Canadian Chiropractic Association*. 2015//. 59:216	Level 1, Form Title/abstract screen
82	**Peter M Haddad, Cecilia Brain, Jan Scott**. Nonadherence with antipsychotic medication in schizophrenia: challenges and management strategies.. *Patient related outcome measures*. 2014//. 5:43	Level 1, Form Title/abstract screen
83	**Masoomeh Maarefvand, Hamid Reza Ghiasvand, Hamed Ekhtiari**. Drug Craving Terminology among Opiate Dependents; A Mixed Method Study.. *Iranian journal of psychiatry*. 2013//. 8:97	Level 1, Form Title/abstract screen

85	**Niki A Miller, Lisa M Najavits**. Creating trauma-informed correctional care: a balance of goals and environment.. *European journal of psychotraumatology*. 2012//. 3:#pages#	Level 1, Form Title/abstract screen
90	**Philippe Nuss, Martina Hummer, Cedric Tessier**. The use of amisulpride in the treatment of acute psychosis.. *Therapeutics and clinical risk management*. 2007//. 3:3	Level 1, Form Title/abstract screen
91	**Avram H Mack, Richard J Frances**. Treatment of alcohol use disorders in adolescents.. *Journal of psychiatric practice*. 2003//. 9:195	Level 1, Form Title/abstract screen
92	**K T Mueser, D L Noordsy, R E Drake, L Fox**. [Integrated treatment for severe mental illness and substance abuse: Effective components of programs for persons with co-occurring disorders.].. *Sante mentale au Quebec*. 2001//. 26:22	Level 2, Form Full Text Screening
93	**D Naber, S Kasper**. The importance of treatment acceptability to patients.. *International journal of psychiatry in clinical practice*. 2000//. 4:25	Level 1, Form Title/abstract screen
94	**A Juntunen, M Hwalek, A V Neale**. Tracking and interviewing clients at risk for HIV and substance abuse in a Latino community.. *Evaluation and program planning*. 1999//. 22:305	Level 1, Form Title/abstract screen
95	**L Luborsky, J P Barber, L Siqueland, S Johnson, L M Najavits, A Frank, D Daley**. The Revised Helping Alliance Questionnaire (HAq-II) : Psychometric Properties.. *The Journal of psychotherapy practice and research*. 1996//. 5:260	Level 1, Form Title/abstract screen
96	**Davida M Schiff, Barry Zuckerman, Elizabeth Hutton, Carolyn Genatossio, Catherine Michelson, Megan Bair-Merritt**. Development and Pilot Implementation of a Trauma-Informed Care Curriculum for Pediatric Residents.. *Academic pediatrics*. 2017//. 17:794	Level 1, Form Title/abstract screen
97	**Laura E Miller, Scott A Eldredge, Elizabeth D Dalton**. "Pain Is What the Patient Says It Is": Nurse-Patient Communication, Information Seeking, and Pain Management.. *The American journal of hospice & palliative care*. 2017//. 34:966	Level 1, Form Title/abstract screen
98	**Lauren K Whiteside, Doyanne Darnell, Karlee Jackson, Jin Wang, Joan Russo, Dennis M Donovan, Douglas F Zatzick**. Collaborative care from the emergency department for injured patients with prescription drug misuse: An open feasibility study.. *Journal of substance abuse treatment*. 2017//. 82:12	Level 1, Form Title/abstract screen
99	**Traci H Abraham, Patricia Wright, Penny White, Brenda M Booth, Michael A Cucciare**. Feasibility and acceptability of shared decision-making to promote alcohol behavior change among women Veterans: Results from focus groups.. *Journal of addictive diseases*. 2017//. 36:252	Level 2, Form Full Text Screening
100	**Samuel F Sestito, Keri L Rodriguez, Shaddy K Saba, James W Conley, Michael A Mitchell, Adam J Gordon**. Homeless veterans' experiences with substance use, recovery, and treatment through photo elicitation.. *Substance abuse*. 2017//. 38:422	Level 1, Form Title/abstract screen
101	**Davida M Schiff, Barry Zuckerman, Elisha M Wachman, Megan Bair-Merritt**. Trainees' knowledge, attitudes, and practices towards caring for the substance-exposed mother-infant dyad.. *Substance abuse*. 2017//. 38:414	Level 1, Form Title/abstract screen
102	**Gladstone C 2nd McDowell, Joseph Winchell**. Role of primary care physicians in intrathecal pain management: a narrative review of the literature.. *Postgraduate medicine*. 2018//. 130:411	Level 1, Form Title/abstract screen
103	**Myriam Forster, Timothy J Grigsby, Christopher J Rogers, Stephanie M**	Level 1, Form

	**Benjamin**. The relationship between family-based adverse childhood experiences and substance use behaviors among a diverse sample of college students.. *Addictive behaviors*. 2018//. 76:298	Title/abstract screen
104	**Marianne S Matthias, Nicole L Johnson, Cleveland G Shields, Matthew J Bair, Palmer MacKie, Monica Huffman, Stewart C Alexander**. "I'm Not Gonna Pull the Rug out From Under You": Patient-Provider Communication About Opioid Tapering.. *The journal of pain : official journal of the American Pain Society*. 2017//. 18:1365	Level 1, Form Title/abstract screen
105	**Maxine Stitzer, Tim Matheson, Colin Cunningham, James L Sorensen, Daniel J Feaster, Lauren Gooden, Alexis S Hammond, Heather Fitzsimons, Lisa R Metsch**. Enhancing patient navigation to improve intervention session attendance and viral load suppression of persons with HIV and substance use: a secondary post hoc analysis of the Project HOPE study.. *Addiction science & clinical practice*. 2017//. 12:16	Level 1, Form Title/abstract screen
106	**Katharine A Bradley, Evette Joy Ludman, Laura J Chavez, Jennifer F Bobb, Susan J Ruedebusch, Carol E Achtmeyer, Joseph O Merrill, Andrew J Saxon, Ryan M Caldeiro, Diane M Greenberg, Amy K Lee, Julie E Richards, Rachel M Thomas, Theresa E Matson, Emily C Williams, Eric Hawkins, Gwen Lapham, Daniel R Kivlahan**. Patient-centered primary care for adults at high risk for AUDs: the Choosing Healthier Drinking Options In primary CarE (CHOICE) trial.. *Addiction science & clinical practice*. 2017//. 12:15	Level 2, Form Full Text Screening
107	**Ryan P Westergaard, Andrew Genz, Kristen Panico, Pamela J Surkan, Jeanne Keruly, Heidi E Hutton, Larry W Chang, Gregory D Kirk**. Acceptability of a mobile health intervention to enhance HIV care coordination for patients with substance use disorders.. *Addiction science & clinical practice*. 2017//. 12:11	Level 1, Form Title/abstract screen
108	**Brittni N Howard, Richard Van Dorn, Bronwyn J Myers, William A Zule, Felicia A Browne, Tara Carney, Wendee M Wechsberg**. Barriers and facilitators to implementing an evidence-based woman-focused intervention in South African health services.. *BMC health services research*. 2017//. 17:746	Level 1, Form Title/abstract screen
109	**Jan Gryczynski, Courtney D Nordeck, Shannon Gwin Mitchell, Kathleen R Page, Luke L Johnsen, Kevin E O'Grady, Robert P Schwartz**. Pilot Studies Examining Feasibility of Substance Use Disorder Screening and Treatment Linkage at Urban Sexually Transmitted Disease Clinics.. *Journal of addiction medicine*. 2017//. 11:350	Level 1, Form Title/abstract screen
110	**Jeffry Shaefer, Antje M Barreveld, Paul Arnstein, Ronald J Kulich**. Interprofessional Education for the Dentist in Managing Acute and Chronic Pain.. *Dental clinics of North America*. 2016//. 60:825	Level 1, Form Title/abstract screen
111	**Leena Mittal, Joji Suzuki**. Feasibility of collaborative care treatment of opioid use disorders with buprenorphine during pregnancy.. *Substance abuse*. 2017//. 38:261	Level 1, Form Title/abstract screen
112	**Ellen L Bassuk, Rachel E Latta, Robert Sember, Sheela Raja, Molly Richard**. Universal Design for Underserved Populations: Person-Centered, Recovery-Oriented and Trauma Informed.. *Journal of health care for the poor and underserved*. 2017//. 28:896	Level 1, Form Title/abstract screen
113	**Evan Senreich**. The Perceptions of White Clients in a Substance Abuse Program in Which They Are in the Minority.. *Substance use & misuse*. 2017//. 52:34	Level 1, Form Title/abstract screen
114	**Michael J Silverman**. Effects of Live and Educational Music Therapy on	Level 2, Form

	Working Alliance and Trust With Patients on Detoxification Unit: A Four-Group Cluster-Randomized Trial.. *Substance use & misuse*. 2016//. 51:1741	Full Text Screening
115	**Frances J Kay-Lambkin, Amanda L Baker, Kerrin Palazzi, Terry J Lewin, Brian J Kelly**. Therapeutic Alliance, Client Need for Approval, and Perfectionism as Differential Moderators of Response to eHealth and Traditionally Delivered Treatments for Comorbid Depression and Substance Use Problems.. *International journal of behavioral medicine*. 2017//. 24:728	Level 2, Form Full Text Screening
116	**Pablo Barrio, Lluisa Ortega, Hugo Lopez, Antoni Gual**. Self-management and Shared Decision-Making in Alcohol Dependence via a Mobile App: a Pilot Study.. *International journal of behavioral medicine*. 2017//. 24:722	Level 1, Form Title/abstract screen
117	**David E Gerber, Heidi A Hamann, Noel O Santini, Suhny Abbara, Hsienchang Chiu, Molly McGuire, Lisa Quirk, Hong Zhu, Simon J Craddock Lee**. Patient navigation for lung cancer screening in an urban safety-net system: Protocol for a pragmatic randomized clinical trial.. *Contemporary clinical trials*. 2017//. 60:78	Level 1, Form Title/abstract screen
118	**A C Sweetland, A Kritski, M A Oquendo, M E Sublette, A Norcini Pala, L R Batista Silva, A Karpati, E C Silva, M O Moraes, J R Lapa E Silva, M L Wainberg**. Addressing the tuberculosis-depression syndemic to end the tuberculosis epidemic.. *The international journal of tuberculosis and lung disease : the official journal of the International Union against Tuberculosis and Lung Disease*. 2017//. 21:852	Level 1, Form Title/abstract screen
119	**C Susana Caxaj, Navjot K Gill**. Belonging and Mental Wellbeing Among a Rural Indian-Canadian Diaspora: Navigating Tensions in "Finding a Space of Our Own".. *Qualitative health research*. 2017//. 27:1119	Level 1, Form Title/abstract screen
120	**Ken Thorpe, Sanjula Jain, Peter Joski**. Prevalence And Spending Associated With Patients Who Have A Behavioral Health Disorder And Other Conditions.. *Health affairs (Project Hope)*. 2017//. 36:124	Level 1, Form Title/abstract screen
121	**Viviane Ben Nifla, Philippe Celli, Helene Samba, Cecile Vincent**. [Day clinic, a gateway towards risk reduction].. *Revue de l'infirmiere*. 2018//. 67:28	Level 1, Form Title/abstract screen
122	**Kara Zivin, Benjamin F Miller, Bruce Finke, Asaf Bitton, Perry Payne, Edith C Stowe, Ashok Reddy, Timothy J Day, Pauline Lapin, Janel L Jin, Laura L Sessums**. Behavioral Health and the Comprehensive Primary Care (CPC) Initiative: findings from the 2014 CPC behavioral health survey.. *BMC health services research*. 2017//. 17:612	Level 1, Form Title/abstract screen
123	**Brendan J Clark, Jacqueline Jones, K Diandra Reed, Rachel Hodapp, Ivor S Douglas, David Van Pelt, Ellen L Burnham, Marc Moss**. The Experience of Patients with Alcohol Misuse after Surviving a Critical Illness. A Qualitative Study.. *Annals of the American Thoracic Society*. 2017//. 14:1154	Level 2, Form Full Text Screening
124	**Timothy F Mott**. Lung Cancer: Screening and Evaluation of Patients With Solitary Pulmonary Nodules.. *FP essentials*. 2018//. 464:17	Level 1, Form Title/abstract screen
125	**Thomas J Stopka, Marguerite Hutcheson, Ashley Donahue**. Access to healthcare insurance and healthcare services among syringe exchange program clients in Massachusetts: qualitative findings from health navigators with the iDU ("I do") Care Collaborative.. *Harm reduction journal*. 2017//. 14:26	Level 1, Form Title/abstract screen
126	**Christine N Runyan, Amber L Hewitt, Stephen A Martin, Daniel Mullin**. Confronting the new epidemic: Integrated care for opioid use disorders.. *Families, systems & health : the journal of collaborative family healthcare*. 2017//. 35:248	Level 1, Form Title/abstract screen

127	**Julie Blandthorn, Katie James, Ellen Bowman, Yvonne Bonomo, Lisa H Amir**. Two Case Studies Illustrating a Shared Decision-Making Approach to Illicit Methamphetamine Use and Breastfeeding.. *Breastfeeding medicine : the official journal of the Academy of Breastfeeding Medicine*. 2017//. 12:381	Level 1, Form Title/abstract screen
129	**Ramin Asgary, Ramesh Naderi, Juan Wisnivesky**. Opt-Out Patient Navigation to Improve Breast and Cervical Cancer Screening Among Homeless Women.. *Journal of women's health (2002)*. 2017//. 26:999	Level 1, Form Title/abstract screen
130	**Patrick W Corrigan, Dana J Kraus, Susan A Pickett, Annie Schmidt, Ed Stellon, Erin Hantke, Juana Lorena Lara**. Using Peer Navigators to Address the Integrated Health Care Needs of Homeless African Americans With Serious Mental Illness.. *Psychiatric services (Washington, D.C.)*. 2017//. 68:264	Level 1, Form Title/abstract screen
132	**Ed Day, Luke Mitcheson**. Psychosocial interventions in opiate substitution treatment services: does the evidence provide a case for optimism or nihilism?.. *Addiction (Abingdon, England)*. 2017//. 112:1329	Level 1, Form Title/abstract screen
133	**Audrey L Jones, Maria K Mor, John P Cashy, Adam J Gordon, Gretchen L Haas, James H Jr Schaefer, Leslie R M Hausmann**. Racial/Ethnic Differences in Primary Care Experiences in Patient-Centered Medical Homes among Veterans with Mental Health and Substance Use Disorders.. *Journal of general internal medicine*. 2016//. 31:1435	Level 1, Form Title/abstract screen
134	**Rob J Fredericksen, Todd C Edwards, Jessica S Merlin, Laura E Gibbons, Deepa Rao, D Scott Batey, Lydia Dant, Edgar Paez, Anna Church, Paul K Crane, Heidi M Crane, Donald L Patrick**. Patient and provider priorities for self-reported domains of HIV clinical care.. *AIDS care*. 2015//. 27:1255	Level 1, Form Title/abstract screen
135	**Ursula Running Bear, Janette Beals, Douglas K Novins, Spero M Manson**. Alcohol detoxification completion, acceptance of referral to substance abuse treatment, and entry into substance abuse treatment among Alaska Native people.. *Addictive behaviors*. 2017//. 65:25	Level 1, Form Title/abstract screen
136	**Elizabeth Bridges, Margaret M McNeill, Nancy Munro**. Research in Review: Advancing Critical Care Practice.. *American journal of critical care : an official publication, American Association of Critical-Care Nurses*. 2016//. 26:77	Level 1, Form Title/abstract screen
138	**Robin M A Clarke, Jessica Jeffrey, Mark Grossman, Thomas Strouse, Michael Gitlin, Samuel A Skootsky**. Delivering On Accountable Care: Lessons From A Behavioral Health Program To Improve Access And Outcomes.. *Health affairs (Project Hope)*. 2016//. 35:1487	Level 1, Form Title/abstract screen
139	**Colleen L Barry, Andrew J Epstein, David A Fiellin, Liana Fraenkel, Susan H Busch**. Estimating demand for primary care-based treatment for substance and alcohol use disorders.. *Addiction (Abingdon, England)*. 2016//. 111:1376	Level 1, Form Title/abstract screen
140	**Susan W Krigel, James E Grobe, Kathy Goggin, Kari Jo Harris, Jose L Moreno, Delwyn Catley**. Motivational interviewing and the decisional balance procedure for cessation induction in smokers not intending to quit.. *Addictive behaviors*. 2017//. 64:171	Level 2, Form Full Text Screening
141	**Allyson Kelley, LaDawn K Medicine Bull, Gary LaFranier**. Participatory visual methods for American Indian communities and mental health conversations.. *American Indian and Alaska native mental health research (Online)*. 2016//. 23:47	Level 1, Form Title/abstract screen
142	**Ramzi G Salloum, Kayla R Getz, Andy S L Tan, Lisa Carter-Harris, Kelly C Young-Wolff, Thomas J Jr George, Elizabeth A Shenkman**. Use of Electronic Cigarettes Among Cancer Survivors in the U.S.. *American journal of preventive medicine*. 2016//. 51:762	Level 1, Form Title/abstract screen

143	**Hiroyuki Kimura, Yasuharu Onishi, Shinichi Kishi, Nobuhiko Kurata, Satoshi Ogiso, Hideya Kamei, Chisato Tsuboi, Naoko Yamaguchi, Azusa Shiga, Mai Kondo, Yushun Yokoyama, Fumika Takasato, Hiroshige Fujishiro, Kanako Ishizuka, Takashi Okada, Yasuhiro Ogura, Norio Ozaki**. Successful Post-Transplant Psychiatric Interventions During Long-Term Follow-Up of Patients Receiving Liver Transplants for Alcoholic Liver Disease.. *The American journal of case reports*. 2017//. 18:1215	Level 1, Form Title/abstract screen
144	**Gail L Rose, Gary J Badger, Joan M Skelly, Tonya A Ferraro, Charles D MacLean, John E Helzer**. A Randomized Controlled Trial of IVR-Based Alcohol Brief Intervention to Promote Patient-Provider Communication in Primary Care.. *Journal of general internal medicine*. 2016//. 31:996	Level 1, Form Title/abstract screen
145	**Cati G Brown-Johnson, Andrea Burbank, Eric J Daza, Arianna Wassmann, Amy Chieng, Geoffrey W Rutledge, Judith J Prochaska**. Online Patient-Provider E-cigarette Consultations: Perceptions of Safety and Harm.. *American journal of preventive medicine*. 2016//. 51:882	Level 1, Form Title/abstract screen
146	**Noe Garin, Beatriz Zurita, Cesar Velasco, Anna Feliu, Mar Gutierrez, Montserrat Masip, M Antonia Mangues**. Prevalence and clinical impact of recreational drug consumption in people living with HIV on treatment: a cross-sectional study.. *BMJ open*. 2017//. 7:e014105	Level 1, Form Title/abstract screen
147	**Neeti M Kanodra, Charlene Pope, Chanita H Halbert, Gerard A Silvestri, LaShanta J Rice, Nichole T Tanner**. Primary Care Provider and Patient Perspectives on Lung Cancer Screening. A Qualitative Study.. *Annals of the American Thoracic Society*. 2016//. 13:1977	Level 1, Form Title/abstract screen
148	**Amandine Petit, Vanessa Risler**. [The group connected bike to help fight addiction].. *Soins. Psychiatrie*. 2017//. 38:34	Level 1, Form Title/abstract screen
149	**Matthew Tobey, Julia Manasson, Kristen Decarlo, Katrina Ciraldo-Maryniuk, Jessie M Gaeta, Erica Wilson**. Homeless Individuals Approaching the End of Life: Symptoms and Attitudes.. *Journal of pain and symptom management*. 2017//. 53:738	Level 1, Form Title/abstract screen
150	**Joseph V Pergolizzi, Frederick A Curro, Nanada Col, Mary Papa Ghods, Don Vena, Robert Taylor, Frederick Naftolin, Jo Ann LeQuang**. A multicentre evaluation of an opioid patient-provider agreement.. *Postgraduate medical journal*. 2017//. 93:613	Level 1, Form Title/abstract screen
151	**Wynne Callon, Mary Catherine Beach, Somnath Saha, Geetanjali Chander, Ira B Wilson, Michael Barton Laws, Victoria Sharp, Jonathan Cohn, Richard Moore, P Todd Korthuis**. Assessing Problematic Substance Use in HIV Care: Which Questions Elicit Accurate Patient Disclosures?.. *Journal of general internal medicine*. 2016//. 31:1141	Level 1, Form Title/abstract screen
152	**Marsha Stanton, Daniel C McClughen**. The CDC Guideline and its Impact on Delivering Patient-Centered Care.. *Pain management nursing : official journal of the American Society of Pain Management Nurses*. 2017//. 18:270	Level 1, Form Title/abstract screen
153	**Kasey Claborn, Sara Becker, Susan Ramsey, Josiah Rich, Peter D Friedmann**. Mobile technology intervention to improve care coordination between HIV and substance use treatment providers: development, training, and evaluation protocol.. *Addiction science & clinical practice*. 2017//. 12:8	Level 1, Form Title/abstract screen
154	**Marjon van Rijn, Jacqueline J Suijker, Wietske Bol, Eva Hoff, Gerben Ter Riet, Sophia E de Rooij, Eric P Moll van Charante, Bianca M Buurman**. Comprehensive geriatric assessment: recognition of identified geriatric conditions by community-dwelling older persons.. *Age and ageing*. 2016//.	Level 1, Form Title/abstract screen

	45:894	
156	**Karin V Rhodes, Simon Basseyn, Robert Gallop, Elizabeth Noll, Aileen Rothbard, Paul Crits-Christoph**. Pennsylvania's Medical Home Initiative: Reductions in Healthcare Utilization and Cost Among Medicaid Patients with Medicaland Psychiatric Comorbidities.. *Journal of general internal medicine*. 2016//. 31:1373	Level 1, Form Title/abstract screen
157	**Magdalena Kulesza, Katherine E Watkins, Allison J Ober, Karen C Osilla, Brett Ewing**. Internalized stigma as an independent risk factor for substance use problems among primary care patients: Rationale and preliminary support.. *Drug and alcohol dependence*. 2017//. 180:52	Level 1, Form Title/abstract screen
158	**Ruthanne Marcus, Iuliia Makarenko, Alyona Mazhnaya, Alexei Zelenev, Maxim Polonsky, Lynn Madden, Sergii Filippovych, Sergii Dvoriak, Sandra A Springer, Frederick L Altice**. Patient preferences and extended-release naltrexone: A new opportunity to treat opioid use disorders in Ukraine.. *Drug and alcohol dependence*. 2017//. 179:213	Level 1, Form Title/abstract screen
160	**Carrie M Farmer, Shauna Stahlman, Kimberly A Hepner**. "You Should Drink Less": Frequency and Predictors of Discussions Between Providers and Patients About Reducing Alcohol Use.. *Substance use & misuse*. 2017//. 52:139	Level 1, Form Title/abstract screen
161	**Traci H Abraham, Eleanor T Lewis, Karen L Drummond, Christine Timko, Michael A Cucciare**. Providers' perceptions of barriers and facilitators to disclosure of alcohol use by women veterans.. *Primary health care research & development*. 2017//. 18:64	Level 1, Form Title/abstract screen
162	**Heather Palis, Kirsten Marchand, Defen Peng, Jill Fikowski, Scott Harrison, Patricia Spittal, Martin T Schechter, Eugenia Oviedo-Joekes**. Factors Associated with Perceived Abuse in the Health Care System Among Long-Term Opioid Users: A Cross-Sectional Study.. *Substance use & misuse*. 2016//. 51:763	Level 1, Form Title/abstract screen
163	**Will Damon, Will Small, Solanna Anderson, Lisa Maher, Evan Wood, Thomas Kerr, Ryan McNeil**. 'Crisis' and 'everyday' initiators: A qualitative study of coercion and agency in the context of methadone maintenance treatment initiation.. *Drug and alcohol review*. 2017//. 36:253	Level 2, Form Full Text Screening
164	**Rongchong Huang, Xiantao Song, Haishan Zhang, Wen Tian, Zheng Huang, Xingwei Zhang, Junqing Yang, Dongfeng Zhang, Jian Wu, Lei Zhong, Henry H Ting,SOS-COMEDY investigators**. The success of opening single chronic total occlusion lesions to improve myocardialviabilitytrial (SOS-COMEDY): Study protocol of a prospective multicenter study.. *Medicine*. 2018//. 97:e0443	Level 1, Form Title/abstract screen
166	**Inge Petersen, Lara Fairall, Arvin Bhana, Tasneem Kathree, One Selohilwe, Carrie Brooke-Sumner, Gill Faris, Erica Breuer, Nomvula Sibanyoni, Crick Lund, Vikram Patel**. Integrating mental health into chronic care in South Africa: the development of a district mental healthcare plan.. *The British journal of psychiatry : the journal of mental science*. 2016//. 208 Suppl 56:s29	Level 1, Form Title/abstract screen
167	**Vikram Patel, Somnath Chatterji**. Integrating Mental Health In Care For Noncommunicable Diseases: An Imperative For Person-Centered Care.. *Health affairs (Project Hope)*. 2015//. 34:1498	Level 1, Form Title/abstract screen
168	**Debbie Zagami, Jessica Hockenhull, Alanna Bodger, Krishna Bajee Sriram**. Communication of Pulmonary Function Test Results: A Survey of Patient's Preferences.. *PloS one*. 2015//. 10:e0126617	Level 1, Form Title/abstract screen
169	**Donna M Evon, Carol E Golin, Paul Stewart, Michael W Fried, Shani**	Level 1, Form

	**Alston, Bryce Reeve, Anna S Lok, Richard K Sterling, Joseph K Lim, Nancy Reau, Souvik Sarkar, David R Nelson, K R Reddy, Adrian M Di Bisceglie**. Patient engagement and study design of PROP UP: A multi-site patient-centered prospective observational study of patients undergoing hepatitis C treatment.. *Contemporary clinical trials*. 2017//. 57:58	Title/abstract screen
170	**Marya Viorst Gwadz, Linda M Collins, Charles M Cleland, Noelle R Leonard, Leo Wilton, Monica Gandhi, R Scott Braithwaite, David C Perlman, Alexandra Kutnick, Amanda S Ritchie**. Using the multiphase optimization strategy (MOST) to optimize an HIV care continuum intervention for vulnerable populations: a study protocol.. *BMC public health*. 2017//. 17:383	Level 1, Form Title/abstract screen
171	**R J Fredericksen, J Tufano, J Ralston, J McReynolds, M Stewart, W B Lober, K H Mayer, W C Mathews, M J Mugavero, P K Crane, H M Crane**. Provider perceptions of the value of same-day, electronic patient-reported measures for use in clinical HIV care.. *AIDS care*. 2016//. 28:1428	Level 1, Form Title/abstract screen
172	**Karriem S Watson, Amanda C Blok, Joanna Buscemi, Yamile Molina, Marian Fitzgibbon, Melissa A Simon, Lance Williams, Kameron Matthews, Jamie L Studts, Sarah E Lillie, Jamie S Ostroff, Lisa Carter-Harris, Robert A Winn**. Society of Behavioral Medicine supports implementation of high quality lung cancer screening in high-risk populations.. *Translational behavioral medicine*. 2016//. 6:669	Level 1, Form Title/abstract screen
173	**Sean M Robinson**. "Alcoholic" or "Person with alcohol use disorder"? Applying person-first diagnostic terminology in the clinical domain.. *Substance abuse*. 2017//. 38:9	Level 1, Form Title/abstract screen
174	**Nathan Crick, Joseph M Gabriel**. Medical Narrative and the Rhetoric of Identification: The Many Faces of Anna White Dildane.. *Health communication*. 2016//. 31:1318	Level 2, Form Full Text Screening
175	**Larry Davidson**. The Recovery Movement: Implications For Mental Health Care And Enabling People To Participate Fully In Life.. *Health affairs (Project Hope)*. 2016//. 35:1091	Level 1, Form Title/abstract screen
176	**Amity E Quinn, Anna D Rubinsky, Anne C Fernandez, Hyeouk Chris Hahm, Jeffrey H Samet**. A Research Agenda to Advance the Coordination of Care for General Medical and Substance Use Disorders.. *Psychiatric services (Washington, D.C.)*. 2017//. 68:400	Level 1, Form Title/abstract screen
177	**Thomas D'Aunno, Harold Pollack, Qixuan Chen, Peter D Friedmann**. Linkages Between Patient-centered Medical Homes and Addiction Treatment Organizations: Results From a National Survey.. *Medical care*. 2017//. 55:379	Level 1, Form Title/abstract screen
178	**Sohini Ghosh, M Bradley Drummond**. Electronic cigarettes as smoking cessation tool: are we there?.. *Current opinion in pulmonary medicine*. 2017//. 23:111	Level 1, Form Title/abstract screen
179	**Kiara K Spooner, Jason L Salemi, Hamisu M Salihu, Roger J Zoorob**. Discharge Against Medical Advice in the United States, 2002-2011.. *Mayo Clinic proceedings*. 2017//. 92:525	Level 1, Form Title/abstract screen
180	**Susan L Calcaterra, Anne D Drabkin, Reina Doyle, Sarah E Leslie, Ingrid A Binswanger, Joseph W Frank, Jennifer A Reich, Stephen Koester**. A Qualitative Study of Hospitalists' Perceptions of Patient Satisfaction Metrics on Pain Management.. *Hospital topics*. 2017//. 95:18	Level 1, Form Title/abstract screen
181	**G H Horner**. Mapping the road to quality collaborative patient care in a behavioral health community treatment center. Avoiding the detours of managed care.. *The Psychiatric clinics of North America*. 2000//. 23:363	Level 1, Form Title/abstract screen

182	**J N Chappel, R L DuPont**. Twelve-step and mutual-help programs for addictive disorders.. *The Psychiatric clinics of North America*. 1999//. 22:425	Level 1, Form Title/abstract screen
183	**Matthew P Romagano, William E Scorza, Stephen E Lammers, Carole Dorr, John C Smulian**. Treatment of a Pregnant Patient in a Persistent Vegetative State.. *Obstetrics and gynecology*. 2017//. 129:107	Level 1, Form Title/abstract screen
184	**Michele R Decker, Lorie Benning, Kathleen M Weber, Susan G Sherman, Adebola Adedimeji, Tracey E Wilson, Jennifer Cohen, Michael W Plankey, Mardge H Cohen, Elizabeth T Golub**. Physical and Sexual Violence Predictors: 20 Years of the Women's Interagency HIV Study Cohort.. *American journal of preventive medicine*. 2016//. 51:731	Level 1, Form Title/abstract screen
185	**Carolyn M Fratto**. Trauma-Informed Care for Youth in Foster Care.. *Archives of psychiatric nursing*. 2016//. 30:439	Level 1, Form Title/abstract screen
186	**Yiwey Shieh, Martin Bohnenkamp**. Low-Dose CT Scan for Lung Cancer Screening: Clinical and Coding Considerations.. *Chest*. 2017//. 152:204	Level 1, Form Title/abstract screen
187	**Leiyu Shi, De-Chih Lee, Michelle Chung, Hailun Liang, Diana Lock, Alek Sripipatana**. Patient-Centered Medical Home Recognition and Clinical Performance in U.S. Community Health Centers.. *Health services research*. 2017//. 52:984	Level 1, Form Title/abstract screen
188	**Stephanie A Gernant, Rachel Bastien, Andrea Lai**. Development and evaluation of a multidisciplinary controlled substances committee within a patient-centered medical home.. *Journal of the American Pharmacists Association : JAPhA*. 2015//. 55:656	Level 1, Form Title/abstract screen
189	**Johanna Chapin-Bardales, Travis Sanchez, Gabriela Paz-Bailey, Kathy Hageman, Michael W Spiller, Yadira Rolon-Colon, Sandra Miranda de Leon**. Factors Associated With Recent Human Immunodeficiency Virus Testing Among Men Who Have Sex With Men in Puerto Rico, National Human Immunodeficiency Virus Behavioral Surveillance System, 2011.. *Sexually transmitted diseases*. 2016//. 43:346	Level 1, Form Title/abstract screen
190	**Karen E Lasser, Lisa M Quintiliani, Ve Truong, Ziming Xuan, Jennifer Murillo, Cheryl Jean, Lori Pbert**. Effect of Patient Navigation and Financial Incentives on Smoking Cessation Among Primary Care Patients at an Urban Safety-Net Hospital: A Randomized Clinical Trial.. *JAMA internal medicine*. 2017//. 177:1798	Level 1, Form Title/abstract screen
191	**Bryan R Garner, Heather J Gotham, Stephen J Tueller, Elizabeth L Ball, David Kaiser, Patricia Stilen, Kathryn Speck, Denna Vandersloot, Traci R Rieckmann, Michael Chaple, Erika G Martin, Steve Martino**. Testing the effectiveness of a motivational interviewing-based brief intervention for substance use as an adjunct to usual care in community-based AIDS service organizations: study protocol for a multisite randomized controlled trial.. *Addiction science & clinical practice*. 2017//. 12:31	Level 1, Form Title/abstract screen
192	**Evan Senreich, Lydia P Ogden, Joy Pastan Greenberg**. A postgraduation follow-up of social work students trained in "SBIRT": Rates of usage and perceptions of effectiveness.. *Social work in health care*. 2017//. 56:412	Level 1, Form Title/abstract screen
193	**Yoo Mi Jeong, Cindy B Veldhuis, Frances Aranda, Tonda L Hughes**. Racial/ethnic differences in unmet needs for mental health and substance use treatment in a community-based sample of sexual minority women.. *Journal of clinical nursing*. 2016//. 25:3557	Level 1, Form Title/abstract screen

194	**Lisa Tlach, Caroline Wusten, Anne Daubmann, Sarah Liebherz, Martin Harter, Jorg Dirmaier**. Information and decision-making needs among people with mental disorders: a systematic review of the literature.. *Health expectations : an international journal of public participation in health care and health policy*. 2015//. 18:1856	Level 1, Form Title/abstract screen
195	**S Varvin**. A retrospective follow-up investigation of a group of schizophrenic patients treated in a psychotherapeutic unit: the Kastanjebakken Study.. *Psychopathology*. 1991//. 24:336	Level 1, Form Title/abstract screen
198	**Mary Brolin, Maria Torres, Dominic Hodgkin, Constance Horgan, Margaret Lee, Elizabeth Merrick, Grant Ritter, Lee Panas, Natasha DeMarco, Jonna Hopwood, Andrea Gewirtz, John Straus, Janice Harrington, Nancy Lane**. Implementation of Client Incentives within a Recovery Navigation Program.. *Journal of substance abuse treatment*. 2017//. 72:25	Level 1, Form Title/abstract screen
199	**Maxine Stitzer, Donald Calsyn, Timothy Matheson, James Sorensen, Lauren Gooden, Lisa Metsch**. Development of a Multi-Target Contingency Management Intervention for HIV Positive Substance Users.. *Journal of substance abuse treatment*. 2017//. 72:66	Level 1, Form Title/abstract screen
200	**Ann-Mari Lofthus, Heidi Westerlund, Dagfinn Bjorgen, Jonas Christoffer Lindstrom, Arnhild Lauveng, Hanne Clausen, Torleif Ruud, Kristin Sverdvik Heiervang**. Are Users Satisfied with Assertive Community Treatment in Spite of Personal Restrictions?.. *Community mental health journal*. 2016//. 52:891	Level 1, Form Title/abstract screen
202	**David Farabee, Maureen Hillhouse, Timothy Condon, Barbara McCrady, Kathryn McCollister, Walter Ling**. Injectable pharmacotherapy for opioid use disorders (IPOD).. *Contemporary clinical trials*. 2016//. 49:70	Level 1, Form Title/abstract screen
203	**Robert P Schwartz, Sharon M Kelly, Shannon G Mitchell, Laura Dunlap, Gary A Zarkin, Anjalee Sharma, Kevin E O'Grady, Jerome H Jaffe**. Interim methadone and patient navigation in jail: Rationale and design of a randomized clinical trial.. *Contemporary clinical trials*. 2016//. 49:21	Level 1, Form Title/abstract screen
204	**Redonna K Chandler, Matthew S Finger, David Farabee, Robert P Schwartz, Timothy Condon, Laura J Dunlap, Gary A Zarkin, Kathryn McCollister, Ryan D McDonald, Eugene Laska, David Bennett, Sharon M Kelly, Maureen Hillhouse, Shannon G Mitchell, Kevin E O'Grady, Joshua D Lee**. The SOMATICS collaborative: Introduction to a National Institute on Drug Abuse cooperative study of pharmacotherapy for opioid treatment in criminal justice settings.. *Contemporary clinical trials*. 2016//. 48:166	Level 1, Form Title/abstract screen
205	**Katherine E Watkins, Allison J Ober, Karen Lamp, Mimi Lind, Claude Setodji, Karen Chan Osilla, Sarah B Hunter, Colleen M McCullough, Kirsten Becker, Praise O Iyiewuare, Allison Diamant, Keith Heinzerling, Harold Alan Pincus**. Collaborative Care for Opioid and Alcohol Use Disorders in Primary Care: The SUMMIT Randomized Clinical Trial.. *JAMA internal medicine*. 2017//. 177:1480	Level 1, Form Title/abstract screen
206	**Sara L Kornfield, Marian Moseley, Dina Appleby, Courtney L McMickens, Mary D Sammel, C Neill Epperson**. Posttraumatic Symptom Reporting and Reported Cigarette Smoking During Pregnancy.. *Journal of women's health (2002)*. 2017//. 26:662	Level 1, Form Title/abstract screen
207	**Traci Rieckmann, John Muench, Mary Ann McBurnie, Michael C Leo, Phillip Crawford, Daren Ford, Jennifer Stubbs, Conall O'Cleirigh, Kenneth H Mayer, Kevin Fiscella, Nicole Wright, Maya Doe-Simkins, Matthew Cuddeback, Elizabeth Salisbury-Afshar, Christine Nelson**.	Level 1, Form Title/abstract screen

	Medication-assisted treatment for substance use disorders within a national community health center research network.. *Substance abuse*. 2016//. 37:625	
208	**Brian D Kiluk, Kelly Serafini, Tami Frankforter, Charla Nich, Kathleen M Carroll**. Only connect: The working alliance in computer-based cognitive behavioral therapy.. *Behaviour research and therapy*. 2014//. 63:139	Level 1, Form Title/abstract screen
209	**Ann Elizabeth Montgomery, Dorota Szymkowiak, Dennis Culhane**. Gender Differences in Factors Associated with Unsheltered Status and Increased Risk of Premature Mortality among Individuals Experiencing Homelessness.. *Women's health issues : official publication of the Jacobs Institute of Women's Health*. 2017//. 27:256	Level 1, Form Title/abstract screen
210	**William G Shadel, Marc N Elliott, Ann C Haas, Amelia M Haviland, Nate Orr, Melissa M Farmer, Sai Ma, Robert Weech-Maldonado, Donna O Farley, Paul D Cleary**. Clinician advice to quit smoking among seniors.. *Preventive medicine*. 2015//. 70:83	Level 1, Form Title/abstract screen
211	**Thomas B Richards, Mary C White, Ralph S Caraballo**. Lung cancer screening with low-dose computed tomography for primary care providers.. *Primary care*. 2014//. 41:307	Level 1, Form Title/abstract screen
212	**Trena I Mukherjee, Jeffrey A Wickersham, Mayur M Desai, Veena Pillai, Adeeba Kamarulzaman, Frederick L Altice**. Factors associated with interest in receiving prison-based methadone maintenance therapy in Malaysia.. *Drug and alcohol dependence*. 2016//. 164:120	Level 1, Form Title/abstract screen
213	**Daniel Ciccarone, George Jay Unick, Jenny K Cohen, Sarah G Mars, Daniel Rosenblum**. Nationwide increase in hospitalizations for heroin-related soft tissue infections: Associations with structural market conditions.. *Drug and alcohol dependence*. 2016//. 163:126	Level 1, Form Title/abstract screen
214	**Lindsay Gressard, Amy S DeGroff, Thomas B Richards, Stephanie Melillo, Julia Kish-Doto, Christina L Heminger, Elizabeth A Rohan, Kristine Gabuten Allen**. A qualitative analysis of smokers' perceptions about lung cancer screening.. *BMC public health*. 2017//. 17:589	Level 1, Form Title/abstract screen
215	**Marcel J Casavant**. Urine drug screening in adolescents.. *Pediatric clinics of North America*. 2002//. 49:317	Level 1, Form Title/abstract screen
216	**M J Werner**. Principles of brief intervention for adolescent alcohol, tobacco, and other drug use.. *Pediatric clinics of North America*. 1995//. 42:335	Level 1, Form Title/abstract screen
217	**Ming Yang, Elizabeth Wu, Huiying Rao, Fanny H Du, Angela Xie, Shanna Cheng, Cassandra Rodd, Andy Lin, Lai Wei, Anna S Lok**. A Comparative Study of Liver Disease Care in the USA and Urban and Rural China.. *Digestive diseases and sciences*. 2016//. 61:2847	Level 1, Form Title/abstract screen
218	**Patrick Vijayalakshmy, Candice Hebert, Seth Green, Charlotte L Ingram**. Integrated multidisciplinary treatment teams; a mental health model for outpatient settings in the military.. *Military medicine*. 2011//. 176:986	Level 1, Form Title/abstract screen
219	**Damon Mitchell, David Joseph Angelone**. Assessing the validity of the Stages of Change Readiness and Treatment Eagerness Scale with treatment-seeking military service members.. *Military medicine*. 2006//. 171:900	Level 1, Form Title/abstract screen
220	**Jonathan P Singer, Roger D Yusen**. Defining patient-reported outcomes in chronic obstructive pulmonary disease: the patient-centered experience.. *The Medical clinics of North America*. 2012//. 96:767	Level 1, Form Title/abstract screen
221	**Adi V Gundlapalli, Andrew Redd, Daniel Bolton, Megan E Vanneman,**	Level 1, Form

	**Marjorie E Carter, Erin Johnson, Matthew H Samore, Jamison D Fargo, Thomas P O'Toole**. Patient-aligned Care Team Engagement to Connect Veterans Experiencing Homelessness With Appropriate Health Care.. *Medical care*. 2017//. 55 Suppl 9 Suppl 2:S104	Title/abstract screen
222	**Thomas R Vetter, Zeev N Kain**. Role of the Perioperative Surgical Home in Optimizing the Perioperative Use of Opioids.. *Anesthesia and analgesia*. 2017//. 125:1653	Level 1, Form Title/abstract screen
223	**Thomas John**. Setting up recovery clinics and promoting service user involvement.. *British journal of nursing (Mark Allen Publishing)*. 2017//. 26:671	Level 1, Form Title/abstract screen
224	**S I Ovcharenko, B A Volel', Ya K Galetskaite**. [A personalized approach to the pulmonary rehabilitation of patients with chronic obstructive pulmonary disease].. *Terapevticheskii arkhiv*. 2017//. 89:18	Level 1, Form Title/abstract screen
225	**Akhil Shenoy, Jacob M Appel**. Safeguarding Confidentiality in Electronic Health Records.. *Cambridge quarterly of healthcare ethics : CQ : the international journal of healthcare ethics committees*. 2017//. 26:337	Level 1, Form Title/abstract screen
226	**Laxmaiah Manchikanti, Adam M Kaye, Nebojsa Nick Knezevic, Heath McAnally, Konstantin Slavin, Andrea M Trescot, Susan Blank, Vidyasagar Pampati, Salahadin Abdi, Jay S Grider, Alan D Kaye, Kavita N Manchikanti, Harold Cordner, Christopher G Gharibo, Michael E Harned, Sheri L Albers, Sairam Atluri, Steve M Aydin, Sanjay Bakshi, Robert L Barkin, Ramsin M Benyamin, Mark V Boswell, Ricardo M Buenaventura, Aaron K Calodney, David L Cedeno, Sukdeb Datta, Timothy R Deer, Bert Fellows, Vincent Galan, Vahid Grami, Hans Hansen, Standiford Helm Ii, Rafael Justiz, Dhanalakshmi Koyyalagunta, Yogesh Malla, Annu Navani, Kent H Nouri, Ramarao Pasupuleti, Nalini Sehgal, Sanford M Silverman, Thomas T Simopoulos, Vijay Singh, Daneshvari R Solanki, Peter S Staats, Ricardo Vallejo, Bradley W Wargo, Arthur Watanabe, Joshua A Hirsch**. Responsible, Safe, and Effective Prescription of Opioids for Chronic Non-Cancer Pain: American Society of Interventional Pain Physicians (ASIPP) Guidelines.. *Pain physician*. 2017//. 20:S3	Level 1, Form Title/abstract screen
227	**Joshua L Akers, Ryan N Hansen, Ryan D Oftebro**. Implementing take-home naloxone in an urban community pharmacy.. *Journal of the American Pharmacists Association : JAPhA*. 2017//. 57:S161	Level 1, Form Title/abstract screen
228	**Mary Beth Sutter, Sarah Gopman, Lawrence Leeman**. Patient-centered Care to Address Barriers for Pregnant Women with Opioid Dependence.. *Obstetrics and gynecology clinics of North America*. 2017//. 44:95	Level 2, Form Full Text Screening
229	**D R Aberle**. Implementing lung cancer screening: the US experience.. *Clinical radiology*. 2017//. 72:401	Level 1, Form Title/abstract screen
230	**Mette Groenkjaer, Charlotte de Crespigny, Dennis Liu, John Moss, Imelda Cairney, Deb Lee, Nicholas Procter, Cherrie Galletly**. "The Chicken or the Egg": Barriers and Facilitators to Collaborative Care for People With Comorbidity in a Metropolitan Region of South Australia.. *Issues in mental health nursing*. 2017//. 38:18	Level 1, Form Title/abstract screen
231	**Kevin Richard, Timothy Baghurst, J Michael Faragher, Erin Stotts**. Practical Treatments Considering the Role of Sociocultural Factors on Problem Gambling.. *Journal of gambling studies*. 2017//. 33:265	Level 1, Form Title/abstract screen
232	**Paulina Sockolow, Seran Schug, Jichen Zhu, T J Smith, Yalini Senathirajah, Sandra Bloom**. At-risk adolescents as experts in a new	Level 1, Form Title/abstract

	requirements elicitation procedure for the development of a smart phone psychoeducational trauma-informed care application.. *Informatics for health & social care*. 2017//. 42:77	screen
233	**David J Steiner,Thomson Reuters Accelus**. Pharmaceuticals and Medical Devices: Medicare Part D.. *Issue brief (Health Policy Tracking Service)*. 2016//. 2016:1	Level 1, Form Title/abstract screen
234	**Tanya E Baker, Grace Chang**. The use of auricular acupuncture in opioid use disorder: A systematic literature review.. *The American journal on addictions*. 2016//. 25:592	Level 1, Form Title/abstract screen
235	**Coral Sirdifield, Sara Owen, Charlie Brooker**. Engaging offenders on probation in health research: lessons from the field.. *Nurse researcher*. 2016//. 24:18	Level 1, Form Title/abstract screen
236	**Elizabeth M Camacho, Dionysios Ntais, Peter Coventry, Peter Bower, Karina Lovell, Carolyn Chew-Graham, Clare Baguley, Linda Gask, Chris Dickens, Linda M Davies**. Long-term cost-effectiveness of collaborative care (vs usual care) for people with depression and comorbid diabetes or cardiovascular disease: a Markov model informed by the COINCIDE randomised controlled trial.. *BMJ open*. 2016//. 6:e012514	Level 1, Form Title/abstract screen
237	**Pascal Wabnitz, Klaus-Thomas Kronmuller, Erwin Wieskus-Friedemann, Sabine Kliem, Johannes Hoppmann, Monika Burek, Michael Lohr, Ulrich Kemper, Andre Nienaber**. ["Nicht von schlechten Eltern - NischE": A Family Orientated Collaborative Care Approach to Support Children in Families with Mentally Ill Parents].. *Praxis der Kinderpsychologie und Kinderpsychiatrie*. 2016//. 65:668	Level 1, Form Title/abstract screen
238	**Marie-Josee Fleury, Guy Grenier, Catherine Vallee, Denise Aube, Lambert Farand, Jean-Marie Bamvita, Genevieve Cyr**. Implementation of the Quebec mental health reform (2005-2015).. *BMC health services research*. 2016//. 16:586	Level 1, Form Title/abstract screen
239	**Todd J Anderson, Jean Gregoire, Glen J Pearson, Arden R Barry, Patrick Couture, Martin Dawes, Gordon A Francis, Jacques Jr Genest, Steven Grover, Milan Gupta, Robert A Hegele, David C Lau, Lawrence A Leiter, Eva Lonn, G B John Mancini, Ruth McPherson, Daniel Ngui, Paul Poirier, John L Sievenpiper, James A Stone, George Thanassoulis, Richard Ward**. 2016 Canadian Cardiovascular Society Guidelines for the Management of Dyslipidemia for the Prevention of Cardiovascular Disease in the Adult.. *The Canadian journal of cardiology*. 2016//. 32:1263	Level 1, Form Title/abstract screen
240	**Rebecca P Barclay, Robert J Hilt**. Integrated Care for Pediatric Substance Abuse.. *Child and adolescent psychiatric clinics of North America*. 2016//. 25:769	Level 1, Form Title/abstract screen
241	**Jennifer J Bowdoin, Rosa Rodriguez-Monguio, Elaine Puleo, David Keller, Joan Roche**. Associations between the patient-centered medical home and preventive care and healthcare quality for non-elderly adults with mental illness: A surveillance study analysis.. *BMC health services research*. 2016//. 16:434	Level 1, Form Title/abstract screen
243	**Irene Tami-Maury, Aditya J Wagh, Nathalie E Abou Khalil, Ellen R Gritz, Mark S Chambers**. Dental care in Texas: An opportunity for implementing a comprehensive and patient-centric approach with special emphasis on cancer patients and survivors.. *Texas dental journal*. 2016//. 133:364	Level 1, Form Title/abstract screen
244	**Shiraz I Mishra, Andrew L Sussman, Ambroshia M Murrietta, Christina M Getrich, Robert Rhyne, Richard E Crowell, Kathryn L Taylor, Ellen J Reifler, Pamela H Wescott, Ali I Saeed, Richard M Hoffman**. Patient	Level 1, Form Title/abstract screen

	Perspectives on Low-Dose Computed Tomography for Lung Cancer Screening, New Mexico, 2014.. *Preventing chronic disease*. 2016//. 13:E108	
245	**Haq Nawaz, Paul V Petraro, Christina Via, Saif Ullah, Lionel Lim, Dorothea Wild, Mary Kennedy, Edward M Phillips**. Lifestyle medicine curriculum for a preventive medicine residency program: implementation and outcomes.. *Medical education online*. 2016//. 21:29339	Level 1, Form Title/abstract screen
246	**Leah C Susser, Stephanie A Sansone, Alison D Hermann**. Selective serotonin reuptake inhibitors for depression in pregnancy.. *American journal of obstetrics and gynecology*. 2016//. 215:722	Level 1, Form Title/abstract screen
247	**Lisa R Metsch, Daniel J Feaster, Lauren Gooden, Tim Matheson, Maxine Stitzer, Moupali Das, Mamta K Jain, Allan E Rodriguez, Wendy S Armstrong, Gregory M Lucas, Ank E Nijhawan, Mari-Lynn Drainoni, Patricia Herrera, Pamela Vergara-Rodriguez, Jeffrey M Jacobson, Michael J Mugavero, Meg Sullivan, Eric S Daar, Deborah K McMahon, David C Ferris, Robert Lindblad, Paul VanVeldhuisen, Neal Oden, Pedro C Castellon, Susan Tross, Louise F Haynes, Antoine Douaihy, James L Sorensen, David S Metzger, Raul N Mandler, Grant N Colfax, Carlos del Rio**. Effect of Patient Navigation With or Without Financial Incentives on Viral Suppression Among Hospitalized Patients With HIV Infection and Substance Use: A Randomized Clinical Trial.. *JAMA*. 2016//. 316:156	Level 1, Form Title/abstract screen
248	**Sebastiano Guarnaccia, Charvonne N Holliday, Emanuele D'Agata, Ada Pluda, Gaia Pecorelli, Valeria Gretter, Susanna Facchetti, Richard A Bilonick, Matthew G Masiello, Edmund Ricci**. Clinical and health promotion asthma management: an intervention for children and adolescents.. *Allergy and asthma proceedings*. 2016//. 37:70	Level 1, Form Title/abstract screen
249	**Denny Z H Levett, Mark Edwards, Mike Grocott, Monty Mythen**. Preparing the patient for surgery to improve outcomes.. *Best practice & research. Clinical anaesthesiology*. 2016//. 30:145	Level 1, Form Title/abstract screen
250	**Nancy Elder, Michelle Penm, Harini Pallerla, Mary Beth Vonder Meulen, Amy Diane Short, Tiffiny Diers, Ryan Joseph Imhoff, Brendan Wilson, Jill Martin Boone**. Provision of Recommended Chronic Pain Assessment and Management in Primary Care: Does Patient-Centered Medical Home (PCMH) Recognition Make a Difference?.. *Journal of the American Board of Family Medicine : JABFM*. 2016//. 29:474	Level 1, Form Title/abstract screen
251	**Kyle L Olesek, Jared Outcalt, Giancarlo Dimaggio, Raffaele Popolo, Sunita George, Paul H Lysaker**. Cluster B Personality Disorder Traits as a Predictor of Therapeutic Alliance Over Time in Residential Treatment for Substance Use Disorders.. *The Journal of nervous and mental disease*. 2016//. 204:736	Level 1, Form Title/abstract screen
252	**Ashley Archiopoli, Tamar Ginossar, Bryan Wilcox, Magdalena Avila, Ricky Hill, John Oetzel**. Factors of interpersonal communication and behavioral health on medication self-efficacy and medication adherence.. *AIDS care*. 2016//. 28:1607	Level 1, Form Title/abstract screen
253	**Sainza Garcia, Monica Martinez-Cengotitabengoa, Saioa Lopez-Zurbano, Inaki Zorrilla, Purificacion Lopez, Eduard Vieta, Ana Gonzalez-Pinto**. Adherence to Antipsychotic Medication in Bipolar Disorder and Schizophrenic Patients: A Systematic Review.. *Journal of clinical psychopharmacology*. 2016//. 36:355	Level 1, Form Title/abstract screen
254	**Kathleen L Ruchalski, Kathleen Brown**. Lung Cancer Screening Update.. *Journal of thoracic imaging*. 2016//. 31:190	Level 1, Form Title/abstract screen

255	**Kathy Puskar, Ann M Mitchell, Susan A Albrecht, Linda R Frank, Irene Kane, Holly Hagle, Dawn Lindsay, Heeyoung Lee, Marie Fioravanti, Kimberly S Talcott**. Interprofessional collaborative practice incorporating training for alcohol and drug use screening for healthcare providers in rural areas.. *Journal of interprofessional care*. 2016//. 30:542	Level 1, Form Title/abstract screen
256	**Jayne Kotz, Ailsa Munns, Rhonda Marriott, Julia V Marley**. Perinatal depression and screening among Aboriginal Australians in the Kimberley.. *Contemporary nurse*. 2016//. 52:42	Level 1, Form Title/abstract screen
258	**Santina Wheat, Dorothy Dschida, Mary R Talen**. Psychiatric Emergencies.. *Primary care*. 2016//. 43:341	Level 1, Form Title/abstract screen
259	**Elizabeth W Cozine, John M Wilkinson**. Depression Screening, Diagnosis, and Treatment Across the Lifespan.. *Primary care*. 2016//. 43:229	Level 1, Form Title/abstract screen
260	**Victoria A Anyikwa**. Trauma-Informed Approach to Survivors of Intimate Partner Violence.. *Journal of evidence-informed social work*. 2016//. 13:484	Level 1, Form Title/abstract screen
261	**Kulnaree Hanpatchaiyakul, Henrik Eriksson, Jureerat Kijsomporn, Gunnel Ostlund**. Healthcare providers' experiences of working with alcohol addiction treatment in Thailand.. *Contemporary nurse*. 2016//. 52:59	Level 1, Form Title/abstract screen
262	**Douglas F Zatzick, Joan Russo, Doyanne Darnell, David A Chambers, Lawrence Palinkas, Erik Van Eaton, Jin Wang, Leah M Ingraham, Roxanne Guiney, Patrick Heagerty, Bryan Comstock, Lauren K Whiteside, Gregory Jurkovich**. An effectiveness-implementation hybrid trial study protocol targeting posttraumatic stress disorder and comorbidity.. *Implementation science : IS*. 2016//. 11:58	Level 1, Form Title/abstract screen
263	**Francoise Labat, Anjali Sharma**. Qualitative study exploring surgical team members' perception of patient safety in conflict-ridden Eastern Democratic Republic of Congo.. *BMJ open*. 2016//. 6:e009379	Level 1, Form Title/abstract screen
264	**Lisa Rapp**. Delinquent-Victim Youth-Adapting a Trauma-Informed Approach for the Juvenile Justice System.. *Journal of evidence-informed social work*. 2016//. 13:492	Level 1, Form Title/abstract screen
266	**Sacha Agrawal, Pat Capponi, Jenna Lopez, Sean Kidd, Charlotte Ringsted, David Wiljer, Sophie Soklaridis**. From Surviving to Advising: A Novel Course Pairing Mental Health and Addictions Service Users as Advisors to Senior Psychiatry Residents.. *Academic psychiatry : the journal of the American Association of Directors of Psychiatric Residency Training and the Association for Academic Psychiatry*. 2016//. 40:475	Level 1, Form Title/abstract screen
267	**Jenna A LoGiudice, Sherifa Douglas**. Incorporation of Sexual Violence in Nursing Curricula Using Trauma-Informed Care: A Case Study.. *The Journal of nursing education*. 2016//. 55:215	Level 1, Form Title/abstract screen
269	**John F Kelly, M Claire Greene, Brandon G Bergman**. Recovery benefits of the "therapeutic alliance" among 12-step mutual-help organization attendees and their sponsors.. *Drug and alcohol dependence*. 2016//. 162:64	Level 1, Form Title/abstract screen
270	**Babak Tofighi, Ellie Grossman, Scott Sherman, Edward V Nunes, Joshua D Lee**. Mobile Phone Messaging During Unobserved "Home" Induction to Buprenorphine.. *Journal of addiction medicine*. 2016//. 10:309	Level 1, Form Title/abstract screen
271	**Anja Gysin-Maillart, Simon Schwab, Leila Soravia, Millie Megert, Konrad Michel**. A Novel Brief Therapy for Patients Who Attempt Suicide: A 24-	Level 1, Form Title/abstract

	months Follow-Up Randomized Controlled Study of the Attempted Suicide Short Intervention Program (ASSIP).. *PLoS medicine*. 2016//. 13:e1001968	screen
272	**David A Richards, Peter Bower, Carolyn Chew-Graham, Linda Gask, Karina Lovell, John Cape, Stephen Pilling, Ricardo Araya, David Kessler, Michael Barkham, J Martin Bland, Simon Gilbody, Colin Green, Glyn Lewis, Chris Manning, Evangelos Kontopantelis, Jacqueline J Hill, Adwoa Hughes-Morley, Abigail Russell**. Clinical effectiveness and cost-effectiveness of collaborative care for depression in UK primary care (CADET): a cluster randomised controlled trial.. *Health technology assessment (Winchester, England)*. 2016//. 20:1	Level 1, Form Title/abstract screen
273	**Sven Wahlin, Sara Wallhed Finn, Sven Andreasson**. [Stop stigmatizing alcohol problems].. *Lakartidningen*. 2016//. 113:#pages#	Level 1, Form Title/abstract screen
275	**Claudia Lin Xiao, Emorfia Gavrilidis, Stuart Lee, Jayashri Kulkarni**. Do mental health clinicians elicit a history of previous trauma in female psychiatric inpatients?.. *Journal of mental health (Abingdon, England)*. 2016//. 25:359	Level 1, Form Title/abstract screen
276	**Kelly A Aschbrenner, John A Naslund, Lydia E Gill, Stephen J Bartels, Dror Ben-Zeev**. A Qualitative Study of Client-Clinician Text Exchanges in a Mobile Health Intervention for Individuals With Psychotic Disorders and Substance Use.. *Journal of dual diagnosis*. 2016//. 12:63	Level 1, Form Title/abstract screen
277	**Valerie Noel, Mary Woods, Jonathan Routhier, Robert Drake**. Planning Treatment and Assessing Recovery in Participants With Dual Diagnosis: Preliminary Evaluation of a New Clinical Tool.. *Journal of dual diagnosis*. 2016//. 12:55	Level 1, Form Title/abstract screen
278	**Barbara St Marie**. Primary care experiences of people who live with chronic pain and receive opioids to manage pain: A qualitative methodology.. *Journal of the American Association of Nurse Practitioners*. 2016//. 28:429	Level 1, Form Title/abstract screen
279	**Jean Yoon, Adam Chow, Lisa V Rubenstein**. Impact of Medical Home Implementation Through Evidence-based Quality Improvement on Utilization and Costs.. *Medical care*. 2016//. 54:118	Level 1, Form Title/abstract screen
280	**Rebekah Sypniewski**. Motivational Interviewing: A Practical Intervention for School Nurses to Engage in Trauma Informed Care.. *NASN school nurse (Print)*. 2016//. 31:40	Level 1, Form Title/abstract screen
282	**Katherine E Lubarsky, Jonathan D Avery**. Dignity Therapy for Alcohol Use Disorder.. *The American journal of psychiatry*. 2016//. 173:90	Level 2, Form Full Text Screening
283	**Babak Tofighi, Ellie Grossman, Sewit Bereket, Joshua D Lee**. Text message content preferences to improve buprenorphine maintenance treatment in primary care.. *Journal of addictive diseases*. 2016//. 35:92	Level 1, Form Title/abstract screen
284	**David Mechanic, Mark Olfson**. The Relevance of the Affordable Care Act for Improving Mental Health Care.. *Annual review of clinical psychology*. 2016//. 12:515	Level 1, Form Title/abstract screen
285	**JoAn R Laes**. The Integration of Medical Toxicology and Addiction Medicine: a New Era in Patient Care.. *Journal of medical toxicology : official journal of the American College of Medical Toxicology*. 2016//. 12:79	Level 1, Form Title/abstract screen
286	**Marjolein Snaterse, Jos Dobber, Patricia Jepma, Ron J G Peters, Gerben Ter Riet, S Matthijs Boekholdt, Bianca M Buurman, Wilma J M Scholte op Reimer**. Effective components of nurse-coordinated care to prevent recurrent coronary events: a systematic review and meta-analysis.. *Heart (British Cardiac*	Level 1, Form Title/abstract screen

	Society). 2016//. 102:50	
288	**Ryan McNeil, Thomas Kerr, Bernie Pauly, Evan Wood, Will Small**. Advancing patient-centered care for structurally vulnerable drug-using populations: a qualitative study of the perspectives of people who use drugs regarding the potential integration of harm reduction interventions into hospitals.. *Addiction (Abingdon, England)*. 2016//. 111:685	Level 2, Form Full Text Screening
289	**Jamie Chang, Leslie Dubbin, Janet Shim**. Negotiating substance use stigma: the role of cultural health capital in provider-patient interactions.. *Sociology of health & illness*. 2016//. 38:90	Level 2, Form Full Text Screening
290	**Susan E Collins, Connor B Jones, Gail Hoffmann, Lonnie A Nelson, Starlyn M Hawes, Veronique S Grazioli, Jessica L Mackelprang, Jessica Holttum, Greta Kaese, James Lenert, Patrick Herndon, Seema L Clifasefi**. In their own words: Content analysis of pathways to recovery among individuals with the lived experience of homelessness and alcohol use disorders.. *The International journal on drug policy*. 2016//. 27:89	Level 1, Form Title/abstract screen
291	**Colleen T LaBelle, Steve Choongheon Han, Alexis Bergeron, Jeffrey H Samet**. Office-Based Opioid Treatment with Buprenorphine (OBOT-B): Statewide Implementation of the Massachusetts Collaborative Care Model in Community Health Centers.. *Journal of substance abuse treatment*. 2016//. 60:6	Level 1, Form Title/abstract screen
292	**Allysha C Robinson, Amy R Knowlton**. Gender Differences in Psychosocial Factors Associated with HIV Viral Suppression Among African-American Injection Drug Users.. *AIDS and behavior*. 2016//. 20:385	Level 1, Form Title/abstract screen
293	**John F Kelly, M Claire Greene, Brandon Bergman, Bettina B Hoeppner, Valerie Slaymaker**. The Sponsor Alliance Inventory: Assessing the Therapeutic Bond Between 12-Step Attendees and Their Sponsors.. *Alcohol and alcoholism (Oxford, Oxfordshire)*. 2016//. 51:32	Level 1, Form Title/abstract screen
294	**Gerald Cochran, Adam J Gordon, Craig Field, Jennifer Bacci, Ranjita Dhital, Thomas Ylioja, Maxine Stitzer, Thomas Kelly, Ralph Tarter**. Developing a framework of care for opioid medication misuse in community pharmacy.. *Research in social & administrative pharmacy : RSAP*. 2016//. 12:293	Level 1, Form Title/abstract screen
295	**Melissa L Anderson, Neil S Glickman, Lisa A Mistler, Marco Gonzalez**. Working therapeutically with deaf people recovering from trauma and addiction.. *Psychiatric rehabilitation journal*. 2016//. 39:27	Level 1, Form Title/abstract screen
296	**Marc L Steinberg, Jill M Williams, Naomi F Stahl, Patricia Dooley Budsock, Nina A Cooperman**. An Adaptation of Motivational Interviewing Increases Quit Attempts in Smokers With Serious Mental Illness.. *Nicotine & tobacco research : official journal of the Society for Research on Nicotine and Tobacco*. 2016//. 18:243	Level 1, Form Title/abstract screen
297	**Jill Sederstrom**. IT PAYS TO BE PATIENT-CENTERED AND TRAUMA-INFORMED.. *Behavioral healthcare*. 2015//. 35:28	Level 2, Form Full Text Screening
298	**Sally Bradford, Debra Rickwood**. Acceptability and utility of an electronic psychosocial assessment (myAssessment) to increase self-disclosure in youth mental healthcare: a quasi-experimental study.. *BMC psychiatry*. 2015//. 15:305	Level 1, Form Title/abstract screen
299	**Annie Lewis-O'Connor, Mardi Chadwick**. Engaging the Voice of Patients Affected by Gender-Based Violence: Informing Practice and Policy.. *Journal of forensic nursing*. 2015//. 11:240	Level 1, Form Title/abstract screen
300	**Geraldine S Whittaker**. An educational approach for "non-compliant"	Level 1, Form

	patients.. *The Canadian journal of critical care nursing*. 2015//. 26:11	Title/abstract screen
301	**Salim S Virani, Thomas M Maddox, Paul S Chan, Fengming Tang, Julia M Akeroyd, Samantha A Risch, William J Oetgen, Anita Deswal, Biykem Bozkurt, Christie M Ballantyne, Laura A Petersen**. Provider Type and Quality of Outpatient Cardiovascular Disease Care: Insights From the NCDR PINNACLE Registry.. *Journal of the American College of Cardiology*. 2015//. 66:1803	Level 1, Form Title/abstract screen
302	**Pia Rydell, Pontus Stralin**. [Investigation and follow-up requires collaboration].. *Lakartidningen*. 2015//. 112:#pages#	Level 1, Form Title/abstract screen
303	**Li-Tzy Wu, Udi E Ghitza, Bryan C Batch, Michael J Pencina, Leoncio Flavio Rojas, Benjamin A Goldstein, Tony Schibler, Ashley A Dunham, Shelley Rusincovitch, Kathleen T Brady**. Substance use and mental diagnoses among adults with and without type 2 diabetes: Results from electronic health records data.. *Drug and alcohol dependence*. 2015//. 156:162	Level 1, Form Title/abstract screen
304	**Lisa M Quintiliani, Zlatka L Russinova, Philippe P Bloch, Ve Truong, Ziming Xuan, Lori Pbert, Karen E Lasser**. Patient navigation and financial incentives to promote smoking cessation in an underserved primary care population: A randomized controlled trial protocol.. *Contemporary clinical trials*. 2015//. 45:449	Level 1, Form Title/abstract screen
305	**David Roll, Margaret Spottswood, Hsiang Huang**. Using Shared Medical Appointments to Increase Access to Buprenorphine Treatment.. *Journal of the American Board of Family Medicine : JABFM*. 2015//. 28:676	Level 1, Form Title/abstract screen
306	**Antoinette Krupski, Imara I West, Meredith C Graves, David C Atkins, Charles Maynard, Kristin Bumgardner, Dennis Donovan, Richard Ries, Peter Roy-Byrne**. Clinical Needs of Patients with Problem Drug Use.. *Journal of the American Board of Family Medicine : JABFM*. 2015//. 28:605	Level 1, Form Title/abstract screen
307	**P Staccini, L Fernandez-Luque**. Health Social Media and Patient-Centered Care: Buzz or Evidence? Findings from the Section "Education and Consumer Health Informatics" of the 2015 Edition of the IMIA Yearbook.. *Yearbook of medical informatics*. 2015//. 10:160	Level 1, Form Title/abstract screen
308	**Carla Kaufmann, Stefan Markun, Susann Hasler, Kaba Dalla Lana, Thomas Rosemann, Oliver Senn, Claudia Steurer-Stey**. Performance Measures in the Management of Chronic Obstructive Pulmonary Disease in Primary Care--A Retrospective Analysis.. *Praxis*. 2015//. 104:897	Level 1, Form Title/abstract screen
309	**Anne Kveim Lie, Terje Jensen, Hanne Lichtwarck, Jon Storaas, Erik Torjussen, Ingrid Amalia Havnes**. [Putting the patient at the center--already as a student].. *Tidsskrift for den Norske laegeforening : tidsskrift for praktisk medicin, ny raekke*. 2015//. 135:1236	Level 2, Form Full Text Screening
310	**Rita Kukafka, In cheol Jeong, Joseph Finkelstein**. Optimizing Decision Support for Tailored Health Behavior Change Applications.. *Studies in health technology and informatics*. 2015//. 216:108	Level 1, Form Title/abstract screen
311	**Michael S Martin, Gordana Eljdupovic, Kwame McKenzie, Ian Colman**. Risk of violence by inmates with childhood trauma and mental health needs.. *Law and human behavior*. 2015//. 39:614	Level 1, Form Title/abstract screen
312	**Bravein Amalakuhan, Sandra G Adams**. Improving outcomes in chronic obstructive pulmonary disease: the role of the interprofessional approach.. *International journal of chronic obstructive pulmonary disease*. 2015//. 10:1225	Level 1, Form Title/abstract screen

313	**Michael P Schaub, Larissa J Maier, Andreas Wenger, Lars Stark, Oliver Berg, Thilo Beck, Boris B Quednow, Severin Haug**. Evaluating the efficacy of a web-based self-help intervention with and without chat counseling in reducing the cocaine use of problematic cocaine users: the study protocol of a pragmatic three-arm randomized controlled trial.. *BMC psychiatry*. 2015//. 15:156	Level 1, Form Title/abstract screen
314	**Colleen Anne Dell, Nancy Poole**. Taking a PAWS to Reflect on How the Work of a Therapy Dog Supports a Trauma-Informed Approach to Prisoner Health.. *Journal of forensic nursing*. 2015//. 11:167	Level 1, Form Title/abstract screen
315	**Jennifer L Saylor, Kathleen A Schell, Mark F Mendell, Jennifer S Graber**. Development of a Clinical Competency Checklist for Care of Patients Experiencing Substance Withdrawal Delirium or Delirium: Use of a Delphi Technique and Expert Panel.. *Journal of psychosocial nursing and mental health services*. 2015//. 53:29	Level 2, Form Full Text Screening
316	**Sasa Brankovic**. Boredom, dopamine, and the thrill of psychosis: psychiatry in a new key.. *Psychiatria Danubina*. 2015//. 27:126	Level 1, Form Title/abstract screen
317	**Terry Shih, Gaetano Paone, Patricia F Theurer, Donna McDonald, David M Shahian, Richard L Prager**. The Society of Thoracic Surgeons Adult Cardiac Surgery Database Version 2.73: More Is Better.. *The Annals of thoracic surgery*. 2015//. 100:516	Level 1, Form Title/abstract screen
318	**Michael J Barry**. A Shared Decision-making Intervention: Success or Failure?.. *Anesthesiology*. 2015//. 123:5	Level 1, Form Title/abstract screen
319	**Kristen P Morie, Charla Nich, Karen Hunkele, Marc N Potenza, Kathleen M Carroll**. Alexithymia level and response to computer-based training in cognitive behavioral therapy among cocaine-dependent methadone maintained individuals.. *Drug and alcohol dependence*. 2015//. 152:157	Level 1, Form Title/abstract screen
320	**Iris Torchalla, Isabelle Aube Linden, Verena Strehlau, Erika K Neilson, Michael Krausz**. "Like a lots happened with my whole childhood": violence, trauma, and addiction in pregnant and postpartum women from Vancouver's Downtown Eastside.. *Harm reduction journal*. 2015//. 12:1	Level 1, Form Title/abstract screen
321	**Roxana-Maria Nemes, Paraschiva Postolache, Adeline Tintila, F D Mihaltan, F D Petrariu**. Aspects of physician-patient communication in the program of smoking cessation.. *Revista medico-chirurgicala a Societatii de Medici si Naturalisti din Iasi*. 2015//. 119:23	Level 2, Form Full Text Screening
322	**Katherine Berry, Lynsey Gregg, Rosalyn Hartwell, Gillian Haddock, Mike Fitzsimmons, Christine Barrowclough**. Therapist-client relationships in a psychological therapy trial for psychosis and substance misuse.. *Drug and alcohol dependence*. 2015//. 152:170	Level 1, Form Title/abstract screen
323	**Allison J Ober, Katherine E Watkins, Sarah B Hunter, Karen Lamp, Mimi Lind, Claude M Setodji**. An organizational readiness intervention and randomized controlled trial to test strategies for implementing substance use disorder treatment into primary care: SUMMIT study protocol.. *Implementation science : IS*. 2015//. 10:66	Level 2, Form Full Text Screening
324	**Katherine L Mills**. The importance of providing trauma-informed care in alcohol and other drug services.. *Drug and alcohol review*. 2015//. 34:231	Level 1, Form Title/abstract screen
325	**Mark van Veen, Bauke Koekkoek, Niels Mulder, Debby Postulart, Eddy Adang, Steven Teerenstra, Lisette Schoonhoven, Theo van Achterberg**.	Level 1, Form Title/abstract

	Cost effectiveness of interpersonal community psychiatric treatment for people with long-term severe non-psychotic mental disorders: protocol of a multi-centre randomized controlled trial.. *BMC psychiatry*. 2015//. 15:100	screen
326	**Bernadette Bernie Pauly, Jane McCall, Annette J Browne, J Parker, Ashley Mollison**. Toward cultural safety: nurse and patient perceptions of illicit substance use in a hospitalized setting.. *ANS. Advances in nursing science*. 2015//. 38:121	Level 2, Form Full Text Screening
327	**Andrew J Epstein, Colleen L Barry, David A Fiellin, Susan H Busch**. Consumers' Valuation of Primary Care-Based Treatment Options for Mental and Substance Use Disorders.. *Psychiatric services (Washington, D.C.)*. 2015//. 66:772	Level 1, Form Title/abstract screen
330	**Robert J Smith, Karin Rhodes, Breah Paciotti, Sheila Kelly, Jeanmarie Perrone, Zachary F Meisel**. Patient Perspectives of Acute Pain Management in the Era of the Opioid Epidemic.. *Annals of emergency medicine*. 2015//. 66:246	Level 1, Form Title/abstract screen
331	**Bethany A DiPaula, Elizabeth Menachery**. Physician-pharmacist collaborative care model for buprenorphine-maintained opioid-dependent patients.. *Journal of the American Pharmacists Association : JAPhA*. 2015//. 55:187	Level 1, Form Title/abstract screen
332	**Teresa Lopez-Castro, Mei-Chen Hu, Santiago Papini, Lesia M Ruglass, Denise A Hien**. Pathways to change: Use trajectories following trauma-informed treatment of women with co-occurring post-traumatic stress disorder and substance use disorders.. *Drug and alcohol review*. 2015//. 34:242	Level 2, Form Full Text Screening
334	**Susan E Collins, Veronique S Grazioli, Nicole I Torres, Emily M Taylor, Connor B Jones, Gail E Hoffman, Laura Haelsig, Mengdan D Zhu, Alyssa S Hatsukami, Molly J Koker, Patrick Herndon, Shawna M Greenleaf, Parker E Dean**. Qualitatively and quantitatively evaluating harm-reduction goal setting among chronically homeless individuals with alcohol dependence.. *Addictive behaviors*. 2015//. 45:184	Level 1, Form Title/abstract screen
335	**Robert J Smith, Austin S Kilaru, Jeanmarie Perrone, Breah Paciotti, Frances K Barg, Sarah M Gadsden, Zachary F Meisel**. How, why, and for whom do emergency medicine providers use prescription drug monitoring programs?.. *Pain medicine (Malden, Mass.)*. 2015//. 16:1122	Level 1, Form Title/abstract screen
336	**Kimber P Richter, Edward F Ellerbeck**. Response to commentaries: changing the treatment default will improve decision-making.. *Addiction (Abingdon, England)*. 2015//. 110:390	Level 1, Form Title/abstract screen
337	**Jennifer Morton, Marjorie Withers, Shelley Cohen Konrad, Carry Buterbaugh, RuthAnne Spence**. Bridging the gaps: An early integrated support collaborative for at risk mothers in rural Maine.. *Work (Reading, Mass.)*. 2015//. 50:413	Level 1, Form Title/abstract screen
338	**Mark D Schwartz, Ashley Jensen, Binhuan Wang, Katelyn Bennett, Anne Dembitzer, Shiela Strauss, Antoinette Schoenthaler, Colleen Gillespie, Scott Sherman**. Panel Management to Improve Smoking and Hypertension Outcomes by VA Primary Care Teams: A Cluster-Randomized Controlled Trial.. *Journal of general internal medicine*. 2015//. 30:916	Level 1, Form Title/abstract screen
339	**Brady J McKee, Andrea B McKee, Andrea Borondy Kitts, Shawn M Regis, Christoph Wald**. Low-dose computed tomography screening for lung cancer in a clinical setting: essential elements of a screening program.. *Journal of thoracic imaging*. 2015//. 30:115	Level 1, Form Title/abstract screen
340	**Alyson H Sheehan, Myrna L Friedlander**. Therapeutic Alliance and Retention in Brief Strategic Family Therapy: A Mixed-Methods Study.. *Journal*	Level 1, Form Title/abstract

	of marital and family therapy. 2015//. 41:415	screen
341	**Elizabeth K McClain, Erin Jewell Burks**. Managing attention-deficit/hyperactivity disorder in children and adolescents.. *Primary care*. 2015//. 42:99	Level 1, Form Title/abstract screen
342	**Pamela J Biernacki, Mary T Champagne, Shane Peng, David R Maizel, Barbara S Turner**. Transformation of Care: Integrating the Registered Nurse Care Coordinator into the Patient-Centered Medical Home.. *Population health management*. 2015//. 18:330	Level 1, Form Title/abstract screen
343	**Paula C G Alves, Celia M D Sales, Mark Ashworth**. Personalising the evaluation of substance misuse treatment: a new approach to outcome measurement.. *The International journal on drug policy*. 2015//. 26:333	Level 1, Form Title/abstract screen
344	**M Lascaux, O Phan**. [Comparison of European therapies for cannabis addiction among adolescents].. *L'Encephale*. 2015//. 41 Suppl 1:S21	Level 2, Form Full Text Screening
346	**John C Fortney, Jeffrey M Pyne, Timothy A Kimbrell, Teresa J Hudson, Dean E Robinson, Ronald Schneider, William M Moore, Paul J Custer, Kathleen M Grubbs, Paula P Schnurr**. Telemedicine-based collaborative care for posttraumatic stress disorder: a randomized clinical trial.. *JAMA psychiatry*. 2015//. 72:58	Level 1, Form Title/abstract screen
347	**Johanne Eliacin, Michelle P Salyers, Marina Kukla, Marianne S Matthias**. Factors influencing patients' preferences and perceived involvement in shared decision-making in mental health care.. *Journal of mental health (Abingdon, England)*. 2015//. 24:24	Level 1, Form Title/abstract screen
348	**Amy E Whittle, Sara M Buckelew, Jason M Satterfield, Paula J Lum, Patricia O'Sullivan**. Addressing Adolescent Substance Use: Teaching Screening, Brief Intervention, and Referral to Treatment (SBIRT) and Motivational Interviewing (MI) to Residents.. *Substance abuse*. 2015//. 36:325	Level 1, Form Title/abstract screen
349	**Eriphylli Argyra, Ioanna Siafaka, Astero Moutzouri, Vassilios Papadopoulos, Martina Rekatsina, Athina Vadalouca, Kassiani Theodoraki**. How does an undergraduate pain course influence future physicians' awareness of chronic pain concepts? A comparative study.. *Pain medicine (Malden, Mass.)*. 2015//. 16:301	Level 1, Form Title/abstract screen
350	**Matthew S Kendra, Kenneth R Weingardt, Michael A Cucciare, Christine Timko**. Satisfaction with substance use treatment and 12-step groups predicts outcomes.. *Addictive behaviors*. 2015//. 40:27	Level 1, Form Title/abstract screen
351	**Barbara K Campbell, Joseph Guydish, Thao Le, Elizabeth A Wells, Dennis McCarty**. The relationship of therapeutic alliance and treatment delivery fidelity with treatment retention in a multisite trial of twelve-step facilitation.. *Psychology of addictive behaviors : journal of the Society of Psychologists in Addictive Behaviors*. 2015//. 29:106	Level 1, Form Title/abstract screen
352	**Louisa G Sylvia, Richard C Shelton, David E Kemp, Emily E Bernstein, Edward S Friedman, Benjamin D Brody, Susan L McElroy, Vivek Singh, Mauricio Tohen, Charles L Bowden, Terence A Ketter, Thilo Deckersbach, Michael E Thase, Noreen A Reilly-Harrington, Andrew A Nierenberg, Dustin J Rabideau, Gustavo Kinrys, James H Kocsis, William V Bobo, Masoud Kamali, Melvin G McInnis, Joseph R Calabrese**. Medical burden in bipolar disorder: findings from the Clinical and Health Outcomes Initiative in Comparative Effectiveness for Bipolar Disorder study (Bipolar CHOICE).. *Bipolar disorders*. 2015//. 17:212	Level 1, Form Title/abstract screen
353	**William R Miller, Theresa B Moyers**. The forest and the trees: relational and	Level 2, Form

	specific factors in addiction treatment.. *Addiction (Abingdon, England)*. 2015//. 110:401	Full Text Screening
354	**Ingrid A Binswanger, Elizabeth Whitley, Paul-Ryan Haffey, Shane R Mueller, Sung-Joon Min**. A patient navigation intervention for drug-involved former prison inmates.. *Substance abuse*. 2015//. 36:34	Level 1, Form Title/abstract screen
356	**Volker Tschuschke, Aureliano Crameri, Miriam Koehler, Jessica Berglar, Katharina Muth, Pia Staczan, Agnes Von Wyl, Peter Schulthess, Margit Koemeda-Lutz**. The role of therapists' treatment adherence, professional experience, therapeutic alliance, and clients' severity of psychological problems: Prediction of treatment outcome in eight different psychotherapy approaches. Preliminary results of a naturalistic study.. *Psychotherapy research : journal of the Society for Psychotherapy Research*. 2015//. 25:420	Level 1, Form Title/abstract screen
357	**Michael J Penkunas, Stephen Hahn-Smith**. An Evaluation of IMPACT for the Treatment of Late-Life Depression in a Public Mental Health System.. *The journal of behavioral health services & research*. 2015//. 42:334	Level 1, Form Title/abstract screen
358	**Celine Dosissard, Jerome Lipari**. [Emergency departments and deliberate medicinal self-poisoning].. *Revue de l'infirmiere*. 2014//. #volume#:18	Level 1, Form Title/abstract screen
359	**Mara Buchbinder, Rachel Wilbur, Diana Zuskov, Samuel McLean, Betsy Sleath**. Teachable moments and missed opportunities for smoking cessation counseling in a hospital emergency department: a mixed-methods study of patient-provider communication.. *BMC health services research*. 2014//. 14:651	Level 1, Form Title/abstract screen
360	**Julie Viveros, Joan Kub**. Interview with Julie Viveros, RN, Director of Nursing, Charlotte Rescue Mission, Rebound men's program. Interview by Joan Kub.. *Journal of addictions nursing*. 2014//. 25:211	Level 2, Form Full Text Screening
361	**C F Rueda-Clausen, E Benterud, T Bond, R Olszowka, M T Vallis, A M Sharma**. Effect of implementing the 5As of obesity management framework on provider-patient interactions in primary care.. *Clinical obesity*. 2014//. 4:39	Level 1, Form Title/abstract screen
362	**Arlene Lovejoy-Bluem**. Drug-exposed newborns and caregivers:quality care collaborative for the mother-baby-community triad.. *Neonatal network : NN*. 2014//. 33:356	Level 1, Form Title/abstract screen
364	**Dror Ben-Zeev, Susan M Kaiser, Izabela Krzos**. Remote "hovering" with individuals with psychotic disorders and substance use: feasibility, engagement, and therapeutic alliance with a text-messaging mobile interventionist.. *Journal of dual diagnosis*. 2014//. 10:197	Level 2, Form Full Text Screening
365	**Iain Atherton, Richard Kyle**. How empathy skills can change nursing.. *Nursing standard (Royal College of Nursing (Great Britain) : 1987)*. 2014//. 29:24	Level 1, Form Title/abstract screen
366	**Siobhan M Phillips, Russell E Glasgow, Ghalib Bello, Marcia G Ory, Beth A Glenn, Sherri N Sheinfeld-Gorin, Roy T Sabo, Suzanne Heurtin-Roberts, Sallie Beth Johnson, Alex H Krist,MOHR Study Group**. Frequency and prioritization of patient health risks from a structured health risk assessment.. *Annals of family medicine*. 2014//. 12:505	Level 1, Form Title/abstract screen
367	**Anonymous**. Unique program aims to connect frequent ED utilizers with medical homes, resources to meet complex needs.. *ED management : the monthly update on emergency department management*. 2014//. 26:117	Level 1, Form Title/abstract screen
368	**Stephen A Maisto, Megan Kirouac, Katie Witkiewitz**. Alcohol use disorder clinical course research: informing clinicians' treatment planning now and in the future.. *Journal of studies on alcohol and drugs*. 2014//. 75:799	Level 1, Form Title/abstract screen

369	**Rodger Kessler, Benjamin F Miller, Mark Kelly, Debbie Graham, Amanda Kennedy, Benjamin Littenberg, Charles D MacLean, Constance van Eeghen, Sarah Hudson Scholle, Manasi Tirodkar, Suzanne Morton, Wilson D Pace**. Mental health, substance abuse, and health behavior services in patient-centered medical homes.. *Journal of the American Board of Family Medicine : JABFM*. 2014//. 27:637	Level 1, Form Title/abstract screen
370	**Nathan D Shippee, Brooke H Rosen, Kurt B Angstman, Manuel E Fuentes, Ramona S DeJesus, Steven M Bruce, Mark D Williams**. Baseline screening tools as indicators for symptom outcomes and health services utilization in a collaborative care model for depression in primary care: a practice-based observational study.. *General hospital psychiatry*. 2014//. 36:563	Level 1, Form Title/abstract screen
371	**David E Arterburn, Anita P Courcoulas**. Bariatric surgery for obesity and metabolic conditions in adults.. *BMJ (Clinical research ed.)*. 2014//. 349:g3961	Level 1, Form Title/abstract screen
372	**Laura P Richardson, Evette Ludman, Elizabeth McCauley, Jeff Lindenbaum, Cindy Larison, Chuan Zhou, Greg Clarke, David Brent, Wayne Katon**. Collaborative care for adolescents with depression in primary care: a randomized clinical trial.. *JAMA*. 2014//. 312:809	Level 1, Form Title/abstract screen
373	**Patrick W Corrigan, Susan Pickett, Karen Batia, Patrick J Michaels**. Peer navigators and integrated care to address ethnic health disparities of people with serious mental illness.. *Social work in public health*. 2014//. 29:581	Level 1, Form Title/abstract screen
374	**Judith Haber, Andrew I Spielman, Mark Wolff, Donna Shelley**. Interprofessional education between dentistry and nursing: the NYU experience.. *Journal of the California Dental Association*. 2014//. 42:44	Level 1, Form Title/abstract screen
375	**Kate Traynor**. Rhode Island's opioid epidemic response features collaborative practice model.. *American journal of health-system pharmacy : AJHP : official journal of the American Society of Health-System Pharmacists*. 2014//. 71:1328	Level 1, Form Title/abstract screen
376	**A Karger**. [Gender differences in depression].. *Bundesgesundheitsblatt, Gesundheitsforschung, Gesundheitsschutz*. 2014//. 57:1092	Level 1, Form Title/abstract screen
377	**Leena Mittal**. Buprenorphine for the treatment of opioid dependence in pregnancy.. *The Journal of perinatal & neonatal nursing*. 2014//. 28:178	Level 1, Form Title/abstract screen
378	**Sharon Levy, Janet F Williams**. Adolescent substance use: the role of the medical home.. *Adolescent medicine: state of the art reviews*. 2014//. 25:1	Level 1, Form Title/abstract screen
379	**Dolly A John, Ichiro Kawachi, Christopher S Lathan, John Z Ayanian**. Disparities in perceived unmet need for supportive services among patients with lung cancer in the Cancer Care Outcomes Research and Surveillance Consortium.. *Cancer*. 2014//. 120:3178	Level 1, Form Title/abstract screen
380	**Elisa Becze**. Centering on wellness. Tobacco cessation and whole-person healing put the focus on the patient.. *ONS connect*. 2014//. 29:26	Level 2, Form Full Text Screening
381	**Zev Schuman-Olivier, Roger D Weiss, Bettina B Hoeppner, Jacob Borodovsky, Mark J Albanese**. Emerging adult age status predicts poor buprenorphine treatment retention.. *Journal of substance abuse treatment*. 2014//. 47:202	Level 1, Form Title/abstract screen
382	**Joji Suzuki, Michele L Matthews, David Brick, Minh-Thuy Nguyen, Ajay D Wasan, Robert N Jamison, Andrew L Ellner, Lori W Tishler, Roger D**	Level 1, Form Title/abstract

	**Weiss**. Implementation of a collaborative care management program with buprenorphine in primary care: a comparison between opioid-dependent patients and patients with chronic pain using opioids nonmedically.. *Journal of opioid management*. 2014//. 10:159	screen
383	**Frederic Ketterer, Linda Symons, Marie-Claire Lambrechts, Philippe Mairiaux, Lode Godderis, Lieve Peremans, Roy Remmen, Marc Vanmeerbeek**. What factors determine Belgian general practitioners' approaches to detecting and managing substance abuse? A qualitative study based on the I-Change Model.. *BMC family practice*. 2014//. 15:119	Level 1, Form Title/abstract screen
384	**Jay I Kumar, Melody Anthony, Steven A Crawford, Ronald A Arky, Asaf Bitton, Garth L Splinter**. A comparison of Tier 1 and Tier 3 medical homes under Oklahoma Medicaid program.. *The Journal of the Oklahoma State Medical Association*. 2014//. 107:157	Level 1, Form Title/abstract screen
385	**Kathleen P Decker, Stephanie L Peglow, Carl R Samples**. Participation in a novel treatment component during residential substance use treatment is associated with improved outcome: a pilot study.. *Addiction science & clinical practice*. 2014//. 9:7	Level 2, Form Full Text Screening
386	**Richard L Brown, Paul D Moberg, Joyce B Allen, Candace T Peterson, Laura A Saunders, Mia D Croyle, Robin M Lecoanet, Sarah M Linnan, Kim Briedenbach, Scott B Caldwell**. A team approach to systematic behavioral screening and intervention.. *The American journal of managed care*. 2014//. 20:e113	Level 1, Form Title/abstract screen
387	**Howard Epstein, Carmen Hansen, David Thorson**. A protocol for addressing acute pain and prescribing opioids.. *Minnesota medicine*. 2014//. 97:47	Level 1, Form Title/abstract screen
388	**Vijay Singh, Ketti Petersen, Simone Rauscher Singh**. Intimate partner violence victimization: identification and response in primary care.. *Primary care*. 2014//. 41:261	Level 1, Form Title/abstract screen
389	**Katharine A Bradley, Daniel R Kivlahan**. Bringing patient-centered care to patients with alcohol use disorders.. *JAMA*. 2014//. 311:1861	Level 1, Form Title/abstract screen
390	**Yasmin Senturias, Barbara Burns**. Managing children and adolescents with fetal alcohol spectrum disorders in the medical home.. *Current problems in pediatric and adolescent health care*. 2014//. 44:96	Level 1, Form Title/abstract screen
391	**Jacob L Taylor, Rebeccah A McKibben, Matthew DeCamp, Margaret S Chisolm**. Putting a face on the prescription opioid epidemic: a case report.. *General hospital psychiatry*. 2014//. 36:449.e1	Level 2, Form Full Text Screening
392	**Douglas Zatzick, Joan Russo, Sarah Peregrine Lord, Christopher Varley, Jin Wang, Lucy Berliner, Gregory Jurkovich, Lauren K Whiteside, Stephen O'Connor, Frederick P Rivara**. Collaborative care intervention targeting violence risk behaviors, substance use, and posttraumatic stress and depressive symptoms in injured adolescents: a randomized clinical trial.. *JAMA pediatrics*. 2014//. 168:532	Level 1, Form Title/abstract screen
393	**Dail Fields, Terry C Blum, Paul M Roman**. Dissemination activities: a critical new role for substance abuse treatment organizations.. *The journal of behavioral health services & research*. 2014//. 41:473	Level 1, Form Title/abstract screen
394	**Joanna L Starrels, Bryan Wu, Deena Peyser, Aaron D Fox, Abigail Batchelder, Frances K Barg, Julia H Arnsten, Chinazo O Cunningham**. It made my life a little easier: primary care providers' beliefs and attitudes about using opioid treatment agreements.. *Journal of opioid management*. 2014//.	Level 1, Form Title/abstract screen

	10:95	
395	**Melissa M Farmer, Danielle E Rose, Lisa V Rubenstein, Ismelda A Canelo, Gordon Schectman, Richard Stark, Elizabeth M Yano**. Challenges facing primary care practices aiming to implement patient-centered medical homes.. *Journal of general internal medicine*. 2014//. 29 Suppl 2:S555	Level 1, Form Title/abstract screen
396	**Jolynne Jo Carter, Annette C Watson, Patrice V Sminkey**. Pain management: screening and assessment of pain as part of a comprehensive case management process.. *Professional case management*. 2014//. 19:126	Level 1, Form Title/abstract screen
398	**Claudia Steurer-Stey, Stefan Markun, Kaba Dalla Lana, Anja Frei, Ulrike Held, Michel Wensing, Thomas Rosemann**. The improving care in chronic obstructive lung disease study: CAROL improving processes of care and quality of life of COPD patients in primary care: study protocol for a randomized controlled trial.. *Trials*. 2014//. 15:96	Level 1, Form Title/abstract screen
399	**Sofie L Champassak, Kathy Goggin, Sarah Finocchario-Kessler, Maghen Farris, Maniza Ehtesham, Rachel Schoor, Delwyn Catley**. A qualitative assessment of provider perspectives on smoking cessation counselling.. *Journal of evaluation in clinical practice*. 2014//. 20:281	Level 1, Form Title/abstract screen
400	**Lawrence A Palinkas, Angela M Robertson, Jennifer L Syvertsen, Daniel O Hernandez, Monica D Ulibarri, M Gudelia Rangel, Gustavo Martinex, Steffanie A Strathdee**. Client perspectives on design and implementation of a couples-based intervention to reduce sexual and drug risk behaviors among female sex workers and their noncommercial partners in Tijuana and Ciudad Juarez, Mexico.. *AIDS and behavior*. 2014//. 18:583	Level 1, Form Title/abstract screen
401	**I Gilles, B Bejaoui, N Courvoisier, A Clemence**. Inhabitants' and professionals' social representations of health determinants in a disadvantaged urban area in France: a qualitative analysis.. *Revue d'epidemiologie et de sante publique*. 2014//. 62:5	Level 1, Form Title/abstract screen
403	**Nena Messina, Stacy Calhoun, Jeremy Braithwaite**. Trauma-informed treatment decreases posttraumatic stress disorder among women offenders.. *Journal of trauma & dissociation : the official journal of the International Society for the Study of Dissociation (ISSD)*. 2014//. 15:6	Level 1, Form Title/abstract screen
404	**Stephanie Rodgers, Martin Grosse Holtforth, Mario Muller, Michael P Hengartner, Wulf Rossler, Vladeta Ajdacic-Gross**. Symptom-based subtypes of depression and their psychosocial correlates: a person-centered approach focusing on the influence of sex.. *Journal of affective disorders*. 2014//. 156:92	Level 1, Form Title/abstract screen
405	**Ted J Johnson, David H Sanders, Judy L Stange**. The Affordable Care Act for behavioral health consumers and families.. *Journal of social work in disability & rehabilitation*. 2014//. 13:110	Level 1, Form Title/abstract screen
406	**Daniel Almirall, Jan A Blalock**. Summary of the SRNT treatment network Webinar: getting SMART about developing individualized sequences of health interventions.. *Nicotine & tobacco research : official journal of the Society for Research on Nicotine and Tobacco*. 2014//. 16:252	Level 1, Form Title/abstract screen
407	**Wan-Sen Yan, Yong-Hui Li, Lin Xiao, Ning Zhu, Antoine Bechara, Nan Sui**. Working memory and affective decision-making in addiction: a neurocognitive comparison between heroin addicts, pathological gamblers and healthy controls.. *Drug and alcohol dependence*. 2014//. 134:194	Level 1, Form Title/abstract screen
408	**Hyun-Hee Heo, Kathryn L Braun**. Culturally tailored interventions of chronic disease targeting Korean Americans: a systematic review.. *Ethnicity & health*. 2014//. 19:64	Level 1, Form Title/abstract screen

409	**Billie Bonevski**. System-centred tobacco management: from 'whole-person' to 'whole-system' change.. *Drug and alcohol review*. 2014//. 33:99	Level 2, Form Full Text Screening
410	**Gillian S Gould**. Patient-centred tobacco management.. *Drug and alcohol review*. 2014//. 33:93	Level 2, Form Full Text Screening
411	**Van L King, Robert K Brooner, Jessica M Peirce, Ken Kolodner, Michael S Kidorf**. A randomized trial of Web-based videoconferencing for substance abuse counseling.. *Journal of substance abuse treatment*. 2014//. 46:36	Level 1, Form Title/abstract screen
412	**Kurt B Angstman, Sara Oberhelman, James E Rohrer, Matthew R Meunier, Norman H Rasmussen, Daniel H Chappell**. Depression remission decreases outpatient utilization at 6 and 12 months after enrollment into collaborative care management.. *Population health management*. 2014//. 17:48	Level 1, Form Title/abstract screen
413	**Kirstyn M Kameg, Nadine Cozzo Englert, Valerie M Howard, Katherine J Perozzi**. Fusion of psychiatric and medical high fidelity patient simulation scenarios: effect on nursing student knowledge, retention of knowledge, and perception.. *Issues in mental health nursing*. 2013//. 34:892	Level 1, Form Title/abstract screen
414	**Stella M Savarimuthu, Ashley E Jensen, Antoinette Schoenthaler, Anne Dembitzer, Craig Tenner, Colleen Gillespie, Mark D Schwartz, Scott E Sherman**. Developing a toolkit for panel management: improving hypertension and smoking cessation outcomes in primary care at the VA.. *BMC family practice*. 2013//. 14:176	Level 1, Form Title/abstract screen
415	**Neelam Mabood, Samina Ali, Kathryn A Dong, T Cameron Wild, Amanda S Newton**. Experiences of pediatric emergency physicians in providing alcohol-related care to adolescents in the emergency department.. *Pediatric emergency care*. 2013//. 29:1260	Level 1, Form Title/abstract screen
416	**Karen E Lasser, Karey S Kenst, Lisa M Quintiliani, Renda Soylemez Wiener, Jennifer Murillo, Lori Pbert, Ziming Xuan, Deborah J Bowen**. Patient navigation to promote smoking cessation among low-income primary care patients: a pilot randomized controlled trial.. *Journal of ethnicity in substance abuse*. 2013//. 12:374	Level 1, Form Title/abstract screen
417	**Amber Cornforth**. COPD self-management supportive care: chaos and complexity theory.. *British journal of nursing (Mark Allen Publishing)*. 2013//. 22:1101	Level 1, Form Title/abstract screen
418	**Thomas P O'Toole, Claire Bourgault, Erin E Johnson, Stephen G Redihan, Matthew Borgia, Riccardo Aiello, Vincent Kane**. New to care: demands on a health system when homeless veterans are enrolled in a medical home model.. *American journal of public health*. 2013//. 103 Suppl 2:S374	Level 1, Form Title/abstract screen
419	**Susanne Buhse, Tabitha Heller, Jurgen Kasper, Ingrid Muhlhauser, Ulrich Alfons Muller, Thomas Lehmann, Matthias Lenz**. An evidence-based shared decision making programme on the prevention of myocardial infarction in type 2 diabetes: protocol of a randomised-controlled trial.. *BMC family practice*. 2013//. 14:155	Level 1, Form Title/abstract screen
420	**Nickeisha Clarke, Eun-Young Mun, Shalonda Kelly, Helene R White, Katherine Lynch**. Treatment outcomes of a combined cognitive behavior therapy and pharmacotherapy for a sample of women with and without substance abuse histories on an acute psychiatric unit: do therapeutic alliance and motivation matter?.. *The American journal on addictions*. 2013//. 22:566	Level 1, Form Title/abstract screen
423	**Jennifer K Manuel, Howard Newville, Sandra E Larios, James L Sorensen**. Confidentiality protections versus collaborative care in the treatment of	Level 1, Form Title/abstract

	substance use disorders.. *Addiction science & clinical practice*. 2013//. 8:13	screen
424	**Janice Pettie**. Toxicology had to be the focus, not psychological care.. *Nursing standard (Royal College of Nursing (Great Britain) : 1987)*. 2013//. 27:33	Level 1, Form Title/abstract screen
425	**David A Richards, Jacqueline J Hill, Linda Gask, Karina Lovell, Carolyn Chew-Graham, Peter Bower, John Cape, Stephen Pilling, Ricardo Araya, David Kessler, J Martin Bland, Colin Green, Simon Gilbody, Glyn Lewis, Chris Manning, Adwoa Hughes-Morley, Michael Barkham**. Clinical effectiveness of collaborative care for depression in UK primary care (CADET): cluster randomised controlled trial.. *BMJ (Clinical research ed.)*. 2013//. 347:f4913	Level 1, Form Title/abstract screen
426	**Ted Nirenberg, Richard Longabaugh, Janette Baird, Michael J Mello**. Treatment may influence self-report and jeopardize our understanding of outcome.. *Journal of studies on alcohol and drugs*. 2013//. 74:770	Level 1, Form Title/abstract screen
427	**William S Blau**. The needle in a haystack: appropriate use of interventional techniques in the management of chronic pain.. *North Carolina medical journal*. 2013//. 74:215	Level 1, Form Title/abstract screen
428	**David L Bell, David J Breland, Mary A Ott**. Adolescent and young adult male health: a review.. *Pediatrics*. 2013//. 132:535	Level 1, Form Title/abstract screen
429	**Laura R Lander, Patrick Marshalek, Miheret Yitayew, Dolly Ford, Carl R Sullivan, Kelly K Gurka**. Rural healthcare disparities: challenges and solutions for the pregnant opioid-dependent population.. *The West Virginia medical journal*. 2013//. 109:22	Level 1, Form Title/abstract screen
430	**Sarah Biggs**. Don't overlook psychological care in paracetamol overdose.. *Nursing standard (Royal College of Nursing (Great Britain) : 1987)*. 2013//. 27:33	Level 1, Form Title/abstract screen
431	**Marcia S Seeberg, Mark Scarbecz, Timothy L Hottel**. An innovative behavioral science curriculum at the University of Tennessee College of Dentistry.. *The Journal of the Tennessee Dental Association*. 2013//. 93:31	Level 1, Form Title/abstract screen
432	**Cheryl J Ho, Charles Preston, Kim Fredericks, Sara L Doorley, Richard J Kramer, Lawrence Kwan, Ahmad Kamal**. A unique model for treating chronic hepatitis C in patients with psychiatric disorders, substance abuse, and/or housing instability.. *Journal of addiction medicine*. 2013//. 7:320	Level 1, Form Title/abstract screen
433	**K Van Royen, R Remmen, M Vanmeerbeek, L Godderis, P Mairiaux, L Peremans**. A review of guidelines for collaboration in substance misuse management.. *Occupational medicine (Oxford, England)*. 2013//. 63:445	Level 1, Form Title/abstract screen
434	**Marica Ferri, Marina Davoli, Roberto D'Amico**. Involving patients in setting the research agenda in drug addiction.. *BMJ (Clinical research ed.)*. 2013//. 347:f4513	Level 1, Form Title/abstract screen
435	**Tonelle E Handley, Frances J Kay-Lambkin, Amanda L Baker, Terry J Lewin, Brian J Kelly, Kerry J Inder, John R Attia, David J Kavanagh**. Incidental treatment effects of CBT on suicidal ideation and hopelessness.. *Journal of affective disorders*. 2013//. 151:275	Level 2, Form Full Text Screening
436	**Carmela Salomon, Bridget Hamilton**. "All roads lead to medication?" Qualitative responses from an Australian first-person survey of antipsychotic discontinuation.. *Psychiatric rehabilitation journal*. 2013//. 36:160	Level 1, Form Title/abstract screen
437	**Ashly E Jordan, Carmen L Masson, Pedro Mateu-Gelabert, Courtney McKnight, Nicole Pepper, Katie Bouche, Laura Guzman, Evan Kletter,**	Level 1, Form Title/abstract

	**Randy M Seewald, Don C Des-Jarlais, James L Sorensen, David C Perlman**. Perceptions of drug users regarding hepatitis C screening and care: a qualitative study.. *Harm reduction journal*. 2013//. 10:10	screen
439	**Leah Farrell-Carnahan, Jennifer Hettema, Justin Jackson, Shivi Kamalanathan, Lee M Ritterband, Karen S Ingersoll**. Feasibility and promise of a remote-delivered preconception motivational interviewing intervention to reduce risk for alcohol-exposed pregnancy.. *Telemedicine journal and e-health : the official journal of the American Telemedicine Association*. 2013//. 19:597	Level 1, Form Title/abstract screen
440	**Marsha Snyder, Lois Platt**. Substance use and brain reward mechanisms in older adults.. *Journal of psychosocial nursing and mental health services*. 2013//. 51:15	Level 1, Form Title/abstract screen
441	**Abigail Zuger**. Talking to patients in the 21st century.. *JAMA*. 2013//. 309:2384	Level 1, Form Title/abstract screen
443	**Eric D Shirley, James O Sanders**. Patient satisfaction: Implications and predictors of success.. *The Journal of bone and joint surgery. American volume*. 2013//. 95:e69	Level 1, Form Title/abstract screen
444	**Jeanne Miranda, Michael K Ong, Loretta Jones, Bowen Chung, Elizabeth L Dixon, Lingqi Tang, Jim Gilmore, Cathy Sherbourne, Victoria K Ngo, Susan Stockdale, Esmeralda Ramos, Thomas R Belin, Kenneth B Wells**. Community-partnered evaluation of depression services for clients of community-based agencies in under-resourced communities in Los Angeles.. *Journal of general internal medicine*. 2013//. 28:1279	Level 1, Form Title/abstract screen
445	**Areej Hassan, Emily A Blood, Aaron Pikcilingis, Emily G Krull, LaQuita McNickles, Glenn Marmon, Sarah Wylie, Elizabeth R Woods, Eric W Fleegler**. Youths' health-related social problems: concerns often overlooked during the medical visit.. *The Journal of adolescent health : official publication of the Society for Adolescent Medicine*. 2013//. 53:265	Level 1, Form Title/abstract screen
446	**Adam Isaiah Newman, Shelley Beckstead, David Beking, Susan Finch, Tina Knorr, Carol Lynch, Meredith MacKenzie, Daphne Mayer, Brenda Melles, Ron Shore**. Treatment of chronic hepatitis C infection among current and former injection drug users within a multidisciplinary treatment model at a community health centre.. *Canadian journal of gastroenterology = Journal canadien de gastroenterologie*. 2013//. 27:217	Level 1, Form Title/abstract screen
447	**William C Becker, Liana Fraenkel, Robert D Kerns, David A Fiellin**. A research agenda for enhancing appropriate opioid prescribing in primary care.. *Journal of general internal medicine*. 2013//. 28:1364	Level 1, Form Title/abstract screen
448	**Frank R Dillon**. Measurement equivalence of the Revised Helping Alliance Questionnaire across African American and non-Latino White substance using adult outpatients.. *Journal of substance abuse treatment*. 2013//. 45:173	Level 1, Form Title/abstract screen
449	**Alexandre B Laudet, Keith Humphreys**. Promoting recovery in an evolving policy context: what do we know and what do we need to know about recovery support services?.. *Journal of substance abuse treatment*. 2013//. 45:126	Level 1, Form Title/abstract screen
450	**Samantha Wolfe, Frances Kay-Lambkin, Jenny Bowman, Steven Childs**. To enforce or engage: the relationship between coercion, treatment motivation and therapeutic alliance within community-based drug and alcohol clients.. *Addictive behaviors*. 2013//. 38:2187	Level 1, Form Title/abstract screen
451	**David R Veroff, Tamara Ochoa-Arvelo, Benjamin Venator**. A randomized study of telephonic care support in populations at risk for musculoskeletal	Level 1, Form Title/abstract

	preference-sensitive surgeries.. *BMC medical informatics and decision making*. 2013//. 13:21	screen
452	**Kristin A Hom, Stephanie J Woods**. Trauma and its aftermath for commercially sexually exploited women as told by front-line service providers.. *Issues in mental health nursing*. 2013//. 34:75	Level 1, Form Title/abstract screen
453	**Richard Wender, Elizabeth T H Fontham, Ermilo Jr Barrera, Graham A Colditz, Timothy R Church, David S Ettinger, Ruth Etzioni, Christopher R Flowers, G Scott Gazelle, Douglas K Kelsey, Samuel J LaMonte, James S Michaelson, Kevin C Oeffinger, Ya-Chen Tina Shih, Daniel C Sullivan, William Travis, Louise Walter, Andrew M D Wolf, Otis W Brawley, Robert A Smith**. American Cancer Society lung cancer screening guidelines.. *CA: a cancer journal for clinicians*. 2013//. 63:107	Level 1, Form Title/abstract screen
455	**Harlan Matusow, Andrew Rosenblum**. The most critical unresolved issue associated with: psychoanalytic theories of addiction: can the talking cure tell us anything about substance use and misuse?.. *Substance use & misuse*. 2013//. 48:239	Level 1, Form Title/abstract screen
456	**Teresa M Salgado, Rebekah Moles, Shalom I Benrimoj, Fernando Fernandez-Llimos**. Exploring the role of pharmacists in outpatient dialysis centers: a qualitative study of nephrologist views.. *Nephrology, dialysis, transplantation : official publication of the European Dialysis and Transplant Association - European Renal Association*. 2013//. 28:397	Level 1, Form Title/abstract screen
457	**R McLaurin, S Geraghty**. Placenta praevia, placental abruption and amphetamine use in pregnancy: a case study.. *Women and birth : journal of the Australian College of Midwives*. 2013//. 26:138	Level 1, Form Title/abstract screen
458	**Jessica De Maeyer, Chijs van Nieuwenhuizen, Ilja L Bongers, Eric Broekaert, Wouter Vanderplasschen**. Profiles of quality of life in opiate-dependent individuals after starting methadone treatment: a latent class analysis.. *The International journal on drug policy*. 2013//. 24:342	Level 1, Form Title/abstract screen
459	**Karine Bertrand, Natacha Brunelle, Isabelle Richer, Isabelle Beaudoin, Annie Lemieux, Jean-Marc Menard**. Assessing covariates of drug use trajectories among adolescents admitted to a drug addiction center: mental health problems, therapeutic alliance, and treatment persistence.. *Substance use & misuse*. 2013//. 48:117	Level 1, Form Title/abstract screen
460	**Stuart J Lee, Elizabeth Crowther, Charlotte Keating, Jayashri Kulkarni**. What is needed to deliver collaborative care to address comorbidity more effectively for adults with a severe mental illness?.. *The Australian and New Zealand journal of psychiatry*. 2013//. 47:333	Level 1, Form Title/abstract screen
462	**Jon M Houck, Theresa B Moyers, Claudia D Tesche**. Through a glass darkly: some insights on change talk via magnetoencephalography.. *Psychology of addictive behaviors : journal of the Society of Psychologists in Addictive Behaviors*. 2013//. 27:489	Level 1, Form Title/abstract screen
463	**Barbara K Campbell, Jennifer K Manuel, Sarah Turcotte Manser, K Michelle Peavy, Julija Stelmokas, Dennis McCarty, Joseph R Guydish**. Assessing fidelity of treatment delivery in group and individual 12-step facilitation.. *Journal of substance abuse treatment*. 2013//. 44:169	Level 1, Form Title/abstract screen
464	**Ameera Aldossary, Louise Barriball, Alison While**. The perceived health promotion practice of nurses in Saudi Arabia.. *Health promotion international*. 2013//. 28:431	Level 1, Form Title/abstract screen
465	**Matthew Brensilver, Shabana Tariq, Steven Shoptaw**. Optimizing pain management through collaborations with behavioral and addiction medicine in	Level 1, Form Title/abstract

	primary care.. *Primary care*. 2012//. 39:661	screen
466	**Michelle Anne Bholat, Lara Ray, Matthew Brensilver, Kimberly Ling, Steven Shoptaw**. Integration of behavioral medicine in primary care.. *Primary care*. 2012//. 39:605	Level 1, Form Title/abstract screen
467	**Adam Pearson, Jon Lurie, Tor Tosteson, Wenyan Zhao, William Abdu, James N Weinstein**. Who should have surgery for spinal stenosis? Treatment effect predictors in SPORT.. *Spine*. 2012//. 37:1791	Level 1, Form Title/abstract screen
468	**Justin B Hunt, Geoffrey Curran, Teresa Kramer, Sip Mouden, Susan Ward-Jones, Richard Owen, John Fortney**. Partnership for implementation of evidence-based mental health practices in rural federally qualified health centers: theory and methods.. *Progress in community health partnerships : research, education, and action*. 2012//. 6:389	Level 1, Form Title/abstract screen
469	**Robert J Ursano, David M Benedek, Charles C Engel**. Trauma-informed care for primary care: the lessons of war.. *Annals of internal medicine*. 2012//. 157:905	Level 1, Form Title/abstract screen
470	**Pierre C Baehni**. Translating science into action--prevention of periodontal disease at patient level.. *Periodontology 2000*. 2012//. 60:162	Level 1, Form Title/abstract screen
471	**Chih-Lin Chi, W Nick Street, Jennifer G Robinson, Matthew A Crawford**. Individualized patient-centered lifestyle recommendations: an expert system for communicating patient specific cardiovascular risk information and prioritizing lifestyle options.. *Journal of biomedical informatics*. 2012//. 45:1164	Level 1, Form Title/abstract screen
472	**David B Cooper, Jo Cooper**. "Palliative care can sustain and support mental health clients".. *Nursing times*. 2012//. 108:11	Level 1, Form Title/abstract screen
473	**Rosalyn Stewart, Leonard Feldman, Daniel Bitzel, M Christopher Gibbons, Maura McGuire**. Urban health and primary care at Johns Hopkins: urban primary care medical home resident training programs.. *Journal of health care for the poor and underserved*. 2012//. 23:103	Level 1, Form Title/abstract screen
474	**Kathleen F Harrington, Julie A McDougal, Maria Pisu, Bin Zhang, Rajani S Sadasivam, Thomas K Houston, William C Bailey,CHART Collaborative Group**. Web-based smoking cessation intervention that transitions from inpatient to outpatient: study protocol for a randomized controlled trial.. *Trials*. 2012//. 13:123	Level 1, Form Title/abstract screen
475	**Frances J Kay-Lambkin, Amanda L Baker, Alison Healey, Samantha Wolfe, Aaron Simpson, Michelle Brooks, Jenny Bowman, Steven Childs**. Study protocol: a dissemination trial of computerized psychological treatment for depression and alcohol/other drug use comorbidity in an Australian clinical service.. *BMC psychiatry*. 2012//. 12:77	Level 1, Form Title/abstract screen
476	**John Muench, Kelly Jarvis, Josh Boverman, Joseph Hardman, Meg Hayes, Jim Winkle**. Tilling the soil while sowing the seeds: combining resident education with medical home transformation.. *Substance abuse*. 2012//. 33:282	Level 1, Form Title/abstract screen
477	**Jennifer Askew Buxton, Alison Chandler-Altendorf, Antonio E Puente**. A novel collaborative practice model for treatment of mental illness in indigent and uninsured patients.. *American journal of health-system pharmacy : AJHP : official journal of the American Society of Health-System Pharmacists*. 2012//. 69:1054	Level 1, Form Title/abstract screen
478	**Robert L Dupont, Gregory E Skipper**. Six lessons from state physician health programs to promote long-term recovery.. *Journal of psychoactive drugs*.	Level 2, Form Full Text

	2012//. 44:72	Screening
479	**Ernest Rasyidi, Jeffery N Wilkins, Itai Danovitch**. Training the next generation of providers in addiction medicine.. *The Psychiatric clinics of North America*. 2012//. 35:461	Level 1, Form Title/abstract screen
480	**David R Pating, Michael M Miller, Eric Goplerud, Judith Martin, Douglas M Ziedonis**. New systems of care for substance use disorders: treatment, finance, and technology under health care reform.. *The Psychiatric clinics of North America*. 2012//. 35:327	Level 1, Form Title/abstract screen
481	**Larry Culpepper**. Does screening for depression in primary care improve outcome?.. *Current psychiatry reports*. 2012//. 14:345	Level 1, Form Title/abstract screen
482	**Todd Molfenter, Victor A Capoccia, Michael G Boyle, Carol K Sherbeck**. The readiness of addiction treatment agencies for health care reform.. *Substance abuse treatment, prevention, and policy*. 2012//. 7:16	Level 1, Form Title/abstract screen
483	**Viviane Rohart**. [Modifying the relationship with psychoactive substances].. *Revue de l'infirmiere*. 2012//. #volume#:22	Level 1, Form Title/abstract screen
484	**Marie-Line Raynal, Fabienne Plancon, Claire du Lac, Sylvie Fourchault, Fabienne Rinaudo, Caroline Buchsbaum, Didier Touzeau**. [Alcohol, an addiction like any other].. *Revue de l'infirmiere*. 2012//. #volume#:14	Level 2, Form Full Text Screening
485	**Jason E Bonner, A Sidney 4th Barritt, Michael W Fried, Donna M Evon**. Tangible resources for preparing patients for antiviral therapy for chronic hepatitis C.. *Digestive diseases and sciences*. 2012//. 57:1439	Level 1, Form Title/abstract screen
486	**Nicole M Capezza, Lisa M Najavits**. Rates of trauma-informed counseling at substance abuse treatment facilities: reports from over 10,000 programs.. *Psychiatric services (Washington, D.C.)*. 2012//. 63:390	Level 2, Form Full Text Screening
488	**Aleksandra Zgierska, Michael Miller, David Rabago**. Patient satisfaction, prescription drug abuse, and potential unintended consequences.. *JAMA*. 2012//. 307:1377	Level 1, Form Title/abstract screen
489	**Mary Rosedale, Dolores Malaspina, Daniel Malamud, Shiela M Strauss, Jaclyn D Horne, Salman Abouzied, Ricardo A Cruciani, Helena Knotkova**. Developing patient-centered treatment protocols in brain stimulation: a rationale for combining quantitative and qualitative approaches in persons with HIV.. *Journal of the American Psychiatric Nurses Association*. 2012//. 18:166	Level 1, Form Title/abstract screen
490	**Susan Kaiser**. Psychiatric and addiction consultation for patients in critical care.. *Critical care nursing clinics of North America*. 2012//. 24:9	Level 1, Form Title/abstract screen
491	**Amal Abdel-Baki, Clairelaine Ouellet-Plamondon, Ashok Malla**. Pharmacotherapy challenges in patients with first-episode psychosis.. *Journal of affective disorders*. 2012//. 138 Suppl:S3	Level 1, Form Title/abstract screen
492	**Melou Jansen, Marleen M E M van Doorn, Anna Lichtwarck-Aschoff, Rowella C W M Kuijpers, Huub Theunissen, Mirjam Korte, Jose van Rossum, Annemiek Wauben, Isabela Granic**. Effectiveness of a cognitive-behavioral therapy (CBT) manualized program for clinically anxious children: study protocol of a randomized controlled trial.. *BMC psychiatry*. 2012//. 12:16	Level 1, Form Title/abstract screen
493	**Rodger Kessler**. Mental health care treatment initiation when mental health services are incorporated into primary care practice.. *Journal of the American Board of Family Medicine : JABFM*. 2012//. 25:255	Level 1, Form Title/abstract screen

494	**Karen-leigh Edward, Rhonda Nelson Hearity, Boyce Felstead**. Service integration for the dually diagnosed.. *Australian journal of primary health*. 2012//. 18:17	Level 1, Form Title/abstract screen
495	**Muhsin Michael Orsini, David L Wyrick, Jeffrey J Milroy**. Collaborative evaluation of a high school prevention curriculum: How methods of collaborative evaluation enhanced a randomized control trial to inform program improvement.. *Evaluation and program planning*. 2012//. 35:529	Level 1, Form Title/abstract screen
496	**Hein de Haan, Evelien Joosten, Toon Wijdeveld, Peter Boswinkel, Job van der Palen, Cor De Jong**. Alexithymia is not a stable personality trait in patients with substance use disorders.. *Psychiatry research*. 2012//. 198:123	Level 1, Form Title/abstract screen
497	**David L Mintz, David F Flynn**. How (not what) to prescribe: nonpharmacologic aspects of psychopharmacology.. *The Psychiatric clinics of North America*. 2012//. 35:143	Level 1, Form Title/abstract screen
498	**Katherine Newman Taylor, Suzanne Sambrook**. CBT for culture change: formulating teams to improve patient care.. *Behavioural and cognitive psychotherapy*. 2012//. 40:496	Level 1, Form Title/abstract screen
499	**David Mechanic**. Seizing opportunities under the Affordable Care Act for transforming the mental and behavioral health system.. *Health affairs (Project Hope)*. 2012//. 31:376	Level 1, Form Title/abstract screen
501	**Kim A Hoffman, Carla A Green, James H 2nd Ford, Jennifer P Wisdom, David H Gustafson, Dennis McCarty**. Improving quality of care in substance abuse treatment using five key process improvement principles.. *The journal of behavioral health services & research*. 2012//. 39:234	Level 2, Form Full Text Screening
502	**Kenneth Wilson, Angela Halsey, Helen Macpherson, Jane Billington, Sharon Hill, Gavin Johnson, Keerthy Raju, Pat Abbott**. The psycho-social rehabilitation of patients with alcohol-related brain damage in the community.. *Alcohol and alcoholism (Oxford, Oxfordshire)*. 2012//. 47:304	Level 1, Form Title/abstract screen
503	**William Robinson, Sarah Moody-Thomas, Deann Gruber**. Patient perspectives on tobacco cessation services for persons living with HIV/AIDS.. *AIDS care*. 2012//. 24:71	Level 1, Form Title/abstract screen
505	**Dail Fields, Paul M Roman, Terry C Blum**. Management systems, patient quality improvement, resource availability, and substance abuse treatment quality.. *Health services research*. 2012//. 47:1068	Level 2, Form Full Text Screening
506	**Marina A Bornovalova, Kim L Gratz, Stacey B Daughters, Elizabeth D Hunt, C W Lejuez**. Initial RCT of a distress tolerance treatment for individuals with substance use disorders.. *Drug and alcohol dependence*. 2012//. 122:70	Level 1, Form Title/abstract screen
507	**Thomas M Kelly, Dennis C Daley, Antoine B Douaihy**. Treatment of substance abusing patients with comorbid psychiatric disorders.. *Addictive behaviors*. 2012//. 37:11	Level 2, Form Full Text Screening
508	**Jennifer Broom, David Sowden, Merran Williams, Kuong Taing, Karen Morwood, Karen McGill**. Moving from viral suppression to comprehensive patient-centered care: the high prevalence of comorbid conditions and health risk factors in HIV-1-infected patients in Australia.. *Journal of the International Association of Physicians in AIDS Care (Chicago, Ill. : 2002)*. 2012//. 11:109	Level 1, Form Title/abstract screen
509	**David Misdrahi, Marion Petit, Olivier Blanc, Franck Bayle, Pierre-Michel Llorca**. The influence of therapeutic alliance and insight on medication adherence in schizophrenia.. *Nordic journal of psychiatry*. 2012//. 66:49	Level 1, Form Title/abstract screen
510	**Helen-Maria Lekas, Karolynn Siegel, Jason Leider**. Challenges facing providers caring for HIV/HCV-coinfected patients.. *Qualitative health research*.	Level 1, Form Title/abstract

	2012//. 22:54	screen
511	**Angela Patterson**. Behaviour change to treat overactive bladder syndrome.. *Nursing times*. 2011//. 107:16	Level 1, Form Title/abstract screen
512	**Len Fromer**. Implementing chronic care for COPD: planned visits, care coordination, and patient empowerment for improved outcomes.. *International journal of chronic obstructive pulmonary disease*. 2011//. 6:605	Level 1, Form Title/abstract screen
513	**Rajani S Sadasivam, Kathryn Delaughter, Katie Crenshaw, Heather J Sobko, Jessica H Williams, Heather L Coley, Midge N Ray, Daniel E Ford, Jeroan J Allison, Thomas K Houston**. Development of an interactive, Web-delivered system to increase provider-patient engagement in smoking cessation.. *Journal of medical Internet research*. 2011//. 13:e87	Level 1, Form Title/abstract screen
514	**Maria C Raven, Kelly M Doran, Shannon Kostrowski, Colleen C Gillespie, Brian D Elbel**. An intervention to improve care and reduce costs for high-risk patients with frequent hospital admissions: a pilot study.. *BMC health services research*. 2011//. 11:270	Level 1, Form Title/abstract screen
515	**Rani Marx, Michael J Drennan, Elizabeth C Johnson, Anne M Hirozawa, Winnie M Tse, Mitchell H Katz**. Assessing and increasing patient panel size in the public sector.. *Journal of public health management and practice : JPHMP*. 2011//. 17:506	Level 1, Form Title/abstract screen
516	**Gordon O Matheson, Martin Klugl, Jiri Dvorak, Lars Engebretsen, Willem H Meeuwisse, Martin Schwellnus, Steven N Blair, Willem van Mechelen, Wayne Derman, Mats Borjesson, Fredrik Bendiksen, Richard Weiler**. Responsibility of sport and exercise medicine in preventing and managing chronic disease: applying our knowledge and skill is overdue.. *British journal of sports medicine*. 2011//. 45:1272	Level 1, Form Title/abstract screen
517	**Allen Smart, Kate B Reynolds, Susan Yaggy**. Integrating substance abuse treatment into the medical home.. *North Carolina medical journal*. 2011//. 72:245	Level 1, Form Title/abstract screen
518	**Linda Rosenberg**. Addressing trauma in mental health and substance use treatment.. *The journal of behavioral health services & research*. 2011//. 38:428	Level 1, Form Title/abstract screen
519	**Trijntje Y G van der Voort, Berno van Meijel, Peter J J Goossens, Janwillem Renes, Aartjan T F Beekman, Ralph W Kupka**. Collaborative care for patients with bipolar disorder: a randomised controlled trial.. *BMC psychiatry*. 2011//. 11:133	Level 1, Form Title/abstract screen
520	**Martin Weegmann, Edward J Khantzian**. Envelopments: immersion in and emergence from drug misuse.. *American journal of psychotherapy*. 2011//. 65:163	Level 1, Form Title/abstract screen
521	**Catherine J Segan, Ron Borland, Kay A Wilhelm, Sunil S Bhar, Ainslie T Hannan, David R Dunt, Ian T Ferretter**. Helping smokers with depression to quit smoking: collaborative care with Quitline.. *The Medical journal of Australia*. 2011//. 195:S7	Level 1, Form Title/abstract screen
522	**Frances J Kay-Lambkin, Amanda L Baker, Brian Kelly, Terry J Lewin**. Clinician-assisted computerised versus therapist-delivered treatment for depressive and addictive disorders: a randomised controlled trial.. *The Medical journal of Australia*. 2011//. 195:S44	Level 2, Form Full Text Screening
523	**Nancy M Petry, Jeremiah Weinstock, Sheila M Alessi**. A randomized trial of contingency management delivered in the context of group counseling.. *Journal*	Level 1, Form Title/abstract

	of consulting and clinical psychology. 2011//. 79:686	screen
524	**Gabriela Novotna, Karen A Urbanoski, Brian R Rush**. Client-centered design of residential addiction and mental health care facilities: staff perceptions of their work environment.. *Qualitative health research*. 2011//. 21:1527	Level 2, Form Full Text Screening
525	**Alison B Hamilton, Ines Poza, Donna L Washington**. "Homelessness and trauma go hand-in-hand": pathways to homelessness among women veterans.. *Women's health issues : official publication of the Jacobs Institute of Women's Health*. 2011//. 21:S203	Level 1, Form Title/abstract screen
526	**Beth Thomas Hertz**. Combining quantity & quality. Adding new revenue streams can enable your practice to offer higher levels of service.. *Medical economics*. 2011//. 88:24	Level 1, Form Title/abstract screen
527	**Anna K McDowell, Timothy W Lineberry, J Michael Bostwick**. Practical suicide-risk management for the busy primary care physician.. *Mayo Clinic proceedings*. 2011//. 86:792	Level 1, Form Title/abstract screen
528	**Lynn E Sullivan, Jeanette M Tetrault, R Scott Braithwaite, Barbara J Turner, David A Fiellin**. A meta-analysis of the efficacy of nonphysician brief interventions for unhealthy alcohol use: implications for the patient-centered medical home.. *The American journal on addictions*. 2011//. 20:343	Level 1, Form Title/abstract screen
529	**William C Wadland, Vincent J WinklerPrins, Mary M Noel, Margaret E Thompson, Carlos F Rios-Bedoya**. Student performance on smoking cessation counseling with standardized patients.. *Family medicine*. 2011//. 43:422	Level 1, Form Title/abstract screen
530	**Louise Stone**. Explaining the unexplainable - crafting explanatory frameworks for medically unexplained symptoms.. *Australian family physician*. 2011//. 40:440	Level 1, Form Title/abstract screen
531	**Lawrence Ward, Nima M Patel, Alexandra Hanlon, Shaden Eldakar-Hein, Kristin Sherlinski, Stephanie H Ward**. Prescription medication borrowing among adult patients at an urban medical center.. *Journal of urban health : bulletin of the New York Academy of Medicine*. 2011//. 88:997	Level 1, Form Title/abstract screen
532	**Anirban Basu**. Economics of individualization in comparative effectiveness research and a basis for a patient-centered health care.. *Journal of health economics*. 2011//. 30:549	Level 1, Form Title/abstract screen
533	**Geir Smedslund, Rigmor C Berg, Karianne T Hammerstrom, Asbjorn Steiro, Kari A Leiknes, Helene M Dahl, Kjetil Karlsen**. Motivational interviewing for substance abuse.. *The Cochrane database of systematic reviews*. 2011//. #volume#:CD008063	Level 1, Form Title/abstract screen
535	**Robert Hoffmann, Virna Little**. Trans-disciplinary care: a new approach to improving the effectiveness of tobacco use interventions.. *Journal of health care for the poor and underserved*. 2011//. 22:409	Level 1, Form Title/abstract screen
536	**Klea D Bertakis, Rahman Azari**. Patient-centered care is associated with decreased health care utilization.. *Journal of the American Board of Family Medicine : JABFM*. 2011//. 24:229	Level 1, Form Title/abstract screen
537	**Christina Nicolaidis**. Police officer, deal-maker, or health care provider? Moving to a patient-centered framework for chronic opioid management.. *Pain medicine (Malden, Mass.)*. 2011//. 12:890	Level 1, Form Title/abstract screen
538	**Courtney Valdez, Rachel Kimerling, Jenny K Hyun, Hanna F Mark, Meghan Saweikis, Joanne Pavao**. Veterans Health Administration mental health treatment settings of patients who report military sexual trauma.. *Journal of trauma & dissociation : the official journal of the International Society for the Study of Dissociation (ISSD)*. 2011//. 12:232	Level 1, Form Title/abstract screen

540	**Rogerio M Pinto, Aimee N C Campbell, Denise A Hien, Gary Yu, Prakash Gorroochurn**. Retention in the National Institute on Drug Abuse Clinical Trials Network Women and Trauma Study: implications for posttrial implementation.. *The American journal of orthopsychiatry*. 2011//. 81:211	Level 2, Form Full Text Screening
541	**Benjamin J Powers, Susanne Danus, Janet M Grubber, Maren K Olsen, Eugene Z Oddone, Hayden B Bosworth**. The effectiveness of personalized coronary heart disease and stroke risk communication.. *American heart journal*. 2011//. 161:673	Level 1, Form Title/abstract screen
542	**Manuela Garcia de la Hera, Maria Carmen Davo, Rosa Ballester-Anon, Jesus Vioque**. The opinions of injecting drug user (IDUs) HIV patients and health professionals on access to antiretroviral treatment and health services in Valencia, Spain.. *Evaluation & the health professions*. 2011//. 34:349	Level 1, Form Title/abstract screen
543	**Daniel P Alford, Colleen T LaBelle, Natalie Kretsch, Alexis Bergeron, Michael Winter, Michael Botticelli, Jeffrey H Samet**. Collaborative care of opioid-addicted patients in primary care using buprenorphine: five-year experience.. *Archives of internal medicine*. 2011//. 171:425	Level 1, Form Title/abstract screen
544	**Stephen R Shirk, Marc S Karver, Renee Brown**. The alliance in child and adolescent psychotherapy.. *Psychotherapy (Chicago, Ill.)*. 2011//. 48:17	Level 1, Form Title/abstract screen
545	**Amy S B Bohnert, Kara Zivin, Deborah E Welsh, Amy M Kilbourne**. Ratings of patient-provider communication among veterans: serious mental illnesses, substance use disorders, and the moderating role of trust.. *Health communication*. 2011//. 26:267	Level 1, Form Title/abstract screen
546	**Laura S Lorenz, Jon A Chilingerian**. Using visual and narrative methods to achieve fair process in clinical care.. *Journal of visualized experiments : JoVE*. 2011//. #volume#:#pages#	Level 1, Form Title/abstract screen
548	**Deborah S Finnell, Jongwon Lee**. Psychometric properties of the decisional balance for patient choice in substance abuse treatment.. *Issues in mental health nursing*. 2011//. 32:243	Level 1, Form Title/abstract screen
549	**David B Buller, Walter F Young, Erwin P Bettinghaus, Ron Borland, Joseph B Walther, Donald Helme, Peter A Andersen, Gary R Cutter, Julie A Maloy**. Continued benefits of a technical assistance web site to local tobacco control coalitions during a state budget shortfall.. *Journal of public health management and practice : JPHMP*. 2011//. 17:E10	Level 1, Form Title/abstract screen
550	**Billie Bonevski, Christine Paul, Catherine D'Este, Robert Sanson-Fisher, Robert West, Afaf Girgis, Mohammad Siahpush, Robert Carter**. RCT of a client-centred, caseworker-delivered smoking cessation intervention for a socially disadvantaged population.. *BMC public health*. 2011//. 11:70	Level 1, Form Title/abstract screen
551	**Frances Kay-Lambkin, Amanda Baker, Terry Lewin, Vaughan Carr**. Acceptability of a clinician-assisted computerized psychological intervention for comorbid mental health and substance use problems: treatment adherence data from a randomized controlled trial.. *Journal of medical Internet research*. 2011//. 13:e11	Level 2, Form Full Text Screening
552	**Larry F Hamm, Bonnie K Sanderson, Philip A Ades, Kathy Berra, Leonard A Kaminsky, Jeffrey L Roitman, Mark A Williams**. Core competencies for cardiac rehabilitation/secondary prevention professionals: 2010 update: position statement of the American Association of Cardiovascular and Pulmonary Rehabilitation.. *Journal of cardiopulmonary rehabilitation and prevention*. 2011//. 31:2	Level 1, Form Title/abstract screen
553	**Brenda Roman, Nicole Borges, Ann K Morrison**. Teaching motivational	Level 1, Form

	interviewing skills to third-year psychiatry clerkship students.. *Academic psychiatry : the journal of the American Association of Directors of Psychiatric Residency Training and the Association for Academic Psychiatry*. 2011//. 35:51	Title/abstract screen
554	**Susan V Eisen, Kathryn A Bottonari, Mark E Glickman, Avron 3rd Spiro, Mark R Schultz, Lawrence Herz, Robert Rosenheck, Ethan S Rofman**. The incremental value of self-reported mental health measures in predicting functional outcomes of veterans.. *The journal of behavioral health services & research*. 2011//. 38:170	Level 1, Form Title/abstract screen
555	**Theresa W Kim, Richard Saitz, Debbie M Cheng, Michael R Winter, Julie Witas, Jeffrey H Samet**. Initiation and engagement in chronic disease management care for substance dependence.. *Drug and alcohol dependence*. 2011//. 115:80	Level 1, Form Title/abstract screen
556	**Benjamin J Morasco, Kathryn Corson, Dennis C Turk, Steven K Dobscha**. Association between substance use disorder status and pain-related function following 12 months of treatment in primary care patients with musculoskeletal pain.. *The journal of pain : official journal of the American Pain Society*. 2011//. 12:352	Level 1, Form Title/abstract screen
557	**Klea D Bertakis, Rahman Azari**. Determinants and outcomes of patient-centered care.. *Patient education and counseling*. 2011//. 85:46	Level 1, Form Title/abstract screen
558	**P Todd Korthuis, Somnath Saha, Geetanjali Chander, Dennis McCarty, Richard D Moore, Jonathan A Cohn, Victoria L Sharp, Mary Catherine Beach**. Substance use and the quality of patient-provider communication in HIV clinics.. *AIDS and behavior*. 2011//. 15:832	Level 1, Form Title/abstract screen
559	**Robert E Booth, Barbara K Campbell, Susan K Mikulich-Gilbertson, Carrie J Tillotson, Dongseok Choi, James Robinson, Donald A Calsyn, Raul N Mandler, Lindsay M Jenkins, Laetitia L Thompson, Catherine L Dempsey, Michael R Liepman, Dennis McCarty**. Reducing HIV-related risk behaviors among injection drug users in residential detoxification.. *AIDS and behavior*. 2011//. 15:30	Level 1, Form Title/abstract screen
560	**John E Zeber, Alexander L Miller, Laurel A Copeland, John F McCarthy, Kara Zivin, Marcia Valenstein, Devra Greenwald, Amy M Kilbourne**. Medication adherence, ethnicity, and the influence of multiple psychosocial and financial barriers.. *Administration and policy in mental health*. 2011//. 38:86	Level 1, Form Title/abstract screen
562	**Nora D Volkow**. Toward individualized treatment for substance abuse.. *Addiction science & clinical practice*. 2010//. 5:2	Level 1, Form Title/abstract screen
563	**Frank Verloin DeGruy, Rebecca S Etz**. Attending to the whole person in the patient-centered medical home: the case for incorporating mental healthcare, substance abuse care, and health behavior change.. *Families, systems & health : the journal of collaborative family healthcare*. 2010//. 28:298	Level 1, Form Title/abstract screen
564	**Gregory A Goldman, Robert J Gregory**. Relationships between techniques and outcomes for borderline personality disorder.. *American journal of psychotherapy*. 2010//. 64:359	Level 1, Form Title/abstract screen
565	**Christauria Welland, Neil Ribner**. Culturally specific treatment for partner-abusive Latino men: a qualitative study to identify and implement program components.. *Violence and victims*. 2010//. 25:799	Level 1, Form Title/abstract screen
566	**Francoise Ninane, Jean-Bernard Daeppen, Olivier Bugnon, Alain Pecoud**. [Integrated care: how to support a culture change?].. *Revue medicale suisse*. 2010//. 6:2302	Level 1, Form Title/abstract screen

567	**Erica S N Tan, Mark A Yarhouse**. Facilitating congruence between religious beliefs and sexual identity with mindfulness.. *Psychotherapy (Chicago, Ill.)*. 2010//. 47:500	Level 1, Form Title/abstract screen
568	**Bernadette Lange**. The yoga mat as common ground: policy from a holistic perspective.. *Beginnings (American Holistic Nurses' Association)*. 2010//. 30:22	Level 1, Form Title/abstract screen
569	**Fadia T Shaya, Xia Yan, Maryam Farshid, Samer Barakat, Miah Jung, Sara Low, Donald Fedder**. Social networks in cardiovascular disease management.. *Expert review of pharmacoeconomics & outcomes research*. 2010//. 10:701	Level 1, Form Title/abstract screen
570	**Amy M Kilbourne, Carrie Farmer Teh, Deborah Welsh, Harold Alan Pincus, Elaine Lasky, Brian Perron, Mark S Bauer**. Implementing composite quality metrics for bipolar disorder: towards a more comprehensive approach to quality measurement.. *General hospital psychiatry*. 2010//. 32:636	Level 1, Form Title/abstract screen
571	**Diane E Boyer, Catherine Kane**. Program evaluation of a community crisis stabilization program.. *Archives of psychiatric nursing*. 2010//. 24:387	Level 1, Form Title/abstract screen
572	**Seddon Savage**. The patient-centered opioid treatment agreement.. *The American journal of bioethics : AJOB*. 2010//. 10:18	Level 1, Form Title/abstract screen
573	**Benjamin G Druss, Barbara J Mauer**. Health care reform and care at the behavioral health--primary care interface.. *Psychiatric services (Washington, D.C.)*. 2010//. 61:1087	Level 1, Form Title/abstract screen
574	**Sara B McMenamin, Nicole M Bellows, Helen A Halpin, Diane R Rittenhouse, Lawrence P Casalino, Stephen M Shortell**. Adoption of policies to treat tobacco dependence in U.S. medical groups.. *American journal of preventive medicine*. 2010//. 39:449	Level 1, Form Title/abstract screen
575	**Astrid Birgden, Luke Grant**. Establishing a compulsory drug treatment prison: Therapeutic policy, principles, and practices in addressing offender rights and rehabilitation.. *International journal of law and psychiatry*. 2010//. 33:341	Level 1, Form Title/abstract screen
576	**Len Fromer, Thomas Barnes, Chris Garvey, Gabriel Ortiz, Dennis F Saver, Barbara Yawn**. Innovations to achieve excellence in COPD diagnosis and treatment in primary care.. *Postgraduate medicine*. 2010//. 122:150	Level 1, Form Title/abstract screen
577	**Molly Magill, Nadine R Mastroleo, Timothy R Apodaca, Nancy P Barnett, Suzanne M Colby, Peter M Monti**. Motivational interviewing with significant other participation: assessing therapeutic alliance and patient satisfaction and engagement.. *Journal of substance abuse treatment*. 2010//. 39:391	Level 1, Form Title/abstract screen
578	**Elizabeth Loder**. The approach to the difficult patient.. *Handbook of clinical neurology*. 2010//. 97:233	Level 1, Form Title/abstract screen
579	**Darryl L Bassett**. Risk assessment and management in bipolar disorders.. *The Medical journal of Australia*. 2010//. 193:S21	Level 1, Form Title/abstract screen
580	**Kara Zivin, Paul N Pfeiffer, Benjamin R Szymanski, Marcia Valenstein, Edward P Post, Erin M Miller, John F McCarthy**. Initiation of Primary Care-Mental Health Integration programs in the VA Health System: associations with psychiatric diagnoses in primary care.. *Medical care*. 2010//. 48:843	Level 1, Form Title/abstract screen
581	**James Tew, Johanna Klaus, David W Oslin**. The Behavioral Health	Level 1, Form

	Laboratory: building a stronger foundation for the patient-centered medical home.. *Families, systems & health : the journal of collaborative family healthcare*. 2010//. 28:130	Title/abstract screen
582	**Brian P Kaskie, Kathleen C Buckwalter**. The collaborative model of mental health care for older Iowans.. *Research in gerontological nursing*. 2010//. 3:200	Level 1, Form Title/abstract screen
583	**Eleanor T Lewis, Ann Combs, Jodie A Trafton**. Reasons for under-use of prescribed opioid medications by patients in pain.. *Pain medicine (Malden, Mass.)*. 2010//. 11:861	Level 1, Form Title/abstract screen
584	**Steven B Andrews, Tyler Drake, William Haslett, Rajesh Munusamy**. Developing web-based online support tools: the Dartmouth decision support software.. *Psychiatric rehabilitation journal*. 2010//. 34:37	Level 1, Form Title/abstract screen
585	**Samadhi Deva Campbell, Simon Justin Adamson, Janet Deborah Carter**. Client language during motivational enhancement therapy and alcohol use outcome.. *Behavioural and cognitive psychotherapy*. 2010//. 38:399	Level 1, Form Title/abstract screen
586	**Douglas L Polcin, Gantt P Galloway, Jason Bond, Rachael Korcha, Thomas K Greenfield**. How do residents of recovery houses experience confrontation between entry and 12-month follow-up?.. *Journal of psychoactive drugs*. 2010//. 42:49	Level 1, Form Title/abstract screen
587	**C Wallace**. Integrated assessment of older adults who misuse alcohol.. *Nursing standard (Royal College of Nursing (Great Britain) : 1987)*. 2010//. 24:51	Level 1, Form Title/abstract screen
588	**Christine Barrowclough, Petra Meier, Ruth Beardmore, Richard Emsley**. Predicting therapeutic alliance in clients with psychosis and substance misuse.. *The Journal of nervous and mental disease*. 2010//. 198:373	Level 1, Form Title/abstract screen
589	**H M Seidling, S P W Schmitt, T Bruckner, J Kaltschmidt, M G Pruszydlo, C Senger, T Bertsche, I Walter-Sack, W E Haefeli**. Patient-specific electronic decision support reduces prescription of excessive doses.. *Quality & safety in health care*. 2010//. 19:e15	Level 1, Form Title/abstract screen
590	**Sonja Pasche, Bronwyn Myers, Mohamed Adam**. Factors associated with retention in alcohol and other drug treatment among disadvantaged communities in Cape Town, South Africa.. *Journal of studies on alcohol and drugs*. 2010//. 71:395	Level 1, Form Title/abstract screen
591	**Megan Petrie, Douglas Zatzick**. Collaborative care interventions in general trauma patients.. *Oral and maxillofacial surgery clinics of North America*. 2010//. 22:261	Level 1, Form Title/abstract screen
592	**Eunice C Wong, Grant N Marshall**. Barriers to the collaborative care of patients with orofacial injury.. *Oral and maxillofacial surgery clinics of North America*. 2010//. 22:247	Level 1, Form Title/abstract screen
593	**Vivek Shetty, Grant N Marshall**. Preface: collaborative care of the facial injury patient.. *Oral and maxillofacial surgery clinics of North America*. 2010//. 22:ix	Level 1, Form Title/abstract screen
594	**Robert A Rosenheck, Michael S Neale, Somaia Mohamed**. Transition to low intensity case management in a VA Assertive Community Treatment model program.. *Psychiatric rehabilitation journal*. 2010//. 33:288	Level 1, Form Title/abstract screen
595	**Leanne Finney**. Nursing care for the patient with co-existing pain and substance misuse: meeting the patient's needs.. *Medsurg nursing : official journal of the Academy of Medical-Surgical Nurses*. 2010//. 19:25	Level 2, Form Full Text Screening

596	**Laura M Garnier, Amelia M Arria, Kimberly M Caldeira, Kathryn B Vincent, Kevin E O'Grady, Eric D Wish**. Sharing and selling of prescription medications in a college student sample.. *The Journal of clinical psychiatry*. 2010//. 71:262	Level 1, Form Title/abstract screen
597	**Kathleen R Delaney**. Recovery paradigm: confession of the unenlightened.. *Archives of psychiatric nursing*. 2010//. 24:137	Level 1, Form Title/abstract screen
598	**Bronwyn J Myers, Sonja Pasche, Mohamed Adam**. Correlates of substance abuse treatment completion among disadvantaged communities in Cape Town, South Africa.. *Substance abuse treatment, prevention, and policy*. 2010//. 5:3	Level 1, Form Title/abstract screen
599	**Vivek Shetty, Larissa J Mooney, Corwin M Zigler, Thomas R Belin, Debra Murphy, Richard Rawson**. The relationship between methamphetamine use and increased dental disease.. *Journal of the American Dental Association (1939)*. 2010//. 141:307	Level 1, Form Title/abstract screen
600	**Kent E Hutchison**. Substance use disorders: realizing the promise of pharmacogenomics and personalized medicine.. *Annual review of clinical psychology*. 2010//. 6:577	Level 1, Form Title/abstract screen
601	**Erdmann Fahndrich, Ingrid Munk**. [Absconding of a patient from an acute psychiatric ward. Whom do the courts hold liable?].. *Psychiatrische Praxis*. 2010//. 37:89	Level 1, Form Title/abstract screen
602	**Susan Swartz Woods, Carlos Roberto Jaen**. Increasing consumer demand for tobacco treatments: Ten design recommendations for clinicians and healthcare systems.. *American journal of preventive medicine*. 2010//. 38:S385	Level 2, Form Full Text Screening
603	**William Fals-Stewart, Wendy K K Lam**. Computer-assisted cognitive rehabilitation for the treatment of patients with substance use disorders: a randomized clinical trial.. *Experimental and clinical psychopharmacology*. 2010//. 18:87	Level 1, Form Title/abstract screen
604	**Nancy M Petry, Sheila M Alessi, David M Ledgerwood, Sean Sierra**. Psychometric properties of the contingency management competence scale.. *Drug and alcohol dependence*. 2010//. 109:167	Level 1, Form Title/abstract screen
605	**Steven D Vannoy, Patricia Arean, Jurgen Unutzer**. Advantages of using estimated depression-free days for evaluating treatment efficacy.. *Psychiatric services (Washington, D.C.)*. 2010//. 61:160	Level 1, Form Title/abstract screen
606	**Paul Crits-Christoph, Sarah Ring-Kurtz, Bridget McClure, Christina Temes, Agatha Kulaga, Robert Gallop, Robert Forman, John Rotrosen**. A randomized controlled study of a web-based performance improvement system for substance abuse treatment providers.. *Journal of substance abuse treatment*. 2010//. 38:251	Level 1, Form Title/abstract screen
607	**T Nordfjaern, T Rundmo, R Hole**. Treatment and recovery as perceived by patients with substance addiction.. *Journal of psychiatric and mental health nursing*. 2010//. 17:46	Level 2, Form Full Text Screening
608	**S Lampen-Imkamp, U Blanke, W Dillo**. ["Help Conference", a new social assistance for integration according to 53 SGB XII--a two-year analysis].. *Gesundheitswesen (Bundesverband der Arzte des Offentlichen Gesundheitsdienstes (Germany))*. 2010//. 72:763	Level 1, Form Title/abstract screen
609	**Doug Sellman**. The 10 most important things known about addiction.. *Addiction (Abingdon, England)*. 2010//. 105:6	Level 1, Form Title/abstract screen
610	**Miguel E Gallardo, Shannon J Curry**. Shifting perspectives: culturally	Level 2, Form

	responsive interventions with latino substance abusers.. *Journal of ethnicity in substance abuse*. 2009//. 8:314	Full Text Screening
611	**William B Jaffee, Genie L Bailey, Michelle Lohman, Paula Riggs, Leah McDonald, Roger D Weiss**. Methods of recruiting adolescents with psychiatric and substance use disorders for a clinical trial.. *The American journal of drug and alcohol abuse*. 2009//. 35:381	Level 1, Form Title/abstract screen
612	**Rebekka S Palmer, Mary K Murphy, Alessandro Piselli, Samuel A Ball**. Substance user treatment dropout from client and clinician perspectives: a pilot study.. *Substance use & misuse*. 2009//. 44:1021	Level 1, Form Title/abstract screen
613	**Brian E Perron, Matthew O Howard, Jenna K Nienhuis, Mark S Bauer, Amanda Toler Woodward, Amy M Kilbourne**. Prevalence and burden of general medical conditions among adults with bipolar I disorder: results from the National Epidemiologic Survey on Alcohol and Related Conditions.. *The Journal of clinical psychiatry*. 2009//. 70:1407	Level 1, Form Title/abstract screen
614	**Vani Nath Simmons, Erika B Litvin, Riddhi D Patel, Paul B Jacobsen, Judith C McCaffrey, Gerold Bepler, Gwendolyn P Quinn, Thomas H Brandon**. Patient-provider communication and perspectives on smoking cessation and relapse in the oncology setting.. *Patient education and counseling*. 2009//. 77:398	Level 1, Form Title/abstract screen
615	**Douglas Gourlay, Howard A Heit**. Commentary.. *Clinical chemistry*. 2009//. 55:1769	Level 1, Form Title/abstract screen
616	**Richard M Bergenstal, Nick Freemantle, Malgorzata Leyk, Gordon B Jr Cutler, Risa P Hayes, Douglas B Muchmore**. Does availability of AIR insulin increase insulin use and improve glycemic control in patients with type 2 diabetes?.. *Diabetes technology & therapeutics*. 2009//. 11 Suppl 2:S45	Level 1, Form Title/abstract screen
617	**Roger Nuttall**. Holistic care of a drug-related wound: a case study from a clinic for homeless people.. *Nursing times*. 2009//. 105:18	Level 1, Form Title/abstract screen
618	**Joseph P Gone**. A community-based treatment for Native American historical trauma: prospects for evidence-based practice.. *Journal of consulting and clinical psychology*. 2009//. 77:751	Level 1, Form Title/abstract screen
619	**Carolyn Coulson, Felicity Ng, Marjan Geertsema, Seetal Dodd, Michael Berk**. Client-reported reasons for non-engagement in drug and alcohol treatment.. *Drug and alcohol review*. 2009//. 28:372	Level 1, Form Title/abstract screen
621	**Christine M J Snyder, Stephen A Anderson**. An examination of mandated versus voluntary referral as a determinant of clinical outcome.. *Journal of marital and family therapy*. 2009//. 35:278	Level 1, Form Title/abstract screen
623	**Carina Marquez, Samuel J Mitchell, C Bradley Hare, Malcolm John, Jeffrey D Klausner**. Methamphetamine use, sexual activity, patient-provider communication, and medication adherence among HIV-infected patients in care, San Francisco 2004-2006.. *AIDS care*. 2009//. 21:575	Level 1, Form Title/abstract screen
624	**Timothy R Apodaca, Richard Longabaugh**. Mechanisms of change in motivational interviewing: a review and preliminary evaluation of the evidence.. *Addiction (Abingdon, England)*. 2009//. 104:705	Level 1, Form Title/abstract screen
625	**Peter G Miller, William R Miller**. What should we be aiming for in the treatment of addiction?.. *Addiction (Abingdon, England)*. 2009//. 104:685	Level 1, Form Title/abstract screen
627	**Pierre Thomas, Koksal Alptekin, Mihai Gheorghe, Mauro Mauri, Jose**	Level 1, Form

	**Manuel Olivares, Michael Riedel**. Management of patients presenting with acute psychotic episodes of schizophrenia.. *CNS drugs*. 2009//. 23:193	Title/abstract screen
628	**Kristin Ross**. Clients best served through collaborative practice.. *Nursing BC*. 2009//. 41:6	Level 1, Form Title/abstract screen
629	**James J Clark**. Contemporary psychotherapy research: implications for substance misuse treatment and research.. *Substance use & misuse*. 2009//. 44:42	Level 1, Form Title/abstract screen
630	**Douglas C Smith, James A Hall, Mijin Jang, Stephan Arndt**. Therapist adherence to a motivational-interviewing intervention improves treatment entry for substance-misusing adolescents with low problem perception.. *Journal of studies on alcohol and drugs*. 2009//. 70:101	Level 1, Form Title/abstract screen
631	**Anonymous**. From the editors.. *Cambridge quarterly of healthcare ethics : CQ : the international journal of healthcare ethics committees*. 2009//. 18:4	Level 1, Form Title/abstract screen
632	**Carol T Mowbray, Amanda Toler Woodward, Mark C Holter, Peter MacFarlane, Deborah Bybee**. Characteristics of users of consumer-run drop-in centers versus clubhouses.. *The journal of behavioral health services & research*. 2009//. 36:361	Level 1, Form Title/abstract screen
633	**Dennis McCarty, David Gustafson, Victor A Capoccia, Frances Cotter**. Improving care for the treatment of alcohol and drug disorders.. *The journal of behavioral health services & research*. 2009//. 36:52	Level 2, Form Full Text Screening
634	**Anita Kihlstrom, Ewa Wikstrom**. Towards network and citizen: collaborative care for drug abusers.. *The International journal of health planning and management*. 2009//. 24:233	Level 1, Form Title/abstract screen
635	**Shailesh Kumar, Darren Malone**. Panic disorder.. *BMJ clinical evidence*. 2008//. 2008:#pages#	Level 1, Form Title/abstract screen
636	**Stephanie S Covington, Cynthia Burke, Sandy Keaton, Candice Norcott**. Evaluation of a trauma-informed and gender-responsive intervention for women in drug treatment.. *Journal of psychoactive drugs*. 2008//. Suppl 5:387	Level 2, Form Full Text Screening
638	**Clive G Long, Barbara Fulton, Clive R Hollin**. The development of a 'best practice' service for women in a medium-secure psychiatric setting: treatment components and evaluation.. *Clinical psychology & psychotherapy*. 2008//. 15:304	Level 1, Form Title/abstract screen
639	**Linda M Chatters, Kai McKeever Bullard, Robert Joseph Taylor, Amanda Toler Woodward, Harold W Neighbors, James S Jackson**. Religious participation and DSM-IV disorders among older African Americans: findings from the National Survey of American Life.. *The American journal of geriatric psychiatry : official journal of the American Association for Geriatric Psychiatry*. 2008//. 16:957	Level 1, Form Title/abstract screen
640	**J Christopher Perry, J Christopher Fowler, Ann Greif Howe**. Subject and interviewer determinants of the adequacy of the dynamic interview.. *The Journal of nervous and mental disease*. 2008//. 196:612	Level 1, Form Title/abstract screen
641	**P Preston Reynolds**. Title VII innovations in American medical and dental education: responding to 21st century priorities for the health of the American public.. *Academic medicine : journal of the Association of American Medical Colleges*. 2008//. 83:1015	Level 1, Form Title/abstract screen
642	**Karni Shelef, Gary M Diamond**. Short form of the revised Vanderbilt	Level 1, Form

	therapeutic alliance scale: development, reliability, and validity.. *Psychotherapy research : journal of the Society for Psychotherapy Research*. 2008//. 18:433	Title/abstract screen
643	**Carole Fernandez, Denise Wilson**. Maori women's views on smoking cessation initiatives.. *Nursing praxis in New Zealand inc*. 2008//. 24:27	Level 1, Form Title/abstract screen
644	**Stephen D Cassivi, Mark S Allen, Gregg D Vanderwaerdt, Lori L Ewoldt, Mary E Cordes, Dennis A Wigle, Francis C Nichols, Peter C Pairolero, Claude Deschamps**. Patient-centered quality indicators for pulmonary resection.. *The Annals of thoracic surgery*. 2008//. 86:927	Level 1, Form Title/abstract screen
645	**Nicholas Steel, Max Bachmann, Susan Maisey, Paul Shekelle, Elizabeth Breeze, Michael Marmot, David Melzer**. Self reported receipt of care consistent with 32 quality indicators: national population survey of adults aged 50 or more in England.. *BMJ (Clinical research ed.)*. 2008//. 337:a957	Level 1, Form Title/abstract screen
646	**Amy L Marr, Tyson Pillow, Stephen Brown**. Southside medical homes network: linking emergency department patients to community care.. *Prehospital and disaster medicine*. 2008//. 23:282	Level 1, Form Title/abstract screen
647	**Hakan Odeberg, Bruce Rodriguez-Silva, Pirjo Salander, Bjorn Martensson**. Individualized continuation electroconvulsive therapy and medication as a bridge to relapse prevention after an index course of electroconvulsive therapy in severe mood disorders: a naturalistic 3-year cohort study.. *The journal of ECT*. 2008//. 24:183	Level 1, Form Title/abstract screen
648	**Aaron Hogue, Craig E Henderson, Sarah Dauber, Priscilla C Barajas, Adam Fried, Howard A Liddle**. Treatment adherence, competence, and outcome in individual and family therapy for adolescent behavior problems.. *Journal of consulting and clinical psychology*. 2008//. 76:544	Level 1, Form Title/abstract screen
649	**Judy C Chang, Diane Dado, Richard M Frankel, Keri L Rodriguez, Susan Zickmund, Bruce S Ling, Robert M Arnold**. When pregnant patients disclose substance use: missed opportunities for behavioral change counseling.. *Patient education and counseling*. 2008//. 72:394	Level 1, Form Title/abstract screen
650	**Simon J Adamson, J Douglas Sellman**. Five-year outcomes of alcohol-dependent persons treated with motivational enhancement.. *Journal of studies on alcohol and drugs*. 2008//. 69:589	Level 1, Form Title/abstract screen
651	**Holly Blake**. Innovation in practice: mobile phone technology in patient care.. *British journal of community nursing*. 2008//. 13:160	Level 1, Form Title/abstract screen
652	**Steven K Dobscha, Kathryn Corson, Ruth Q Leibowitz, Mark D Sullivan, Martha S Gerrity**. Rationale, design, and baseline findings from a randomized trial of collaborative care for chronic musculoskeletal pain in primary care.. *Pain medicine (Malden, Mass.)*. 2008//. 9:1050	Level 1, Form Title/abstract screen
653	**Sean J Tollison, Christine M Lee, Clayton Neighbors, Teryl A Neil, Nichole D Olson, Mary E Larimer**. Questions and reflections: the use of motivational interviewing microskills in a peer-led brief alcohol intervention for college students.. *Behavior therapy*. 2008//. 39:183	Level 1, Form Title/abstract screen
654	**Zoe Slote Morris, Maria Gannon**. Drug misuse treatment services in Scotland: predicting outcomes.. *International journal for quality in health care : journal of the International Society for Quality in Health Care*. 2008//. 20:271	Level 1, Form Title/abstract screen
655	**Kimberly Horn, Geri Dino, Candice Hamilton, N Noerachmanto, Jianjun Zhang**. Feasibility of a smoking cessation intervention for teens in the emergency department: reach, implementation fidelity, and acceptability..	Level 1, Form Title/abstract screen

	American journal of critical care : an official publication, American Association of Critical-Care Nurses. 2008//. 17:205	
657	**Michael L Dennis, Melissa L Ives, Michelle K White, Randolph D Muck**. The Strengthening Communities for Youth (SCY) initiative: a cluster analysis of the services received, their correlates and how they are associated with outcomes.. *Journal of psychoactive drugs*. 2008//. 40:3	Level 1, Form Title/abstract screen
658	**Robert M Bossarte, Ernest E 3rd Sullivent, Julie Sinclair, Danae Bixler, Thomas R Simon, Monica H Swahn, Kristin Wilson**. Injury, violence, and risk among participants in a mass gathering of the Rainbow Family of Living Light.. *Journal of health care for the poor and underserved*. 2008//. 19:588	Level 1, Form Title/abstract screen
659	**Jose Capece**. [Rethinking addictions: the cognitive paradigm and substance dependence disorder].. *Vertex (Buenos Aires, Argentina)*. 2008//. 19:527	Level 1, Form Title/abstract screen
660	**Michael G Rank, Iris Chaffin, Charles R Figley, Tiffany Lawrence**. The treatment of posttraumatic stress disorder in an extended care psychiatric rehabilitation program.. *Work (Reading, Mass.)*. 2008//. 30:113	Level 1, Form Title/abstract screen
661	**David Forbes, Ruth Parslow, Mark Creamer, Nicholas Allen, Tony McHugh, Mal Hopwood**. Mechanisms of anger and treatment outcome in combat veterans with posttraumatic stress disorder.. *Journal of traumatic stress*. 2008//. 21:142	Level 1, Form Title/abstract screen
663	**Teresa J Hudson, Richard R Owen, Carol R Thrush, Tracey L Armitage, Purushottam Thapa**. Guideline implementation and patient-tailoring strategies to improve medication adherence for schizophrenia.. *The Journal of clinical psychiatry*. 2008//. 69:74	Level 1, Form Title/abstract screen
664	**Jane Scullion**. Patient-focused outcomes in chronic obstructive pulmonary disease.. *Nursing standard (Royal College of Nursing (Great Britain) : 1987)*. 2008//. 22:50	Level 1, Form Title/abstract screen
665	**Richard A Grucza, Thomas R Przybeck, C Robert Cloninger**. Screening for alcohol problems: an epidemiological perspective and implications for primary care.. *Missouri medicine*. 2008//. 105:67	Level 1, Form Title/abstract screen
666	**Donna J Keyser, Jeanie Knox Houtsinger, Katherine Watkins, Harold Alan Pincus**. Applying the institute of medicine quality chasm framework to improving health care for mental and substance use conditions.. *The Psychiatric clinics of North America*. 2008//. 31:43	Level 1, Form Title/abstract screen
667	**Sharon M Flicker, Charles W Turner, Holly B Waldron, Janet L Brody, Timothy J Ozechowski**. Ethnic background, therapeutic alliance, and treatment retention in functional family therapy with adolescents who abuse substances.. *Journal of family psychology : JFP : journal of the Division of Family Psychology of the American Psychological Association (Division 43)*. 2008//. 22:167	Level 2, Form Full Text Screening
668	**Barbara Lopez, Seth J Schwartz, Guillermo Prado, Ana E Campo, Hilda Pantin**. Adolescent neurological development and its implications for adolescent substance use prevention.. *The journal of primary prevention*. 2008//. 29:5	Level 1, Form Title/abstract screen
669	**Gillian M Craig, Helen Booth, Jo Hall, Alistair Story, Andrew Hayward, Ann Goodburn, Alimuddin Zumla**. Establishing a new service role in tuberculosis care: the tuberculosis link worker.. *Journal of advanced nursing*. 2008//. 61:413	Level 1, Form Title/abstract screen
670	**Aaron Hogue, Sarah Dauber, Priscilla Chinchilla, Adam Fried, Craig**	Level 1, Form

	**Henderson, Jaime Inclan, Robert H Reiner, Howard A Liddle**. Assessing fidelity in individual and family therapy for adolescent substance abuse.. *Journal of substance abuse treatment*. 2008//. 35:137	Title/abstract screen
671	**S Keshavjee, I Y Gelmanova, A D Pasechnikov, S P Mishustin, Y G Andreev, A Yedilbayev, J J Furin, J S Mukherjee, M L Rich, E A Nardell, P E Farmer, J Y Kim, S S Shin**. Treating multidrug-resistant tuberculosis in Tomsk, Russia: developing programs that address the linkage between poverty and disease.. *Annals of the New York Academy of Sciences*. 2008//. 1136:1	Level 1, Form Title/abstract screen
673	**Daniel H Angres, Ailsa K Nielsen**. The role of the TCI-R (Temperament Character Inventory) in individualized treatment plannning in a population of addicted professionals.. *Journal of addictive diseases*. 2007//. 26 Suppl 1:51	Level 1, Form Title/abstract screen
674	**Rachel Gonzales, David C Perlman**. Response: a case for collaborative care.. *Addiction science & clinical practice*. 2007//. 4:43	Level 1, Form Title/abstract screen
675	**Jennifer M Strickland, Angela Huskey, David B Brushwood**. Pharmacist-physician collaboration in pain management practice.. *Journal of opioid management*. 2007//. 3:295	Level 1, Form Title/abstract screen
676	**Laura Weiss Roberts, Mark E Johnson, Christiane Brems, Teddy D Warner**. Ethical disparities: challenges encountered by multidisciplinary providers in fulfilling ethical standards in the care of rural and minority people.. *The Journal of rural health : official journal of the American Rural Health Association and the National Rural Health Care Association*. 2007//. 23 Suppl:89	Level 1, Form Title/abstract screen
677	**Thomas F Kresina**. Medication assisted treatment of drug abuse and dependence: global availability and utilization.. *Recent patents on anti-infective drug discovery*. 2007//. 2:79	Level 1, Form Title/abstract screen
678	**Louis-Georges Cournoyer, Serge Brochu, Michel Landry, Jacques Bergeron**. Therapeutic alliance, patient behaviour and dropout in a drug rehabilitation programme: the moderating effect of clinical subpopulations.. *Addiction (Abingdon, England)*. 2007//. 102:1960	Level 1, Form Title/abstract screen
679	**Ronald W Manderscheid**. A room in the medical home. Consumers deserve "first-floor" access to mental and substance use care in collaborative care systems.. *Behavioral healthcare*. 2007//. 27:45	Level 1, Form Title/abstract screen
680	**Betty D Morgan, Anne P Rossi**. Difficult-to-manage HIV/AIDS clients with psychiatric illness and substance abuse problems: a collaborative practice with psychiatric advanced practice nurses.. *The Journal of the Association of Nurses in AIDS Care : JANAC*. 2007//. 18:77	Level 1, Form Title/abstract screen
681	**Mayur M Desai, Robert A Rosenheck, Rani A Desai**. Prevalence and correlates of human immunodeficiency virus testing and posttest counseling among outpatients with serious mental illness.. *The Journal of nervous and mental disease*. 2007//. 195:776	Level 1, Form Title/abstract screen
682	**Maureen Fitzgerald, Jay Ford**. Helping states and providers work together.. *Behavioral healthcare*. 2007//. 27:14	Level 1, Form Title/abstract screen
683	**Ian B Hickie, Andrea S Fogarty, Tracey A Davenport, Georgina M Luscombe, Jane Burns**. Responding to experiences of young people with common mental health problems attending Australian general practice.. *The Medical journal of Australia*. 2007//. 187:S47	Level 1, Form Title/abstract screen
684	**Greer Sullivan, Michelle G Craske, Cathy Sherbourne, Mark J Edlund,**	Level 1, Form

	**Raphael D Rose, Daniela Golinelli, Denise A Chavira, Alexander Bystritsky, Murray B Stein, Peter P Roy-Byrne**. Design of the Coordinated Anxiety Learning and Management (CALM) study: innovations in collaborative care for anxiety disorders.. *General hospital psychiatry*. 2007//. 29:379	Title/abstract screen
685	**Mitchell P Karno**. A case study of mediators of treatment effectiveness.. *Alcoholism, clinical and experimental research*. 2007//. 31:33s	Level 1, Form Title/abstract screen
686	**Kahryn Hughes**. Migrating identities: the relational constitution of drug use and addiction.. *Sociology of health & illness*. 2007//. 29:673	Level 1, Form Title/abstract screen
687	**Meaghan Nelson, Heidi E Hamilton**. Improving in-office discussion of chronic obstructive pulmonary disease: results and recommendations from an in-office linguistic study in chronic obstructive pulmonary disease.. *The American journal of medicine*. 2007//. 120:S28	Level 1, Form Title/abstract screen
688	**Jon Morgenstern, James R McKay**. Rethinking the paradigms that inform behavioral treatment research for substance use disorders.. *Addiction (Abingdon, England)*. 2007//. 102:1377	Level 1, Form Title/abstract screen
689	**Marcia Andersen, Elaine Hockman, Geoffrey Smereck, Jannie Tinsley, Dollie Milfort, Robert Wilcox, Teresa Smith, Christopher Connelly, Latonia Adams, Richard Thomas**. Retaining women in HIV medical care.. *The Journal of the Association of Nurses in AIDS Care : JANAC*. 2007//. 18:33	Level 1, Form Title/abstract screen
690	**Samuel A Ball**. Comparing individual therapies for personality disordered opioid dependent patients.. *Journal of personality disorders*. 2007//. 21:305	Level 1, Form Title/abstract screen
691	**Robert Forman, Paul Crits-Christoph, Ovgu Kaynak, Matt Worley, Donald A Hantula, Agatha Kulaga, John Rotrosen, Melissa Chu, Robert Gallop, Jennifer Potter, Patrice Muchowski, Kirk Brower, Stephen Strobbe, Kathy Magruder, A'Delle H Chellis, Tad Clodfelter, Margaret Cawley**. A feasibility study of a web-based performance improvement system for substance abuse treatment providers.. *Journal of substance abuse treatment*. 2007//. 33:363	Level 1, Form Title/abstract screen
692	**John C Fortney, Jeffrey M Pyne, Mark J Edlund, David K Williams, Dean E Robinson, Dinesh Mittal, Kathy L Henderson**. A randomized trial of telemedicine-based collaborative care for depression.. *Journal of general internal medicine*. 2007//. 22:1086	Level 1, Form Title/abstract screen
693	**Kieran J Moriarty, Helen Platt, Sandra Crompton, Wendy Darling, Martin Blakemore, Sue Hutchinson, David Proctor, Malcolm Brown, Burt Burtun, George Lipscomb, Kadukkavil Padmakumar**. Collaborative care for alcohol-related liver disease.. *Clinical medicine (London, England)*. 2007//. 7:125	Level 1, Form Title/abstract screen
694	**John D Corrigan, Jennifer Bogner**. Interventions to promote retention in substance abuse treatment.. *Brain injury*. 2007//. 21:343	Level 2, Form Full Text Screening
695	**Norma G Bartholomew, George W Joe, Grace A Rowan-Szal, D Dwayne Simpson**. Counselor assessments of training and adoption barriers.. *Journal of substance abuse treatment*. 2007//. 33:193	Level 1, Form Title/abstract screen
696	**Bruce R Schackman, Paul A Teixeira, Ann B Beeder**. Offers of hepatitis C care do not lead to treatment.. *Journal of urban health : bulletin of the New York Academy of Medicine*. 2007//. 84:455	Level 1, Form Title/abstract screen
697	**Linda Weinreb, Joanne Nicholson, Valerie Williams, Fran Anthes**.	Level 1, Form

	Integrating behavioral health services for homeless mothers and children in primary care.. *The American journal of orthopsychiatry*. 2007//. 77:142	Title/abstract screen
698	**Susan A Murphy, L M Collins, A John Rush**. Customizing treatment to the patient: adaptive treatment strategies.. *Drug and alcohol dependence*. 2007//. 88 Suppl 2:S1	Level 1, Form Title/abstract screen
699	**Eunice C Wong, Grant N Marshall, Vivek Shetty, Annie Zhou, Howard Belzberg, Dennis-Duke R Yamashita**. Survivors of violence-related facial injury: psychiatric needs and barriers to mental health care.. *General hospital psychiatry*. 2007//. 29:117	Level 1, Form Title/abstract screen
700	**J Miles, K Sugumar, F Macrory, D G Sims, S W D'Souza**. Methadone-exposed newborn infants: outcome after alterations to a service for mothers and infants.. *Child: care, health and development*. 2007//. 33:206	Level 1, Form Title/abstract screen
701	**Robert K Brooner, Michael S Kidorf, Van L King, Kenneth B Stoller, Karin J Neufeld, Ken Kolodner**. Comparing adaptive stepped care and monetary-based voucher interventions for opioid dependence.. *Drug and alcohol dependence*. 2007//. 88 Suppl 2:S14	Level 1, Form Title/abstract screen
702	**Sandra L Tunis, Douglas E Faries, Michael D Stensland, Donald P Hay, Bruce J Kinon**. An examination of factors affecting persistence with initial antipsychotic treatment in patients with schizophrenia.. *Current medical research and opinion*. 2007//. 23:97	Level 1, Form Title/abstract screen
703	**Laurie Drabble**. Pathways to collaboration: exploring values and collaborative practice between child welfare and substance abuse treatment fields.. *Child maltreatment*. 2007//. 12:31	Level 1, Form Title/abstract screen
704	**Daniel E Rivera, Michael D Pew, Linda M Collins**. Using engineering control principles to inform the design of adaptive interventions: a conceptual introduction.. *Drug and alcohol dependence*. 2007//. 88 Suppl 2:S31	Level 1, Form Title/abstract screen
705	**Susan A Murphy, Kevin G Lynch, David Oslin, James R McKay, Tom TenHave**. Developing adaptive treatment strategies in substance abuse research.. *Drug and alcohol dependence*. 2007//. 88 Suppl 2:S24	Level 1, Form Title/abstract screen
706	**Ronda C Zakocs, Sarah Guckenburg**. What coalition factors foster community capacity? Lessons learned from the Fighting Back Initiative.. *Health education & behavior : the official publication of the Society for Public Health Education*. 2007//. 34:354	Level 1, Form Title/abstract screen
707	**Joanne Csete**. "Second on the needle": human rights of women who use drugs.. *HIV/AIDS policy & law review*. 2006//. 11:66	Level 1, Form Title/abstract screen
708	**Alexie Cintron, R Sean Morrison**. Pain and ethnicity in the United States: A systematic review.. *Journal of palliative medicine*. 2006//. 9:1454	Level 1, Form Title/abstract screen
709	**Christianne Esposito-Smythers, Anthony Spirito, Rebecca Uth, Heather LaChance**. Cognitive behavioral treatment for suicidal alcohol abusing adolescents: development and pilot testing.. *The American journal on addictions*. 2006//. 15 Suppl 1:126	Level 1, Form Title/abstract screen
711	**Thuy Boardman, Delwyn Catley, James E Grobe, Todd D Little, Jasjit S Ahluwalia**. Using motivational interviewing with smokers: do therapist behaviors relate to engagement and therapeutic alliance?.. *Journal of substance abuse treatment*. 2006//. 31:329	Level 1, Form Title/abstract screen
712	**Samantha P Clarke, Lindsay G Oades, Trevor P Crowe, Frank P Deane**. Collaborative goal technology: theory and practice.. *Psychiatric rehabilitation*	Level 1, Form Title/abstract

	journal. 2006//. 30:129	screen
713	**Barbara J Felton, Amy Barr, Gary Clark, Sam J Tsemberis**. ACT team members' responses to training in recovery-oriented practices.. *Psychiatric rehabilitation journal*. 2006//. 30:112	Level 1, Form Title/abstract screen
714	**J Cornuz, R Bize**. Motivating for cancer prevention.. *Recent results in cancer research. Fortschritte der Krebsforschung. Progres dans les recherches sur le cancer*. 2006//. 168:7	Level 1, Form Title/abstract screen
715	**Lawrence Blonde, Judith Dempster, Joanne M Gallivan, Elizabeth Warren-Boulton**. Reducing cardiovascular disease risk in patients with diabetes: a message from the National Diabetes Education Program.. *Journal of the American Academy of Nurse Practitioners*. 2006//. 18:524	Level 1, Form Title/abstract screen
716	**Rebecca de Guzman, Noelle R Leonard, Marya Viorst Gwadz, Rebecca Young, Amanda S Ritchie, Gricel Arredondo, Marion Riedel**. "I thought there was no hope for me": a behavioral intervention for urban mothers with problem drinking.. *Qualitative health research*. 2006//. 16:1252	Level 2, Form Full Text Screening
717	**Daniel Z Lieberman**. Effects of a personified guide on adherence to an online program for alcohol abusers.. *Cyberpsychology & behavior : the impact of the Internet, multimedia and virtual reality on behavior and society*. 2006//. 9:603	Level 1, Form Title/abstract screen
718	**Natalie I Vokes, Jeannine M Bailey, Karin V Rhodes**. "Should I give you my smoking lecture now or later?" Characterizing emergency physician smoking discussions and cessation counseling.. *Annals of emergency medicine*. 2006//. 48:406	Level 1, Form Title/abstract screen
719	**J Brazier, I Tumur, M Holmes, M Ferriter, G Parry, K Dent-Brown, S Paisley**. Psychological therapies including dialectical behaviour therapy for borderline personality disorder: a systematic review and preliminary economic evaluation.. *Health technology assessment (Winchester, England)*. 2006//. 10:iii	Level 1, Form Title/abstract screen
721	**Mudita Rastogi, Serena Wadhwa**. Substance abuse1 among Asian Indians in the United States: a consideration of cultural factors in etiology and treatment.. *Substance use & misuse*. 2006//. 41:1239	Level 1, Form Title/abstract screen
722	**Alexandra Fickenscher, Douglas K Novins, Spero M Manson**. Illicit peyote use among American Indian adolescents in substance abuse treatment: a preliminary investigation.. *Substance use & misuse*. 2006//. 41:1139	Level 1, Form Title/abstract screen
723	**Robin Dauterive**. Was my patient fortunate or forsaken?.. *The Journal of clinical ethics*. 2006//. 17:90	Level 1, Form Title/abstract screen
724	**Suzanne M Burke**. The case manager's view.. *The Journal of clinical ethics*. 2006//. 17:83	Level 1, Form Title/abstract screen
725	**Ann Boyer, Debbie Indyk**. Shaping garments of care: tools for maximizing adherence potential.. *Social work in health care*. 2006//. 42:151	Level 1, Form Title/abstract screen
726	**Amy Knowlton, Julia Arnsten, Lois Eldred, James Wilkinson, Marc Gourevitch, Starley Shade, Krista Dowling, David Purcell,INSPIRE Team**. Individual, interpersonal, and structural correlates of effective HAART use among urban active injection drug users.. *Journal of acquired immune deficiency syndromes (1999)*. 2006//. 41:486	Level 1, Form Title/abstract screen
727	**Sandra G Adams, Jacqueline A Pugh, Lewis E Kazis, Shuko Lee, Antonio Anzueto**. Characteristics associated with sustained abstinence from smoking among patients with COPD.. *The American journal of medicine*. 2006//.	Level 1, Form Title/abstract screen

	119:441	
728	**James D Wilkinson, Wei Zhao, Scott Santibanez, Julia Arnsten, Amy Knowlton, Cynthia A Gomez, Lisa R Metsch,INSPIRE Study Group**. Providers' HIV prevention discussions with HIV-seropositive injection drug users.. *AIDS and behavior*. 2006//. 10:699	Level 1, Form Title/abstract screen
730	**Marc D Basson, Timothy W Butler, Harish Verma**. Predicting patient nonappearance for surgery as a scheduling strategy to optimize operating room utilization in a veterans' administration hospital.. *Anesthesiology*. 2006//. 104:826	Level 1, Form Title/abstract screen
733	**Judy Graham-Garcia, Beverly George-Gay, Douglas Heater, Abigail Butts, Janie Heath**. Application of the Synergy Model with the surgical care of smokers.. *Critical care nursing clinics of North America*. 2006//. 18:29	Level 1, Form Title/abstract screen
735	**C A Binks, M Fenton, L McCarthy, T Lee, C E Adams, C Duggan**. Psychological therapies for people with borderline personality disorder.. *The Cochrane database of systematic reviews*. 2006//. #volume#:CD005652	Level 1, Form Title/abstract screen
736	**Mark P Popenhagen**. Collaborative practice. Undertreatment of pain and fears of addiction in pediatric chronic pain patients: how do we stop the problem?.. *Journal for specialists in pediatric nursing : JSPN*. 2006//. 11:61	Level 1, Form Title/abstract screen
737	**Jane L Givens, Catherine J Datto, Katy Ruckdeschel, Kathryn Knott, Cynthia Zubritsky, David W Oslin, Soumya Nyshadham, Poornima Vanguri, Frances K Barg**. Older patients' aversion to antidepressants. A qualitative study.. *Journal of general internal medicine*. 2006//. 21:146	Level 1, Form Title/abstract screen
739	**Karen Herzig, Dale Danley, Rebecca Jackson, Ruth Petersen, Linda Chamberlain, Barbara Gerbert**. Seizing the 9-month moment: addressing behavioral risks in prenatal patients.. *Patient education and counseling*. 2006//. 61:228	Level 2, Form Full Text Screening
740	**J Hamann, A Loh, J Kasper, B Neuner, C Spies, W Kissling, M Harter, C Heesen**. [Effects of a shared decision making model in psychiatric and neurologic practice].. *Der Nervenarzt*. 2006//. 77:1071	Level 1, Form Title/abstract screen
741	**Linda S Kahn, Chester H Fox, Julie Krause-Kelly, Diane E Berdine, Renee B Cadzow**. Identifying barriers and facilitating factors to improve screening mammography rates in women diagnosed with mental illness and substance use disorders.. *Women & health*. 2005//. 42:111	Level 1, Form Title/abstract screen
742	**Maxima Encinares, Gabriella Golea**. Client-centered care for individuals with dual diagnoses in the justice system.. *Journal of psychosocial nursing and mental health services*. 2005//. 43:29	Level 1, Form Title/abstract screen
743	**Mark Ilgen, Rudolf Moos**. Deterioration following alcohol-use disorder treatment in project MATCH.. *Journal of studies on alcohol*. 2005//. 66:517	Level 1, Form Title/abstract screen
744	**Cynthia M A Geppert, Sanjeev Arora**. Ethical issues in the treatment of hepatitis C.. *Clinical gastroenterology and hepatology : the official clinical practice journal of the American Gastroenterological Association*. 2005//. 3:937	Level 1, Form Title/abstract screen
745	**Annemarie Beldon, Suzanne Crozier**. Health promotion in pregnancy: the role of the midwife.. *The journal of the Royal Society for the Promotion of Health*. 2005//. 125:216	Level 1, Form Title/abstract screen
746	**Chanson D Noether, Norma Finkelstein, Nancy R VanDeMark, Andrea Savage, Beth Glover Reed, Dawn Jahn Moses**. Design strengths and issues of SAMHSA's Women, Co-occurring Disorders, and Violence Study.. *Psychiatric services (Washington, D.C.)*. 2005//. 56:1233	Level 2, Form Full Text Screening

747	**Emily Gray, Jim McCambridge, John Strang**. The effectiveness of motivational interviewing delivered by youth workers in reducing drinking, cigarette and cannabis smoking among young people: quasi-experimental pilot study.. *Alcohol and alcoholism (Oxford, Oxfordshire)*. 2005//. 40:535	Level 1, Form Title/abstract screen
748	**Duncan Mortimer, Leonie Segal**. Economic evaluation of interventions for problem drinking and alcohol dependence: cost per QALY estimates.. *Alcohol and alcoholism (Oxford, Oxfordshire)*. 2005//. 40:549	Level 1, Form Title/abstract screen
749	**Richard Christensen, Lorrie Garces**. Encouraging transdisciplinary approaches to care.. *Journal of health care for the poor and underserved*. 2005//. 16:181	Level 1, Form Title/abstract screen
750	**Victoria A Cargill, Valerie E Stone**. HIV/AIDS: a minority health issue.. *The Medical clinics of North America*. 2005//. 89:895	Level 1, Form Title/abstract screen
751	**Margaret Lynch**. Caring for the patient with delirium tremens.. *The Journal of practical nursing*. 2005//. 55:21	Level 1, Form Title/abstract screen
753	**H Westley Clark, A Kathryn Power**. Women, Co-occurring Disorders, and Violence Study: a case for trauma-informed care.. *Journal of substance abuse treatment*. 2005//. 28:145	Level 1, Form Title/abstract screen
754	**Steffanie A Strathdee, M Latka, J Campbell, P T O'Driscoll, E T Golub, F Kapadia, R A Pollini, R S Garfein, D L Thomas, H Hagan,Study to Reduce Intravenous Exposures Project**. Factors associated with interest in initiating treatment for hepatitis C Virus (HCV) infection among young HCV-infected injection drug users.. *Clinical infectious diseases : an official publication of the Infectious Diseases Society of America*. 2005//. 40 Suppl 5:S304	Level 1, Form Title/abstract screen
756	**Jeffrey T Parsons, Elana Rosof, Joseph C Punzalan, Lauren Di Maria**. Integration of motivational interviewing and cognitive behavioral therapy to improve HIV medication adherence and reduce substance use among HIV-positive men and women: results of a pilot project.. *AIDS patient care and STDs*. 2005//. 19:31	Level 1, Form Title/abstract screen
757	**Mark Sullivan, Betty Ferrell**. Ethical challenges in the management of chronic nonmalignant pain: negotiating through the cloud of doubt.. *The journal of pain : official journal of the American Pain Society*. 2005//. 6:2	Level 1, Form Title/abstract screen
758	**Scott D Miller, Barry L Duncan, Ryan Sorrell, George S Brown**. The partners for change outcome management system.. *Journal of clinical psychology*. 2005//. 61:199	Level 1, Form Title/abstract screen
759	**Kathleen M Wesa, Patricia Culliton**. Recommendations and guidelines regarding the preferred research protocol for investigating the impact of an optimal healing environment on patients with substance abuse.. *Journal of alternative and complementary medicine (New York, N.Y.)*. 2004//. 10 Suppl 1:S193	Level 1, Form Title/abstract screen
760	**Denise M Dudzinski, Mark Sullivan**. When agreeing with the patient is not enough: a schizophrenic woman requests pregnancy termination.. *General hospital psychiatry*. 2004//. 26:475	Level 1, Form Title/abstract screen
762	**Benny J Primm, Lucille Perez, Gary C Dennis, Lennette Benjamin, Westley Clark, Kathy Keough, W David Leak, Richard Payne, Deborah Smith, Louis W Sullivan,National Medical Association**. Managing pain: The Challenge in Underserved Populations: Appropriate Use Versus Abuse and Diversion.. *Journal of the National Medical Association*. 2004//. 96:1152	Level 1, Form Title/abstract screen

763	**Roberto Secades-Villa, Jose Ramon Fernande-Hermida, Cristina Arnaez-Montaraz**. Motivational interviewing and treatment retention among drug user patients: a pilot study.. *Substance use & misuse*. 2004//. 39:1369	Level 1, Form Title/abstract screen
764	**Holly A Sindelar, Ana M Abrantes, Chantelle Hart, William Lewander, Anthony Spirito**. Motivational interviewing in pediatric practice.. *Current problems in pediatric and adolescent health care*. 2004//. 34:322	Level 1, Form Title/abstract screen
765	**Roxanne Struthers, Felicia S Hodge**. Sacred tobacco use in Ojibwe communities.. *Journal of holistic nursing : official journal of the American Holistic Nurses' Association*. 2004//. 22:209	Level 1, Form Title/abstract screen
767	**John de Miranda**. Coming to the addiction community: consumer--directed care.. *Behavioral healthcare tomorrow*. 2004//. 13:25	Level 1, Form Title/abstract screen
768	**D A Murphy, W D Marelich, D Hoffman, W N Steers**. Predictors of antiretroviral adherence.. *AIDS care*. 2004//. 16:471	Level 1, Form Title/abstract screen
769	**Ching-Yen Chen, Ying-Jen Chen, Yeong-Yuh Juang, Chia-Yih Liu, Ching-I Hung**. Role and attitude of companions on geriatric psychiatry outpatient visits in Taiwan.. *Psychiatry and clinical neurosciences*. 2004//. 58:257	Level 1, Form Title/abstract screen
770	**Daliah Heller, Kate McCoy, Chinazo Cunningham**. An invisible barrier to integrating HIV primary care with harm reduction services: philosophical clashes between the harm reduction and medical models.. *Public health reports (Washington, D.C. : 1974)*. 2004//. 119:32	Level 1, Form Title/abstract screen
771	**Douglas Zatzick, Peter Roy-Byrne, Joan Russo, Frederick Rivara, RoseAnne Droesch, Amy Wagner, Chris Dunn, Gregory Jurkovich, Edwina Uehara, Wayne Katon**. A randomized effectiveness trial of stepped collaborative care for acutely injured trauma survivors.. *Archives of general psychiatry*. 2004//. 61:498	Level 1, Form Title/abstract screen
773	**Beverly Beltran Cagle**. Kicking butt. A review of smoking cessation strategies.. *Advance for nurse practitioners*. 2004//. 12:61	Level 1, Form Title/abstract screen
774	**Henning Krampe, Thilo Wagner, Heinrich Kufner, Henriette Jahn, Sabina Stawicki, Jennifer Reinhold, Wiebke Timner, Birgit Kroner-Herwig, Hannelore Ehrenreich**. Therapist rotation--a new element in the outpatient treatment of alcoholism.. *Substance use & misuse*. 2004//. 39:135	Level 2, Form Full Text Screening
775	**Wanda K Nicholson, Bergina Brickhouse, Neil R Powe, Yvonne Bronner**. Prenatal patients' views of prenatal care services: a medical center-based assessment of knowledge and intent to use support services.. *Ethnicity & disease*. 2004//. 14:13	Level 1, Form Title/abstract screen
776	**Lewis E Kazis, Donald R Miller, Katherine M Skinner, Austin Lee, Xinhua S Ren, Jack A Clark, William H Rogers, Avron 3rd Spiro, Alfredo Selim, Mark Linzer, Susan M Payne, Dorcas Mansell, R Graeme Fincke**. Patient-reported measures of health: The Veterans Health Study.. *The Journal of ambulatory care management*. 2004//. 27:70	Level 1, Form Title/abstract screen
777	**Douglas B Marlowe**. Integrating substance abuse treatment and criminal justice supervision.. *Science & practice perspectives*. 2003//. 2:4	Level 1, Form Title/abstract screen
778	**Andrew Tatarsky**. Harm reduction psychotherapy: extending the reach of traditional substance use treatment.. *Journal of substance abuse treatment*. 2003//. 25:249	Level 2, Form Full Text Screening

779	**Kathryn Godfrey**. Clinical management where medicine meets management. Get out, stay out.. *The Health service journal*. 2003//. 113:28	Level 1, Form Title/abstract screen
781	**Julia B Graham, Barbara L Brush, Reverend Maureen Andrew**. Spiritual-care process and content: lessons learned from the ECHO Project.. *Journal of the American Academy of Nurse Practitioners*. 2003//. 15:473	Level 1, Form Title/abstract screen
782	**Richard T Penson, Laura A Fergus, Ross J Haston, John R Clark, Andrew Demotses, James J O'Connell, Bruce A Chabner, Thomas J Jr Lynch**. The Kenneth B. Schwartz Center at Massachusetts General Hospital hematology-oncology department: hope for the homeless.. *The oncologist*. 2003//. 8:488	Level 1, Form Title/abstract screen
783	**Eric F Wagner**. Conceptualizing alcohol treatment research for Hispanic/Latino adolescents.. *Alcoholism, clinical and experimental research*. 2003//. 27:1349	Level 2, Form Full Text Screening
784	**J Scott Tonigan**. Project Match treatment participation and outcome by self-reported ethnicity.. *Alcoholism, clinical and experimental research*. 2003//. 27:1340	Level 1, Form Title/abstract screen
785	**Chris Dunn**. Brief motivational interviewing interventions targeting substance abuse in the acute care medical setting.. *Seminars in clinical neuropsychiatry*. 2003//. 8:188	Level 1, Form Title/abstract screen
786	**John Foster, Colin Brewer, Terry Steele**. Naltrexone implants can completely prevent early (1-month) relapse after opiate detoxification: a pilot study of two cohorts totalling 101 patients with a note on naltrexone blood levels.. *Addiction biology*. 2003//. 8:211	Level 1, Form Title/abstract screen
787	**George E Woody, Robert Gallop, Lester Luborsky, Jack Blaine, Arlene Frank, Ihsan M Salloum, David Gastfriend, Paul Crits-Christoph,Cocaine Psychotherapy Study Group**. HIV risk reduction in the National Institute on Drug Abuse Cocaine Collaborative Treatment Study.. *Journal of acquired immune deficiency syndromes (1999)*. 2003//. 33:82	Level 1, Form Title/abstract screen
789	**Kevin Moore, Derek McLaughlin**. Depression: the challenge for all healthcare professionals.. *Nursing standard (Royal College of Nursing (Great Britain) : 1987)*. 2003//. 17:45	Level 1, Form Title/abstract screen
790	**Elias Vasquez, Marie Eileen Onieal**. Substance abuse education for nurse practitioners in primary care.. *Substance abuse*. 2002//. 23:235	Level 1, Form Title/abstract screen
791	**Patricia E Penn, Audrey J Brooks, Brenda DeWitt Worsham**. Treatment concerns of women with co-occurring serious mental illness and substance abuse disorders.. *Journal of psychoactive drugs*. 2002//. 34:355	Level 1, Form Title/abstract screen
792	**Louise Tiffen, Susan Sheridan**. Improving take-up of hepatitis C services.. *Nursing times*. 2002//. 98:30	Level 1, Form Title/abstract screen
793	**Jonathan P Lacro, Laura B Dunn, Christian R Dolder, Susan G Leckband, Dilip V Jeste**. Prevalence of and risk factors for medication nonadherence in patients with schizophrenia: a comprehensive review of recent literature.. *The Journal of clinical psychiatry*. 2002//. 63:892	Level 1, Form Title/abstract screen
794	**Jorg Andreas Nikitopoulos, Bernhard Croissant**. [New holistic treatment strategies in alcoholism: the goal is abstinence].. *Pflege Zeitschrift*. 2002//. 55:545	Level 1, Form Title/abstract screen
795	**R Conviser, M B Pounds**. The role of ancillary services in client-centred systems of care.. *AIDS care*. 2002//. 14 Suppl 1:S119	Level 1, Form Title/abstract

		screen
797	**Kathryn I Pollak, Kimberly S H Yarnall, Barbara K Rimer, Isaac Lipkus, Pauline R Lyna**. Factors associated with patient-recalled smoking cessation advice in a low-income clinic.. *Journal of the National Medical Association*. 2002//. 94:354	Level 1, Form Title/abstract screen
798	**Arthur Margolin, S Kelly Avants, Theodore R Holford**. Interpreting conflicting findings from clinical trials of auricular acupuncture for cocaine addiction: does treatment context influence outcome?.. *Journal of alternative and complementary medicine (New York, N.Y.)*. 2002//. 8:111	Level 1, Form Title/abstract screen
799	**Jamie Winters, William Fals-Stewart, Timothy J O'Farrell, Gary R Birchler, Michelle L Kelley**. Behavioral couples therapy for female substance-abusing patients: effects on substance use and relationship adjustment.. *Journal of consulting and clinical psychology*. 2002//. 70:344	Level 1, Form Title/abstract screen
800	**Mark Latowsky**. Safer injection facilities for injection drug users: the debate continues.. *CMAJ : Canadian Medical Association journal = journal de l'Association medicale canadienne*. 2002//. 166:419	Level 1, Form Title/abstract screen
802	**A Lancelot, J Sims**. Mental illness and substance abuse.. *Nursing times*. 2001//. 97:36	Level 1, Form Title/abstract screen
803	**N Falsafi**. The use of holistic concepts in professional practice.. *Journal of holistic nursing : official journal of the American Holistic Nurses' Association*. 2001//. 19:390	Level 1, Form Title/abstract screen
804	**R C Kessler, P A Berglund, M L Bruce, J R Koch, E M Laska, P J Leaf, R W Manderscheid, R A Rosenheck, E E Walters, P S Wang**. The prevalence and correlates of untreated serious mental illness.. *Health services research*. 2001//. 36:987	Level 1, Form Title/abstract screen
805	**G J Treisman, A F Angelino, H E Hutton**. Psychiatric issues in the management of patients with HIV infection.. *JAMA*. 2001//. 286:2857	Level 1, Form Title/abstract screen
806	**L R Fenton, J J Cecero, C Nich, T L Frankforter, K M Carroll**. Perspective is everything: the predictive validity of six working alliance instruments.. *The Journal of psychotherapy practice and research*. 2001//. 10:262	Level 1, Form Title/abstract screen
808	**M Encinares, K A Lorbergs**. Framing nursing practice within a forensic outpatient service.. *Journal of psychosocial nursing and mental health services*. 2001//. 39:35	Level 1, Form Title/abstract screen
809	**G Bonner**. Decision making for health care professionals: use of decision trees within the community mental health setting.. *Journal of advanced nursing*. 2001//. 35:349	Level 1, Form Title/abstract screen
810	**R MacQueen**. Alcohol and drug services have been patient centred for years.. *BMJ (Clinical research ed.)*. 2001//. 323:111	Level 1, Form Title/abstract screen
811	**H C Mutasa**. Risk factors associated with noncompliance with methadone substitution therapy (MST) and relapse among chronic opiate users in an Outer London community.. *Journal of advanced nursing*. 2001//. 35:97	Level 1, Form Title/abstract screen
812	**B T Hoyt, R L Norton**. Online medical control and initial refusal of care: does it help to talk with the patient?.. *Academic emergency medicine : official journal of the Society for Academic Emergency Medicine*. 2001//. 8:725	Level 1, Form Title/abstract screen
813	**D F Zatzick, P Roy-Byrne, J E Russo, F P Rivara, A Koike, G J Jurkovich, W Katon**. Collaborative interventions for physically injured trauma survivors: a	Level 1, Form Title/abstract

	pilot randomized effectiveness trial.. *General hospital psychiatry*. 2001//. 23:114	screen
814	**P Denning**. Strategies for implementation of harm reduction in treatment settings.. *Journal of psychoactive drugs*. 2001//. 33:23	Level 2, Form Full Text Screening
815	**J P Barber, L Luborsky, R Gallop, P Crits-Christoph, A Frank, R D Weiss, M E Thase, M B Connolly, M Gladis, C Foltz, L Siqueland**. Therapeutic alliance as a predictor of outcome and retention in the National Institute on Drug Abuse Collaborative Cocaine Treatment Study.. *Journal of consulting and clinical psychology*. 2001//. 69:119	Level 2, Form Full Text Screening
816	**K R Williams, J Galas, D Light, C Pepper, C Ryan, A E Kleinmann, R Burright, P Donovick**. Head injury and alexithymia: implications for family practice care.. *Brain injury*. 2001//. 15:349	Level 1, Form Title/abstract screen
817	**R D Fallot, M Harris**. A trauma-informed approach to screening and assessment.. *New directions for mental health services*. 2001//. #volume#:23	Level 1, Form Title/abstract screen
818	**L B Mauksch, S M Tucker, W J Katon, J Russo, J Cameron, E Walker, R Spitzer**. Mental illness, functional impairment, and patient preferences for collaborative care in an uninsured, primary care population.. *The Journal of family practice*. 2001//. 50:41	Level 1, Form Title/abstract screen
819	**E M McGee**. Alcoholics Anonymous and nursing. Lessons in holism and spiritual care.. *Journal of holistic nursing : official journal of the American Holistic Nurses' Association*. 2000//. 18:11	Level 1, Form Title/abstract screen
820	**K E Bauman, S T Ennett, V A Foshee, M Pemberton, T S King, G G Koch**. Influence of a family-directed program on adolescent cigarette and alcohol cessation.. *Prevention science : the official journal of the Society for Prevention Research*. 2000//. 1:227	Level 1, Form Title/abstract screen
821	**M Thompson**. Five giant leaps toward integrating health care delivery and ways to drive organizations to leap or get out of the way.. *The Journal of ambulatory care management*. 2000//. 23:1	Level 1, Form Title/abstract screen
822	**C G Long, M Williams, M Midgley, C R Hollin**. Within-program factors as predictors of drinking outcome following cognitive-behavioral treatment.. *Addictive behaviors*. 2000//. 25:573	Level 1, Form Title/abstract screen
823	**M S Neale, R A Rosenheck**. Therapeutic limit setting in an assertive community treatment program.. *Psychiatric services (Washington, D.C.)*. 2000//. 51:499	Level 1, Form Title/abstract screen
824	**M Olfson, D Mechanic, S Hansell, C A Boyer, J Walkup, P J Weiden**. Predicting medication noncompliance after hospital discharge among patients with schizophrenia.. *Psychiatric services (Washington, D.C.)*. 2000//. 51:216	Level 1, Form Title/abstract screen
825	**G D Comerci, R Schwebel**. Substance abuse: an overview.. *Adolescent medicine (Philadelphia, Pa.)*. 2000//. 11:79	Level 1, Form Title/abstract screen
827	**M Olfson, D Mechanic, C A Boyer, S Hansell, J Walkup, P J Weiden**. Assessing clinical predictions of early rehospitalization in schizophrenia.. *The Journal of nervous and mental disease*. 1999//. 187:721	Level 1, Form Title/abstract screen
828	**Anonymous**. Fiscal year (FY) 1999 funding opportunities. Substance Abuse and Mental Health Services Administration, HHS. Notice of funding availability.. *Federal register*. 1999//. 64:20318	Level 1, Form Title/abstract screen

829	**M S Edmands, L A Hoff, L Kaylor, L Mower, S Sorrell**. Bridging gaps between mind, body, & spirit. Healing the whole person.. *Journal of psychosocial nursing and mental health services*. 1999//. 37:35	Level 1, Form Title/abstract screen
830	**J K Ockene, A Adams, T G Hurley, E V Wheeler, J R Hebert**. Brief physician- and nurse practitioner-delivered counseling for high-risk drinkers: does it work?.. *Archives of internal medicine*. 1999//. 159:2198	Level 2, Form Full Text Screening
831	**J B Frank, M F Rodowski**. Review of psychological issues in victims of domestic violence seen in emergency settings.. *Emergency medicine clinics of North America*. 1999//. 17:657	Level 1, Form Title/abstract screen
832	**O Kampman, K Lehtinen**. Compliance in psychoses.. *Acta psychiatrica Scandinavica*. 1999//. 100:167	Level 1, Form Title/abstract screen
833	**G T Wilson, K L Loeb, B T Walsh, E Labouvie, E Petkova, X Liu, C Waternaux**. Psychological versus pharmacological treatments of bulimia nervosa: predictors and processes of change.. *Journal of consulting and clinical psychology*. 1999//. 67:451	Level 1, Form Title/abstract screen
835	**N el-Guebaly, D C Hodgins, S Armstrong, J Addington**. Methodological and clinical challenges in evaluating treatment outcome of substance-related disorders and comorbidity.. *Canadian journal of psychiatry. Revue canadienne de psychiatrie*. 1999//. 44:264	Level 1, Form Title/abstract screen
837	**L A Lawendowski**. A motivational intervention for adolescent smokers.. *Preventive medicine*. 1998//. 27:A39	Level 2, Form Full Text Screening
838	**D P Stevens, K W Kizer, T W Elwood, G L Warden**. VA aligns health professions education with healthcare priorities.. *Journal of allied health*. 1998//. 27:123	Level 1, Form Title/abstract screen
839	**S Creamer, C McMurtrie**. Special needs of pregnant and parenting women in recovery: a move toward a more woman-centered approach.. *Women's health issues : official publication of the Jacobs Institute of Women's Health*. 1998//. 8:239	Level 1, Form Title/abstract screen
840	**P Crits-Christoph, L Siqueland, J Chittams, J P Barber, A T Beck, A Frank, B Liese, L Luborsky, D Mark, D Mercer, L S Onken, L M Najavits, M E Thase, G Woody**. Training in cognitive, supportive-expressive, and drug counseling therapies for cocaine dependence.. *Journal of consulting and clinical psychology*. 1998//. 66:484	Level 2, Form Full Text Screening
841	**C C Butler, R Pill, N C Stott**. Qualitative study of patients' perceptions of doctors' advice to quit smoking: implications for opportunistic health promotion.. *BMJ (Clinical research ed.)*. 1998//. 316:1878	Level 1, Form Title/abstract screen
842	**D J Hess, C Kenner**. Families caring for children with fetal alcohol syndrome: the nurse's role in early identification and intervention.. *Holistic nursing practice*. 1998//. 12:47	Level 1, Form Title/abstract screen
843	**S N Fisk**. The Association of Nurses in AIDS Care. Position paper on harm reduction and HIV care for drug users: integrating harm-reduction methods and HIV care.. *The Journal of the Association of Nurses in AIDS Care : JANAC*. 1998//. 9:19	Level 1, Form Title/abstract screen
844	**K M Carroll, G J Connors, N L Cooney, C C DiClemente, D M Donovan, R R Kadden, R L Longabaugh, B J Rounsaville, P W Wirtz, A Zweben**. Internal validity of Project MATCH treatments: discriminability and integrity.. *Journal of consulting and clinical psychology*. 1998//. 66:290	Level 1, Form Title/abstract screen

845	**P D Friedmann, R Saitz, J H Samet**. Management of adults recovering from alcohol or other drug problems: relapse prevention in primary care.. *JAMA*. 1998//. 279:1227	Level 2, Form Full Text Screening
846	**S E Teagle, C D Brindis**. Substance use among pregnant adolescents: a comparison of self-reported use and provider perception.. *The Journal of adolescent health : official publication of the Society for Adolescent Medicine*. 1998//. 22:229	Level 1, Form Title/abstract screen
847	**M Vojnovic, M Martinov-Cvejin, V Grujic**. [Biosocial aspects of physician-patient communication in general medicine].. *Medicinski pregled*. 1997//. 50:395	Level 1, Form Title/abstract screen
848	**M Soyka, C Kirchmayer, G Kotter, C John, E Lohnert, H J Moller**. [New possibilities in therapy and rehabilitation of alcohol dependent patients. Catamnestic study of the efficacy of ambulatory withdrawal therapy exemplified by a model facility].. *Fortschritte der Neurologie-Psychiatrie*. 1997//. 65:407	Level 1, Form Title/abstract screen
849	**W S Fenton, C R Blyler, R K Heinssen**. Determinants of medication compliance in schizophrenia: empirical and clinical findings.. *Schizophrenia bulletin*. 1997//. 23:637	Level 1, Form Title/abstract screen
850	**J K Ockene, E V Wheeler, A Adams, T G Hurley, J Hebert**. Provider training for patient-centered alcohol counseling in a primary care setting.. *Archives of internal medicine*. 1997//. 157:2334	Level 2, Form Full Text Screening
851	**Anonymous**. Beyond the Therapeutic Alliance: Keeping the Drug-Dependent Individual in Treatment. Proceedings of a meeting. May 10-11, 1994.. *NIDA research monograph*. 1997//. 165:1	Level 1, Form Title/abstract screen
853	**M H Kearney**. Drug treatment for women: traditional models and new directions.. *Journal of obstetric, gynecologic, and neonatal nursing : JOGNN*. 1997//. 26:459	Level 2, Form Full Text Screening
857	**G Westland**. Biodynamic massage.. *Complementary therapies in nursing & midwifery*. 1996//. 2:47	Level 1, Form Title/abstract screen
858	**L Rew**. Health risks of homeless adolescents. Implications for holistic nursing.. *Journal of holistic nursing : official journal of the American Holistic Nurses' Association*. 1996//. 14:348	Level 1, Form Title/abstract screen
859	**E Arborelius, B Peterson**. Smoking discussions at the child health clinic. A passive, an advisory or a judgemental approach?.. *Scandinavian journal of caring sciences*. 1996//. 10:169	Level 1, Form Title/abstract screen
860	**J L Jacobs, L C Damson, D E Rogers**. One approach to care for patients infected with human immunodeficiency virus in an academic medical center.. *Bulletin of the New York Academy of Medicine*. 1996//. 73:301	Level 1, Form Title/abstract screen
861	**W R Miller**. Motivational interviewing: research, practice, and puzzles.. *Addictive behaviors*. 1996//. 21:835	Level 2, Form Full Text Screening
862	**F D Butterfoss, R M Goodman, A Wandersman**. Community coalitions for prevention and health promotion: factors predicting satisfaction, participation, and planning.. *Health education quarterly*. 1996//. 23:65	Level 1, Form Title/abstract screen
863	**S B Fawcett, A Paine-Andrews, V T Francisco, J A Schultz, K P Richter, R K Lewis, E L Williams, K J Harris, J Y Berkley, J L Fisher**. Using empowerment theory in collaborative partnerships for community health and development.. *American journal of community psychology*. 1995//. 23:677	Level 1, Form Title/abstract screen

864	**C E Grella, J J Annon, M D Anglin**. Ethnic differences in HIV risk behaviors, self-perceptions, and treatment outcomes among women in methadone maintenance treatment.. *Journal of psychoactive drugs*. 1995//. 27:421	Level 1, Form Title/abstract screen
865	**D M Wing, T Thompson**. Causes of alcoholism: a qualitative study of traditional Muscogee (Creek) Indians.. *Public health nursing (Boston, Mass.)*. 1995//. 12:417	Level 2, Form Full Text Screening
866	**R Rosenheck**. Substance abuse and the chronically mentally ill: therapeutic alliance and therapeutic limit-setting.. *Community mental health journal*. 1995//. 31:283	Level 1, Form Title/abstract screen
867	**H Chabrol, R Fouraste**. [Cognitive-behavioral and systemic approaches to drug addiction among adolescents: value and limitations].. *Annales medico-psychologiques*. 1995//. 153:240	Level 1, Form Title/abstract screen
868	**J Finke, L Teusch, M Gastpar**. [Psychotherapy in the psychiatric clinic--an empirical study of the expectations of patients].. *Psychiatrische Praxis*. 1995//. 22:112	Level 1, Form Title/abstract screen
869	**T Allen, P Heffington**. A case study. The challenges of serving mental health and addictions recovery patients.. *PFCA review*. 1994//. #volume#:4	Level 1, Form Title/abstract screen
871	**F Shapiro, S Vogelmann-Sine, L F Sine**. Eye movement desensitization and reprocessing: treating trauma and substance abuse.. *Journal of psychoactive drugs*. 1994//. 26:379	Level 1, Form Title/abstract screen
872	**C C DiClemente, K M Carroll, G J Connors, R M Kadden**. Process assessment in treatment matching research.. *Journal of studies on alcohol. Supplement*. 1994//. 12:156	Level 1, Form Title/abstract screen
873	**L Agapetus**. Yalom's model. Applied to an outpatient better breathers group.. *Journal of psychosocial nursing and mental health services*. 1994//. 32:11	Level 1, Form Title/abstract screen
874	**H Waitzkin, T Britt**. Processing narratives of self-destructive behavior in routine medical encounters: health promotion, disease prevention, and the discourse of health care.. *Social science & medicine (1982)*. 1993//. 36:1121	Level 1, Form Title/abstract screen
875	**M Olfson, I D Glick, D Mechanic**. Inpatient treatment of schizophrenia in general hospitals.. *Hospital & community psychiatry*. 1993//. 44:40	Level 1, Form Title/abstract screen
877	**B Johnson**. Psychoanalysis of a man with active alcoholism.. *Journal of substance abuse treatment*. 1992//. 9:111	Level 1, Form Title/abstract screen
878	**J R Buskirk**. Headlock: psychotherapy of a patient with multiple neurological and psychiatric problems.. *Bulletin of the Menninger Clinic*. 1992//. 56:361	Level 1, Form Title/abstract screen
879	**B Spielman**. Expanding the boundaries of informed consent: disclosing alcoholism and HIV status to patients.. *The American journal of medicine*. 1992//. 93:216	Level 1, Form Title/abstract screen
880	**S S O'Malley, A J Jaffe, G Chang, R S Schottenfeld, R E Meyer, B Rounsaville**. Naltrexone and coping skills therapy for alcohol dependence. A controlled study.. *Archives of general psychiatry*. 1992//. 49:881	Level 1, Form Title/abstract screen
881	**P Lurie, P R Lee**. Fifteen solutions to the problems of prescription drug abuse.. *Journal of psychoactive drugs*. 1991//. 23:349	Level 1, Form Title/abstract screen

882	**W Pfeiffer, W Feuerlein, E Brenk-Schulte**. The motivation of alcohol dependents to undergo treatment.. *Drug and alcohol dependence*. 1991//. 29:87	Level 2, Form Full Text Screening
884	**H J Luderer**. [Client-centered self-experience groups in male alcohol-dependent patients].. *Psychiatrische Praxis*. 1987//. 14:169	Level 1, Form Title/abstract screen
885	**J F Clarkin, S W Hurt, J L Crilly**. Therapeutic alliance and hospital treatment outcome.. *Hospital & community psychiatry*. 1987//. 38:871	Level 1, Form Title/abstract screen
886	**L Wermuth, S Scheidt**. Enlisting family support in drug treatment.. *Family process*. 1986//. 25:25	Level 1, Form Title/abstract screen
887	**R B Millman**. Considerations on the psychotherapy of the substance abuser.. *Journal of substance abuse treatment*. 1986//. 3:103	Level 1, Form Title/abstract screen
888	**J M Ellison, D Jacobs**. Emergency psychopharmacology: a review and update.. *Annals of emergency medicine*. 1986//. 15:962	Level 1, Form Title/abstract screen
889	**J D McCarroll**. Client-centered treatment for the alcoholic in an ambulatory care setting.. *Family & community health*. 1985//. 7:26	Level 2, Form Full Text Screening
890	**M Jones**. Therapeutic communities, old and new.. *The American journal of drug and alcohol abuse*. 1979//. 6:137	Level 1, Form Title/abstract screen
892	**F W Obitz**. Alcoholics' perceptions of selected counseling techniques.. *The British journal of addiction to alcohol and other drugs*. 1975//. 70:187	Level 1, Form Title/abstract screen
893	**S I Greenspan**. The clinical use of operant learning approaches: some complex issues.. *The American journal of psychiatry*. 1974//. 131:852	Level 1, Form Title/abstract screen
894	**P R Kilmann, R J Howell**. Effects of structure of marathon group therapy and locus of control of therapeutic outcome.. *Journal of consulting and clinical psychology*. 1974//. 42:912	Level 1, Form Title/abstract screen
895	**R Kellner**. The evidence in favour of psychotherapy.. *The British journal of medical psychology*. 1967//. 40:341	Level 1, Form Title/abstract screen
896	**S G ALLISON**. Nondirective group therapy of alcoholics in a state hospital.. *Quarterly journal of studies on alcohol*. 1952//. 13:596	Level 1, Form Title/abstract screen
897	**Edwards S.,Levitzky E.,Molina P.E.,Gunaldo T.P.**. Interprofessional education (IPE) toequip healthcare professional students for future team-based alcohol use disorder treatment. *Alcoholism: Clinical and Experimental Research*. 2018//. 42:81A	Level 1, Form Title/abstract screen
898	**Epstein E.E.,McCrady B.S.,Hallgren K.A.**. Individual versusgroup female-specific cognitive behavior therapy for alcohol use disorder. *Alcoholism: Clinical and Experimental Research*. 2018//. 42:294A	Level 1, Form Title/abstract screen
899	**Ludman E.J.,Matson T.,Bobb J.F.,Richards J.E.,Lapham G.T.,Bradley K.A.**. Alcohol-related nurse care management in primary care: Randomized	Level 1, Form Title/abstract

	controlled trial depressive symptom outcomes. *Alcoholism: Clinical and Experimental Research*. 2018//. 42:264A	screen
900	**Borelli J.L.,Sohn L.,Wang B.A.,Hong K.,DeCoste C.,Suchman N.E.**. Therapist-Client Language Matching: Initial Promise as a Measure of Therapist-Client Relationship Quality. *Psychoanalytic Psychology*. 2018//. #volume#:#pages#	Level 2, Form Full Text Screening
901	**Lowenstein L.M.,Deyter G.M.R.,Nishi S.,Wang T.,Volk R.J.**. Shared decision-making conversations and smoking cessation interventions: Critical components of low-dose CT lung cancer screening programs. *Translational Lung Cancer Research*. 2018//. 7:254	Level 1, Form Title/abstract screen
902	**Yedidia M.J.**. Competencies for engaging high-needs patients in primary care. *Healthcare*. 2018//. 6:122	Level 1, Form Title/abstract screen
903	**Ferrari M.,Flora N.,Anderson K.K.,Haughton A.,Tuck A.,Archie S.,Kidd S.,McKenzie K.**. Gender differences in pathways to care for early psychosis. *Early Intervention in Psychiatry*. 2018//. 12:355	Level 1, Form Title/abstract screen
904	**Giannitrapani K.,Glassman P.,Azarfar A.,Silveira M.,Midboe A.,Kerns R.D.,Pearlman R.,Becker W.,Lorenz K.**. Conducting signature informed consent for opioid therapy in patients with cancer: A qualitative analysis of multidisciplinary provider's perspectives. *Journal of General Internal Medicine*. 2018//. 33:148	Level 1, Form Title/abstract screen
907	**Steckowych K.,Smith M.**. Lessons learned and real-world challenges of implementing clinical pharmacy services in a primary care office. *Journal of the American Pharmacists Association*. 2018//. 58:e132	Level 1, Form Title/abstract screen
908	**Gurney M.,Shim C.,Hwang S.**. APPE OTC registry project: Patient decisions, cost savings, and pharmacy consultation. *Journal of the American Pharmacists Association*. 2018//. 58:e103	Level 1, Form Title/abstract screen
909	**Baumann A.,Skirvin J.,Youn R.,Poliskey K.,Ryan V.**. Pharmacists' role in an interdisciplinary team-based care approach to the treatment of opioid use disorder at a community health center. *Journal of the American Pharmacists Association*. 2018//. 58:e174	Level 1, Form Title/abstract screen
913	**Boden S.D.**. Spinescope. *Seminars in Spine Surgery*. 2018//. 30:135	Level 1, Form Title/abstract screen
917	**Goggin K.,Bradley-Ewing A.,Myers A.L.,Lee B.R.,Hurley E.A.,Delay K.B.,Schlachter S.,Ramphal A.,Pina K.,Yu D.,Weltmer K.,Linnemayr S.,Butler C.C.,Newland J.G.**. Protocol for a randomised trial of higher versus lower intensity patient-provider communication interventions to reduce antibiotic misuse in two paediatric ambulatory clinics in the USA. *BMJ Open*. 2018//. 8:020981	Level 1, Form Title/abstract screen
918	**Morley J.,Levak S.,Kapoor S.,Ritter M.K.,O'Grady M.,Morgenstern J.,Conigliaro J.**. Project reduce: Beyond the brief intervention. buildingamodelfortreatingalcoholusedisorder within a patient centered medical home-early lessons. *Journal of General Internal Medicine*. 2018//. 33:827	Level 1, Form Title/abstract screen
919	**Conneely A.,Marshall S.,Bristowe K.,McQuillan R.**. Qualitative study exploring the experience of homelessness staff working with homeless people with life-limiting illnesses in Dublin, Ireland. *Palliative Medicine*. 2018//. 32:88	Level 1, Form Title/abstract screen
920	**Hudson B.F.,Shulman C.,Brophy N.,Kennedy P.,Stone P.**. Developing and evaluating a training workshop for hostel staff around supporting homeless	Level 1, Form Title/abstract

	people with advanced ill health. *Palliative Medicine*. 2018//. 32:123	screen
921	**Moore K.**. The perils of the incidentaloma: A case of lung cancer screening gone wrong. *Journal of General Internal Medicine*. 2018//. 33:642	Level 1, Form Title/abstract screen
922	**Spacht A.,Tang J.W.,Farnan J.M.,Kostas T.,Arora V.M.,Scheetz M.H.,White S.R.,Press V.G.**. Engaging pritzker students in inter-professional chronic disease management: Integrating the curriculum and defining value added roles within the recover QI program. *Journal of General Internal Medicine*. 2018//. 33:703	Level 1, Form Title/abstract screen
923	**Schapira M.M.,Rodriquez K.,Kaminstein D.,Chhatre S.,Kravetz J.,Bastian L.A.,Asan O.,Prigge J.,Villegas J.,Hruska K.L.,Karas C.,Shea J.A.,Fraenkel L.**. Implementation of lung cancer screening decision support in the VA: Where providers and patients thinking converges and diverges. *Journal of General Internal Medicine*. 2018//. 33:236	Level 1, Form Title/abstract screen
924	**Soh M.,Schutz K.,Cowan B.J.,Kopelson K.,Seragaki S.,Keenan C.,Gelberg L.,Warde C.**. Developing and assessing a super huddle for an interprofessional (IP) training program. *Journal of General Internal Medicine*. 2018//. 33:698	Level 1, Form Title/abstract screen
925	**Percac-Lima S.,Hodgkin D.,Ashburner J.M.,Pandharipande P.,Park E.R.,Gorton E.,Atlas S.J.**. Provider perceptions and costs outcomes of a community-health center based patient navigation program for lung cancer screening. *Journal of General Internal Medicine*. 2018//. 33:316	Level 1, Form Title/abstract screen
926	**Laks J.,Patel K.,Alford D.P.,Jones S.M.,Armstrong S.E.,Waite K.,Henault L.,Paasche-Orlow M.K.**. Just sign here? A national survey of opioid treatment agreements for chronic pain management. *Journal of General Internal Medicine*. 2018//. 33:251	Level 1, Form Title/abstract screen
927	**Jacob R.A.,Paranjape A.**. Trauma alert! How social complexity contributes to medical complexity. *Journal of General Internal Medicine*. 2018//. 33:379	Level 1, Form Title/abstract screen
928	**Taksler G.B.,DeGrandis F.M.,Montori V.M.,Nagykaldi Z.,Rothberg M.B.**. Individualizing disease prevention for middle-aged adults: A pilot study. *Journal of General Internal Medicine*. 2018//. 33:244	Level 1, Form Title/abstract screen
932	**Isenberg S.R.,Maragh-Bass A.C.,Ridgeway K.,Beach M.C.,Knowlton A.R.**. A qualitative exploration of chronic pain and opioid treatment among HIV patients with drug use disorders. *Journal of Opioid Management*. 2017//. 13:5	Level 1, Form Title/abstract screen
939	**Viron M.**. Smoke free program at Massachusetts mental health center: A peer-supported approach. *Psychiatric Services*. 2016//. 67:696	Level 1, Form Title/abstract screen
942	**Kowalski A.J.,Poongothai S.,Chwastiak L.,Hutcheson M.,Tandon N.,Khadgawat R.,Sridhar G.R.,Aravind S.R.,Sosale B.,Anjana R.M.,Rao D.,Sagar R.,Mehta N.,Narayan K.M.V.,Unutzer J.,Katon W.,Mohan V.,Ali M.K.**. The INtegrating DEPrEssioN and Diabetes treatmENT (INDEPENDENT) study: Design and methods to address mental healthcare gaps in India. *Contemporary Clinical Trials*. 2017//. 60:113	Level 1, Form Title/abstract screen
950	**Feng X.,Tan X.,Riley B.,Zheng T.,Bias T.,Sambamoorthi U.**. Polypharmacy and Multimorbidity among Medicaid Enrollees: A Multistate Analysis. *Population Health Management*. 2018//. 21:123	Level 1, Form Title/abstract screen
954	**Kirchhof P.,Benussi S.,Kotecha D.,Ahlsson A.,Atar D.,Casadei B.,Castella M.,DIener H.-C.,Heidbuchel H.,Hendriks J.,Hindricks G.,Manolis A.S.,Oldgren J.,Popescu B.A.,Schotten U.,Van Putte B.,Vardas P.,Agewall**	Level 1, Form Title/abstract screen

	**S.,Camm J.,Baron Esquivias G.,Budts W.,Carerj S.,Casselman F.,Coca A.,De Caterina R.,Deftereos S.,Dobrev D.,Ferro J.M.,Filippatos G.,Fitzsimons D.,Gorenek B.,Guenoun M.,Hohnloser S.H.,Kolh P.,Lip G.Y.H.,Manolis A.,McMurray J.,Ponikowski P.,Rosenhek R.,Ruschitzka F.,Savelieva I.,Sharma S.,Suwalski P.,Tamargo J.L.,Taylor C.J.,Van Gelder I.C.,Voors A.A.,Windecker S.,Zamorano J.L.,Zeppenfeld K.**. 2016 ESC Guidelines for the management of atrial fibrillation developed in collaboration with EACTS. *European Heart Journal*. 2016//. 37:2893	
956	**Danielson E.,Mazurenko O.,Andraka-Christou B.,Dilulio J.,Downs S.,Hurley R.,Harle C.**. How do primary care clinicians and patients discuss risks, benefits, and goals in chronic opioid therapy?. *Journal of Pain*. 2018//. 19:S38	Level 1, Form Title/abstract screen
959	**O'connor M.K.,Mueller L.,Kwon E.,Drebing C.E.,O'Connor A.A.,Semiatin A.,Wang S.,Daley R.**. Enhanced vocational rehabilitation for Veterans with mild traumatic brain injury and mental illness: Pilot study. *Journal of Rehabilitation Research and Development*. 2016//. 53:307	Level 1, Form Title/abstract screen
961	**Tucker C.B.**. Bridging the gap: Increasing the screening for depression among high-risk adolescents. *Ochsner Journal*. 2018//. 18:e12	Level 1, Form Title/abstract screen
965	**Carter B.L.**. Primary care physician-pharmacist collaborative care model: Strategies for implementation. *Pharmacotherapy*. 2016//. 36:363	Level 1, Form Title/abstract screen
966	**Mazzone P.J.,Silvestri G.A.,Patel S.,Kanne J.P.,Kinsinger L.S.,Wiener R.S.,Soo Hoo G.,Detterbeck F.C.**. Screening for Lung Cancer: CHEST Guideline and Expert Panel Report. *Chest*. 2018//. 153:954	Level 1, Form Title/abstract screen
967	**Peppas I.,Fitchett E.J.A.,Kenworthy W.,Kimkool P.,Fertleman C.R.**. Designing transformative osces: Lessons from an adolescent smoking cessation station. *Archives of Disease in Childhood*. 2018//. 103:A76	Level 1, Form Title/abstract screen
968	**Malhi G.S.,Outhred T.,Hamilton A.,Boyce P.M.,Bryant R.,Fitzgerald P.B.,Lyndon B.,Mulder R.,Murray G.,Porter R.J.,Singh A.B.,Fritz K.**. Royal australian and New Zealand college of psychiatrists clinical practice guidelines for mood disorders: Major depression summary. *Medical Journal of Australia*. 2018//. 208:175	Level 1, Form Title/abstract screen
971	**Spattini L.,Mattei G.,Raisi F.,Ferrari S.,Pingani L.,Galeazzi G.M.**. Efficacy of animal assisted therapy on people with mental disorders: An update on the evidence. *Minerva Psichiatrica*. 2018//. 59:54	Level 1, Form Title/abstract screen
973	**Erkmen C.,Mitchell M.**. Lung cancer screening in a predominantly African-American population. *International Journal of Radiation Oncology Biology Physics*. 2017//. 98:242	Level 1, Form Title/abstract screen
974	**Dixon L.B.,Holoshitz Y.,Nossel I.**. Treatment engagement of individuals experiencing mental illness: Review and update. *World Psychiatry*. 2016//. 15:13	Level 1, Form Title/abstract screen
976	**Garvey W.T.,Hurley D.L.,Kushner R.F.**. Patient-centered care of the patient with obesity. *Endocrine Practice*. 2016//. 22:1	Level 1, Form Title/abstract screen
978	**Dissanayake V.,Dalka E.T.,Koh C.,Bisanzo M.,Brandt R.S.,Erickson T.B.,Chamberlain S.**. A pilot study on the management and outcomes of self-poisoning in a rural Ugandan Emergency Centre. *African Journal of Emergency Medicine*. 2018//. 8:25	Level 1, Form Title/abstract screen

985	**Randhawa S.,Moore R.F.,DiSesa V.,Kaiser L.,Ma G.X.,Jaklitsch M.T.,Erkmen C.P.**. Role of Thoracic Surgeons in Lung Cancer Screening: Opportune Time for Involvement. *Journal of Thoracic Oncology*. 2018//. 13:e33	Level 1, Form Title/abstract screen
987	**Mazzone P.J.**. Obstacles to and Solutions for a Successful Lung Cancer Screening Program. *Seminars in Respiratory and Critical Care Medicine*. 2016//. 37:659	Level 1, Form Title/abstract screen
990	**Garber G.,Petok A.,McLafferty L.,Jangro W.,Bahnsen R.,Vaughan-Briggs C.**. Collaborative care approach to managing acute psychiatric conditions in patients with cancer. *Psycho-Oncology*. 2018//. 27:25	Level 1, Form Title/abstract screen
991	**Shin S.,Leslie K.,Carlisle C.,Rosenkranz S.,Abid S.,Tulloch T.**. Medical complexity and concurrent disorders in adolescents. *Journal of Adolescent Health*. 2018//. 62:S128	Level 1, Form Title/abstract screen
993	**Jacobson F.L.,Jaklitsch M.T.**. Computed Tomography Scanning for Early Detection of Lung Cancer. *Annual Review of Medicine*. 2018//. 69:235	Level 1, Form Title/abstract screen
995	**Carson N.J.,Katz A.M.,Alegria M.**. How patients and clinicians make meaning of physical suffering in mental health evaluations. *Transcultural Psychiatry*. 2016//. 53:595	Level 1, Form Title/abstract screen
996	**Irena A.,Reid K.,Battiola R.**. Bias in the eyes of resident physicians. *Wisconsin Medical Journal*. 2017//. 116:269	Level 1, Form Title/abstract screen
999	**Jansson I.,Fors A.,Ekman I.,Ulin K.**. Documentation of person-centred health plans for patients with acute coronary syndrome. *European Journal of Cardiovascular Nursing*. 2018//. 17:114	Level 1, Form Title/abstract screen
1003	**Portacolone E.,Covinsky K.,Johnson J.,Rubinstein R.,Halpern J.**. The perspective of people with cognitive impairment on therapeutic alliances: Acase study. *Alzheimer's and Dementia*. 2017//. 13:P489	Level 1, Form Title/abstract screen
1005	**Castillo E.G.,Shaner R.,Tang L.,Chung B.,Jones F.,Whittington Y.,Miranda J.,Wells K.B.**. Improving depression care for adults with serious mental illness in underresourced areas: Community coalitions versus technical support. *Psychiatric Services*. 2018//. 69:195	Level 1, Form Title/abstract screen
1013	**Balekian A.A.,Tanner N.T.,Fisher J.M.,Silvestri G.A.,Gould M.K.**. Factors associated with a positive baseline screening exam result in the national lung screening trial. *Annals of the American Thoracic Society*. 2016//. 13:1568	Level 1, Form Title/abstract screen
1020	**Copeland A.,Criswell A.**. The primary care provider role in the us screening context: Current practices and strategies for physician engagement. *Journal of Thoracic Oncology*. 2017//. 12:S2170	Level 1, Form Title/abstract screen
1021	**Fathi J.**. Communicating complex issues simply: Pivotal role of nursing in lung cancer screening. *Journal of Thoracic Oncology*. 2017//. 12:S1743	Level 1, Form Title/abstract screen
1023	**Henderson J.L.,Cheung A.,Cleverley K.,Chaim G.,Moretti M.E.,De Oliveira C.,Hawke L.D.,Willan A.R.,O'Brien D.,Heffernan O.,Herzog T.,Courey L.,McDonald H.,Grant E.,Szatmari P.**. Integrated collaborative care teams to enhance service delivery to youth with mental health and substance use challenges: Protocol for a pragmatic randomised controlled trial. *BMJ Open*. 2017//. 7:e014080	Level 1, Form Title/abstract screen
1028	**Ahn M.S.**. Beyond the prescription pad: Psychotherapy interventions for parents/caregivers of youth with serious mental illnesses and treatment nonadherence. *Journal of the American Academy of Child and Adolescent*	Level 1, Form Title/abstract screen

	Psychiatry. 2017//. 56:S23	
1029	**Rackley S.J.**. Collaborative care for adolescent depression: The Mayo Clinic experience. *Journal of the American Academy of Child and Adolescent Psychiatry*. 2017//. 56:S105	Level 1, Form Title/abstract screen
1030	**Al-Mateen C.S.**. Unconscious bias, microaggressions, and health. *Journal of the American Academy of Child and Adolescent Psychiatry*. 2017//. 56:S76	Level 1, Form Title/abstract screen
1031	**Krampe H.,Stawicki S.,Ribbe K.,Wagner T.,Bartels C.,Kroener-Herwig B.,Ehrenreich H.**. Development of an outcome prediction measure for alcoholism therapy by multimodal monitoring of treatment processes. *Journal of Psychiatric Research*. 2008//. 43:30	Level 1, Form Title/abstract screen
1037	**Kuerbis A.,Houser J.,Levak S.,Shao S.,Morgenstern J.**. Exploration of treatment matching of problem drinker characteristics to motivational interviewing and non-directive client-centered psychotherapy. *Journal of Substance Abuse Treatment*. 2018//. 86:9	Level 1, Form Title/abstract screen
1042	**Ravi A.,Pfeiffer M.R.,Rosner Z.,Shea J.A.**. Trafficking and Trauma: Insight and Advice for the Healthcare System from Sex-trafficked Women Incarcerated on Rikers Island. *Medical Care*. 2017//. 55:1017	Level 1, Form Title/abstract screen
1043	**Gilbert C.R.,Ely R.,Fathi J.T.,Louie B.E.,Wilshire C.L.,Modin H.,Aye R.W.,Farivar A.S.,Vallieres E.,Gorden J.A.**. The economic impact of a nurse practitioner-directed lung cancer screening, incidental pulmonary nodule, and tobacco-cessation clinic. *Journal of Thoracic and Cardiovascular Surgery*. 2018//. 155:416	Level 1, Form Title/abstract screen
1044	**Bristow B.,Lewis D.**. Electronic smoking cessation documentation in an ambulatory cancer centre: Development and implementation. *Journal of Medical Imaging and Radiation Sciences*. 2015//. 46:S24	Level 1, Form Title/abstract screen
1045	**Plevinsky J.,Miller S.,Maddux M.,Fishman L.,Noe J.,Kahn S.,Greenley R.**. Substance use and self-management in college students with inflammatory bowel diseases (IBD). *Journal of Pediatric Gastroenterology and Nutrition*. 2017//. 65:S153	Level 1, Form Title/abstract screen
1049	**Sairenji T.,Collins K.L.,Evans D.V.**. An Update on Inflammatory Bowel Disease. *Primary Care - Clinics in Office Practice*. 2017//. 44:673	Level 1, Form Title/abstract screen
1051	**Orkaby A.R.,Rich M.W.**. Cardiovascular Screening and Primary Prevention in Older Adults. *Clinics in Geriatric Medicine*. 2018//. 34:81	Level 1, Form Title/abstract screen
1052	**Oliva E.M.,Christopher M.L.D.,Wells D.,Bounthavong M.,Harvey M.,Himstreet J.,Emmendorfer T.,Valentino M.,Franchi M.,Goodman F.,Trafton J.A.**. Opioid overdose education and naloxone distribution: Development of the Veterans Health Administration's national program. *Journal of the American Pharmacists Association*. 2017//. 57:S168	Level 1, Form Title/abstract screen
1053	**Erkmen C.P.,Ma G.X.,Sferra S.R.**. Novel use of an electronic medical record to monitor performance of a lung cancer screening program. *Journal of Thoracic Oncology*. 2017//. 12:S1549	Level 1, Form Title/abstract screen
1054	**Moore R.,Sferra S.R.,Kaiser L.R.,DiSesa V.J.,Ma G.X.,Erkmen C.P.**. Implementation of lung cancer screening with quality metrics in a diverse urban population. *Journal of the American College of Surgeons*. 2017//. 225:S120	Level 1, Form Title/abstract screen
1056	**Andreasson S.B.**. Tackling alcohol use: Screening, target group, and patient centred care. *BMJ (Online)*. 2017//. 356:j1119	Level 1, Form Title/abstract

		screen
1067	**Miranda L.S.,Datta S.,Melzer A.C.,Wiener R.S.,Davis J.M.,Tong B.C.,Golden S.E.,Slatore C.G.**. Rationale and design of the lung cancer screening implementation: Evaluation of patient-centered care study. *Annals of the American Thoracic Society*. 2017//. 14:1581	Level 1, Form Title/abstract screen
1068	**Moyers T.B.,Houck J.,Glynn L.H.,Hallgren K.A.,Manuel J.K.**. A randomized controlled trial to influence client language in substance use disorder treatment. *Drug and Alcohol Dependence*. 2017//. 172:43	Level 1, Form Title/abstract screen
1071	**Johnson K.A.T.,Seitz-Brown C.,Anderson K.,Degeorge D.,Blevins E.,Daughters S.B.**. 1-Year post treatment outcomes from a RCT of a behavioral activation treatment for substance use and depression. *Drug and Alcohol Dependence*. 2017//. 171:e96	Level 1, Form Title/abstract screen
1072	**Kelly S.M.,O'Grady K.E.,Gryczynski J.,Mitchell S.G.,Jaffe J.H.,Schwartz R.P.**. Methadone patients in patient-centered treatment: One-year arrest data. *Drug and Alcohol Dependence*. 2017//. 171:e100	Level 2, Form Full Text Screening
1074	**Mann K.,Aubin H.-J.,Charlet K.,Witkiewitz K.**. Can reduced drinking be a viable goal for alcohol dependent patients?. *World Psychiatry*. 2017//. 16:325	Level 1, Form Title/abstract screen
1075	**Howard H.,Clark K.**. Interprofessional-shared decision making for pregnant women with opioid use: Results from a provider training. *Current Women's Health Reviews*. 2017//. 13:121	Level 1, Form Title/abstract screen
1077	**Grad R.,Legare F.,Bell N.R.,Dickinson J.A.,Singh H.,Moore A.E.,Kasperavicius D.,Kretschmer K.L.**. Shared decision making in preventive health care: What it is; What. *Canadian Family Physician*. 2017//. 63:#pages#	Level 1, Form Title/abstract screen
1078	**Michaud G.C.,Channick C.L.,Caplan-Shaw C.,Iaccarino J.M.,Slatore C.G.,Bade B.,Tanner N.,Robitaille C.,Gonzalez A.V.,Goudie E.,Liberman M.,Sharma D.,Shojaee S.,Merrick C.M.,Maldonado F.,Nguyen Q.L.,Rivera-Lebron B.,Poston J.T.**. ATS Core Curriculum 2017: Part IV. Adult pulmonary medicine series editor: Jason T. Poston Part IV Editors: Gaetane C. Michaud, Colleen L. Channick, and Caralee Caplan-Shaw. *Annals of the American Thoracic Society*. 2017//. 14:S196	Level 1, Form Title/abstract screen
1083	**Dowrick C.**. Update on advances in psychiatric treatment in primary care. *BJ Psych Advances*. 2016//. 22:99	Level 1, Form Title/abstract screen
1084	**Young-Hyman D.,De Groot M.,Hill-Briggs F.,Gonzalez J.,Hood K.,Peyrot M.**. Psychosocial care for people with diabetes: A position statement of the American Diabetes Association. Diabetes care 2016;39:2126-2140. *Diabetes Care*. 2017//. 40:e131	Level 1, Form Title/abstract screen
1088	**Clavisi O.,Sabanovic H.,Harris B.,Bywaters L.**. Attitudes and beliefs of opioid medication among patients with musculoskeletal pain. *Internal Medicine Journal*. 2017//. 47:18	Level 1, Form Title/abstract screen
1090	**Santos N.,Chin C.**. Navigating the complexities of patients with substance use disorders through stem cell transplant: The role of the social worker. *Journal of Psychosocial Oncology*. 2016//. 34:140	Level 1, Form Title/abstract screen
1091	**Villafuerte D.,Mangat M.,Malave A.**. Abrikossoff's wheeze. *American Journal of Respiratory and Critical Care Medicine*. 2017//. 195:#pages#	Level 1, Form Title/abstract screen
1092	**Fathi J.T.,Blanshan S.C.,Wilson A.K.,Wilshire C.L.,Gilbert C.R.,Aye**	Level 2, Form

	**R.W.,Farivar A.S.,Louie B.E.,Vallieres E.,Gorden J.A.**. Integrating smoking cessation counseling and treatment in a lung cancer screening program: An effective approach to long-term smoking cessation success. *American Journal of Respiratory and Critical Care Medicine*. 2017//. 195:#pages#	Full Text Screening
1093	**Landon C.,Gallardo K.**. COPD access to community health - Catch smoking cessation in severely mentally ill patients. *American Journal of Respiratory and Critical Care Medicine*. 2017//. 195:#pages#	Level 1, Form Title/abstract screen
1094	**Zeliadt S.,Greene P.,Krebs P.,Klein D.,Ko B.,Swanson L.,Todd K.,Feemster L.C.,Au D.H.,Reinke L.F.,Heffner J.**. Knowledge gaps and biased risk perceptions among current smokers participating in lung cancer screening. *American Journal of Respiratory and Critical Care Medicine*. 2017//. 195:#pages#	Level 1, Form Title/abstract screen
1095	**Nicklas D.,Lane J.L.,Hanson J.,Owens J.,Treitz M.**. Using Digital Stories to Reflect on the Culture of Overuse, Misuse, and Underuse in Medicine and Enhance the Patient-Provider Relationship. *Academic Pediatrics*. 2017//. 17:694	Level 1, Form Title/abstract screen
1096	**Hoffman R.M.,Sanchez R.**. Lung Cancer Screening. *Medical Clinics of North America*. 2017//. 101:769	Level 1, Form Title/abstract screen
1097	**Spithoff S.,Kahan M.,Hardy K.,Clarke S.,Mukerji G.,Robertson A.,Pripstein L.,Molnar L.,Krishnamurthy A.,Lamba W.**. Pilot feasibility study of a primary care and addiction medicine collaborative care model SUN:SHARE. *Canadian Family Physician*. 2016//. 62:S35	Level 1, Form Title/abstract screen
1098	**Rabien A.,Price M.**. Development of a clinical decision support tool for the primary prevention of cardiovascular disease. *Canadian Family Physician*. 2015//. 61:S2	Level 1, Form Title/abstract screen
1099	**Li G.,Thabane L.,Delate T.,Witt D.M.,Levine M.A.H.,Cheng J.,Holbrook A.**. Can we predict individual combined benefit and harm of therapy? Warfarin therapy for atrial fibrillation as a test case. *PLoS ONE*. 2016//. 11:e0160713	Level 1, Form Title/abstract screen
1100	**Baumann M.,Tchicaya A.,Lorentz N.,Le Bihan E.**. Impact of patients' communication with the medical practitioners, on their adherence declared to preventive behaviours, five years after a coronary angiography, in Luxembourg. *PLoS ONE*. 2016//. 11:e0157321	Level 1, Form Title/abstract screen
1102	**Mehta P.,Brown A.,Chung B.,Jones F.,Tang L.,Gilmore J.,Miranda J.,Wells K.**. Community partners in care: 6-month outcomes of two quality improvement depression care interventions in male participants. *Ethnicity and Disease*. 2017//. 27:223	Level 1, Form Title/abstract screen
1103	**Inzucchi S.E.,Bergenstal R.M.,Buse J.B.,Diamant M.,Ferrannini E.,Nauck M.,Peters A.L.,Tsapas A.,Wender R.,Matthews D.R.**. Management of hyperglycemia in type 2 diabetes: A patient-centered approach. *Diabetes Care*. 2012//. 35:1364	Level 1, Form Title/abstract screen
1106	**Percac-Lima S.,Ashburner J.M.,Rigotti N.,Park E.R.,Chang Y.,Atlas S.J.**. Lung cancer screening patient navigation for current smokers in community health centers: A randomized controlled trial. *Journal of Clinical Oncology*. 2017//. 35:#pages#	Level 1, Form Title/abstract screen
1107	**Schneiderhan J.,Clauw D.,Schwenk T.L.**. Primary care of patients with chronic pain. *JAMA - Journal of the American Medical Association*. 2017//. 317:2367	Level 1, Form Title/abstract screen
1108	**Padwa H.,Ling W.,Antonini V.P.,Grossman J.**. Development of a patient-centered research agenda for patients with opioid use disorders. *Journal of*	Level 2, Form Full Text

	Neuroimmune Pharmacology. 2017//. 12:S109	Screening
1109	**Garde E.L.,Manning V.,Lubman D.I.**. Characteristics of clients currently accessing a national online alcohol and drug counselling service. *Australasian Psychiatry*. 2017//. 25:250	Level 1, Form Title/abstract screen
1110	**Blyer K.,Hulton L.**. College students, shared decision making, and the appropriate use of antibiotics for respiratory tract infections: A systematic literature review. *Journal of American college health : J of ACH*. 2016//. 64:334	Level 1, Form Title/abstract screen
1113	**Mailis-Gagnon A.,Nicholson K.,Chaparro L.**. Analysis of complaints to a tertiary care pain clinic over a nine-year period. *Pain Research and Management*. 2010//. 15:17	Level 1, Form Title/abstract screen
1114	**Pignone M.P.,Bibbins-Domingo K.**. Update on Key Clinical Preventive Services for Adults. *Medical Clinics of North America*. 2017//. 101:#pages#	Level 1, Form Title/abstract screen
1117	**Ryan P.,Johnson B.,Rathbun J.,Woo K.**. There is still time to avoid the 4% CMS payment penalty. *Journal of Vascular Surgery: Venous and Lymphatic Disorders*. 2017//. 5:606	Level 1, Form Title/abstract screen
1121	**Srinivasa S.R.**. Use of yoga as a therapy for substance abuse treatment. *Alcoholism: Clinical and Experimental Research*. 2017//. 41:363A	Level 1, Form Title/abstract screen
1122	**Morgenstern J.,Kuerbis A.,Levak S.,Amrhein P.**. Dismantling motivational interviewing (MI) for problem drinkers. *Alcoholism: Clinical and Experimental Research*. 2017//. 41:275A	Level 2, Form Full Text Screening
1123	**Erkmen C.,Sferra S.,Goldman C.,Kaiser L.,Disesa V.,Ma G.**. Smoking patterns in a predominantly African American population undergoing lung cancer screening. *Journal of Thoracic Oncology*. 2017//. 12:S572	Level 1, Form Title/abstract screen
1124	**Christensen J.,Garst J.,Wahidi M.,Hogan C.,Crittenden H.,Bruce S.,D'Amico T.,Tong B.**. A structured lung cancer screening program facilitates patient and provider compliance. *Journal of Thoracic Oncology*. 2017//. 12:S572	Level 1, Form Title/abstract screen
1125	**Henderson L.,Jones L.,Benefield T.,Reuland D.,Brenner A.,Molina P.,Rivera M.**. Appropriateness of lung cancer screening with low dose computed tomography. *Journal of Thoracic Oncology*. 2017//. 12:S565	Level 1, Form Title/abstract screen
1129	**Yuan A.S.,Siggins C.A.,Erekson E.**. Perioperative Management of Older Women in Urogynecologic Surgery. *Current Geriatrics Reports*. 2017//. 6:43	Level 1, Form Title/abstract screen
1133	**Shukla A.,McQueen P.,Alqadri S.,Govindarajan R.**. Compassionate Neurological Care OSCEA Primer for Teaching Patient Centered Care in Neurology Clerkship. *Neurology*. 2017//. 88:#pages#	Level 1, Form Title/abstract screen
1135	**Tzelepis F.,Paul C.L.,Williams C.M.,Gilligan C.,Regan T.,Daly J.,Hodder R.K.,Wiggers J.**. Real-time video counselling for smoking cessation. *Cochrane Database of Systematic Reviews*. 2017//. 2017:CD012659	Level 1, Form Title/abstract screen
1136	**Kuo E.J.,Owen M.,Chok S.,Salman A.,Lema J.,Kalantari H.,Hassen G.**. Predicting different kinds of utilization patterns by frequent users in the emergency department. *Annals of Emergency Medicine*. 2016//. 68:S123	Level 1, Form Title/abstract screen
1137	**Redberg R.F.,Katz M.H.**. Statins for primary prevention: The debate is intense, but the data are weak. *JAMA - Journal of the American Medical Association*. 2016//. 316:1979	Level 1, Form Title/abstract screen
1153	**Oral R.,Ramirez M.,Coohey C.,Nakada S.,Walz A.,Kuntz A.,Benoit**	Level 1, Form

	**J.,Peek-Asa C.**. Adverse childhood experiences and trauma informed care: The future of health care. *Pediatric Research*. 2016//. 79:227	Title/abstract screen
1158	**Beharie N.,Lennon M.C.,McKay M.M.**. Assessing the Relationship between the Perceived Shelter Environment and Mental Health among Homeless Caregivers. *Behavioral Medicine*. 2015//. 41:107	Level 1, Form Title/abstract screen
1159	**St Clair M.C.,Neufeld S.,Jones P.B.,Fonagy P.,Bullmore E.T.,Dolan R.J.,Moutoussis M.,Toseeb U.,Goodyer I.M.**. Characterising the latent structure and organisation of self-reported thoughts, feelings and behaviours in adolescents and young adults. *PLoS ONE*. 2017//. 12:e0175381	Level 1, Form Title/abstract screen
1167	**Chung B.,Ngo V.K.,Ong M.K.,Pulido E.,Jones F.,Gilmore J.,Stoker-Mtume N.,Johnson M.,Tang L.,Wells K.B.,Sherbourne C.,Miranda J.**. Participation in training for depression care quality improvement: A randomized trial of community engagement or technical support. *Psychiatric Services*. 2015//. 66:831	Level 1, Form Title/abstract screen
1168	**Rose G.L.,Guth S.E.,Badger G.J.,Plante D.A.,Fazzino T.L.,Helzer J.E.**. Brief intervention for heavy drinking in primary care: Role of patient initiation. *Journal of Addiction Medicine*. 2015//. 9:368	Level 1, Form Title/abstract screen
1169	**Percac-Lima S.,Ashburner J.M.,Rigotti N.A.,Park E.R.,Atlas S.J.**. Patient navigation program for lung cancer screening in community health centers. *Journal of General Internal Medicine*. 2017//. 32:S782	Level 1, Form Title/abstract screen
1170	**Lowenstein M.,Vijayaraghavan M.,Burke N.J.,Karliner L.S.,Peters M.,Kaplan C.P.**. It's complicated: Patient and physician attitudes towards lung cancer screening implementation. *Journal of General Internal Medicine*. 2017//. 32:S237	Level 1, Form Title/abstract screen
1171	**Hemming P.,Greenblatt L.,Revels J.**. Identifying behavioral health competencies for internal medicine residents: Qualitative interviews with residents, faculty and behavioral health clinicians at a clinic with integrated behavioral health. *Journal of General Internal Medicine*. 2017//. 32:S211	Level 1, Form Title/abstract screen
1172	**Bekelman D.,Allen L.,Hattler B.,Havranek E.P.,Fairclough D.,McBryde C.F.,Meek P.**. Collaborative care to alleviate symptoms and adjust to illness (CASA): Primary efficacy results from the casa randomized clinical trial of a palliative symptom and psychosocial care intervention in heart failure. *Journal of General Internal Medicine*. 2017//. 32:S141	Level 1, Form Title/abstract screen
1173	**Mo S.S.,Hu Y.E.,Nash T.R.,Feingold S.B.**. Tracking patient reported outcome measures in a student-faculty collaborative clinic. *Journal of General Internal Medicine*. 2017//. 32:S357	Level 1, Form Title/abstract screen
1174	**Weinstein E.,Mallano D.,Botnick L.,Hamblin C.,Delgado M.**. Preventing avoidableed visits and inpatient admissions in high risk patients. *Journal of General Internal Medicine*. 2017//. 32:S788	Level 1, Form Title/abstract screen
1175	**Roche M.S.**. The long road home. *Journal of General Internal Medicine*. 2017//. 32:S619	Level 1, Form Title/abstract screen
1178	**Farmer A.D.,Gallagher J.,Bruckner-Holt C.,Aziz Q.**. Narcotic bowel syndrome. *The Lancet Gastroenterology and Hepatology*. 2017//. 2:361	Level 1, Form Title/abstract screen
1179	**Roncero C.,Szerman N.,Teran A.,Pino C.,Vazquez J.M.,Velasco E.,Garcia-Dorado M.,Casas M.**. Professionals' perception on the management of patients with dual disorders. *Patient Preference and Adherence*. 2016//. 10:1855	Level 1, Form Title/abstract screen
1189	**Samson P.,Waters E.A.,Meyers B.,Politi M.C.**. Shared Decision Making and	Level 1, Form

	Effective Risk Communication in the High-Risk Patient with Operable Stage i Non-Small Cell Lung Cancer. *Annals of Thoracic Surgery*. 2016//. 101:2049	Title/abstract screen
1190	**Van Den Heuvel J.,Groenhof K.,Franx A.,Lely T.,Bekker M.**. E-Health: The next generation obstetrical care. *BJOG: An International Journal of Obstetrics and Gynaecology*. 2017//. 124:147	Level 1, Form Title/abstract screen
1191	**Gonzalez K.F.,Meyers S.,Portelli-Gupta J.,Valentine A.,Brick D.,Matthews M.,Suzuki J.**. Undergraduate-level health coaches as volunteers to assist with office-based opioid treatment with buprenorphine. *American Journal on Addictions*. 2017//. 26:285	Level 1, Form Title/abstract screen
1192	**Bisaga A.**. Integrating MAT into systems based practice and innovative collaborative care models, emphasizing data from vermont hub and Spoke System. *American Journal on Addictions*. 2017//. 26:235	Level 1, Form Title/abstract screen
1193	**Edinger J.D.,Grubber J.,Ulmer C.,Zervakis J.,Olsen M.**. A Collaborative paradigm for improving management of sleep disorders in primary care: A randomized clinical trial. *Sleep*. 2016//. 39:237	Level 1, Form Title/abstract screen
1194	**Lewandowski A.N.,Skillings J.L.**. Who gets a lung transplant? Assessing the psychosocial decision-making process for transplant listing. *Global Cardiology Science and Practice*. 2016//. 2016:26	Level 1, Form Title/abstract screen
1200	**Barnicot K.,Gonzalez R.,McCabe R.,Priebe S.**. Skills use and common treatment processes in dialectical behaviour therapy for borderline personality disorder. *Journal of Behavior Therapy and Experimental Psychiatry*. 2016//. 52:147	Level 1, Form Title/abstract screen
1201	**Arnow B.A.,Steidtmann D.,Blasey C.,Manber R.,Constantino M.J.,Klein D.N.,Markowitz J.C.,Rothbaum B.O.,Thase M.E.,Fisher A.J.,Kocsis J.H.**. The relationship between the therapeutic alliance and treatment outcome in two distinct psychotherapies for chronic depression. *Journal of Consulting and Clinical Psychology*. 2013//. 81:627	Level 1, Form Title/abstract screen
1202	**Butow P.,Sharpe L.**. The impact of communication on adherence in pain management. *Pain*. 2013//. 154:S101	Level 1, Form Title/abstract screen
1203	**Pressley J.,Smith R.**. No Ordinary Life: Complex Narratives of Trauma and Resilience in Under-Resourced Communities. *Journal of Aggression, Maltreatment and Trauma*. 2017//. 26:137	Level 1, Form Title/abstract screen
1207	**Ghitza U.E.**. Commentary: Addictions neuroclinical assessment: A neuroscience-based framework for addictive disorders. *Frontiers in Psychiatry*. 2017//. 8:2	Level 1, Form Title/abstract screen
1208	**Munoz R.T.,Fox M.D.,Brahm N.,McIntosh H.C.**. The 'treatment' effect of illicit drug use on the relationship between childhood trauma and perceptions of health among a homeless sample. *Journal of Pharmacy Practice*. 2016//. 29:286	Level 1, Form Title/abstract screen
1209	**McCarthy C.,Bateman M.T.,Wagner M.**. Impact of a clinical pharmacist-led seminar series on primary care provider knowledge of psychotropic medications. *Journal of Pharmacy Practice*. 2016//. 29:309	Level 1, Form Title/abstract screen
1212	**Uliaszek A.A.,Rashid T.,Williams G.E.,Gulamani T.**. Group therapy for university students: A randomized control trial of dialectical behavior therapy and positive psychotherapy. *Behaviour Research and Therapy*. 2016//. 77:78	Level 1, Form Title/abstract screen
1216	**Henry S.G.,Holt Z.B.**. Frustrated Patients and Fearful Physicians. *Journal of General Internal Medicine*. 2017//. 32:148	Level 1, Form Title/abstract screen

1217	**Garcia Fernandez S.**. Adherence to antipsychotic medication in bipolar disorder and schizophrenia patients: A systematic review. *European Neuropsychopharmacology*. 2016//. 26:S434	Level 1, Form Title/abstract screen
1218	**Macneil C.A.,Hasty M.K.,Evans M.,Redlich C.,Berk M.**. The therapeutic alliance: is it necessary or sufficient to engender positive outcomes?. *Acta Neuropsychiatrica*. 2009//. 21:95	Level 1, Form Title/abstract screen
1219	**Prabhu M.,McQuaid-Hanson E.,Hopp S.,Kaimal A.,Leffert L.,Bateman B.T.**. Shared decision-making for opioid prescribing after cesarean delivery. *American Journal of Obstetrics and Gynecology*. 2017//. 216:S469	Level 1, Form Title/abstract screen
1220	**Abbasi F.**. Crisis of faith or mental illness?. *Journal of the American Academy of Child and Adolescent Psychiatry*. 2016//. 55:S22	Level 1, Form Title/abstract screen
1221	**Szigethy E.**. Medical home model in treatment of functional abdominal pain. *Journal of the American Academy of Child and Adolescent Psychiatry*. 2016//. 55:S35	Level 1, Form Title/abstract screen
1222	**Dawson K.L.**. Overview of pharmacological interventions for adolescent tobacco use disorder. *Journal of the American Academy of Child and Adolescent Psychiatry*. 2016//. 55:S20	Level 1, Form Title/abstract screen
1226	**Bluml B.M.,Watson L.L.,Skelton J.B.,Manolakis P.G.,Brock K.A.**. Improving outcomes for diverse populations disproportionately affected by diabetes: Final results of Project IMPACT: Diabetes. *Journal of the American Pharmacists Association*. 2014//. 54:477	Level 1, Form Title/abstract screen
1227	**Buhrer C.**. Extremely immature infants at the limits of viability. *Monatsschrift fur Kinderheilkunde*. 2016//. 164:660	Level 1, Form Title/abstract screen
1228	**Levenson J.S.,Grady M.D.**. The Influence of Childhood Trauma on Sexual Violence and Sexual Deviance in Adulthood. *Acta Anaesthesiologica Belgica*. 2016//. #volume#:#pages#	Level 1, Form Title/abstract screen
1236	**Carroll J.F.X.,Hall C.E.,Kearse R.,Mooney M.,Potestivo J.,Forman N.**. Meeting the treatment needs of veterans with substance use disorders. *Alcoholism Treatment Quarterly*. 2016//. 34:354	Level 1, Form Title/abstract screen
1238	**Catchpole R.E.H.,Brownlie E.B.**. Characteristics of youth presenting to a Canadian youth concurrent disorders program: Clinical complexity, trauma, adaptive functioning and treatment priorities. *Journal of the Canadian Academy of Child and Adolescent Psychiatry*. 2016//. 25:106	Level 1, Form Title/abstract screen
1241	**Bollard E.R.**. The Management of Chronic Pain: What Do We Know, What Do We Do, and How Should We Redesign Our Comprehensive Assessment and Treatment in order to Provide for More Patient-Centered Care?. *Medical Clinics of North America*. 2016//. 100:#pages#	Level 1, Form Title/abstract screen
1242	**Van Den Heuvel M.,Barozzino T.,Milligan K.,Ford-Jones E.,Freeman S.**. We need psychologists!. *Paediatrics and Child Health (Canada)*. 2016//. 21:e1	Level 1, Form Title/abstract screen
1246	**Jonas D.E.,Garza D.**. An evidence-based approach to screening and providing appropriate interventions for unhealthy alcohol use in primary care settings. *Journal of Comparative Effectiveness Research*. 2016//. 5:521	Level 1, Form Title/abstract screen
1250	**Weinstock T.,Kidambi P.,Channick C.L.,Michaud G.C.,Broaddus C.,Makani S.S.,Soylemez Wiener R.,Wilson K.C.,Thomson C.C.**. Implementation of lung cancer screening programs with low-dose computed tomography in clinical practice. *Annals of the American Thoracic Society*.	Level 1, Form Title/abstract screen

	2016//. 13:425	
1257	**Pang R.,Van Leur P.,Sullivan S.,Wotherspoon C.**. The clinical and psychosocial impacts on management to patients with COPD in harp settings. *Respirology*. 2016//. 21:54	Level 1, Form Title/abstract screen
1258	**Jewell G.,Gera J.,DeRosa M.**. Transforming maternity care: Signature's strong start maternity medical home model. *Obstetrics and Gynecology*. 2016//. 127:106S	Level 1, Form Title/abstract screen
1259	**Anonymous.**. 8th AACR Conference on the Science of Health Disparities in Racial/Ethnic Minorities and the Medically Underserved. *Cancer Epidemiology Biomarkers and Prevention*. 2016//. 25:#pages#	Level 1, Form Title/abstract screen
1260	**Coyne I.,Gibson F.,Shields L.,Sheaf G.,Leclercq E.,O'Mathuna D.**. Interventions for promoting participation in shared decision-making for children with cancer: Update of a cochrane review. *Pediatric Blood and Cancer*. 2016//. 63:S224	Level 1, Form Title/abstract screen
1261	**Rutka M.,Ferenci T.,Nagy F.,Szepes Z.,Farkas K.,Balint A.,Bor R.,Milassin A.,Lenart Z.,Molnar T.**. Do you think that complementary and alternative medicine use is frequent amongst inflammatory bowel disease patients: Forget it!-results from a comparative study. *Journal of Crohn's and Colitis*. 2016//. 10:S276	Level 1, Form Title/abstract screen
1262	**Moadel A.,Kolidas E.,Ghavamian R.**. Psychosocial needs assessment of underserved prostate cancer patients: Survivorship program planning. *Journal of Clinical Oncology*. 2016//. 34:#pages#	Level 1, Form Title/abstract screen
1263	**Chen A.,Kiersma M.,Hutchison R.,Wright K.**. Psychometric testing of the kiersma-chen empathy scale-patient version. *Journal of the American Pharmacists Association*. 2016//. 56:e107	Level 1, Form Title/abstract screen
1264	**Andress M.,Lanius P.,Ricchetti C.,Haight R.,Willis R.**. Assessing 30-day readmissions and interventions provided by community and hospital pharmacy residents collaborating with a provider in a transitions of care clinic. *Journal of the American Pharmacists Association*. 2016//. 56:e64	Level 1, Form Title/abstract screen
1265	**Zatzick D.**. The design and implementation of a pragmatic trial targeting the full spectrum of alcohol, drug and ptsd comorbidity after injury. *Alcoholism: Clinical and Experimental Research*. 2016//. 40:307A	Level 1, Form Title/abstract screen
1266	**Palmer R.H.C.,Brick L.A.,Agrawal A.,Keller M.C.,Heath A.C.,McGeary J.E.,Bierut L.J.,Knopik V.S.**. Multivariate genetic architecture of DSM-5 alcohol use disorder. *Alcoholism: Clinical and Experimental Research*. 2016//. 40:306A	Level 1, Form Title/abstract screen
1267	**Clark B.J.,Jones J.,Reed K.D.,Burnham E.L.,Douglas I.S.,Van Pelt D.,Moss M.**. Barriers to alcohol treatment in medical intensive care unit survivors with alcohol misuse: A qualitative study. *Alcoholism: Clinical and Experimental Research*. 2016//. 40:243A	Level 1, Form Title/abstract screen
1268	**Kuerbis A.,Houser J.,Amrhein P.,Morgenstern J.**. In-session commitment language predicting weekly average of daily commitment among problem drinkers. *Alcoholism: Clinical and Experimental Research*. 2016//. 40:188A	Level 1, Form Title/abstract screen
1269	**Parrish D.E.,Benjamins L.,Von Sternberg K.,Duron J.,Velasquez M.M.**. Choices-teen: Phase I trial to pilot a gender-specfic intervention to reduce bundled health risks among females in the juvenile justice system. *Alcoholism: Clinical and Experimental Research*. 2016//. 40:64A	Level 1, Form Title/abstract screen
1270	**Dash G.,Garcia T.,Anderson K.G.**. Therapeutic alliance and participant satisfaction in a high school-based group alcohol intervention program.	Level 1, Form Title/abstract

	Alcoholism: Clinical and Experimental Research. 2016//. 40:64A	screen
1271	**Nelson K.M.,Sylling P.W.,Taylor L.,Curtis I.,Rose D.,Schectman G.,Stark R.,Fihn S.D.**. Elements of the patient centered medical home associated with clinical quality: Evidence for access, continuity and care coordination. *Journal of General Internal Medicine*. 2016//. 31:S209	Level 1, Form Title/abstract screen
1272	**Corveleyn A.,Irwin K.**. Cancer care equity for patients with severe mental illness: A case study of a woman with lung cancer and schizophrenia on clozapine. *Psycho-Oncology*. 2016//. 25:140	Level 1, Form Title/abstract screen
1273	**Gulati R.,Nawaz M.,Eltoukhy H.,Pyrsopoulos N.**. A comparative readability analysis of online patient education materials for cirrhosis and liver transplant. *Gastroenterology*. 2016//. 150:S246	Level 1, Form Title/abstract screen
1274	**Papademetriou M.,Perreault G.,Gillespie C.,Zabar S.,Weinshel E.,Williams R.**. Reducing medical errors: Using osces to assess fellows' performance in system based practice milestones. *Gastroenterology*. 2016//. 150:S146	Level 1, Form Title/abstract screen
1275	**Chin C.,Santos N.**. Managing the complexities of patients with substance use disorders through stem cell transplant: Psychoeducation and social work interventions. *Biology of Blood and Marrow Transplantation*. 2016//. 22:S184	Level 1, Form Title/abstract screen
1276	**Slatore C.G.,Au D.H.,Press N.,Wiener R.S.,Golden S.E.,Ganzini L.**. Decision making among Veterans with incidental pulmonary nodules: A qualitative analysis. *Respiratory Medicine*. 2015//. 109:532	Level 1, Form Title/abstract screen
1277	**Ash D.,Suetani S.,Nair J.,Halpin M.**. Recovery-based services in a psychiatric intensive care unit - The consumer perspective. *Australasian Psychiatry*. 2015//. 23:524	Level 1, Form Title/abstract screen
1280	**Levine Baruch R.,Vishnevsky B.,Kalman T.**. Split-care patients and their caregivers: How collaborative is collaborative care?. *Journal of Nervous and Mental Disease*. 2015//. 203:412	Level 1, Form Title/abstract screen
1281	**Rickerby M.L.,Roesler T.A.**. Training Child Psychiatrists in Family-Based Integrated Care. *Child and Adolescent Psychiatric Clinics of North America*. 2015//. 24:501	Level 1, Form Title/abstract screen
1288	**Deck S.M.,Platt P.A.**. Homelessness Is Traumatic: Abuse, Victimization, and Trauma Histories of Homeless Men. *Journal of Aggression, Maltreatment and Trauma*. 2015//. 24:1022	Level 1, Form Title/abstract screen
1290	**Ellsberg M.,Arango D.J.,Morton M.,Gennari F.,Kiplesund S.,Contreras M.,Watts C.**. Prevention of violence against women and girls: What does the evidence say?. *The Lancet*. 2015//. 385:1555	Level 1, Form Title/abstract screen
1292	**Raistrick D.S.,Tober G.W.,Unsworth S.L.**. Attitudes of healthcare professionals in a general hospital to patients with substance misuse disorders. *Journal of Substance Use*. 2015//. 20:56	Level 1, Form Title/abstract screen
1294	**Tsuyuki R.T.**. Supporting patient-centred care / A l'appui des soins centres sur le patient. *Canadian Pharmacists Journal*. 2015//. 148:57	Level 1, Form Title/abstract screen
1295	**Anonymous.**. A patient being treated in a primary care clinic receives collaborative care treatment for hypertension and depression. *American Journal of Psychiatry*. 2015//. 172:722	Level 1, Form Title/abstract screen
1297	**Loza B.,Murawiec S.**. Long-acting injectable (LAI) antipsychotics for schizophrenia treatment: The new treatment standards. *Psychiatria*. 2015//. 12:119	Level 1, Form Title/abstract screen
1304	**Teoh Y.P.**. Advances in the diagnosis and management of dyslipidaemia.	Level 1, Form

	Prescriber. 2015//. 26:21	Title/abstract screen
1306	**Aboujaoude E.,Salame W.,Naim L.**. Telemental health: A status update. *World Psychiatry*. 2015//. 14:223	Level 1, Form Title/abstract screen
1307	**Sicot R.,Yguel J.,Mazeas C.,Kalamarides S.,Questel F.,Azuar J.,Braganca A.,Curac S.,Desaive P.,Harbonnier J.,Trabut J.B.,Hispard E.**. Alcoholic patient's orientation from an emergency department: Situation and perspectives. *Journal Europeen des Urgences et de Reanimation*. 2015//. 27:77	Level 1, Form Title/abstract screen
1308	**McLachlan A.,Sutton T.,Ding P.,Kerr A.**. A Nurse Practitioner Clinic: A Novel Approach to Supporting Patients Following Heart Valve Surgery. *Heart Lung and Circulation*. 2015//. 24:1126	Level 1, Form Title/abstract screen
1309	**Kajeepeta S.,Gelaye B.,Jackson C.L.,Williams M.A.**. Adverse childhood experiences are associated with adult sleep disorders: A systematic review. *Sleep Medicine*. 2015//. 16:320	Level 1, Form Title/abstract screen
1314	**Park M.,Reynolds C.F.**. Depression among older adults with diabetes mellitus. *Clinics in Geriatric Medicine*. 2015//. 31:117	Level 1, Form Title/abstract screen
1322	**Lefebvre K.**. Usability of a mobile application to educate military personnel about prescription drug abuse. *Journal of the American Pharmacists Association*. 2015//. 55:e421	Level 1, Form Title/abstract screen
1323	**Zeev Y.B.,Bovill M.,Bonevski B.,Gould G.**. Indigenous Counselling And Nicotine (ICAN) quit in pregnancy-developing an evidence-based intervention for smoking cessation for indigenous pregnant women. *Asia-Pacific Journal of Clinical Oncology*. 2015//. 11:8	Level 2, Form Full Text Screening
1324	**David P.**. Multidisciplinary approach to a comprehensive CT screening. *Journal of Thoracic Oncology*. 2015//. 10:S156	Level 1, Form Title/abstract screen
1325	**Sawa T.,Eguchi K.,Nakanishi Y.,Nakagawa K.,Mitsudomi T.**. Advocates making a responsible case for high-risk screening. *Journal of Thoracic Oncology*. 2015//. 10:S120	Level 1, Form Title/abstract screen
1326	**Bogojevic G.,Zigmund D.,Ziravac L.**. The problem of non-compliance during treatment of schizophrenia. *European Archives of Psychiatry and Clinical Neuroscience*. 2015//. 265:S66	Level 1, Form Title/abstract screen
1327	**Catino A.,Aloe F.,Piccininno M.E.,Daniele A.,Misino A.,Ricci D.,Damiani B.,Cormio C.,Logroscino A.,Galetta D.**. An interdisciplinary early simultaneous palliative approach in advanced lung cancer (a-LC): Preliminary data in outpatient setting experience. *Journal of Clinical Oncology*. 2015//. 33:#pages#	Level 1, Form Title/abstract screen
1329	**Mitchell S.G.,Monico L.,Lertch E.,Gryczynski J.,Kelly S.M.,O'Grady K.E.,Jaffe J.H.,Schwartz R.P.**. Counselors' views of providing patient-centered methadone treatment in a clinical trial. *Drug and Alcohol Dependence*. 2015//. 156:e154	Level 2, Form Full Text Screening
1330	**Plaisanu C.,Iorgoveanu V.,Neagu A.,Ionescu R.**. Evaluation of patients' awareness regarding glucocorticoids side effects and prevention strategies. *Annals of the Rheumatic Diseases*. 2015//. 74:413	Level 1, Form Title/abstract screen
1331	**Weinshel E.,Balzora S.,Dikman A.,Malter L.,Gillespie C.,Zabar S.**. Experiential faculty development program: Using objective structured clinical examinations (OSCEs) to assess and reinforce practicing physicians' patient-	Level 1, Form Title/abstract screen

	centered care skills. *American Journal of Gastroenterology*. 2015//. 110:S927	
1332	**Belle-Isle L.,Pauly B.,Benoit C.**. At the table with people who use drugs: How is power in decision-making being shared?. *Canadian Journal of Infectious Diseases and Medical Microbiology*. 2015//. 26:119B	Level 1, Form Title/abstract screen
1333	**Farinha T.,Lanius P.,Shea L.,Vatanka P.,Willis R.,Ricchetti C.**. Does a community pharmacist's intervention post hospital discharge have an impact on 30-day or 90-day smoking cessation rates?. *Journal of the American Pharmacists Association*. 2015//. 55:e203	Level 1, Form Title/abstract screen
1334	**Geng S.,Roberts P.,Maris L.,Nichol A.,Bloch D.**. Effects of a non-narcotic pain management program through collaborative practice with pharmacists and physicians. *Journal of the American Pharmacists Association*. 2015//. 55:e188	Level 1, Form Title/abstract screen
1335	**Simmons V.,Diaz D.,Meltzer L.,Hoehn A.,Sutton S.,Meade C.,Jacobsen P.,McCaffrey J.,Haura E.,Brandon T.**. Smoking relapse prevention intervention for cancer patients. *Psycho-Oncology*. 2015//. 24:33	Level 1, Form Title/abstract screen
1337	**Vasilian C.,Tamasan S.**. Changing mentalities in general hospital mental health care-arguments from reality based liaison psychiatry data Romanian experience. *European Psychiatry*. 2015//. 30:611	Level 1, Form Title/abstract screen
1338	**Bradley K.,Lee A.,Richards J.,Ludman E.,Lozano P.,Caldeiro R.**. Improving care for alcohol misuse in medical settings: Patient and family needs. *Alcoholism: Clinical and Experimental Research*. 2015//. 39:205A	Level 2, Form Full Text Screening
1339	**Schlauch R.C.,Connors G.J.,Maisto S.A.,Dearing R.,Prince M.,Duerr M.**. Therapeutic alliance and drinking behavior over the course of treatment for alcohol dependence. *Alcoholism: Clinical and Experimental Research*. 2015//. 39:140A	Level 2, Form Full Text Screening
1340	**Prince M.A.,Connors G.J.,Maisto S.A.,Dearing R.L.**. Within treatment therapeutic alliance rating profiles predict post treatment frequency of alcohol use. *Alcoholism: Clinical and Experimental Research*. 2015//. 39:140A	Level 2, Form Full Text Screening
1341	**Aharonovich E.,Hasin D.,Delker E.,Cannizzaro D.**. Prevalence and clinical correlates of binge drinking among HIV drug abusers in brief interventions. *Alcoholism: Clinical and Experimental Research*. 2015//. 39:20A	Level 1, Form Title/abstract screen
1342	**Lapham G.T.,Lee A.,Williams E.C.,Richards J.,Ludman E.,Bradley K.A.**. The relationship between social network support and readiness to change drinking among primary care patients with unhealthy drinking. *Alcoholism: Clinical and Experimental Research*. 2015//. 39:18A	Level 1, Form Title/abstract screen
1343	**Quinn A.E.,Horgan C.M.,Hodgkin D.,Stewart M.,Brolin M.,Lane N.**. Bundled payment for alcohol use disorder treatment. *Alcoholism: Clinical and Experimental Research*. 2015//. 39:12A	Level 1, Form Title/abstract screen
1344	**Epstein A.J.,Barry C.L.,Fiellin D.A.,Busch S.H.**. Patient valuation of different approaches to mental health and substance use disorder treatment. *Value in Health*. 2015//. 18:A277	Level 1, Form Title/abstract screen
1346	**Jackson S.L.,Chwastiak L.,Chew L.,Kiefer M.M.,Mertens K.,De keyser P.,Lin E.**. Implementation of team-basedcare for diabetes for safetynet population with complex medical and psychosocial comorbidities. *Journal of General Internal Medicine*. 2015//. 30:S536	Level 1, Form Title/abstract screen
1347	**Roth S.L.,Ricketts S.,Shaparin N.,Jacobs C.,Desantis D.,Khalid L.,Haughton L.,Starrels J.L.**. Copilots: A collaborative pain intervention for long-term opioid treatment safety. *Journal of General Internal Medicine*. 2015//. 30:S529	Level 1, Form Title/abstract screen

1348	**Sedhom R.,Sharma R.**. The importance of patient education and communication at hospital discharge: A case of rapid recognition and readmission for guillain-barre syndrome as a result of the teach-back method. *Journal of General Internal Medicine*. 2015//. 30:S463	Level 1, Form Title/abstract screen
1349	**Nelson K.M.,Sylling P.W.,Wong E.,Taylor L.,Helfrich C.D.,Curtis I.,Schectman G.,Stark R.,Fihn S.D.**. Implementation of the patient centered medical home (PCMH) in the veterans health administration (VHA): Associations with clinical outcomes, patient satisfaction, provider burnoutand health care use. *Journal of General Internal Medicine*. 2015//. 30:S185	Level 1, Form Title/abstract screen
1350	**Block L.,LaVine N.A.,Verbsky J.,Conigliaro J.,Chaudhry S.**. Do internal medicine residents perform patient centered medical home EPAs? A mixed-methods study. *Journal of General Internal Medicine*. 2015//. 30:S141	Level 1, Form Title/abstract screen
1351	**Barry C.,Busch S.,Epstein A.,Fiellin D.**. Willingness to enter drug treatment: The role of treatment models, copays and financial incentives. *Drug and Alcohol Dependence*. 2015//. 146:e218	Level 1, Form Title/abstract screen
1352	**Rivera-Suazo S.,Albizu C.E.,Santiago S.,Perez C.**. Stigmatizing experiences while in drug abuse treatment: A qualitative exploration of client's perceptions. *Drug and Alcohol Dependence*. 2015//. 146:e81	Level 1, Form Title/abstract screen
1353	**Morse D.,Cerulli C.,Bandyopadhyay S.,Guido J.J.,Yang H.,Wilson J.L.,Taxman F.**. Support, traumatization, and employment differences in drug court outcomes. *Drug and Alcohol Dependence*. 2015//. 146:e47	Level 1, Form Title/abstract screen
1354	**Tofighi B.,Grossman E.,Buirkle E.,Lee J.D.**. Mobile phone and text messaging in a public sector, office-based buprenorphine program. *Drug and Alcohol Dependence*. 2015//. 146:e4	Level 1, Form Title/abstract screen
1355	**Mullin M.,Kovalenko E.,Ousley P.,Hurd C.,Harris H.,Kinderman A.**. Bringing it to the streets: A novel approach to improve palliative care for homeless adults. *Journal of Pain and Symptom Management*. 2015//. 49:383	Level 1, Form Title/abstract screen
1356	**Tierce-Hazard S.,Sadarangani T.**. Optimizing the primary care management of chronic pain through telecare. *Journal of Clinical Outcomes Management*. 2014//. 21:493	Level 1, Form Title/abstract screen
1357	**Garimella P.S.,Hirsch A.T.**. Peripheral artery disease and chronic kidney disease: Clinical synergy to improve outcomes. *Advances in Chronic Kidney Disease*. 2014//. 21:460	Level 1, Form Title/abstract screen
1358	**Powell A.A.,White K.M.,Partin M.R.,Halek K.,Hysong S.J.,Zarling E.,Kirsh S.R.,Bloomfield H.E.**. More than a score: A qualitative study of ancillary benefits of performance measurement. *BMJ Quality and Safety*. 2014//. 23:651	Level 1, Form Title/abstract screen
1360	**Siantz E.,Aranda M.P.**. Chronic disease self-management interventions for adults with serious mental illness: A systematic review of the literature. *General Hospital Psychiatry*. 2014//. 36:233	Level 1, Form Title/abstract screen
1361	**Brenner C.J.,Shyn S.I.**. Diagnosis and Management of Bipolar Disorder in Primary Care. A DSM-5 Update. *Medical Clinics of North America*. 2014//. 98:1025	Level 1, Form Title/abstract screen
1362	**Ardino V.**. Trauma-informed care: Is cultural competence a viable solution for efficient policy strategies?. *Clinical Neuropsychiatry*. 2014//. 11:45	Level 1, Form Title/abstract screen
1363	**Hollon S.D.,Arean P.A.,Craske M.G.,Crawford K.A.,Kivlahan D.R.,Magnavita J.J.,Ollendick T.H.,Sexton T.L.,Spring B.,Bufka L.F.,Galper D.I.,Kurtzman H.**. Development of clinical practice guidelines.	Level 1, Form Title/abstract screen

	Annual Review of Clinical Psychology. 2014//. 10:213	
1365	**Esquibel A.Y.,Borkan J.**. Doctors and patients in pain: Conflict and collaboration in opioid prescription in primary care. *Pain*. 2014//. 155:2575	Level 1, Form Title/abstract screen
1366	**Chao D.V.K.**. Collaborative care. *Hong Kong Practitioner*. 2014//. 36:1	Level 1, Form Title/abstract screen
1367	**Helling D.K.,Johnson S.G.**. Defining and advancing ambulatory care pharmacy practice: It is time to lengthen our stride. *American Journal of Health-System Pharmacy*. 2014//. 71:1348	Level 1, Form Title/abstract screen
1368	**Tumber P.S.**. Optimizing perioperative analgesia for the complex pain patient: Medical and interventional strategies. *Canadian Journal of Anesthesia*. 2014//. 61:131	Level 1, Form Title/abstract screen
1372	**Rice K.,Bourbeau J.,MacDonald R.,Wilt T.J.**. Collaborative self-management and behavioral change. *Clinics in Chest Medicine*. 2014//. 35:337	Level 1, Form Title/abstract screen
1373	**Bender B.G.**. Nonadherence in chronic obstructive pulmonary disease patients: What do we know and what should we do next?. *Current Opinion in Pulmonary Medicine*. 2014//. 20:132	Level 1, Form Title/abstract screen
1374	**Wiarda N.R.,McMinn M.R.,Peterson M.A.,Gregor J.A.**. Use of technology for note taking and therapeutic alliance. *Psychotherapy*. 2014//. 51:443	Level 1, Form Title/abstract screen
1377	**Glowacz F.,Buzitu R.**. Girls victims of sexual abuse and delinquent trajectory: What factors of resiliency?. *Neuropsychiatrie de l'Enfance et de l'Adolescence*. 2014//. 62:349	Level 1, Form Title/abstract screen
1379	**Chung B.,Ong M.,Ettner S.L.,Jones F.,Gilmore J.,McCreary M.,Sherbourne C.,Ngo V.,Koegel P.,Tang L.,Dixon E.,Miranda J.,Belin T.R.,Wells K.B.**. 12-month outcomes of community engagement versus technical assistance to implement depression collaborative care. *Annals of Internal Medicine*. 2014//. 161:S23	Level 1, Form Title/abstract screen
1380	**Planner C.,Gask L.,Reilly S.**. Serious mental illness and the role of primary care. *Current Psychiatry Reports*. 2014//. 16:458	Level 1, Form Title/abstract screen
1382	**Arnow B.A.,Steidtmann D.**. Harnessing the potential of the therapeutic alliance. *World Psychiatry*. 2014//. 13:238	Level 1, Form Title/abstract screen
1384	**Kramer U.,Berthoud L.,Keller S.,Caspar F.**. Motive-oriented psychotherapeutic relationship facing a patient presenting with narcissistic personality disorder: A case study. *Journal of Contemporary Psychotherapy*. 2014//. 44:71	Level 1, Form Title/abstract screen
1385	**Johnson A.M.,Goldstein L.B.,Bennett P.,O'Brien E.C.,Rosamond W.D.**. Compliance with acute stroke care quality measures in hospitals with and without primary stroke center certification: The North Carolina Stroke Care Collaborative. *Journal of the American Heart Association*. 2014//. 3:e000423	Level 1, Form Title/abstract screen
1386	**Du Plessis J.,Brar S.,Kalluri M.**. A review of fibrotic lung diseases in older age. *CME Journal Geriatric Medicine*. 2014//. 15:58	Level 1, Form Title/abstract screen
1391	**Williams I.L.**. Considering discontinuation of drug use as a potentially traumatizing event. *Mental Health and Substance Use: Dual Diagnosis*. 2014//.	Level 1, Form Title/abstract

	7:1	screen
1392	**Baharudin D.F.,Mohd Hussin A.H.,Sumari M.,Mohamed S.,Zakaria M.Z.,Sawai R.P.**. Family intervention for the treatment and rehabilitation of drug addiction: An exploratory study. *Journal of Substance Use*. 2014//. 19:301	Level 1, Form Title/abstract screen
1394	**Berardi D.,Ferrannini L.,Menchetti M.,Vaggi M.**. Primary care psychiatry in Italy. *Journal of Nervous and Mental Disease*. 2014//. 202:460	Level 1, Form Title/abstract screen
1396	**Yohannes A.M.,Alexopoulos G.S.**. Depression and anxiety in patients with COPD. *European Respiratory Review*. 2014//. 23:345	Level 1, Form Title/abstract screen
1400	**Inzerillo F.,Barrale C.,Catanese G.,Calandra F.**. That obscure object of desire: Psychological and psychodynamic aspects of the relationship between elderly and drug. *Giornale di Gerontologia*. 2014//. 62:506	Level 1, Form Title/abstract screen
1402	**Ruescas-Escolano E.,Orozco-Beltran D.,Gaubert-Tortosa M.,Navarro-Palazon A.,Cordero-Fort A.,Navarro-Perez J.,Carratala-Munuera C.,Pertusa-Martinez S.,Soler-Bahilo E.,Brotons-Munto F.,Bort-Cubero J.,Nunez-Martinez M.A.,Bertomeu-Martinez V.,Lopez-Pineda A.,Gil-Guillen V.F.**. The PROPRESE trial: Results of a new health care organizational model in primary care for patients with chronic coronary heart disease based on a multifactorial intervention. *Atencion Primaria*. 2014//. 46:10	Level 1, Form Title/abstract screen
1413	**Erickson A.,Becker M.,Shaw S.,Kasper K.,Keynan Y.**. Substance use and its impact on care outcomes among HIV-infected individuals in Manitoba. *Canadian Journal of Infectious Diseases and Medical Microbiology*. 2014//. 25:91A	Level 1, Form Title/abstract screen
1414	**Stridsman C.,Zingmark K.,Lindberg A.,Skar L.**. Experiences of well-being when living with chronic obstructive pulmonary disease (COPD). *European Respiratory Journal*. 2014//. 44:#pages#	Level 1, Form Title/abstract screen
1415	**Smith A.L.,MacDonald N.,Thavarajah K.,DiGiovine B.,Kalus J.S.**. Development and implementation of clinical pharmacy services within an outpatient pulmonary clinic. *Pharmacotherapy*. 2014//. 34:e232	Level 1, Form Title/abstract screen
1416	**Johnson S.C.**. Building bridges: Networking to establish the exchange of culturally sensitive addiction treatment modalities and research. *Alcohol and Alcoholism*. 2014//. 49:#pages#	Level 1, Form Title/abstract screen
1417	**Mizusawa T.**. Building bridges: Networking to establish the exchange of culturally sensitive addiction treatment modalities and research. *Alcohol and Alcoholism*. 2014//. 49:#pages#	Level 1, Form Title/abstract screen
1418	**Matsumoto T.**. Building bridges: Networking to establish the exchange of culturally sensitive addiction treatment modalities and research. *Alcohol and Alcoholism*. 2014//. 49:#pages#	Level 1, Form Title/abstract screen
1419	**Saito T.**. The history of alcoholism treatment in Japan. *Alcohol and Alcoholism*. 2014//. 49:#pages#	Level 1, Form Title/abstract screen
1420	**O'Mara B.**. Lessons learned: A partnership approach for 'smoking care' in a housing crisis and homelessness services organisation. *Asia-Pacific Journal of Clinical Oncology*. 2014//. 10:201	Level 1, Form Title/abstract screen
1421	**McNair C.,Alker-Jones H.**. Youth Mental Health Nurse Practitioners: An Australian regional and rural approach. *Early Intervention in Psychiatry*. 2014//. 8:161	Level 1, Form Title/abstract screen

1422	**Addington J.**. Psychosocial treatment in the RAISE-ETP study. *Early Intervention in Psychiatry*. 2014//. 8:1	Level 1, Form Title/abstract screen
1423	**Graffi J.,Quoix E.,Molard A.,Kopferschmitt-Kubler M.-C.,Bacque M.-F.**. Conflict and therapeutic alliance: The refusal of smoking cessation despite medical guidelines. *Psycho-Oncology*. 2014//. 23:230	Level 1, Form Title/abstract screen
1424	**Harley B.**. Should HIV and tuberculosis services be integrated in settings with high TB/HIV co-infection?. *International Journal of Infectious Diseases*. 2014//. 21:44	Level 1, Form Title/abstract screen
1425	**Di Prospero L.S.,Robson S.,Gupta T.D.,Bristow B.,Peacock M.,O'Leary B.**. Leading practice: Integrating best practice initiatives as part of the radiation therapist role. *Journal of Medical Imaging and Radiation Sciences*. 2014//. 45:164	Level 1, Form Title/abstract screen
1426	**Bradley K.A.,Lapham G.T.,Richards J.,Merrill J.O.,Williams E.C.,Lee A.K.,Holden E.,Chavez L.J.,Kivlahan D.R.**. The prevalenceof DSM-IV versus DSM-5 alcohol use disorders (AUD) in primary care patients at high risk for AUD in the choice trial. *Alcoholism: Clinical and Experimental Research*. 2014//. 38:124A	Level 1, Form Title/abstract screen
1427	**Hawkins E.J.,Lapham G.T.,Lee A.K.,Kivlahan D.R.,Berger B.D.,Hebert P.L.,Saxon A.J.,Bradley K.A.**. Prevalence and comorbidity of PTSD among heavy drinkers enrolled in a collaborative care trial for alcohol use disorders in VA primary care. *Alcoholism: Clinical and Experimental Research*. 2014//. 38:63A	Level 1, Form Title/abstract screen
1428	**Williams E.C.,Lapham G.T.,Lee A.K.,Richards J.E.,Berger D.B.,Ludman E.,Bradley K.A.**. Readiness to change and severity of unhealthy alcohol use among primary care patients recruited to a trial of collaborative care. *Alcoholism: Clinical and Experimental Research*. 2014//. 38:57A	Level 1, Form Title/abstract screen
1429	**Ludman E.J.,Williams E.C.,Lapham G.,Richards J.,Chavez L.,Merrill J.,Holden E.,Greenberg D.,Bradley K.**. Baseline prevalence and correlates of depression among heavy drinking veterans enrolled in a collaborative care randomized trial. *Alcoholism: Clinical and Experimental Research*. 2014//. 38:46A	Level 1, Form Title/abstract screen
1430	**Seal K.H.,Sachs E.,Lin T.,Tarasovsky G.,Bertenthal D.,Koenig C.J.**. Implementing change in clinical practice culture: Pain and opioid management bootcamps for VA clinicians. *Journal of General Internal Medicine*. 2014//. 29:S522	Level 1, Form Title/abstract screen
1431	**Pace C.A.,Tsui J.I.,Nader C.,Bergeron L.P.,LaBelle C.T.,Samet J.H.**. Integrating care for hepatitis C virus infection with office-based therapy for opioid dependence. *Journal of General Internal Medicine*. 2014//. 29:S488	Level 1, Form Title/abstract screen
1432	**Bradley K.,Lapham G.T.,Richards J.E.,Merrill J.,Williams E.,Lee A.K.,Holden E.,Chavez L.,Kivlahan D.**. Results of population-based, proactive outreach to nontreatment seeking primary care patients at high risk for aud in the choice trial. *Journal of General Internal Medicine*. 2014//. 29:S202	Level 1, Form Title/abstract screen
1433	**Nelson K.M.,Helfrich C.,Sun H.,Hebert P.,Liu C.-F.,Dolan E.D.,Taylor L.,Wong E.,Maynard C.,Hernandez S.,Sanders W.J.,Curtis I.,Randall I.A.,Schectman G.,Stark R.,Fihn S.D.**. Implementation of the patient centered medical home (PCMH) in the veterans health administration (VHA); Associations with clinical outcomes, patient satisfaction, provider burnout and utilization. *Journal of General Internal Medicine*. 2014//. 29:S123	Level 1, Form Title/abstract screen
1434	**Rodgers M.A.,Shofer F.S.,Rhodes K.V.**. Impact of childhood sexual abuse on	Level 1, Form

	the severity of psychosocial risks among female ED patients. *Academic Emergency Medicine*. 2014//. 21:S59	Title/abstract screen
1435	**Kennedy G.,Ricketts S.,Ceide M.E.,Marcus P.**. Emerging models of psychiatric services in a pioneer accountable care organization: Personal reflections of psychiatrists on the front line. *American Journal of Geriatric Psychiatry*. 2014//. 22:S14	Level 1, Form Title/abstract screen
1436	**Ramirez M.D.,Marsh B.**. Cotton fever: A self-limiting syndrome in IVDA. *Journal of Investigative Medicine*. 2014//. 62:281	Level 1, Form Title/abstract screen
1437	**White J.**. Understanding the value of the lung cancer nurse specialist. *Lung Cancer*. 2014//. 83:S40	Level 1, Form Title/abstract screen
1438	**Leclerc E.,Mansur R.B.,Brietzke E.**. Determinants of adherence to treatment in bipolar disorder: A comprehensive review. *Journal of Affective Disorders*. 2013//. 149:247	Level 1, Form Title/abstract screen
1440	**Pompili M.,Venturini P.,Palermo M.,Stefani H.,Seretti M.E.,Lamis D.A.,Serafini G.,Amore M.,Girardi P.**. Mood disorders medications: Predictors of nonadherence - Review of the current literature. *Expert Review of Neurotherapeutics*. 2013//. 13:809	Level 1, Form Title/abstract screen
1442	**Kuehn B.M.**. Health reform, research pave way for collaborative care for mental illness. *JAMA - Journal of the American Medical Association*. 2013//. 309:2425	Level 1, Form Title/abstract screen
1444	**Elos D.**. A therapeutic community for female patients with dual diagnosis: Protected residence for treatment of sexually abused women Clinical reflections, difficulties in the treatment and hypotheses for intervention in the therapeutic community. *Psychotropes (Belgium)*. 2013//. 19:109	Level 1, Form Title/abstract screen
1445	**Crawford G.B.,Burgess T.A.,Young M.,Brooksbank M.A.,Brown M.**. A patient-centred model of care incorporating a palliative approach: A framework to meet the needs of people with advanced COPD?. *Progress in Palliative Care*. 2013//. 21:286	Level 1, Form Title/abstract screen
1446	**Casimir Y.E.,Williams M.M.,Liang M.Y.,Pitakmongkolkul S.,Slyer J.T.**. Effectiveness of patient-centered self-care education for adults with heart failure on knowledge, self-care behaviors, quality of life, and readmissions: A systematic review protocol. *JBI Database of Systematic Reviews and Implementation Reports*. 2013//. 11:107	Level 1, Form Title/abstract screen
1449	**Shirley E.D.,Sanders D.O.**. The orthopaedic forum :Patient satisfaction: Implications and predictors of success. *Journal of Bone and Joint Surgery - Series A*. 2013//. 95:e691	Level 1, Form Title/abstract screen
1451	**Juska M.,Balon R.**. Chronic non-cancer pain and substance use disorders: Challenges and strategies. *Current Psychiatry*. 2013//. 12:35	Level 2, Form Full Text Screening
1453	**Pavao J.,Turchik J.A.,Hyun J.K.,Karpenko J.,Saweikis M.,McCutcheon S.,Kane V.,Kimerling R.**. Military sexual trauma among homeless veterans. *Journal of General Internal Medicine*. 2013//. 28:S536	Level 1, Form Title/abstract screen
1455	**Matthias M.S.,Krebs E.E.,Collins L.A.,Bergman A.A.,Coffing J.,Bair M.J.**. "I'm Not Abusing or Anything": Patient-physician communication about opioid treatment in chronic pain. *Patient Education and Counseling*. 2013//. 93:197	Level 1, Form Title/abstract screen
1456	**Boiselle P.M.**. Computed tomography screening for lung cancer. *JAMA - Journal of the American Medical Association*. 2013//. 309:1163	Level 1, Form Title/abstract

		screen
1459	**Linden I.A.,Torchalla I.,Krausz M.**. Addiction in maternity: Prevalence of mental illness, substance use, and trauma. *Journal of Aggression, Maltreatment and Trauma*. 2013//. 22:1070	Level 1, Form Title/abstract screen
1461	**Pallaveshi L.,Zisman-Ilani Y.,Roe D.,Rudnick A.**. Psychiatric rehabilitation pertaining to health care environments: Facilitating skills and supports of people with mental illness in relation to their mental and physical health care. *Current Psychiatry Reviews*. 2013//. 9:214	Level 1, Form Title/abstract screen
1466	**Cahana A.,Dansie E.J.,Theodore B.R.,Wilson H.D.,Turk D.C.**. Redesigning Delivery of Opioids to Optimize Pain Management, Improve Outcomes, and Contain Costs. *Pain Medicine (United States)*. 2013//. 14:36	Level 1, Form Title/abstract screen
1467	**Angstman K.B.,Shippee N.D.,MacLaughlin K.L.,Rasmussen N.H.,Wilkinson J.M.,Williams M.D.,Katzelnick D.J.**. Patient self-assessment factors predictive of persistent depressive symptoms 6 months after enrollment in collaborative care management. *Depression and Anxiety*. 2013//. 30:143	Level 1, Form Title/abstract screen
1468	**Wortzel H.S.,Matarazzo B.,Homaifar B.**. A model for therapeutic risk management of the suicidal patient. *Journal of Psychiatric Practice*. 2013//. 19:323	Level 1, Form Title/abstract screen
1470	**Politi M.C.,Wolin K.Y.,Legare F.**. Implementing clinical practice guidelines about health promotion and disease prevention through shared decision making. *Journal of General Internal Medicine*. 2013//. 28:838	Level 1, Form Title/abstract screen
1472	**Goodrich D.E.,Kilbourne A.M.,Nord K.M.,Bauer M.S.**. Mental health collaborative care and its role in primary care settings. *Current Psychiatry Reports*. 2013//. 15:383	Level 1, Form Title/abstract screen
1474	**Munizza C.,Argentero P.,Coppo A.,Tibaldi G.,Di Giannantonio M.,Picci R.L.,Rucci P.**. Public Beliefs and Attitudes towards Depression in Italy: A National Survey. *PLoS ONE*. 2013//. 8:e63806	Level 1, Form Title/abstract screen
1479	**Roberts B.M.**. The seeds of dual diagnosis discourse in an Australian state. *Mental Health and Substance Use: Dual Diagnosis*. 2013//. 6:325	Level 1, Form Title/abstract screen
1490	**Antonov N.,Donitova V.,Sakharova G.**. Survey on smoking cessation among patients in hospitals. *European Respiratory Journal*. 2013//. 42:#pages#	Level 1, Form Title/abstract screen
1491	**Anonymous.**. 5th International Congress on Psychopharmacology & International Symposium on Child and Adolescent Psychopharmacology. *Bulletin of Clinical Psychopharmacology*. 2013//. 23:S1	Level 1, Form Title/abstract screen
1492	**Balouch H.,Noott A.**. The therapeutic alliance between pharmacist and services users accessing substitute treatment for opiate addiction-keys to successful engagement and outcomes. *International Journal of Pharmacy Practice*. 2013//. 21:125	Level 2, Form Full Text Screening
1493	**Levy K.,Plard V.,Jeannin R.,Baubet T.,Reyre A.**. Addicts parents and their toddlers: A therapeutic approach using video. *Archives of Women's Mental Health*. 2013//. 16:S127	Level 1, Form Title/abstract screen
1494	**Grote N.,Katon W.,Lohr M.J.**. Momcare: Culturally relevant treatment services for perinatal depression. *Archives of Women's Mental Health*. 2013//. 16:S4	Level 1, Form Title/abstract screen
1495	**Graffi J.,Quoix E.,Molard A.,Bacque M.-F.**. Personality factors in adhesion to tobacco cessation after lung cancer diagnosis. *Psycho-Oncology*. 2013//. 22:348	Level 1, Form Title/abstract

		screen
1496	**Smith R.C.**. Intensive mental health training for medical residents. *Journal of General Internal Medicine*. 2013//. 28:S469	Level 1, Form Title/abstract screen
1497	**Sue K.,Simon R.E.,Sneh G.,Chaudhary M.J.,Zeidman J.,Cohen M.J.**. Development of a student-faculty collaborative clinic with a special focus on serving post-incarcerated patients. *Journal of General Internal Medicine*. 2013//. 28:S434	Level 1, Form Title/abstract screen
1498	**Singh S.,Chang H.-Y.,Richards T.M.,Weiner J.,Clark J.M.,Segal J.B.**. Glucagon-like peptide-1-based therapies and risk of hospitalization for acute pancreatitis in type 2 diabetes: Population based matched case-control study. *Journal of General Internal Medicine*. 2013//. 28:S82	Level 1, Form Title/abstract screen
1500	**Simon S.R.,Checchi K.,McNair S.S.,Rubin A.,Marcello T.,Bickmore T.**. A pilot study of a computer-based relational agent to screen for substance-use problems in primary care. *Journal of General Internal Medicine*. 2013//. 28:S6	Level 1, Form Title/abstract screen
1501	**Graham D.M.,Espin-Garcia O.,Brown C.,Halytskyy O.,Mahler M.,Pringle D.,Eng L.,Niu C.,Lam C.,Charow R.,Villeneuve J.,Shani R.M.,Tiessen K.,Howell D.,Jones J.M.,Alibhai S.M.H.,Xu W.,Liu G.**. Complementary and alternative medicine and other health behaviors. *Journal of Clinical Oncology*. 2013//. 31:#pages#	Level 1, Form Title/abstract screen
1502	**Schippers G.M.**. Benefits of patient-centred care in alcohol dependence. *European Neuropsychopharmacology*. 2013//. 23:S634	Level 2, Form Full Text Screening
1503	**Di Paula B.A.,Menachery E.**. Pharmacist-physician collaborative care model for suboxone-maintained patients at a health department. *Journal of Pharmacy Practice*. 2013//. 26:305	Level 1, Form Title/abstract screen
1504	**Crotwell S.M.,Brovko J.,Epstein E.,McCrady B.S.**. Studying client, partner, and therapist behavior in alcohol behavioral couple therapy: Relation among therapist common factors and change over time. *Alcoholism: Clinical and Experimental Research*. 2013//. 37:201A	Level 2, Form Full Text Screening
1505	**Agyemang L.,Dearing R.,Duerr M.,Maisto S.,Connors G.**. Therapeutic alliances and post-treatment health functioning following outpatient treatment for an alcohol use disorder. *Alcoholism: Clinical and Experimental Research*. 2013//. 37:145A	Level 2, Form Full Text Screening
1506	**Agyemang L.,Dearing R.,Duerr M.,Connors G.,Maisto S.**. Predictors of therapeutic alliance in the early phases of outpatient treatment for an alcohol use disorder. *Alcoholism: Clinical and Experimental Research*. 2013//. 37:144A	Level 1, Form Title/abstract screen
1507	**Van Royen K.,Remmen R.,Vanmeerbeek M.,Peremans L.**. Collaborative care in the management of addiction to alcohol, illegal drugs, hypnotics and tranquilizers. A review. *European Journal of General Practice*. 2013//. 19:34	Level 1, Form Title/abstract screen
1508	**Chen K.L.,Ratanawongsa N.**. Room to improve: Infrequent use of behavior change counseling and patient-centered communication with diabetic patients in a safety net clinic. *Journal of Investigative Medicine*. 2013//. 61:127	Level 1, Form Title/abstract screen
1509	**Nambiar S.P.,Chand P.K.,Murthy P.,Suman L.N.**. Emotional and interpersonal themes in psychotherapy: Recognizing maintaining factors in men with substance use disorders. *Indian Journal of Psychiatry*. 2013//. 55:S103	Level 2, Form Full Text Screening
1510	**Balhara,Dhal A.**. E-health: Expanding the horizons to address problem alcohol use in India. *Indian Journal of Psychiatry*. 2013//. 55:S12	Level 1, Form Title/abstract screen

1511	**Altman M.,Apter A.J.,Ginsburg K.,Naimi D.R.**. Interviewing adolescents with asthma: Are we doing enough?. *Journal of Allergy and Clinical Immunology*. 2013//. 131:AB236	Level 1, Form Title/abstract screen
1512	**Rosenberger J.G.,Stupiansky N.,Zimet G.D.,Weaver B.,Novak D.S.,Rosenthal S.**. Health care utilization among a national sample of young men who have sex with men recruited from online social and sexual networks. *Journal of Adolescent Health*. 2013//. 52:S36	Level 1, Form Title/abstract screen
1513	**Obradovic I.,Palle C.**. How to improve the attractiveness of services for young drug users ? the benefits of focus group discussions. *Psychotropes (Belgium)*. 2012//. 18:77	Level 1, Form Title/abstract screen
1516	**Khalily M.T.,Wota A.P.,Hallahan B.**. Post-traumatic stress disorder (PTSD) symptoms in adults with psychiatric disorders. *Irish Journal of Psychological Medicine*. 2012//. 29:102	Level 1, Form Title/abstract screen
1517	**Simon G.E.,Imel Z.E.,Ludman E.J.,Steinfeld B.J.**. Is dropout after a first psychotherapy visit always a bad outcome?. *Psychiatric Services*. 2012//. 63:705	Level 1, Form Title/abstract screen
1518	**Bowden C.L.,Perlis R.H.,Thase M.E.,Ketter T.A.,Ostacher M.M.,Calabrese J.R.,Reilly-Harrington N.A.,Gonzalez J.M.,Singh V.,Nierenberg A.A.,Sachs G.S.**. Aims and Results of the NIMH Systematic Treatment Enhancement Program for Bipolar Disorder (STEP-BD). *CNS Neuroscience and Therapeutics*. 2012//. 18:243	Level 1, Form Title/abstract screen
1520	**Servili C.**. An international perspective on youth mental health: The role of primary health care and collaborative care models. *Journal of the Canadian Academy of Child and Adolescent Psychiatry*. 2012//. 21:127	Level 1, Form Title/abstract screen
1522	**Thor-Wiedemann S.,Wiedemann G.J.**. Breast cancer prevention - State of the art. *Klinikarzt*. 2012//. 41:119	Level 1, Form Title/abstract screen
1524	**Tallian K.B.,Hirsch J.D.,Kuo G.M.,Chang C.A.,Gilmer T.,Messinger M.,Chan P.,Daniels C.E.,Lee K.C.**. Development of a pharmacist-psychiatrist collaborative medication therapy management clinic. *Journal of the American Pharmacists Association*. 2012//. 52:e252	Level 1, Form Title/abstract screen
1527	**Bertakis K.D.,Azari R.**. Patient-centered care: The influence of patient and resident physician gender and gender concordance in primary care. *Journal of Women's Health*. 2012//. 21:326	Level 1, Form Title/abstract screen
1528	**Gestuvo M.K.**. Health maintenance in older adults: Combining evidence and individual preferences. *Mount Sinai Journal of Medicine*. 2012//. 79:560	Level 1, Form Title/abstract screen
1534	**Barnicot K.,Katsakou C.,Bhatti N.,Savill M.,Fearns N.,Priebe S.**. Factors predicting the outcome of psychotherapy for borderline personality disorder: A systematic review. *Clinical Psychology Review*. 2012//. 32:400	Level 1, Form Title/abstract screen
1536	**Earl G.L.,Henstenburg J.A.**. Dietary approaches to hypertension: A call to pharmacists to promote lifestyle changes. *Journal of the American Pharmacists Association*. 2012//. 52:637	Level 1, Form Title/abstract screen
1539	**Leib O.,Lutz-Vorderbrugge A.,Borner N.,Godderz W.,Clement T.,Bertram H.,Weber W.**. Preventive measures and screening examinations in the elderly. *Pravention und Rehabilitation*. 2012//. 24:1	Level 1, Form Title/abstract screen
1547	**Bellino S.,Rinaldi C.,Brunetti C.,Bogetto F.**. Interpersonal psychotherapy: Recent indications beyond major depression. *Journal of Psychopathology*. 2012//. 18:359	Level 1, Form Title/abstract screen

1549	**Feldman M.D.,Berkowitz S.A.**. Role of behavioral medicine in primary care. *Current Opinion in Psychiatry*. 2012//. 25:121	Level 1, Form Title/abstract screen
1551	**Soler J.K.,Okkes I.,Oskam S.,van Boven K.,Zivotic P.,Jevtic M.,Dobbs F.,Lamberts H.,van Boven C.,Dijksterhuis P.H.,Groen A.,de Haan J.,Groot A.M.H.,Janssen D.,Polman T.A.L.,Polderman G.O.,Stolp K.E.I.,Valken N.,Veltman M.T.M.,Woerdeman M.,Calleja F.P.,Sammut C.,Sammut M.R.,Sammut D.,Bonnici J.,Buhagiar J.,Baldacchino A.**. An international comparative family medicine study of the transition project data from the Netherlands, Malta and Serbia. Is family medicine an international discipline? Comparing diagnostic odds ratios across populations. *Family Practice*. 2012//. 29:299	Level 1, Form Title/abstract screen
1552	**Rafeyan R.**. Outcomes and cost effectiveness of collaborative care disease management approach in depression patients. *Journal of Managed Care Medicine*. 2012//. 15:10	Level 1, Form Title/abstract screen
1553	**Stepankova L.,Kalvachova M.,Kofiinkova I.**. Seminar for HPH coordinators (Health Promoting Hospitals) and WHO HPH Autumn School 2011 held in Prague. *Casopis Lekaru Ceskych*. 2012//. 151:149	Level 1, Form Title/abstract screen
1554	**Hall K.,Gibbie T.,Lubman D.I.**. Motivational interviewing techniques: Facilitating behaviour change in the general practice setting. *Australian Family Physician*. 2012//. 41:660	Level 1, Form Title/abstract screen
1560	**Couteron J.**. The French network of consultations for young drug consumer. *Neuropsychiatrie de l'Enfance et de l'Adolescence*. 2012//. 60:S26	Level 2, Form Full Text Screening
1561	**Van Dijk D.,Koeter M.W.J.,Hijman R.,Kahn R.S.,Van Den Brink W.**. Effect of cannabis use on the course of schizophrenia in male patients: A prospective cohort study. *Schizophrenia Research*. 2012//. 136:S372	Level 1, Form Title/abstract screen
1562	**Mallampati D.,Knudsen J.,Cohen M.J.,Cunningham J.,Lee C.-M.,Ticona L.,Ignacio R.B.,Bollman B.**. The crimson care collaborative chelsea clinic: Integrating medical, mental, and social healthcare for post-incarceration and urgent care patients in a student-faculty clinic. *Journal of General Internal Medicine*. 2012//. 27:S568	Level 1, Form Title/abstract screen
1563	**Consoli S.M.,Lemogne C.,Guelfi J.D.,Letessier L.,Nordmann J.-P.,Renard J.-P.,Estephan M.,Troy S.,Denis P.,Rouland J.-F.,Sellem E.**. Influence of motivational interviewing training on therapeutic alliance in glaucoma patients. *Psychosomatic Medicine*. 2012//. 74:A42	Level 1, Form Title/abstract screen
1564	**Kelehan M.**. The healthy lifestyle capacity building project for the remote physical activity workforce in Aborginal communties across the Northern Territory, Australia. *Journal of Science and Medicine in Sport*. 2012//. 15:S258	Level 1, Form Title/abstract screen
1565	**Mann K.**. Nalmefene reduces alcohol consumption in alcohol dependent patients, results of randomized controlled trials in Europe. *Alcoholism: Clinical and Experimental Research*. 2012//. 36:18A	Level 1, Form Title/abstract screen
1566	**Carstens C.,Wingenfeld S.,Tam K.**. The relationship between cultural competence, service use, and treatment outcomes. *Journal of Mental Health Policy and Economics*. 2012//. 15:S6	Level 1, Form Title/abstract screen
1567	**Lalonde L.,Goudreau J.,Hudon E.,Duhamel F.,Lussier M.T.,Bareil C.,Levesque L.,Ladouceur M.,Poirier N.,Milard A.**. TRANSIT: A primary care interprofessional intervention programto improve the management of cardiovascular risk factors in patients with multiple chronic conditions. *Journal of Population Therapeutics and Clinical Pharmacology*. 2012//. 19:e143	Level 1, Form Title/abstract screen

1568	**Le S.,Bull V.,Spring S.,Baharom A.,Paramanantham J.,Beswick L.,Mulley W.,Polkinghorne K.,Ratnam D.,Dev A.,Sievert W.,Pianko S.**. Low rates of hepatitis C virus (HCV) treatment and seroclearance associated with reduced graft survival in renal transplant recipients in a single centre Australian cohort. *Journal of Gastroenterology and Hepatology*. 2012//. 27:83	Level 1, Form Title/abstract screen
1569	**Bird L.,Longo R.,Hocking A.,Watson F.,Joubert L.**. Aboriginal cancer care at peter MAC: Connecting and responding (ACCAP). *Journal of Thoracic Oncology*. 2012//. 7:S159	Level 1, Form Title/abstract screen
1570	**Lalonde L.,Bareil C.,Lussier M.-T.,Goudreau J.,Hudon E.**. TRANSIT: Improving interprofessional management of cardiovascular risk in primary care. *Canadian Pharmacists Journal*. 2012//. 145:S45	Level 1, Form Title/abstract screen
1571	**Nirenberg T.D.,Baird J.,Longabaugh R.,Mello M.J.**. Mi-based intervention may decrease the lie: Self reported pretreatment audit scores of court referred youth increase after they receive MI. *Alcoholism: Clinical and Experimental Research*. 2012//. 36:238A	Level 1, Form Title/abstract screen
1572	**Paves A.P.,Hsu S.H.,Liu J.,Lostutter T.W.**. Acculturation, peer drinking, protective behaviors, and alcoholrelated problems in asian and pacific islander american college students: A path model. *Alcoholism: Clinical and Experimental Research*. 2012//. 36:221A	Level 1, Form Title/abstract screen
1573	**Blom A.,Cloete M.,Hendricks N.,Joubert B.,Roux S.,Barnard R.,Snell C.,Marais A.-S.,Seedat S.,Gossage J.P.,Blankenship J.,May P.A.**. High risk pregnant women and case management: Efficacy of prevention in a community with the highest fetal alcohol syndrome prevalence in the world. *Alcoholism: Clinical and Experimental Research*. 2012//. 36:213A	Level 1, Form Title/abstract screen
1574	**Epstein E.E.,McCrady B.S.,Hildebrandt T.,Jensen N.,Cook S.,Gaba A.**. Preliminary efficacy of a single gender female-specific cognitivebehavioral therapy (CBT) group therapy for alcohol dependent women. *Alcoholism: Clinical and Experimental Research*. 2012//. 36:159A	Level 1, Form Title/abstract screen
1575	**Stasiewicz P.R.,Bradizza C.M.**. Identifying multiple mechanisms of change in alcoholism treatment. *Alcoholism: Clinical and Experimental Research*. 2012//. 36:158A	Level 2, Form Full Text Screening
1576	**Khorsandi P.,Dopheide J.A.**. Psychiatric pharmacist management of depression in patients with diabetes. *Journal of Pharmacy Practice*. 2012//. 25:289	Level 1, Form Title/abstract screen
1577	**Flaherty L.T.**. School-based interventions as part of the therapeutic alliance. *Adolescent Psychiatry*. 2012//. 2:105	Level 1, Form Title/abstract screen
1578	**Lewis D.,Osmar K.**. Interprofessional collaboration in the creation and standardization of patient assessment and documentation for radiation therapy. *Journal of Medical Imaging and Radiation Sciences*. 2012//. 43:S57	Level 1, Form Title/abstract screen
1579	**Salvo M.C.,Nigro S.C.**. Cardiometabolic disease: (part1) the pharmacist's tools for managing dyslipidemia and hypertension. *Drug Topics*. 2011//. 155:25	Level 1, Form Title/abstract screen
1584	**Brown P.,Kritzler R.,Petryna M.,Polley C.M.,Tietze K.J.,Binaso K.A.,Watson L.,Ellis W.M.,Bluml B.M.,Cummings J.,Sullivan M.,Fitch M.L.,Belknap B.**. White paper on expanding the role of pharmacists in chronic obstructive pulmonary disease. *Journal of the American Pharmacists Association*. 2011//. 51:203	Level 1, Form Title/abstract screen
1587	**Farro S.A.,Clark C.,Hopkins Eyles C.**. Assessing trauma-informed care	Level 1, Form

	readiness in behavioral health: An organizational case study. *Journal of Dual Diagnosis*. 2011//. 7:228	Title/abstract screen
1588	**Kellogg S.H.,Tatarsky A.**. Re-envisioning addiction treatment: A six-point plan. *Alcoholism Treatment Quarterly*. 2011//. 30:109	Level 1, Form Title/abstract screen
1590	**Tsosie U.,Nannauck S.,Buchwald D.,Russo J.,Trusz S.G.,Foy H.,Zatzick D.**. Staying connected: A feasibility study linking American Indian and Alaska native trauma survivors to their tribal communities. *Psychiatry*. 2011//. 74:349	Level 1, Form Title/abstract screen
1600	**Muzik M.,Thelen K.,Rosenblum K.L.**. Perinatal depression: Detection and treatment. *Neuropsychiatry*. 2011//. 1:179	Level 1, Form Title/abstract screen
1606	**Campo R.A.,Rowland J.H.,Irwin M.L.,Nathan P.C.,Gritz E.R.,Kinney A.Y.**. Cancer prevention after cancer: Changing the paradigm - A report from the american society of preventive oncology. *Cancer Epidemiology Biomarkers and Prevention*. 2011//. 20:2317	Level 1, Form Title/abstract screen
1608	**Diener M.J.,Monroe J.M.**. The relationship between adult attachment style and therapeutic alliance in individual psychotherapy: A meta-analytic review. *Psychotherapy*. 2011//. 48:237	Level 1, Form Title/abstract screen
1613	**Perestelo-Perez L.,Gonzalez-Lorenzo M.,Perez-Ramos J.,Rivero-Santana A.,Serrano-Aguilar P.**. Patient involvement and shared decision-making in mental health care. *Current Clinical Pharmacology*. 2011//. 6:83	Level 1, Form Title/abstract screen
1614	**Tibaldi G.,Salvador-Carulla L.,Garcia-Gutierrez J.C.**. From treatment adherence to advanced shared decision making: New professional strategies and attitudes in mental health care. *Current Clinical Pharmacology*. 2011//. 6:91	Level 1, Form Title/abstract screen
1619	**Woltmann E.M.,Wilkniss S.M.,Teachout A.,McHugo G.J.,Drake R.E.**. Trial of an electronic decision support system to facilitate shared decision making in community mental health. *Psychiatric Services*. 2011//. 62:54	Level 1, Form Title/abstract screen
1621	**Chawla N.**. A multiple-behavior lifestyle intervention for cancer patients and their families delivered in a community-based oncological and hematologic treatment clinic. *Journal of Family Psychotherapy*. 2011//. 22:74	Level 1, Form Title/abstract screen
1622	**Fortune Z.,Barrett B.,Armstrong D.,Coid J.,Crawford M.,Mudd D.,Rose D.,Slade M.,Spence R.,Tyrer P.,Moran P.**. Clinical and economic outcomes from the UK pilot psychiatric services for personality-disordered offenders. *International Review of Psychiatry*. 2011//. 23:61	Level 1, Form Title/abstract screen
1623	**Brinkhaus B.,Lewith G.,Rehberg B.,Heusser P.,Cummings M.,Michalsen A.,Teut M.,Willich S.N.,Irnich D.**. How to treat a patient with chronic low back pain - Methodology and results of the first international case conference of integrative medicine. *Complementary Therapies in Medicine*. 2011//. 19:54	Level 1, Form Title/abstract screen
1624	**Tsai J.,O'Connell M.,Kasprow W.J.,Rosenheck R.A.**. Factors related to rapidity of housing placement in housing and Urban Development-Department of Veterans affairs supportive housing program of 1990s. *Journal of Rehabilitation Research and Development*. 2011//. 48:755	Level 1, Form Title/abstract screen
1626	**Gittins H.,Patel S.,Ennals G.,Plant J.,Redfern J.,Shober M.,Collins A.,Nazareth D.,Davies L.**. Smoking cessation advice (SCA) and referral: Do we need to further educate healthcare professionals?. *European Respiratory Journal*. 2011//. 38:#pages#	Level 1, Form Title/abstract screen
1627	**Tan R.,Su A.,Hendriks M.M.,Mahendran R.**. Collaborative care for improving the clinical outcomes of patients with psychiatric conditions. *Annals of the Academy of Medicine Singapore*. 2011//. 40:S199	Level 1, Form Title/abstract screen

1628	**Dalkilic A.**. Psychopharmacological treatments in eating disorders. *Klinik Psikofarmakoloji Bulteni*. 2011//. 21:S93	Level 1, Form Title/abstract screen
1629	**DiPaula B.A.,Park D.**. Collaborative drug therapy management for buprenorphine-maintained patients. *Journal of Pharmacy Practice*. 2011//. 24:280	Level 1, Form Title/abstract screen
1630	**Elfrink M.**. ACT and Recovery: A new perspective on treatment for early psychosis. The first 3 years: Qualitative and quantitative data. *European Archives of Psychiatry and Clinical Neuroscience*. 2011//. 261:S33	Level 1, Form Title/abstract screen
1631	**Von Sternberg K.,Velasquez M.M.,Ingersoll K.,Green C.,Hettema J.,Wagner C.**. Using latent profile analyses to examine key components of an evidence-based intervention to reduce alcohol-exposed pregnancy: Project choices. *Alcoholism: Clinical and Experimental Research*. 2011//. 35:145A	Level 1, Form Title/abstract screen
1632	**Urbanoski K.A.,Kelly J.F.,Hoeppner B.B.,Slaymaker V.**. The influence of therapeutic alliance on treatment response and outcome among young adults in residential substance use disorder treatment. *Alcoholism: Clinical and Experimental Research*. 2011//. 35:84A	Level 1, Form Title/abstract screen
1633	**Yousif O.E.,Ibrahim A.S.**. TB/HIV among patients attending referred clinics in Shaab Hospital, Khartoum. *Annals of Thoracic Medicine*. 2011//. 6:182	Level 1, Form Title/abstract screen
1635	**Iliopoulou L.,Koutras V.,Komninou K.,Fidi E.,Gonta S.,Basogianni V.**. The management of relapse in an outpatient program for heroin user by administration of naltrexone. *European Psychiatry*. 2011//. 26:#pages#	Level 2, Form Full Text Screening
1636	**Ortega L.,Robles N.,Matrai S.,Gual A.**. The e-mail as a potential therapeutic tool in patients with alcohol dependence: The patient's perspective. *European Psychiatry*. 2011//. 26:#pages#	Level 1, Form Title/abstract screen
1637	**Mcquaid J.,Slater M.,Golish M.,Parkes K.,Chircop-Rollick T.,Rutledge T.,Cone R.,Peterzell D.,Atkinson J.,Nance P.**. Visual and psychosocial feedback intervention for phantom limb pain: Participant characteristics. *Journal of Pain*. 2011//. 12:P76	Level 1, Form Title/abstract screen
1638	**Leucht S.**. Psychoeducation and shared decision making as a way to reduce non-compliance in mental disorders. *European Neuropsychopharmacology*. 2011//. 21:S109	Level 1, Form Title/abstract screen
1639	**Robards F.,Jarrett C.,Bennett D.**. Resources to support psychosocial assessments with young people. *Journal of Adolescent Health*. 2011//. 48:S97	Level 1, Form Title/abstract screen
1655	**Bertakis K.D.**. The influence of gender on the doctor-patient interaction. *Patient Education and Counseling*. 2009//. 76:356	Level 1, Form Title/abstract screen
1658	**Kreyenbuhl J.,Nossel I.R.,Dixon L.B.**. Disengagement from mental health treatment among individuals with schizophrenia and strategies for facilitating connections to care: A review of the literature. *Schizophrenia Bulletin*. 2009//. 35:696	Level 1, Form Title/abstract screen
1659	**Gold S.H.,Hilsenroth M.J.**. Effects of graduate clinicians' personal therapy on therapeutic alliance. *Clinical Psychology and Psychotherapy*. 2009//. 16:159	Level 1, Form Title/abstract screen
1660	**Wisnivesky J.P.,Kattan M.,Evans D.,Leventhal H.,Musumeci-Szabo T.J.,McGinn T.,Halm E.A.**. Assessing the relationship between language proficiency and asthma morbidity among inner-city asthmatics. *Medical Care*.	Level 1, Form Title/abstract screen

	2009//. 47:243	
1662	**Despland J.-N.**. Which psychotherapy for which patient? Research data and clinical issues. *Psychotropes*. 2010//. 16:9	Level 1, Form Title/abstract screen
1665	**Hohman M.,Roads L.,Corbett R.**. Initial validation of a subtle trauma symptom screening scale embedded in a needs assessment given to women entering drug treatment. *Journal of Dual Diagnosis*. 2010//. 6:2	Level 1, Form Title/abstract screen
1668	**Mammucari M.,Fiore M.,Geppetti P.**. Opioids prescription: The art of the two-way communication. *Trends in Medicine*. 2010//. 10:135	Level 1, Form Title/abstract screen
1669	**Webb C.A.,DeRubeis R.J.,Barber J.P.**. Therapist Adherence/Competence and Treatment Outcome: A Meta-Analytic Review. *Journal of Consulting and Clinical Psychology*. 2010//. 78:200	Level 1, Form Title/abstract screen
1670	**Krones T.,Keller H.,Becker A.,Sonnichsen A.,Baum E.,Donner-Banzhoff N.**. The theory of planned behaviour in a randomized trial of a decision aid on cardiovascular risk prevention. *Patient Education and Counseling*. 2010//. 78:169	Level 1, Form Title/abstract screen
1671	**McKinney K.A.,Greenfield B.G.**. Self-compliance at 'Prozac campus'. *Anthropology and Medicine*. 2010//. 17:173	Level 1, Form Title/abstract screen
1673	**Angstman K.B.,DeJesus R.S.,Rohrer J.E.**. Correlation between mental health comorbidity screening scores and clinical response in collaborative care treatment for depression. *Mental Health in Family Medicine*. 2010//. 7:129	Level 1, Form Title/abstract screen
1675	**Hauser W.**. Fibromyalgia syndrome (FMS). *DoctorConsult - The Journal. Wissen fur Klinik und Praxis*. 2010//. 1:e93	Level 1, Form Title/abstract screen
1676	**Kotwani A.,Wattal C.,Katewa S.,Joshic P.C.,Holloway K.**. Factors influencing primary care physicians to prescribe antibiotics in Delhi India. *Family Practice*. 2010//. 27:684	Level 1, Form Title/abstract screen
1679	**Calabro K.S.,Costello T.C.,Prokhorov A.V.**. Denormalization of tobacco use and the role of the pediatric health-care provider. *Pediatric, Allergy, Immunology, and Pulmonology*. 2010//. 23:273	Level 1, Form Title/abstract screen
1683	**Ozanne E.,Esserman L.**. Decision making in breast cancer prevention. *Psicooncologia*. 2010//. 7:299	Level 1, Form Title/abstract screen
1684	**Bamatter W.,Carroll K.M.,Anez L.M.,Paris M.,Ball S.A.,Nich C.,Frankforter T.L.,Suarez-Morales L.,Szapocznik J.,Martino S.**. Informal discussions in substance abuse treatment sessions with Spanish-speaking clients. *Journal of Substance Abuse Treatment*. 2010//. 39:353	Level 1, Form Title/abstract screen
1687	**Bremmon K.E.,La Barge R.A.**. Evaluation of a unique collaborative practice model for tobacco cessation in a veteran population. *Pharmacotherapy*. 2010//. 30:462e	Level 1, Form Title/abstract screen
1688	**Sajatovic M.**. Enhancing treatment adherence: A patient-centered approach. *Bipolar Disorders*. 2010//. 12:48	Level 1, Form Title/abstract screen
1689	**Lalonde L.,Belanger D.,Duhamel F.,Goudreau J.,Hudon E.,Lussier M.-T.,Martin E.,Levesque L.**. Preventing cardiovascular disease in primary care: Priorities for action. *Journal of Population Therapeutics and Clinical*	Level 1, Form Title/abstract screen

	Pharmacology. 2010//. 17:e115	
1690	**Jacobsen B.,Elfrink M.B.,Gijsman H.J.**. Collaboration works! Remission and recovery in ACT for early psychosis. *Early Intervention in Psychiatry*. 2010//. 4:180	Level 1, Form Title/abstract screen
1691	**Gent L.C.,Garson J.K.,Dundon W.D.,Courtright L.E.,Pettinati H.M.,Oslin D.W.**. A comparison of CBI therapist and MM clinician ratings of the therapeutic alliance in the treatment of alcohol dependent clients. *Alcoholism: Clinical and Experimental Research*. 2010//. 34:226A	Level 2, Form Full Text Screening
1692	**Garson J.K.,Gent L.C.,Dundon W.D.,Courtright L.E.,Pettinati H.M.,Oslin D.W.**. Further evidence supporting the relationship of the therapeutic alliance and treatment outcome when utilizing medical-based interventions. *Alcoholism: Clinical and Experimental Research*. 2010//. 34:226A	Level 2, Form Full Text Screening
1693	**Tonigan J.S.,Rice S.L.**. The importance of early impressions about AA usefulness for later abstinence. *Alcoholism: Clinical and Experimental Research*. 2010//. 34:174A	Level 1, Form Title/abstract screen
1694	**Mahone I.,Mistler L.**. Shared decision making in medication use: An emerging best practice in mental health. *European Psychiatry*. 2010//. 25:#pages#	Level 1, Form Title/abstract screen
1695	**De Fruyt J.,Axters M.,Haspeslagh M.,Demyttenaere K.**. Decision making capacity in patients admitted to a psychiatric emergency service: A field trial. *European Psychiatry*. 2010//. 25:#pages#	Level 1, Form Title/abstract screen
1696	**Moczygemba L.,Matzke G.,Gatewood S.,Alexander A.,Kennedy A.,Osborn R.,Goode J.,Reynolds-Cane D.**. Development of a clinical pharmacy services provider network for homeless patients. *Journal of the American Pharmacists Association*. 2010//. 50:275	Level 1, Form Title/abstract screen
1697	**Gatewood S.,Alexander A.,Osborn R.,Kennedy A.,Goode J.,Matzke G.,Reynolds-Cane D.**. Expansion of clinical pharmacy services in an underserved population through an academic-community partnership. *Journal of the American Pharmacists Association*. 2010//. 50:268	Level 1, Form Title/abstract screen
1698	**Chartier F.,Rouillon F.,Lukasiewicz M.,Kraemer S.,D'yachkova Y.**. Effectiveness of olanzapine standard oral and orally disintegrating tablets in a 1-year European observational study. *European Neuropsychopharmacology*. 2010//. 20:S508	Level 1, Form Title/abstract screen
1699	**Richardson D.F.,Adamson S.J.,Deering D.E.A.**. The role of therapeutic alliance in treatment for people with mild to moderate alochol dependence. *Alcoholism: Clinical and Experimental Research*. 2010//. 34:169A	Level 2, Form Full Text Screening
1701	**Kilbourne A.M.,Biswas K.,Pirraglia P.A.,Sajatovic M.,Williford W.O.,Bauer M.S.**. Is the collaborative chronic care model effective for patients with bipolar disorder and co-occurring conditions?. *Journal of Affective Disorders*. 2009//. 112:256	Level 1, Form Title/abstract screen
1707	**Bass C.,Jones D.P.H.**. Fabricated or induced illness: assessment of perpetrators and approaches to management. *Psychiatry*. 2009//. 8:158	Level 1, Form Title/abstract screen
1710	**Joe G.W.,Simpson D.D.,Rowan-Szal G.A.**. Interaction of counseling rapport and topics discussed in sessions with methadone treatment clients. *Substance Use and Misuse*. 2009//. 44:3	Level 2, Form Full Text Screening
1711	**Phan O.,Lascaux M.**. Motivational interviews with cannabis dependant adolescents. *Annales Medico-Psychologiques*. 2009//. 167:523	Level 2, Form Full Text Screening

1713	**Salous A.,Omar H.A.**. Substance abuse among adolescents: A cross-cultural review. *International Journal on Disability and Human Development*. 2009//. 8:155	Level 1, Form Title/abstract screen
1715	**Anstiss T.**. Motivational interviewing in primary care. *Journal of Clinical Psychology in Medical Settings*. 2009//. 16:87	Level 1, Form Title/abstract screen
1718	**Vannoy S.D.,Arean P.,Unutzer J.D.**. The use of depression free days for tracking late-life depression outcomes. *American Journal of Geriatric Psychiatry*. 2009//. 17:A98	Level 1, Form Title/abstract screen
1719	**Chandler G.M.,Cowperthwait C.M.,Hay A.C.,Sylvia L.G.,Ostacher M.J.,Nierenberg A.A.,Perlis R.H.,Sachs G.S.**. Anxiety or irritability can lead to misdiagnosis of bipolar disorder: Results from systematic consultations at the MGH collaborative care initiative (CCI) consultation service. *Bipolar Disorders*. 2009//. 11:28	Level 1, Form Title/abstract screen
1720	**Houck J.M.,Manuel J.K.,Pyeatt C.J.,Moyers T.B.,Montoya V.L.**. A new scale to assess barriers to adopting motivational interviewing. *Alcoholism: Clinical and Experimental Research*. 2009//. 33:137A	Level 1, Form Title/abstract screen
1721	**Newman A.,Beckstead S.,Finch S.,Knorr T.,Lynch C.,MacKenzie M.,Robertson R.,Shore R.**. Hepatitis C treatment of a marginalized population: A multi-disciplinary primary practitioner-led treatment model in practice. *Canadian Journal of Gastroenterology*. 2009//. 23:#pages#	Level 1, Form Title/abstract screen
1722	**Martins M.,Castro L.C.**. Comorbid borderline personality disorder and alcohol use disorder: A case of combined psycotherapeutic and pharmocotherapeutic interventions. *European Psychiatry*. 2009//. 24:S407	Level 2, Form Full Text Screening
1741	**Stergiopoulos V.,Dewa C.S.,Rouleau K.,Yoder S.,Chau N.**. Collaborative mental health care for the homeless: The role of psychiatry in positive housing and mental health outcomes. *Canadian Journal of Psychiatry*. 2008//. 53:61	Level 1, Form Title/abstract screen
1743	**Yohannes A.M.**. Management of anxiety and depression in patients with COPD. *Expert Review of Respiratory Medicine*. 2008//. 2:337	Level 1, Form Title/abstract screen
1745	**Trinh N.-H.,Moore D.,Brendel D.H.**. Ethics consultation to PACT teams: Balancing client autonomy and clinical necessity. *Harvard Review of Psychiatry*. 2008//. 16:365	Level 1, Form Title/abstract screen
1746	**Davis P.,Abou-Saleh M.T.**. Developing an enhanced counseling intervention for the primary prevention of hepatitis C among injecting drug users. *Addictive Disorders and their Treatment*. 2008//. 7:65	Level 1, Form Title/abstract screen
1747	**Holmes-Rovner M.,Stommel M.,Corser W.D.,Olomu A.,Holtrop J.S.,Siddiqi A.,Dunn S.L.**. Does outpatient telephone coaching add to hospital quality improvement following hospitalization for acute coronary syndrome?. *Journal of General Internal Medicine*. 2008//. 23:1464	Level 1, Form Title/abstract screen
1751	**Singh A.N.,Strand P.S.**. A person-centered, strength-based treatment of aggression and sexually inappropriate behavior in mental health. *Clinical Case Studies*. 2008//. 7:397	Level 1, Form Title/abstract screen
1753	**Barber J.P.,Gallop R.,Crits-Christoph P.,Barrett M.S.,Klostermann S.,McCarthy K.S.,Sharpless B.A.**. THE ROLE OF THE ALLIANCE AND TECHNIQUES IN PREDICTING OUTCOME OF SUPPORTIVE-EXPRESSIVE DYNAMIC THERAPY FOR COCAINE DEPENDENCE. *Psychoanalytic Psychology*. 2008//. 25:461	Level 2, Form Full Text Screening
1755	**Bonsack C.,Montagrin Y.,Gibellini S.,Favrod J.,Besson J.,Conus P.**.	Level 2, Form

	Practice of a motivational intervention for cannabis users with psychosis. *Schweizer Archiv fur Neurologie und Psychiatrie*. 2008//. 159:378	Full Text Screening
1756	**Haskard K.B.,Williams S.L.,DiMatteo M.R.,Rosenthal R.,White M.K.,Goldstein M.G.**. Physician and Patient Communication Training in Primary Care: Effects on Participation and Satisfaction. *Health Psychology*. 2008//. 27:513	Level 1, Form Title/abstract screen
1757	**Henderson D.**. Improving adherence: How far have we come?. *European Neuropsychopharmacology*. 2008//. 18:S590	Level 1, Form Title/abstract screen
1762	**Ambresin G.,de Roten Y.,Drapeau M.,Despland J.-N.**. Early change in maladaptive defence style and development of therapeutic alliance. *Clinical Psychology and Psychotherapy*. 2007//. 14:89	Level 1, Form Title/abstract screen
1766	**Angell B.,Martinez N.I.,Mahoney C.A.,Corrigan P.W.**. Payeeship, financial leverage, and the client-provider relationship. *Psychiatric Services*. 2007//. 58:365	Level 1, Form Title/abstract screen
1770	**Mendez V. J.C.,Maluenda M. R.,Loo T. X.,Isla P. R.,Anza A.**. Therapeutic alliance in addict patients of two institutions of Antofagasta. *Revista Chilena de Neuro-Psiquiatria*. 2007//. 45:141	Level 1, Form Title/abstract screen
1772	**Bricker J.B.,Russo J.,Stein M.B.,Sherbourne C.,Craske M.,Schraufnagel T.J.,Roy-Byrne P.**. Does occasional cannabis use impact anxiety and depression treatment outcomes? Results from a randomized effectiveness trial. *Depression and Anxiety*. 2007//. 24:392	Level 1, Form Title/abstract screen
1773	**Stawicki S.,Krampe H.,Niehaus S.,Ribbe K.,Wagner T.,Bartels C.,Kroner-Herwig B.,Ehrenreich H.**. Multimodal monitoring of psychotherapeutic processes in the treatment of alcohol dependent patients. *Suchtmedizin in Forschung und Praxis*. 2007//. 9:27	Level 2, Form Full Text Screening
1774	**Anonymous.**. Part A: Treatment recommendations for patients with substance use disorders. *American Journal of Psychiatry*. 2007//. 164:5	Level 2, Form Full Text Screening
1776	**Wiederholt P.A.,Connor N.P.,Hartig G.K.,Harari P.M.**. Bridging Gaps in Multidisciplinary Head and Neck Cancer Care: Nursing Coordination and Case Management. *International Journal of Radiation Oncology Biology Physics*. 2007//. 69:S88	Level 1, Form Title/abstract screen
1777	**Huguelet P.**. Recovery as an organising principle for the care of patients with severe mental disorders. *Schweizer Archiv fur Neurologie und Psychiatrie*. 2007//. 158:271	Level 1, Form Title/abstract screen
1780	**Teplin D.**. Substance dependence and personality disorders: What's the deal?. *Journal of Opioid Management*. 2007//. 3:242	Level 1, Form Title/abstract screen
1783	**Mann B.J.,Grana W.A.,Indelicato P.A.,O'Neill D.F.,George S.Z.**. A survey of sports medicine physicians regarding psychological issues in patient-athletes. *American Journal of Sports Medicine*. 2007//. 35:2140	Level 1, Form Title/abstract screen
1785	**Rastogi M.,Wadhwa S.**. Substance abuse among Asian Indians in the United States: A consideration of cultural factors in etiology and treatment. *Substance Use and Misuse*. 2006//. 41:1239	Level 1, Form Title/abstract screen
1790	**Swann A.C.**. Long-term treatment in bipolar disorder. *Journal of Clinical Psychiatry*. 2005//. 66:7	Level 1, Form Title/abstract screen

1791	**Hensley R.D.,Jones A.K.,Williams A.G.,Willsher L.B.,Cain P.P.**. One-year clinical outcomes for Louisiana residents diagnosed with type 2 diabetes and hypertension. *Journal of the American Academy of Nurse Practitioners*. 2005//. 17:363	Level 1, Form Title/abstract screen
1793	**Kobin C.,Tyson E.**. Thematic analysis of hip-hop music: Can hip-hop in therapy facilitate empathic connections when working with clients in urban settings?. *Arts in Psychotherapy*. 2006//. 33:343	Level 1, Form Title/abstract screen
1796	**Pomm H.A.**. Regaining balance after "reality vertigo:" Teaching learners to attend to the psychological aspects of patients with chronic, nonmalignant pain. *Family Medicine*. 2006//. 38:86	Level 1, Form Title/abstract screen
1797	**Meier P.S.,Donmall M.C.**. Differences in client and therapist views of the working alliance in drug treatment. *Journal of Substance Use*. 2006//. 11:73	Level 2, Form Full Text Screening
1798	**Onate J.**. Psychiatric consultation in outpatient primary care settings: Should consultation change to collaboration?. *Primary Psychiatry*. 2006//. 13:41	Level 1, Form Title/abstract screen
1799	**Stoddard F.J.,Usher C.T.,Abrams A.N.**. Psychopharmacology in Pediatric Critical Care. *Child and Adolescent Psychiatric Clinics of North America*. 2006//. 15:611	Level 1, Form Title/abstract screen
1806	**De Lucas Taracena Ma.T.,Montanes Rada F.**. Attachment styles and representations in drug users. *Adicciones*. 2006//. 18:377	Level 1, Form Title/abstract screen
1809	**Jones S.,Burrell-Hodgson G.,Tate G.,Fowler B.**. Personality disorder in primary care: Factors associated with therapist views of process and outcome. *Behavioural and Cognitive Psychotherapy*. 2006//. 34:453	Level 1, Form Title/abstract screen
1810	**Holmes A.,Hodge M.,Lenten S.,Fielding J.,Castle D.,Velakoulis D.,Bradley G.**. Chronic mental illness and community treatment resistance. *Australasian Psychiatry*. 2006//. 14:272	Level 1, Form Title/abstract screen
1813	**Brook M.,Hilty D.M.,Liu W.,Hu R.,Frye M.A.**. Discharge against medical advice from inpatient psychiatric treatment: A literature review. *Psychiatric Services*. 2006//. 57:1192	Level 1, Form Title/abstract screen
1814	**Gifford E.V.,Ritsher J.B.,McKellar J.D.,Moos R.H.**. Acceptance and relationship context: A model of substance use disorder treatment outcome. *Addiction*. 2006//. 101:1167	Level 2, Form Full Text Screening
1816	**Redfern J.M.,Mckevitt C.,Wolfe C.D.A.**. Risk management after stroke: The limits of a patient-centred approach. *Health, Risk and Society*. 2006//. 8:123	Level 1, Form Title/abstract screen
1820	**Kisely S.,Duerden D.,Shaddick S.,Jayabarathan A.**. Collaboration between primary care and psychiatric services: Does it help family physicians?. *Canadian Family Physician*. 2006//. 52:876	Level 1, Form Title/abstract screen
1821	**Hu L.-X.,Wu J.-L.,Feng J.,Hou Y.-M.**. Ameliorative effect of client-centered therapy in combination with individual and group interventions on the psychological health status in members of drugs rehabilitation through labor. *Chinese Journal of Clinical Rehabilitation*. 2006//. 10:72	Level 1, Form Title/abstract screen
1822	**Barber J.P.,Gallop R.,Crits-Christoph P.,Frank A.,Thase M.E.,Weiss R.D.,Connolly Gibbons M.B.**. The role of therapist adherence, therapist competence, and alliance in predicting outcome of individual drug counseling: Results from the National Institute Drug Abuse Collaborative Cocaine Treatment Study. *Psychotherapy Research*. 2006//. 16:229	Level 2, Form Full Text Screening

1826	**Piccoli G.B.,Soragna G.,Rossetti M.,Putaggio S.,Perrotta L.,Bonetto A.,Magnano A.,Vercellone F.**. Non-compliance after a kidney-pancreas transplantation - A narrative case-analysis involving different patient-physician relationships and ethical frames. *Transplantationsmedizin: Organ der Deutschen Transplantationsgesellschaft*. 2005//. 17:107	Level 1, Form Title/abstract screen
1827	**Galluzzi K.E.**. Editor's message: Patient-centered care for chronic nonmalignant pain. *Journal of the American Osteopathic Association*. 2005//. 105:#pages#	Level 1, Form Title/abstract screen
1828	**Roy-Byrne P.,Stein M.B.,Russo J.,Craske M.,Katon W.,Sullivan G.,Sherbourne C.**. Medical illness and response to treatment in primary care panic disorder. *General Hospital Psychiatry*. 2005//. 27:237	Level 1, Form Title/abstract screen
1830	**Rumpold G.,Doering S.,Smrekar U.,Schubert C.,Koza R.,Schatz D.S.,Bertl-Schuessler A.,Janecke N.,Lampe A.,Schuessler G.**. Changes in motivation and the therapeutic alliance during a pretherapy diagnostic and motivation-enhancing phase among psychotherapy outpatients. *Psychotherapy Research*. 2005//. 15:117	Level 1, Form Title/abstract screen
1831	**Faw L.,Hogue A.,Johnson S.,Diamond G.M.,Liddle H.A.**. The Adolescent Therapeutic Alliance Scale (ATAS): Initial psychometrics and prediction of outcome in family-based substance abuse prevention counseling. *Psychotherapy Research*. 2005//. 15:141	Level 1, Form Title/abstract screen
1836	**Nielsen A.S.**. Factors influencing the therapeutic alliance and treatment compliance: The individual experience. *NAD Publication*. 2005//. #volume#:187	Level 1, Form Title/abstract screen
1837	**Park E.R.,Wolfe T.J.,Gokhale M.,Winickoff J.P.,Rigotti N.A.**. Perceived preparedness to provide preventive counseling reports of graduating primary care residents at academic health centers. *Journal of General Internal Medicine*. 2005//. 20:386	Level 1, Form Title/abstract screen
1840	**Jensen P.S.,Weersing R.,Hoagwood K.E.,Goldman E.**. What is the evidence for evidence-based treatments? A hard look at our soft underbelly. *Mental Health Services Research*. 2005//. 7:53	Level 1, Form Title/abstract screen
1841	**Leichsenring F.**. Are psychodynamic and psychoanalytic therapies effective? A review of empirical data. *International Journal of Psychoanalysis*. 2005//. 86:841	Level 1, Form Title/abstract screen
1842	**Blaise M.**. From hand to hand: Reflections on the prescription and dispensing of medication at the moment of consultation as a tool against drug addiction. *Information Psychiatrique*. 2005//. 81:429	Level 1, Form Title/abstract screen
1848	**Powers T.A.,Alonso A.**. Dynamic psychotherapy and the problem of time. *Journal of Contemporary Psychotherapy*. 2004//. 34:125	Level 1, Form Title/abstract screen
1849	**Goforth H.W.,Lupash D.P.,Brown M.E.,Tan J.,Fernandez F.**. Role of alcohol and substances of abuse in the immunomodulation of human immunodeficiency virus disease: A review. *Addictive Disorders and their Treatment*. 2004//. 3:174	Level 1, Form Title/abstract screen
1855	**Moretti F.,Goss C.,Del Piccolo L.**. Communication strategies to motivate patients to follow doctor's advice. *Recenti Progressi in Medicina*. 2004//. 95:61	Level 1, Form Title/abstract screen
1863	**Galloway V.A.,Brodsky S.L.**. Caring less, doing more: The role of therapeutic detachment with volatile and unmotivated clients. *American Journal of Psychotherapy*. 2003//. 57:32	Level 1, Form Title/abstract screen

1864	**Demmel R.,Rist F.,Hagen J.,Aulhorn I.,Scheuren B.,Scherbaum N.,Gesenhues S.,Rollnick S.**. Secondary prevention beyond screening and brief advice. *Suchtmedizin in Forschung und Praxis*. 2003//. 5:33	Level 1, Form Title/abstract screen
1866	**Oetzel K.B.,Scherer D.G.**. Therapeutic engagement with adolescents in psychotherapy. *Psychotherapy*. 2003//. 40:215	Level 1, Form Title/abstract screen
1867	**Jessup M.A.,Humphreys J.C.,Brindis C.D.,Lee K.A.**. Extrinsic barriers to substance abuse treatment among pregnant drug dependent women. *Journal of Drug Issues*. 2003//. 33:285	Level 1, Form Title/abstract screen
1868	**Mills P.D.,Harvey P.W.**. Beyond community-based diabetes management and the COAG coordinated care trial. *Australian Journal of Rural Health*. 2003//. 11:131	Level 1, Form Title/abstract screen
1870	**Fenton W.S.**. Shared decision making: A model for the physician-patient relationship in the 21st century?. *Acta Psychiatrica Scandinavica*. 2003//. 107:401	Level 1, Form Title/abstract screen
1874	**Barber J.P.,Liese B.S.,Abrams M.J.**. Development of the cognitive therapy adherence and competence scale. *Psychotherapy Research*. 2003//. 13:205	Level 1, Form Title/abstract screen
1884	**Walters G.D.**. Lessons learned from project MATCH. *Addictive Disorders and their Treatment*. 2002//. 1:135	Level 1, Form Title/abstract screen
1885	**Milbrodt T.**. Breaking the cycle of alcohol problems among native Americans: Culturally-sensitive treatment in the Lakota community. *Alcoholism Treatment Quarterly*. 2002//. 20:19	Level 2, Form Full Text Screening
1888	**Mallow A.J.**. To sink or swim: The use of dream material in addiction treatment. *Journal of Substance Use*. 2002//. 7:157	Level 1, Form Title/abstract screen
1889	**Santiago N.J.,Klein D.N.,Vivian D.,Vocisano C.,Dowling F.,Arnow B.A.,Manber R.,Markowitz J.C.,McCullough Jr. J.P.,Riso L.P.L.,Rothbaum B.O.,Rush A.J.,Thase M.E.,Keller M.B.**. Pretreatment correlates of the therapeutic alliance in the chronically depressed. *Journal of Contemporary Psychotherapy*. 2002//. 32:281	Level 1, Form Title/abstract screen
1893	**Teusch L.,Bohme H.,Finke J.,Gastpar M.**. Effects of client-centered psychotherapy for personality disorders alone and in combination with psychopharmacological treatment: An empirical follow-up study. *Psychotherapy and Psychosomatics*. 2001//. 70:328	Level 1, Form Title/abstract screen
1894	**Cecero J.J.,Fenton L.R.,Nich C.,Frankforter T.L.,Carroll K.M.**. Focus on therapeutic alliance: The psychometric properties of six measures across three treatments. *Psychotherapy*. 2001//. 38:1	Level 1, Form Title/abstract screen
1898	**Waldstein S.R.,Neumann S.A.,Drossman D.A.,Novack D.H.**. Teaching psychosomatic (biopsychosocial) medicine in United States medical schools: Survey findings. *Psychosomatic Medicine*. 2001//. 63:335	Level 1, Form Title/abstract screen
1902	**Martin D.J.,Garske J.P.,Katherine Davis M.**. Relation of the therapeutic alliance with outcome and other variables: A meta-analytic review. *Journal of Consulting and Clinical Psychology*. 2000//. 68:438	Level 1, Form Title/abstract screen
1911	**Rihmer Z.,Pestality P.**. Bipolar II disorder and suicidal behavior. *Psychiatric Clinics of North America*. 1999//. 22:667	Level 1, Form Title/abstract screen

1915	**Guinjoan S.M.,Ross D.R.**. The use of metaphors by the 'Ambulatory inpatients' of the managed care era. *American Journal of Psychotherapy*. 1999//. 53:188	Level 1, Form Title/abstract screen
1916	**Schwartz B.**. Preventing the spread of antimicrobial resistance among bacterial respiratory pathogens in industrialized countries: The case for judicious antimicrobial use. *Clinical Infectious Diseases*. 1999//. 28:211	Level 1, Form Title/abstract screen
1918	**Gordon J.R.**. Harm reduction psychotherapy comes out of the closet. *In Session - Psychotherapy in Practice*. 1998//. 4:69	Level 1, Form Title/abstract screen
1931	**Cadilhac P.,Schmitt L.,Sztulman H.,Moron P.,Reinert M.**. Assessment of the interpersonal psychotherapy's effect during opioid detoxification: Statistical analysis of the textual data. *Annales Medico-Psychologiques*. 1996//. 154:382	Level 1, Form Title/abstract screen
1933	**Varma S.C.,Siris S.G.**. Alcohol abuse in Asian Americans. Epidemiological and treatment issues. *American Journal on Addictions*. 1996//. 5:136	Level 1, Form Title/abstract screen
1935	**Bremner A.J.**. The therapeutic alliance in severe mental disorder. *Therapeutic Communities: the International Journal for Therapeutic and Supportive Organizations*. 1995//. 16:193	Level 1, Form Title/abstract screen
1937	**Najavits L.M.,Luborsky L.,Frank A.,Weiss R.D.,Liese B.S.,Thompson H.,Nakayama E.,Siqueland L.,Daley D.,Onken L.S.**. Therapists' emotional reactions to substance abusers: A new questionnaire and initial findings. *Psychotherapy*. 1995//. 32:669	Level 1, Form Title/abstract screen
1940	**Kalir H.H.**. Psychiatric primary care. *Clinical Consultations in Obstetrics and Gynecology*. 1995//. 7:27	Level 1, Form Title/abstract screen
1941	**MacKinney T.G.,Walters D.,Bird G.L.,Nattinger A.B.**. Improvements in preventive care and communication for deaf patients: Results of a novel primary health care program. *Journal of General Internal Medicine*. 1995//. 10:133	Level 1, Form Title/abstract screen
1948	**Goldblatt M.J.**. Hospitalization of the suicidal patient. *Death Studies*. 1994//. 18:453	Level 1, Form Title/abstract screen
1949	**Aapro N.,Dazord A.,Gerin P.,De Coulon N.,Scariati G.,Lastrico A.,Andreoli A.**. Follow-up of patients receiving psychotherapy at an institution in Geneva. *Psychotherapies*. 1994//. 14:183	Level 1, Form Title/abstract screen
1953	**Rockland L.H.**. A review of supportive psychotherapy, 1986-1992. *Hospital and Community Psychiatry*. 1993//. 44:1053	Level 1, Form Title/abstract screen
1956	**Southwick S.M.,Satel S.L.**. Exploring the meanings of substance abuse: An important dimension of early work with borderline patients. *American Journal of Psychotherapy*. 1990//. 44:61	Level 1, Form Title/abstract screen
1957	**German P.S.,Burton L.C.**. Clinicians, the elderly and drugs. *Journal of Drug Issues*. 1989//. 19:221	Level 1, Form Title/abstract screen
1958	**Hambrecht M.**. Relapse in psychotherapy. *PPmP Psychotherapie Psychosomatik Medizinische Psychologie*. 1988//. 38:425	Level 1, Form Title/abstract screen
1959	**Levine D.M.**. The physician's role in health-promotion and disease prevention. *Bulletin of the New York Academy of Medicine: Journal of Urban Health*.	Level 1, Form Title/abstract

	1987//. 63:950	screen
1961	**Feselmayer S.,Heinzl K.**. Client-centered psychotherapy with addicts. *Wiener Zeitschrift fur Suchtforschung*. 1985//. 8:39	Level 2, Form Full Text Screening
1963	**Windsor R.A.,Green L.W.,Roseman J.M.**. Health promotion and maintenance for patients with chronic obstructive pulmonary disease: A review. *Journal of Chronic Diseases*. 1980//. 33:5	Level 1, Form Title/abstract screen
1966	**Mansell Pattison E.**. The Jack Donovan memorial lecture-1978: Differential approaches to multiple problems associated with alcoholism. *Contemporary Drug Problems*. 1978//. 7:265	Level 1, Form Title/abstract screen
1968	**Flynn W.R.**. Drug abuse as a defense in adolescence: a follow up. *Adolescence*. 1973//. 8:363	Level 1, Form Title/abstract screen
1972	**Deirdre O'Sullivan, Justin Watts, Chad Shenk**. Child maltreatment severity, chronic substance abuse, and disability status.. *Rehabilitation Psychology*. 2018/05//. 63:313	Level 1, Form Title/abstract screen
1974	**Björn Philips, Roger Karlsson, Rebecca Nygren, Amelie Rother-Schirren, Andrzej Werbart**. Early therapeutic process related to dropout in mentalization-based treatment with dual diagnosis patients.. *Psychoanalytic Psychology*. 2018/04//. 35:205	Level 2, Form Full Text Screening
1975	**Jocelyn C. Anderson, Jacquelyn C. Campbell, Nancy E. Glass, Michele R. Decker, Nancy Perrin, Jason Farley**. Impact of intimate partner violence on clinic attendance, viral suppression and CD4 cell count of women living with HIV in an urban clinic setting.. *AIDS Care*. 2018/04//. 30:399	Level 1, Form Title/abstract screen
1977	**Jennifer J. Esala, Maria M. Vukovich, Ashley Hanbury, Shraddha Kashyap, Amy Joscelyne**. Collaborative care for refugees and torture survivors: Key findings from the literature.. *Traumatology*. 2018/03/08/. #volume#:#pages#	Level 1, Form Title/abstract screen
1978	**P. Kushalnagar, A. Engleman, G. Sadler**. Deaf patient-provider communication and lung cancer screening: Health information national trends survey in american sign language (hints-asl).. *Patient Education and Counseling*. 2018/03/05/. #volume#:#pages#	Level 1, Form Title/abstract screen
1979	**Annett Lotzin, Sven Buth, Susanne Sehner, Philipp Hiller, Marcus-Sebastian Martens, Silke Pawils, Franka Metzner, John Read, Martin Härter, Ingo Schäfer**. 'Learning how to ask': Effectiveness of a training for trauma inquiry and response in substance use disorder healthcare professionals.. *Psychological Trauma: Theory, Research, Practice, and Policy*. 2018/03//. 10:229	Level 1, Form Title/abstract screen
1982	**Catherine DeCarlo Santiago, Tali Raviv, Lisa H. Jaycox**. Universal and schoolwide interventions for trauma.. *Creating healing school communities: School-based interventions for students exposed to trauma.*. 2018///. #volume#:37	Level 1, Form Title/abstract screen
1983	**Randi Sokol, Chiara Albanese, Deviney Chaponis, Jessica Early, George Maxted, Diana Morrill, Grace Poirier, Fran Puopolo, Zev Schuman-Olivier**. Why use group visits for opioid use disorder treatment in primary care? A patient-centered qualitative study.. *Substance Abuse*. 2018/01//. 39:52	Level 1, Form Title/abstract screen
1984	**Charles Geier, Nicole Roberts, David Lydon-Staley**. The effects of smoking abstinence on incentivized spatial working memory.. *Substance Use & Misuse*. 2018/01//. 53:86	Level 1, Form Title/abstract screen

1985	**April Joy Damian, Joseph J. Gallo, Tamar Mendelson**. Barriers and facilitators for access to mental health services by traumatized youth.. *Children and Youth Services Review*. 2018/01//. 85:273	Level 1, Form Title/abstract screen
1987	**Lortiz I. Babilonia**. Integrating person-centered therapy with rational emotive behavioral therapy to treatment alcohol use disorder in Hispanic armed forces members.. *Dissertation Abstracts International: Section B: The Sciences and Engineering*. 2018///. 78:#pages#	Level 2, Form Full Text Screening
1988	**Jessica Ann Brown**. A relational investigation of the therapeutic alliance in substance abuse treatment: A qualitative inquiry.. *Dissertation Abstracts International: Section B: The Sciences and Engineering*. 2018///. 78:#pages#	Level 2, Form Full Text Screening
1989	**Jennifer J. Buckley**. Exploring the role of trauma in women's substance use disorder treatment: A naturalistic study.. *Dissertation Abstracts International: Section B: The Sciences and Engineering*. 2018///. 78:#pages#	Level 2, Form Full Text Screening
1990	**Laura Eidlitz**. Therapeutic alliance and age of trauma onset in treatment of Comorbid Posttraumatic Stress Disorder and Alcohol Use Disorder.. *Dissertation Abstracts International: Section B: The Sciences and Engineering*. 2018///. 79:#pages#	Level 2, Form Full Text Screening
1991	**Faith May Golden**. Lived experiences and coping styles of Alaskan women with opioid use disorders.. *Dissertation Abstracts International: Section B: The Sciences and Engineering*. 2018///. 78:#pages#	Level 1, Form Title/abstract screen
1992	**Maya Hodgdon**. Impact of voices curriculum on adolescent mothers.. *Dissertation Abstracts International: Section B: The Sciences and Engineering*. 2018///. 78:#pages#	Level 1, Form Title/abstract screen
1993	**Alexandra H. Kutnick**. Understanding adaptation to an HIV diagnosis in the context of urban poverty: A qualitative examination.. *Dissertation Abstracts International: Section B: The Sciences and Engineering*. 2018///. 78:#pages#	Level 1, Form Title/abstract screen
1994	**Mebane E. Powell**. Impact of psychological maltreatment during childhood by one's maternal figure on the mental and physical health of older adult men.. *Dissertation Abstracts International Section A: Humanities and Social Sciences*. 2018///. 79:#pages#	Level 1, Form Title/abstract screen
1996	**Alberta S. J. van der Watt, Gareth Nortje, Lola Kola, John Appiah-Poku, Caleb Othieno, Benjamin Harris, Bibilola D. Oladeji, Oluyomi Esan, Victor Makanjuola, LeShawndra N. Price, Soraya Seedat, Oye Gureje**. Collaboration between biomedical and complementary and alternative care providers: Barriers and pathways.. *Qualitative Health Research*. 2017/12//. 27:2177	Level 1, Form Title/abstract screen
1997	**Yvonne C. M. Rensen, Jos I. M. Egger, Josette Westhoff, Serge J. W. Walvoort, Roy P. C. Kessels**. The effect of errorless learning on quality of life in patients with Korsakoff's syndrome.. *Neuropsychiatric Disease and Treatment*. 2017/11/27/. 13:#pages#	Level 1, Form Title/abstract screen
2003	**Emily Gardiner, Grace Iarocci, Marlene Moretti**. Integrative care for adolescents with dual diagnosis: Considering trauma and attachment within an innovative model for clinical practice.. *Journal of Mental Health Research in Intellectual Disabilities*. 2017/10//. 10:321	Level 1, Form Title/abstract screen
2005	**Pablo Barrio, Lluisa Ortega, Hugo López, Antoni Gual**. Self-management and shared decision-making in alcohol dependence via a mobile app: A pilot study.. *International Journal of Behavioral Medicine*. 2017/10//. 24:722	Level 2, Form Full Text Screening
2007	**Lesia M. Ruglass, Alina Shevorykin, Christina Brezing, Mei-Chen Hu, Denise A. Hien**. Demographic and clinical characteristics of treatment seeking	Level 1, Form Title/abstract

	women with full and subthreshold PTSD and concurrent cannabis and cocaine use disorders.. *Journal of Substance Abuse Treatment*. 2017/09//. 80:45	screen
2008	**Bethany M. Kwan, Sanjay Chadha, Mika K. Hamer, David Spagnolo, Sheila Kee**. Mixed methods evaluation of a collaborative care implementation using RE-AIM.. *Families, Systems, & Health*. 2017/09//. 35:295	Level 1, Form Title/abstract screen
2009	**Andy Guise, Maureen Seguin, Gitau Mburu, Susie McLean, Pippa Grenfell, Zahed Islam, Sergii Filippovych, Happy Assan, Andrea Low, Peter Vickerman, Tim Rhodes**. Integrated opioid substitution therapy and HIV care: A qualitative systematic review and synthesis of client and provider experiences.. *AIDS Care*. 2017/09//. 29:1119	Level 2, Form Full Text Screening
2010	**Eileen Klein**. Using social support for LGBTQ clients with mental illness to be out of the closet, in treatment, and in the community.. *Journal of Gay & Lesbian Social Services: The Quarterly Journal of Community & Clinical Practice*. 2017/07//. 29:221	Level 1, Form Title/abstract screen
2012	**Eugenia Oviedo-Joekes, Kirsten Marchand, Heather Palis, Daphne Guh, Suzanne Brissette, Kurt Lock, Scott MacDonald, Scott Harrison, Aslam H. Anis, Michael Krausz, David C. Marsh, Martin T. Schechter**. Predictors of treatment allocation guesses in a randomized controlled trial testing double-blind injectable hydromorphone and diacetylmorphine for severe opioid use disorder.. *Addiction Research & Theory*. 2017/07//. 25:263	Level 1, Form Title/abstract screen
2013	**James F. Boswell, Nicole M. Cain, Jennifer M. Oswald, Andrew A. McAleavey, Robert Adelman**. Interpersonal pathoplasticity and trajectories of change in routine adolescent and young adult residential substance abuse treatment.. *Journal of Consulting and Clinical Psychology*. 2017/07//. 85:676	Level 1, Form Title/abstract screen
2015	**Laurel A. Mattos, Adam T. Schmidt, Craig E. Henderson, Aaron Hogue**. Therapeutic alliance and treatment outcome in the outpatient treatment of urban adolescents: The role of callous–unemotional traits.. *Psychotherapy*. 2017/06//. 54:136	Level 1, Form Title/abstract screen
2016	**Elena Argento, Steffanie A. Strathdee, Shira Goldenberg, Melissa Braschel, Julio Montaner, Kate Shannon**. Violence, trauma and living with HIV: Longitudinal predictors of initiating crystal methamphetamine injection among sex workers.. *Drug and Alcohol Dependence*. 2017/06/01/. 175:198	Level 1, Form Title/abstract screen
2018	**Laurence Rigaud, Sabine de Bazelaire, Lucia Romo**. Utilisation d'outils numériques en vue du traitement de l'addiction au cannabis d'un public francophone: Synthèse de la recherche. = Use of electronic tools to treat cannabis addition in a francophone population: A research overview.. *Alcoologie et Addictologie*. 2017/06//. 39:41S	Level 1, Form Title/abstract screen
2021	**Aymeric Reyre, Raphaël Jeannin, Myriam Largueche, Marie Rose Moro, Thierry Baubet, Olivier Taieb**. Overcoming professionals' challenging experiences to promote a trustful therapeutic alliance in addiction treatment: A qualitative study.. *Drug and Alcohol Dependence*. 2017/05/01/. 174:30	Level 2, Form Full Text Screening
2022	**Audrey L. Jones, Leslie R. M. Hausmann, Gretchen L. Haas, Maria K. Mor, John P. Cashy, James H. Jr. Schaefer, Adam J. Gordon**. A national evaluation of homeless and nonhomeless veterans' experiences with primary care.. *Psychological Services*. 2017/05//. 14:174	Level 1, Form Title/abstract screen
2023	**Marco Antonio Nocito Echevarria, Tassio Andrade Reis, Giulianno Ruffo Capatti, Victor Siciliano Soares, Dartiu Xavier da Silveira, Thiago Marques Fidalgo**. N-acetylcysteine for treating cocaine addiction—A systematic review.. *Psychiatry Research*. 2017/05//. 251:197	Level 1, Form Title/abstract screen
2024	**Cathryn Glanton Holzhauer, Stephanie A. Gamble**. Depressive symptoms	Level 1, Form

	mediate the relationship between changes in emotion regulation during treatment and abstinence among women with alcohol use disorders.. *Psychology of Addictive Behaviors*. 2017/05//. 31:284	Title/abstract screen
2027	**Suzanne Carlberg-Racich, Lindsey Roden**. Barriers to patient-centered human immunodeficiency virus (HIV) care with African American patients who use drugs: Social construction of the typical and ideal care visit.. *Substance Abuse*. 2017/04//. 38:205	Level 1, Form Title/abstract screen
2030	**Vincent Chin-Hung Chen, Hua Ting, Meng-Huan Wu, Tsang-Yaw Lin, Michael Gossop**. Sleep disturbance and its associations with severity of dependence, depression and quality of life among heroin-dependent patients: A cross-sectional descriptive study.. *Substance Abuse Treatment, Prevention, and Policy*. 2017/03/20/. 12:#pages#	Level 1, Form Title/abstract screen
2032	**Kristen Clements-Nolle, Sandra Larson, Aliya Buttar, Lindsey Dermid-Gray**. Childhood maltreatment and unprotected sex among female juvenile offenders: Evidence of mediation by substance abuse and psychological distress.. *Women's Health Issues*. 2017/03//Mar-Apr, 2017. 27:188	Level 1, Form Title/abstract screen
2037	**Carrisa S. Hoelscher, Michael W. Kramer, Christopher Nguyen, Olivia D. Cooper, Eric Anthony Day**. Decision making and communication in a statewide interagency task force: An investigation of planned versus utilized processes.. *Management Communication Quarterly*. 2017/02//. 31:39	Level 1, Form Title/abstract screen
2038	**Nhat Huynh, Natalie Arabian, Anna Naito, Stan Louie, Michael W. Jakowec, Liana Asatryan, Daryl L. Davies**. Preclinical development of moxidectin as a novel therapeutic for alcohol use disorder.. *Neuropharmacology*. 2017/02//. 113:60	Level 1, Form Title/abstract screen
2039	**Elias M. Klemperer, John R. Hughes, Peter W. Callas, Laura J. Solomon**. Working alliance and empathy as mediators of brief telephone counseling for cigarette smokers who are not ready to quit.. *Psychology of Addictive Behaviors*. 2017/02//. 31:130	Level 1, Form Title/abstract screen
2040	**Jennifer Pecina, Frederick North, Mark D. Williams, Kurt B. Angstman**. Use of an on-line patient portal in a depression collaborative care management program.. *Journal of Affective Disorders*. 2017/01/15/. 208:1	Level 1, Form Title/abstract screen
2041	**Lisa K. Kearney, Laura O. Wray, Katherine M. Dollar, Andrew S. Pomerantz**. Psychological assessment in the Veterans Health Administration (VHA) model of integrated primary care.. *Handbook of psychological assessment in primary care settings*.. 2017///. #volume#:709	Level 1, Form Title/abstract screen
2042	**Adam Silberstein, Lindsey Boone**. Multiple relationships in recovery communities.. *Multiple relationships in psychotherapy and counseling: Unavoidable, common, and mandatory dual relations in therapy*.. 2017///. #volume#:130	Level 1, Form Title/abstract screen
2043	**Elyse R. Park, Christina M. Luberto, Conall O'Cleirigh, Giselle K. Perez, Julianne G. Wilner**. Smoking cessation.. *The Massachusetts General Hospital handbook of behavioral medicine: A clinician's guide to evidence-based psychosocial interventions for individuals with medical illness*.. 2017///. #volume#:9	Level 1, Form Title/abstract screen
2044	**Mrigaya Sinha**. Shame and psychotherapy: Theory, method and practice.. *The value of shame: Exploring a health resource in cultural contexts*.. 2017///. #volume#:251	Level 1, Form Title/abstract screen
2049	**Jacquelyn H. Flaskerud**. Collaboration needed: Pain care and opioid abuse.. *Issues in Mental Health Nursing*. 2017/01//. 38:92	Level 1, Form Title/abstract screen

2051	**J. Valdes-Stauber**. Suizidales Verhalten aus anthropologischer Sicht: Dialektik zwischen dem medizinischen und dem philosophischen Paradigma. = Suicide, suicidal behaviour, anthropology, holistic view of human being, practical science, therapeutic relationship.. *Nervenheilkunde: Zeitschrift für interdisziplinaere Fortbildung*. 2017///. 36:251	Level 1, Form Title/abstract screen
2052	**Teresa Lyn Fresquez**. Effect of therapeutic alliance of clients on methadone maintenance treatment outcomes.. *Dissertation Abstracts International: Section B: The Sciences and Engineering*. 2017///. 78:#pages#	Level 2, Form Full Text Screening
2053	**Timofey S. Galuza**. Using big data in psychotherapy research: Possibilities and perils.. *Dissertation Abstracts International: Section B: The Sciences and Engineering*. 2017///. 78:#pages#	Level 1, Form Title/abstract screen
2054	**Melissa K. Rosenow**. Substance abuse and trauma in Latino adolescents: A workbook.. *Dissertation Abstracts International: Section B: The Sciences and Engineering*. 2017///. 77:#pages#	Level 2, Form Full Text Screening
2055	**Vikram Patel, Shuiyuan Xiao, Hanhui Chen, Fahmy Hanna, A. T. Jotheeswaran, Dan Luo, Rachana Parikh, Eesha Sharma, Shamaila Usmani, Yu Yu, Benjamin G. Druss, Shekhar Saxena**. The magnitude of and health system responses to the mental health treatment gap in adults in India and China.. *The Lancet*. 2016/12/17/. 388:3074	Level 1, Form Title/abstract screen
2056	**Sarah K. Hill, Peggy Cantrell, Joellen Edwards, Will Dalton**. Factors influencing mental health screening and treatment among women in a rural south central Appalachian primary care clinic.. *The Journal of Rural Health*. 2016///Win 2016. 32:82	Level 1, Form Title/abstract screen
2062	**Cynthia Shellhaas, Elizabeth Conrey, Dushka Crane, Allison Lorenz, Andrew Wapner, Reena Oza-Frank, Jo Bouchard**. The Ohio gestational diabetes postpartum care learning collaborative: Development of a quality improvement initiative to improve systems of care for women.. *Maternal and Child Health Journal*. 2016/11//. 20:71	Level 1, Form Title/abstract screen
2066	**Jeanette Waxmonsky, Lilia Verchinina, Hyungjin Myra Kim, Zongshan Lai, Daniel Eisenberg, Julia T. Kyle, Kristina M. Nord, Jenny H. Rementer, David E. Goodrich, Mark S. Bauer, Marshall R. Thomas, Amy M. Kilbourne**. Correlates of emergency department use by individuals with bipolar disorder enrolled in a collaborative care implementation study.. *Psychiatric Services*. 2016/11/01/. 67:1265	Level 1, Form Title/abstract screen
2068	**William R. Morrone**. President's message: Food and Drug Administration approved naloxone and continued use of improvised nasal naloxone: What is a treatment advocate and educator to do?. *Journal of Addictive Diseases*. 2016/10//. 35:339	Level 1, Form Title/abstract screen
2069	**Laura Pavia, Maria Di Blasi, Antonia Cinquegrana, Ernesto Scioti, Tiziana Bussola, Annaluisa Pasinelli, Paola Cavani**. The influence of retention, turnover, and alliance on process and outcomes in rolling group psychotherapy for cocaine disorder.. *International Journal of Group Psychotherapy*. 2016/10//. 66:526	Level 1, Form Title/abstract screen
2072	**Robert Sky Allen, Bradley D. Olson**. Predicting attrition in the treatment of substance use disorders.. *International Journal of Mental Health and Addiction*. 2016/10//. 14:728	Level 1, Form Title/abstract screen
2075	**Nuria Mallorquí-Bagué, Ana B. Fagundo, Susana Jimenez-Murcia, Rafael de la Torre, Rosa M. Baños, Cristina Botella, Felipe F. Casanueva, Ana B. Crujeiras, Jose C. Fernández-García, Jose M. Fernández-Real, Gema Frühbeck, Roser Granero, Amaia Rodríguez, Iris Tolosa-Sola, Francisco J.**	Level 1, Form Title/abstract screen

	**Ortega, Francisco J. Tinahones, Eva Alvarez-Moya, Cristian Ochoa, Jose M. Menchón, Fernando Fernández-Aranda**. Decision making impairment: A shared vulnerability in obesity, gambling disorder and substance use disorders?. *PLoS ONE*. 2016/09/30/. 11:#pages#	
2076	**Rinat Feniger-Schaal**. A dramatherapy case study with a young man who has dual diagnosis of intellectual disability and mental health problems.. *The Arts in Psychotherapy*. 2016/09//. 50:40	Level 1, Form Title/abstract screen
2078	**Chelsea Faye Dale, Victoria Christine Fontana, Julia Anna Martinez**. What's your 'vice?': A combined approach to drugs and other addictive substances and activities.. *Addiction Research & Theory*. 2016/09//. 24:366	Level 1, Form Title/abstract screen
2080	**Gregorio González-Alcaide, Amador Calafat, Elisardo Becoña, Bart Thijs, Wolfgang Glänzel**. Co-citation analysis of articles published in substance abuse journals: Intellectual structure and research fields (2001–2012).. *Journal of Studies on Alcohol and Drugs*. 2016/09//. 77:710	Level 1, Form Title/abstract screen
2082	**Megan Kirouac, Katie Witkiewitz, Dennis M. Donovan**. Client Evaluation of Treatment for alcohol use disorder in COMBINE.. *Journal of Substance Abuse Treatment*. 2016/08//. 67:38	Level 1, Form Title/abstract screen
2083	**Robert M. Bray, Charles C. Engel, Jason Williams, Lisa H. Jaycox, Marian E. Lane, Jessica K. Morgan, Jürgen Unützer**. Posttraumatic stress disorder in U.S. Military primary care: Trajectories and predictors of one-year prognosis.. *Journal of Traumatic Stress*. 2016/08//. 29:340	Level 1, Form Title/abstract screen
2087	**Julie F. Brown, Johnnie Hamilton-Mason, Peter Maramaldi, L. Jarrett Barnhill**. Exploring perspectives of individuals with intellectual disabilities and histories of challenging behaviors about family relationships: An emergent topic in a grounded theory focus group study.. *Journal of Mental Health Research in Intellectual Disabilities*. 2016/07//. 9:133	Level 1, Form Title/abstract screen
2089	**Debra Houry, Grant Baldwin**. Announcing the CDC guideline for prescribing opioids for chronic pain.. *Journal of Safety Research*. 2016/06//. 57:83	Level 1, Form Title/abstract screen
2094	**Wesley Sowers, Annelle Primm, Deborah Cohen, Jacquelyn Pettis, Ken Thompson**. Transforming psychiatry: A curriculum on recovery-oriented care.. *Academic Psychiatry*. 2016/06//. 40:461	Level 1, Form Title/abstract screen
2100	**Jack Bergman, Paul Willner, Louk Vanderschuren, Bart Ellenbroek**. Addiction: Etiology and therapeutics.. *Behavioural Pharmacology*. 2016/04//. 27:83	Level 1, Form Title/abstract screen
2104	**Rolf Sundet, Hesook Suzie Kim, Ottar Ness, Marit Borg, Bengt Karlsson, Stian Biong**. Collaboration: Suggested understandings.. *Australian and New Zealand Journal of Family Therapy*. 2016/03//. 37:93	Level 1, Form Title/abstract screen
2105	**Agnes Higgins, Louise Doyle, Carmel Downes, Jean Morrissey, Paul Costello, Michael Brennan, Michael Nash**. There is more to risk and safety planning than dramatic risks: Mental health nurses' risk assessment and safety-management practice.. *International Journal of Mental Health Nursing*. 2016/03//. 25:159	Level 1, Form Title/abstract screen
2106	**Diana L. Perry, Monica L. Daniels**. Implementing trauma—Informed practices in the school setting: A pilot study.. *School Mental Health*. 2016/03//. 8:177	Level 1, Form Title/abstract screen
2108	**Vibeke Zoffmann, Åsa Hörnsten, Solveig Storbække‍n, Marit Graue, Bodil Rasmussen, Astrid Wahl, Marit Kirkevold**. Translating person-centered care into practice: A comparative analysis of motivational interviewing, illness-	Level 1, Form Title/abstract screen

	integration support, and guided self-determination.. *Patient Education and Counseling*. 2016/03//. 99:400	
2110	**Kulnaree Hanpatchaiyakul, Henrik Eriksson, Jureerat Kijsomporn, Gunnel Östlund**. Healthcare providers' experiences of working with alcohol addiction treatment in Thailand.. *Contemporary Nurse*. 2016/02//. 52:59	Level 1, Form Title/abstract screen
2111	**Todd C. Edwards, Rob J. Fredericksen, Heidi M. Crane, Paul K. Crane, Mari M. Kitahata, William C. Mathews, Kenneth H. Mayer, Leo S. Morales, Michael J. Mugavero, Rosa Solorio, Frances M. Yang, Donald L. Patrick**. Content validity of Patient-Reported Outcomes Measurement Information System (PROMIS) items in the context of HIV clinical care.. *Quality of Life Research: An International Journal of Quality of Life Aspects of Treatment, Care & Rehabilitation*. 2016/02//. 25:293	Level 1, Form Title/abstract screen
2113	**Michael K. Monisse-Redman**. Collaborative practice development to improve clinical outcomes for adolescents with co-occurring mental illness, substance use and impulse control disorders.. *Child and adolescent health yearbook 2015*.. 2016///. #volume#:117	Level 1, Form Title/abstract screen
2114	**Nananda F. Col, Vicky Springmann**. Decision aids: Do they work?. *Handbook of health decision science*.. 2016///. #volume#:279	Level 1, Form Title/abstract screen
2115	**Ramin Mojtabai**. Commentary: Common themes and divergent views on heterogeneity in long-term course and outcome of adult mental and substance disorders.. *Long-term outcomes in psychopathology research: Rethinking the scientific agenda*.. 2016///. #volume#:77	Level 1, Form Title/abstract screen
2116	**Caroline Giroux, W. Edwin Smith**. Psychotherapy principles.. *On-call geriatric psychiatry: Handbook of principles and practice*.. 2016///. #volume#:45	Level 1, Form Title/abstract screen
2118	**Douglas Opler, Shaojie Han**. Stimulants.. *Pocket guide to addiction assessment and treatment*.. 2016///. #volume#:201	Level 1, Form Title/abstract screen
2119	**David R. McDuff**. Adjustment and anxiety disorders.. *Sports psychiatry*.. 2016///. #volume#:1	Level 1, Form Title/abstract screen
2120	**Casey T. Taft, Christopher M. Murphy, Suzannah K. Creech**. Adjunctive interventions.. *Trauma-informed treatment and prevention of intimate partner violence*.. 2016///. #volume#:175	Level 1, Form Title/abstract screen
2121	**Marie Tournier**. A need for personalised suicidology: Pharmacoepidemiology.. *Understanding suicide: From diagnosis to personalized treatment*.. 2016///. #volume#:403	Level 1, Form Title/abstract screen
2124	**Michael Rentrop, Susanne Hörz-Sagstetter**. Übertragungsfokussierte Psychotherapie und Sucht. = Transference-focused psychotherapy and addiction.. *PTT: Persönlichkeitsstörungen Theorie und Therapie*. 2016///. 20:43	Level 1, Form Title/abstract screen
2130	**Denise M. Connor, G. David Elkin, Kewchang Lee, Vanessa Thompson, Heather Whelan**. The unbefriended patient: An exercise in ethical clinical reasoning.. *Journal of General Internal Medicine*. 2016/01//. 31:128	Level 1, Form Title/abstract screen
2131	**Kim Markie-Frost**. The challenges and possibilities of social work with indigenous families.. *International Journal of Disability, Development and Education*. 2016/01//. 63:140	Level 1, Form Title/abstract screen
2134	**Mary Ann Burg, Oliver Oyama**. The behavioral health specialist in primary care: Skills for integrated practice.. *#journal#*. 2016///. #volume#:#pages#	Level 1, Form Title/abstract

		screen
2135	**Damita SunWolf LaRue**. Effects of history on Native American Indians: Providing culturally-sensitive therapeutic care.. *Dissertation Abstracts International: Section B: The Sciences and Engineering*. 2016///. 77:#pages#	Level 1, Form Title/abstract screen
2136	**Florencia Lebensohn-Chialvo**. Adaptive significance of personal pronoun use in families of adolescent substance abusers.. *Dissertation Abstracts International: Section B: The Sciences and Engineering*. 2016///. 76:#pages#	Level 1, Form Title/abstract screen
2137	**Bethany Remeniuk**. Capturing affective dimensions of cancer-induced bone pain preclinically.. *Dissertation Abstracts International: Section B: The Sciences and Engineering*. 2016///. 76:#pages#	Level 1, Form Title/abstract screen
2138	**Christopher L. Smith**. Role of Substance Use and Working Alliance on relapse in treatment for early psychosis.. *Dissertation Abstracts International: Section B: The Sciences and Engineering*. 2016///. 76:#pages#	Level 1, Form Title/abstract screen
2139	**Songyuan Tang**. Factors associated with effectiveness of Methadone Maintenance Treatment in Yunnan province of China.. *Dissertation Abstracts International: Section B: The Sciences and Engineering*. 2016///. 76:#pages#	Level 1, Form Title/abstract screen
2140	**Kathleen M. Grubbs, John C. Fortney, Jeffrey M. Pyne, Teresa Hudson, William Mark Moore, Paul Custer, Ronald Schneider, Paula P. Schnurr**. Predictors of initiation and engagement of cognitive processing therapy among veterans with PTSD enrolled in collaborative care.. *Journal of Traumatic Stress*. 2015/12//. 28:580	Level 1, Form Title/abstract screen
2142	**Lisa Tlach, Caroline Wüsten, Anne Daubmann, Sarah Liebherz, Martin Härter, Jörg Dirmaier**. Information and decision-making needs among people with mental disorders: A systematic review of the literature.. *Health Expectations: An International Journal of Public Participation in Health Care & Health Policy*. 2015/12//. 18:1856	Level 1, Form Title/abstract screen
2143	**Rahul Shidhaye, Crick Lund, Dan Chisholm**. Closing the treatment gap for mental, neurological and substance use disorders by strengthening existing health care platforms: Strategies for delivery and integration of evidence-based interventions.. *International Journal of Mental Health Systems*. 2015/12//. 9:#pages#	Level 1, Form Title/abstract screen
2147	**Douglas Zatzick, Stephen S. O'Connor, Joan Russo, Jin Wang, Nigel Bush, Jeff Love, Roselyn Peterson, Leah Ingraham, Doyanne Darnell, Lauren Whiteside, Erik Van Eaton**. Technology-enhanced stepped collaborative care targeting posttraumatic stress disorder and comorbidity after injury: A randomized controlled trial.. *Journal of Traumatic Stress*. 2015/10//. 28:391	Level 1, Form Title/abstract screen
2149	**Traci C. Green, Emily F. Dauria, Jeffrey Bratberg, Corey S. Davis, Alexander Y. Walley**. Orienting patients to greater opioid safety: Models of community pharmacy-based naloxone.. *Harm Reduction Journal*. 2015/08/06/. 12:#pages#	Level 1, Form Title/abstract screen
2150	**Neil J. Kitchiner**. Review of Care of military service members, veterans, and their families.. *The British Journal of Psychiatry*. 2015/08//. 207:180	Level 1, Form Title/abstract screen
2151	**Mary P. Ghods, Ian T. Schmid, Carol A. Pamer, Brian M. Lappin, Dale C. Slavin**. Developing and initiating validation of a model opioid patient-prescriber agreement as a tool for patient-centered pain treatment.. *The Patient: Patient-Centered Outcomes Research*. 2015/08//. 8:349	Level 1, Form Title/abstract screen
2153	**Richard M. Hoffman, Andrew L. Sussman, Christina M. Getrich, Robert L. Rhyne, Richard E. Crowell, Kathryn L. Taylor, Ellen J. Reifler, Pamela**	Level 1, Form Title/abstract

	**H. Wescott, Ambroshia M. Murrietta, Ali I. Saeed, Shiraz I. Mishra**. Attitudes and beliefs of primary care providers in New Mexico about lung cancer screening using low-dose computed tomography.. *Preventing Chronic Disease: Public Health Research, Practice, and Policy*. 2015/07/09/. 12:#pages#	screen
2157	**George De Leon**. 'The gold standard' and related considerations for a maturing science of substance abuse treatment. Therapeutic communities; A case in point.. *Substance Use & Misuse*. 2015/07//. 50:1106	Level 1, Form Title/abstract screen
2158	**Susan E. Collins, Véronique S. Grazioli, Nicole I. Torres, Emily M. Taylor, Connor B. Jones, Gail E. Hoffman, Laura Haelsig, Mengdan D. Zhu, Alyssa S. Hatsukami, Molly J. Koker, Patrick Herndon, Shawna M. Greenleaf, Parker E. Dean**. Qualitatively and quantitatively evaluating harm-reduction goal setting among chronically homeless individuals with alcohol dependence.. *Addictive Behaviors*. 2015/06//. 45:184	Level 1, Form Title/abstract screen
2159	**Sanno E. Zack, Louis G. Castonguay, James F. Boswell, Andrew A. McAleavey, Robert Adelman, David R. Kraus, George A. Pate**. Attachment history as a moderator of the alliance outcome relationship in adolescents.. *Psychotherapy*. 2015/06//. 52:258	Level 1, Form Title/abstract screen
2164	**Paula C. G. Alves, Célia M. D. Sales, Mark Ashworth**. Personalising the evaluation of substance misuse treatment: A new approach to outcome measurement.. *International Journal of Drug Policy*. 2015/04//. 26:333	Level 1, Form Title/abstract screen
2165	**Udi E. Ghitza**. Needed relapse-prevention research on novel framework (ASPIRE model) for substance use disorders treatment.. *Frontiers in Psychiatry*. 2015/03/06/. 6:#pages#	Level 2, Form Full Text Screening
2166	**Arieahn Matamonasa-Bennett**. 'A disease of the outside people': Native American men's perceptions of intimate partner violence.. *Psychology of Women Quarterly*. 2015/03//. 39:20	Level 1, Form Title/abstract screen
2168	**Christina Brezing, Maria Ferrara, Oliver Freudenreich**. The syndemic Illness of HIV and trauma: Implications for a trauma-informed model of care.. *Psychosomatics: Journal of Consultation and Liaison Psychiatry*. 2015/03//. 56:107	Level 1, Form Title/abstract screen
2170	**J. MacLaren Kelly, Ewgeni Jakubovski, Michael H. Bloch**. Prognostic subgroups for remission and response in the Coordinated Anxiety Learning and Management (CALM) trial.. *The Journal of Clinical Psychiatry*. 2015/03//. 76:267	Level 1, Form Title/abstract screen
2172	**Iain H. McKillop**. Alcohol and the brain–liver axis: A further case of mind over matter?. *Alcoholism: Clinical and Experimental Research*. 2015/03//. 39:405	Level 1, Form Title/abstract screen
2175	**Bradley V. Watts, Paula P. Schnurr, Maha Zayed, Yinong Young-Xu, Patricia Stender, Hilary Llewellyn-Thomas**. A randomized controlled clinical trial of a patient decision aid for posttraumatic stress disorder.. *Psychiatric Services*. 2015/02/01/. 66:149	Level 1, Form Title/abstract screen
2176	**Rachel Kimerling, Lori A. Bastian, Bevanne A. Bean-Mayberry, Meggan M. Bucossi, Diane V. Carney, Karen M. Goldstein, Ciaran S. Phibbs, Alyssa Pomernacki, Anne G. Sadler, Elizabeth M. Yano, Susan M. Frayne**. Patient-centered mental health care for female veterans.. *Psychiatric Services*. 2015/02/01/. 66:155	Level 1, Form Title/abstract screen
2178	**Lori Raney, Gina Lasky, Clare Scott**. The collaborative care team in action.. *Integrated care: Working at the interface of primary care and behavioral health*.. 2015///. #volume#:17	Level 1, Form Title/abstract screen

2179	**Joseph Parks**. Behavioral health homes.. *Integrated care: Working at the interface of primary care and behavioral health*.. 2015///. #volume#:193	Level 2, Form Full Text Screening
2180	**W. Scott Craig**. The importance of integrated psychological services in primary care.. *Integrated psychological services in primary care*.. 2015///. #volume#:3	Level 1, Form Title/abstract screen
2181	**Ou Sha, Yue Hao, Eric Yu-Pang Cho, Li Zhou**. Clinical applications and side effects of ketamine.. *Ketamine: Use and abuse*.. 2015///. #volume#:13	Level 1, Form Title/abstract screen
2182	**Kimberly Stoner**. Laboratory tests.. *Psychiatric care of the medical patient*.. 2015///. #volume#:105	Level 1, Form Title/abstract screen
2183	**Kenneth Wayne Phelps, Ashley Blackmon Jones, Rebecca Ann Payne**. The interplay between mental and sexual health.. *Systemic sex therapy*.. 2015///. #volume#:255	Level 1, Form Title/abstract screen
2184	**Maxine Stitzer, Nancy Petry**. Contingency management.. *The American Psychiatric Publishing textbook of substance abuse treatment*.. 2015///. #volume#:423	Level 1, Form Title/abstract screen
2185	**Henry J. Steadman, Brian Case, Chanson Noether, Samantha Califano, Susan Salasin**. From resource center to systems change: The GAINS Model.. *The sequential intercept model and criminal justice: Promoting community alternatives for individuals with serious mental illness*.. 2015///. #volume#:137	Level 1, Form Title/abstract screen
2186	**Lorraine Greaves, Nancy Poole, Ellexis Boyle**. The future of transdisciplinarity in addiction.. *Transforming addiction: Gender, trauma, transdisciplinarity*.. 2015///. #volume#:216	Level 2, Form Full Text Screening
2187	**Patrick J. Raue, Patricia A. Areán**. Interpersonal psychotherapy for late-life depression.. *Treatment of late-life depression, anxiety, trauma, and substance abuse*.. 2015///. #volume#:71	Level 1, Form Title/abstract screen
2188	**Frederik Haarig, Stephan Mühlig**. Nutzung von Conjoint-Analysen zur Messung von Therapiezielpräferenzen aus Patientenperspektive in der Behandlung psychischer Störungen: Eine systematische Literaturübersicht. = Conjoint analysis for measuring treatment preferences of patients with psychiatric disorders: A systematic literature review.. *Zeitschrift für Klinische Psychologie und Psychotherapie: Forschung und Praxis*. 2015/01//. 44:1	Level 1, Form Title/abstract screen
2190	**Molly R. Wolf, Thomas H. Nochajski, Hon. Mark G. Farrell**. The effects of childhood sexual abuse and other trauma on drug court participants.. *Journal of Social Work Practice in the Addictions*. 2015/01//. 15:44	Level 1, Form Title/abstract screen
2196	**Lori Holleran Steiker**. Trauma-Informed Care and Addiction Recovery: An Interview With Nancy J. Smyth, PhD, LCSW.. *Journal of Social Work Practice in the Addictions*. 2015/01//. 15:134	Level 2, Form Full Text Screening
2197	**Hal Arkowitz, William R. Miller, Stephen Rollnick**. Motivational interviewing in the treatment of psychological problems., 2nd ed.. *#journal#*. 2015///. #volume#:#pages#	Level 1, Form Title/abstract screen
2198	**Jessica Lynn Larsen**. Trauma and the justice-involved veteran.. *Dissertation Abstracts International: Section B: The Sciences and Engineering*. 2015///. 76:#pages#	Level 1, Form Title/abstract screen
2199	**John S. Wodarski, Sarah V. Curtis**. E-therapy for substance abuse and co-morbidity.. *#journal#*. 2015///. #volume#:#pages#	Level 1, Form Title/abstract screen

2202	**Thomas J. Waltz, Duncan G. Campbell, JoAnn E. Kirchner, Anayansi Lombardero, Cory Bolkan, Kara Zivin, Andrew B. Lanto, Edmund F. Chaney, Lisa V. Rubenstein**. Veterans with depression in primary care: Provider preferences, matching, and care satisfaction.. *Families, Systems, & Health*. 2014/12//. 32:367	Level 1, Form Title/abstract screen
2203	**My Frankl, Björn Philips, Peter Wennberg**. Psychotherapy role expectations and experiences—Discrepancy and therapeutic alliance among patients with substance use disorders.. *Psychology and Psychotherapy: Theory, Research and Practice*. 2014/12//. 87:411	Level 1, Form Title/abstract screen
2204	**Alexis Kuerbis, Stephen Armeli, Frederick Muench, Jon Morgenstern**. Profiles of confidence and commitment to change as predictors of moderated drinking: A person-centered approach.. *Psychology of Addictive Behaviors*. 2014/12//. 28:1065	Level 1, Form Title/abstract screen
2205	**Ingo Schäfer, Lil Gromus, Armita Atabaki, Silke Pawils, Uwe Verthein, Jens Reimer, Bernd Schulte, Marcus Martens**. Are experiences of sexual violence related to special needs in patients with substance use disorders? A study in opioid-dependent patients.. *Addictive Behaviors*. 2014/12//. 39:1691	Level 1, Form Title/abstract screen
2206	**Beth D. Darnall, Michael E. Schatman**. Urine drug screening: Necessary or alienating?. *Pain Medicine*. 2014/12//. 15:1999	Level 1, Form Title/abstract screen
2211	**Mustafa C. Karakus**. Affordable Care Act and behavioral health services: Special section editor's note.. *The Journal of Behavioral Health Services & Research*. 2014/10//. 41:408	Level 1, Form Title/abstract screen
2215	**Rachael Hinton, Pat Bradley, Thomas Trauer, Tricia Nagel**. Strengthening acute inpatient mental health care for Indigenous clients.. *Advances in Mental Health*. 2014/08//. 12:125	Level 1, Form Title/abstract screen
2216	**Kurt Kroenke, Erin E. Krebs, Jingwei Wu, Zhangsheng Yu, Neale R. Chumbler, Matthew J. Bair**. Telecare collaborative management of chronic pain in primary care: A randomized clinical trial.. *JAMA: Journal of the American Medical Association*. 2014/07/16/. 312:240	Level 1, Form Title/abstract screen
2219	**Jessica Roberts Williams, Kevin D. Hennessy**. Increasing adoption of comparative effectiveness research in community behavioral health: Introduction to special section.. *The Journal of Behavioral Health Services & Research*. 2014/07//. 41:254	Level 1, Form Title/abstract screen
2220	**Jessica Roberts Williams, Stephen J. Tregear, Tracy Dusablon, Lizbeth E. Caceda-Castro, Kristin J. Miller, Gary Hill, Kevin D. Hennessy**. Increasing adoption of comparative effectiveness research in community behavioral health: Methodology.. *The Journal of Behavioral Health Services & Research*. 2014/07//. 41:258	Level 1, Form Title/abstract screen
2223	**Noell L. Rowan, Sandra S. Butler**. Resilience in attaining and sustaining sobriety among older lesbians with alcoholism.. *Journal of Gerontological Social Work*. 2014/05//. 57:176	Level 1, Form Title/abstract screen
2224	**Susan H. McDaniel, Frank V. III deGruy**. An introduction to primary care and psychology.. *American Psychologist*. 2014/05//May-Jun, 2014. 69:325	Level 1, Form Title/abstract screen
2225	**Debra Sheets**. Review of Transgender Tuesdays: A clinic in the tenderloin.. *Journal of Gerontological Social Work*. 2014/05//. 57:413	Level 1, Form Title/abstract screen
2226	**Melissa M. Goldstein**. Health information privacy and health information	Level 1, Form

	technology in the US correctional setting.. *American Journal of Public Health*. 2014/05//. 104:803	Title/abstract screen
2227	**Jeffrey P. Haibach, Gregory P. Beehler, Katherine M. Dollar, Deborah S. Finnell**. Moving toward integrated behavioral intervention for treating multimorbidity among chronic pain, depression, and substance-use disorders in primary care.. *Medical Care*. 2014/04//. 52:322	Level 1, Form Title/abstract screen
2228	**Stephanie Rodgers, Martin Grosse Holtforth, Mario Müller, Michael P. Hengartner, Wulf Rössler, Vladeta Ajdacic-Gross**. Symptom-based subtypes of depression and their psychosocial correlates: A person-centered approach focusing on the influence of sex.. *Journal of Affective Disorders*. 2014/03/01/. 156:92	Level 1, Form Title/abstract screen
2229	**Patricia A. Cioe**. 'Regarding: Cardiovascular risk-factor knowledge and risk perception among HIV-infected adults': Reply to the editor.. *JANAC: Journal of the Association of Nurses in AIDS Care*. 2014/03//. 25:106	Level 1, Form Title/abstract screen
2230	**Gerhard H. H. Müller-Schwefe, Antonie M. Wimmer, Joachim Dejonckheere, Antje Eggers, Renato Vellucci**. Patients' and physicians' perspectives on opioid therapy for chronic cancer and musculoskeletal pain in Germany, Italy, and Turkey: Pain Research (PARES) Survey.. *Current Medical Research and Opinion*. 2014/03//. 30:339	Level 1, Form Title/abstract screen
2231	**Josephine D. Korchmaros, Sally J. Stevens**. Examination of the role of therapeutic alliance, treatment dose, and treatment completion in the effectiveness of The Seven Challenges.. *Child & Adolescent Social Work Journal*. 2014/02//. 31:1	Level 1, Form Title/abstract screen
2233	**David B. Bekelman, Stephanie Hooker, Carolyn T. Nowels, Deborah S. Main, Paula Meek, Connor McBryde, Brack Hattler, Karl A. Lorenz, Paul A. Heidenreich**. Feasibility and acceptability of a collaborative care intervention to improve symptoms and quality of life in chronic heart failure: Mixed methods pilot trial.. *Journal of Palliative Medicine*. 2014/02//. 17:145	Level 1, Form Title/abstract screen
2235	**Ritu Chahil**. Narcissism.. *Essential psychopathology casebook.*. 2014///. #volume#:537	Level 1, Form Title/abstract screen
2236	**Edna Hamera**. Motivational interviewing.. *Psychotherapy for the advanced practice psychiatric nurse: A how-to guide for evidence-based practice.*. 2014///. #volume#:299	Level 2, Form Full Text Screening
2237	**Susie Adams, Deborah Antai-Otong**. Psychotherapeutic approaches for addictions and related disorders.. *Psychotherapy for the advanced practice psychiatric nurse: A how-to guide for evidence-based practice.*. 2014///. #volume#:565	Level 2, Form Full Text Screening
2238	**Anne C. Dobmeyer, Jeffrey L. Goodie, Christopher L. Hunter**. Health care provider and system interventions promoting health behavior change.. *The handbook of health behavior change.*. 2014///. #volume#:417	Level 1, Form Title/abstract screen
2243	**Scott T. Walters, Steven J. Ondersma, Karen S. Ingersoll, Mayra Rodriguez, Jennifer Lerch, Matthew E. Rossheim, Faye S. Taxman**. MAPIT: Development of a web-based intervention targeting substance abuse treatment in the criminal justice system.. *Journal of Substance Abuse Treatment*. 2014/01//. 46:60	Level 1, Form Title/abstract screen
2244	**Jeanette Waxmonsky, Amy M. Kilbourne, David E. Goodrich, Kristina M. Nord, Zongshan Lai, Christina Laird, Julia Clogston, Hyungjin Myra Kim, Christopher Miller, Mark S. Bauer**. Enhanced fidelity to treatment for bipolar disorder: Results From a randomized controlled implementation trial..	Level 1, Form Title/abstract screen

	Psychiatric Services. 2014/01/01/. 65:81	
2250	**Myrna M. Weissman, Sidney H. Hankerson, Pamela Scorza, Mark Olfson, Helena Verdeli, Steven Shea, Rafael Lantigua, Milton Wainberg**. Interpersonal counseling (IPC) for depression in primary care.. *American Journal of Psychotherapy*. 2014///. 68:359	Level 1, Form Title/abstract screen
2251	**Ellen Frank, Fiona C. Ritchey, Jessica C. Levenson**. Is interpersonal psychotherapy infinitely adaptable? A compendium of the multiple modifications of IPT.. *American Journal of Psychotherapy*. 2014///. 68:385	Level 1, Form Title/abstract screen
2252	**Neal Adams, Diane M. Grieder**. Treatment planning for person-centered care: Shared decision making for whole health., 2nd ed.. *#journal#*. 2014///. #volume#:#pages#	Level 1, Form Title/abstract screen
2253	**LaTrica Y. Henegar**. A qualitative study of educator and counselor interdisciplinary collaboration regarding adolescent substance abusers' academic attainment.. *Dissertation Abstracts International Section A: Humanities and Social Sciences*. 2014///. 74:#pages#	Level 1, Form Title/abstract screen
2254	**Tanya R. Sorrell**. Mental health treatment preferences for persons of Mexican heritage.. *Dissertation Abstracts International: Section B: The Sciences and Engineering*. 2014///. 74:#pages#	Level 1, Form Title/abstract screen
2255	**James John Weidel**. The relationship of temporal discounting and working alliance to substance abuse treatment process in hispanic adolescents.. *Dissertation Abstracts International: Section B: The Sciences and Engineering*. 2014///. 75:#pages#	Level 2, Form Full Text Screening
2256	**Garry Williamson**. The effect of counselor changes and ruptures in the therapeutic alliance on treatment outcome for methadone maintenance clients in an outpatient clinic.. *Dissertation Abstracts International: Section B: The Sciences and Engineering*. 2014///. 75:#pages#	Level 2, Form Full Text Screening
2258	**Laura J. Holt, Stephen Armeli, Howard Tennen, Carol S. Austad, Sarah A. Raskin, Carolyn R. Fallahi, Rebecca Wood, Rivkah I. Rosen, Meredith K. Ginley, Godfrey D. Pearlson**. A person-centered approach to understanding negative reinforcement drinking among first year college students.. *Addictive Behaviors*. 2013/12//. 38:2937	Level 1, Form Title/abstract screen
2259	**Vivian Barnett Brown, Maxine Harris, Roger Fallot**. Moving toward trauma-informed practice in addiction treatment: A collaborative model of agency assessment.. *Journal of Psychoactive Drugs*. 2013/11//. 45:386	Level 2, Form Full Text Screening
2262	**Catrin Lewis, Neil Roberts, Tracey Vick, Jonathan I. Bisson**. Development of a guided self-help (GSH) program for the treatment of mild-to-moderate posttraumatic stress disorder (PTSD).. *Depression and Anxiety*. 2013/11//. 30:1121	Level 1, Form Title/abstract screen
2263	**Thérèse von Braun, Sam Larsson, Yvonne Sjöblom**. Chapter 10. Perspectives on treatment, alliance and narratives concerning substance use-related dependency.. *Substance Use & Misuse*. 2013/11//. 48:1386	Level 2, Form Full Text Screening
2265	**Christoph Flückiger, Aaron C. Del Re, Adam O. Horvath, Dianne Symonds, Michael Ackert, Bruce E. Wampold**. Substance use disorders and racial/ethnic minorities matter: A meta-analytic examination of the relation between alliance and outcome.. *Journal of Counseling Psychology*. 2013/10//. 60:610	Level 1, Form Title/abstract screen
2266	**Hein A. de Haan, Evelien A. G. Joosten, Lydia de Haan, Arnt F. A. Schellekens, Jan K. Buitelaar, Job van der Palen, Cor A. J. De Jong**. A family history of alcoholism relates to alexithymia in substance use disorder	Level 1, Form Title/abstract screen

	patients.. *Comprehensive Psychiatry*. 2013/10//. 54:911	
2269	**John Oldham**. From the editor: Integrated care.. *Journal of Psychiatric Practice*. 2013/09//. 19:343	Level 1, Form Title/abstract screen
2270	**Arthur M. Michalek**. Back to the future… the challenge for cancer education and training in developing countries.. *Journal of Cancer Education*. 2013/09//. 28:395	Level 1, Form Title/abstract screen
2273	**Henry Chung, Azalea Kim, Charles J. Neighbors, Johnine Cummings, Sally Ricketts, Megan A. O'Grady, Donald Raum**. Early experience of a pilot intervention for patients with depression and chronic medical illness in an urban ACO.. *General Hospital Psychiatry*. 2013/09//Sep-Oct, 2013. 35:468	Level 1, Form Title/abstract screen
2274	**Yolonda R. Pickett, Kisha N. Bazelais, Martha L. Bruce**. Late-life depression in older African Americans: A comprehensive review of epidemiological and clinical data.. *International Journal of Geriatric Psychiatry*. 2013/09//. 28:903	Level 1, Form Title/abstract screen
2279	**Jane M. Plagge, Mary W. Lu, Travis I. Lovejoy, Andrea I. Karl, Steven K. Dobscha**. Treatment of comorbid pain and PTSD in returning veterans: A collaborative approach utilizing behavioral activation.. *Pain Medicine*. 2013/08//. 14:1164	Level 1, Form Title/abstract screen
2283	**Margaret W. Bultas, Mary P. Curtis**. Using simulation to teach child injury prevention to mothers recovering from substance abuse.. *Journal of Community Health Nursing*. 2013/07//. 30:155	Level 1, Form Title/abstract screen
2284	**Nicola Thomas, Rosamund Bryar**. An evaluation of self-management package for people with diabetes at risk of chronic kidney disease.. *Primary Health Care Research and Development*. 2013/07//. 14:270	Level 1, Form Title/abstract screen
2287	**Brad Donohue, Michelle Pitts, Yulia Gavrilova, Ashley Ayarza, Kristina I. Cintron**. A culturally sensitive approach to treating substance abuse in athletes using evidence-supported methods.. *Journal of Clinical Sport Psychology*. 2013/06//. 7:98	Level 1, Form Title/abstract screen
2289	**Jennifer S. Funderburk, Robyn L. Fielder**. A primary mental health care model for advanced practicum training in a university health clinic.. *Training and Education in Professional Psychology*. 2013/05//. 7:112	Level 1, Form Title/abstract screen
2291	**Carolyn Baird**. Delivering trauma-informed care.. *Journal of Addictions Nursing*. 2013/04//Apr-Jun, 2013. 24:126	Level 1, Form Title/abstract screen
2293	**Robert M. Post, Peter Kalivas**. Bipolar disorder and substance misuse: Pathological and therapeutic implications of their comorbidity and cross-sensitisation.. *The British Journal of Psychiatry*. 2013/03//. 202:172	Level 1, Form Title/abstract screen
2294	**Ellen J. Hahn, Mary Kay Rayens, Nancy York**. Readiness for smoke-free policy and overall strength of tobacco control in rural tobacco-growing communities.. *Health Promotion Practice*. 2013/03//. 14:238	Level 1, Form Title/abstract screen
2295	**Kurt B. Angstman, Norman H. Rasmussen, Kathy L. MacLaughlin, Jeffrey P. Staab**. Inter-relationship of the functional status question of the PHQ-9 and depression remission after six months of collaborative care management.. *Journal of Psychiatric Research*. 2013/03//. 47:418	Level 1, Form Title/abstract screen
2298	**Lianne Fuino Estefan, Martha L. Coulter, Carla L. VandeWeerd, Mary Armstrong, Peter Gorski**. Relationships between stressors and parenting attitudes in a child welfare parenting program.. *Journal of Child and Family*	Level 1, Form Title/abstract screen

	Studies. 2013/02//. 22:199	
2300	**Thomas J. Doyle, Peter D. Friedmann, William H. Zywiak**. Management of patients with alcohol dependence in recovery: Options for maintenance and anticipating and managing relapse in primary care.. *Addressing unhealthy alcohol use in primary care*.. 2013///. #volume#:85	Level 1, Form Title/abstract screen
2301	**Laura Weiss Roberts, Kim Bullock**. Ethical considerations in caring for people living with addictions.. *Applied ethics in mental health care: An interdisciplinary reader*.. 2013///. #volume#:223	Level 2, Form Full Text Screening
2302	**Jennifer A. Robinson, James M. Bolton**. Substance use in response to anxiety disorders.. *Comprehensive addictive behaviors and disorders, Vol. 1: Principles of addiction*.. 2013///. #volume#:507	Level 1, Form Title/abstract screen
2303	**Kevin Huckshorn, Janice L. LeBel**. Trauma-informed care.. *Modern community mental health: An interdisciplinary approach*.. 2013///. #volume#:62	Level 1, Form Title/abstract screen
2304	**David Murphy, Stephen Joseph**. Facilitating posttraumatic growth through relational depth.. *Relational depth: New perspectives and developments*.. 2013///. #volume#:90	Level 1, Form Title/abstract screen
2305	**Donna Sabella**. Health issues and interactions with adult survivors.. *Sex trafficking: A clinical guide for nurses*.. 2013///. #volume#:151	Level 1, Form Title/abstract screen
2306	**Michael Mancini**. Assessment strategies for substance use disorders.. *Social work practice in the addictions*.. 2013///. #volume#:49	Level 2, Form Full Text Screening
2308	**Paula P. Schnurr, Matthew J. Friedman, Thomas E. Oxman, Allen J. Dietrich, Mark W. Smith, Brian Shiner, Elizabeth Forshay, Jiang Gui, Veronica Thurston**. RESPECT-PTSD: Re-engineering systems for the primary care treatment of PTSD, a randomized controlled trial.. *Journal of General Internal Medicine*. 2013/01//. 28:32	Level 1, Form Title/abstract screen
2309	**Francesco Bartoli, Silvio Scarone, Massimo Clerici**. Il rischio di ricovero in pazienti comorbili affetti da disturbi psicotici e da uso di sostanze: Follow-up a 12 anni. = Risk of hospitalization among patients with co-occurring psychotic and substance use disorders: A 12-year follow-up.. *Rivista di Psichiatria*. 2013/01//Jan-Feb, 2013. 48:51	Level 1, Form Title/abstract screen
2312	**Sylvia Trent-Adams, Laura W. Cheever**. Providing HIV pre-exposure prophylaxis: Lessons learned from the Ryan White HIV/AIDS Program.. *American Journal of Preventive Medicine*. 2013/01//. 44:S147	Level 1, Form Title/abstract screen
2313	**Cristina M. Benki**. Risks and needs of participants in batterer intervention programs.. *Dissertation Abstracts International: Section B: The Sciences and Engineering*. 2013///. 74:#pages#	Level 1, Form Title/abstract screen
2314	**Holly Joy D'Angelo-Scott**. An investigation of the impact of a multidisciplinary, collaborative maternity care model in Nova Scotia, Canada.. *Dissertation Abstracts International: Section B: The Sciences and Engineering*. 2013///. 74:#pages#	Level 1, Form Title/abstract screen
2315	**Anthony N. Giardina**. Factors affecting the therapeutic alliances' impact on substance use disorder treatment outcomes.. *Dissertation Abstracts International: Section B: The Sciences and Engineering*. 2013///. 74:#pages#	Level 2, Form Full Text Screening
2316	**Emily Jones**. Collaborative behavioral and medical care in community health centers: Trends 2000-2007, correlates of capacity, and elements of collaborative care.. *Dissertation Abstracts International Section A: Humanities and Social*	Level 1, Form Title/abstract screen

	Sciences. 2013///. 74:#pages#	
2317	**Sean P. McGowan**. Exploring whether the working alliance is a mediator between client resistance, therapist directiveness, and drinking outcomes in an alcohol dependent population.. *Dissertation Abstracts International: Section B: The Sciences and Engineering*. 2013///. 74:#pages#	Level 2, Form Full Text Screening
2318	**Annette M. McTague**. Examining correlations between readiness for treatment and relapse potential: Communicating data to improve client show rate.. *Dissertation Abstracts International: Section B: The Sciences and Engineering*. 2013///. 73:#pages#	Level 1, Form Title/abstract screen
2319	**D. Eugene Mead**. Becoming a marriage and family therapist: From classroom to consulting room.. *#journal#*. 2013///. #volume#:#pages#	Level 1, Form Title/abstract screen
2320	**Julie A. Nelson**. The relationships among shame, working alliance and aftercare attendance to treatment outcomes.. *Dissertation Abstracts International: Section B: The Sciences and Engineering*. 2013///. 73:#pages#	Level 1, Form Title/abstract screen
2321	**Barbara Jones Warren**. Shared decision making: A recovery cultural process.. *Journal of Psychosocial Nursing and Mental Health Services*. 2012/12//. 50:1	Level 1, Form Title/abstract screen
2324	**Allison R. Gilbert, Marisa E. Domino, Joseph P. Morrissey, Bradley N. Gaynes**. Differential service utilization associated with trauma-informed integrated treatment for women with co-occurring disorders.. *Administration and Policy in Mental Health and Mental Health Services Research*. 2012/11//. 39:426	Level 2, Form Full Text Screening
2325	**Inga Dennhag, Mary Beth Connolly Gibbons, Jacques P. Barber, Robert Gallop, Paul Crits-Christoph**. Do supervisors and independent judges agree on evaluations of therapist adherence and competence in the treatment of cocaine dependence?. *Psychotherapy Research*. 2012/11//. 22:720	Level 1, Form Title/abstract screen
2326	**Michael P. Chaney, Michael D. Brubaker**. Addiction in LGBTQ communities: Influences, treatment, and prevention.. *Journal of LGBT Issues in Counseling*. 2012/10//. 6:234	Level 1, Form Title/abstract screen
2327	**Laurie Drabble, Michele J. Eliason**. Substance use disorders treatment for sexual minority women.. *Journal of LGBT Issues in Counseling*. 2012/10//. 6:274	Level 2, Form Full Text Screening
2329	**Tytti Artkoski, Pekka Saarnio**. Therapist effects in substance abuse treatment: A naturalistic study.. *Journal of Substance Use*. 2012/10//. 17:456	Level 2, Form Full Text Screening
2331	**Patricia Nemec**. Review of The heart and soul of change: Delivering what works in therapy (2nd ed.).. *Psychiatric Rehabilitation Journal*. 2012/09//. 35:415	Level 1, Form Title/abstract screen
2332	**Liz Beddoe**. Review of Knowledge-in-practice in the caring professions: Multidisciplinary perspectives.. *Australian Social Work*. 2012/09//. 65:435	Level 1, Form Title/abstract screen
2333	**Larry Davidson**. Use of coercion in recovery-oriented care: Staying vigilant.. *Psychiatric Services*. 2012/08/01/. 63:834	Level 1, Form Title/abstract screen
2336	**Inga Dennhag, Mary Beth Connolly Gibbons, Jacques P. Barber, Robert Gallop, Paul Crits-Christoph**. How many treatment sessions and patients are needed to create a stable score of adherence and competence in the treatment of cocaine dependence?. *Psychotherapy Research*. 2012/07//. 22:475	Level 1, Form Title/abstract screen

2337	**Siama Rashid, Alex Copello, Max Birchwood**. Muslim faith healers' views on substance misuse and psychosis.. *Mental Health, Religion & Culture*. 2012/07//. 15:653	Level 1, Form Title/abstract screen
2340	**Ambreen Rashid**. Review of Human needs and intellectual disabilities: Applications for person centred planning, dual diagnosis and crisis intervention.. *Journal of Intellectual Disability Research*. 2012/06//. 56:652	Level 1, Form Title/abstract screen
2344	**Marina L. Merrill, Nicole L. Taylor, Alison J. Martin, Lauren A. Maxim, Ryan D'Ambrosio, Roy M. Gabriel, Staci J. Wendt, Danyelle Mannix, Michael E. Wells**. A mixed-method exploration of functioning in safe schools/healthy students partnerships.. *Evaluation and Program Planning*. 2012/05//. 35:280	Level 1, Form Title/abstract screen
2345	**Anilkrishna B. Thota, Theresa Ann Sipe, Guthrie J. Byard, Carlos S. Zometa, Robert A. Hahn, Lela R. McKnight-Eily, Daniel P. Chapman, Ana F. Abraido-Lanza, Jane L. Pearson, Clinton W. Anderson, Alan J. Gelenberg, Kevin D. Hennessy, Farifteh F. Duffy, Mary E. Vernon-Smiley, Donald E. Jr. Nease, Samantha P. Williams**. Collaborative care to improve the management of depressive disorders: A community guide systematic review and meta-analysis.. *American Journal of Preventive Medicine*. 2012/05//. 42:525	Level 1, Form Title/abstract screen
2348	**Thomas M. Richardson, Bruce Friedman, Carol Podgorski, Kerry Knox, Susan Fisher, Hua He, Yeates Conwell**. Depression and its correlates among older adults accessing aging services.. *The American Journal of Geriatric Psychiatry*. 2012/04//. 20:346	Level 1, Form Title/abstract screen
2350	**Annette Christy, Colleen Clark, Autumn Frei, Sarah Rynearson-Moody**. Challenges of diverting veterans to trauma informed care: The heterogeneity of Intercept 2.. *Criminal Justice and Behavior*. 2012/04//. 39:461	Level 1, Form Title/abstract screen
2351	**Telsie A. Davis, Julie Ancis**. Look to the relationship: A review of African American women substance users' poor treatment retention and working alliance development.. *Substance Use & Misuse*. 2012/04//. 47:662	Level 2, Form Full Text Screening
2355	**Elizabeth M. Yano, Edmund F. Chaney, Duncan G. Campbell, Ruth Klap, Barbara F. Simon, Laura M. Bonner, Andrew B. Lanto, Lisa V. Rubenstein**. Yield of practice-based depression screening in VA primary care settings.. *Journal of General Internal Medicine*. 2012/03//. 27:331	Level 1, Form Title/abstract screen
2357	**Anja Koski-Jännes, Tanja Hirschovits-Gerz, Marjo Pennonen**. Population, professional, and client support for different models of managing addictive behaviors.. *Substance Use & Misuse*. 2012/02//. 47:296	Level 1, Form Title/abstract screen
2358	**Olivier Taïeb, Sylvie Chevret, Marie Rose Moro, Mitchell G. Weiss, Anne Biadi-Imhof, Aymeric Reyre, Thierry Baubet**. Impact of migration on explanatory models of illness and addiction severity in patients with drug dependence in a Paris suburb.. *Substance Use & Misuse*. 2012/02//. 47:347	Level 1, Form Title/abstract screen
2359	**Tammi Vacha-Haase**. Clinical practice with older adults.. *APA handbook of counseling psychology, Vol. 2: Practice, interventions, and applications.*. 2012///. #volume#:497	Level 1, Form Title/abstract screen
2360	**Laura Mufson, Laurie Reider Lewis, Meredith Gunlicks-Stoessel, Jami F. Young**. Treatment of adolescent depression with interpersonal psychotherapy.. *Casebook of interpersonal psychotherapy.*. 2012///. #volume#:203	Level 1, Form Title/abstract screen
2361	**Shelley Cohen Konrad, Jennifer Morton**. If I feel judged by you, I will not trust you: Relational practice with addicted mothers.. *Falling through the cracks: Psychodynamic practice with vulnerable and oppressed populations.*. 2012///. #volume#:107	Level 2, Form Full Text Screening

2362	**Brian K. Sullivan**. The one-stop shop: Collaboration beyond psychology.. *Getting better at private practice*.. 2012///. #volume#:292	Level 1, Form Title/abstract screen
2363	**Julian D. Ford**. Posttraumatic stress disorder among youth involved in juvenile justice.. *Handbook of juvenile forensic psychology and psychiatry*.. 2012///. #volume#:485	Level 1, Form Title/abstract screen
2364	**Mark B. Scholl, A. Scott McGowan, James T. Hansen**. Introduction to humanistic perspectives on contemporary counseling issues.. *Humanistic perspectives on contemporary counseling issues*.. 2012///. #volume#:3	Level 1, Form Title/abstract screen
2365	**Jane E. Myers, Phillip Clarke, Jennifer B. Brown, Denisha A. Champion**. Wellness: Theory, research, and applications for counselors.. *Humanistic perspectives on contemporary counseling issues*.. 2012///. #volume#:17	Level 2, Form Full Text Screening
2366	**Michael D'Andrea, Judy Daniels**. Humanism and multiculturalism.. *Humanistic perspectives on contemporary counseling issues*.. 2012///. #volume#:45	Level 1, Form Title/abstract screen
2367	**Mark B. Scholl, Emma Kendrick, Dallas Wilkes, W. Bryce Hagedorn**. Humanism and substance abuse counseling.. *Humanistic perspectives on contemporary counseling issues*.. 2012///. #volume#:85	Level 1, Form Title/abstract screen
2368	**Sylvia K. Fisher, Gary M. Blau, Jeffrey M. Poirier**. Where do we go from here? Next steps for research, practice, and policy.. *Improving emotional and behavioral outcomes for LGBT youth: A guide for professionals*.. 2012///. #volume#:267	Level 1, Form Title/abstract screen
2369	**Rodger Kessler**. An outcome and clinical research focus in an integrated care patient-centered medical home.. *Integrated care: Applying theory to practice*.. 2012///. #volume#:297	Level 1, Form Title/abstract screen
2370	**Frances E. Aboud**. Health psychology.. *Internationalizing the psychology curriculum in the United States*.. 2012///. #volume#:263	Level 1, Form Title/abstract screen
2371	**Maree Teeson, Wayne Hall, Louise Mewton, Margaret Grigg**. Substance-related disorders.. *Mental health in Australia: Collaborative community practice*.. 2012///. #volume#:694	Level 1, Form Title/abstract screen
2372	**Mark J. Bates, John C. Bradley, Nazanin Bahraini, Matthew N. Goldenberg**. Clinical management of suicide risk with military and veteran personnel.. *The American Psychiatric Publishing textbook of suicide assessment and management*.. 2012///. #volume#:40	Level 1, Form Title/abstract screen
2373	**John T. Maltsberger, Joseph B. Stoklosa**. Outpatient treatment.. *The American Psychiatric Publishing textbook of suicide assessment and management*.. 2012///. #volume#:303	Level 1, Form Title/abstract screen
2374	**Daniel J. Fischer, Theresa B. Moyers**. Motivational interviewing as a brief psychotherapy.. *The art and science of brief psychotherapies: An illustrated guide*.. 2012///. #volume#:27	Level 1, Form Title/abstract screen
2375	**Lisa Lopez Levers, Elizabeth M. Ventura, Demond E. Bledsoe**. Models for trauma intervention: Integrative approaches to therapy.. *Trauma counseling: Theories and interventions*.. 2012///. #volume#:493	Level 1, Form Title/abstract screen
2378	**Telsie A. Davis**. Removing a barrier to widen the door to recovery: Working alliance development with African American women substance abusers.. *Dissertation Abstracts International: Section B: The Sciences and Engineering*. 2012///. 73:612	Level 2, Form Full Text Screening

2379	**Susan E. Rosenkranz**. The contributions of psychological maltreatment and complex PTSD to the prediction of substance use problem severity among youth.. *Dissertation Abstracts International: Section B: The Sciences and Engineering*. 2012///. 73:631	Level 1, Form Title/abstract screen
2380	**Marsha Nneka Sargeant**. Psycholinguistic indicators of motivation for substance use behavior change among individuals with serious mental illness.. *Dissertation Abstracts International: Section B: The Sciences and Engineering*. 2012///. 73:1266	Level 1, Form Title/abstract screen
2381	**Nickeisha Clarke**. The effects of therapeutic alliance and client readiness to change on cognitive behavior therapy treatment outcomes for a sample of substance and non-substance abusing psychiatric inpatient women.. *Dissertation Abstracts International: Section B: The Sciences and Engineering*. 2012///. 73:1843	Level 1, Form Title/abstract screen
2382	**Lindsey W. North**. Working alliance and outcome in adolescents and adults in treatment for substance abuse.. *Dissertation Abstracts International: Section B: The Sciences and Engineering*. 2012///. 73:2513	Level 2, Form Full Text Screening
2383	**Claudette Voelkel Fette**. School-based occupational therapy: Perspectives on strength-based assessment and providing related interventions for elementary students with mental health needs.. *Dissertation Abstracts International: Section B: The Sciences and Engineering*. 2012///. 73:3544	Level 1, Form Title/abstract screen
2384	**Lindsay C. Sharp**. Older adult patient-doctor communication regarding alcohol use: A qualitative study.. *Dissertation Abstracts International: Section B: The Sciences and Engineering*. 2012///. 72:6707	Level 1, Form Title/abstract screen
2385	**Jon M. Houck**. The neuroscience of motivational interviewing change talk.. *Dissertation Abstracts International: Section B: The Sciences and Engineering*. 2012///. 72:7707	Level 1, Form Title/abstract screen
2386	**Patt Denning, Jeannie Little**. Practicing harm reduction psychotherapy: An alternative approach to addictions., 2nd ed.. *#journal#*. 2012///. #volume#:#pages#	Level 2, Form Full Text Screening
2388	**Louise Johns**. Review of CBT for psychosis: A symptom-based approach.. *Cognitive Behaviour Therapy*. 2011/12//. 40:313	Level 1, Form Title/abstract screen
2389	**Geoffrey M. Curran, Jeffrey Pyne, John C. Fortney, Allen Gifford, Stephen M. Asch, David Rimland, Maria Rodriguez-Barradas, Thomas P. Monson, Amy M. Kilbourne, Hilde Hagedorn, Joseph Atkinson**. Development and implementation of collaborative care for depression in HIV clinics.. *AIDS Care*. 2011/12//. 23:1626	Level 1, Form Title/abstract screen
2390	**Samantha S. Yard, David Huh, Kevin M. King, Jane M. Simoni**. Patient-level moderators of the efficacy of peer support and pager reminder interventions to promote antiretroviral adherence.. *AIDS and Behavior*. 2011/11//. 15:1596	Level 1, Form Title/abstract screen
2391	**Bernadette Pereira, Gracy Andrew, Sulochana Pednekar, Betty R Kirkwood, Vikram Patel**. The integration of the treatment for common mental disorders in primary care: Experiences of health care providers in the MANAS trial in Goa, India.. *International Journal of Mental Health Systems*. 2011/10/03/. 5:#pages#	Level 1, Form Title/abstract screen
2395	**Racquel J. Merritt, Mary S. Jackson, Sara M. Bunn, Lashaunda R. Joyner**. Substance abuse prevention interventions for pregnant women: A synopsis of a need for collaborative work.. *Journal of Human Behavior in the Social Environment*. 2011/10//. 21:858	Level 1, Form Title/abstract screen

2396	**Kathryn Corson, Melanie N. Doak, Lauren Denneson, Megan Crutchfield, Geoffrey Soleck, Kathryn C. Dickinson, Martha S. Gerrity, Steven K. Dobscha**. Primary care clinician adherence to guidelines for the management of chronic musculoskeletal pain: Results from the study of the effectiveness of a collaborative approach to pain.. *Pain Medicine*. 2011/10//. 12:1490	Level 1, Form Title/abstract screen
2399	**Kathleen Ell, Wayne Katon, Bin Xie, Pey-Jiuan Lee, Suad Kapetanovic, Jeffrey Guterman, Chih-Ping Chou**. One-year postcollaborative depression care trial outcomes among predominantly Hispanic diabetes safety net patients.. *General Hospital Psychiatry*. 2011/09//Sep-Oct, 2011. 33:436	Level 1, Form Title/abstract screen
2401	**Kathryn Sheridan, Wendy L. Haight, Leah Cleeland**. The role of grandparents in preventing aggressive and other externalizing behavior problems in children from rural, methamphetamine-involved families.. *Children and Youth Services Review*. 2011/09//. 33:1583	Level 1, Form Title/abstract screen
2405	**Ethan S. Rofman**. Review of Primary care mental health.. *The Journal of Clinical Psychiatry*. 2011/07//. 72:1018	Level 1, Form Title/abstract screen
2407	**Monica Payne, Megan Gething, Alison A. Moore, M. Carrington Reid**. Primary care providers' perspectives on psychoactive medication disorders in older adults.. *American Journal of Geriatric Pharmacotherapy (AJGP)*. 2011/06//. 9:164	Level 1, Form Title/abstract screen
2408	**Paul Crits-Christoph, Jessica L. Hamilton, Sarah Ring-Kurtz, Robert Gallop, Bridget McClure, Agatha Kulaga, John Rotrosen**. Program, counselor, and patient variability in the alliance: A multilevel study of the alliance in relation to substance use outcomes.. *Journal of Substance Abuse Treatment*. 2011/06//. 40:405	Level 2, Form Full Text Screening
2410	**Elizabeth Evans, M. Douglas Anglin, Darren Urada, Joy Yang**. Promising practices for delivery of court-supervised substance abuse treatment: Perspectives from six high-performing California counties operating Proposition 36.. *Evaluation and Program Planning*. 2011/05//. 34:124	Level 1, Form Title/abstract screen
2413	**Paul Crits-Christoph, Jennifer Johnson, Robert Gallop, Mary Beth Connolly Gibbons, Sarah Ring-Kurtz, Jessica L. Hamilton, Xin Tu**. A generalizability theory analysis of group process ratings in the treatment of cocaine dependence.. *Psychotherapy Research*. 2011/05//. 21:252	Level 1, Form Title/abstract screen
2418	**Branka Agic, Robert E. Mann, Marianne Kobus-Matthews**. Alcohol use in seven ethnic communities in Ontario: A qualitative investigation.. *Drugs: Education, Prevention & Policy*. 2011/04//. 18:116	Level 1, Form Title/abstract screen
2419	**Rogério M. Pinto, Aimee N. C. Campbell, Denise A. Hien, Gary Yu, Prakash Gorroochurn**. Retention in the National Institute on Drug Abuse Clinical Trials Network Women and Trauma Study: Implications for posttrial implementation.. *American Journal of Orthopsychiatry*. 2011/04//. 81:211	Level 1, Form Title/abstract screen
2421	**Ihsan M. Salloum, Juan E. Mezzich**. Outlining the bases of person‐centred integrative diagnosis.. *Journal of Evaluation in Clinical Practice*. 2011/04//. 17:354	Level 1, Form Title/abstract screen
2422	**Kim J. Masters**. 'Ultrashort stays and a focus on recovery': To the editor.. *Psychiatric Services*. 2011/04//. 62:434	Level 1, Form Title/abstract screen
2424	**Douglas Zatzick, Frederick Rivara, Gregory Jurkovich, Joan Russo, Sarah Geiss Trusz, Jin Wang, Amy Wagner, Kari Stephens, Chris Dunn, Edwina Uehara, Megan Petrie, Charles Engel, Dimitri Davydow, Wayne Katon**.	Level 1, Form Title/abstract screen

	Enhancing the population impact of collaborative care interventions: Mixed method development and implementation of stepped care targeting posttraumatic stress disorder and related comorbidities after acute trauma.. *General Hospital Psychiatry*. 2011/03//Mar-Apr, 2011. 33:123	
2426	**Anne Kari Knudsen, Cinzia Brunelli, Stein Kaasa, Giovanni Apolone, Oscar Corli, Mauro Montanari, Robin Fainsinger, Nina Aass, Peter Fayers, Augusto Caraceni, Pål Klepstad**. Which variables are associated with pain intensity and treatment response in advanced cancer patients? – implications for a future classification system for cancer pain.. *European Journal of Pain*. 2011/03//. 15:320	Level 1, Form Title/abstract screen
2428	**Henny A. Westra, Hal Arkowitz**. Introduction.. *Cognitive and Behavioral Practice*. 2011/02//. 18:1	Level 1, Form Title/abstract screen
2429	**Charles Nelson, Kate St. Cyr, Margaret Weiser, Shannon Gifford, Jane Gallimore, Andrew Morningstar**. Knowledge gained from the Traumatic Brain Injury Screen—Implications for treating Canadian military personnel.. *Military Medicine*. 2011/02//. 176:156	Level 1, Form Title/abstract screen
2430	**Kathleen Ell, Bin Xie, Suad Kapetanovic, David I. Quinn, Pey-Jiuan Lee, Anjanette Wells, Chih-Ping Chou**. One-year follow-up of collaborative depression care for low-income, predominantly Hispanic patients with cancer.. *Psychiatric Services*. 2011/02//. 62:162	Level 1, Form Title/abstract screen
2431	**Amo Fuhrmann, Bemhard M. Schroer, Renate de Jong-Meyer**. Systematic motivational counseling in groups: Promoting therapeutic change through client interaction.. *Handbook of motivational counseling: Goal-based approaches to assessment and intervention with addiction and other problems*.. 2011///. #volume#:303	Level 1, Form Title/abstract screen
2432	**Michael R. Tilus, Kevin M. McGuinness, Mimi Sa, Earl Sutherland, Bret A. Moore, Vincen Barnes, Johna C. Hartnell, Anthony Tranchita**. Collaborative practice with pediatricians within the Indian Health Service: Taking care of frontier children.. *Pediatricians and pharmacologically trained psychologists: Practitioner's guide to collaborative treatment*.. 2011///. #volume#:95	Level 1, Form Title/abstract screen
2434	**Betty Vreeland, Anna Marie Toto, Marie Verna, Jill Williams**. Cultivating physical health and wellness utilizing a person-centered approach.. *Serious mental illness: Person-centered approaches*.. 2011///. #volume#:284	Level 1, Form Title/abstract screen
2435	**Leon Ginsberg**. Editorial note.. *Administration in Social Work*. 2011/01//. 35:1	Level 1, Form Title/abstract screen
2437	**A. L. Roberts, S. E. Gilman, J. Breslau, N. Breslau, K. C. Koenen**. Race/ethnic differences in exposure to traumatic events, development of post-traumatic stress disorder, and treatment-seeking for post-traumatic stress disorder in the United States.. *Psychological Medicine*. 2011/01//. 41:71	Level 1, Form Title/abstract screen
2438	**Laurie Drabble**. Advancing collaborative practice between substance abuse treatment and child welfare fields: What helps and hinders the process?. *Administration in Social Work*. 2011/01//. 35:88	Level 1, Form Title/abstract screen
2439	**Lisa D . Butler, Filomena M . Critelli, Elaine S. Rinfrette**. Trauma-informed care and mental health.. *Directions in Psychiatry*. 2011///. 31:197	Level 1, Form Title/abstract screen
2440	**David Menges**. The role of therapeutic alliance in individual and couples cognitive-behavioral therapy for women with alcohol dependence.. *Dissertation*	Level 1, Form Title/abstract

	Abstracts International Section A: Humanities and Social Sciences. 2011///. 72:1108	screen
2441	**Harriett Elizabeth Reeh**. The relationships between perceived therapeutic alliance, therapist self-disclosure, and dropout expectancy among male substance abuse treatment participants.. *Dissertation Abstracts International: Section B: The Sciences and Engineering*. 2011///. 72:1150	Level 2, Form Full Text Screening
2442	**Ashley C. Lyman**. Experienced clinicians' perspectives on dual diagnosis: A qualitative investigation.. *Dissertation Abstracts International: Section B: The Sciences and Engineering*. 2011///. 72:1799	Level 1, Form Title/abstract screen
2443	**Allison J. Applebaum**. Correlates of neuropsychological functioning and associations with therapeutic alliance in HIV-infected and HIV-uninfected opiate-dependent patients.. *Dissertation Abstracts International: Section B: The Sciences and Engineering*. 2011///. 72:2430	Level 1, Form Title/abstract screen
2444	**Marina Lalayants**. Multidisciplinary clinical consultation in child protection: Contextual influences and stakeholder perceptions of best practices.. *Dissertation Abstracts International Section A: Humanities and Social Sciences*. 2011///. 71:3040	Level 1, Form Title/abstract screen
2445	**Masha Godkin**. A case study of the treatment team approach in addiction.. *Dissertation Abstracts International: Section B: The Sciences and Engineering*. 2011///. 71:5122	Level 1, Form Title/abstract screen
2446	**Tracey Cherie Gilbert**. Coping, spirituality, motivation to change, and the working alliance: The associations with substance abuse treatment outcomes.. *Dissertation Abstracts International: Section B: The Sciences and Engineering*. 2011///. 71:5788	Level 1, Form Title/abstract screen
2447	**Donna M. Sudak**. Combining CBT and medication: An evidence-based approach.. *#journal#*. 2011///. #volume#:#pages#	Level 1, Form Title/abstract screen
2451	**Steve Sussman**. Introduction to issue on special populations: Persons, places, and situations.. *Evaluation & the Health Professions*. 2010/12//. 33:412	Level 1, Form Title/abstract screen
2453	**Rowena J. Dolor, Truls Østbye, Pauline Lyna, Cynthia J. Coffman, Stewart C. Alexander, James A. Tulsky, Rebecca J. Namenek Brouwer, Iguehi Esoimeme, Kathryn I. Pollak**. What are physicians' and patients' beliefs about diet, weight, exercise, and smoking cessation counseling?. *Preventive Medicine: An International Journal Devoted to Practice and Theory*. 2010/11//. 51:440	Level 1, Form Title/abstract screen
2457	**Julie Kipp**. Review of Ordinary life therapy: Experiences from a collaborative systemic practice.. *Psychosis: Psychological, Social and Integrative Approaches*. 2010/10//. 2:261	Level 1, Form Title/abstract screen
2458	**Alicia L. Picken, Katherine Berry, Nicholas Tarrier, Christine Barrowclough**. Traumatic events, posttraumatic stress disorder, attachment style, and working alliance in a sample of people with psychosis.. *Journal of Nervous and Mental Disease*. 2010/10//. 198:775	Level 1, Form Title/abstract screen
2459	**Mehrnaz Davoudi, Richard A. Rawson**. Screening, brief intervention, and referral to treatment (SBIRT) initiatives in California: Notable trends, challenges, and recommendations.. *Journal of Psychoactive Drugs*. 2010/09//. Sarc Suppl 6:239	Level 1, Form Title/abstract screen
2464	**Kathleen Ell, María P. Aranda, Bin Xie, Pey-Jiuan Lee, Chih-Ping Chou**. Collaborative depression treatment in older and younger adults with physical illness: Pooled comparative analysis of three randomized clinical trials.. *The*	Level 1, Form Title/abstract screen

	American Journal of Geriatric Psychiatry. 2010/06//. 18:520	
2466	**Eric Latimer, Olivier Farmer, Anne G. Crocker, Todd Jenkins**. Perceived coercion, client-centredness, and positive and negative pressures in an assertive community treatment program: An exploratory study.. *Canadian Journal of Community Mental Health*. 2010///Spr 2010. 29:35	Level 1, Form Title/abstract screen
2469	**Steven D. Vannoy, Patricia Arean, Jürgen Unützer**. Advantages of using estimated depression-free days for evaluating treatment efficacy.. *Psychiatric Services*. 2010/02//. 61:160	Level 1, Form Title/abstract screen
2470	**Niklaus Stulz, Robert Gallop, Wolfgang Lutz, Glenda L. Wrenn, Paul Crits-Christoph**. Examining differential effects of psychosocial treatments for cocaine dependence: An application of latent trajectory analyses.. *Drug and Alcohol Dependence*. 2010/01/15/. 106:164	Level 1, Form Title/abstract screen
2471	**Meredith L. Gunlicks-Stoessel, Laura Mufson**. Interpersonal psychotherapy for depressed adolescents.. *Dulcan's textbook of child and adolescent psychiatry.*. 2010///. #volume#:887	Level 1, Form Title/abstract screen
2472	**Paul Nagy**. Motivational interviewing.. *Dulcan's textbook of child and adolescent psychiatry.*. 2010///. #volume#:915	Level 1, Form Title/abstract screen
2473	**Leanne Hides, Steve Carroll, Dan I. Lubman, Amanda Baker**. Brief motivational interviewing for depression and anxiety.. *Oxford guide to low intensity CBT interventions.*. 2010///. #volume#:177	Level 1, Form Title/abstract screen
2474	**Gary M. Blau, Shannon CrossBear, Coretta Mallery**. Adaptation and innovation.. *The leadership equation: Strategies for individuals who are champions for children, youth, and families.*. 2010///. #volume#:141	Level 1, Form Title/abstract screen
2475	**Catherine Eubanks-Carter, J. Christopher Muran, Jeremy D. Safran**. Alliance ruptures and resolution.. *The therapeutic alliance: An evidence-based guide to practice.*. 2010///. #volume#:74	Level 1, Form Title/abstract screen
2476	**Lorna Smith Benjamin, Kenneth L. Critchfield**. An interpersonal perspective on therapy alliances and techniques.. *The therapeutic alliance: An evidence-based guide to practice.*. 2010///. #volume#:123	Level 1, Form Title/abstract screen
2479	**Paul Branscum, Manoj Sharma**. A review of motivational interviewing-based interventions targeting problematic drinking among college students.. *Alcoholism Treatment Quarterly*. 2010/01//. 28:63	Level 1, Form Title/abstract screen
2482	**Brian D. Roland**. The impact of counselor recovery status, disclosure, education, and experience on the working alliance in the treatment of substance use disorders.. *Dissertation Abstracts International Section A: Humanities and Social Sciences*. 2010///. 71:1798	Level 1, Form Title/abstract screen
2483	**Margaret M. Murray**. Opportunistic interventions to treat alcohol problems in emergency department settings: Is there a role for social work and other non-physician health professions?. *Dissertation Abstracts International: Section B: The Sciences and Engineering*. 2010///. 71:3588	Level 1, Form Title/abstract screen
2484	**Nazli Seewer**. The effects of adult attachment styles on treatment outcome of methamphetamine abusers.. *Dissertation Abstracts International: Section B: The Sciences and Engineering*. 2010///. 70:5846	Level 1, Form Title/abstract screen
2485	**Robert A. DiTomasso, Barbara A. Golden, Harry Morris**. Handbook of cognitive behavioral approaches in primary care.. *#journal#*. 2010///. #volume#:#pages#	Level 1, Form Title/abstract screen
2486	**Katherine van Wormer, Bruce A. Thyer**. Evidence-based practice in the field	Level 2, Form Full Text

	of substance abuse: A book of readings.. *#journal#*. 2010///. #volume#:#pages#	Screening
2487	**Neeraj K. Arora, Richard L. Jr. Street, Ronald M. Epstein, Phyllis N. Butow**. Facilitating patient-centered cancer communication: A road map.. *Patient Education and Counseling*. 2009/12//. 77:319	Level 1, Form Title/abstract screen
2488	**Henri Gomez**. La fonction soignante en alcoologie. = Healthcare function in alcohol rehabilitation.. *Alcoologie et Addictologie*. 2009/12//. 31:337	Level 1, Form Title/abstract screen
2491	**Margaret Pereyra, Lisa R. Metsch, Lauren Gooden**. HIV-positive patients' discussion of oral health with their HIV primary care providers in Miami, Florida.. *AIDS Care*. 2009/12//. 21:1578	Level 1, Form Title/abstract screen
2492	**Domin Chan, Allen D. Cheadle, Gayle Reiber, Jürgen Unützer, Edmund F. Chaney**. Health care utilization and its costs for depressed veterans with and without comorbid PTSD symptoms.. *Psychiatric Services*. 2009/12//. 60:1612	Level 1, Form Title/abstract screen
2493	**Jean Sandra Adams, Sandra Steele, Alyson McGregor Kettles, Helen Walker, Ian Brown, Mick Collins, Susan Sookoo, Phil Woods**. Experience of collaborative research practice in forensic mental health.. *The British Journal of Forensic Practice*. 2009/11//. 11:4	Level 1, Form Title/abstract screen
2494	**Michael R. Clark**. Psychiatry and chronic pain: Examining the interface and designing a structure for a patient-center approach to treatment.. *European Journal of Pain Supplements*. 2009/11//. 3:95	Level 1, Form Title/abstract screen
2495	**Christopher C. Wagner, Karen S. Ingersoll**. Beyond behavior: Eliciting broader change with motivational interviewing.. *Journal of Clinical Psychology*. 2009/11//. 65:1180	Level 1, Form Title/abstract screen
2496	**Jeffrey A. Buck**. Recent changes in Medicaid policy and their possible effects on mental health services.. *Psychiatric Services*. 2009/11//. 60:1504	Level 1, Form Title/abstract screen
2498	**Leigh Gemmell, Carlo C. DiClemente**. Styles of physician advice about smoking cessation in college students.. *Journal of American College Health*. 2009/09//Sep-Oct, 2009. 58:113	Level 1, Form Title/abstract screen
2499	**Michael J. Silverman**. The effect of lyric analysis on treatment eagerness and working alliance in consumers who are in detoxification: A randomized clinical effectiveness study.. *Music Therapy Perspectives*. 2009/09//. 27:115	Level 1, Form Title/abstract screen
2500	**William R. Miller, Gary S. Rose**. Toward a theory of motivational interviewing.. *American Psychologist*. 2009/09//. 64:527	Level 1, Form Title/abstract screen
2501	**Steve J. Allsop, Clare F. Stevens**. Evidence-based practice or imperfect seduction? Developing capacity to respond effectively to drug-related problems.. *Drug and Alcohol Review*. 2009/09//. 28:541	Level 1, Form Title/abstract screen
2503	**Thomas O'Hare, Margaret Sherrer**. Effects of clinic staff support on psychosocial well-being and PTSD symptom severity in clients with severe mental illnesses.. *Best Practices in Mental Health: An International Journal*. 2009/07//. 5:1	Level 1, Form Title/abstract screen
2505	**Joan Mitchell, Judith Belle Brown, Carrie Smith**. Interprofessional education: A nurse practitioner impacts family medicine residents' smoking cessation counselling experiences.. *Journal of Interprofessional Care*. 2009/07//. 23:401	Level 1, Form Title/abstract screen
2506	**Nevin J. Harper**. The relationship of therapeutic alliance to outcome in wilderness treatment.. *Journal of Adventure Education and Outdoor Learning*.	Level 1, Form Title/abstract

	2009/06//. 9:45	screen
2507	**Martin C. Wesley, Neresa B. Minatrea, Joshua C. Watson**. Animal-assisted therapy in the treatment of substance dependence.. *Anthrozoös*. 2009/06//. 22:137	Level 2, Form Full Text Screening
2509	**Judith K. Ockene, George W. Reed, Sarah Reiff-Hekking**. Brief patient-centered clinician-delivered counseling for high-risk drinking: 4-year results.. *Annals of Behavioral Medicine*. 2009/06//. 37:335	Level 2, Form Full Text Screening
2510	**Ora Nakash, Sarah Dargouth, Vanessa Oddo, Shan Gao, Margarita Alegría**. Patient initiation of information: Exploring its role during the mental health intake visit.. *Patient Education and Counseling*. 2009/05//. 75:220	Level 1, Form Title/abstract screen
2511	**J. Law, C. Plunkett, J. Taylor, M. Gunning**. Developing policy in the provision of parenting programmes: Integrating a review of reviews with the perspectives of both parents and professionals.. *Child: Care, Health and Development*. 2009/05//. 35:302	Level 1, Form Title/abstract screen
2517	**Wadih Maalouf, Cynthia L. Arfken**. Guest editors' introduction: Assessing the problem of substance abuse in the Arab world.. *Journal of Muslim Mental Health*. 2009/03//. 4:5	Level 1, Form Title/abstract screen
2518	**Chuan-Fen Liu, Cory Bolkan, Domin Chan, Elizabeth M. Yano, Lisa V. Rubenstein, Edmund F. Chaney**. Dual use of VA and non-VA services among primary care patients with depression.. *Journal of General Internal Medicine*. 2009/03//. 24:305	Level 1, Form Title/abstract screen
2519	**Marie Borum, Mary Reyes, Huy Nguyen**. Providing counseling on alcohol use for patients with liver disease: Another missed opportunity.. *Patient Education and Counseling*. 2009/02//. 74:277	Level 1, Form Title/abstract screen
2520	**Rudolf H. Moos**. Addictive disorders in context: Principles and puzzles of effective treatment and recovery.. *Addictive behaviors: New readings on etiology, prevention, and treatment.*. 2009///. #volume#:537	Level 1, Form Title/abstract screen
2521	**Virginia Hill Rice**. Monitoring the tobacco epidemic with national, regional, and international databases and systematic reviews: Evidence for nursing research and clinical decision making.. *Annual review of nursing research, 2009: Advancing nursing science in tobacco control.*. 2009///. 27:91	Level 1, Form Title/abstract screen
2522	**Eric R. Levensky, Brian C. Kersh, Lavina L. Cavasos, J. Annette Brooks**. Motivational interviewing.. *General principles and empirically supported techniques of cognitive behavior therapy.*. 2009///. #volume#:455	Level 1, Form Title/abstract screen
2523	**Peter H. Musser, Joshua N. Semiatin, Casey T. Taft, Christopher M. Murphy**. Motivational interviewing as a pregroup intervention for partner-violent men.. *Motivational interviewing and stages of change in intimate partner violence.*. 2009///. #volume#:61	Level 1, Form Title/abstract screen
2524	**James R. McKay**. Other developments in disease management for substance use disorders.. *Treating substance use disorders with adaptive continuing care.*. 2009///. #volume#:191	Level 2, Form Full Text Screening
2528	**Glenn Roberts**. Review of Principles and practice of psychiatric rehabilitation: An empirical approach.. *The British Journal of Psychiatry*. 2009/01//. 194:93	Level 1, Form Title/abstract screen
2530	**Kathleen Ell, Wayne Katon, Leopoldo J. Cabassa, Bin Xie, Pey-Jiuan Lee, Suad Kapetanovic, Jeffry Guterman**. Depression and diabetes among low-income Hispanics: Design elements of a socio-culturally adapted collaborative care model randomized controlled trial.. *International Journal of Psychiatry in Medicine*. 2009///. 39:113	Level 1, Form Title/abstract screen

2531	**Larry F. Forthun, Marilyn J. Montgomery**. Profiles of adolescent identity development: Response to an intervention for alcohol/other drug problems.. *Alcoholism Treatment Quarterly*. 2009///. 27:132	Level 1, Form Title/abstract screen
2532	**Ellen T. Bartley**. Family stability, social support and working alliance with a clinical case manager as predictors of exits from homelessness among the mentally ill.. *Dissertation Abstracts International Section A: Humanities and Social Sciences*. 2009///. 70:688	Level 1, Form Title/abstract screen
2533	**Nyasanu A. Barbee**. The clinical implications of racial differences and inpatient psychological outcome in a sample of veterans.. *Dissertation Abstracts International: Section B: The Sciences and Engineering*. 2009///. 69:6399	Level 1, Form Title/abstract screen
2534	**Peter Berzins**. Therapeutic alliance as a predictor of psychotherapy process and outcome: The role of expert versus novice raters.. *Dissertation Abstracts International: Section B: The Sciences and Engineering*. 2009///. 69:7129	Level 1, Form Title/abstract screen
2535	**Jennifer Fende Guajardo**. The effects of pretreatment preparation with clients in a substance abuse treatment program.. *Dissertation Abstracts International: Section B: The Sciences and Engineering*. 2009///. 69:7810	Level 1, Form Title/abstract screen
2536	**Ricky Greenwald**. Treating problem behaviors: A trauma-informed approach.. *#journal#*. 2009///. #volume#:#pages#	Level 1, Form Title/abstract screen
2537	**Linda Frisman, Michael Prendergast, Hsiu-Ju Lin, Eleni Rodis, Lisa Greenwell**. Applying classification and regression tree analysis to identify prisoners with high HIV risk behaviors.. *Journal of Psychoactive Drugs*. 2008/12//. 40:447	Level 1, Form Title/abstract screen
2540	**Vivian B. Brown, Lisa A. Melchior**. Women with co-occuring disorders (COD): Treatment settings and service needs.. *Journal of Psychoactive Drugs*. 2008/11//. Sarc Suppl 5:365	Level 2, Form Full Text Screening
2542	**John W. Jr. Williams, J. Sloan Manning**. 'Collaborative mental health and primary care for bipolar disorder': Erratum.. *Journal of Psychiatric Practice*. 2008/11//. 14:411	Level 1, Form Title/abstract screen
2543	**Rose C. Maly, Judith A. Stein, Yoshiko Umezawa, Barbara Leake, M. Douglas Anglin**. Racial/ethnic differences in breast cancer outcomes among older patients: Effects of physician communication and patient empowerment.. *Health Psychology*. 2008/11//. 27:728	Level 1, Form Title/abstract screen
2550	**Ashley Christiani, Angela L. Hudson, Adeline Nyamathi, Malaika Mutere, Jeffrey Sweat**. Attitudes of homeless and drug-using youth regarding barriers and facilitators in delivery of quality and culturally sensitive health care.. *Journal of Child and Adolescent Psychiatric Nursing*. 2008/08//. 21:154	Level 1, Form Title/abstract screen
2552	**Heather Liszka Rose, Peter M. Miller, Lynne S. Nemeth, Ruth G. Jenkins, Paul J. Nietert, Andrea M. Wessell, Steven Ornstein**. Alcohol screening and brief counseling in a primary care hypertensive population: A quality improvement intervention.. *Addiction*. 2008/08//. 103:1271	Level 1, Form Title/abstract screen
2553	**Carol E. Blixen, Noah J. Webster, Andrew J. Hund, Adam T. Perzynski, Stephanie W. Kanuch, Eleanor Palo Stoller, Richard A. McCormick, Neal V. Dawson**. Communicating about alcohol consumption to nonharmful drinkers with hepatitis C: Patient and provider perspectives.. *Journal of General Internal Medicine*. 2008/08//. 23:1290	Level 1, Form Title/abstract screen
2554	**Marja Humphrey**. Review of Collaborative treatment of traumatized children and teens: The trauma systems therapy approach.. *The Family Journal*. 2008/07//. 16:275	Level 1, Form Title/abstract screen

2557	**Paul Crits-Christoph, Mary Beth Connolly Gibbons, Robert Gallop, Sarah Ring-Kurtz, Jacques P. Barber, Matthew Worley, Julie Present, Bridget Hearon**. Supportive-expressive psychodynamic therapy for cocaine dependence: A closer look.. *Psychoanalytic Psychology*. 2008/07//. 25:483	Level 1, Form Title/abstract screen
2559	**John Greist**. A promising debut for computerized therapies.. *The American Journal of Psychiatry*. 2008/07//. 165:793	Level 1, Form Title/abstract screen
2563	**Constance H. Fung, Claude M. Setodji, Fuan-Yue Kung, Joan Keesey, Steven M. Asch, John Adams, Elizabeth A. McGlynn**. The relationship between multimorbidity and patients' ratings of communication.. *Journal of General Internal Medicine*. 2008/06//. 23:788	Level 1, Form Title/abstract screen
2565	**Bauke Koekkoek, John G. Gunderson, Ad Kaasenbrood, Thomas G. Gutheil**. Chronic suicidality in a physician: An alliance yet to become therapeutic.. *Harvard Review of Psychiatry*. 2008/05//. 16:195	Level 1, Form Title/abstract screen
2567	**Stian Biong, Bengt Karlsson, Tommy Svensson**. Metaphors of a shifting sense of self in men recovering from substance abuse and suicidal behavior.. *Journal of Psychosocial Nursing and Mental Health Services*. 2008/04//. 46:35	Level 1, Form Title/abstract screen
2568	**John E. Zeber, Laurel A. Copeland, Chester B. Good, Michael J. Fine, Mark S. Bauer, Amy M. Kilbourne**. Therapeutic alliance perceptions and medication adherence in patients with bipolar disorder.. *Journal of Affective Disorders*. 2008/04//. 107:53	Level 1, Form Title/abstract screen
2569	**Jennifer Wisdom, Roy Gabriel, Eldon Edmundson, Sarann Bielavitz, Joe Hromco**. Challenges substance abuse treatment agencies faced in adoption of computer-based technology to improve assessment.. *The Journal of Behavioral Health Services & Research*. 2008/04//. 35:158	Level 1, Form Title/abstract screen
2572	**Janie Sheridan, Felicity Goodyear-Smith, Rachael Butler, Amanda Wheeler, Annette Gohns**. Barriers to, and incentives for, the transfer of opioid-dependent people on methadone maintenance treatment from secondary care to primary health care.. *Drug and Alcohol Review*. 2008/03//. 27:178	Level 1, Form Title/abstract screen
2573	**Brenda D. Smith, Cristina Mogro-Wilson**. Inter-agency collaboration: Policy and practice in child welfare and substance abuse treatment.. *Administration in Social Work*. 2008/02//. 32:5	Level 1, Form Title/abstract screen
2575	**Cory F. Newman**. Substance use disorders.. *Adapting cognitive therapy for depression: Managing complexity and comorbidity*.. 2008///. #volume#:233	Level 1, Form Title/abstract screen
2577	**Rodger Kessler, Dale Stafford**. Introduction.. *Collaborative medicine case studies: Evidence in practice*.. 2008///. #volume#:3	Level 1, Form Title/abstract screen
2578	**Rodger Kessler, Dale Stafford**. Primary care is the de facto mental health system.. *Collaborative medicine case studies: Evidence in practice*.. 2008///. #volume#:9	Level 1, Form Title/abstract screen
2579	**Rodger Kessler**. How I learned about integrated care by failing miserably: The deadly sins of integration.. *Collaborative medicine case studies: Evidence in practice*.. 2008///. #volume#:39	Level 1, Form Title/abstract screen
2580	**Parinda Khatri, Gregg Perry, Febe Wallace**. Walking the tightrope without a net: Integrated care for the patient with diabetes, cardiovascular disease, and bipolar disorder...And no insurance.. *Collaborative medicine case studies: Evidence in practice*.. 2008///. #volume#:309	Level 1, Form Title/abstract screen
2581	**Shelly A. Wiechelt, Wendy Lutz, Nancy J. Smyth, Charles Syms**. Integrating	Level 1, Form

	research and practice: A collaborative model for addressing trauma and addiction.. *Stress, trauma and substance use*.. 2008///. #volume#:101	Title/abstract screen
2582	**David W. Brook**. Group therapy.. *The American Psychiatric Publishing textbook of substance abuse treatment*.. 2008///. #volume#:413	Level 1, Form Title/abstract screen
2583	**Bonita M. Veysey**. Mental health, substance abuse, and trauma.. *Treating the juvenile offender*.. 2008///. #volume#:210	Level 1, Form Title/abstract screen
2584	**Stanley Sacks, Karen McKendrick, JoAnn Y. Sacks, Steven Banks, Michael Harle**. Enhanced outpatient treatment for co-occurring disorders: Main outcomes.. *Journal of Substance Abuse Treatment*. 2008/01//. 34:48	Level 1, Form Title/abstract screen
2585	**Maite P. Mena, Victoria B. Mitrani, Joan A. Muir, Daniel A. Santisteban**. Extended parent-child separations: Impact on substance-abusing Hispanic adolescents.. *Journal for Specialists in Pediatric Nursing*. 2008/01//. 13:50	Level 1, Form Title/abstract screen
2587	**Jo Brocato, Eric F. Wagner**. Predictors of retention in an alternative-to-prison substance abuse treatment program.. *Criminal Justice and Behavior*. 2008/01//. 35:99	Level 1, Form Title/abstract screen
2589	**Diana Marie Hess**. An investigation of the counseling needs of adult female Mexican Americans with low income status: A modified Delphi study.. *Dissertation Abstracts International Section A: Humanities and Social Sciences*. 2008///. 69:1283	Level 1, Form Title/abstract screen
2590	**Sarah Watson Feldstein**. Motivational interviewing with late-adolescent/college underage drinkers: An investigation of therapeutic alliance.. *Dissertation Abstracts International: Section B: The Sciences and Engineering*. 2008///. 68:4821	Level 1, Form Title/abstract screen
2591	**Elissa Mccarthy**. Role of the case management relationship in helping homeless mothers with addictive disorders.. *Dissertation Abstracts International: Section B: The Sciences and Engineering*. 2008///. 68:4834	Level 2, Form Full Text Screening
2592	**Bradley Roger Brummett**. Attachment style, early maladaptive schemas, coping self-efficacy, therapy alliance and their influence on addiction severity in methadone-maintenance treatment.. *Dissertation Abstracts International: Section B: The Sciences and Engineering*. 2008///. 68:6952	Level 1, Form Title/abstract screen
2593	**Stephen Rollnick, William R. Miller, Christopher C. Butler**. Motivational interviewing in health care: Helping patients change behavior.. *#journal#*. 2008///. #volume#:#pages#	Level 1, Form Title/abstract screen
2594	**Kenneth W. Wanberg, Harvey B. Milkman**. Criminal conduct and substance abuse treatment: Strategies for self-improvement and change, pathways to responsible living: The provider's guide., 2nd ed.. *#journal#*. 2008///. #volume#:#pages#	Level 1, Form Title/abstract screen
2595	**James L. Levenson**. Psychosomatic medicine: Future tasks and priorities for the new psychiatric subspecialty.. *Revista Brasileira de Psiquiatria*. 2007/12//. 29:301	Level 1, Form Title/abstract screen
2596	**Robert Forman, Paul Crits-Christoph, Övgü Kaynak, Matt Worley, Donald A. Hantula, Agatha Kulaga, John Rotrosen, Melissa Chu, Robert Gallop, Jennifer Potter, Patrice Muchowski, Kirk Brower, Stephen Strobbe, Kathy Magruder, A'Delle H. Chellis, Tad Clodfelter, Margaret Cawley**. A feasibility study of a web-based performance improvement system for substance abuse treatment providers.. *Journal of Substance Abuse Treatment*. 2007/12//. 33:363	Level 1, Form Title/abstract screen

2599	**Georgiana Shick Tryon, Sasha Collins Blackwell, Elizabeth Felleman Hammel**. A meta-analytic examination of client-therapist perspectives of the working alliance.. *Psychotherapy Research*. 2007/11//. 17:629	Level 1, Form Title/abstract screen
2600	**Damon Lipinski, James P. Whelan, Andrew W. Meyers**. Treatment of pathological gambling using a guided self-change approach.. *Clinical Case Studies*. 2007/10//. 6:394	Level 1, Form Title/abstract screen
2604	**Vikram Patel, Ricardo Araya, Sudipto Chatterjee, Dan Chisholm, Alex Cohen, Mary De Silva, C. Hosman, Hugh McGuire, Graciela Rojas, Mark van Ommeren**. Treatment and prevention of mental disorders in low-income and middle-income countries.. *The Lancet*. 2007/09//. 370:991	Level 1, Form Title/abstract screen
2606	**Mona M. Shattell, Sharon S. Starr, Sandra P. Thomas**. 'Take my hand, help me out': Mental health service recipients' experience of the therapeutic relationship.. *International Journal of Mental Health Nursing*. 2007/08//. 16:274	Level 1, Form Title/abstract screen
2609	**Carole Schauer, Anita Everett, Paolo del Vecchio, Leigh Anderson**. Promoting the value and practice of shared decision-making in mental health care.. *Psychiatric Rehabilitation Journal*. 2007///Sum 2007. 31:54	Level 1, Form Title/abstract screen
2610	**Duncan G. Campbell, Bradford L. Felker, Chuan-Fen Liu, Elizabeth M. Yano, JoAnn E. Kirchner, Domin Chan, Lisa V. Rubenstein, Edmund F. Chaney**. Prevalence of depression–PTSD comorbidity: Implications for clinical practice guidelines and primary care-based interventions.. *Journal of General Internal Medicine*. 2007/06//. 22:711	Level 1, Form Title/abstract screen
2613	**Brenda D. Smith, Cristina Mogro-Wilson**. Multi-level influences on the practice of inter-agency collaboration in child welfare and substance abuse treatment.. *Children and Youth Services Review*. 2007/05//. 29:545	Level 1, Form Title/abstract screen
2614	**A. Kathryn Power, Paolo del Vecchio**. Consumer-directed behavioral health care.. *Psychiatric Services*. 2007/05//. 58:714	Level 1, Form Title/abstract screen
2618	**Eunice C. Wong, Larry E. Beutler, Nolan W. Zane**. Using mediators and moderators to test assumptions underlying culturally sensitive therapies: An exploratory example.. *Cultural Diversity and Ethnic Minority Psychology*. 2007/04//. 13:169	Level 1, Form Title/abstract screen
2621	**Michael Münchow**. Nej/ja, tak! Frustration og gratifikation i misbrugsbehandling. = No/yes please! Frustration and gratification in treatment of addictions.. *Matrix: Nordisk Tidsskrift for Psykoterapi*. 2007/03//. 24:31	Level 1, Form Title/abstract screen
2624	**Paul Crits-Christoph, Mary Beth Connolly Gibbons, Jacques P. Barber, Bojun Hu, Bridget Hearon, Matt Worley, Robert Gallop**. Predictors of sustained abstinence during psychosocial treatments for cocaine dependence.. *Psychotherapy Research*. 2007/03//. 17:240	Level 1, Form Title/abstract screen
2626	**Robert J. Gallop, Paul Crits-Christoph, Thomas R. Ten Have, Jacques P. Barber, Arlene Frank, Margaret L. Griffin, Michael E. Thase**. Differential transitions between cocaine use and abstinence for men and women.. *Journal of Consulting and Clinical Psychology*. 2007/02//. 75:95	Level 1, Form Title/abstract screen
2628	**Susan Dowd Stone**. Using dialectical behavior therapy in clinical practice: Client empowerment, social work values.. *Cognitive behavior therapy in clinical social work practice.*. 2007///. #volume#:147	Level 1, Form Title/abstract screen
2629	**Christina Rosen Galvin, John Sommers-Flanagan, Linwood G. Vereen**. 'Are you saying Corey is an addict?': Childhood substance abuse.. *Critical incidents in counseling children.*. 2007///. #volume#:337	Level 1, Form Title/abstract screen
2630	**Maria Hodermarska, Suzannah Scott-Moncrieff**. Operatic play: A drama and	Level 1, Form

	music therapy collaboration.. *Healing the inner city child: Creative arts therapies with at-risk youth*.. 2007///. #volume#:242	Title/abstract screen
2631	**Steven P. Verney, Billie Jo Kipp**. Acculturation and alcohol treatment in ethnic minority populations: Assessment issues and implications.. *Alcoholism Treatment Quarterly*. 2007///. 25:47	Level 1, Form Title/abstract screen
2632	**Howard A. Paul**. Review of The therapist's guide to psychopharmacology: Working with patients, families, and physicians to optimize care.. *Child & Family Behavior Therapy*. 2007///. 29:83	Level 1, Form Title/abstract screen
2633	**Andrea Savage, Laura Quiros, Sarah-Jane Dodd, Diane Bonavota**. Building trauma informed practice: Appreciating the impact of trauma in the lives of women with substance abuse and mental health problems.. *Journal of Social Work Practice in the Addictions*. 2007///. 7:91	Level 2, Form Full Text Screening
2635	**Doreen D. Salina, Linda M. Lesondak, Lisa A. Razzano, Ann Weilbaecher**. Co-occurring mental disorders among incarcerated women: Preliminary findings from an integrated health treatment study.. *Journal of Offender Rehabilitation*. 2007///. 45:207	Level 1, Form Title/abstract screen
2636	**Sarah W. Feldstein, Alyssa A. Forcehimes**. Motivational interviewing with underage college drinkers: A preliminary look at the role of empathy and alliance.. *The American Journal of Drug and Alcohol Abuse*. 2007///. 33:737	Level 1, Form Title/abstract screen
2637	**Susan M. Grantham**. Major depression and alcohol comorbidity in an elderly population: Access, outcomes, and services use.. *Dissertation Abstracts International Section A: Humanities and Social Sciences*. 2007///. 68:1153	Level 1, Form Title/abstract screen
2638	**Michele M. Sutliffe**. A program evaluation of the pediatric behavioral health project: A co-location model of integrated behavioral health care.. *Dissertation Abstracts International: Section B: The Sciences and Engineering*. 2007///. 68:1946	Level 1, Form Title/abstract screen
2639	**Andrew J. Darchuk**. The role of the therapeutic alliance and its relationship to treatment outcome and client motivation in an adolescent substance abuse treatment setting.. *Dissertation Abstracts International: Section B: The Sciences and Engineering*. 2007///. 68:3392	Level 1, Form Title/abstract screen
2640	**Michael C. Wolff**. Substance abuse counseling: Exploring the pathways of therapeutic process.. *Dissertation Abstracts International: Section B: The Sciences and Engineering*. 2007///. 67:4728	Level 2, Form Full Text Screening
2641	**Stephen Brayton Woolley**. Mental illness, its treatment, and symptoms of mental and physical illness.. *Dissertation Abstracts International: Section B: The Sciences and Engineering*. 2007///. 67:5459	Level 1, Form Title/abstract screen
2642	**Martin Cortez Wesley**. Animal-assisted therapy and the therapeutic alliance in the treatment of substance dependence.. *Dissertation Abstracts International: Section B: The Sciences and Engineering*. 2007///. 67:6083	Level 2, Form Full Text Screening
2643	**Hoyle Leigh, John Mark Streltzer**. Handbook of consultation-liaison psychiatry.. *#journal#*. 2007///. #volume#:#pages#	Level 1, Form Title/abstract screen
2644	**Glenn N. Saxe, B. Heidi Ellis, Julie B. Kaplow**. Collaborative treatment of traumatized children and teens: The trauma systems therapy approach.. *#journal#*. 2007///. #volume#:#pages#	Level 1, Form Title/abstract screen
2645	**Myrna M. Weissman, John C. Markowitz, Gerald L. Klerman**. Clinician's quick guide to interpersonal psychotherapy.. *#journal#*. 2007///. #volume#:#pages#	Level 1, Form Title/abstract screen

2646	**Mayyada Wazaify, Carmel M. Hughes, James C. McElnay**. The implementation of a harm minimisation model for the identification and treatment of over-the-counter drug misuse and abuse in community pharmacies in Northern Ireland.. *Patient Education and Counseling*. 2006/12//. 64:136	Level 1, Form Title/abstract screen
2647	**Christopher D. Carroll, Ronald W. Manderscheid, Allen S. Daniels, Amelia Compagni**. Convergence of Service, Policy, and Science Toward Consumer-Driven Mental Health Care.. *Journal of Mental Health Policy and Economics*. 2006/12//. 9:185	Level 1, Form Title/abstract screen
2651	**Monica H. Swahn, John E. Donovan**. Alcohol and violence: Comparison of the psychosocial correlates of adolescent involvement in alcohol-related physical fighting versus other physical fighting.. *Addictive Behaviors*. 2006/11//. 31:2014	Level 1, Form Title/abstract screen
2652	**Aloen L. Townsend, David E. Biegel, Karen J. Ishler, Barbara Wieder, Amy Rini**. Families of Persons With Substance Use and Mental Disorders: A Literature Review and Conceptual Framework.. *Family Relations: An Interdisciplinary Journal of Applied Family Studies*. 2006/10//. 55:473	Level 1, Form Title/abstract screen
2654	**Louis J. Presenza**. Naltrexone as a 'mandate' or as a choice: Comments on 'Judicially mandated naltrexone use by criminal offenders: A legal analysis.'. *Journal of Substance Abuse Treatment*. 2006/09//. 31:129	Level 1, Form Title/abstract screen
2657	**Ron Langevin**. Acceptance and Completion of Treatment Among Sex Offenders.. *International Journal of Offender Therapy and Comparative Criminology*. 2006/08//. 50:402	Level 1, Form Title/abstract screen
2660	**Steven H. Woolf, Alex H. Krist, Robert E. Johnson, Diane B. Wilson, Stephen F. Rothemich, Gregory J. Norman, Kelly J. Devers**. 'A Practice-Sponsored Web Site to Help Patients Pursue Healthy Behaviors: An ACORN Study': Corrections.. *Annals of Family Medicine*. 2006/07//Jul-Aug, 2006. 4:371	Level 1, Form Title/abstract screen
2661	**Michael Fendrich, Amy Hubbell, Arthur J. Lurigio**. Providers' perceptions of gender-specific drug treatment.. *Journal of Drug Issues*. 2006///Sum 2006. 36:667	Level 1, Form Title/abstract screen
2664	**Nancy Suchman, Marjukka Pajulo, Cindy DeCoste, Linda Mayes**. Parenting interventions for drug-dependent mothers and their young children: the case for an attachment-based approach.. *Family Relations: An Interdisciplinary Journal of Applied Family Studies*. 2006/04//. 55:211	Level 1, Form Title/abstract screen
2665	**Amelia Compagni, Ronald W. Manderscheid**. A Neuroscientist-Consumer Alliance to Transform Mental Health Care.. *The Journal of Behavioral Health Services & Research*. 2006/04//. 33:265	Level 1, Form Title/abstract screen
2667	**Catherine J. Datto, Richard Thompson, Kathryn Knott, Ira R. Katz**. Older Adult Report of Change in Depressive Symptoms as a Treatment Decision Tool.. *Journal of the American Geriatrics Society*. 2006/04//. 54:627	Level 1, Form Title/abstract screen
2668	**Robert Elliott, Dave Mearns, Peter F. Schmid, William B. Stiles**. Potsdam and beyond.. *Person-Centered and Experiential Psychotherapies*. 2006///Spr 2006. 5:1	Level 1, Form Title/abstract screen
2674	**Ralph Aquila, John Kelleher, Thomas Sweet**. Housing.. *Clinical guide to the treatment of the mentally ill homeless person.*. 2006///. #volume#:65	Level 1, Form Title/abstract screen
2675	**A. Simpson**. Shared care and inter-professional practice.. *Dual diagnosis nursing.*. 2006///. #volume#:130	Level 1, Form Title/abstract screen
2678	**Elizabeth H. B. Lin, Wayne Katon, Carolyn Rutter, Greg E. Simon, Evette**	Level 1, Form

	**J. Ludman, Michael Von Korjff, Bessie Young, Malia Oliver, Paul C. Ciechanowski, Leslie Kinder, Edward Walker**. Effects of Enhanced Depression Treatment on Diabetes Self-Care.. *Annals of Family Medicine*. 2006/01//Jan-Feb, 2006. 4:46	Title/abstract screen
2681	**Douglas L. Polcin**. Reexamining confrontation and motivational interviewing.. *Addictive Disorders & Their Treatment*. 2006///. 5:201	Level 1, Form Title/abstract screen
2684	**Leslie Alkalay**. Profiling adolescent substance abusers by therapeutic alliance.. *Dissertation Abstracts International: Section B: The Sciences and Engineering*. 2006///. 67:2822	Level 2, Form Full Text Screening
2685	**Lesia M. Ruglass**. Ethnocultural differences in therapeutic alliance and outcome for women with comorbid posttraumatic stress disorder and substance use disorder.. *Dissertation Abstracts International: Section B: The Sciences and Engineering*. 2006///. 66:4499	Level 2, Form Full Text Screening
2686	**Thuy Tran Boardman**. Motivational interviewing: Examining the therapeutic process.. *Dissertation Abstracts International: Section B: The Sciences and Engineering*. 2006///. 66:6262	Level 2, Form Full Text Screening
2687	**Anna Chur-Hansen**. 'Adding Life to Years': A Manual of Instruction for Practitioners to Help Clients Quit Smoking.. *PsycCRITIQUES*. 2006///. 51:#pages#	Level 1, Form Title/abstract screen
2688	**Christopher Gillberg, Richard Harrington, Hans-Christoph Steinhausen**. A clinician's handbook of child and adolescent psychiatry.. *#journal#*. 2006///. #volume#:#pages#	Level 1, Form Title/abstract screen
2689	**Zoë Henderson**. Review of Drug induced. Addiction and treatment in perspective.. *Drugs: Education, Prevention & Policy*. 2005/12//. 12:511	Level 1, Form Title/abstract screen
2691	**Monica Paccaloni, Francesca Moretti, Christa Zimmermann**. Le informazioni fornite e il coinvolgimento alla cura in psichiatria: Cosa pensano gli psichiatri? Una revisione della letteratura. = Giving information and involving in treatment: What do psychiatrists think? A review.. *Epidemiologia e Psichiatria Sociale*. 2005/10//Oct-Dec, 2005. 14:198	Level 1, Form Title/abstract screen
2692	**David Leonard, Susan Brann, John Tiller**. Dissociative disorders: Pathways to diagnosis, clinician attitudes and their impact.. *Australian and New Zealand Journal of Psychiatry*. 2005/10//. 39:940	Level 1, Form Title/abstract screen
2695	**Alain D Lesage**. Can Psychiatrists Prevent Suicide? Yes, in Collaboration.. *The Canadian Journal of Psychiatry / La Revue canadienne de psychiatrie*. 2005/08//. 50:507	Level 1, Form Title/abstract screen
2698	**Mads Uffe Pedersen**. Arbejdsalliancens todimensionalitet og dens betydning for behandling af heroinafhængige: En kvantitativ undersøgelse af forskellige allianceskalaer. = The Two-Dimensionality of the Working Alliance and some Implications for Treatment of Heroin Dependent Persons. A quantitative study of different alliance scales.. *Nordisk Psykologi*. 2005/07//. 57:131	Level 2, Form Full Text Screening
2700	**Satish Kedia, Stephanie W. Perry**. Factors associated with client-collateral agreement in substance abuse post-treatment self-reports.. *Addictive Behaviors*. 2005/07//. 30:1086	Level 1, Form Title/abstract screen
2702	**Saxby Pridmore**. Review of Pain and depression. An interdisciplinary client-centered approach. Advances in psychosomatic medicine, Vol. 25.. *Australian and New Zealand Journal of Psychiatry*. 2005/05//. 39:427	Level 1, Form Title/abstract screen
2703	**Daniela Wittmann**. Review of Social Skills Training for Schizophrenia	Level 1, Form

	(Second Edition) A Step-by-Step Guide.. *Annals of Clinical Psychiatry*. 2005/04//Apr-Jun, 2005. 17:102	Title/abstract screen
2704	**Connie R. Matthews, Peggy Lorah**. An Examination of Addiction Treatment Completion by Gender and Ethnicity.. *Journal of Addictions & Offender Counseling*. 2005/04//. 25:114	Level 1, Form Title/abstract screen
2705	**Maurice Dongier**. Review of Integrated Treatment for Dual Disorders. A Guide to Effective Practice.. *The Canadian Journal of Psychiatry / La Revue canadienne de psychiatrie*. 2005/04//. 50:299	Level 1, Form Title/abstract screen
2706	**Ronda L. Dearing, Christopher Barrick, Kurt H. Dermen, Kimberly S. Walitzer**. Indicators of Client Engagement: Influences on Alcohol Treatment Satisfaction and Outcomes.. *Psychology of Addictive Behaviors*. 2005/03//. 19:71	Level 1, Form Title/abstract screen
2708	**Kathleen M. Carroll**. Editorial: Only connect?. *Addiction*. 2005/03//. 100:267	Level 1, Form Title/abstract screen
2710	**Roger D. Weiss, Margaret L. Griffin, Robert J. Gallop, Lisa M. Najavits, Arlene Frank, Paul Crits-Christoph, Michael E. Thase, Jack Blaine, David R. Gastfriend, Dennis Daley, Lester Luborsky**. The effect of 12-step self-help group attendance and participation on drug use outcomes among cocaine-dependent patients.. *Drug and Alcohol Dependence*. 2005/02//. 77:177	Level 1, Form Title/abstract screen
2711	**Patrick Boyle, Christina M. Delos Reyes, Richard A. Kruszynski**. Integrated Dual-Disorder Treatment.. *Evidence-based mental health practice: A textbook..* 2005///. #volume#:349	Level 2, Form Full Text Screening
2714	**Richard Dembo, Wansley Walters, Kathleen Meyers**. A Practice/Research Collaborative: An Innovative Approach to Identifying and Responding to Psychosocial Functioning Problems and Recidivism Risk Among Juvenile Arrestees.. *Journal of Offender Rehabilitation*. 2005///. 41:39	Level 1, Form Title/abstract screen
2715	**Irene Panagopoulos, Lina A. Ricciardelli**. Harm reduction and decision making among recreational ecstasy users.. *International Journal of Drug Policy*. 2005/01//. 16:54	Level 1, Form Title/abstract screen
2716	**Sharlene A. Wolchik, Irwin N. Sandler, Emily Winslow, Vicki Smith-Daniels**. Programs for Promoting Parenting of Residential Parents: Moving From Efficacy to Effectiveness.. *Family Court Review*. 2005/01//. 43:65	Level 1, Form Title/abstract screen
2718	**Carla Maine**. Feminist-narrative therapy: Treating PTSD and substance abuse in women.. *Dissertation Abstracts International: Section B: The Sciences and Engineering*. 2005///. 66:564	Level 1, Form Title/abstract screen
2719	**Sharon M. Flicker**. The relationship between ethnic matching, therapeutic alliance, and treatment outcome with Hispanic and Anglo adolescents in family therapy.. *Dissertation Abstracts International: Section B: The Sciences and Engineering*. 2005///. 65:4282	Level 2, Form Full Text Screening
2720	**Lana R. Gaiton**. Investigation of therapeutic alliance in a treatment study with substance-abusing women with PTSD.. *Dissertation Abstracts International: Section B: The Sciences and Engineering*. 2005///. 65:4828	Level 2, Form Full Text Screening
2721	**Julie M. Liszka-Chaloner**. An exploration of the relationship of attachment style and working alliance on substance abuse client treatment satisfaction.. *Dissertation Abstracts International: Section B: The Sciences and Engineering*. 2005///. 65:6660	Level 2, Form Full Text Screening
2722	**Andrés G. Gil, Eric F. Wagner, Jonathan G. Tubman**. Culturally sensitive substance abuse intervention for Hispanic and African American adolescents:	Level 1, Form Title/abstract

	Empirical examples from the Alcohol Treatment Targeting Adolescents in Need (ATTAIN) Project.. *Addiction*. 2004/11//. 99:140	screen
2724	**Sylvia Dennison**. Review of Seeking Safety: A Treatment Manual for PTSD and Substance Misuse.. *The American Journal on Addictions*. 2004/10//Oct-Dec, 2004. 13:501	Level 1, Form Title/abstract screen
2725	**Lynne Siqueland, Paul Crits-Christoph, Jacques P. Barber, Mary Beth Connolly Gibbons, Robert Gallop, Margaret Griffin, Arlene Frank, Michael E. Thase, Lester Luborsky, Bruce Liese**. What aspects of treatment matter to the patient in the treatment of cocaine dependence?. *Journal of Substance Abuse Treatment*. 2004/09//. 27:169	Level 1, Form Title/abstract screen
2726	**Dan Chisholm, Kristy Sanderson, Jose Luis Ayuso-Mateos, Shekhar Saxena**. Reducing the global burden of depression: Population-level analysis of intervention cost-effectiveness in 14 world regions.. *The British Journal of Psychiatry*. 2004/05//. 184:393	Level 1, Form Title/abstract screen
2730	**Peter Schlebusch**. Review of Problem drinking: A person-centred dialogue.. *Person-Centered and Experiential Psychotherapies*. 2004///Spr 2004. 3:70	Level 1, Form Title/abstract screen
2731	**Jon Glasby, Helen Lester**. Cases for change in mental health: partnership working in mental health services.. *Journal of Interprofessional Care*. 2004/02//. 18:7	Level 1, Form Title/abstract screen
2732	**Joanne Neale**. Drug driving in Scotland: Prevalence and correlates amongst drug users entering treatment.. *International Journal of Drug Policy*. 2004/02//. 15:27	Level 1, Form Title/abstract screen
2733	**Richard Dobbins**. Spiritual interventions in the treatment of dysthymia and alcoholism.. *Casebook for a spiritual strategy in counseling and psychotherapy*.. 2004///. #volume#:105	Level 2, Form Full Text Screening
2734	**Meredith Hanson, Nabila El-Bassel**. Motivating substance-abusing clients through the helping process.. *Clinical work with substance-abusing clients*.. 2004///. #volume#:39	Level 1, Form Title/abstract screen
2735	**Marion S. Forgatch, Bernadette Marie Bullock, Gerald R. Patterson**. From Theory to Practice: Increasing Effective Parenting Through Role-Play.. *Handbook of mental health interventions in children and adolescents: An integrated developmental approach*.. 2004///. #volume#:782	Level 1, Form Title/abstract screen
2736	**Gerard J. Connors, Scott H. Stewart**. Alcohol and Other Substance Use Disorders.. *Handbook of primary care psychology*.. 2004///. #volume#:187	Level 1, Form Title/abstract screen
2737	**C. Robert Cloninger**. Sickly.. *Treatment companion to the DSM-IV-TR casebook*.. 2004///. #volume#:306	Level 1, Form Title/abstract screen
2738	**Jacques P. Barber, Carol Foltz, Paul Crits-Christoph, Jesse Chittams**. Therapists' Adherence and Competence and Treatment Discrimination in the NIDA Collaborative Cocaine Treatment Study.. *Journal of Clinical Psychology*. 2004/01//. 60:29	Level 1, Form Title/abstract screen
2740	**Stanton Peele**. How I Found Common Cause with Social Workers.. *Journal of Social Work Practice in the Addictions*. 2004///. 4:117	Level 1, Form Title/abstract screen
2743	**Roberto Secades-Villa, José Ramón Fernánde-Hermida, Cristina Arnáez-Montaraz**. Motivational interviewing and treatment retention among drug user patients: A pilot study.. *Substance Use & Misuse*. 2004///. 39:1369	Level 1, Form Title/abstract screen

2744	**Nathilee A. Caldeira**. Dissociation and treatment outcome in urban women with comorbid PTSD and substance use disorders.. *Dissertation Abstracts International: Section B: The Sciences and Engineering*. 2004///. 65:1540	Level 1, Form Title/abstract screen
2745	**Jolae Brocato**. Predictors of client retention in alternative-to-prison substance abuse programs.. *Dissertation Abstracts International Section A: Humanities and Social Sciences*. 2004///. 65:1963	Level 1, Form Title/abstract screen
2747	**Eva Kit-San Pang**. Substance abuse in a Chinese-American family: A qualitative analysis of family attitudes and experiences.. *Dissertation Abstracts International: Section B: The Sciences and Engineering*. 2004///. 65:2644	Level 1, Form Title/abstract screen
2748	**Cheryl L. Pugh**. An interface of the twelve-step theory and Afrocentric theory for the treatment of substance abuse in African Americans.. *Dissertation Abstracts International: Section B: The Sciences and Engineering*. 2004///. 64:5798	Level 1, Form Title/abstract screen
2749	**W. Robert Nay**. Taking charge of anger: How to resolve conflict, sustain relationships, and express yourself without losing control.. *#journal#*. 2004///. #volume#:#pages#	Level 1, Form Title/abstract screen
2750	**Eileen Britt, Neville M. Blampied, Stephen M. Hudson**. Motivational interviewing: A review.. *Australian Psychologist*. 2003/11//. 38:193	Level 1, Form Title/abstract screen
2751	**William D. Marelich, D. A. Murphy**. Effects of empowerment among HIV-positive women on the patient-provider relationship.. *AIDS Care*. 2003/08//. 15:475	Level 1, Form Title/abstract screen
2752	**Roger D. Weiss, Margaret L. Griffin, Carissa Mazurick, Benjamin Berkman, David R. Gastfriend, Arlene Frank, Jacques P. Barber, Jack Blaine, Ihsan Salloum, Karla Moras**. The Relationship Between Cocaine Craving, Psychosocial Treatment, and Subsequent Cocaine Use.. *The American Journal of Psychiatry*. 2003/07//. 160:1320	Level 1, Form Title/abstract screen
2756	**Cheryl A. Hosley, Linda Gensheimer, Mai Yang**. Building Effective Working Relationships Across Culturally and Ethnically Diverse Communities.. *Child Welfare: Journal of Policy, Practice, and Program*. 2003/03//Mar-Apr, 2003. 82:157	Level 1, Form Title/abstract screen
2757	**Jonathan Andrew Pastor**. Latino clinicians' perceptions of Latino men in psychotherapy: An exploratory study.. *Dissertation Abstracts International: Section B: The Sciences and Engineering*. 2003/03//. 63:3933	Level 1, Form Title/abstract screen
2758	**Alice A. Gleghorn, Frances Cotter**. National and local perspectives on the Center for Substance Abuse Treatment Practice/Research Collaborative and Practice Improvement Collaborative Initiatives.. *Drug abuse treatment through collaboration: Practice and research partnerships that work.*. 2003///. #volume#:213	Level 1, Form Title/abstract screen
2759	**Sheila Krystal**. A Nondual Approach To EMDR: Psychotherapy as Satsang.. *The sacred mirror: Nondual wisdom and psychotherapy.*. 2003///. #volume#:116	Level 1, Form Title/abstract screen
2760	**Carlo C. DiClemente, Kathleen M. Carroll, William R. Miller, Gerard J. Connors, Dennis M. Donovan**. A look inside treatment: Therapist effects, the therapeutic alliance, and the process of intentional behavior change.. *Treatment matching in alcoholism.*. 2003///. #volume#:166	Level 1, Form Title/abstract screen
2762	. Review of Addiction Treatment: A Strengths Perspective.. *Family Therapy*. 2003///. 30:62	Level 1, Form Title/abstract screen

2763	**Lynne Siqueland, Paul Crits-Christoph, Bob Gallop, David Gastfriend, Judy Lis, Arlene Frank, Margaret Griffin, Jack Blaine, Lester Luborsky**. Who starts treatment: Engagement in the NIDA collaborative cocaine treatment study.. *The American Journal on Addictions*. 2002///Win 2002. 11:10	Level 1, Form Title/abstract screen
2764	**Lynne Siqueland, Paul Crits-Christoph, Robert Gallop, Jacques P. Barber, Margaret L. Griffin, Michael E. Thase, Denis Daley, Arlene Frank, David R. Gastfriend, Jack Blaine, Mary Beth Connolly, Madeline Gladis**. Retention in psychosocial treatment of cocaine dependence: Predictors and impact on outcome.. *The American Journal on Addictions*. 2002///Win 2002. 11:24	Level 1, Form Title/abstract screen
2767	**R. Conviser, M. B. Pounds**. The role of ancillary services in client-centered systems of care.. *AIDS Care*. 2002/08//. 14:S119	Level 1, Form Title/abstract screen
2769	**L. A. Aday**. A health services research perspective on the use of ancillary services to retain persons living with HIV/AIDS in primary care.. *AIDS Care*. 2002/08//. 14:S133	Level 1, Form Title/abstract screen
2770	**Robert I. Simon, Thomas G. Gutheil**. A recurrent pattern of suicide risk factors observed in litigated cases: Lessons in risk management.. *Psychiatric Annals*. 2002/07//. 32:384	Level 1, Form Title/abstract screen
2771	**Noreen Lillie**. Women, alcohol, self-concept and self-esteem: A qualitative study of the experience of person-centred counselling.. *Counselling & Psychotherapy Research*. 2002/06//. 2:99	Level 2, Form Full Text Screening
2772	**J. Arthur Jr. Gillaspy, Anna R. Wright, Catherine Campbell, Sandra Stokes, Byron Adinoff**. Group alliance and cohesion as predictors of drug and alcohol abuse treatment outcomes.. *Psychotherapy Research*. 2002///Sum 2002. 12:213	Level 1, Form Title/abstract screen
2773	**Suzanne Spear, Richard A. Rawson**. Perspectives from the conference, 'Common Ground, Common Goals, Common Language: Bringing Substance Abuse Practice and Research Together.'. *Journal of Drug Issues*. 2002///Sum 2002. 32:751	Level 1, Form Title/abstract screen
2774	**H. Westley Clark**. Bridging the gap between substance abuse practice and research: The National Treatment Plan Initiative.. *Journal of Drug Issues*. 2002///Sum 2002. 32:757	Level 1, Form Title/abstract screen
2775	**Kathryn I. Pollack, Kimberly S. H. Yarnall, Barbara K. Rimer, Isaac Lipkus, Pauline R. Lyna**. Factors associated with patient-recalled smoking cessation advice in a low-income clinic.. *Journal of the National Medical Association*. 2002/05//. 94:354	Level 1, Form Title/abstract screen
2777	**Margaret K. Keiley**. Case 13. Visiting all the monsters.. *Clinical epiphanies in marital and family therapy: A practitioner's casebook of therapeutic insights, perceptions, and breakthroughs*.. 2002///. #volume#:317	Level 1, Form Title/abstract screen
2778	**Eric E. McCollum**. Trading monsters for dreams.. *Clinical epiphanies in marital and family therapy: A practitioner's casebook of therapeutic insights, perceptions, and breakthroughs*.. 2002///. #volume#:330	Level 1, Form Title/abstract screen
2779	**Leslie L. Feinauer**. Embracing monsters.. *Clinical epiphanies in marital and family therapy: A practitioner's casebook of therapeutic insights, perceptions, and breakthroughs*.. 2002///. #volume#:336	Level 1, Form Title/abstract screen
2780	**Deborah Jones-Saumty**. Substance abuse treatment for Native Americans.. *Ethnicity and substance abuse: Prevention and intervention*.. 2002///. #volume#:270	Level 2, Form Full Text Screening

2782	**John Macleod**. Working with people who use illicit drugs.. *Promoting collaboration in primary mental health care*.. 2002///. #volume#:158	Level 1, Form Title/abstract screen
2783	**Kurt Renders**. Individuele motivationele cliëntgerichte psychotherapie met drugsgebruikers. = Individual motivational client-centered psychotherapy of substance abusers.. *Tijdschrift voor Psychotherapie*. 2002/01//. 28:17	Level 2, Form Full Text Screening
2786	**Drew D. Wallace**. Therapeutic alliance and the mediation of patient factors in treatment outcome: Latent variable path model.. *Dissertation Abstracts International: Section B: The Sciences and Engineering*. 2002/01//. 62:3393	Level 1, Form Title/abstract screen
2787	**Katherine Tyson, Emily Carroll**. Innovative therapeutic care for homeless, mentally ill clients: Intrapsychic humanism in a residential setting.. *Families in Society*. 2001/11//Nov-Dec, 2001. 82:591	Level 1, Form Title/abstract screen
2788	**Angela Maureen Kirk**. The perceptions and experiences of HIV positive individuals concerning the obstacles to their medical care.. *Dissertation Abstracts International: Section B: The Sciences and Engineering*. 2001/11//. 62:2488	Level 1, Form Title/abstract screen
2789	**Woody Caan**. Coming together on alcohol and drugs: A capital idea.. *Journal of Mental Health*. 2001/10//. 10:477	Level 1, Form Title/abstract screen
2790	**Paul Crits-Christoph, Lynne Siqueland, Elizabeth McCalmont, Roger D. Weiss, David R. Gastfriend, Arlene Frank, Karla Moras, Jacques P. Barber, Jack Blaine, Michael E. Thase**. Impact of psychosocial treatments on associated problems of cocaine-dependent patients.. *Journal of Consulting and Clinical Psychology*. 2001/10//. 69:825	Level 1, Form Title/abstract screen
2791	**Lisa R. Fenton, John J. Cecero, Charla Nich, Tami L. Frankforter, Kathleen M. Carroll**. Perspective is everything: The predictive validity working alliance instruments.. *Journal of Psychotherapy Practice & Research*. 2001///Fal 2001. 10:262	Level 1, Form Title/abstract screen
2792	**Timothy T. Freitas**. The relationship between neurocognitive impairment, working alliance, and length of stay in a therapeutic community.. *Dissertation Abstracts International: Section B: The Sciences and Engineering*. 2001/09//. 62:1573	Level 1, Form Title/abstract screen
2794	**Ralf Demmel**. Motivational interviewing: Ein Literaturüberblick. = Motivational Interviewing: A Review.. *Sucht: Zeitschrift für Wissenschaft und Praxis*. 2001/06//. 47:171	Level 2, Form Full Text Screening
2796	**George De Leon**. A commentary on 'Retention in substance dependence treatment: The relevance of in-treatment factors.'. *Journal of Substance Abuse Treatment*. 2001/06//. 20:263	Level 1, Form Title/abstract screen
2797	**Paul John Lamberty**. The process of long-term recovery from substance abuse among African American men: Implications for counselor training and practice.. *Dissertation Abstracts International Section A: Humanities and Social Sciences*. 2001/06//. 61:4681	Level 2, Form Full Text Screening
2798	**Sarah Michaela Logan**. Predicting working alliance of substance-abusing clients and counselors.. *Dissertation Abstracts International: Section B: The Sciences and Engineering*. 2001/06//. 61:6743	Level 1, Form Title/abstract screen
2799	**Suzanne Johnson**. The therapeutic alliance with early adolescents: Introduction of an instrument.. *Dissertation Abstracts International: Section B: The Sciences and Engineering*. 2001/04//. 61:5567	Level 1, Form Title/abstract screen
2803	**Bernadine Kay Flynn**. The consumer/case manager working alliance and its	Level 2, Form

	relationship to dual-disordered client outcomes in a representative payee treatment program.. *Dissertation Abstracts International: Section B: The Sciences and Engineering*. 2001/02//. 61:4078	Full Text Screening
2804	**Pia Marinangeli**. Italian culture and its impact on addiction.. *Ethnocultural factors in substance abuse treatment*.. 2001///. #volume#:216	Level 1, Form Title/abstract screen
2805	**Jim Gilbert, Jan Langrod**. Polish identity and substance abuse.. *Ethnocultural factors in substance abuse treatment*.. 2001///. #volume#:234	Level 1, Form Title/abstract screen
2806	**Mary Ann Bromley, S. K. Chhem Sip**. Substance abuse treatment issues with Cambodian Americans.. *Ethnocultural factors in substance abuse treatment*.. 2001///. #volume#:321	Level 1, Form Title/abstract screen
2807	**Daya Singh Sandhu, Ruby Malik**. Ethnocultural background and substance abuse treatment of Asian Indian Americans.. *Ethnocultural factors in substance abuse treatment*.. 2001///. #volume#:368	Level 1, Form Title/abstract screen
2809	**Frederick K. Goodwin, S. Nassir Ghaemi**. The difficult-to-treat patient with bipolar disorder.. *The difficult-to-treat psychiatric patient*.. 2001///. #volume#:7	Level 1, Form Title/abstract screen
2810	**Garry Prouty**. Pre-therapy: A treatment method for people with mental retardation who are also psychotic.. *Treating mental illness and behavior disorders in children and adults with mental retardation*.. 2001///. #volume#:155	Level 1, Form Title/abstract screen
2812	**Glenn D. Walters**. The Shaman effect in counseling clients with alcohol problems.. *Alcoholism Treatment Quarterly*. 2001///. 19:31	Level 1, Form Title/abstract screen
2813	**Judith Mishne**. Transformation of narcissism and the intersubjective therapeutic exchange: A depressed adolescent patient shares his music and lyrics with his therapist.. *Psychoanalytic Social Work*. 2001///. 8:71	Level 1, Form Title/abstract screen
2814	**Zora Raboteg-Šarić, Majda Rijavec, Andrea Brajša-Žganec**. The relation of parental practices and self-conceptions to young adolescent problem behaviors and substance use.. *Nordic Journal of Psychiatry*. 2001///. 55:203	Level 1, Form Title/abstract screen
2815	**Patricia E. Penn, Audrey J. Brooks**. Five years, twelve steps, and REBT in the treatment of dual diagnosis.. *Journal of Rational-Emotive & Cognitive-Behavior Therapy*. 2000///Win 2000. 18:197	Level 1, Form Title/abstract screen
2817	**Roger D. Weiss, Margaret L. Griffin, Robert Gallop, Lester Luborsky, Lynne Siqueland, Arlene Frank, Lisa S. Onken, Dennis C. Daley, David R. Gastfriend**. Predictors of self-help group attendance in cocaine dependent patients.. *Journal of Studies on Alcohol*. 2000/09//. 61:714	Level 1, Form Title/abstract screen
2818	**Jennie Echols-Hurst**. Examination of a structural model of empowerment and patient satisfaction.. *Dissertation Abstracts International: Section B: The Sciences and Engineering*. 2000/09//. 61:1322	Level 1, Form Title/abstract screen
2819	**Craig Robert Lareau**. The effect of therapeutic alliance on perceived levels of formal and informal coercion.. *Dissertation Abstracts International: Section B: The Sciences and Engineering*. 2000/09//. 61:1640	Level 1, Form Title/abstract screen
2822	**Elayne L. Chou**. Predictors of treatment acceptability, willingness to see a counselor, and counselor preferences for asian-americans and whites: Acculturation, loss of face, self-construals, and collective self-esteem.. *Dissertation Abstracts International: Section B: The Sciences and Engineering*. 2000/02//. 60:4209	Level 1, Form Title/abstract screen

2823	**Ian H. Gotlib, Pamela K. Schraedley**. Interpersonal psychotherapy.. *Handbook of psychological change: Psychotherapy processes & practices for the 21st century*.. 2000///. #volume#:258	Level 1, Form Title/abstract screen
2827	**Christie Crews Taylor**. An analysis of urban smokable cocaine clients' perception of intensive out-patient treatment dynamics and treatment outcome.. *Dissertation Abstracts International Section A: Humanities and Social Sciences*. 1999/08//. 60:0351	Level 1, Form Title/abstract screen
2828	**Richardt Sapir-Weise, Mats Berglund, Arne Frank, Hans Kristenson**. Acupuncture in alcoholism treatment: A randomized out-patient study.. *Alcohol and Alcoholism*. 1999/07//Jul-Aug, 1999. 34:629	Level 1, Form Title/abstract screen
2829	**Paul Crits-Christoph, Lynne Siqueland, Jack Blaine, Arlene Frank, Lester Luborsky, Lisa S. Onken, Larry R. Muenz, Michael E. Thase, Roger D. Weiss, David R. Gastfriend, George E. Woody, Jacques P. Barber, Stephen F. Butler, Dennis Daley, Ihsan Salloum, Sarah Bishop, Lisa M. Najavits, Judy Lis, Delinda Mercer, Margaret L. Griffin, Karla Moras, Aaron T. Beck**. Psychosocial treatments for cocaine dependence: National Institute on Drug Abuse Collaborative Cocaine Treatment Study.. *Archives of General Psychiatry*. 1999/06//. 56:493	Level 1, Form Title/abstract screen
2832	**Marta Meana, Sheri D. Pruitt, Timothy R. Dresselhaus**. Opioid therapy for chronic pancreatitis: Controlling aberrant use through behavioral management.. *General Hospital Psychiatry*. 1999/03//Mar-Apr, 1999. 21:137	Level 1, Form Title/abstract screen
2836	**Joan S. Doyle, David Stoop**. Witness and victim of multiple abuses: Case of Randy, age 10, in a residential treatment center, and follow-up at age 19 in prison.. *Play therapy with children in crisis: Individual, group, and family treatment*.. 1999///. #volume#:131	Level 1, Form Title/abstract screen
2837	**Lawrence D. Rickards, Walter Leginski, Frances L. Randolph, Deidre Oakley, James M. Herrell, Cheryl Gallagher**. Cooperative agreements for CMHS/CSAT collaborative program to prevent homelessness: An overview.. *Alcoholism Treatment Quarterly*. 1999///. 17:1	Level 1, Form Title/abstract screen
2839	**Lauren Lawendowski Aubrey**. Motivational interviewing with adolescents presenting for outpatient substance abuse treatment.. *Dissertation Abstracts International: Section B: The Sciences and Engineering*. 1998/09//. 59:1357	Level 2, Form Full Text Screening
2840	**Gary Marc Diamond**. Therapist alliance building techniques with adolescents in family therapy.. *Dissertation Abstracts International: Section B: The Sciences and Engineering*. 1998/04//. 58:5640	Level 2, Form Full Text Screening
2841	**Lisa M. Najavits, David R. Gastfriend, Jacques P. Barber, Sharon Reif, Larry R. Muenz, Jack Blaine, Arlene Frank, Paul Crits-Christoph, Michael Thase, Roger D. Weiss**. Cocaine dependence with and without PTSD among subjects in the National Institute on Drug Abuse Collaborative Cocaine Treatment Study.. *The American Journal of Psychiatry*. 1998/02//. 155:214	Level 1, Form Title/abstract screen
2842	**Myrna M. Weissman, John C. Markowitz**. An overview of interpersonal psychotherapy.. *Interpersonal psychotherapy*.. 1998///. #volume#:1	Level 1, Form Title/abstract screen
2843	**Laura Mufson, Donna Moreau**. Interpersonal psychotherapy for adolescent depression.. *Interpersonal psychotherapy*.. 1998///. #volume#:35	Level 1, Form Title/abstract screen
2844	**Robert D. Margolis, Joan E. Zweben**. Determining Appropriate Treatment: The Collaborative Challenge.. *Treating patients with alcohol and other drug problems: An integrated approach*.. 1998///. #volume#:145	Level 2, Form Full Text Screening

2845	**Rudolf H. Moos, Bernice S. Moos**. The staff workplace and the quality and outcome of substance abuse treatment.. *Journal of Studies on Alcohol*. 1998/01//. 59:43	Level 1, Form Title/abstract screen
2846	**Jacques P. Barber, Ilana Krakauer, Naomi Calvo, Peter C. Badgio**. Measuring adherence and competence of dynamic therapists in the treatment of cocaine dependence.. *Journal of Psychotherapy Practice & Research*. 1997///Win 1997. 6:12	Level 1, Form Title/abstract screen
2848	**M. Soyka, C. Kirchmayer, G. Kotter, C. John, E. Lőhnert, H. -J. Mőller**. Neue Mőglichkeiten der Therapie und Rehabilitation alkohol-abhängiger Patienten. Katamnestische Untersuchung zur Effizienz ambulanter Entwőhnungstherapien am Beispiel einer Modelleinrichtung. = New possibilities of treatment and rehabilitation of alcohol-dependent patients. Catamnestic study on the efficacy of outpatient treatment programmes demonstrated by a model procedure.. *Fortschritte der Neurologie, Psychiatrie*. 1997/09//. 65:407	Level 1, Form Title/abstract screen
2849	**Paul Crits-Christoph, Lynne Siqueland, Jack Blaine, Arlene Frank, Lester Luborsky, Lisa Simon Onken, Larry Muenz, Michael E. Thase, Roger D. Weiss, David R. Gastfriend, George Woody, Jacques P. Barber, Stephen F. Butler, Dennis Daley, Sarah Bishop, Lisa M. Najavits, Judy Lis, Delinda Mercer, Margaret L. Griffin, Karla Moras, Aaron T. Beck**. The National Institute on Drug Abuse Collaborative Cocaine Treatment Study: Rationale and methods.. *Archives of General Psychiatry*. 1997/08//. 54:721	Level 1, Form Title/abstract screen
2850	**John Lilja, Sam Larsson, David Hamilton**. Toward a theory of social pharmacology: The actor–spectator paradox applied to the psychotropic prescribing process.. *Substance Use & Misuse*. 1997/07//. 32:1175	Level 1, Form Title/abstract screen
2851	**Roger D. Weiss, Margaret L. Griffin, Cathryn Hufford, Larry R. Muenz, Lisa M. Najavits, Stephanie B. Jansson, Julia Kogan, Heather J. Thompson**. Early prediction of initiation of abstinence from cocaine: Use of a craving questionnaire.. *The American Journal on Addictions*. 1997///Sum 1997. 6:224	Level 1, Form Title/abstract screen
2853	**A. E. Eyler, Laurie L. Dicken, J. T. Fitzgerald, Mary S. Oh, Fredric M. Wolf, Andrew J. Zweifler**. Teaching smoking-cessation counseling to medical students using simulated patients.. *American Journal of Preventive Medicine*. 1997/05//May-Jun, 1997. 13:153	Level 1, Form Title/abstract screen
2854	**Agneta Öjehagen, Mats Berglund, Lars Hansson**. The relationship between helping alliance and outcome in outpatient treatment of alcoholics: A comparative study of psychiatric treatment and multimodal behavioural therapy.. *Alcohol and Alcoholism*. 1997/05//May-Jun, 1997. 32:241	Level 1, Form Title/abstract screen
2855	**Jerry A. Morris, James G. Hill**. Rural hospital addictions screening and treatment.. *Practicing psychology in rural settings: Hospital privileges and collaborative care*.. 1997///. #volume#:127	Level 1, Form Title/abstract screen
2856	**Juan José Sănchez Sosa, Laura Hernández Guzmán, Lorena Romero M.**. Predictores psicosociales del fracaso conyugal: Un estudio exploratorio. = Psychosocial predictors of marital distress: An exploratory study.. *Archivos Hispanoamericanos de Sexología*. 1997///. 3:125	Level 1, Form Title/abstract screen
2857	**Ray Dean**. Tyrannosaurus therapy: How to avoid emotional extinction.. *#journal#*. 1997///. #volume#:#pages#	Level 1, Form Title/abstract screen
2858	**Roger D. Weiss, Margaret L. Griffin, Lisa M. Najavits, Cathryn Hufford, Julia Kogan, Heather J. Thompson, Joseph H. Albeck, Sarah Bishop,**	Level 1, Form Title/abstract

	**Dennis C. Daley, Delinda Mercer, Lynne Siqueland**. Self-help activities in cocaine dependent patients entering treatment: Results from the NIDA collaborative cocaine treatment study.. *Drug and Alcohol Dependence*. 1996/12//. 43:79	screen
2862	**Kate B. Carey**. Substance use reduction in the context of outpatient psychiatric treatment: A collaborative, motivational, harm reduction approach.. *Community Mental Health Journal*. 1996/06//. 32:291	Level 1, Form Title/abstract screen
2864	**Martha Morrison Dore, Leslie B. Alexander**. Preserving families at risk of child abuse and neglect: The role of the helping alliance.. *Child Abuse & Neglect*. 1996/04//. 20:349	Level 1, Form Title/abstract screen
2866	**Gerald L. Vigdal, Donald W. Stadler**. Assessment, client treatment matching, and managing the substance abusing offender.. *Drug treatment behind bars: Prison-based strategies for change.*. 1996///. #volume#:17	Level 1, Form Title/abstract screen
2868	**Frances A. Stillman**. Tobacco control and smoking cessation efforts in an inner-city African American community.. *Journal of Social Distress & the Homeless*. 1996/01//. 5:55	Level 1, Form Title/abstract screen
2870	**Terri Gullickson**. Review of Addictions Treatment for Older Adults: Evaluation of an Innovative Client-Centered Approach.. *Contemporary Psychology*. 1995/11//. 40:1115	Level 1, Form Title/abstract screen
2871	**Helen Stephanie Raytek**. If you build it, they will come: Therapeutic alliance and the retention of couples in conjoint alcoholism treatment.. *Dissertation Abstracts International: Section B: The Sciences and Engineering*. 1995/11//. 56:2881	Level 2, Form Full Text Screening
2872	**James H. Bray, John C. Rogers**. Linking psychologists and family physicians for collaborative practice.. *Professional Psychology: Research and Practice*. 1995/04//. 26:132	Level 1, Form Title/abstract screen
2873	**Jacques Morgan Ama Okonji**. Counseling style preference and perception of counselors by African American male students.. *Dissertation Abstracts International: Section B: The Sciences and Engineering*. 1995/03//. 55:3811	Level 1, Form Title/abstract screen
2874	**Robert B. Jr. Rutherford, Mary Magee Quinn**. Establishing Effective Collaboration in the Schools.. *Contemporary Psychology*. 1995/02//. 40:132	Level 1, Form Title/abstract screen
2875	**Jay Fagan, Howard Stevenson**. Men as teachers: A self-help program on parenting for African American men.. *Social Work with Groups: A Journal of Community and Clinical Practice*. 1995///. 17:29	Level 1, Form Title/abstract screen
2876	**Kathryn Marie Graham, Sarah J. Saunders, Margaret C. Flower, Carol Birchmore Timney, Marilyn White-Campbell, Anne Zeidman Pietropaolo**. Addictions treatment for older adults: Evaluation of an innovative client-centered approach.. *#journal#*. 1995///. #volume#:#pages#	Level 2, Form Full Text Screening
2877	**Richard Longabaugh, Philip W. Wirtz, Carlo C. DiClemente, Mark Litt**. Issues in the development of client-treatment matching hypotheses.. *Journal of Studies on Alcohol*. 1994/12//. Suppl 12:46	Level 1, Form Title/abstract screen
2878	**Neil B. Colan, Katherine C. Mague, Ronna S. Cohen, Robert J. Schneider**. Family education in the workplace: A prevention program for working parents and school-age children.. *The Journal of Primary Prevention*. 1994///Win 1994. 15:161	Level 1, Form Title/abstract screen
2879	**Eleanor J. Sullivan, Sandra M. Handley, Helen Connors**. The role of nurses in primary care: Managing alcohol-abusing patients.. *Alcohol Health & Research World*. 1994///. 18:158	Level 2, Form Full Text Screening

2880	**James L. Sorensen, Joan E. Zweben**. Psychosocial treatment of the addictions: Problems in specific populations.. *Psychology of Addictive Behaviors*. 1993/09//. 7:147	Level 1, Form Title/abstract screen
2881	**M. Dawn Terrell**. Ethnocultural factors and substance abuse: Toward culturally sensitive treatment models.. *Psychology of Addictive Behaviors*. 1993/09//. 7:162	Level 2, Form Full Text Screening
2882	**Mark Olfson**. The array of psychiatric services in general hospitals.. *General Hospital Psychiatry*. 1993/09//. 15:277	Level 1, Form Title/abstract screen
2884	**Raymond Sanchez Mayers, Federico Jr. Souflee, Carann Simpson Feazell**. Prevention and treatment of Hispanic substance abuse: A workplace perspective.. *Hispanic substance abuse*.. 1993///. #volume#:175	Level 1, Form Title/abstract screen
2885	**Allen Zweben, David Barrett**. Brief couples treatment for alcohol problems.. *Treating alcohol problems: Marital and family interventions*.. 1993///. #volume#:353	Level 1, Form Title/abstract screen
2887	**Jaime Inclan, Miguel Hernandez**. Cross-cultural perspectives and codependence: The case of poor Hispanics.. *American Journal of Orthopsychiatry*. 1992/04//. 62:245	Level 1, Form Title/abstract screen
2890	**John B. Peachey, J. Spencer Madden, D. Adrian Wilkinson, Martha Sanchez-Craig, Charles P. O'Brien, Anna Rose Childress, George E. Vaillant, D. Colin Drummond, W. Falkowski**. Therapeutic skills.. *The international handbook of addiction behaviour*.. 1991///. #volume#:207	Level 1, Form Title/abstract screen
2891	**Carole L. Donovan**. Factors predisposing, enabling and reinforcing routine screening of patients for preventing fetal alcohol syndrome: A survey of New Jersey physicians.. *Journal of Drug Education*. 1991///. 21:35	Level 1, Form Title/abstract screen
2892	**Patrick B. Johnson**. Review of Alcoholism in minority populations.. *Psychology of Addictive Behaviors*. 1991///. 5:97	Level 1, Form Title/abstract screen
2893	**Alice S. Honig, Annette E. Pfannenstiel**. Difficulties in reaching low-income new fathers: Issues and cases.. *Early Child Development and Care*. 1991///. 77:115	Level 1, Form Title/abstract screen
2894	**Christine M. Nezu, Arthur M. Nezu, Patricia Arean**. Assertiveness and problem-solving training for mildly mentally retarded persons with dual diagnoses.. *Research in Developmental Disabilities*. 1991///. 12:371	Level 1, Form Title/abstract screen
2895	**L. F. Lowenstein**. The peer group promoting socialised behaviour: How can the peer group be mobilised to counteract and remedy negative behaviour?. *Education Today*. 1989///. 39:27	Level 1, Form Title/abstract screen
2896	**Kaj Noschis**. Testing a self-help instrument with early-risk alcohol consumers in general practice: A progress report.. *Contemporary Drug Problems: An Interdisciplinary Quarterly*. 1988///Fal 1988. 15:365	Level 1, Form Title/abstract screen
2898	**M. D. Jackson, Duane Brown**. Use of Systematic Training for Effective Parenting (STEP) with elementary school parents.. *School Counselor*. 1986/11//. 34:100	Level 1, Form Title/abstract screen
2901	**Robert Q. Dana**. Pretreatment assertion levels as they relate to treatment outcome in an alcohol abusing sample.. *Dissertation Abstracts International*. 1985/09//. 46:956	Level 1, Form Title/abstract screen
2903	**Joseph Westermeyer, John Neider**. Cultural affiliation among American Indian alcoholics: Correlations and change over a ten year period.. *Journal of*	Level 1, Form Title/abstract

	Operational Psychiatry. 1985///. 16:17	screen
2904	**Paul A. Mider**. Patient–treatment interaction: Person versus thing centeredness of addicts in methadone and drug-free approaches.. *International Journal of the Addictions*. 1985///. 20:1191	Level 1, Form Title/abstract screen
2905	**John Rogers, Margaret Durkin**. The semi-structured genogram interview. I: Protocol. II: Evaluation.. *Family Systems Medicine*. 1984///Sum 1984. 2:176	Level 1, Form Title/abstract screen
2906	**M. Keith Langley**. Post-traumatic stress disorders among Vietnam combat veterans.. *Social Casework*. 1982/12//. 63:593	Level 1, Form Title/abstract screen
2907	**Kenneth M. Ralph**. Asserting rights: A seductive option.. *Personnel & Guidance Journal*. 1982/01//. 60:328	Level 1, Form Title/abstract screen
2908	**David M. McElroy**. A modified marathon with voluntarily institutionalized substance abusers: Effects on psychopathology, self-actualization and ward behavior.. *Dissertation Abstracts International*. 1976/05//. 36:7213	Level 1, Form Title/abstract screen
2909	**George de Leon, D. Vincent Biase**. Encounter group: Measurement of systolic blood pressure.. *Psychological Reports*. 1975/10//. 37:439	Level 1, Form Title/abstract screen
2910	**Peter W. Bradshaw**. The problem of cigarette smoking and its control.. *International Journal of the Addictions*. 1973///. 8:353	Level 1, Form Title/abstract screen
2911	. Medical school education on abuse of alcohol and other psychoactive drugs.. *JAMA: Journal of the American Medical Association*. 1972/03//. 219:1746	Level 1, Form Title/abstract screen
2912	**Laney K. Jones, Rebecca Pulk, Michael R. Gionfriddo, Michael A. Evans, Dean Parry**. Utilizing big data to provide better health at lower cost.. *American Journal of Health-System Pharmacy*. 2018/04//4/1/2018. 75:427	Level 1, Form Title/abstract screen
2915	**Corinne Ann Coppinger**. Collaborative care in a rural setting for a pregnant woman with heroin addiction.. *Women's Healthcare: A Clinical Journal for NPs*. 2017/02//. 5:28	Level 2, Form Full Text Screening
2916	**Henk Den Ouden, Rimke C. Vos, Guy E. H. M. Rutten**. Effectiveness of shared goal setting and decision making to achieve treatment targets in type 2 diabetes patients: A cluster-randomized trial (OPTIMAL).. *Health Expectations*. 2017/10//. 20:1172	Level 1, Form Title/abstract screen
2917	**Susan C. Walley, Grant M. Mussman, Michele Lossius, Kristin A. Shadman, Lauren Destino, Matthew Garber, Shawn L. Ralston**. Implementing Parental Tobacco Dependence Treatment Within Bronchiolitis QI Collaboratives.. *Pediatrics*. 2018/06//. 141:1	Level 1, Form Title/abstract screen
2918	**Ilene A. Claudius, Shoma Desai, Ebony Davis, Sean Henderson**. Case-controlled Analysis of Patient-based Risk Factors for Assault in the Healthcare Workplace.. *Western Journal of Emergency Medicine: Integrating Emergency Care with Population Health*. 2017/10//. 18:1153	Level 1, Form Title/abstract screen
2919	**Daniel L. Hall, Inga T. Lennes, Alaina Carr, Justin R. Eusebio, Gloria Y. Yeh, Elyse R. Park**. Lung Cancer Screening Uncertainty among Patients Undergoing LDCT.. *American Journal of Health Behavior*. 2018/01//Jan/Feb2018. 42:69	Level 1, Form Title/abstract screen
2920	**Jessica L. Norman, Miranda E. Kroehl, Huong Mindy Lam, Carmen L. Lewis, Chelsea N. Mitchell, Cindy L. O'Bryant, Katy E. Trinkley**.	Level 1, Form Title/abstract

	Implementation of a pharmacist-managed clinic for patients with chronic nonmalignant pain.. *American Journal of Health-System Pharmacy*. 2017/08/15/. 74:1229	screen
2922	**Felicity A. E. Homsted, Chelsea E. Magee, Noah Nesin**. Population health management in a small health system: Impact of controlled substance stewardship in a patient-centered medical home.. *American Journal of Health-System Pharmacy*. 2017/09/15/. 74:1468	Level 1, Form Title/abstract screen
2927	**Anna E. Austin, Meghan E. Shanahan**. Association of childhood abuse and neglect with prescription opioid misuse: Examination of mediation by adolescent depressive symptoms and pain.. *Children & Youth Services Review*. 2018/02//. 86:84	Level 1, Form Title/abstract screen
2929	**Glenn Albright, Craig Bryan, Cyrille Adam, Jeremiah McMillan, Kristen Shockley**. Using Virtual Patient Simulations to Prepare Primary Health Care Professionals to Conduct Substance Use and Mental Health Screening and Brief Intervention.. *Journal of the American Psychiatric Nurses Association*. 2018/05//May/Jun2018. 24:247	Level 1, Form Title/abstract screen
2940	**BEVERLEY BOSTOCK COX**. Asthma-COPD overlap: diagnosis and management.. *Practice Nurse*. 2018/03//. 48:14	Level 1, Form Title/abstract screen
2941	**Ashley Tewksbury, Kevin M. Bozymski, Laura Ruekert, Cheen Lum, Elizabeth Cunningham, Frank Covington**. Development of Collaborative Drug Therapy Management and Clinical Pharmacy Services in an Outpatient Psychiatric Clinic.. *Journal of Pharmacy Practice*. 2018/06//. 31:272	Level 1, Form Title/abstract screen
2942	**Cheyenne Newsome, Leslie Colip, Nathaniel Sharon, Jessica Conklin**. Incorporating a pharmacist into an interprofessional team providing transgender care under a medical home model.. *American Journal of Health-System Pharmacy*. 2017/02//2/1/2017. 74:135	Level 1, Form Title/abstract screen
2943	**Margaret B. Nolan, Katherine E. Kemper, Thomas J. Glynn, Richard D. Hurt, J. Taylor Hays**. Tobacco Dependence Treatment Grants: A Collaborative Approach to the Implementation of WHO Tobacco Control Initiatives.. *Journal of Environmental & Public Health*. 2018/03/22/. #volume#:1	Level 1, Form Title/abstract screen
2944	**Lili Li, Shimin Zhu, Nick Tse, Samson Tse, Paul Wong**. Effectiveness of motivational interviewing to reduce illicit drug use in adolescents: a systematic review and meta-analysis.. *Addiction*. 2016/05//. 111:795	Level 1, Form Title/abstract screen
2946	**John D. Cramer, Brad Wisler, Christopher J. Gouveia**. Opioid Stewardship in Otolaryngology: State of the Art Review.. *Otolaryngology-Head & Neck Surgery*. 2018/05//. 158:817	Level 1, Form Title/abstract screen
2947	**Michele C. Walsh, Moira Crowley, Scott Wexelblatt, Susan Ford, Pierce Kuhnell, Heather C. Kaplan, Richard McClead, Maurizio Macaluso, Carole Lannon**. Ohio Perinatal Quality Collaborative Improves Care of Neonatal Narcotic Abstinence Syndrome.. *Pediatrics*. 2018/04//. 141:1	Level 1, Form Title/abstract screen
2952	**Leontine van der Meer, Anna Petra Nieboer, Harry Finkenflügel, Jane Murray Cramm**. The importance of person‐centred care and co‐creation of care for the well‐being and job satisfaction of professionals working with people with intellectual disabilities.. *Scandinavian Journal of Caring Sciences*. 2018/03//. 32:76	Level 1, Form Title/abstract screen
2953	**Veronica L. Ford**. Measuring Patient Satisfaction in New Mothers with Substance Use Disorders: A Correlative Investigation.. *Health Science Journal*. 2016/10//. 10:1	Level 1, Form Title/abstract screen

2956	. Bibliography.. *Progress in Palliative Care*. 2018/06//. 26:148	Level 1, Form Title/abstract screen
2957	**Christina Powers, Megan Comfort, Andrea M. Lopez, Alex H. Kral, Owen Murdoch, Jennifer Lorvick**. Addressing Structural Barriers to HIV Care among Triply Diagnosed Adults: Project Bridge Oakland.. *Health & Social Work*. 2017/05//. 42:e53	Level 1, Form Title/abstract screen
2960	**Jeanne E. Savage, Jessica E. Salvatore, Fazil Aliev, Alexis C. Edwards, Matthew Hickman, Kenneth S. Kendler, John Macleod, Antti Latvala, Anu Loukola, Jaakko Kaprio, Richard J. Rose, Grace Chan, Victor Hesselbrock, Bradley T. Webb, Amy Adkins, Tim B. Bigdeli, Brien P. Riley, Danielle M. Dick**. Polygenic Risk Score Prediction of Alcohol Dependence Symptoms Across Population‐Based and Clinically Ascertained Samples.. *Alcoholism: Clinical & Experimental Research*. 2018/03//. 42:520	Level 1, Form Title/abstract screen
2962	**Marie A. Fioravanti, Holly Hagle, Kathy Puskar, Emily Knapp, Irene Kane, Dawn Lindsay, Lauren Terhorst, Ann M. Mitchell**. Creative Learning Through the Use of Simulation to Teach Nursing Students Screening, Brief Intervention, and Referral to Treatment for Alcohol and Other Drug Use in a Culturally Competent Manner.. *Journal of Transcultural Nursing*. 2018/07//. 29:387	Level 1, Form Title/abstract screen
2963	**Guido Mannaioni, Cecilia Lanzi, Michela Lotti, Valentina Galli, Arianna Totti, Ilaria Pacileo, Maria Sili, Chiara Pracucci, Arianna Dilaghi, Lara Bertieri, Mariarita Quaranta, Francesco Orsini, Brunella Occupati, Assia Michahelles, Riccardo Ciuti, Elisa Bianchini, Giancarlo Fabbro, Annibale Biggeri, Emanuela Masini, Flavio Moroni**. Methadone Dose Adjustments, Plasma R-Methadone Levels and Therapeutic Outcome of Heroin Users: A Randomized Clinical Trial.. *European Addiction Research*. 2018/03//. 24:9	Level 1, Form Title/abstract screen
2964	**Mickey Sperlich, Julia S. Seng, Yang Li, Julie Taylor, Caroline Bradbury-Jones**. Integrating Trauma-Informed Care Into Maternity Care Practice: Conceptual and Practical Issues.. *Journal of Midwifery & Women's Health*. 2017/11//Nov/Dec2017. 62:661	Level 1, Form Title/abstract screen
2965	**Sadaaki Fukui, Michelle P. Salyers, Charlie Rapp, Richard Goscha, Leslie Young, Ally Mabry**. Supporting shared decision making beyond consumer-prescriber interactions: Initial development of the CommonGround fidelity scale.. *American Journal of Psychiatric Rehabilitation*. 2016/07//Jul-Sep2016. 19:252	Level 1, Form Title/abstract screen
2967	**Deborah Rigby**. The Case For.. *Journal of Pharmacy Practice & Research*. 2018/06//. 48:280	Level 1, Form Title/abstract screen
2969	**KENT MOORE, BARBARA HAYS**. Coding Changes for Family Medicine in 2018.. *Family Practice Management*. 2018/01//Jan/Feb2018. 25:5	Level 1, Form Title/abstract screen
2970	**Bill Reddy**. COMBATING DRUG ADDICTION & THE OPIOID CRISIS: AN ANALYSIS.. *Acupuncture Today*. 2018/02//. 19:1	Level 1, Form Title/abstract screen
2974	**Nicky Bundy**. The care programme approach and the politics of 'personality disorder'.. *Mental Health Practice*. 2017/07//. 20:32	Level 1, Form Title/abstract screen
2975	**GARY A. ENOS**. A lesson in LANGUAGE.. *Addiction Professional*. 2017///Spring2017. 15:10	Level 1, Form Title/abstract

		screen
2977	**Naweed I. Chowdhury, Jess C. Mace, Timothy L. Smith, Luke Rudmik**. What drives productivity loss in chronic rhinosinusitis? A SNOT-22 subdomain analysis.. *Laryngoscope*. 2018/01//. 128:23	Level 1, Form Title/abstract screen
2981	**Anthony Estreet, Paul Archibald, M. Taqi Tirmazi, Sapphire Goodman, Tracy Cudjoe**. Exploring social work student education: The effect of a harm reduction curriculum on student knowledge and attitudes regarding opioid use disorders.. *Substance Abuse*. 2017/10//Oct-Dec2017. 38:369	Level 1, Form Title/abstract screen
2982	**Lisa Carter-Harris, DuyKhanh Pham Ceppa, Nasser Hanna, Susan M. Rawl**. Lung cancer screening: what do long-term smokers know and believe?. *Health Expectations*. 2017/02//. 20:59	Level 1, Form Title/abstract screen
2983	**Kathryn R. Puskar,Heeyoung Lee, Ann M. Mitchell, Irene Kane, Susan A. Albrecht, Linda Rose Frank, Holly Hagle, Dawn L. Lindsay, Martin P. Houze**. Interprofessional Collaborative Education for Substance Use Screening: Rural Areas and Challenges.. *Online Journal of Rural Nursing & Health Care*. 2016/01//. 16:76	Level 1, Form Title/abstract screen
2985	**Rebekah Blowers**. MILITARY VETERANS AND MASSAGE THERAPY.. *Massage Therapy Journal*. 2018///Spring2018. 57:51	Level 1, Form Title/abstract screen
2987	**Rhona Reardon, Sarah Grogan**. Talking about smoking cessation with pregnant women: Exploring midwives' accounts.. *British Journal of Midwifery*. 2016/01//. 24:38	Level 2, Form Full Text Screening
2988	**Kristoffer Nordheim, Espen Walderhaug, Ståle Alstadius, Ann Kern-Godal, Espen Arnevik, Fanny Duckert**. Young adults' reasons for dropout from residential substance use disorder treatment.. *Qualitative Social Work*. 2018/01//. 17:24	Level 1, Form Title/abstract screen
2989	**Guilhème Pérodeau, Émilie Grenon, Sébastien Grenier, Kieron O'Connor**. Systemic model of chronic benzodiazepine use among mature adults.. *Aging & Mental Health*. 2016/04//. 20:380	Level 1, Form Title/abstract screen
2990	**Laurie Knis Matthews, Claire M. Mulry, Lynne Richard**. Matthews Model of Clinical Reasoning: A Systematic Approach to Conceptualize Evaluation and Intervention.. *Occupational Therapy in Mental Health*. 2017/10//Oct-Dec2017. 33:360	Level 1, Form Title/abstract screen
2991	**Alison Knopf**. Pharmacies and buprenorphine prescribing: Team effort.. *Alcoholism & Drug Abuse Weekly*. 2018/04/16/. 30:3	Level 1, Form Title/abstract screen
2992	**Julie Edwards, Jenna Butner**. ADOLESCENCE AND ALCOHOL USE DISORDERS.. *Family Doctor: A Journal of the New York State Academy of Family Physicians*. 2017///Winter2017. 5:22	Level 1, Form Title/abstract screen
2994	**Jillian Ireland, Ruth Evans, Rachel Buisson-Lex**. 'Bump, Baby and Beyond': Participant-led antenatal sessions using creative collaboration.. *British Journal of Midwifery*. 2016/09//. 24:650	Level 1, Form Title/abstract screen
2995	**Elizabeth Mollard, Diane Brage Hudson**. Nurse-Led Trauma-Informed Correctional Care for Women.. *Perspectives in Psychiatric Care*. 2016/07//. 52:224	Level 1, Form Title/abstract screen
2997	**Sarah Carter Narendorf**. Intersection of homelessness and mental health: A mixed methods study of young adults who accessed psychiatric emergency services.. *Children & Youth Services Review*. 2017/10//. 81:54	Level 1, Form Title/abstract screen

3001	**Christine Baker, Marianna Bruno, Laura Grant, Chloe Johnson, Bryan Bennett, Elaine Brohan, Birol Emir, Christine L Baker**. Content Validity of a Willingness to Quit Tool for Use with Current Smokers in Clinical Practice.. *Advances in Therapy*. 2017/10//. 34:2295	Level 1, Form Title/abstract screen
3003	**Alain H. Romanos, Ramzi V. Abou-Arraj, Stephanie E. Cruz, Zeina A. K. Majzoub**. Clinical and Patient-Centered Outcomes Following Treatment of Multiple Gingival Recessions Using Acellular Dermal Matrix Allografts.. *International Journal of Periodontics & Restorative Dentistry*. 2017/11//Nov/Dec2017. 37:842	Level 1, Form Title/abstract screen
3004	**Jane Murray Cramm, Anna Petra Nieboer**. Validation of an instrument to assess the delivery of patient-centred care to people with intellectual disabilities as perceived by professionals.. *BMC Health Services Research*. 2017/07/11/. 17:1	Level 1, Form Title/abstract screen
3005	**Megan Shen, Heidi Hamann, Anna Thomas, Jamie Ostroff, Megan Johnson Shen, Heidi A Hamann, Anna J Thomas, Jamie S Ostroff**. Association between patient-provider communication and lung cancer stigma.. *Supportive Care in Cancer*. 2016/05//. 24:2093	Level 1, Form Title/abstract screen
3010	**McLean D. Pollock, Sherri L. Green**. Effects of a Rural Family Drug Treatment Court Collaborative on Child Welfare Outcomes: Comparison Using Propensity Score Analysis.. *Child Welfare*. 2015/05//. 94:139	Level 1, Form Title/abstract screen
3013	**Brendan McCormack, Marit Borg, Shaun Cardiff, Jan Dewing, Gaby Jacobs, Angie Titchen, Famke van Lieshout, Valerie Wilson**. A Kaleidoscope of Hope: Exploring Experiences of Hope Among Service Users and Informal Carers in Health Care Contexts.. *Journal of Holistic Nursing*. 2017/09//. 35:247	Level 1, Form Title/abstract screen
3016	**Claire Fitzpatrick**. What do we know about girls in the care and criminal justice systems?. *Safer Communities*. 2017/07//. 16:134	Level 1, Form Title/abstract screen
3017	**Stefan G. Kertesz**. The Unexpected Urine Test: A Matter Far From Simple.. *Journal of Addiction Medicine*. 2017/11//Nov/Dec2017. 11:417	Level 1, Form Title/abstract screen
3018	**Laurie Abler, Kathleen J. Sikkema, Melissa H. Watt, Nathan B. Hansen, Patrick A. Wilson, Arlene Kochman**. Depression and HIV Serostatus Disclosure to Sexual Partners Among Newly HIV-Diagnosed Men Who Have Sex with Men.. *AIDS Patient Care & STDs*. 2015/10//. 29:550	Level 1, Form Title/abstract screen
3021	**John F. Kelly**. Tens of millions successfully in long-term recovery-let us find out how they did it.. *Addiction*. 2017/05//. 112:762	Level 1, Form Title/abstract screen
3022	**Kamilah Konrad**. Basics of Patient Navigation.. *Journal of Oncology Navigation & Survivorship*. 2016/05//. 7:20	Level 1, Form Title/abstract screen
3023	**Emma Wilkinson**. Promoting smoking cessation as an essential part of diabetes care.. *Practice Nursing*. 2015/01//. 26:36	Level 1, Form Title/abstract screen
3025	**Travis Hales, Nancy Kusmaul, Thomas Nochajski**. Exploring the Dimensionality of Trauma-Informed Care: Implications for Theory and Practice.. *Human Service Organizations: Management, Leadership & Governance*. 2017/06//Jun-Aug2017. 41:317	Level 1, Form Title/abstract screen
3026	**Patricia Leighton, LeAnn Perkins**. Long-term cancer survivorship nurse	Level 1, Form

	practitioner care model promotes patient quality of life.. *American Nurse Today*. 2015/06//. 10:37	Title/abstract screen
3028	**Lori Ebert, Carol Malte, Kim Hamlett-Berry, Jean Beckham, Miles McFall, Andrew Saxon**. Use of a Learning Collaborative to Support Implementation of Integrated Care for Smoking Cessation for Veterans With Posttraumatic Stress Disorder.. *American Journal of Public Health*. 2014/10//. 104:1935	Level 1, Form Title/abstract screen
3029	**Michael T. McKay, James R. Andretta, Jon C. Cole**. How Individuals Feel About the Past, Present, and Future Bears Little Relation to Alcohol-Related Problems, Anxiety, and Depression: A Person-Centered Analysis in a University Sample.. *Substance Use & Misuse*. 2017/05//. 52:734	Level 1, Form Title/abstract screen
3030	**David Otiashvili, Irma Kirtadze, Kevin E. O'Grady, William Zule, Evgeny Krupitsky, Wendee M. Wechsberg, Hendrée E. Jones**. Comprehensive women-centered treatment for substance use disorders in Georgia: current status and future directions.. *Journal of Substance Use*. 2015/10//. 20:367	Level 2, Form Full Text Screening
3031	**Johanna Taylor, David Shiers**. Don't Just Screen -- Intervene: Protecting the cardiometabolic health of people with severe mental illness.. *Diabetes & Primary Care*. 2017/09//. 19:217	Level 1, Form Title/abstract screen
3034	**Brandon Snead, Debra Pakstis, Breonna Evans, Rhonda Nelson**. The Use of Creative Writing Interventions in Substance Abuse Treatment.. *Therapeutic Recreation Journal*. 2015/03//. 49:179	Level 1, Form Title/abstract screen
3039	**Ranak B. Trivedi, Edward P. Post, Haili Sun, Andrew Pomerantz, Andrew J. Saxon, John D. Piette, Charles Maynard, Bruce Arnow, Idamay Curtis, Stephan D. Fihn, Karin Nelson**. Prevalence, Comorbidity, and Prognosis of Mental Health Among US Veterans.. *American Journal of Public Health*. 2015/12//. 105:2564	Level 1, Form Title/abstract screen
3042	**Amy O'Donnell, Catherine Haighton, David Chappel, Colin Shevills, Eileen Kaner**. Impact of financial incentives on alcohol intervention delivery in primary care: a mixed-methods study.. *BMC Family Practice*. 2016/11/25/. 17:1	Level 1, Form Title/abstract screen
3046	**Phillip Coffin, Glenn-Milo Santos, Grant Colfax, Moupali Das, Tim Matheson, Erin DeMicco, James Dilley, Eric Vittinghoff, Jerris Raiford, Monique Carry, Jeffrey Herbst**. Adapted Personalized Cognitive Counseling for Episodic Substance-Using Men Who Have Sex with Men: A Randomized Controlled Trial.. *AIDS & Behavior*. 2014/07//. 18:1390	Level 1, Form Title/abstract screen
3047	**Charl Els**. Interrupting the disease of tobacco addiction.. *Canadian Journal of Dental Hygiene*. 2014/11//. 48:167	Level 1, Form Title/abstract screen
3049	**Gary Enos**. Many buprenorphine prescribers falling well short of patient limits, study shows.. *Alcoholism & Drug Abuse Weekly*. 2017/11/13/. 29:1	Level 1, Form Title/abstract screen
3050	**Taeho Greg Rhee, Roni L. Evans, Donna D. McAlpine, Pamela Jo Johnson**. Racial/Ethnic Differences in the Use of Complementary and Alternative Medicine in US Adults With Moderate Mental Distress.. *Journal of Primary Care & Community Health*. 2017/04//. 8:43	Level 1, Form Title/abstract screen
3051	**Coral Sirdifield, Susan Chipchase, Sara Owen, Aloysius Siriwardena**. A Systematic Review and Meta-Synthesis of Patients' Experiences and Perceptions of Seeking and Using Benzodiazepines and Z-Drugs: Towards Safer Prescribing.. *Patient*. 2017/02//. 10:1	Level 1, Form Title/abstract screen
3053	**Anita Ho**. Reconciling Patient Safety and Epistemic Humility: An Ethical Use	Level 1, Form

	of Opioid Treatment Plans.. *Hastings Center Report*. 2017/05//. 47:34	Title/abstract screen
3056	**Eva Biringer, Larry Davidson, Bengt Sundfør, Torleif Ruud, Marit Borg**. Service users' expectations of treatment and support at the Community Mental Health Centre in their recovery.. *Scandinavian Journal of Caring Sciences*. 2017/09//. 31:505	Level 1, Form Title/abstract screen
3058	**Juliana MacÃªdo MagalhÃ£es, Claudete Ferreira de Souza Monteiro, Maria do Livramento Fortes Figueiredo**. The teenagers' idea about the prevention of crack.. *Revista de Pesquisa: Cuidado e Fundamental*. 2013/12/15/dez2013 special issue. 5:28	Level 1, Form Title/abstract screen
3059	**Patricia E. Fikar, Kent A. Edlund, Dave Newell**. Current preventative and health promotional care offered to patients by chiropractors in the United Kingdom: a survey.. *Chiropractic & Manual Therapies*. 2015/03//. 23:1	Level 1, Form Title/abstract screen
3061	**Brooke Faria da Cunha**. Ethics and Undertreatment of Pain in Patients with a History of Drug Abuse.. *Med-Surg Matters*. 2015/01//Jan/Feb2015. 24:4	Level 1, Form Title/abstract screen
3063	**Kristina B. Wolff, Christina Soncrant, Peter D. Mills, Robin R. Hemphill**. Flash Burns While on Home Oxygen Therapy: Tracking Trends and Identifying Areas for Improvement.. *American Journal of Medical Quality*. 2017/07//Jul/Aug2017. 32:445	Level 1, Form Title/abstract screen
3066	**Mary Perez, Kristen Yawea**. Recovery Practice Implementation on Adult/Geriatric Acute Psychiatric Units...APNA 31st Annual Conference, October 18-21, 2017, Phoenix, Arizona: Part I. *Journal of the American Psychiatric Nurses Association*. 2018/05//May/Jun2018. 24:280	Level 1, Form Title/abstract screen
3068	**Nancy K. Young, Julie Collins**. Special Foreword: Substance Use and Child Welfare.. *Child Welfare*. 2015/07//. 94:11	Level 1, Form Title/abstract screen
3070	**Herbert Fillmore, C. Annette DuBard, Grant A. Ritter, Carlos T. Jackson**. Health Care Savings with the Patient-Centered Medical Home: Community Care of North Carolina's Experience.. *Population Health Management*. 2014/06//. 17:141	Level 1, Form Title/abstract screen
3072	**Deborah W. Busch**. Clinical Management of the Breast-Feeding Mother-Infant Dyad in Recovery From Opioid Dependence.. *Journal of Addictions Nursing (Lippincott Williams & Wilkins)*. 2016/04//Apr-Jun2016. 27:68	Level 1, Form Title/abstract screen
3074	**Jennifer Percival**. How nurses can help patients with heart conditions to stop smoking.. *Nurse Prescribing*. 2013/09//. 11:442	Level 1, Form Title/abstract screen
3076	**Bob Price**. Discussing risk with patients.. *Nursing Standard*. 2017/04/12/. 31:53	Level 1, Form Title/abstract screen
3077	**Carol Strike, Tara Marie Watson**. Relationships between needle and syringe programs and police: An exploratory analysis of the potential role of in-service training.. *Drug & Alcohol Dependence*. 2017/06//. 175:51	Level 1, Form Title/abstract screen
3079	**J. Bold**. Nutrition and integrative approaches to infertility: improving patient experience and outcomes.. *Journal of Pelvic, Obstetric & Gynaecological Physiotherapy*. 2017///Spring2017. #volume#:28	Level 1, Form Title/abstract screen
3080	**John Hansen**. Opiates for Chronic Pain.. *Pain Practice*. 2017/01//. 17:147	Level 1, Form Title/abstract screen

3083	**Álvaro Camacho, Patricia González, Sheila Castañeda, Alan Simmons, Christina Buelna, Hector Lemus, Gregory Talavera**. Improvement in Depressive Symptoms Among Hispanic/Latinos Receiving a Culturally Tailored IMPACT and Problem-Solving Intervention in a Community Health Center.. *Community Mental Health Journal*. 2015/05//. 51:385	Level 1, Form Title/abstract screen
3085	**Ethel Bastos da Silva, Stella Maris de Mello Padoin, Lucila Amaral Carneiro Vianna**. Violência contra a mulher: limites e potencialidades da prática assistencial.. *Acta Paulista de Enfermagem*. 2013/12//. 26:608	Level 1, Form Title/abstract screen
3086	**Ethel Bastos da Silva, Stella Maris de Mello Padoin, Lucila Amaral Carneiro Vianna**. Violence against women: the limits and potentialities of care practice.. *Acta Paulista de Enfermagem*. 2013/12//. 26:608	Level 1, Form Title/abstract screen
3087	**Caroline Stephens, Nathan Sackett, Read Pierce, David Schopfer, Gabriela Schmajuk, Nicholas Moy, Melissa Bachhuber, Margaret I. Wallhagen, Sei J. Lee**. Transitional Care Challenges of Rehospitalized Veterans: Listening to Patients and Providers.. *Population Health Management*. 2013/10//. 16:326	Level 1, Form Title/abstract screen
3088	**Suzanne M. Peloquin, Carrie A. Ciro**. Population-Centered Life Skills Groups: Perceptions of Satisfaction and Engagement.. *American Journal of Occupational Therapy*. 2013/09//Sep/Oct2013. 67:594	Level 1, Form Title/abstract screen
3090	**Heather Howard**. Experiences of opioid-dependent women in their prenatal and postpartum care: Implications for social workers in health care.. *Social Work in Health Care*. 2016/01//. 55:61	Level 1, Form Title/abstract screen
3091	**Rakesh K. Chadda, Biswadeep Chatterjee**. Need for psychosocial interventions: From resistance to therapeutic alliance.. *Indian Journal of Psychiatry*. 2018/02/04/2018 Supplement. 60:S440	Level 1, Form Title/abstract screen
3092	**James Fricton, Jeffrey Crandall**. Solving the Access to Care Problem for Patients with Orofacial Pain.. *Journal of Oral & Facial Pain & Headache*. 2018///Winter2018. 32:e5	Level 1, Form Title/abstract screen
3096	**Jenny Edwins**. Supporting smoking cessation in pregnancy.. *British Journal of Midwifery*. 2013/03//. 21:174	Level 1, Form Title/abstract screen
3098	**Benjamin R. Nordstrom, Elizabeth C. Saunders, Bethany McLeman, Andrea Meier,Haiyi Xie, Chantal Lambert-Harris, Beth Tanzman, John Brooklyn, Gregory King, Nels Kloster, Clifton Frederick Lord, William Roberts, Mark P. McGovern**. Using a Learning Collaborative Strategy With Office-based Practices to Increase Access and Improve Quality of Care for Patients With Opioid Use Disorders.. *Journal of Addiction Medicine*. 2016/03//Mar/Apr2016. 10:117	Level 1, Form Title/abstract screen
3100	**Courtney S. Davis, Leigh Ann R. Ross, Lauren S. Bloodworth**. The Impact of Clinical Pharmacist Integration on a Collaborative Interdisciplinary Diabetes Management Team.. *Journal of Pharmacy Practice*. 2017/06//. 30:286	Level 1, Form Title/abstract screen
3101	**Melanie D. Parks**. Barriers to Clinical Practice.. *Journal of Doctoral Nursing Practice*. 2017/03//. 10:135	Level 1, Form Title/abstract screen
3103	**Kathryn A. Becker-Blease**. As the world becomes trauma–informed, work to do.. *Journal of Trauma & Dissociation*. 2017/03//Mar/Apr2017. 18:131	Level 1, Form Title/abstract screen
3104	**Lisa Carter-Harris, Susan Brandzel, Karen J. Wernli, Joshua A. Roth, Diana S. M. Buist**. A qualitative study exploring why individuals opt out of lung cancer screening.. *Family Practice*. 2017/04//4/1/2017. 34:238	Level 1, Form Title/abstract screen

3105	**Stephen J. Bartels, Renee Pepin, Lydia E. Gill**. The Paradox of Scarcity in a Land of Plenty: Meeting the Needs of Older Adults with Mental Health and Substance Use Disorders.. *Generations*. 2014///Fall2014. 38:6	Level 1, Form Title/abstract screen
3107	**Ottar Ness, Marit Borg, Randi Semb, Alain Topor**. "Negotiating partnerships:" parents' experiences of collaboration in community mental health and substance use services.. *Advances in Dual Diagnosis*. 2016/12//. 9:130	Level 1, Form Title/abstract screen
3108	**Richard Zheng, Maria Altieri, Jie Yang, Hao Chen, Aurora Pryor, Andrew Bates, Mark Talamini, Dana Telem, Maria S Altieri, Aurora D Pryor, Mark A Talamini, Dana A Telem**. Long-term incidence of contralateral primary hernia repair following unilateral inguinal hernia repair in a cohort of 32,834 patients.. *Surgical Endoscopy*. 2017/02//. 31:817	Level 1, Form Title/abstract screen
3109	**Lisa Carter-Harris, James E. Slaven, Patrick Monohan, Susan M. Rawl**. Development and Psychometric Evaluation of the Lung Cancer Screening Health Belief Scales.. *Cancer Nursing*. 2017/05//May/Jun2017. 40:237	Level 1, Form Title/abstract screen
3111	**Virgil Dickson**. Hospitals turn to collaborative agreements to battle addiction crisis.. *Modern Healthcare*. 2018/01/15/. 48:0006	Level 1, Form Title/abstract screen
3115	**Rosalind Abdool, Michael Szego, Daniel Buchman, Leah Justason, Sally Bean, Ann Heesters, Hannah Kaufman, Bob Parke, Frank Wagner, Jennifer Gibson**. Difficult healthcare transitions.. *Nursing Ethics*. 2016/11//. 23:770	Level 1, Form Title/abstract screen
3119	**Jeffrey Abracen, Jan Looman, Meaghan Ferguson**. Substance abuse among sexual offenders: review of research and clinical implications.. *Journal of Sexual Aggression*. 2017/11//. 23:235	Level 1, Form Title/abstract screen
3121	**Stephen F. Butler, Kevin L. Zacharoff, Sadaf Charity, Ryan A. Black, Emma Chung, Antje Barreveld, Molly S. Clark, Robert N. Jamison**. Impact of an Electronic Pain and Opioid Risk Assessment Program: Are There Improvements in Patient Encounters and Clinic Notes?. *Pain Medicine*. 2016/11//. 17:2047	Level 1, Form Title/abstract screen
3122	**Marc Pimsler**. The cunning, powerful addiction that is VALIDATION.. *Addiction Professional*. 2015///Spring2015. 13:22	Level 1, Form Title/abstract screen
3123	**Jim McCambridge, Stephen Rollnick**. Big issues.. *Addiction*. 2014/07//. 109:1063	Level 1, Form Title/abstract screen
3124	**Evalina van Wijk, Annalene Traut, Hester Julie**. Environmental and nursing-staff factors contributing to aggressive and violent behaviour of patients in mental health facilities.. *Curationis*. 2014/09//. 37:1	Level 1, Form Title/abstract screen
3125	**Rachel A. Sebastian, Mary M. Ramos**. 265 - Adolescent Substance Use and Quality of Care: Self-Reported Substance Use, Receipt of Anticipatory Guidance, and Patient-Centered Care among School-Based Health Center Users in New Mexico.. *Journal of Adolescent Health*. 2018/02/02/2018 Supplement. 62:S134	Level 1, Form Title/abstract screen
3126	**Sala Horowitz**. Treating Veterans' Chronic Pain and Mental Health Disorders: An Integrative, Patient-Centered Approach.. *Alternative & Complementary Therapies*. 2013/06//. 19:133	Level 1, Form Title/abstract screen
3127	**Victoria Adewale, Dianne Ritchie, Sarah Everhart Skeels**. African-American and African perspectives on mental health: A pilot study of the pre and post colonial and slavery influences and their implications on mental health.. *Journal*	Level 1, Form Title/abstract screen

	of Communication in Healthcare. 2016/07//. 9:78	
3131	. In Case You Haven't Heard.. *Alcoholism & Drug Abuse Weekly*. 2018/04/30/. 30:8	Level 1, Form Title/abstract screen
3132	**Mahesh Jayaram, Malcolm Hopwood, Christos Pantelis**. Can we raise the standard of care in treating schizophrenia: A cautionary note!. *Australian & New Zealand Journal of Psychiatry*. 2017/06//. 51:557	Level 1, Form Title/abstract screen
3133	**Ahmed Rashid**. Yonder: Chronic pain, asthma, obstructive sleep apnoea, and methadone prescribing.. *British Journal of General Practice*. 2018/05//. 68:237	Level 1, Form Title/abstract screen
3136	**John Epling**. THE "OTHER" PREVENTIVE SERVICES: Behavioral Risk Factor Counseling and Chemoprophylaxis Recommendations from the US Preventive Services Task Force.. *Family Doctor: A Journal of the New York State Academy of Family Physicians*. 2014///Spring2014. #volume#:17	Level 1, Form Title/abstract screen
3143	**Freyr Sigmundsson, Bo Jönsson, Björn Strömqvist, Freyr Gauti Sigmundsson**. Determinants of patient satisfaction after surgery for central spinal stenosis without concomitant spondylolisthesis: a register study of 5100 patients.. *European Spine Journal*. 2017/02//. 26:473	Level 1, Form Title/abstract screen
3145	**W. Richard Cowling**. Where Is Holistic Nursing?. *Journal of Holistic Nursing*. 2018/03//. 36:4	Level 1, Form Title/abstract screen
3146	**Shane A. Phillips, Sharon Martino, Ross Arena**. Research Opportunities and Challenges in the Era of Healthy Living Medicine: Unlocking the Potential.. *Progress in Cardiovascular Diseases*. 2017/03//. 59:498	Level 1, Form Title/abstract screen
3147	**Maria Asunción González Mestre, Paloma Amil Bujan, Esther Gil Sanchez, Eva Maria Rodriguez Pérez, Juan Antonio Camus Heras, Carme Boix De la Casa, Waleska Badia Rafecas, Edith Garcia Solanes, Marta Palou Aligue, M. Carmen de Amo Castillo, Montserrat Agramunt Perello, Meritxell Feixes Betriu, Margarita Jorda Recort, Maria Rosa Garcia Cerdan, Cristina Sanz Espuny, M. Ángeles Hierro Lapeyre, Julia Mena Sánchez, Maria del Carmen Rodriguez Pérez, Laura Muñoz Alfonso, Violeta Pérez Cáceres**. 10-year experience of expert patient programme Catalonia. Impact and results.. *International Journal of Integrated Care (IJIC)*. 2016/12/02/2016 Supplement. 16:1	Level 1, Form Title/abstract screen
3148	**Mark D. Litt, Ronald M. Kadden, Howard Tennen, Elise Kabela-Cormier**. Network Support II: Randomized controlled trial of Network Support treatment and cognitive behavioral therapy for alcohol use disorder.. *Drug & Alcohol Dependence*. 2016/08//. 165:203	Level 1, Form Title/abstract screen
3149	**Lee L. Prina**. Primary Care: Foundations Aim To Improve Access And Quality.. *Health Affairs*. 2017/10//. 36:1852	Level 1, Form Title/abstract screen
3152	**Amy W. Baughman, Phyllis Brawarsky, Tracy Onega, Tor D. Tosteson,Qianfei Wang, Anna N. A. Tosteson, Jennifer S. Haas**. Medical Home Transformation and Breast Cancer Screening.. *American Journal of Managed Care*. 2016/11//. 22:e382	Level 1, Form Title/abstract screen
3155	**Laverne H. Stevens**. RELATING CLINICAL ASSESSMENT CONSIDERATIONS TO EHR MEANINGFUL USE PART II...Electronic Health Record. *Counselor: The Magazine for Addiction Professionals*. 2014/06//. 15:70	Level 1, Form Title/abstract screen

3158	**Rhea E. Powell, Amanda Doty, Robin J. Casten, Barry W. Rovner, Kristin L. Rising**. A qualitative analysis of interprofessional healthcare team members' perceptions of patient barriers to healthcare engagement.. *BMC Health Services Research*. 2016/09/20/. 16:1	Level 1, Form Title/abstract screen
3159	**Deborah Duncan**. Quit smoking support.. *Independent Nurse*. 2012/12/03/. #volume#:30	Level 1, Form Title/abstract screen
3162	**Sara Bitting, Angela Nash, Ashley Ochoa**. Houston Recovery Initiative.. *Journal of Addictions Nursing (Lippincott Williams & Wilkins)*. 2016/04//Apr-Jun2016. 27:120	Level 2, Form Full Text Screening
3164	**Martin Härter, Anna-Lena Bartsch, Nina Egger, Hans-Helmut König, Levente Kriston, Holger Schulz, Michael Tiemann, Anna Levke Brütt, Angela Buchholz, Anna Levke Brütt**. Evaluating a collaborative smoking cessation intervention in primary care (ENTER): study protocol for a cluster-randomized controlled trial.. *Trials*. 2015/10/10/. 16:1	Level 1, Form Title/abstract screen
3165	**Nancy M. Lucero, Marian Bussey**. A Collaborative and Trauma-Informed Practice Model for Urban Indian Child Welfare.. *Child Welfare*. 2012/05//May/Jun2012. 91:89	Level 1, Form Title/abstract screen
3166	**Lisa Carter-Harris, Andy S.L. Tan, Ramzi G. Salloum, Kelly C. Young-Wolff**. Patient-provider discussions about lung cancer screening pre- and post-guidelines: Health Information National Trends Survey (HINTS).. *Patient Education & Counseling*. 2016/11//. 99:1772	Level 1, Form Title/abstract screen
3171	**Laura Markwick**. Male, Female, Other: Transgender and the Impact in Primary Care.. *Journal for Nurse Practitioners*. 2016/05//. 12:330	Level 1, Form Title/abstract screen
3172	**Anne Krueger**. Secrets YOUR DOCTOR WON'T TELL YOU.. *Arthritis Today*. 2013/09//Sep/Oct2013. 27:62	Level 1, Form Title/abstract screen
3176	**Hashem S. Abu Tariah, Razan T. Hamed, Hassan D. Al-Omari**. Jordanian client perspectives of substance abuse: Implications for occupational therapists.. *International Journal of Therapy & Rehabilitation*. 2015/12//. 22:566	Level 1, Form Title/abstract screen
3178	**Harriet Burgess, Joanne Marie Ford, Sarah Kendal**. Are mental health nurses natural smoking cessation practitioners?. *Mental Health Practice*. 2015/11//. 19:34	Level 1, Form Title/abstract screen
3180	**Rolf Gjestad, Johan Franck, Staffan Lindberg, Brit Haver**. Early Treatment for Women with Alcohol Addiction (EWA) Reduces Mortality: A Randomized Controlled Trial with Long-Term Register Follow-up.. *Alcohol & Alcoholism*. 2011/03//Mar/Apr2011. 46:170	Level 1, Form Title/abstract screen
3182	**Alison Knopf**. Congressional investigation into Alkermes opens.. *Alcoholism & Drug Abuse Weekly*. 2017/11/13/. 29:5	Level 1, Form Title/abstract screen
3183	**Doran D,Paterson J,Clark C,Srivastava R,Goering PN,Kushniruk AW,Bajnok I,Nagle L,Almost J,Carryer J**. A pilot study of an electronic interprofessional evidence-based care planning tool for clients with mental health problems and addictions.. *Worldviews on Evidence-Based Nursing*. 2010///2010 3rd Quarter. 7:174	Level 1, Form Title/abstract screen
3184	**Nichole M. Scaglione, Kimberly A. Mallett, Rob Turrisi, Racheal Reavy, Michael J. Cleveland, Sarah Ackerman**. Who Will Experience the Most Alcohol Problems in College? The Roles of Middle and High School Drinking	Level 1, Form Title/abstract screen

	Tendencies.. *Alcoholism: Clinical & Experimental Research*. 2015/10//. 39:2039	
3186	**Paul E. Terry, Erin LD Seaverson, Michael J. Staufacker, Akiko Tanaka**. The Effectiveness of a Telephone-Based Tobacco Cessation Program Offered as Part of a Worksite Health Promotion Program.. *Population Health Management*. 2011/06//. 14:117	Level 1, Form Title/abstract screen
3189	**Rebecca Bosworth**. STUDENT KNOWLEDGE OF PROVIDING CARE FOR WOMEN WHO EXPERIENCE ALCOHOL-USE DISORDERS.. *Australian Nursing & Midwifery Journal*. 2016/12//Dec2016/Jan2017. 24:37	Level 1, Form Title/abstract screen
3190	**Peter Cremer-Schaeffer, K. Broich**. Betäubungsmittelrecht und Palliativversorgung.. *Gesundheitsökonomie & Qualitätsmanagement*. 2015/10//. 20:216	Level 1, Form Title/abstract screen
3191	**Rickard Elmore**. An interventionist's VIEW of treatment centers.. *Addiction Professional*. 2015///Spring2015. 13:19	Level 1, Form Title/abstract screen
3196	**Jennifer Steel, David A Geller, Allan Tsung, J Wallis Marsh, Mary Amanda Dew, Michael Spring, Jonathan Grady, Sonja Likumahuwa, Andrea Dunlavy, Michael Youssef, Michael Antoni, Lisa H Butterfield, Richard Schulz, Richard Day, Vicki Helgeson, Kevin H Kim, T Clark Gamblin**. Randomized controlled trial of a collaborative care intervention to manage cancer-related symptoms: lessons learned.. *Clinical Trials*. 2011/06//. 8:298	Level 1, Form Title/abstract screen
3199	**DANIEL B. CARR**. PRESIDENT'S MESSAGE. Patients with Pain Need Less Stigma, Not More.. *Pain Medicine*. 2016/08//. 17:1391	Level 1, Form Title/abstract screen
3202	**Deborah Antai-Otong, Kristine Theis, Dee Dee Patrick**. Dual Diagnosis.. *Nursing Clinics of North America*. 2016/06//. 51:237	Level 1, Form Title/abstract screen
3205	**Resa M. Jones, Kara P. Wiseman, Marina Kharitonova**. Association between high school students' cigarette smoking, asthma and related beliefs: a population-based study.. *BMC Public Health*. 2016/09//9/1/2016. 16:1	Level 1, Form Title/abstract screen
3209	**Quanbeck AR,Madden L,Edmundson E,Ford JH 2nd,McConnell KJ,McCarty D,Gustafson DH, Andrew R Quanbeck, Lynn Madden, Eldon Edmundson, James H 2nd Ford, K John McConnell, Dennis McCarty, David H Gustafson**. A business case for quality improvement in addiction treatment: evidence from the NIATx collaborative.. *Journal of Behavioral Health Services & Research*. 2012/01//. 39:91	Level 2, Form Full Text Screening
3212	**Strike C,Rufo C**. Embarrassing, degrading, or beneficial: patient and staff perspectives on urine drug testing in methadone maintenance treatment.. *Journal of Substance Use*. 2010/10//. 15:303	Level 2, Form Full Text Screening
3213	**Jolynne 'Jo' Carter, Sandra Zawalski, Patrice V. Sminkey, Bruce Christopherson**. Assessing the Whole Person: Case Managers Take a Holistic Approach to Physical and Mental Health.. *Professional Case Management*. 2015/05//May/Jun2015. 20:140	Level 1, Form Title/abstract screen
3215	**Arnetz JE,Winblad U,Höglund AT,Lindahl B,Spångberg K,Wallentin L,Wang Y,Ager J,Arnetz BB**. Is patient involvement during hospitalization for acute myocardial infarction associated with post-discharge treatment outcome? An exploratory study.. *Health Expectations*. 2010/09//. 13:298	Level 1, Form Title/abstract screen
3218	**Pevzner ES,Robison S,Donovan J,Allis D,Spitters C,Friedman R,Ijaz**	Level 1, Form

	**K,Oeltmann JE**. Tuberculosis transmission and use of methamphetamines in Snohomish County, WA, 1991-2006.. *American Journal of Public Health*. 2010/12//. 100:2481	Title/abstract screen
3220	**Tina M. Trudel, Marcia J. Scherer, Eileen Elias**. Understanding Traumatic Brain Injury: An Introduction.. *Exceptional Parent*. 2011/07//. 41:33	Level 1, Form Title/abstract screen
3221	**Andreas Heinz, Tomislav Majić**. Effective and Neurobiologically Sound.. *Deutsches Aerzteblatt International*. 2015/10/09/. 112:681	Level 2, Form Full Text Screening
3222	**Gary Enos**. Study suggests viable alternative to mandate-driven OTP approaches.. *Alcoholism & Drug Abuse Weekly*. 2017/03/27/. 29:1	Level 2, Form Full Text Screening
3223	**Yong Liu, Janet B. Croft, Anne G. Wheaton, Dafna Kanny, Timothy J. Cunningham,Hua Lu, Stephen Onufrak, Ann M. Malarcher, Kurt J. Greenlund, Wayne H. Giles, Yong Liu, Hua Lu**. Clustering of Five Health-Related Behaviors for Chronic Disease Prevention Among Adults, United States, 2013.. *Preventing Chronic Disease*. 2016/05/26/. 13:1	Level 1, Form Title/abstract screen
3224	**Stephen W. Patrick, Robert E. Schumacher, Jeffrey D. Horbar, Madge E. Buus-Frank**. Improving Care for Neonatal Abstinence Syndrome.. *Pediatrics*. 2016/05//. 137:1	Level 1, Form Title/abstract screen
3225	**Andrea H. Kline-Simon, Constance Weisner, Stacy Sterling**. Point Prevalence of Co-Occurring Behavioral Health Conditions and Associated Chronic Disease Burden Among Adolescents.. *Journal of the American Academy of Child & Adolescent Psychiatry*. 2016/05//. 55:408	Level 1, Form Title/abstract screen
3226	**Kim Manley, Val Hills, Sheila Marriot**. Person-centred care: principle of nursing practice D.. *Nursing Standard*. 2011/04/06/. 25:35	Level 1, Form Title/abstract screen
3227	**Bryant-Jefferies R**. Alcohol misuse: a very human tragedy.. *Healthcare Counselling & Psychotherapy Journal*. 2010/10//. 10:22	Level 1, Form Title/abstract screen
3228	**Jeremy Ader, Christopher J. Stille, David Keller, Benjamin F. Miller, Michael S. Barr, James M. Perrin**. The Medical Home and Integrated Behavioral Health: Advancing the Policy Agenda.. *Pediatrics*. 2015/05//. 135:909	Level 1, Form Title/abstract screen
3229	**Jason D. Woollard, James E. Bost, Sara R. Piva, G. Kelley Fitzgerald, Mark W. Rodosky, James J. Irrgang**. The relationship of preoperative factors to patient-reported outcome in rotator cuff repair: a systematic review.. *Physical Therapy Reviews*. 2016/06//Jun-Dec2016. 21:138	Level 1, Form Title/abstract screen
3230	**Jane Carole Warner**. Behaviour change strategies for novice practice nurses.. *Primary Health Care*. 2010/11//. 20:25	Level 1, Form Title/abstract screen
3233	**LISA HAUK**. Management of Chronic Pain and Opioid Misuse: A Position Paper from the AAFP.. *American Family Physician*. 2017/04//4/1/2017. 95:458	Level 2, Form Full Text Screening
3237	**Fred J. Dyer**. Therapeutic Alliance: The Glue That Makes Therapy Work.. *Counselor: The Magazine for Addiction Professionals*. 2015/06//. 16:35	Level 1, Form Title/abstract screen
3241	**Stephanie V. Phan**. Medication adherence in patients with schizophrenia.. *International Journal of Psychiatry in Medicine*. 2016/02//. 51:211	Level 1, Form Title/abstract

		screen
3243	**Roman Shrestha, Tania B. Huedo-Medina, Michael M. Copenhaver**. Sex-Related Differences in Self-Reported Neurocognitive Impairment among High-Risk Cocaine Users in Methadone Maintenance Treatment Program.. *Substance Abuse: Research & Treatment*. 2015/01//. #volume#:17	Level 1, Form Title/abstract screen
3245	**Joanna L. Hart, Emily Pflug, Vanessa Madden, Scott D. Halpern**. Thinking Forward: Future-oriented Thinking among Patients with Tobacco-associated Thoracic Diseases and Their Surrogates.. *American Journal of Respiratory & Critical Care Medicine*. 2016/02//2/1/2016. 193:321	Level 1, Form Title/abstract screen
3249	**Handa K,Grace J,Trigoboff E,Olympia JL,Annalett D,Watson T,Poulose MC,Muzaffar T,Noyes FL,Kabatt A,Cushman S,Antonelli M,Baxter-Banks G,Newcomer D**. Continuing day treatment programs promote recovery in schizophrenia: a case-based study.. *Psychiatry (1550-5952)*. 2009/04//. 6:32	Level 1, Form Title/abstract screen
3250	**Madson M,Schumacher J,Bonnell M**. Motivational interviewing and alcohol.. *Healthcare Counselling & Psychotherapy Journal*. 2010/10//. 10:13	Level 1, Form Title/abstract screen
3252	**Mark Holmes, Hugo Jones**. Using online tools to treat alcohol misuse.. *Nursing Times*. 2016/04/27/. 112:12	Level 1, Form Title/abstract screen
3254	**Anita Slomski**. Primary Care Treatment of Substance Use Disorder Reaches More Patients.. *JAMA: Journal of the American Medical Association*. 2017/11/28/. 318:1968	Level 1, Form Title/abstract screen
3255	**Karen Jordan**. Integrating wellbeing services in healthcare pathways.. *Primary Health Care*. 2015/02//. 25:30	Level 1, Form Title/abstract screen
3258	**Gilbert DJ,Harvey AR,Belgrave FZ**. Advancing the Africentric paradigm shift discourse: building toward evidence-based Africentric interventions in social work practice with African Americans.. *Social Work*. 2009/07//. 54:243	Level 1, Form Title/abstract screen
3267	**Deirdre Marshall**. Twenty-four-hour sexual assault care -- incorporating courtesy, dignity, privacy and respect.. *Healthcare Counselling & Psychotherapy Journal*. 2012/01//. 12:26	Level 1, Form Title/abstract screen
3268	**Alyssa L. Rodgers**. Hope for Families Struggling with Adolescents and Addiction.. *PARADIGM (Targeted Publications Group, Inc)*. 2017/10//. 21:2	Level 1, Form Title/abstract screen
3270	**Amanda H. Teague, Amy J. Jnah, Desi Newberry**. Intraprofessional Excellence in Nursing: Collaborative Strategies for Neonatal Abstinence Syndrome.. *Neonatal Network*. 2015/11//Nov/Dec2015. 34:320	Level 1, Form Title/abstract screen
3275	**Esta Sužić, Ema Ničea Gruber, Blaženka Guberina Korotaj**. Bio-psycho-social model of treatment and rehabilitation of addicts during the conduction of safety measure of obligatory psychiatric treatment in prison hospital Zagreb.. *Alcoholism: Journal on Alcoholism & Related Addictions*. 2014/09//. 50:93	Level 1, Form Title/abstract screen
3278	**Ling S**. Nurse practitioners in drug and alcohol: where are they?. *Australian Journal of Advanced Nursing*. 2009/06//Jun-Aug2009. 26:64	Level 1, Form Title/abstract screen
3279	. UK Alcohol Treatment Trial: client-treatment matching effects.. *Addiction*. 2008/02//. 103:228	Level 2, Form Full Text Screening
3281	**Wu C,Lin C**. The application of motivational interviewing in nursing practice..	Level 1, Form Title/abstract

	Journal of Nursing. 2009/04//. 56:89	screen
3282	**Betty J. Braxter, Kathy Puskar, Ann M. Mitchell, Holly Hagle, Heather Gotham, Martha Ann Terry**. Nursing Students' Experiences With Screening, Brief Intervention, and Referral to Treatment for Substance Use in the Clinical/Hospital Setting.. *Journal of Addictions Nursing (Lippincott Williams & Wilkins)*. 2014/07//Jul-Sep2014. 25:122	Level 1, Form Title/abstract screen
3283	**Mariana Chilton, Molly Knowles, Jenny Rabinowich, Kimberly T Arnold**. The relationship between childhood adversity and food insecurity: 'It's like a bird nesting in your head'.. *Public Health Nutrition*. 2015/10//. 18:2643	Level 1, Form Title/abstract screen
3286	**Furlong M,Leddy J,Ferguson J,Heart K**. Assertive community treatment and recovery at Thresholds.. *American Journal of Psychiatric Rehabilitation*. 2009/04//Apr-Jun2009. 12:108	Level 1, Form Title/abstract screen
3287	**Thomas JR,Fraser VV**. Implementing evidence-based supported employment in a recovery-oriented mental health agency.. *American Journal of Psychiatric Rehabilitation*. 2009/04//Apr-Jun2009. 12:143	Level 1, Form Title/abstract screen
3288	**Jennifer Kawi**. Managing CHRONIC PAIN in primary care.. *Nurse Practitioner*. 2016/03//. 41:14	Level 1, Form Title/abstract screen
3289	**Andrew Day**. Commentary on Stuart et al. (2013): Domestic violence and interventions to reduce alcohol use.. *Addiction*. 2013/08//. 108:1385	Level 1, Form Title/abstract screen
3293	**Larissa Nekhlyudov, Christina Lacchetti, Nancy B. Davis, Thomas Q. Garvey, David P. Goldstein, J. Chris Nunnink, Jose I. Ruades Ninfea, Andrew L. Salner, Talya Salz, Lillian L. Siu, Jose I Ruades Ninfea**. Head and Neck Cancer Survivorship Care Guideline: American Society of Clinical Oncology Clinical Practice Guideline Endorsement of the American Cancer Society Guideline.. *Journal of Clinical Oncology*. 2017/05/10/. 35:1606	Level 1, Form Title/abstract screen
3294	**O'Connell N**. Smoking cessation management must become more individualized.. *Nurse Prescribing*. 2009/11//. 7:486	Level 1, Form Title/abstract screen
3298	**Stacy L. Haber, Virginia Boomershine, Erin Raney**. Safety of Varenicline in Patients With Cardiovascular Disease.. *Journal of Pharmacy Practice*. 2014/02//. 27:65	Level 1, Form Title/abstract screen
3299	**Ursula Kelly, Mary Ann Boyd, Sharon M. Valente, Elizabeth Czekanski**. Trauma-Informed Care: Keeping Mental Health Settings Safe for Veterans.. *Issues in Mental Health Nursing*. 2014/06//. 35:413	Level 1, Form Title/abstract screen
3300	**Daniel Z. Lieberman, Anne Cioletti, Suena H. Massey, Rochelle S. Collantes, Brad B. Moore**. TREATMENT PREFERENCES AMONG PROBLEM DRINKERS IN PRIMARY CARE.. *International Journal of Psychiatry in Medicine*. 2014/04//. 47:231	Level 1, Form Title/abstract screen
3301	**Laura C. Feemster, J. Randall Curtis**. "We Understand the Prognosis, but We Live with Our Heads in the Clouds": Understanding Patient and Family Outcome Expectations and Their Influence on Shared Decision Making.. *American Journal of Respiratory & Critical Care Medicine*. 2016/02//2/1/2016. 193:239	Level 1, Form Title/abstract screen
3302	**Pierre Leichner, Carissa Wieler**. maladjusted : Participatory theatre about human-centred care.. *Arts & Health: International Journal for Research, Policy & Practice*. 2015/02//. 7:75	Level 1, Form Title/abstract screen
3304	**Benjamin Doolittle, William Becker**. A Case Series of	Level 1, Form

	Buprenorphine/Naloxone Treatment in a Primary Care Practice.. *Substance Abuse*. 2011/10//Oct-Dec2011. 32:262	Title/abstract screen
3306	**Weisner C,Parthasarathy S,Moore C,Mertens JR**. Individuals receiving addiction treatment: are medical costs of their family members reduced?. *Addiction*. 2010/07//. 105:1226	Level 1, Form Title/abstract screen
3310	**Jomella Watson-Thompson, Nikki Keene Woods, Daniel J. Schober, Jerry A. Schultz**. Enhancing the Capacity of Substance Abuse Prevention Coalitions Through Training and Technical Assistance.. *Journal of Prevention & Intervention in the Community*. 2013/07//Jul-Sep2013. 41:176	Level 1, Form Title/abstract screen
3316	**Cory R. Cummings, Kia J. Bentley**. Contemporary Health-Related Decision Aids: Tools for Social Work Practice.. *Social Work in Health Care*. 2014/09//. 53:762	Level 1, Form Title/abstract screen
3318	**Joan Trujols, Maria J. Portella, Ioseba Iraurgi, M. Josefa Campins, Núria Siñol, José Pérez de Los Cobos**. Patient-reported outcome measures: Are they patient-generated, patient-centred or patient-valued?. *Journal of Mental Health*. 2013/12//. 22:555	Level 1, Form Title/abstract screen
3320	**Mitch Winemaker, Danielle Petruccelli, Conrad Kabali, Justin de Beer**. Not all total joint replacement patients are created equal: preoperative factors and length of stay in hospital.. *Canadian Journal of Surgery*. 2015/06//Jun/jun2015. 58:160	Level 1, Form Title/abstract screen
3322	**Thomas SA,Hargett T**. Mental health care: a collaborative, holistic approach.. *Holistic Nursing Practice*. 1999/01//. 13:78	Level 1, Form Title/abstract screen
3323	**Giovazolias T,Davis P**. Matching therapeutic interventions to drug and alcohol abusers' stage of motivation: the clients' perspective.. *Counselling Psychology Quarterly*. 2005/09//. 18:171	Level 1, Form Title/abstract screen
3325	**G. Dom, M. Wojnar, C.L. Crunelle, N. Thon, J. Bobes, U.W. Preuss, G. Addolorato, H.K. Seitz, F.M. Wurst**. Assessing and Treating Alcohol Relapse Risk in Liver Transplantation Candidates.. *Alcohol & Alcoholism*. 2015/03//. 50:164	Level 1, Form Title/abstract screen
3327	**Joseph M. Cerimele, Anna Ratzliff, Jennifer M. Sexton**. Collaborative care for a patient with bipolar disorder in primary care: a case example.. *General Hospital Psychiatry*. 2015/03//Mar/Apr2015. 37:144	Level 1, Form Title/abstract screen
3328	**Paolo Del Vecchio**. Bringing Recovery to Practice: Improving Provider Competencies and Promoting Positive Outcomes.. *Psychiatric Services*. 2015/07//. 66:750	Level 1, Form Title/abstract screen
3329	**Nicole P. Yuan, Heide Castañeda, Mark Nichter, Mimi Nichter, Steven Wind, Lauren Carruth, Myra Muramoto**. Lay Health Influencers: How They Tailor Brief Tobacco Cessation Interventions.. *Health Education & Behavior*. 2012/10//. 39:544	Level 2, Form Full Text Screening
3331	**Sadie Geraghty**. Reaching out: caring for women prisoners in Western Australia.. *Practising Midwife*. 2015/01//. 18:26	Level 1, Form Title/abstract screen
3332	**Moyer A,Finney JW**. Brief interventions for alcohol problems: factors that facilitate implementation.. *Alcohol Research & Health*. 2004/03//2004/2005. 28:44	Level 1, Form Title/abstract screen
3334	**Amanda Conley**. Strengths-Based Nursing Care.. *International Journal for Human Caring*. 2014/07//. 18:67	Level 1, Form Title/abstract screen

3335	**Pamela J. Black, Michael Woodworth, Moreen Tremblay, Tara Carpenter**. A Review of Trauma-Informed Treatment for Adolescents.. *Canadian Psychology*. 2012/08//. 53:192	Level 1, Form Title/abstract screen
3336	**Jennifer B Green**. understanding the type 2 diabetes mellitus and cardiovascular disease risk paradox.. *Postgraduate Medicine*. 2014/05//2014 May. 126:190	Level 1, Form Title/abstract screen
3338	**Aysha Mendes**. COPD in the community: working beyond boundaries.. *British Journal of Community Nursing*. 2014/10//. 19:514	Level 1, Form Title/abstract screen
3340	**Karen Y. Frantz**. Care, Collaboration, Community for Maternal Opiate Addiction.. *JOGNN: Journal of Obstetric, Gynecologic & Neonatal Nursing*. 2016/06//Jun2016 Supplement. 45:S6	Level 2, Form Full Text Screening
3341	**S. McIlfatrick, S. Keeney, H. McKenna, N. McCarley, G. McIlwee**. Exploring the actual and potential role of the primary care nurse in the prevention of cancer: a mixed methods study.. *European Journal of Cancer Care*. 2014/05//. 23:288	Level 1, Form Title/abstract screen
3342	**Malcolm W Battersby, Jill Beattie, Rene G Pols, David P Smith, John Condon, Sarah Blunden**. A randomised controlled trial of the Flinders Program™ of chronic condition management in Vietnam veterans with co-morbid alcohol misuse, and psychiatric and medical conditions.. *Australian & New Zealand Journal of Psychiatry*. 2013/05//. 47:451	Level 1, Form Title/abstract screen
3344	**Daniela Bundalo-Vrbanac, Danijel Buljan, Vjekoslav Peitl, Josipa Gelo**. Integrating psychotherapy and pharmacotherapy in treatment of substance dependence.. *Alcoholism: Journal on Alcoholism & Related Addictions*. 2012/09//. 48:107	Level 1, Form Title/abstract screen
3345	**Neville Greaves**. The smoking cessation clinic.. *Journal of the Acupuncture Association of Chartered Physiotherapists*. 2014/09//. 30:105	Level 1, Form Title/abstract screen
3346	**Masterson A**. Community matrons: the value of knowing self (part two). *Nursing Older People*. 2007/06//. 19:29	Level 1, Form Title/abstract screen
3347	. Chapter 9: the prenatal assessment.. *Maternal-Child Nursing Care: Optimizing Outcomes for Mothers, Children & Families*. 2009/01//. #volume#:215	Level 1, Form Title/abstract screen
3348	**Flannery Fielding, Tanya M. Sanford, Mellar P. Davis**. Achieving effective control in cancer pain: a review of current guidelines.. *International Journal of Palliative Nursing*. 2013/12//. 19:584	Level 1, Form Title/abstract screen
3349	**Sandeep Grover, Naresh Nebhinani, Subho Chakrabarti, Ruchita Shah, Ajit Avasthi**. Relationship between First Treatment Contact and Supernatural Beliefs in Caregivers of Patients with Schizophrenia.. *East Asian Archives of Psychiatry*. 2014/06//. 24:58	Level 1, Form Title/abstract screen
3350	. Treatment programs appear eager to leave no possible client behind...[corrected][published erratum appears in ALCOHOL DRUG ABUSE WKLY 2013 Jul 22; 25(28): 7]. *Alcoholism & Drug Abuse Weekly*. 2013/07/15/. 25:1	Level 1, Form Title/abstract screen
3352	**Mendez D,Jacobson PD,Hassmiller KM,Zellman GL**. The effect of legal and hospital policies on physician response to prenatal substance exposure.. *Maternal & Child Health Journal*. 2003/09//. 7:187	Level 1, Form Title/abstract screen
3353	**Decker S,Cary P,Krautscheid L**. From the streets to assisted living:	Level 1, Form

	perceptions of a vulnerable population.. *Journal of Psychosocial Nursing & Mental Health Services*. 2006/06//. 44:18	Title/abstract screen
3354	**Marcus MT,Walker T,Swint JM,Smith BP,Brown C,Busen N,Edwards T,Liehr P,Taylor WC,Williams D,Von Sternberg K**. Community-based participatory research to prevent substance abuse and HIV/AIDS in African-American adolescents.. *Journal of Interprofessional Care*. 2004/11//. 18:347	Level 1, Form Title/abstract screen
3355	**Anderson JE,Larke SC**. The Sooke Navigator project: using community resources and research to improve local service for mental health and addictions.. *Mental Health in Family Medicine*. 2009/03//. 6:21	Level 1, Form Title/abstract screen
3356	**Anderson JE,Larke SC**. Navigating the mental health and addictions maze: a community-based pilot project of a new role in primary mental health care.. *Mental Health in Family Medicine*. 2009/03//. 6:15	Level 1, Form Title/abstract screen
3360	**Evan Senreich**. Lesbian, Gay, and Bisexual Clients in a Substance Abuse Treatment Program Serving a Mostly Black and Hispanic Population.. *Journal of LGBT Issues in Counseling*. 2012/10//Oct-Dec2012. 6:310	Level 1, Form Title/abstract screen
3363	**Linda S. Beeber, Madeline A. Naegle, Geraldine S. Pearson, Rebecca E. Salomon**. Health Needs of Persons With Mental Disorders.. *Journal of the American Psychiatric Nurses Association*. 2016/07//Jul/Aug2016. 22:287	Level 1, Form Title/abstract screen
3364	**Cecilia Pompili**. Quality of life after lung resection for lung cancer.. *Journal of Thoracic Disease*. 2015/04//2015 Supplement 2. 7:S138	Level 1, Form Title/abstract screen
3365	**Ryan Kemp**. The symbolic constitution of addiction: Language, alienation, ambivalence.. *Health: An Interdisciplinary Journal for the Social Study of Health, Illness & Medicine*. 2012/07/15/. 16:434	Level 1, Form Title/abstract screen
3368	. UPFRONT: Roundup. .4M for research to combat drug abuse.. *Canadian Chiropractor*. 2016/04//. 21:8	Level 1, Form Title/abstract screen
3370	**Marianna Fontana, Perviz Asaria, Michela Moraldo, Judith Finegold, Khalil Hassanally, Charlotte H Manisty, Darrel P Francis**. Patient-accessible tool for shared decision making in cardiovascular primary prevention: balancing longevity benefits against medication disutility.. *Circulation*. 2014/06/17/. 129:2539	Level 1, Form Title/abstract screen
3371	**Michael W Drazer, Sandip M Prasad, Dezheng Huo, Mara A Schonberg, William Dale, Russell Z Szmulewitz, Scott E Eggener**. National trends in prostate cancer screening among older American men with limited 9-year life expectancies: Evidence of an increased need for shared decision making.. *Cancer (0008543X)*. 2014/05/15/. 120:1491	Level 1, Form Title/abstract screen
3375	**Sarah McMahon, Rachel Schwartz**. A Review of Rape in the Social Work Literature: A Call to Action.. *Affilia: Journal of Women & Social Work*. 2011/08//. 26:250	Level 1, Form Title/abstract screen
3376	**Shirley A. Murphy**. Interdisciplinary Education in the Addictions.. *Journal of Addictions Nursing (Lippincott Williams & Wilkins)*. 2013/03//Jan-Mar2013. 24:4	Level 1, Form Title/abstract screen
3377	**Robin P Newhouse, Cheryl Dennison Himmelfarb, Laura Morlock, Kevin D Frick, Peter Pronovost, Yulan Liang**. A Phased Cluster-randomized Trial of Rural Hospitals Testing a Quality Collaborative to Improve Heart Failure Care: Organizational Context Matters.. *Medical Care*. 2013/05//2013 May. 51:396	Level 1, Form Title/abstract screen
3378	**Margrethe Smidth**. Integrated Care for people who want Alcohol	Level 1, Form

	Detoxification in the middle of Denmark.. *International Journal of Integrated Care (IJIC)*. 2016/12/02/2016 Supplement. 16:1	Title/abstract screen
3379	**Varela TA,Montbach J,Shipe S**. Psychoactive medication adherence in substance users living with HIV/AIDS.. *Journal of Addictions Nursing (Taylor & Francis Ltd)*. 2007/03//. 18:5	Level 1, Form Title/abstract screen
3380	**Wei-Chen Tung, Maureen Barnes**. Heart Diseases Among Native Hawaiians and Pacific Islanders.. *Home Health Care Management & Practice*. 2014/05//. 26:110	Level 1, Form Title/abstract screen
3381	**Gabriela Novotná**. Competing institutional logics in the development and implementation of integrated treatment for concurrent disorders in Ontario: A case study.. *Journal of Social Work*. 2014/05//. 14:260	Level 2, Form Full Text Screening
3382	**Malaika Mutere, Adeline Nyamathi, Ashley Christiani, Jeff Sweat, Glenna Avila, Leo Hobaica**. Homeless Youth Seeking Health and Life-Meaning Through Popular Culture and the Arts.. *Child & Youth Services*. 2014/07//Jul-Sep2014. 35:273	Level 1, Form Title/abstract screen
3383	**Cynthia Reade, Rosemary Nourse**. RESEARCH CORNER. Intervening to prevent violence in psychiatric units.. *Nursing*. 2012/07//. 42:14	Level 1, Form Title/abstract screen
3385	**Ann B. Smith, Michelle Odlum, Manik Sikka, Suzanne Bakken, Tim Kanter**. Patient Perceptions of Pre-Implementation of Personal Health Records (PHRs): A Qualitative Study of People Living With HIV in New York City.. *Journal of HIV/AIDS & Social Services*. 2012/10//Oct-Dec2012. 11:406	Level 1, Form Title/abstract screen
3387	**Deborah Bailey, Samir Pathak, Niaz Ahmad**. Is liver transplant for alcohol-related end-stage liver disease appropriate?. *British Journal of Hospital Medicine (17508460)*. 2013/08//. 74:439	Level 1, Form Title/abstract screen
3388	**Hilary Connery, Shelly Greenfield, Viktoriya Livchits, Lana McGrady, Nickolette Patrick, Charmaine S. Lastimoso, Jessica H. Heney, Adrianne Katrina Nelson, Alan Shields, Yekaterina P. Stepanova, Lidia Y. Petrova, Oleg V. Anastasov, Olga I. Novoseltseva, Sonya S. Shin**. Training and Fidelity Monitoring of Alcohol Treatment Interventions Integrated Into Routine Tuberculosis Care in Tomsk, Russia: the IMPACT Effectiveness Trial.. *Substance Use & Misuse*. 2013/07//. 48:806	Level 2, Form Full Text Screening
3391	**Akhavain P,Amaral D,Murphy M,Uehlinger KC**. Collaborative practice: a nursing perspective of the psychiatric interdisciplinary treatment team.. *Holistic Nursing Practice*. 1999/01//. 13:1	Level 1, Form Title/abstract screen
3392	**Michelle Morris, Carmel Seibold, Ruth Webber**. Drugs and having babies: An exploration of how a specialist clinic meets the needs of chemically dependent pregnant women.. *Midwifery*. 2012/04//. 28:163	Level 2, Form Full Text Screening
3395	. Best treatments for young people with heroin addiction: No rule book.. *Alcoholism & Drug Abuse Weekly*. 2013/04/29/. 25:1	Level 1, Form Title/abstract screen
3397	**William J. Doherty, Susan H. McDaniel, Jeri Hepworth**. Contributions of Medical Family Therapy to the Changing Health Care System.. *Family Process*. 2014/09//. 53:529	Level 1, Form Title/abstract screen
3401	**Hussein A Tahan, Patrice V Schminkey**. Motivational Interviewing: Building Rapport With Clients to Encourage Desirable Behavioral and Lifestyle Changes...ceu ex pg 173-4. *Professional Case Management*. 2012/07//2012 Jul-Aug. 17:164	Level 1, Form Title/abstract screen
3403	**Dennis KW,Lourie IS**. Everything is normal until proven otherwise..	Level 1, Form

	Children's Voice. 2006/11//Nov/Dec2006. 15:38	Title/abstract screen
3404	. Substance use must be included in electronic patient records: Study.. *Alcoholism & Drug Abuse Weekly*. 2012/01/23/. 24:1	Level 1, Form Title/abstract screen
3405	**Scrandis DA,Fauchald SK,Radsma J**. Global gendercide: interwoven threats to women's health and nursing obligations.. *Journal of Multicultural Nursing & Health (JMCNH)*. 2004///Winter2004. 10:7	Level 1, Form Title/abstract screen
3406	**VanderSchie-Bezyak JL**. Service problems and solutions for individuals with mental retardation and mental illness.. *Journal of Rehabilitation*. 2003/01//Jan-Mar2003. 69:53	Level 1, Form Title/abstract screen
3407	**Marcus MT,Gerace LM,Sullivan EJ**. Enhancing nursing competence with substance abusing clients.. *Journal of Nursing Education*. 1996/11//. 35:361	Level 1, Form Title/abstract screen
3409	**Rassool GH**. Prescription for change: perspectives on prescribing authority for addiction nurses in the United Kingdom.. *Journal of Addictions Nursing (Taylor & Francis Ltd)*. 2004/12//. 15:193	Level 1, Form Title/abstract screen
3410	**Sara Faithfull, Cathy Burton, Sinead Clarke, Mike Kirby, Alex Lyon, Gill Levitt, Karen Poole, Fiona Walter**. Mitigating risk of cardiovascular disease in people living with and beyond cancer.. *Cancer Nursing Practice*. 2017/02//. 16:18	Level 1, Form Title/abstract screen
3414	**Harold Alan Pincus, Brigitta Spaeth-Rublee, Katherine E. Watkins**. The Case For Measuring Quality In Mental Health And Substance Abuse Care.. *Health Affairs*. 2011/04//. 30:730	Level 1, Form Title/abstract screen
3415	**Luann Richardson**. Motivational Interviewing: Helping Patients Move Toward Change.. *Journal of Christian Nursing*. 2012/01//Jan-Mar2012. 29:18	Level 1, Form Title/abstract screen
3418	**Woods ER,Samples CL,Melchiono MW,Keenan PM,Fox DJ,Harris SK**. Initiation of services in the Boston HAPPENS Program: human immunodeficiency virus-positive, homeless, and at-risk youth can access services.. *AIDS Patient Care & STDs*. 2002/10//. 16:497	Level 1, Form Title/abstract screen
3420	**Anika Kristin Boyd**. A Nurse-Led Intervention to Improve Chronic Non-Cancer Pain.. *Nurse-Led Intervention to Improve Chronic Non-Cancer Pain*. 2017/01//. #volume#:1	Level 1, Form Title/abstract screen
3423	**Vivek Shetty, Gran N Marshall**. Collaborative Care of the Facial Injury Patient.. *Perioperative Nursing Clinics*. 2011/12//2011 Dec. 6:xi	Level 1, Form Title/abstract screen
3424	**Lisa Goldsmith**. Counselling smokers.. *Pharmacy News*. 2012/07//. #volume#:39	Level 1, Form Title/abstract screen
3427	**Jones A**. Clinical. Causes and effects of chronic obstructive pulmonary disease.. *British Journal of Nursing*. 2001/07/12/. 10:845	Level 1, Form Title/abstract screen
3428	**Scott DeMuro, Kenneth Wanberg, Rachel Anderson**. Driving While Impaired (DWI) Intervention Service Provider Orientations: The Scales of the DWI Therapeutic Educator Inventory (DTEI). *Substance Abuse*. 2011/10//Oct-Dec2011. 32:225	Level 1, Form Title/abstract screen
3431	**Jennifer DiPiazza**. The Lived Experience of Maintaining Cigarette Smoking Cessation for a Year or More: Implications for Nursing Practice...28th Annual	Level 1, Form Title/abstract

	Scientific Session, June 2-6, 2017, Baltimore, Maryland. *Nursing Research*. 2016/03//Mar/Apr2016. 65:E6	screen
3432	**Niven JA**. Client-centered, culture-friendly behavioral health care techniques for work with Alaska natives in the bering strait region.. *Social Work in Mental Health*. 2010/07//Jul/Aug2010. 8:398	Level 2, Form Full Text Screening
3435	**Bonner JE,Barritt AS 4th,Fried MW,Evon DM, Jason E Bonner, A Sidney 4th Barritt, Michael W Fried, Donna M Evon**. Time to rethink antiviral treatment for hepatitis C in patients with coexisting mental health/substance abuse issues.. *Digestive Diseases & Sciences*. 2012/06//. 57:1469	Level 1, Form Title/abstract screen
3437	. Research Briefs.. *AHRQ Research Activities*. 2012/10//. #volume#:29	Level 1, Form Title/abstract screen
3441	**George M, Maureen George**. Health beliefs, treatment preferences and complementary and alternative medicine for asthma, smoking and lung cancer self-management in diverse Black communities.. *Patient Education & Counseling*. 2012/12//. 89:489	Level 1, Form Title/abstract screen
3445	**Allison R. Gilbert, Joseph P. Morrissey, Marisa E. Domino**. Service Utilization Patterns as Predictors of Response to Trauma-Informed Integrated Treatment for Women With Co-occurring Disorders.. *Journal of Dual Diagnosis*. 2011/07//Jul-Sep2011. 7:117	Level 1, Form Title/abstract screen
3448	**Alison Evans Cuellar, Jehanzeb Cheema**. As Roughly 700,000 Prisoners Are Released Annually, About Half Will Gain Health Coverage And Care Under Federal Laws.. *Health Affairs*. 2012/05//. 31:931	Level 1, Form Title/abstract screen
3449	**Karen Davis-Brown, Naeemah Carter, Bethany D Miller**. Youth advisors driving action: hearing the youth voice in mental health systems of care...CE exam 44-5. *Journal of Psychosocial Nursing & Mental Health Services*. 2012/03//. 50:39	Level 1, Form Title/abstract screen
3452	**Matheson GO,Klügl M,Dvorak J,Engebretsen L,Meeuwisse WH,Schwellnus M,Blair SN,van Mechelen W,Derman W,Börjesson M,Bendiksen F,Weiler R**. Responsibility of sport and exercise medicine in preventing and managing chronic disease: applying our knowledge and skill is overdue.. *British Journal of Sports Medicine*. 2011/12/15/. 45:1272	Level 1, Form Title/abstract screen
3455	**Ford C,Oliver J,Whitehead B**. Treating drug users: a collaborative method.. *Therapy Today*. 2006/03//. 17:17	Level 1, Form Title/abstract screen
3456	**Ulla Timlin, Kaisa Riala, Helvi Kyngäs**. Adherence to treatment among adolescents in a psychiatric ward.. *Journal of Clinical Nursing*. 2013/05//. 22:1332	Level 1, Form Title/abstract screen
3457	**Frances J. Kay-Lambkin, Amanda L. Baker, Brian J. Kelly, Terry J. Lewin**. It's Worth a Try: The Treatment Experiences of Rural and Urban Participants in a Randomized Controlled Trial of Computerized Psychological Treatment for Comorbid Depression and Alcohol/Other Drug Use.. *Journal of Dual Diagnosis*. 2012/11//Oct-Dec2012. 8:262	Level 1, Form Title/abstract screen
3459	**Karen-leigh Edward, Rhonda Nelson Hearity, Boycer Felstead**. Service integration for the dually diagnosed.. *Australian Journal of Primary Health*. 2012/03//. 18:17	Level 1, Form Title/abstract screen
3462	**Center BH**. Grappling with a hard case: assessment and case management of the substance abuse patient.. *Case in Point*. 2009/08//2009 Aug-Sep. 7:44	Level 1, Form Title/abstract screen

3463	**Croghan E,Johnson C**. Supporting smoking cessation and dietary change.. *Nursing Standard*. 2005/04/27/. 19:52	Level 1, Form Title/abstract screen
3465	**Knight DK,Edwards JR,Flynn PM**. Predictors of change in the provision of services within outpatient substance abuse treatment programs.. *Journal of Public Health Management & Practice*. 2010/11//2010 Nov-Dec. 16:553	Level 1, Form Title/abstract screen
3467	**Amy Endee**. Characterizing a new mindset for substance use professionals.. *Addiction Professional*. 2014/11//Nov/Dec2014. 12:8	Level 1, Form Title/abstract screen
3469	**LEWIS M,ALLEN H,WARR J**. The development and implementation of a nurse-led hepatitis C protocol for people with serious mental health problems.. *Journal of Psychiatric & Mental Health Nursing*. 2010/09//. 17:651	Level 1, Form Title/abstract screen
3470	. New Jersey nonprofit emphasizes expansion of its capabilities.. *Alcoholism & Drug Abuse Weekly*. 2012/04/02/. 24:1	Level 1, Form Title/abstract screen
3471	. Center blends improvement efforts to sharpen methadone, detox care.. *Alcoholism & Drug Abuse Weekly*. 2012/01/23/. 24:1	Level 1, Form Title/abstract screen
3472	**Duran B,Harrison M,Shurley M,Foley K,Morris P,Davidson-Stroh L,Iralu J,Jiang Y,Andrasik MP**. Tribally-driven HIV/AIDS health services partnerships: evidence-based meets culture-centered interventions.. *Journal of HIV/AIDS & Social Services*. 2010/04//Apr-Jun2010. 9:110	Level 1, Form Title/abstract screen
3473	. Answer/evaluation form: Nursing care for the patient with co-existing pain and substance misuse: meeting the patient's needs.. *MEDSURG Nursing*. 2010/01//Jan/Feb2010. 19:31	Level 1, Form Title/abstract screen
3474	**Balachandra K,Petrakis I**. Setting up a buprenorphine clinic: one year later.. *Addictive Disorders & Their Treatment*. 2005/09//. 4:111	Level 1, Form Title/abstract screen
3475	**Peter J Mazzone, Amanda Tenenbaum, Meredith Seeley, Hilary Petersen, Christina Lyon, Xiaozhen Han, Xiao-Feng Wang**. Impact of a Lung Cancer Screening Counseling and Shared Decision-Making Visit.. *CHEST*. 2016/11//. 150:N.PAG	Level 1, Form Title/abstract screen
3478	**Connie Miyao**. EXPANDING THE REACH OF TOBACCO CESSATION STRATEGIES.. *Oregon Nurse*. 2013/02//. #volume#:13	Level 1, Form Title/abstract screen
3480	**Jane Meschan Foy**. The Medical Home and Integrated Behavioral Health.. *Pediatrics*. 2015/05//. 135:930	Level 1, Form Title/abstract screen
3482	**Rossiter R,Black J**. Challenging therapeutic pessimism: borderline personality disorder and co-morbid substance abuse.. *Mental Health & Substance Use: Dual Diagnosis*. 2009/06//. 2:140	Level 1, Form Title/abstract screen
3483	**Nancy Murphy**. Advancing the Interdisciplinary Collaborative Health Team Model: Professionalism, Implementation Science, and Therapeutic Alliance to Enact Social Justice Practice.. *Advances in Nursing Science*. 2015/07//Jul-Sep2015. 38:215	Level 1, Form Title/abstract screen
3484	**Lantz MS**. Helping an older man quit smoking.. *Clinical Geriatrics*. 2010/07//2010 Jul-Aug. 18:32	Level 1, Form Title/abstract screen

3485	. Study urges anti-burnout aid for intensive care nurses.. *Nursing Standard*. 2016/07/20/. 30:10	Level 1, Form Title/abstract screen
3486	**Andis Robeznieks**. Aiming to provide concierge care 'for the masses'...Feinberg, David. *Modern Healthcare*. 2015/09/14/. 45:0030	Level 1, Form Title/abstract screen
3490	**Susan Kreitz, Mischelle Knipe**. An Ethical Perspective of Pain Management.. *Minnesota Nursing Accent*. 2013///Winter2013. 85:19	Level 1, Form Title/abstract screen
3491	**Kathleen Sciacca**. A language for integrated care.. *Behavioral Healthcare*. 2012/11//Nov/Dec2012. 32:24	Level 1, Form Title/abstract screen
3496	**Mercer SO**. Navajo elderly people in a reservation nursing home: admission predictors and culture care practices.. *Social Work*. 1996/03//. 41:181	Level 1, Form Title/abstract screen
3497	**Roes NA**. No single path to addressing trauma.. *Addiction Professional*. 2010/11//Nov/Dec2010. 8:30	Level 1, Form Title/abstract screen
3499	**David Oslin**. Personalized Addiction Treatment: How Close Are We?. *Alcohol & Alcoholism*. 2011/05//May/Jun2011. 46:231	Level 1, Form Title/abstract screen
3501	**Shannon Bourke**. Drinking In Nevada: the Nursing Solution.. *Nevada RNformation*. 2012/08//. 21:12	Level 1, Form Title/abstract screen
3503	**Malcolm Battersby, Michael Von Korff, Judith Schaefer, Connie Davis, Evette Ludman, Sarah M. Greene, Melissa Parkerton, Edward H. Wagner**. Twelve evidence-based principles for implementing self-management support in primary care.. *Joint Commission Journal on Quality & Patient Safety*. 2010/12//2010 Dec. 36:561	Level 1, Form Title/abstract screen
3504	**Delacroix J,Brown J,Kadenhe-Chiweshe A,Bodenstein L,Stimell-Rauch M,Lowe T**. Rectal perforation secondary to rape and fisting in a female adolescent.. *Pediatric Emergency Care*. 2011/02//2011 Feb. 27:116	Level 1, Form Title/abstract screen
3505	. Minnesota Nurses Advocate for a Monitoring Bill that Supports Nurses and Patient Safety.. *Minnesota Nursing Accent*. 2014///Spring2014. 86:12	Level 1, Form Title/abstract screen
3506	**Alakeson V,Frank RG,Katz RE**. Specialty care medical homes for people with severe, persistent mental disorders.. *Health Affairs*. 2010/05//. 29:867	Level 1, Form Title/abstract screen
3509	**R. Gary Sibbald, Laurie Goodman, Kevin Y. Woo, Diane L. Krasner, Hiske Smart, Gulnaz Tariq, Elizabeth A. Ayello, Robert E. Burrell, David H. Keast, Dieter Mayer, Linda Norton, Richard 'Sal' Salcido**. Special considerations in wound bed preparation 2011: an update.. *World Council of Enterostomal Therapists Journal*. 2012/04//Apr-Jun2012. 32:10	Level 1, Form Title/abstract screen
3510	**Adam Simning, Yeates Conwell, Susan G Fisher, Thomas M Richardson, Edwin van Wijngaarden**. The characteristics of anxiety and depression symptom severity in older adults living in public housing.. *International Psychogeriatrics*. 2012/04/22/. 24:614	Level 1, Form Title/abstract screen
3513	**Michelle Cleary, Catherine Hungerford**. Trauma-informed Care and the Research Literature: How Can the Mental Health Nurse Take the Lead to	Level 1, Form Title/abstract

	Support Women Who Have Survived Sexual Assault?. *Issues in Mental Health Nursing*. 2015/05//. 36:370	screen
3514	**Haug NA,Sorensen JL,Gruber VA,Lollo N,Roth G**. HAART adherence strategies for methadone clients who are HIV-positive: a treatment manual for implementing contingency management and medication coaching.. *Behavior Modification*. 2006/11//. 30:752	Level 1, Form Title/abstract screen
3517	**William R. Miller**. Sacred Cows and Greener Pastures: Reflections from 40 Years in Addiction Research.. *Alcoholism Treatment Quarterly*. 2016/01//Jan-Mar2016. 34:92	Level 2, Form Full Text Screening
3519	**Leslie O'Neill**. Comprehensive Risk Counseling Services (CRCS) at HIV specialty clinics in New Hampshire.. *Health Education Monograph Series*. 2011/09//. 28:64	Level 1, Form Title/abstract screen
3520	**Taylor-Young P,Hildebrandt E**. The multidimensional burden of hepatitis C and its treatment: a case study.. *Gastroenterology Nursing*. 2009/05//2009 May-Jun. 32:180	Level 1, Form Title/abstract screen
3522	**Hellard ME,Nguyen OK,Guy RJ,Jardine D,Mijch A,Higgs PG**. The prevalence and risk behaviours associated with the transmission of blood-borne viruses among ethnic-Vietnamese injecting drug users.. *Australian & New Zealand Journal of Public Health*. 2006/12//. 30:519	Level 1, Form Title/abstract screen
3528	**Gary A. Enos**. Multiple disabilities, multiple strategies.. *Addiction Professional*. 2012/01//Jan/Feb2012. 10:29	Level 1, Form Title/abstract screen
3529	**Chua HF,Polk T,Welsh R,Liberzon I,Strecher V**. Annual review of cybertherapy and telemedicine 2009. Neural responses to elements of a Web-based smoking cessation program.. *Studies in Health Technology & Informatics*. 2009/03//. 144:174	Level 1, Form Title/abstract screen
3531	**Schatzberg AF,Weiss RD,Brady KT,Culpepper L**. Bridging the clinical gap: managing patients with co-occurring mood, anxiety, and alcohol use disorders.. *Primary Psychiatry*. 2008/04/02/April 2008 Expert Review. 15:1	Level 1, Form Title/abstract screen
3532	**Rash EM**. Clinicians' perspectives on motivational interviewing-based brief interventions in college health.. *Journal of American College Health*. 2008/11//Nov/Dec2008. 57:379	Level 1, Form Title/abstract screen
3535	**Lacey Lacey**. Interventions in alcohol use.. *Independent Nurse*. 2012/05/07/. #volume#:1	Level 1, Form Title/abstract screen
3537	. Noticeboard.. *Nursing Older People*. 2015/11//. 27:12	Level 1, Form Title/abstract screen
3538	**Andy McLachlan, Andrew Kerr, Mildred Lee, Nicola Dalbeth**. Nurse-led cardiovascular disease risk management intervention for patients with gout.. *European Journal of Cardiovascular Nursing*. 2011/06//. 10:94	Level 1, Form Title/abstract screen
3540	**Mark Sanders**. A Recovery Revolution: Integrating the Addictions and Mental Health Recovery Movements.. *Addiction Professional*. 2011/09//Sep/Oct2011. 9:N3	Level 1, Form Title/abstract screen
3543	**Finney JW**. Commentary. Matching clients to psychosocial treatments: at the cusp of hope and evidence?. *Addiction*. 2008/02//. 103:239	Level 1, Form Title/abstract screen
3544	**Mitchell AM,Dewey CM**. Chronic pain in patients with substance abuse disorder: general guidelines and an approach to treatment.. *Postgraduate*	Level 1, Form Title/abstract

	Medicine. 2008/04//2008 Apr. 120:75	screen
3548	**Kamholz BW,Gulliver SB,Morissette SB**. Revisiting the bioinformational model of tobacco dependence: specific application to psychiatric populations.. *Journal of Dual Diagnosis*. 2006/09//. 2:19	Level 1, Form Title/abstract screen
3550	**Allen S,Beech AR**. Exploring factors that influence nurses: judgements of violence risk in a female forensic population.. *British Journal of Forensic Practice*. 2010/02//. 12:4	Level 1, Form Title/abstract screen
3552	**Bonnie Bristow, Lisa Di Prospero, Elaine Curle, Leslie Gibson, Arlene Court, Andrea Eisen, Marg Fitch**. Perceptions of Receptivity: Exploring Tobacco Use and Smoking Cessation Best Practices from the Perspectives of Individuals with Lung Cancer and Health Care Professionals: Findings from Phase I.. *Journal of Medical Imaging & Radiation Sciences*. 2014/06//. 45:168	Level 1, Form Title/abstract screen
3553	**Capoccia VA,Cotter F,Gustafson DH,Cassidy EF,Ford JH II,Madden L,Owens BH,Farnum SO,McCarty D,Molfenter T**. Making 'stone soup': improvements in clinic access and retention in addiction treatment.. *Joint Commission Journal on Quality & Patient Safety*. 2007/02//2007 Feb. 33:95	Level 1, Form Title/abstract screen
3558	**Sarah H Serling**. ICD-10-CM Coding and the Medical Home: A Focus on Medical Necessity.. *Case in Point*. 2013/07//2013 Jul. 11:21	Level 1, Form Title/abstract screen
3563	**Knudsen HK,Abraham AJ,Johnson JA,Roman PM, Hannah K Knudsen, Amanda J Abraham, J Aaron Johnson, Paul M Roman**. Buprenorphine adoption in the National Drug Abuse Treatment Clinical Trials Network.. *Journal of Substance Abuse Treatment*. 2009/10//. 37:307	Level 1, Form Title/abstract screen
3564	**Ritchie G,Weldon S,Macpherson G,Laithwaite H**. Evaluation of a drug and alcohol relapse prevention programme in a special hospital: an interpretative phenomenological analysis.. *British Journal of Forensic Practice*. 2010/08//. 12:17	Level 1, Form Title/abstract screen
3566	**Adam J. Gordon, Lauren M. Broyles**. A Physician-Centered Approach to Addiction Identification and Treatment Misses the Opportunity for Interdisciplinary Solutions.. *Substance Abuse*. 2014/04//Apr-Jun2014. 35:108	Level 1, Form Title/abstract screen
3569	**Shelly R Noe**. Office-based buprenorphine treatment: Identifying factors that promote retention in opioid dependent patients.. *Office-based Buprenorphine Treatment: Identifying Factors That Promote Retention in Opioid Dependent Patients*. 2012/01//. #volume#:41 p	Level 1, Form Title/abstract screen
3571	**Bush NJ,Wickham R**. Knowledge central.. *Oncology Nursing Forum*. 2010/03//. 37:225	Level 1, Form Title/abstract screen
3572	**D'Arcy Y**. Pain solutions. Managing chronic pain in acute care.. *Nurse Practitioner*. 2010/12//. 35:14	Level 1, Form Title/abstract screen
3573	**Husted J**. Exploring substance use's role in dementia: an abuse history could inform care for some elderly patients.. *Addiction Professional*. 2006/09//2006 Sep-Oct. 4:43	Level 1, Form Title/abstract screen
3577	**Kihlström A,Wikström E**. Towards network and citizen: Collaborative care for drug abusers.. *International Journal of Health Planning & Management*. 2009/07//. 24:233	Level 1, Form Title/abstract screen
3578	**Garrard J,Choudary V,Groom H,Dieperink E,Willenbring ML,Durfee JM,Ho SB**. Organizational change in management of hepatitis C: evaluation of a CME program.. *Journal of Continuing Education in the Health Professions*.	Level 1, Form Title/abstract screen

	2006///Spring2006. 26:145	
3579	**Thomas BS**. Postoperative pain management in a patient with a history of drug addiction.. *Pennsylvania Nurse*. 2009/06//. 64:14	Level 1, Form Title/abstract screen
3580	**Champney-Smith J,Angove R,Barrowcliff A,Sampson C,Jones C,Hughes S**. A therapeutic day programme for problem substance users in Cardiff and the Vale of Glamorgan: development and client-centred evaluation.. *Journal of Substance Use*. 2001/01/02/. 6:40	Level 1, Form Title/abstract screen
3581	**Power AK,Chawla N**. Transformations in collaborative healthcare.. *Families, Systems & Health: The Journal of Collaborative Family HealthCare*. 2008/12//. 26:459	Level 1, Form Title/abstract screen
3583	**Weiss RD,Griffin ML,Gallop R,Onken LS,Gastfriend DR,Daley D,Crits-Christoph P,Bishop S,Barber JP, R D Weiss, M L Griffin, R Gallop, L S Onken, D R Gastfriend, D Daley, P Crits-Christoph, S Bishop, J P Barber**. Self-help group attendance and participation among cocaine dependent patients.. *Drug & Alcohol Dependence*. 2000/08//. 60:169	Level 1, Form Title/abstract screen
3584	**Bremner J,Edmonds N**. Supporting people with mental health problems to quit smoking.. *A Life in the Day*. 2007/08//. 11:15	Level 1, Form Title/abstract screen
3587	**Hunter TN**. Child welfare and alcohol and other drug treatment (AOD): bridging the gap to comprehensive services.. *Journal of Family Social Work*. 2003/12//. 7:63	Level 1, Form Title/abstract screen
3588	**Bering S**. Facing the commissioning challenge: responding effectively to people whose behaviour is challenging.. *Advances in Mental Health & Intellectual Disabilities*. 2010/06//. 4:4	Level 1, Form Title/abstract screen
3589	**Lee M,George S**. Drug Strategy Unit.. *British Journal of Forensic Practice*. 2005/11//. 7:39	Level 1, Form Title/abstract screen
3590	**van der Meer IM,Ruggenenti P,Remuzzi G**. The diabetic CKD patient-a major cardiovascular challenge.. *Journal of Renal Care*. 2010/05/02/May2010 Supplement 1. 36:34	Level 1, Form Title/abstract screen
3594	**Rubin IL,Fahs JJ,Beasley JB**. Delivery of health care for people with 'dual diagnosis': from the person to the policy.. *Mental Health Aspects of Developmental Disabilities*. 2007/07//Jul-Sep2007. 10:107	Level 1, Form Title/abstract screen
3595	**Diehl-Svrjcek BC,Richardson R**. Decreasing NICU costs in the managed care arena: the positive impact of collaborative high-risk OB and NICU disease management programs.. *Lippincott's Case Management*. 2005/05//. 10:159	Level 1, Form Title/abstract screen
3598	**Dallas Bastian**. Bespoke care essential.. *Nursing Review (1326-0472)*. 2014/09//. #volume#:26	Level 1, Form Title/abstract screen
3599	**Killian Welch**. Bottled Up.. *Alcohol & Alcoholism*. 2011/03//Mar/Apr2011. 46:217	Level 1, Form Title/abstract screen
3600	**Fidelindo A. Lim, Donald V. Brown Jr.,Sung Min Justin Kim**. Addressing Health Care Disparities in the Lesbian, Gay, Bisexual, and Transgender Population: A Review of Best Practices.. *AJN American Journal of Nursing*. 2014/06//. 114:24	Level 1, Form Title/abstract screen
3605	**Swain E,Boulter S,Piek N**. Overcoming the challenges of evaluating dual diagnosis interventions in medium secure units.. *British Journal of Forensic*	Level 1, Form Title/abstract

	Practice. 2010/02//. 12:33	screen
3606	**Menihan CA**. Limited sonography in collaborative midwifery practice.. *Journal of Midwifery & Women's Health*. 2000/11//2000 Nov-Dec. 45:508	Level 1, Form Title/abstract screen
3609	**Bartels SJ,Coakley EH,Zubritsky C,Ware JH,Miles KM,Areán PA,Chen H,Oslin DW,Llorente MD,Costantino G,Quijano L,McIntyre JS,Linkins KW,Oxman TE,Maxwell J,Levkoff SE**. Improving access to geriatric mental health services: a randomized trial comparing treatment engagement with integrated versus enhanced referral care for depression, anxiety, and at-risk alcohol use.. *American Journal of Psychiatry*. 2004/08//. 161:1455	Level 1, Form Title/abstract screen
3610	**Thompson HS,Mitchell EA**. Exploring interventions in secondary stroke prevention: a case study.. *British Journal of Neuroscience Nursing*. 2006/02//2006 Feb-Mar. 2:28	Level 1, Form Title/abstract screen
3611	**Schmidt MJ,Dostal KU**. Optimizing outcomes when patients leave against medical advice.. *Journal of Clinical Outcomes Management*. 2007/12//2007 Dec. 14:645	Level 1, Form Title/abstract screen
3614	**Stoeckle-Roberts S,Reeves MJ,Jacobs BS,Maddox K,Choate L,Wehner S,Mullard AJ**. Performance improvement. Closing gaps between evidence-based stroke care guidelines and practices with a collaborative quality improvement project.. *Joint Commission Journal on Quality & Patient Safety*. 2006/09//2006 Sep. 32:517	Level 1, Form Title/abstract screen
3618	**Odeberg H,Rodriguez-Silva B,Salander P,Mårtensson B**. Individualized continuation electroconvulsive therapy and medication as a bridge to relapse prevention after an index course of electroconvulsive therapy in severe mood disorders: a naturalistic 3-year cohort study.. *Journal of ECT*. 2008/09//2008 Sep. 24:183	Level 1, Form Title/abstract screen
3620	**Koelewijn-van Loon MS,van der Weijden T,Ronda G,van Steenkiste B,Winkens B,Elwyn G,Grol R**. Improving lifestyle and risk perception through patient involvement in nurse-led cardiovascular risk management: a cluster-randomized controlled trial in primary care.. *Preventive Medicine*. 2010/01//. 50:35	Level 1, Form Title/abstract screen
3621	**Gibson LE,Ruzek JI,Naturale AJ,Watson PJ,Bryant RA,Rynearson T,Young BH,Hamblen JL**. Interventions for individuals after mass violence and disaster: recommendations from the roundtable on screening and assessment, outreach, and intervention for mental health and substance abuse needs following disasters and mass violence.. *Journal of Trauma Practice*. 2006/10//. 5:1	Level 1, Form Title/abstract screen
3624	. Washington merges divisions and revamps for person-centered care.. *Alcoholism & Drug Abuse Weekly*. 2009/09/14/. 21:1	Level 1, Form Title/abstract screen
3625	. Oregon transformed health system to include key mental health support.. *Mental Health Weekly*. 2012/03/12/. 22:1	Level 1, Form Title/abstract screen
3627	**Baldwin JA,Johnson JL,Benally CC**. Building partnerships between indigenous communities and universities: lessons learned in HIV/AIDS and substance abuse prevention research.. *American Journal of Public Health*. 2009/03/02/Mar2009 Supplement. 99:S77	Level 1, Form Title/abstract screen
3628	**Connors K,Connors Kelley,Bernstein H**. Innovative online smoking prevention education for pediatric providers, 'tween' girls, and their families.. *Journal of Communication in Healthcare*. 2010/04//. 3:9	Level 1, Form Title/abstract screen

3629	**Daniels AS,Adams N,Carroll C,Beinecke RH**. A conceptual model for behavioral health and primary care integration: emerging challenges and strategies for improving international mental health services.. *International Journal of Mental Health*. 2009///Spring2009. 38:100	Level 1, Form Title/abstract screen
3630	**Wilson A,Sinfield P,Rodgers S,Hammersley V,Coleman T**. Drugs to support smoking cessation in UK general practice: are evidence based guidelines being followed?. *Quality & Safety in Health Care*. 2006/08//. #volume#:284	Level 1, Form Title/abstract screen
3631	**Catherine Vanderwater, Julia Culhane**. Successes and Challenges: One Cancer Centre's Experience Implementing a Smoking Cessation Model of Care into Daily Practice.. *Journal of Medical Imaging & Radiation Sciences*. 2014/06//. 45:165	Level 1, Form Title/abstract screen
3632	**Kayla Joy Thompson**. Implementation of the alcohol use disorders identification test to improve practice in a rural primary care clinic.. *Implementation of the Alcohol Use Disorders Identification Test to Improve Practice in a Rural Primary Care Clinic*. 2014/01//. #volume#:88 p	Level 1, Form Title/abstract screen
3636	**Quinlivan JA,Evans SF**. Impact of domestic violence and drug abuse in pregnancy on maternal attachment and infant temperament in teenage mothers in the setting of best clinical practice.. *Archives of Women's Mental Health*. 2005/09//. 8:191	Level 1, Form Title/abstract screen
3637	**Kleinpeter C,Deschenes EP,Blanks J,Lepage CR,Knox M**. Providing recovery services for offenders with co-occurring disorders.. *Journal of Dual Diagnosis*. 2006/12//. 3:59	Level 1, Form Title/abstract screen
3638	**Liz Ledger**. COPD.. *Nursing Standard*. 2015/04//4/1/2015. 29:61	Level 1, Form Title/abstract screen
3640	**Siegal HA,Cole PA,Li L,Eddy MF**. Can a brief clinical practicum influence physicians' communications with patients about alcohol and drug problems? Results of a long-term follow-up.. *Teaching & Learning in Medicine*. 2000///Spring2000. 12:72	Level 1, Form Title/abstract screen
3641	**Cynthia R. Harr, Gaynor I. Yancey**. Social Work Collaboration with Faith Leaders and Faith Groups Serving Families in Rural Areas.. *Journal of Religion & Spirituality in Social Work*. 2014/04//Apr-Jun2014. 33:148	Level 1, Form Title/abstract screen
3642	**Slawson D,Shaughnessy AF,Saririan S,Ebell M,Barry H,Ali U**. POEMs.. *American Family Physician*. 2006/09//9/1/2006. 74:823	Level 1, Form Title/abstract screen
3644	**Boughey L**. Alcohol related brain damage: step by step support leads back to a rewarding life.. *Journal of Dementia Care*. 2005/07//2005 Jul-Aug. 13:22	Level 1, Form Title/abstract screen
3645	**Gunn J,Hegarty K,Nagle C,Forster D,Brown S,Lumley J**. Putting woman-centered care into practice: a new (ANEW) approach to psychosocial risk assessment during pregnancy.. *Birth: Issues in Perinatal Care*. 2006/03//. 33:46	Level 1, Form Title/abstract screen
3646	. New & trends.. *Nurse.com Nursing Spectrum (New York/New Jersey Metro)*. 2011/09/12/2011 Sep 12. 23:42	Level 1, Form Title/abstract screen
3647	. News & trends.. *Nurse.com NurseWeek (West)*. 2011/09/12/2011 Sep 12. 24:38	Level 1, Form Title/abstract screen
3648	**Cristofalo M,Boutain D,Schraufnagel TJ,Bumgardner K,Zatzick D,Roy-Byrne PP, Meg Cristofalo, Doris Boutain, Trevor J Schraufnagel, Kristin**	Level 1, Form Title/abstract

	**Bumgardner, Doug Zatzick, Peter P Roy-Byrne**. Unmet need for mental health and addictions care in urban community health clinics: frontline provider accounts.. *Psychiatric Services*. 2009/04//. 60:505	screen
3649	**Sean M . Getty**. Implementing a Mental Health Program Using the Recovery Model.. *OT Practice*. 2015/02/23/. 20:CE	Level 1, Form Title/abstract screen
3651	**Rohrer JE,Merry SP,Lopez-Jimenez F,Adamson SC,Wilshusen L**. A patient-centered decision rule for referral of patients to weight-loss programs.. *Quality Management in Health Care*. 2007/07//Jul-Sep2007. 16:250	Level 1, Form Title/abstract screen
3652	**Spangler JG,George G,Foley KL,Crandall SJ, John G Spangler, Geeta George, Kristie Long Foley, Sonia J Crandall**. Tobacco intervention training: current efforts and gaps in US medical schools.. *JAMA: Journal of the American Medical Association*. 2002/09/04/. 288:1102	Level 1, Form Title/abstract screen
3654	**Mena MP,Mitrani VB,Muir JA,Santisteban DA**. Collaborative practice. Extended parent-child separations: impact on substance-abusing Hispanic adolescents.. *Journal for Specialists in Pediatric Nursing*. 2008/01//. 13:50	Level 1, Form Title/abstract screen
3656	**Van Hasselt VB,Killam G,Schlessinger KM,DiCicco TM,Anzalone WF Jr.,Leslie TL,George JA,Werder EJ,Massey LL**. The Adolescent Drug Abuse Prevention and Treatment (ADAPT) program: a mental health-law enforcement collaboration.. *Journal of Child & Adolescent Substance Abuse*. 2005/12//. 15:87	Level 1, Form Title/abstract screen
3661	**Graham J**. Motivational interviewing: a hammer looking for a nail?. *Journal of Psychiatric & Mental Health Nursing*. 2004/08//. 11:494	Level 1, Form Title/abstract screen
3663	**van Wormer K**. Harm reduction: a model for social work practice with adolescents.. *Social Policy Journal*. 2004/04//. 3:19	Level 1, Form Title/abstract screen
3664	**Tran NK,Kost GJ**. Guidelines for home testing in primary care: education, integration, information, limitations, and indications.. *Point of Care*. 2006/12//2006 Dec. 5:145	Level 1, Form Title/abstract screen
3667	**Green C**. Providing treatment and care for adolescent substance users.. *Journal of Substance Use*. 2000/03//. 4:227	Level 1, Form Title/abstract screen
3671	**Clemmens DA,Kerr AR**. Improving oral health in women: nurses' call to action.. *MCN: The American Journal of Maternal Child Nursing*. 2008/01//Jan/Feb2008. 33:10	Level 1, Form Title/abstract screen
3672	**McCrystal P,McAleavy G**. Addressing health care in Northern Ireland through collaborative peer education.. *International Journal of Health Promotion & Education*. 2000/08//. 38:76	Level 1, Form Title/abstract screen
3673	**van de Mheen HD**. Demand-driven care: can it work in the addiction field?. *Journal of Substance Use*. 2003/06//. 8:119	Level 1, Form Title/abstract screen
3676	**Wieder BL,Lutz WJ,Boyle P**. Adapting integrated dual disorders treatment for inpatient settings.. *Journal of Dual Diagnosis*. 2005/12//. 2:101	Level 1, Form Title/abstract screen
3677	**Rotgers F,Kishline A**. Moderation Management: a support group for persons who want to reduce their drinking, but not not necessarily abstain.. *International Journal of Self Help & Self Care*. 1999/04//1999-2000. 1:145	Level 1, Form Title/abstract screen

3680	**Wutzke SE,Conigrave KM,Saunders JB,Hall WD**. The long-term effectiveness of brief interventions for unsafe alcohol consumption: a 10-year follow-up.. *Addiction*. 2002/06//. 97:665	Level 1, Form Title/abstract screen
3681	**Heit HA,Gourlay DL**. Urine drug testing in pain medicine.. *Journal of Pain & Symptom Management*. 2004/03//. 27:260	Level 1, Form Title/abstract screen
3683	**Greenblatt J,Koman S**. Treat eating disorders concurrently: treatment will require intensive monitoring at the outset.. *Addiction Professional*. 2006/07//2006 Jul-Aug. 4:43	Level 1, Form Title/abstract screen
3684	**Spencer MS,Muroff JR,Delva J**. Conditional welfare: a family social work perspective on mandatory drug testing...co-published simultaneously in Journal of Family Social Work (The Haworth Press, Inc) Vol. 4, No. 4, 2000, pp. 3-14; and: Substance Abuse Issues Among Families in Diverse Populations (ed: Jorge Delva) The Haworth Press, Inc., 2000, pp. 3-14. *Journal of Family Social Work*. 2000/09/21/. 4:3	Level 1, Form Title/abstract screen
3685	**Hannah Rachel Capon**. Therapist discourse in manualised therapy for alcohol addictions.. *Therapist discourse in manualised therapy for alcohol addictions*. 2014/01//. #volume#:N.PAG p	Level 1, Form Title/abstract screen
3686	**Wulfensmith C**. Understanding the introverted client.. *Addiction Professional*. 2009/03//Mar/Apr2009. 7:3p	Level 1, Form Title/abstract screen
3687	**D'Ambrosio R,Laws KE,Gabriel RM,Hromco J,Kelly P**. Implementing Motivational Interviewing in a Non-MI world: a MI Knowledge Adoption Study.. *Journal of Teaching in the Addictions*. 2006/10//. 5:21	Level 1, Form Title/abstract screen
3689	**Penson RT,Fergus LA,Haston RJ,Clark JR,Demotses A,O'Connell JJ,Chabner BA,Lynch TJ Jr.**. Schwartz Center rounds. The Kenneth B. Schwartz Center at Massachusetts General Hospital Hematology-Oncology Department: hope for the homeless.. *Oncologist*. 2003/09//. 8:488	Level 1, Form Title/abstract screen
3690	**Phillips S,Haycock C,Boyle D**. Development of an alcohol withdrawal protocol: CNS collaborative exemplar.. *Clinical Nurse Specialist: The Journal for Advanced Nursing Practice*. 2006/07//. 20:190	Level 1, Form Title/abstract screen
3694	**Ursula E Bauer, Peter A Briss, Richard A Goodman, Barbara A Bowman**. Prevention of chronic disease in the 21st century: elimination of the leading preventable causes of premature death and disability in the USA.. *Lancet*. 2014/07/05/. 384 North American Edition:45	Level 1, Form Title/abstract screen
3700	**M Abdel-Mawgoud, M K al-Haddad**. Heroin addiction in Bahrain: 15 years experience.. *Addiction*. 1996/12//. 91:1859	Level 1, Form Title/abstract screen
3704	**Ahijevych K,Boyle KK,Burger K**. Microcomputers enhance student health fairs.. *Journal of Nursing Education*. 1985/01//. 24:16	Level 1, Form Title/abstract screen
3705	**Busen NH,Beech B**. A collaborative model for community-based health care screening of homeless adolescents.. *Journal of Professional Nursing*. 1997/09//1997 Sep-Oct. 13:316	Level 1, Form Title/abstract screen
3706	**Boyle AH,Locke DL**. Update on chronic obstructive pulmonary disease.. *MEDSURG Nursing*. 2004/02//. 13:42	Level 1, Form Title/abstract screen
3707	**Pauline E. Hill**. Perinatal Addiction: Providing Compassionate and Competent Care.. *Clinical Obstetrics & Gynecology*. 2013/03//. 56:178	Level 2, Form Full Text

		Screening
3708	**Jessica W Blanchard, J T Petherick, Heather Basara**. Stakeholder engagement: a model for tobacco policy planning in Oklahoma Tribal communities.. *American Journal of Preventive Medicine*. 2015/01/02/Jan2015 Supplement 1. 48:S44	Level 1, Form Title/abstract screen
3709	**Alexander P**. An investigation of inpatient referrals to a cinical psychologist in a hospice.. *European Journal of Cancer Care*. 2004/03//. 13:36	Level 1, Form Title/abstract screen
3710	**Hackbarth DP,Schnopp-Wyatt D,Katz D,Williams J,Silvestri B,Pfleger M**. Collaborative research and action to control the geographic placement of outdoor advertising of alcohol and tobacco products in Chicago.. *Public Health Reports*. 2001/11//Nov/Dec2001. 116:558	Level 1, Form Title/abstract screen
3711	**Baldwin JA,Rolf JE,Johnson J,Bowers J,Benally C,Trotter RT II**. Developing culturally sensitive HIV/AIDS and substance abuse prevention curricula for Native American youth.. *Journal of School Health*. 1996/11//. 66:322	Level 1, Form Title/abstract screen
3712	**Arborelius E,Nyberg K**. How should midwives discuss smoking behaviour in pregnancy with women of low educational attainment?. *Midwifery*. 1997/12//1997 Dec. 13:210	Level 1, Form Title/abstract screen
3713	**Smith GR Jr.,Manderscheid RW,Flynn LM,Steinwachs DM**. Principles for assessment of patient outcomes in mental health care.. *Psychiatric Services*. 1997/08//. 48:1033	Level 1, Form Title/abstract screen
3715	. Waiting lists for SUD treatment at the VA nothing new.. *Alcoholism & Drug Abuse Weekly*. 2014/06/09/. 26:4	Level 1, Form Title/abstract screen
3717	**Workman E,Hendrix J**. Anxiety and depression in a psychiatrically informed pain medicine practice.. *Psychiatric Times*. 2007/12//. 24:62	Level 1, Form Title/abstract screen
3718	**Jumnoodoo R,Marlatt A,Coyne P,Matt E,Singaram E,Lambert L**. Development of a 'whole system' approach to relapse prevention (RP) in Brent Mental Health Services.. *N2N: Nurse2Nurse*. 2002/05//2002 May. 2:46	Level 1, Form Title/abstract screen
3722	**Ka'ano'i ME,Braun KL,Gotay CC**. Primary care physicians' knowledge, attitudes and practices related to cancer screening and cancer prevention clinical trials.. *Pacific Health Dialog*. 2004/09//2004 Sep. 11:160	Level 1, Form Title/abstract screen
3723	**Pringle-Nelson C,Perry GP**. The ferris wheel: understanding FASD: compassion, flexibility are important elements in treating young clients affected by prenatal alcohol exposure.. *Addiction Professional*. 2006/11//2006 Nov. 4:24	Level 1, Form Title/abstract screen
3724	**Alphonso CD**. Reflection on a critical incident.. *Contemporary Nurse: A Journal for the Australian Nursing Profession*. 2007/02//. 24:89	Level 1, Form Title/abstract screen
3726	**P J Raue, G S Alexopoulos, M L Bruce, S Klimstra, B H Mulsant, J J Gallo**. The systematic assessment of depressed elderly primary care patients.. *International Journal of Geriatric Psychiatry*. 2001/06//. 16:560	Level 1, Form Title/abstract screen
3727	**Amy Dawel, Kaarin J Anstey**. Interventions for Midlife Smoking Cessation: A Literature Review.. *Australian Psychologist*. 2011/09//. 46:190	Level 1, Form Title/abstract screen
3728	. Tailored, individualized treatment plus naltrexone effective for alcohol-dependent patients.. *DATA: The Brown University Digest of Addiction Theory &*	Level 1, Form Title/abstract

	Application. 2007/10//. 26:1	screen
3729	**Elizabeth Murray**. Screening and brief intervention for alcohol use disorders in primary care.. *BMJ: British Medical Journal*. 2013/01/26/. 346:8	Level 1, Form Title/abstract screen
3730	**Judy Cain**. Abuse and addiction in females: program offered with Brandywine Counseling Center.. *DNA Reporter*. 2013/08//2013 Aug-Oct. 38:5	Level 1, Form Title/abstract screen
3732	**Rosenfeld SL,Keenan PM,Fox DJ,Chase LH,Melchiono MW,Woods ER**. Youth perceptions of comprehensive adolescent health services through the Boston HAPPENS program.. *Journal of Pediatric Healthcare*. 2000/03//2000 Mar-Apr. 14:60	Level 1, Form Title/abstract screen
3733	**J B Saunders, O G Aasland, A Amundsen, M Grant**. Alcohol consumption and related problems among primary health care patients: WHO collaborative project on early detection of persons with harmful alcohol consumption--I.. *Addiction*. 1993/03//. 88:349	Level 1, Form Title/abstract screen
3734	. Directions for Future Patient-Centered and Comparative Effectiveness Research for People With Serious Mental Illness.. *Schizophrenia Bulletin*. 2014/01/02/. 40:i	Level 1, Form Title/abstract screen
3736	**Paris W,Thompson S,Rihner T,Quisenberry M,Cooper DKC**. A comparison of transplant patient and social worker attitudes in regard to transplant patient psychosocial selection criteria, role expectations, and communication style.. *Social Work in Health Care*. 1996/06//. 23:39	Level 1, Form Title/abstract screen
3737	**Shpilko I**. Russian-American health care: bridging the communication gap between physicians and patients.. *Patient Education & Counseling*. 2006/12//. 64:331	Level 1, Form Title/abstract screen
3738	**Bryant-Jefferies R**. Becoming an alcohol counsellor.. *CPJ: Counselling & Psychotherapy Journal*. 2001/08//2001 Aug. 12:18	Level 1, Form Title/abstract screen
3742	**Reiskin H,Lindenberg CS**. Involving the target population and their providers in evaluation of substance abuse videos.. *NursingConnections*. 1992///1992 Winter. 5:47	Level 1, Form Title/abstract screen
3743	**Caudle P**. Providing culturally sensitive health care to Hispanic clients.. *Nurse Practitioner*. 1993/12//1993 Dec. 18:40	Level 1, Form Title/abstract screen
3744	**Wiest JV**. The hospice advantage.. *American Journal of Hospice & Palliative Care*. 1992/11//1992 Nov-Dec. 9:13	Level 1, Form Title/abstract screen
3745	**Fernandez J,Rooney G,Leahy M,Alcock J,Baski N**. Shared care: a working relationship?. *N2N: Nurse2Nurse*. 2004/08//2004 Aug. 4:43	Level 1, Form Title/abstract screen
3746	**Ford T**. Effective strategies for working with dually diagnosed clients: clinicians need creative approaches to assessment, treatment.. *Addiction Professional*. 2003/05//2003 May. 1:21	Level 1, Form Title/abstract screen
3747	**J F A Murphy**. Practicing medicine thirty years on.. *Irish Medical Journal*. 2014/05//2014 May. 107:132	Level 1, Form Title/abstract screen
3748	**Lang MA,Davidson L,Bailey P,Levine MS**. Clinicians' and clients' perspectives on the impact of assertive community treatment.. *Psychiatric*	Level 1, Form Title/abstract

	Services. 1999/10//. 50:1331	screen
3749	**Shaked G,Renert N,Mahuda I,Strous RD**. Psychiatric care in the Middle East: a 'mental health supermarket' in the town of Lod.. *Psychiatric Rehabilitation Journal*. 2004///Winter2004. 27:207	Level 1, Form Title/abstract screen
3752	**Sheppard R,Maloney S,Maye M,Ward J,Geoghegan N**. The best laid care plans.. *World of Irish Nursing & Midwifery*. 2009/07//Jul/Aug2009. 17:42	Level 2, Form Full Text Screening
3753	**A Golay, E Brock, R Gabriel, T Konrad, N Lalic, M Laville, G Mingrone, J Petrie, T-M Phan, K H Pietiläinen, C-H Anderwald**. Taking small steps towards targets - perspectives for clinical practice in diabetes, cardiometabolic disorders and beyond.. *International Journal of Clinical Practice*. 2013/04//. 67:322	Level 1, Form Title/abstract screen
3754	**Thomas SP,Shattell M,Martin T**. What's therapeutic about the therapeutic milieu?. *Archives of Psychiatric Nursing*. 2002/06//2002 Jun. 16:99	Level 1, Form Title/abstract screen
3757	**Morrow M,Ngoc DH,Hoang TT,Trinh TH**. Smoking and young women in Vietnam: the influence of normative gender roles.. *Social Science & Medicine*. 2002/08/15/. 55:681	Level 1, Form Title/abstract screen
3758	**McPhillips-Tangum C,Cahill A,Bocchino C,Cutler CM**. Addressing tobacco in managed care: results of the 2000 survey.. *American Journal of Managed Care*. 2002/06/26/2002 Jun 25 Preventive Medicine in Managed Care. 3:85	Level 1, Form Title/abstract screen
3759	**T van Elderen-van Kemenade, S Maes, Y van den Broek**. Effects of a health education programme with telephone follow-up during cardiac rehabilitation.. *British Journal of Clinical Psychology*. 1994/09//1994 Sep. 33 (Pt 3):367	Level 1, Form Title/abstract screen
3760	**Woods ER,Samples CL,Melchiono MW,Harris SK**. Boston HAPPENS Program: HIV-positive, homeless, and at-risk youth can access care through youth-oriented HIV services.. *Seminars in Pediatric Infectious Diseases*. 2003/01//2003 Jan. 14:43	Level 1, Form Title/abstract screen
3761	. Leading researcher tells R.I. audience to emphasize clients in system change.. *Alcoholism & Drug Abuse Weekly*. 2009/01/26/. 21:1	Level 1, Form Title/abstract screen
3762	. Screening is not about the paperwork: Minkoff.. *Alcoholism & Drug Abuse Weekly*. 2012/04/23/. 24:7	Level 1, Form Title/abstract screen
3763	**Veronica Hall**. Understanding the causes and symptoms of IBD.. *Nursing Times*. 2014/11/12/. 110:16	Level 1, Form Title/abstract screen
3766	**Whatling J**. Managing chronic obstructive disease.. *Nursing Standard*. 1995/11/15/1995 Nov 15-21. 10:34	Level 1, Form Title/abstract screen
3767	**Nancy Murphy**. Studying and facilitating the development, installation, and initial implementation of an interdisciplinary buprenorhine treatment/practice with a publicly funded, HIV primary care, designated AIDS center in New York City: A practice-focused.... *Studying & Facilitating the Development, Installation & Initial Implementation of an Interdisciplinary Buprenorhine Treatment/practice With a Publicly Funded, HIV Primary Care, Designated AIDS Center in New York City: A Practice-focused...*. 2013/01//. #volume#:281 p	Level 1, Form Title/abstract screen
3768	**Carr VJ,Lewin TJ,Walton JM,Faehrmann C,Reid ALA**. Consultation-	Level 1, Form

	liaison psychiatry in general practice.. *Australian & New Zealand Journal of Psychiatry*. 1997/02//1997 Feb. 31:85	Title/abstract screen
3769	**Currier PA**. An Azorean-American nursing accord of cooperation and exchange: nurturing the process.. *Journal of Multicultural Nursing & Health (JMCNH)*. 2001///2001 Summer. 7:23	Level 1, Form Title/abstract screen
3770	**Wright C**. Healing the invisible wound: self-help in addictions treatment.. *Journal of the American Academy of Physician Assistants*. 1990/06//1990 Jun. 3:261	Level 1, Form Title/abstract screen
3773	. A patient's perspective: Army Major Steve McCullough, retired, wounded warrior.. *Case in Point*. 2012/07//2012 Jul. 10:15	Level 1, Form Title/abstract screen
3774	**Arborelius E,Bremberg S, E Arborelius, S Bremberg**. Prevention in practice. How do general practitioners discuss life-style issues with their patients?. *Patient Education & Counseling*. 1994/04//1994 Apr. 23:23	Level 1, Form Title/abstract screen
3775	**Tom McKeithen, Sheila Robertson, Mike Speight**. Developing clinical competencies to assess learning needs and outcomes: The experience of the CS2day initiative.. *Journal of Continuing Education in the Health Professions*. 2011/09/02/Fall2011 Supplement. 31:S21	Level 1, Form Title/abstract screen
3776	**Corse SJ,Hirschinger NB,Caldwell S**. Brief reports. Conducting treatment outcome research in a community mental health center: a university-agency collaboration.. *Psychiatric Rehabilitation Journal*. 1996///Summer1996. 20:59	Level 1, Form Title/abstract screen
3780	**Laura Zimmerman**. MEDiC's 'Health Talk' benefits underserved.. *Nursingmatters*. 2012/12//2012 Dec. 23:5	Level 1, Form Title/abstract screen
3783	**Mary W. Ales, Shelly B. Rodrigues, Robyn Snyder, Mary Conklin**. Developing and implementing an effective framework for collaboration: The experience of the CS2day collaborative.. *Journal of Continuing Education in the Health Professions*. 2011/09/02/Fall2011 Supplement. 31:S13	Level 1, Form Title/abstract screen
3784	**Saitz R,Larson MJ,LaBelle C,Richardson J,Samet JH**. The case for chronic disease management for addiction.. *Journal of Addiction Medicine*. 2008/06//. 2:55	Level 2, Form Full Text Screening
3785	**Cynthia D. Connelly**. Perinatal Mental Health Model: Design, Implementation, and Acceptability of a Community-Based Collaborative Care Intervention.. *JOGNN: Journal of Obstetric, Gynecologic & Neonatal Nursing*. 2011/05/02/May2011 Supplement 1. 40:S100	Level 1, Form Title/abstract screen
3786	**Peele S**. Endpage. How I found common cause with social workers.. *Journal of Social Work Practice in the Addictions*. 2004/09//. 4:117	Level 1, Form Title/abstract screen
3787	**Parsons ML,Warner-Robbins C**. In our community. Holistic nursing on the front lines: a community program helps women's transition from jail to society.. *AJN American Journal of Nursing*. 2002/06//. 102:73	Level 1, Form Title/abstract screen
3790	**Johns C**. To educate or what? Marking the holistic ground within the CCU.. *Nursing in Critical Care*. 2001/11//2001 Nov-Dec. 6:297	Level 1, Form Title/abstract screen
3792	**Francis X Holt**. False-positive self-reporting at a Massachusetts Level III.7 detox: Intersection of individual level of need and systemic constraint of choice.. *False-positive Self-reporting at a Massachusetts Level Iii.7 Detox: Intersection of Individual Level of Need & Systemic Constraint of Choice*. 2011/01//. #volume#:120 p	Level 1, Form Title/abstract screen

3797	**Eric Goplerud**. SBIRT coming to hospitals via Joint Commission...Screening, Brief Intervention, and Referral to Treatment. *Alcoholism & Drug Abuse Weekly*. 2012/05/28/. 24:5	Level 1, Form Title/abstract screen
3798	**Mulvihill DG**. Help-seeking for alcohol dependency by women with post-traumatic stress disorder and a history of intimate partner violence.. *Help-seeking for Alcohol Dependency by Women With Post-traumatic Stress Disorder & a History of Intimate Partner Violence*. 2009/01//. #volume#:279 p	Level 1, Form Title/abstract screen
3799	. Spotlight.. *PARADIGM (Targeted Publications Group, Inc)*. 2012///2012 Spring. 16:2	Level 1, Form Title/abstract screen
3800	. Therapeutic alliance may influence medical interventions for alcohol dependence.. *DATA: The Brown University Digest of Addiction Theory & Application*. 2008/09//. 27:3	Level 1, Form Title/abstract screen
3803	**Lynn Owens, Graham Butcher, Ian Gilmore, Ruwanthi Kolamunnage-Dona, James Oyee, Liz Perkins, Tom Walley, Paula Williamson, Ken Wilson, Munir Pirmohamed**. A randomised controlled trial of extended brief intervention for alcohol dependent patients in an acute hospital setting (ADPAC).. *BMC Public Health*. 2011/01//. 11:528	Level 1, Form Title/abstract screen
3804	**Brink PJ**. Patientology one more time.. *Western Journal of Nursing Research*. 1991/02//. 13:9	Level 1, Form Title/abstract screen
3805	**Adrian H Taylor, Emma S Everson-Hock, Michael Ussher**. Integrating the promotion of physical activity within a smoking cessation programme: findings from collaborative action research in UK Stop Smoking Services.. *BMC Health Services Research*. 2010/01//. 10:317	Level 1, Form Title/abstract screen
3808	**Staiger PK,Long C,Baker A**. Health service systems and comorbidity: stepping up to the mark.. *Mental Health & Substance Use: Dual Diagnosis*. 2010/06//. 3:148	Level 1, Form Title/abstract screen
3809	**Mohatt DF**. Collaborative mental health care needed to help returning veterans.. *Rural Clinician Quarterly*. 2008///2008 Summer. #volume#:1	Level 1, Form Title/abstract screen
3811	**Elaine M Edelman**. Patients' perception of family involvement and its relationship to medication adherence for persons with schizophrenia and schizoaffective disorders.. *Patients' Perception of Family Involvement & its Relationship to Medication Adherence for Persons With Schizophrenia & Schizoaffective Disorders*. 2010/01//. #volume#:158 p	Level 1, Form Title/abstract screen
3812	**Kathleen Sciacca**. Integrating three interventions for dual-diagnosis patients.. *Alcoholism & Drug Abuse Weekly*. 2012/02/06/. 24:5	Level 1, Form Title/abstract screen
3813	**Munro I,Edward K**. Mental illness and substance use: an Australian perspective.. *International Journal of Mental Health Nursing*. 2008/08//. 17:255	Level 1, Form Title/abstract screen
3814	**Galanter M,Glickman L,Singer D**. An overview of outpatient treatment of adolescent substance abuse.. *Substance Abuse*. 2007/06//. 28:51	Level 1, Form Title/abstract screen
3815	**Lehman WE,Fletcher BW,Wexler HK,Melnick G**. Organizational factors and collaboration and integration activities in criminal justice and drug abuse treatment agencies.. *Drug & Alcohol Dependence*. 2009/08/02/Aug2009 Supplement 1. 103:S65	Level 1, Form Title/abstract screen

3816	**Jamie Crawley**. The experience of chronic pain as described by African American indigent adults attending an urban primary care clinic.. *Experience of Chronic Pain as Described by African American Indigent Adults Attending an Urban Primary Care Clinic*. 2010/01//. #volume#:306 p	Level 1, Form Title/abstract screen
3817	**Litt MD,Kadden RM**. Looking into the Black Box: a commentary on Individualized Assessment and Treatment Program (IATP) for alcohol dependence.. *DATA: The Brown University Digest of Addiction Theory & Application*. 2010/03//. 29:8	Level 1, Form Title/abstract screen
3818	**Taber M**. Adolescent treatment. Programs can adjust to wraparound model.. *Addiction Professional*. 2006/09//2006 Sep-Oct. 4:50	Level 1, Form Title/abstract screen
3820	. Newsletter.. *American Family Physician*. 2007/07/15/. 76:179	Level 1, Form Title/abstract screen
3821	**DeSimone ME,Crowe A**. Nonpharmacological approaches in the management of hypertension.. *Journal of the American Academy of Nurse Practitioners*. 2009/04//. 21:189	Level 1, Form Title/abstract screen
3822	**Eisele J**. Balancing body and mind in treatment: centers see biochemical repair as a needed element for success.. *Addiction Professional*. 2007/01//2007 Jan-Feb. 5:23	Level 1, Form Title/abstract screen
3824	**McMillan I**. Tribal quest... US Indians... Indian Health Service (IHS). *Nursing Times*. 1995/01/18/1995 Jan 18-24 Prof Dev. 91:46	Level 1, Form Title/abstract screen
3826	. AAFP news now.. *American Family Physician*. 2008/02/15/. 77:423	Level 1, Form Title/abstract screen
3827	**Montauk SL**. The homeless in America: adapting your practice.. *American Family Physician*. 2006/10//10/1/2006. 74:1132	Level 1, Form Title/abstract screen
3828	**Lehman AF,Lieberman JA,Dixon LB,McGlashan TH,Miller AL,Perkins DO,Kreyenbuhl J, Anthony F Lehman, Jeffrey A Lieberman, Lisa B Dixon, Thomas H McGlashan, Alexander L Miller, Diana O Perkins, Julie Kreyenbuhl**. Practice guideline for the treatment of patients with schizophrenia, second edition.. *American Journal of Psychiatry*. 2004/02/02/Feb2004 Supplement. 161:1	Level 1, Form Title/abstract screen
3829	**Manderscheid RW**. Perspectives. A room in the medical home: consumers deserve 'first-floor' access to mental and substance use care in collaborative care systems.. *Behavioral Healthcare*. 2007/10//. 27:45	Level 1, Form Title/abstract screen
3831	**Wade D,Johnston A,Campbell B,Littlefield L**. Early intervention services in youth mental health.. *Clinical Psychologist*. 2007/10//. 11:108	Level 1, Form Title/abstract screen
3832	**Cheung AH**. Review: more high quality studies needed to determine the effects on patient outcomes of psychiatric care guideline implementation.. *Evidence Based Mental Health*. 2008/02//. 11:21	Level 1, Form Title/abstract screen
3833	**Gilbody SM**. IMPACT collaborative care programme reduces suicide ideation in depressed older adults.. *Evidence Based Mental Health*. 2007/05//. 10:51	Level 1, Form Title/abstract screen
3835	**Troum OM,Crues JV III**. The young adult with hip pain: diagnosis and medical treatment, circa 2004.. *Clinical Orthopaedics & Related Research*.	Level 1, Form Title/abstract

	2004/01//2004 Jan. 418:9	screen
3836	**Felix-Aaron K,Moy E,Kang M,Patel M,Chesley FD,Clancy C**. Variation in quality of men's health care by race/ethnicity and social class.. *Medical Care*. 2005/03/02/2005 Mar Supplement. 43:I	Level 1, Form Title/abstract screen
3838	**Barker P**. Psychotherapy series. The humanistic therapies... second of five articles.. *Nursing Times*. 1998/02/11/1998 Feb 11-17. 94:52	Level 1, Form Title/abstract screen
3839	**Miller WR**. Motivational interviewing in service to health promotion.. *American Journal of Health Promotion*. 2004/01//Jan/Feb2004. 18:N	Level 1, Form Title/abstract screen
3840	**Goering P**. Collaborative care speeds recovery from depression.. *Evidence Based Mental Health*. 2003/11//. 6:116	Level 1, Form Title/abstract screen
3841	**Christman SK**. Intervention to slow progression of peripheral arterial disease.. *Intervention to Slow Progression of Peripheral Arterial Disease*. 2003/01//. #volume#:123 p	Level 1, Form Title/abstract screen
3842	. CSAT and NIDA promote use of the ASI for treatment planning.. *Alcoholism & Drug Abuse Weekly*. 2006/10/30/. 18:7	Level 1, Form Title/abstract screen
3843	. Collaborative care for acutely injured trauma survivors can reduce post-traumatic distress symptoms and alcohol abuse.. *AHRQ Research Activities*. 2004/09//2004 Sep. #volume#:19	Level 1, Form Title/abstract screen
3844	**Bisson JI**. Trauma-focused group psychotherapy is not effective for posttraumatic stress disorder in Vietnam veterans.. *Evidence Based Mental Health*. 2003/11//. 6:124	Level 1, Form Title/abstract screen
3847	**Nowak F,Nagle L,Bernardo A**. Review: case management programmes improve patient outcomes [commentary on Ferguson JA, Weinberger M. Case management programs in primary care. J GEN INTERN MED 1998 Feb;13:123-6].. *Evidence Based Nursing*. 1998/10//. #volume#:128	Level 1, Form Title/abstract screen
3848	**Bienkowski J**. An overview of the progression of diabetic retinopathy with treatment recommendations.. *Nurse Practitioner*. 1994/07//1994 Jul. 19:50	Level 1, Form Title/abstract screen
3850	**Shakeshaft A,Clifford A,Shakeshaft M**. Reducing alcohol related harm experienced by Indigenous Australians: identifying opportunities for Indigenous primary health care services.. *Australian & New Zealand Journal of Public Health*. 2010/07/02/Jul2010 Supplement. 34:S41	Level 1, Form Title/abstract screen
3851	**Gutman SA**. The psychosocial sequelae of traumatic brain injury, part II: treatment.. *OT Practice*. 2001/03/06/2001 Mar 5 Suppl. 6:CE	Level 1, Form Title/abstract screen
3855	**M. Zhang, C. C. Yang,Ieee**. Classifying User Intention and Social Support Types in Online Healthcare Discussions. *2014 Ieee International Conference on Healthcare Informatics*. 2014. #volume#:51-60	Level 1, Form Title/abstract screen
3857	**D. Zatzick, J. Russo, F. Rivara, P. Roy-Byrne, G. Jurkovich, W. Katon**. The detection and treatment of posttraumatic distress and substance intoxication in the acute care inpatient setting. *General Hospital Psychiatry*. 2005. 27:57-62	Level 1, Form Title/abstract screen
3864	**E. K. Yuen, E. M. Goetter, J. D. Herbert, E. M. Forman**. Challenges and Opportunities in Internet-Mediated Telemental Health. *Professional Psychology-Research and Practice*. 2012. 43:1-8	Level 1, Form Title/abstract screen

3865	**D. A. Young, M. Shumway, A. Flentje, E. D. Riley**. The Relationship Between Childhood Abuse and Violent Victimization in Homeless and Marginally Housed Women: The Role of Dissociation as a Potential Mediator. *Psychological Trauma-Theory Research Practice and Policy*. 2017. 9:613-621	Level 1, Form Title/abstract screen
3866	**B. J. H. Yarborough, S. P. Stumbo, D. McCarty, J. Mertens, C. Weisner, C. A. Green**. Methadone, buprenorphine and preferences for opioid agonist treatment: A qualitative analysis. *Drug and Alcohol Dependence*. 2016. 160:112-118	Level 1, Form Title/abstract screen
3870	**H. Yaman, M. Akdeniz, E. Katirci**. DIABETES EDUCATION IN PRIMARY CARE AND THE 5 MINUTE SURVIVAL KIT. *Nobel Medicus*. 2010. 6:5-9	Level 1, Form Title/abstract screen
3872	**R. Wynn, K. Karlsen, B. Lorntzsen, T. N. Bjerke, S. Bergvik**. Users' and GPs' causal attributions of illegal substance use: An exploratory interview study. *Patient Education and Counseling*. 2009. 76:227-232	Level 1, Form Title/abstract screen
3877	**Y. Woodhead, D. Cameron, S. Blackwell, F. W. Seymour**. Family Court Judges' Decisions Regarding Post-Separation Care Arrangements for Young Children. *Psychiatry Psychology and Law*. 2015. 22:520-534	Level 1, Form Title/abstract screen
3878	**J. Wood, K. Crew, R. Kukafka, J. Finkelstein,Ieee**. A Comprehensive Informatics Framework to Increase Breast Cancer Risk Assessment and Chemoprevention in the Primary Care Setting. *2016 Ieee International Conference on Healthcare Informatics*. 2016. #volume#:293-296	Level 1, Form Title/abstract screen
3885	**T. M. Winhusen, F. Kropp**. Psychosocial treatments for women with substance use disorders. *Obstetrics and Gynecology Clinics of North America*. 2003. 30:483-+	Level 1, Form Title/abstract screen
3886	**T. E. Wilson, E. S. Kay, B. Turan, M. O. Johnson, M. C. Kempf, J. M. Turan, M. H. Cohen, A. A. Adimora, M. Pereyra, E. T. Golub, L. Goparaju, L. Murchison, G. M. Wingood, L. R. Metsch**. Healthcare Empowerment and HIV Viral Control: Mediating Roles of Adherence and Retention in Care. *American Journal of Preventive Medicine*. 2018. 54:756-764	Level 1, Form Title/abstract screen
3887	**S. Wilson, M. King, G. Yeowell, C. Wibberley**. Disclosure decisions within transition narratives: How ex-drug users decide what information to share when working toward employment in the substance misuse field. *Journal of Substance Use*. 2016. 21:575-580	Level 1, Form Title/abstract screen
3888	**M. Wilson, J. M. Roll, C. Corbett, C. Barbosa-Leiker**. Empowering Patients with Persistent Pain Using an Internet-based Self-Management Program. *Pain Management Nursing*. 2015. 16:503-514	Level 1, Form Title/abstract screen
3892	**G. C. Williams, H. A. McGregor, D. King, C. C. Nelson, R. E. Glasgow**. Variation in perceived competence, glycemic control, and patient satisfaction: relationship to autonomy support from physicians. *Patient Education and Counseling*. 2005. 57:39-45	Level 1, Form Title/abstract screen
3893	**I. R. Wiechers, O. Freudenreich**. The Role of Consultation-Liaison Psychiatrists in Improving Health Care of Patients with Schizophrenia. *Psychosomatics*. 2013. 54:22-27	Level 1, Form Title/abstract screen
3896	**N. Weymann, M. Harter, F. Petrak, J. Dirmaier**. Health information, behavior change, and decision support for patients with type 2 diabetes: development of a tailored, preference-sensitive health communication application. *Patient Preference and Adherence*. 2013. 7:1091-1099	Level 1, Form Title/abstract screen
3901	**I. Weissbecker, C. Clark**. The impact of violence and abuse on women's physical health: Can trauma-informed treatment make a difference?. *Journal of*	Level 1, Form Title/abstract

	Community Psychology. 2007. 35:909-923	screen
3911	**K. Waters, S. Holttum, I. Perrin**. Narrative and attachment in the process of recovery from substance misuse. *Psychology and Psychotherapy-Theory Research and Practice*. 2014. 87:222-236	Level 2, Form Full Text Screening
3913	**C. Wang, B. Cho, D. Xiao, D. Wajsbrot, P. W. Park**. Effectiveness and safety of varenicline as an aid to smoking cessation: results of an inter-Asian observational study in real-world clinical practice. *International Journal of Clinical Practice*. 2013. 67:469-476	Level 1, Form Title/abstract screen
3915	**G. D. Walters**. Working alliance between substance abusing offenders and their parole officers and counselors: its impact on outcome and role as a mediator. *Journal of Crime & Justice*. 2016. 39:421-437	Level 1, Form Title/abstract screen
3916	**G. D. Walters**. Proactive criminal thinking, cold heartedness, and counselor rapport in correctional clients participating in substance abuse treatment. *Personality and Individual Differences*. 2016. 98:239-243	Level 1, Form Title/abstract screen
3918	**T. von Braun, S. Larsson, Y. Sjoblom**. Narratives of Clients' Experiences of Drug Use and Treatment of Substance Use-Related Dependency. *Substance Use & Misuse*. 2013. 48:1404-1415	Level 1, Form Title/abstract screen
3919	**J. Volavka, R. A. Van Dorn, L. Citrome, R. S. Kahn, W. W. Fleischhacker, P. Czobor**. Hostility in schizophrenia: An integrated analysis of the combined Clinical Antipsychotic Trials of Intervention Effectiveness (CATIE) and the European First Episode Schizophrenia Trial (EUFEST) studies. *European Psychiatry*. 2016. 31:13-19	Level 1, Form Title/abstract screen
3921	**I. Vogt**. Coercion and Quasi-Compulsory Settings in the Treatment of Substance Dependent Men and Women in Germany: a Review. *Suchttherapie*. 2012. 13:81-89	Level 1, Form Title/abstract screen
3924	**A. Verdejo-Garcia, J. Verdejo-Roman, N. Albein-Urios, J. M. Martinez-Gonzalez, C. Soriano-Mas**. Brain substrates of social decision-making in dual diagnosis: cocaine dependence and personality disorders. *Addiction Biology*. 2017. 22:457-467	Level 1, Form Title/abstract screen
3925	**A. Verdejo-Garcia, A. Benbrook, F. Funderburk, P. David, J. L. Cadet, K. I. Bolla**. The differential relationship between cocaine use and marijuana use on decision-making performance over repeat testing with the Iowa Gambling Task. *Drug and Alcohol Dependence*. 2007. 90:2-11	Level 1, Form Title/abstract screen
3927	**K. L. Venner, H. Matzger, A. A. Forcehimes, R. H. Moos, S. W. Feldstein, M. L. Willenbring, C. Weisner**. Course of recovery from alcoholism. *Alcoholism-Clinical and Experimental Research*. 2006. 30:1079-1090	Level 1, Form Title/abstract screen
3928	**D. I. Velligan, P. J. Weiden, M. Sajatovic, J. Scott, D. Carpenter, R. Ross, J. P. Docherty**. The Expert Consensus Guideline Series: Adherence Problems in Patients with Serious and Persistent Mental Illness Introduction. *Journal of Clinical Psychiatry*. 2009. 70:6-46	Level 1, Form Title/abstract screen
3930	**J. K. Vederhus, T. Clausen, K. Humphreys**. Assessing understandings of substance use disorders among Norwegian treatment professionals, patients and the general public. *Bmc Health Services Research*. 2016. 16:10	Level 1, Form Title/abstract screen
3939	**R. A. Van Dorn, K. J. Grimm, S. L. Desmarais, S. J. Tueller, K. L. Johnson, M. S. Swartz**. Leading indicators of community-based violent events among adults with mental illness. *Psychological Medicine*. 2017. 47:1179-1191	Level 1, Form Title/abstract screen
3940	**R. A. Van Dorn, E. B. Elbogen, A. D. Redlich, J. W. Swanson, M. S. Swartz, S. Mustillo**. The relationship between mandated community treatment and perceived barriers to care in persons with severe mental illness. *International*	Level 1, Form Title/abstract screen

	Journal of Law and Psychiatry. 2006. 29:495-506	
3944	**D. Urada, C. Teruya, L. Gelberg, R. Rawson**. Integration of substance use disorder services with primary care: health center surveys and qualitative interviews. *Substance Abuse Treatment Prevention and Policy*. 2014. 9:9	Level 1, Form Title/abstract screen
3948	**S. G. Trusz, A. W. Wagner, J. Russo, J. Love, D. E. Zatzick**. Assessing Barriers to Care and Readiness for Cognitive Behavioral Therapy in Early Acute Care PTSD Interventions. *Psychiatry-Interpersonal and Biological Processes*. 2011. 74:207-223	Level 1, Form Title/abstract screen
3949	**K. J. Trudeau, R. A. Black, J. L. Kamon, S. Sussman**. A Randomized Controlled Trial of an Online Relapse Prevention Program for Adolescents in Substance Abuse Treatment. *Child & Youth Care Forum*. 2017. 46:437-454	Level 1, Form Title/abstract screen
3950	**R. B. Trivedi, J. A. Nieuwsma, J. W. Williams**. Examination of the Utility of Psychotherapy for Patients with Treatment Resistant Depression: A Systematic Review. *Journal of General Internal Medicine*. 2011. 26:643-650	Level 1, Form Title/abstract screen
3951	**M. H. Trivedi, E. J. Daly**. Measurement-based care for refractory depression: A clinical decision support model for clinical research and practice. *Drug and Alcohol Dependence*. 2007. 88:S61-S71	Level 1, Form Title/abstract screen
3952	**E. Tomba**. Assessment of Lifestyle in Relation to Health. *Psychosomatic Assessment: Strategies to Improve Clinical Practice*. 2012. 32:72-96	Level 1, Form Title/abstract screen
3955	**B. L. Tiep, M. C. Barnett**. Disease Management for Chronic Obstructive Pulmonary Disease A Clinical Strategy. *Disease Management & Health Outcomes*. 2008. 16:305-313	Level 1, Form Title/abstract screen
3959	**N. Tejani, R. Rosenheck, J. Tsai, W. Kasprow, J. F. McGuire**. Incarceration Histories of Homeless Veterans and Progression Through a National Supported Housing Program. *Community Mental Health Journal*. 2014. 50:514-519	Level 1, Form Title/abstract screen
3961	**D. I. Taylor, C. Lennings,Alcohol, F. D. N. Drug**. Ethical decision-making and convergence of views between doctors and long-term prescribed opiate users. *Drugs - Policies, Programs and People - 1996 Conference Papers*. 1996. #volume#:248-253	Level 1, Form Title/abstract screen
3962	**C. Tarquinio, J. Kivits, L. Minary, J. Coste, F. Alla**. Evaluating complex interventions: Perspectives and issues for health behaviour change interventions. *Psychology & Health*. 2015. 30:35-51	Level 1, Form Title/abstract screen
3963	**O. Taieb, S. Chevret, M. R. Moro, M. G. Weiss, A. Biadi-Imhof, A. Reyre, T. Baubet**. Impact of Migration on Explanatory Models of Illness and Addiction Severity in Patients With Drug Dependence in a Paris Suburb. *Substance Use & Misuse*. 2012. 47:347-355	Level 1, Form Title/abstract screen
3966	**H. Sweeting**. WORKSHOP - COLLABORATIVE CARE FOR ALCOHOL MISUSE - APPROACH TO PHARMACOLOGICAL TREATMENT. *Drug and Alcohol Review*. 2010. 29:73-74	Level 1, Form Title/abstract screen
3968	**E. C. Sundin, T. Baguley**. Prevalence of childhood abuse among people who are homeless in Western countries: a systematic review and meta-analysis. *Social Psychiatry and Psychiatric Epidemiology*. 2015. 50:183-194	Level 1, Form Title/abstract screen
3969	**Y. Sun, L. Y. Zhao, G. B. Wang, W. H. Yue, Y. He, N. Shu, Q. X. Lin, F. Wang, J. L. Li, N. Chen, H. M. Wang, T. R. Kosten, J. J. Feng, J. Wang, Y. D. Tang, S. X. Liu, G. F. Deng, G. H. Diao, Y. L. Tan, H. B. Han, L. Lin, J. Shi**. ZNF804A variants confer risk for heroin addiction and affect decision making and gray matter volume in heroin abusers. *Addiction Biology*. 2016. 21:657-666	Level 1, Form Title/abstract screen

3973	**C. S. Stover**. Commentary: Factors Predicting Family Court Decisions in High-Conflict Divorce. *Journal of the American Academy of Psychiatry and the Law*. 2013. 41:219-223	Level 1, Form Title/abstract screen
3975	**J. M. Stoffers, B. A. Vollm, G. Rucker, A. Timmer, N. Huband, K. Lieb**. Psychological therapies for people with borderline personality disorder. *Cochrane Database of Systematic Reviews*. 2012. #volume#:256	Level 1, Form Title/abstract screen
3977	**M. T. Stewart, C. M. Horgan, A. E. Quinn, D. W. Garnick, S. Reif, T. B. Creedon, E. L. Merrick**. The Role of Health Plans in Supporting Behavioral Health Integration. *Administration and Policy in Mental Health and Mental Health Services Research*. 2017. 44:967-977	Level 1, Form Title/abstract screen
3979	**C. M. Stephen, O. S. Hermiz, E. J. Halcomb, S. McInnes, N. Zwar**. Feasibility and acceptability of a nurse-led hypertension management intervention in general practice. *Collegian*. 2018. 25:33-38	Level 1, Form Title/abstract screen
3982	**B. Spring**. Health Decision Making: Lynchpin of Evidence-Based Practice. *Medical Decision Making*. 2008. 28:866-874	Level 1, Form Title/abstract screen
3983	**H. Spielvogle, C. A. McCarty, L. P. Richardson**. Brief Therapy for Anxiety and Depression in the Pediatric Primary Care Setting Implications and Next Steps. *Jama Pediatrics*. 2017. 171:1006-1007	Level 1, Form Title/abstract screen
3984	**N. A. Sowa, A. Bengtson, B. N. Gaynes, B. W. Pence**. Predictors of depression recovery in HIV-infected individuals managed through measurement-based care in infectious disease clinics. *Journal of Affective Disorders*. 2016. 192:153-161	Level 1, Form Title/abstract screen
3985	**E. Sorensen, J. Goldman, M. Ward, I. Albanese, L. Graves, C. Chamberlain**. JUDICIAL DECISION-MAKING IN CONTESTED CUSTODY CASES - THE INFLUENCE OF REPORTED CHILD-ABUSE, SPOUSE ABUSE, AND PARENTAL SUBSTANCE-ABUSE. *Child Abuse & Neglect*. 1995. 19:251-260	Level 1, Form Title/abstract screen
3986	**T. M. Sokhadze, R. L. Cannon, D. L. Trudeau**. EEG biofeedback as a treatment for substance use disorders: Review, rating of efficacy, and recommendations for further research. *Applied Psychophysiology and Biofeedback*. 2008. 33:1-28	Level 1, Form Title/abstract screen
3987	**T. Soh, A. Lovett**. COLLABORATIVE CARE FOR ALCOHOL MISUSE - EXPLORING THE ROLE OF ADDICTION MEDICINE SPECIALISTS. *Drug and Alcohol Review*. 2010. 29:74-74	Level 1, Form Title/abstract screen
3991	**R. G. Smith**. An Appraisal of Potential Drug Interactions in Cigarette Smokers and Alcohol Drinkers. *Journal of the American Podiatric Medical Association*. 2009. 99:81-88	Level 1, Form Title/abstract screen
3992	**M. Y. Smith, J. D. Depue, C. Rini**. Computerized decision-support systems for chronic pain management in primary care. *Pain Medicine*. 2007. 8:S155-S166	Level 1, Form Title/abstract screen
3995	**C. S. Skinner, K. I. Pollak, D. Farrell, M. K. Olsen, A. S. Jeffreys, J. A. Tulsky**. Use of and reactions to a tailored CD-ROM designed to enhance oncologist-patient communication: The SCOPE trial intervention. *Patient Education and Counseling*. 2009. 77:90-96	Level 1, Form Title/abstract screen
3996	**J. Skeem, J. E. Louden, S. Manchak, S. Vidal, E. Haddad**. Social Networks and Social Control of Probationers with Co-Occurring Mental and Substance Abuse Problems. *Law and Human Behavior*. 2009. 33:122-135	Level 1, Form Title/abstract screen
3999	**J. M. A. Sinclair, S. E. Chambers, C. C. Manson**. Internet Support for Dealing with Problematic Alcohol Use: A Survey of the Soberistas Online	Level 1, Form Title/abstract

	Community. *Alcohol and Alcoholism*. 2017. 52:220-226	screen
4001	**R. Shrestha, P. Karki, F. L. Altice, O. Dubov, L. Fraenkel, T. Huedo-Medina, M. Copenhaver**. Measuring Acceptability and Preferences for Implementation of Pre-Exposure Prophylaxis (PrEP) Using Conjoint Analysis: An Application to Primary HIV Prevention Among High Risk Drug Users. *Aids and Behavior*. 2018. 22:1228-1238	Level 1, Form Title/abstract screen
4013	**L. Shattock, K. Berry, A. Degnan, D. Edge**. Therapeutic alliance in psychological therapy for people with schizophrenia and related psychoses: A systematic review. *Clinical Psychology & Psychotherapy*. 2018. 25:E60-E85	Level 1, Form Title/abstract screen
4014	**F. Shapiro, S. Vogelmannsine, L. F. Sine**. EYE-MOVEMENT DESENSITIZATION AND REPROCESSING - TREATING TRAUMA AND SUBSTANCE-ABUSE. *Journal of Psychoactive Drugs*. 1994. 26:379-391	Level 2, Form Full Text Screening
4015	**A. A. Shalaby, G. E. Brumberg, L. Pointer, D. B. Bekelman, J. S. Rumsfeld, Y. F. Yang, C. N. Pellegrini, P. A. Heidenreich, E. Keung, B. M. Massie, P. D. Varosy**. Depression and Outcome among Veterans with Implantable Cardioverter Defibrillators with or without Cardiac Resynchronization Therapy Capability. *Pace-Pacing and Clinical Electrophysiology*. 2014. 37:994-1001	Level 1, Form Title/abstract screen
4017	**G. Sescousse**. Gambling addiction: insights from neuroscience and neuroimaging. *M S-Medecine Sciences*. 2015. 31:784-791	Level 1, Form Title/abstract screen
4024	**A. C. Schulte, J. E. Easton, J. Parker**. Advances in Treatment Integrity Research: Multidisciplinary Perspectives on the Conceptualization, Measurement, and Enhancement of Treatment Integrity. *School Psychology Review*. 2009. 38:460-475	Level 1, Form Title/abstract screen
4025	**L. Schmidt, T. Greenfield, N. Mulia**. Unequal treatment - Racial and ethnic disparities in alcoholism treatment services. *Alcohol Research & Health*. 2006. 29:49-54	Level 1, Form Title/abstract screen
4029	**I. Schafer, L. Gromus, A. Atabaki, S. Pawils, U. Verthein, J. Reimer, B. Schulte, M. Martens**. Are experiences of sexual violence related to special needs in patients with substance use disorders? A study in opioid-dependent patients. *Addictive Behaviors*. 2014. 39:1691-1694	Level 1, Form Title/abstract screen
4030	**A. J. Saxon, D. A. Calsyn, E. A. Wells, V. V. Stanton**. The use of urine toxicology to enhance patient control of take-home doses in methadone maintenance: Effects on reducing illicit drug use. *Addiction Research*. 1998. 6:203-214	Level 2, Form Full Text Screening
4032	**A. S. A. Sawares, N. Shen, Y. L. Xue, A. Abi-Jaoude, D. Wiljer**. The Impact of Mobile Apps on Alcohol Use Disorder: A Systematic Review Protocol. *Jmir Research Protocols*. 2017. 6:8	Level 1, Form Title/abstract screen
4035	**J. M. Sales, A. Swartzendruber, A. L. Phillips**. Trauma-Informed HIV Prevention and Treatment. *Current Hiv/Aids Reports*. 2016. 13:374-382	Level 1, Form Title/abstract screen
4036	**M. Sajatovic, R. V. Ignacio, J. A. West, K. A. Cassidy, R. Safavi, A. M. Kilbourne, F. C. Blow**. Predictors of nonadherence among individuals with bipolar disorder receiving treatment in a community mental health clinic. *Comprehensive Psychiatry*. 2009. 50:100-107	Level 1, Form Title/abstract screen
4039	**S. Saha, M. Freeman, J. Toure, K. M. Tippens, C. Weeks, S. Ibrahim**. Racial and ethnic disparities in the VA health care system: A systematic review. *Journal of General Internal Medicine*. 2008. 23:654-671	Level 1, Form Title/abstract screen
4042	**P. Saarnio**. Therapists' big five personality traits and interpersonal functioning	Level 1, Form

	in the substance abuse field: A cluster-analytic study. *Journal of Substance Use*. 2011. 16:348-358	Title/abstract screen
4043	**S. Ryan, J. Hislop, S. Ziebland**. Do we all agree what "good health care" looks like? Views from those who are "seldom heard" in health research, policy and service improvement. *Health Expectations*. 2017. 20:878-885	Level 1, Form Title/abstract screen
4044	**R. Ryan, N. Santesso, D. Lowe, S. Hill, J. Grimshaw, M. Prictor, C. Kaufman, G. Cowie, M. Taylor**. Interventions to improve safe and effective medicines use by consumers: an overview of systematic reviews. *Cochrane Database of Systematic Reviews*. 2014. #volume#:74	Level 1, Form Title/abstract screen
4045	**L. Rutkow, K. C. Smith, A. Y. H. Lai, J. S. Vernick, C. S. Davis, G. C. Alexander**. Prescription drug monitoring program design and function: A qualitative analysis. *Drug and Alcohol Dependence*. 2017. 180:395-400	Level 1, Form Title/abstract screen
4051	**M. A. Rosemberg, L. Gultekin, M. Pardee**. High-ACE Low Wage Workers Occupational Health Nursing Research and Praxis Through a Trauma-Informed Lens. *Workplace Health & Safety*. 2018. 66:233-240	Level 1, Form Title/abstract screen
4060	**S. Robie, M. J. Dill, R. S. Hooker**. Commentaries on health services research. *Jaapa-Journal of the American Academy of Physician Assistants*. 2016. 29:2	Level 1, Form Title/abstract screen
4062	**M. Rivalan, S. H. Ahmed, F. Dellu-Hagedorn**. Risk-Prone Individuals Prefer the Wrong Options on a Rat Version of the Iowa Gambling Task. *Biological Psychiatry*. 2009. 66:743-749	Level 1, Form Title/abstract screen
4071	**A. M. F. Reiter, L. Deserno, T. Wilbertz, H. J. Heinze, F. Schlagenhauf**. Risk Factors for Addiction and Their Association with Model-Based Behavioral Control. *Frontiers in Behavioral Neuroscience*. 2016. 10:12	Level 1, Form Title/abstract screen
4072	**S. D. Reed, Y. H. Li, S. Kamble, D. Polsky, F. L. Graham, M. T. Bowers, G. P. Samsa, S. Paul, K. A. Schulman, D. J. Whellan, B. J. Riegel**. Introduction of the Tools for Economic Analysis of Patient Management Interventions in Heart Failure Costing Tool A User-Friendly Spreadsheet Program to Estimate Costs of Providing Patient-Centered Interventions. *Circulation-Cardiovascular Quality and Outcomes*. 2012. 5:113-119	Level 1, Form Title/abstract screen
4074	**L. Rayner, A. Simpson, F. Matcham, S. Shetty, O. Lahoti, G. Groom, M. Hotopf**. Mental disorder in limb reconstruction: Prevalence, associations and impact on work disability. *Journal of Psychosomatic Research*. 2016. 89:53-60	Level 1, Form Title/abstract screen
4078	**J. Raisler**. Midwifery care research: What questions are being asked? What lessons have been learned?. *Journal of Midwifery & Womens Health*. 2000. 45:20-36	Level 1, Form Title/abstract screen
4079	**A. R. Rabinowitz, W. Dundon, H. M. Pettinati, D. Oslin, T. Whittingham, C. Makadon**. Supportive medical management treatment and the therapeutic alliance in the treatment of alcohol dependence. *Alcoholism-Clinical and Experimental Research*. 2006. 30:98A-98A	Level 2, Form Full Text Screening
4080	**R. A. Rabin, K. Kozak, K. K. Zakzanis, G. Remington, C. Stefan, A. J. Budney, T. P. George**. A method to achieve extended cannabis abstinence in cannabis dependent patients with schizophrenia and non-psychiatric controls. *Schizophrenia Research*. 2018. 194:47-54	Level 1, Form Title/abstract screen
4083	**S. Price, J. Goyette**. Role of the psychiatrist in the care of patients with hepatitis C and HIV/AIDS. *Psychiatric Quarterly*. 2003. 74:261-276	Level 1, Form Title/abstract screen
4089	**P. Pinderup**. Training Changes Professionals' Attitudes Towards Dual Diagnosis. *International Journal of Mental Health and Addiction*. 2017. 15:53-	Level 1, Form Title/abstract

	62	screen
4091	**R. Phillips, H. Bourne**. The impact of worker values on client outcomes within a drug treatment service. *International Journal of Drug Policy*. 2008. 19:33-41	Level 2, Form Full Text Screening
4095	**S. Petersen, P. Hutchings, G. Shrader, K. Brake**. Integrating Health Care: The Clear Advantage for Underserved Diverse Populations. *Psychological Services*. 2011. 8:69-81	Level 1, Form Title/abstract screen
4099	**L. Penzenstadler, S. Kolly, S. Rothen, Y. Khazaal, U. Kramer**. Effects of substance use disorder on treatment process and outcome in a ten-session psychiatric treatment for borderline personality disorder. *Substance Abuse Treatment Prevention and Policy*. 2018. 13:11	Level 1, Form Title/abstract screen
4100	**R. T. Penson, C. Nunn, J. Younger, N. J. Schaeffer, B. A. Chabner, G. L. Fricchione, T. E. Quinn, T. J. Lynch**. Trust violated: Analgesics for addicts. *Oncologist*. 2003. 8:199-209	Level 2, Form Full Text Screening
4110	**V. Patel, R. Aroya, S. Chatterjee, D. Chisholm, A. Cohen, M. De Silva, C. Hosman, H. McGuire, G. Rojas, M. van Ommeren**. Global Mental Health 3 - Treatment and prevention of mental disorders in low-income and middle-income countries. *Lancet*. 2007. 370:991-1005	Level 1, Form Title/abstract screen
4111	**T. W. Park, D. M. Cheng, J. H. Samet, M. R. Winter, R. Saitz**. Chronic Care Management for Substance Dependence in Primary Care Among Patients With Co-Occurring Disorders. *Psychiatric Services*. 2015. 66:72-79	Level 1, Form Title/abstract screen
4112	**B. Park, E. Beckman, C. Glatz, A. Pisansky, J. Song**. A place to heal: a qualitative focus group study of respite care preferences among individuals experiencing homelessness. *Journal of Social Distress and the Homeless*. 2017. 26:104-115	Level 1, Form Title/abstract screen
4113	**A. F. Parayre, M. Labrecque, M. Rousseau, S. Turcotte, F. Legare**. Validation of SURE, a Four-Item Clinical Checklist for Detecting Decisional Conflict in Patients. *Medical Decision Making*. 2014. 34:54-62	Level 1, Form Title/abstract screen
4117	**M. Ortendahl, J. F. Fries**. Discounting and risk characteristics in clinical decision-making. *Medical Science Monitor*. 2006. 12:RA41-RA45	Level 1, Form Title/abstract screen
4118	**M. E. M. Ortega, J. C. C. Nieves, A. F. Zamora, P. L. Gelman, B. A. Palma**. Addictions, genomic findings. *Salud Mental*. 2012. 35:129-135	Level 1, Form Title/abstract screen
4120	**V. V. Olsen, R. G. Lugo, S. Sutterlin**. The somatic marker theory in the context of addiction: contributions to understanding development and maintenance. *Psychology Research and Behavior Management*. 2015. 8:187-200	Level 1, Form Title/abstract screen
4125	**C. T. C. Okoli, J. K. Otachi, A. Manuel, M. Woods**. A cross-sectional analysis of factors associated with the intention to engage in tobacco treatment among inpatients in a state psychiatric hospital. *Journal of Psychiatric and Mental Health Nursing*. 2018. 25:14-25	Level 1, Form Title/abstract screen
4126	**M. Oehl, M. Hummer, W. W. Fleischhacker**. Compliance with antipsychotic treatment. *Acta Psychiatrica Scandinavica*. 2000. 102:83-86	Level 1, Form Title/abstract screen
4131	**H. P. O'Rourke, D. P. MacKinnon**. Reasons for Testing Mediation in the Absence of an Intervention Effect: A Research Imperative in Prevention and Intervention Research. *Journal of Studies on Alcohol and Drugs*. 2018. 79:171-181	Level 1, Form Title/abstract screen

4132	**A. O'Brien, R. Fahmy, S. P. Singh**. Disengagement from mental health services. *Social Psychiatry and Psychiatric Epidemiology*. 2009. 44:558-568	Level 1, Form Title/abstract screen
4133	**A. Nyamathi, C. J. Reback, S. Shoptaw, B. E. Salem, S. Zhang, K. Yadav**. Impact of Tailored Interventions to Reduce Drug Use and Sexual Risk Behaviors Among Homeless Gay and Bisexual Men. *American Journal of Mens Health*. 2017. 11:208-220	Level 2, Form Full Text Screening
4135	**C. W. Norwood, E. R. Wright**. Integration of prescription drug monitoring programs (PDMP) in pharmacy practice: Improving clinical decision-making and supporting a pharmacist's professional judgment. *Research in Social & Administrative Pharmacy*. 2016. 12:257-266	Level 1, Form Title/abstract screen
4140	**V. K. Ngo, C. Sherbourne, B. W. Chung, L. Q. Tang, A. L. Wright, Y. Whittington, K. Wells, J. Miranda**. Community Engagement Compared With Technical Assistance to Disseminate Depression Care Among Low-Income, Minority Women: A Randomized Controlled Effectiveness Study. *American Journal of Public Health*. 2016. 106:1833-1841	Level 1, Form Title/abstract screen
4146	**J. Neale, C. N. E. Tompkins, J. Strang**. Qualitative exploration of relationships between peers in residential addiction treatment. *Health & Social Care in the Community*. 2018. 26:E39-E46	Level 1, Form Title/abstract screen
4147	**A. D. Neacsiu, J. W. Eberle, S. L. Keng, C. M. Fang, M. Z. Rosenthal**. Understanding Borderline Personality Disorder Across Sociocultural Groups: Findings, Issues, and Future Directions. *Current Psychiatry Reviews*. 2017. 13:188-223	Level 1, Form Title/abstract screen
4150	**L. M. Najavits, P. Crits-Christoph, A. Dierberger**. Clinicians' impact on the quality of substance use disorder treatment. *Substance Use & Misuse*. 2000. 35:2161-2190	Level 2, Form Full Text Screening
4151	**L. Nafradi, K. Nakamoto, P. J. Schulz**. Is patient empowerment the key to promote adherence? A systematic review of the relationship between self-efficacy, health locus of control and medication adherence. *Plos One*. 2017. 12:23	Level 1, Form Title/abstract screen
4153	**R. Nadeau, K. Hasstedt, A. B. Sunstrum, C. Wagner, H. Tu**. Addressing the Opioid Epidemic: Impact of Opioid Prescribing Protocol at the University of Minnesota School of Dentistry. *Craniomaxillofacial Trauma & Reconstruction*. 2018. 11:104-110	Level 1, Form Title/abstract screen
4156	**A. R. Muse, A. L. Lamson, K. W. Didericksen, J. L. Hodgson**. A Systematic Review of Evaluation Research in Integrated Behavioral Health Care: Operational and Financial Characteristics. *Families Systems & Health*. 2017. 35:136-154	Level 1, Form Title/abstract screen
4162	**J. Morton, S. C. Konrad**. Introducing a Caring/Relational Framework for Building Relationships With Addicted Mothers. *Jognn-Journal of Obstetric Gynecologic and Neonatal Nursing*. 2009. 38:206-213	Level 2, Form Full Text Screening
4167	**M. Morash, D. A. Kashy, S. W. Smith, J. E. Cobina**. THE EFFECTS OF PROBATION OR PAROLE AGENT RELATIONSHIP STYLE AND WOMEN OFFENDERS' CRIMINOGENIC NEEDS ON OFFENDERS' RESPONSES TO SUPERVISION INTERACTIONS. *Criminal Justice and Behavior*. 2015. 42:412-434	Level 1, Form Title/abstract screen
4168	**R. H. Moos**. Theory-based active ingredients of effective treatments for substance use disorders. *Drug and Alcohol Dependence*. 2007. 88:109-121	Level 1, Form Title/abstract screen

4169	**J. E. Moore, A. Mompe, E. Moy**. Disparities by Sex Tracked in the 2015 National Healthcare Quality and Disparities Report: Trends across National Quality Strategy Priorities, Health Conditions, and Access Measures. *Womens Health Issues*. 2018. 28:97-103	Level 1, Form Title/abstract screen
4170	**I. D. Montoya, J. R. Schroeder, K. L. Preston, L. Covi, A. Umbricht, C. Contoreggi, P. J. Fudala, R. E. Johnson, D. A. Gorelick**. Influence of psychotherapy attendance on buprenorphine treatment outcome. *Journal of Substance Abuse Treatment*. 2005. 28:247-254	Level 1, Form Title/abstract screen
4178	**M. Mirsaeidi, R. T. Sadikot**. Patients at High Risk of Tuberculosis Recurrence. *International Journal of Mycobacteriology*. 2018. 7:1-6	Level 1, Form Title/abstract screen
4179	**G. M. Mirdal, E. Ryding, M. E. Sondej**. Traumatized refugees, their therapists, and their interpreters: Three perspectives on psychological treatment. *Psychology and Psychotherapy-Theory Research and Practice*. 2012. 85:436-455	Level 1, Form Title/abstract screen
4184	**L. Mikesell**. Medicinal relationships: caring conversation. *Medical Education*. 2013. 47:443-452	Level 1, Form Title/abstract screen
4185	**M. M. Metzler, D. L. Higgins, C. G. Beeker, N. Freudenberg, P. M. Lantz, K. D. Senturia, A. A. Eisinger, E. A. Viruell-Fuentes, B. Geisar, A. G. Palermo, D. Softley**. Addressing urban health in Detroit, New York City, and Seattle through community-based participatory research partnerships. *American Journal of Public Health*. 2003. 93:803-811	Level 1, Form Title/abstract screen
4190	**T. Meixner, K. Milligan, K. Urbanoski, K. McShane**. Conceptualizing integrated service delivery for pregnant and parenting women with addictions: Defining key factors and processes. *Canadian Journal of Addiction*. 2016. 7:57-65	Level 2, Form Full Text Screening
4193	**P. S. Meier, D. Best**. Programme factors that influence completion of residential treatment. *Drug and Alcohol Review*. 2006. 25:349-355	Level 1, Form Title/abstract screen
4197	**B. D. McLeod, M. A. Southam-Gerow, C. B. Tully, A. Rodriguez, M. M. Smith**. Making a Case for Treatment Integrity as a Psychosocial Treatment Quality Indicator for Youth Mental Health Care. *Clinical Psychology-Science and Practice*. 2013. 20:14-32	Level 1, Form Title/abstract screen
4200	**P. J. McGrath, A. Sourander, P. Lingley-Pottie, T. Ristkari, C. Cunningham, J. Huttunen, K. Filbert, M. Aromaa, P. Corkum, S. Hinkka-Yli-Salomaki, M. Kinnunen, K. Lampi, A. Penttinen, A. Sinokki, A. Unruh, J. Vuorio, C. Watters**. Remote population-based intervention for disruptive behavior at age four: study protocol for a randomized trial of Internet-assisted parent training (Strongest Families Finland-Canada). *Bmc Public Health*. 2013. 13:11	Level 1, Form Title/abstract screen
4204	**D. McCarty, K. C. Priest, P. T. Korthuis**. Treatment and Prevention of Opioid Use Disorder: Challenges and Opportunities. *Annual Review of Public Health, Vol 39*. 2018. 39:525-541	Level 1, Form Title/abstract screen
4205	**R. Mazza, M. Lina, R. Boffi, G. Invernizzi, C. De Marco, M. Pierotti**. Taking care of smoker cancer patients: a review and some recommendations. *Annals of Oncology*. 2010. 21:1404-1409	Level 2, Form Full Text Screening
4206	**V. M. Mays, A. L. Jones, A. Delany-Brumsey, C. Coles, S. D. Cochran**. Perceived Discrimination in Health Care and Mental Health/Substance Abuse Treatment Among Blacks, Latinos, and Whites. *Medical Care*. 2017. 55:173-	Level 1, Form Title/abstract screen

	181	
4207	**J. Mayeya, R. Chazulwa, P. N. Mayeya, E. Mbewe, L. M. Magolo, F. Kasisi, A. C. Bowa**. Zambia mental health country profile. *International Review of Psychiatry*. 2004. 16:63-72	Level 1, Form Title/abstract screen
4208	**S. Mavandadi, J. R. Klaus, D. W. Oslin**. Age Group Differences Among Veterans Enrolled in a Clinical Service for Behavioral Health Issues in Primary Care. *American Journal of Geriatric Psychiatry*. 2012. 20:205-214	Level 1, Form Title/abstract screen
4210	**S. Matthews, P. Smith, P. Chadwick, V. Smyth**. Implementing a community-based structured exercise programme for patients with peripheral arterial disease in conjunction with an existing cardiac rehabilitation service results in better outcomes. *British Journal of Diabetes*. 2016. 16:193-+	Level 1, Form Title/abstract screen
4213	**J. M. Martinez-Gonzalez, N. Albein-Urios, O. Lozano-Rojas, A. Verdejo-Garcia**. Differential aspects of treatment dropout risk in cocaine dependent patients with and without personality disorders. *Adicciones*. 2014. 26:116-125	Level 1, Form Title/abstract screen
4214	**K. A. Martinez, K. Resnicow, G. C. Williams, M. Silva, P. Abrahamse, D. A. Shumway, L. P. Wallner, S. J. Katz, S. T. Hawley**. Does physician communication style impact patient report of decision quality for breast cancer treatment?. *Patient Education and Counseling*. 2016. 99:1947-1954	Level 1, Form Title/abstract screen
4216	**B. Marsh, M. G. Drake**. Outpatient Management for Acute Exacerbations of Obstructive Lung Diseases. *Medical Clinics of North America*. 2017. 101:537-+	Level 1, Form Title/abstract screen
4217	**P. L. Marotta**. Childhood Adversities and Substance Misuse Among the Incarcerated: Implications for Treatment and Practice in Correctional Settings. *Substance Use & Misuse*. 2017. 52:717-733	Level 1, Form Title/abstract screen
4218	**J. Marich**. What Makes a Good EMDR Therapist? Exploratory Findings From Client-Centered Inquiry. *Journal of Humanistic Psychology*. 2012. 52:401-422	Level 2, Form Full Text Screening
4220	**S. R. Marder**. Facilitating compliance with antipsychotic medication. *Journal of Clinical Psychiatry*. 1998. 59:21-25	Level 1, Form Title/abstract screen
4224	**S. L. Manne, D. A. Kashy, S. Rubin, E. Hernandez, C. Bergman**. Therapist and Patient Perceptions of Alliance and Progress in Psychological Therapy for Women Diagnosed With Gynecological Cancers. *Journal of Consulting and Clinical Psychology*. 2012. 80:800-810	Level 1, Form Title/abstract screen
4225	**A. Manhapra, I. Petrakis, R. Rosenheck**. Three-year retention in buprenorphine treatment for opioid use disorder nationally in the Veterans Health Administration. *American Journal on Addictions*. 2017. 26:572-580	Level 1, Form Title/abstract screen
4227	**S. M. Malone, M. Luciana, S. Wilson, J. C. Sparks, R. H. Hunt, K. M. Thomas, W. G. Iacono**. Adolescent Drinking and Motivated Decision-Making: A Cotwin-Control Investigation with Monozygotic Twins. *Behavior Genetics*. 2014. 44:407-418	Level 1, Form Title/abstract screen
4231	**E. Magen, H. M. DeLisser**. Best Practices in Relational Skills Training for Medical Trainees and Providers: An Essential Element of Addressing Adverse Childhood Experiences and Promoting Resilience. *Academic Pediatrics*. 2017. 17:S102-S107	Level 1, Form Title/abstract screen
4232	**J. MacLellan, J. Surey, I. Abubakar, H. R. Stagg, J. Mannell**. Using peer advocates to improve access to services among hard-to-reach populations with hepatitis C: a qualitative study of client and provider relationships. *Harm Reduction Journal*. 2017. 14:9	Level 1, Form Title/abstract screen

4233	**M. A. Mackintosh, L. A. Morland, B. C. Frueh, C. J. Greene, C. S. Rosen**. Peeking into the black box: Mechanisms of action for anger management treatment. *Journal of Anxiety Disorders*. 2014. 28:687-695	Level 1, Form Title/abstract screen
4235	**S. J. Mackain, L. Lecci**. Perceived coercion in substance abuse treatment: The eye of the beholder?. *Journal of Substance Use*. 2010. 15:24-30	Level 1, Form Title/abstract screen
4237	**Y. C. Lou, H. F. Lin**. Estimate of global research trends and performance in family therapy in Social Science Citation Index. *Scientometrics*. 2012. 90:807-823	Level 1, Form Title/abstract screen
4243	**C. Lions, M. P. Carrieri, L. Michel, M. Mora, F. Marcellin, A. Morel, B. Spire, P. Roux, Grp Methaville Study**. Predictors of non-prescribed opioid use after one year of methadone treatment: An attributable-risk approach (ANRS-Methaville trial). *Drug and Alcohol Dependence*. 2014. 135:1-8	Level 2, Form Full Text Screening
4244	**W. R. Lindsay, L. Murphy, G. Smith, D. Murphy, Z. Edwards, C. Chittock, A. Grieve, S. J. Young**. The dynamic risk assessment and management system: An assessment of immediate risk of violence for individuals with offending and challenging behaviour. *Journal of Applied Research in Intellectual Disabilities*. 2004. 17:267-274	Level 1, Form Title/abstract screen
4247	**J. M. Liebschutz, Z. M. Xuan, C. W. Shanahan, M. LaRochelle, J. Keosaian, D. Beers, G. Guara, K. O'Connor, D. P. Alford, V. Parker, R. D. Weiss, J. H. Samet, J. Crosson, P. A. Cushman, K. E. Lasser**. Improving Adherence to Long-term Opioid Therapy Guidelines to Reduce Opioid Misuse in Primary Care A Cluster-Randomized Clinical Trial. *Jama Internal Medicine*. 2017. 177:1265-1272	Level 1, Form Title/abstract screen
4249	**H. A. Liddle**. CONCEPTUAL AND CLINICAL DIMENSIONS OF A MULTIDIMENSIONAL, MULTISYSTEMS ENGAGEMENT STRATEGY IN FAMILY-BASED ADOLESCENT TREATMENT. *Psychotherapy*. 1995. 32:39-58	Level 1, Form Title/abstract screen
4253	**T. Lewinson, M. L. Thomas, S. White**. Traumatic Transitions Homeless Women's Narratives of Abuse, Loss, and Fear. *Affilia-Journal of Women and Social Work*. 2014. 29:192-205	Level 1, Form Title/abstract screen
4254	**S. E. Levkoff, H. T. Chen, E. Coakley, E. C. M. Herr, D. W. Oslin, I. Katz, S. J. Bartels, J. Maxwell, E. Olsen, K. M. Miles, G. Costantino, J. H. Ware**. Design and sample characteristics of the PRISM-E multisite randomized trial to improve behavioral health care for the elderly. *Journal of Aging and Health*. 2004. 16:3-27	Level 1, Form Title/abstract screen
4255	**T. Levin, D. W. Kissane**. Psychooncology - the state of its development in 2006. *European Journal of Psychiatry*. 2006. 20:183-197	Level 1, Form Title/abstract screen
4257	**E. Lenaerts, C. Mathei, F. Matthys, D. Zeeuws, L. Pas, P. Anderson, B. Aertgeerts**. Continuing care for patients with alcohol use disorders: A systematic review. *Drug and Alcohol Dependence*. 2014. 135:9-21	Level 2, Form Full Text Screening
4260	**K. Lehavot, D. Ben-Zeev, R. E. Neville**. Ethical Considerations and Social Media: A Case of Suicidal Postings on Facebook. *Journal of Dual Diagnosis*. 2012. 8:341-346	Level 1, Form Title/abstract screen
4261	**S. LeGrand, S. Reif, K. Sullivan, K. Murray, M. L. Barlow, K. Whetten**. A Review of Recent Literature on Trauma Among Individuals Living with HIV. *Current Hiv/Aids Reports*. 2015. 12:397-405	Level 1, Form Title/abstract screen
4262	**F. Legare, M. C. Politi, R. Drolet, S. Desroches, D. Stacey, H. Bekker, Sdm-**	Level 1, Form

	**Cpd Team**. Training health professionals in shared decision-making: An international environmental scan. *Patient Education and Counseling*. 2012. 88:159-169	Title/abstract screen
4264	**C. J. W. Ledford, J. J. Womack, H. A. Rider, A. B. Seehusen, S. J. Conner, R. A. Lauters, J. A. Hodge**. Unexpected Effects of a System-Distributed Mobile Application in Maternity Care: A Randomized Controlled Trial. *Health Education & Behavior*. 2018. 45:323-330	Level 1, Form Title/abstract screen
4265	**A. J. Lawrence, J. Luty, N. A. Bogdan, B. J. Sahakian, L. Clark**. Problem gamblers share deficits in impulsive decision-making with alcohol-dependent individuals. *Addiction*. 2009. 104:1006-1015	Level 1, Form Title/abstract screen
4269	**D. Lamanna, V. Stergiopoulos, J. Durbin, P. O'Campo, D. Poremski, J. Tepper**. Promoting continuity of care for homeless adults with unmet health needs: The role of brief interventions. *Health & Social Care in the Community*. 2018. 26:56-64	Level 1, Form Title/abstract screen
4270	**P. Lagisetty, K. Klasa, C. Bush, M. Heisler, V. Chopra, A. Bohnert**. Primary care models for treating opioid use disorders: What actually works? A systematic review. *Plos One*. 2017. 12:40	Level 1, Form Title/abstract screen
4273	**C. T. LaBelle, S. C. Han, A. Bergeron, J. H. Samet**. Office-Based Opioid Treatment with Buprenorphine (OBOT-B): State-wide Implementation of the Massachusetts Collaborative Care Model in Community Health Centers. *Journal of Substance Abuse Treatment*. 2016. 60:6-13	Level 1, Form Title/abstract screen
4274	**S. Y. Kye, K. Park**. Psychosocial Factors and Health Behavior among Korean Adults: A Cross-sectional Study. *Asian Pacific Journal of Cancer Prevention*. 2012. 13:49-56	Level 1, Form Title/abstract screen
4275	**K. Kuusisto, V. Knuuttila, P. Saarnio**. Pre-Treatment Expectations in Clients: Impact on Retention and Effectiveness in Outpatient Substance Abuse Treatment. *Behavioural and Cognitive Psychotherapy*. 2011. 39:257-271	Level 1, Form Title/abstract screen
4279	**A. Krupski, K. Campbell, J. M. Joesch, B. A. Lucenko, P. Roy-Byrne**. Impact of Access to Recovery services on alcohol/drug treatment outcomes. *Journal of Substance Abuse Treatment*. 2009. 37:435-442	Level 1, Form Title/abstract screen
4280	**K. Kroenke, J. Unutzer**. Closing the False Divide: Sustainable Approaches to Integrating Mental Health Services into Primary Care. *Journal of General Internal Medicine*. 2017. 32:404-410	Level 1, Form Title/abstract screen
4281	**T. Krmpotich, S. Mikulich-Gilbertson, J. Sakai, L. Thompson, M. T. Banich, J. Tanabe**. Impaired Decision-Making, Higher Impulsivity, and Drug Severity in Substance Dependence and Pathological Gambling. *Journal of Addiction Medicine*. 2015. 9:273-280	Level 1, Form Title/abstract screen
4285	**T. L. Kramer, K. L. Drummond, G. M. Curran, J. C. Fortney**. Assessing Culture and Climate of Federally Qualified Health Centers: A Plan for Implementing Behavioral Health Interventions. *Journal of Health Care for the Poor and Underserved*. 2017. 28:973-987	Level 1, Form Title/abstract screen
4286	**D. D. Krahn, S. J. Bartels, E. Coakley, D. W. Oslin, H. T. Chen, J. McIntyre, H. Chung, J. Maxwell, J. Ware, S. E. Levkoff**. PRISM-E: Comparison of integrated care and enhanced specialty referral models in depression outcomes. *Psychiatric Services*. 2006. 57:946-953	Level 1, Form Title/abstract screen
4288	**A. Koski-Jannes, T. Hirschovits-Gerz, M. Pennonen**. Population, Professional, and Client Support for Different Models of Managing Addictive Behaviors. *Substance Use & Misuse*. 2012. 47:296-308	Level 1, Form Title/abstract screen
4289	**P. T. Korthuis, D. McCarty, M. Weimer, C. Bougatsos, I. Blazina, B.**	Level 1, Form

	**Zakher, S. Grusing, B. Devine, R. Chou**. Primary Care-Based Models for the Treatment of Opioid Use Disorder A Scoping Review. *Annals of Internal Medicine*. 2017. 166:268-+	Title/abstract screen
4292	**C. Kong, M. Dunn, M. Parker**. Psychiatric Genomics and Mental Health Treatment: Setting the Ethical Agenda. *American Journal of Bioethics*. 2017. 17:3-12	Level 1, Form Title/abstract screen
4293	**M. E. Kolodziej, P. M. Muchowski, N. R. Hamdi, P. Morrissette, A. J. McGowan, R. D. Weiss**. Adaptation of the Patient Feedback Survey at a Community Treatment Setting. *American Journal on Addictions*. 2012. 21:63-71	Level 1, Form Title/abstract screen
4294	**B. Koekkoek, B. van Meijel, B. Tiemens, A. Schene, G. Hutschemaekers**. What makes community psychiatric nurses label non-psychotic chronic patients as 'difficult': patient, professional, treatment and social variables. *Social Psychiatry and Psychiatric Epidemiology*. 2011. 46:1045-1053	Level 1, Form Title/abstract screen
4295	**K. A. Kobak, J. C. Mundt, B. Kennard**. Integrating technology into cognitive behavior therapy for adolescent depression: a pilot study. *Annals of General Psychiatry*. 2015. 14:10	Level 1, Form Title/abstract screen
4300	**Y. Kishi, R. G. Kathol, D. D. McAlpine, W. H. Meller, S. W. Richards**. What should non-US behavioral health systems learn from the USA? US behavior health services trends in the 1980s and 1990s. *Psychiatry and Clinical Neurosciences*. 2006. 60:261-270	Level 1, Form Title/abstract screen
4302	**R. King, T. O'Brien, S. G. Giacomantonio**. Psychoanalytic perspectives on substance use and antisocial personality disorder. *Australian Psychologist*. 2005. 40:137-145	Level 1, Form Title/abstract screen
4310	**M. S. Kendra, J. J. Mohr, J. W. Pollard**. The Stigma of Having Psychological Problems: Relations With Engagement, Working Alliance, and Depression in Psychotherapy. *Psychotherapy*. 2014. 51:563-573	Level 1, Form Title/abstract screen
4311	**P. C. Kendall, T. H. Ollendick**. Setting the research and practice agenda for anxiety in children and adolescence: A topic comes of age. *Cognitive and Behavioral Practice*. 2004. 11:65-74	Level 1, Form Title/abstract screen
4317	**E. C. Katz, B. S. Brown, R. P. Schwartz, K. E. O'Grady, S. D. King, D. Gandhi**. Transitioning opioid-dependent patients from detoxification to long-term treatment: Efficacy of intensive role induction. *Drug and Alcohol Dependence*. 2011. 117:24-30	Level 1, Form Title/abstract screen
4318	**W. J. Katon, A. Unutzer, G. Simon**. Treatment of depression in primary care - Where we are, where we can go. *Medical Care*. 2004. 42:1153-1157	Level 1, Form Title/abstract screen
4319	**N. J. Kaslow, S. Kapoor, S. E. Dunn, C. C. Graves**. Psychologists' Contributions to Patient-Centered Medical Homes. *Journal of Clinical Psychology in Medical Settings*. 2015. 22:199-212	Level 1, Form Title/abstract screen
4326	**N. Jordan, M. W. Sohn, B. Bartle, M. Valenstein, Y. Lee, T. A. Lee**. Association Between Chronic Illness Complexity and Receipt of Evidence-based Depression Care. *Medical Care*. 2014. 52:S126-S131	Level 1, Form Title/abstract screen
4331	**A. W. Jones**. The impact of Alcohol and Alcoholism among substance abuse journals. *Alcohol and Alcoholism*. 1999. 34:25-34	Level 1, Form Title/abstract screen
4333	**J. Johnson, M. L. Williams**. A PRELIMINARY ETHNOGRAPHIC DECISION TREE MODEL OF INJECTION-DRUG USERS (IDUS) NEEDLE SHARING. *International Journal of the Addictions*. 1993. 28:997-1014	Level 1, Form Title/abstract screen

4336	**G. W. Joe, K. M. Broome, D. D. Simpson, G. A. Rowan-Szal**. Counselor perceptions of organizational factors and innovations training experiences. *Journal of Substance Abuse Treatment*. 2007. 33:171-182	Level 1, Form Title/abstract screen
4337	**M. C. Jhandler, W. H. Mason**. SOLUTION-FOCUSED THERAPY - AN ALTERNATIVE APPROACH TO ADDICTIONS NURSING. *Perspectives in Psychiatric Care*. 1995. 31:8-13	Level 2, Form Full Text Screening
4340	**B. T. Jensen, S. V. Lauridsen, J. B. Jensen**. Prehabilitation for major abdominal urologic oncology surgery. *Current Opinion in Urology*. 2018. 28:243-250	Level 1, Form Title/abstract screen
4341	**M. Jansen, Mmem van Doorn, A. Lichtwarck-Aschoff, Rcwm Kuijpers, H. Theunissen, M. Korte, J. van Rossum, A. Wauben, I. Granic**. Effectiveness of a cognitive-behavioral therapy (CBT) manualized program for clinically anxious children: study protocol of a randomized controlled trial. *Bmc Psychiatry*. 2012. 12:9	Level 1, Form Title/abstract screen
4343	**S. M. Jack, M. Ford-Gilboe, D. Davidov, H. L. MacMillan, Nfp Ipv Res Team**. Identification and assessment of intimate partner violence in nurse home visitation. *Journal of Clinical Nursing*. 2017. 26:2215-2228	Level 1, Form Title/abstract screen
4345	**L. Iozzino, C. Ferrari, M. Large, O. Nielssen, G. de Girolamo**. Prevalence and Risk Factors of Violence by Psychiatric Acute Inpatients: A Systematic Review and Meta-Analysis. *Plos One*. 2015. 10:18	Level 1, Form Title/abstract screen
4350	**K. Humphreys, J. C. Blodgett, T. H. Wagner**. Estimating the Efficacy of Alcoholics Anonymous without Self-Selection Bias: An Instrumental Variables Re-Analysis of Randomized Clinical Trials. *Alcoholism-Clinical and Experimental Research*. 2014. 38:2688-2694	Level 1, Form Title/abstract screen
4351	**D. Hui, Z. M. Weinstein, D. M. Cheng, E. Quinn, H. Kim, C. Labelle, J. H. Samet**. Very early disengagement and subsequent re-engagement in primary care Office Based Opioid Treatment (OBOT) with buprenorphine. *Journal of Substance Abuse Treatment*. 2017. 79:12-19	Level 1, Form Title/abstract screen
4353	**H. Huang, Y. F. Chan, A. M. Bauer, J. Suzuki, W. Katon, J. Russo, D. Hogan, J. Unutzer**. Specialty Behavioral Health Service Use Among Chronically Ill Medicare Advantage Patients With Substance Use Problems. *Psychosomatics*. 2013. 54:546-551	Level 1, Form Title/abstract screen
4357	**S. Hornberger, S. L. Smith**. Family involvement in adolescent substance abuse treatment and recovery: What do we know? What lies ahead?. *Children and Youth Services Review*. 2011. 33:S70-S76	Level 2, Form Full Text Screening
4358	**C. M. Horgan, D. W. Garnick, E. L. Merrick, A. Hoyt**. Health plan requirements for mental health and substance use screening in primary care. *Journal of General Internal Medicine*. 2007. 22:930-936	Level 1, Form Title/abstract screen
4362	**H. Hohenberger, K. Delahanty**. Patient-Centered Care-Enhanced Recovery After Surgery and Population Health Management. *Aorn Journal*. 2015. 102:578-583	Level 1, Form Title/abstract screen
4364	**A. Hogue, E. Lichvar, M. Bobek**. Pilot Evaluation of the Medication Integration Protocol for Adolescents With ADHD in Behavioral Care: Treatment Fidelity and Medication Uptake. *Journal of Emotional and Behavioral Disorders*. 2016. 24:223-234	Level 1, Form Title/abstract screen
4376	**J. Hettema, J. Steele, W. R. Miller**. Motivational interviewing. *Annual Review of Clinical Psychology*. 2005. 1:91-111	Level 1, Form Title/abstract screen
4377	**K. A. Hepner, M. Rowe, K. Rost, S. C. Hickey, C. D. Sherbourne, D. E.**	Level 1, Form

	**Ford, L. S. Meredith, L. V. Rubenstein**. The effect of adherence to practice guidelines on depression outcomes. *Annals of Internal Medicine*. 2007. 147:320-329	Title/abstract screen
4378	**P. Hemming, A. Hewitt, J. J. Gallo, R. Kessler, R. B. Levine**. Residents' Confidence Providing Primary Care With Behavioral Health Integration. *Family Medicine*. 2017. 49:361-368	Level 1, Form Title/abstract screen
4380	**C. J. Heckman, F. Zhu, S. L. Manne, J. D. Kloss, B. N. Collins, S. B. Bass, S. R. Lessin**. Process and outcomes of a skin protection intervention for young adults. *Journal of Health Psychology*. 2013. 18:561-573	Level 1, Form Title/abstract screen
4381	**C. L. Haynes, G. A. Cook**. Audit of health promotion practice within a UK hospital: results of a pilot study. *Journal of Evaluation in Clinical Practice*. 2008. 14:103-109	Level 1, Form Title/abstract screen
4382	**E. J. Hawkins, C. A. Malte, J. S. Baer, D. R. Kivlahan**. Prevalence, predictors, and service utilization of patients with recurrent use of Veterans Affairs substance use disorder specialty care. *Journal of Substance Abuse Treatment*. 2012. 43:221-230	Level 1, Form Title/abstract screen
4383	**E. J. Hawkins, C. A. Malte**. Prioritizing the reach and patient-centeredness of substance use-related care. *American Journal of Drug and Alcohol Abuse*. 2016. 42:245-249	Level 1, Form Title/abstract screen
4385	**E. J. Hawkins, J. S. Baer, D. R. Kivlahan**. Concurrent monitoring of psychological distress and satisfaction measures as predictors of addiction treatment retention. *Journal of Substance Abuse Treatment*. 2008. 35:207-216	Level 1, Form Title/abstract screen
4387	**B. Hartzler, B. Beadnell, D. Donovan**. Predictive Validity of Addiction Treatment Clinicians' Post-Training Contingency Management Skills for Subsequent Clinical Outcomes. *Journal of Substance Abuse Treatment*. 2017. 72:126-133	Level 1, Form Title/abstract screen
4388	**G. H. Harris, S. M. Strauss, C. Katigbak, B. S. Brar, L. S. Brown, S. S. Kipnis, S. A. Kritz, M. W. Parrino**. Variation among state-level approaches to addressing alcohol abuse in opioid treatment programs. *Journal of Substance Abuse Treatment*. 2010. 39:58-64	Level 1, Form Title/abstract screen
4393	**K. P. Haggerty, S. E. Barkan, M. L. Skinner, W. Ben Packard, J. J. Cole**. Feasibility of Connecting, a Substance-Abuse Prevention Program for Foster Teens and their Caregivers. *Journal of the Society for Social Work and Research*. 2016. 7:639-659	Level 1, Form Title/abstract screen
4394	**J. Haggarty, B. O'Connor, S. Dubois, A. M. Blackadar, T. McKinnon, D. Boudreau, D. Haslam**. A pilot study of a Canadian shared mental health care programme: Changes in patient symptoms and disability. *Primary Care & Community Psychiatry*. 2008. 13:27-35	Level 1, Form Title/abstract screen
4395	**S. E. Hadland, J. F. Wharam, M. A. Schuster, F. Zhang, J. H. Samet, M. R. Larochelle**. Trends in Receipt of Buprenorphine and Naltrexone for Opioid Use Disorder Among Adolescents and Young Adults, 2001-2014. *Jama Pediatrics*. 2017. 171:747-755	Level 1, Form Title/abstract screen
4396	**F. Haarig, S. Muhlig**. Conjoint Analysis for Measuring Treatment Preferences of Patients With Psychiatric Disorders: A Systematic Literature Review. *Zeitschrift Fur Klinische Psychologie Und Psychotherapie*. 2015. 44:1-16	Level 1, Form Title/abstract screen
4397	**B. L. N. Gutierrez, F. F. Palacios**. A search for a different world. Social representation that determines decision-making 26 by Mexican adolescent user of illegal drugs. *Salud Mental*. 2004. 27:26-34	Level 1, Form Title/abstract screen
4399	**R. Gueorguieva, R. Wu, J. H. Krystal, D. Donovan, S. S. O'Malley**.	Level 1, Form

	Temporal patterns of adherence to medications and behavioral treatment and their relationship to patient characteristics and treatment response. *Addictive Behaviors*. 2013. 38:2119-2127	Title/abstract screen
4403	**K. A. Greiner**. THE ETHICS OF USING ALTERNATIVE THERAPIES IN HIV AIDS. *Aids Patient Care*. 1995. 9:175-181	Level 1, Form Title/abstract screen
4404	**S. F. Greenfield, D. E. Sugarman, C. M. Freid, G. L. Bailey, M. A. Crisafulli, J. S. Kaufman, S. Wigderson, H. S. Connery, J. Rodolico, A. A. Morgan-Lopez, G. M. Fitzmaurice**. Group therapy for women with substance use disorders: Results from the Women's Recovery Group Study. *Drug and Alcohol Dependence*. 2014. 142:245-253	Level 1, Form Title/abstract screen
4405	**T. C. Green, J. Johnson, M. Harrington, E. R. Pouget, A. G. Rhodes, F. S. Taxman, D. J. O'Connell, S. S. Martin, M. Prendergast, P. D. Friedmann**. Parole Officer-parolee Relationships and HIV Risk Behaviors during Community Supervision. *Aids and Behavior*. 2013. 17:2667-2675	Level 1, Form Title/abstract screen
4408	**D. B. Green, B. B. Pua, C. B. Crawford, G. N. Abbey, I. R. Drexler, A. C. Legasto, J. F. Gruden**. Screening for Lung Cancer: Communicating With Patients. *American Journal of Roentgenology*. 2018. 210:497-502	Level 1, Form Title/abstract screen
4412	**L. Grabbe, A. F. Amar, U. A. Kelly**. The Triad of Childhood Trauma, Mental Illness, and Substance Abuse: Applying Trauma-Informed Care. *Journal of the American Psychiatric Nurses Association*. 2014. 20:65-65	Level 1, Form Title/abstract screen
4413	**A. Gorini, C. Lucchiari, W. Russell-Edu, G. Pravettoni**. Modulation of risky choices in recently abstinent dependent cocaine users: a transcranial direct-current stimulation study. *Frontiers in Human Neuroscience*. 2014. 8:9	Level 1, Form Title/abstract screen
4418	**D. J. Goldsmith, K. A. Lindholm, J. J. Bute**. Dilemmas of talking about lifestyle changes among couples coping with a cardiac event. *Social Science & Medicine*. 2006. 63:2079-2090	Level 1, Form Title/abstract screen
4420	**S. B. Goldberg, A. C. Del Re, W. T. Hoyt, J. M. Davis**. The Secret Ingredient in Mindfulness Interventions? A Case for Practice Quality Over Quantity. *Journal of Counseling Psychology*. 2014. 61:491-497	Level 1, Form Title/abstract screen
4423	**K. Giyaur, J. Sharf, M. J. Hilsenroth**. The capacity for dynamic process scale (CDPS) and patient engagement in opiate addiction treatment. *Journal of Nervous and Mental Disease*. 2005. 193:833-838	Level 1, Form Title/abstract screen
4425	**J. M. Girard, A. G. C. Wright, J. E. Beeney, S. A. Lazarus, L. N. Scott, S. D. Stepp, P. A. Pilkonis**. Interpersonal problems across levels of the psychopathology hierarchy. *Comprehensive Psychiatry*. 2017. 79:53-69	Level 1, Form Title/abstract screen
4431	**E. V. Gifford**. Commentary on Cunningham et al. (2012): Benefit to clients - outcome monitoring and knowledge translation. *Addiction*. 2012. 107:1525-1526	Level 1, Form Title/abstract screen
4432	**L. G. Gidding, M. G. Spigt, G. J. Dinant**. Patients with psychological ICPC codes in primary care; a case-control study investigating the decade before presenting with problems. *European Journal of General Practice*. 2017. 23:217-224	Level 1, Form Title/abstract screen
4433	**C. A. Gibson**. Review of posttraumatic stress disorder and chronic pain: The path to integrated care. *Journal of Rehabilitation Research and Development*. 2012. 49:753-776	Level 1, Form Title/abstract screen
4434	**S. W. Gibbons, L. Migliore, S. P. Convoy, S. Greiner, P. H. DeLeon**. Military Mental Health Stigma Challenges: Policy and Practice Consideration's. *Jnp-Journal for Nurse Practitioners*. 2014. 10:365-372	Level 1, Form Title/abstract screen

4435	**C. J. Gibbons, C. Nich, K. Steinberg, R. A. Roffman, J. Corvino, T. F. Babor, K. M. Carroll**. Treatment process, alliance and outcome in brief versus extended treatments for marijuana dependence. *Addiction*. 2010. 105:1799-1808	Level 2, Form Full Text Screening
4441	**E. Gervilla, B. Cajal, A. Palmer**. Quantification of the influence of friends and antisocial behaviour in adolescent consumption of cannabis using the ZINB model and data mining. *Addictive Behaviors*. 2011. 36:368-374	Level 1, Form Title/abstract screen
4442	**M. R. Gerber, M. W. King, K. M. Iverson, S. L. Pineles, S. G. Haskell**. Association Between Mental Health Burden and Coronary Artery Disease in US Women Veterans Over 45: A National Cross-Sectional Study. *Journal of Womens Health*. 2018. 27:238-244	Level 1, Form Title/abstract screen
4445	**J. Gaume, N. Heather, G. Tober, J. McCambridge**. A Mediation Analysis of Treatment Processes in the UK Alcohol Treatment Trial. *Journal of Consulting and Clinical Psychology*. 2018. 86:321-329	Level 1, Form Title/abstract screen
4450	**M. P. Garcia-Portilla, L. Garcia-Alvarez, F. Sarramea, G. Galvan, E. Diaz-Mesa, T. Bobes-Bascaran, S. Al-Halabi, E. Elizagarate, C. Iglesias, P. A. S. Martinez, J. Bobes**. It is feasible and effective to help patients with severe mental disorders to quit smoking: An ecological pragmatic clinical trial with transdermal nicotine patches and varenicline. *Schizophrenia Research*. 2016. 176:272-280	Level 1, Form Title/abstract screen
4452	**L. Garbrick, M. A. Levitt, M. Barrett, L. Graham**. Agreement between emergency physicians and psychiatrists regarding admission decisions. *Academic Emergency Medicine*. 1996. 3:1027-1030	Level 1, Form Title/abstract screen
4454	**C. Galandra, G. Basso, S. Cappa, N. Canessa**. The alcoholic brain: neural bases of impaired reward-based decision-making in alcohol use disorders. *Neurological Sciences*. 2018. 39:423-435	Level 1, Form Title/abstract screen
4455	**J. S. Funderburk, D. E. Sugarman, A. K. Labbe, A. Rodrigues, S. A. Maisto, B. Nelson**. Behavioral Health Interventions Being Implemented in a VA Primary Care System. *Journal of Clinical Psychology in Medical Settings*. 2011. 18:22-29	Level 1, Form Title/abstract screen
4456	**R. Fuchs-Strizek, T. Berger**. Psychocardiology in inpatient rehabilitation. *Wiener Medizinische Wochenschrift*. 2018. 168:31-38	Level 1, Form Title/abstract screen
4461	**R. C. Freeman, G. M. Rodriguez, J. F. French**. Compliance with AZT treatment regimen of HIV-seropositive injection drug users: A neglected issue. *Aids Education and Prevention*. 1996. 8:58-71	Level 1, Form Title/abstract screen
4470	**M. I. Fontao, C. Massau, K. Hoffmann, T. Ross**. Therapeutic Alliance and Therapeutic Processes in a Group Therapy with Substance Addicted Offenders. *Gruppenpsychotherapie Und Gruppendynamik*. 2012. 48:173-197	Level 1, Form Title/abstract screen
4471	**C. A. Fontanella, D. L. Hiance-Steelesmith, R. Gilchrist, J. A. Bridge, D. Weston, J. V. Campo**. Quality of Care for Medicaid-Enrolled Youth with Bipolar Disorders. *Administration and Policy in Mental Health and Mental Health Services Research*. 2015. 42:126-138	Level 1, Form Title/abstract screen
4472	**S. A. Flocke, E. Antognoli, M. M. Step, S. Marsh, T. Parran, M. J. Mason**. A teachable moment communication process for smoking cessation talk: description of a group randomized clinician-focused intervention. *Bmc Health Services Research*. 2012. 12:13	Level 1, Form Title/abstract screen
4475	**J. Finke**. PATIENTS EXPECTATIONS CONCERNING PSYCHOTHERAPY IN A PSYCHIATRIC-HOSPITAL - AN EMPIRICAL-INVESTIGATION. *Psychiatrische Praxis*. 1995. 22:112-116	Level 1, Form Title/abstract screen

4476	**C. Field, R. Caetano**. The Role of Ethnic Matching Between Patient and Provider on the Effectiveness of Brief Alcohol Interventions With Hispanics. *Alcoholism-Clinical and Experimental Research*. 2010. 34:262-271	Level 1, Form Title/abstract screen
4481	**S. B. Fawcett, A. PaineAndrews, V. T. Francisco, J. A. Schultz, K. P. Richter, R. K. Lewis, E. L. Williams, K. J. Harris, J. Y. Berkley, J. L. Fisher, C. M. Lopez**. Using empowerment theory in collaborative partnerships for community health and development. *American Journal of Community Psychology*. 1995. 23:677-697	Level 1, Form Title/abstract screen
4484	**M. Farrell, L. Gowing, J. Marsden, W. Ling, R. Ali**. Effectiveness of drug dependence treatment in HIV prevention. *International Journal of Drug Policy*. 2005. 16:S67-S75	Level 1, Form Title/abstract screen
4485	**J. R. Fann, C. H. Bombardier, J. S. Richards, C. S. Wilson, A. W. Heinemann, A. M. Warren, L. Brooks, C. B. McCullumsmith, N. R. Temkin, C. Warms, D. G. Tate, Prisms Invest**. Venlafaxine Extended-Release for Depression Following Spinal Cord Injury A Randomized Clinical Trial. *Jama Psychiatry*. 2015. 72:247-258	Level 1, Form Title/abstract screen
4487	**K. M. Fairfield, H. Libman, R. B. Davis, D. M. Eisenberg, R. S. Phillips**. Delays in protease inhibitor use in clinical practice. *Journal of General Internal Medicine*. 1999. 14:395-401	Level 1, Form Title/abstract screen
4489	**R. M. Etheridge, R. L. Hubbard**. Conceptualizing and assessing treatment structure and process in community-based drug dependency treatment programs. *Substance Use & Misuse*. 2000. 35:1757-1795	Level 1, Form Title/abstract screen
4491	**S. J. Erickson, M. Gerstle, S. W. Feldstein**. Brief interventions and motivational interviewing with children, adolescents, and their parents in pediatric health care settings - A review. *Archives of Pediatrics & Adolescent Medicine*. 2005. 159:1173-1180	Level 1, Form Title/abstract screen
4494	**B. Engle, M. J. Macgowan, E. F. Wagner, P. C. Amrhein**. Markers of Marijuana Use Outcomes Within Adolescent Substance Abuse Group Treatment. *Research on Social Work Practice*. 2010. 20:271-282	Level 1, Form Title/abstract screen
4497	**L. E. Egede, R. Acierno, R. G. Knapp, C. Lejuez, M. Hernandez-Tejada, E. H. Payne, B. C. Frueh**. Psychotherapy for depression in older veterans via telemedicine: a randomised, open-label, non-inferiority trial. *Lancet Psychiatry*. 2015. 2:693-701	Level 1, Form Title/abstract screen
4498	**E. O. Efraimsson, B. Klang, A. Ehrenberg, K. Larsson, B. Fossum, L. Olai**. Nurses' and patients' communication in smoking cessation at nurse-led COPD clinics in primary health care. *European Clinical Respiratory Journal*. 2015. 2:13	Level 1, Form Title/abstract screen
4500	**E. J. Edelman, N. B. Hansen, C. J. Cutter, C. Danton, L. E. Fiellin, P. G. O'Connor, E. C. Williams, S. A. Maisto, K. J. Bryant, D. A. Fiellin**. Implementation of integrated stepped care for unhealthy alcohol use in HIV clinics. *Addiction Science & Clinical Practice*. 2016. 11:14	Level 1, Form Title/abstract screen
4501	**V. Dzul-Church, J. W. Cimino, S. R. Adler, P. Wong, W. G. Anderson**. "I'm Sitting Here By Myself ... ": Experiences of Patients with Serious Illness at an Urban Public Hospital. *Journal of Palliative Medicine*. 2010. 13:695-701	Level 1, Form Title/abstract screen
4504	**L. L. DuBenske, D. H. Gustafson, B. R. Shaw, J. F. Cleary**. Web-Based Cancer Communication and Decision Making Systems: Connecting Patients, Caregivers, and Clinicians for Improved Health Outcomes. *Medical Decision Making*. 2010. 30:732-744	Level 1, Form Title/abstract screen
4505	**B. G. Druss, S. A. von Esenwein**. Improving general medical care for persons	Level 1, Form

	with mental and addictive disorders: systematic review. *General Hospital Psychiatry*. 2006. 28:145-153	Title/abstract screen
4507	**K. H. Drieschner, S. M. M. Lammers, C. P. F. van der Staak**. Treatment motivation: An attempt for clarification of an ambiguous concept. *Clinical Psychology Review*. 2004. 23:1115-1137	Level 1, Form Title/abstract screen
4508	**R. E. Drake, E. L. O'Neal, M. A. Wallach**. A systematic review of psychosocial research on psychosocial interventions for people with co-occurring severe mental and substance use disorders. *Journal of Substance Abuse Treatment*. 2008. 34:123-138	Level 1, Form Title/abstract screen
4512	**D. M. Donovan, D. C. Daley, G. S. Brigham, C. C. Hodgkins, H. I. Perl, A. S. Floyd**. How Practice and Science Are Balanced and Blended in the NIDA Clinical Trials Network: The Bidirectional Process in the Development of the STAGE-12 Protocol as an Example. *American Journal of Drug and Alcohol Abuse*. 2011. 37:408-416	Level 1, Form Title/abstract screen
4513	**J. M. Donohue, R. G. Frank**. Medicaid behavioral health carve-outs: A new generation of privatization decisions. *Harvard Review of Psychiatry*. 2000. 8:231-241	Level 1, Form Title/abstract screen
4518	**C. DiIorio, P. O. Shafer, R. Letz, T. R. Henry, D. L. Schomer, K. Yeager, Ease Study Grp Project**. Project EASE: a study to test a psychosocial model of epilepsy medication management. *Epilepsy & Behavior*. 2004. 5:926-936	Level 1, Form Title/abstract screen
4519	**E. Dieperink, A. Knott, P. Thuras, C. Pocha**. The effect of stimulant use on antiviral treatment in an integrated hepatitis clinic. *General Hospital Psychiatry*. 2013. 35:387-392	Level 1, Form Title/abstract screen
4521	**S. Dewing, C. Mathews, M. Lurie, A. Kagee, T. Padayachee, C. Lombard**. Predictors of poor adherence among people on antiretroviral treatment in Cape Town, South Africa: a case-control study. *Aids Care-Psychological and Socio-Medical Aspects of Aids/Hiv*. 2015. 27:342-349	Level 1, Form Title/abstract screen
4522	**M. Devens**. Personality disorders. *Primary Care*. 2007. 34:623-+	Level 1, Form Title/abstract screen
4524	**C. B. Dennis, B. D. Roland, B. Loneck**. The impact of twelve-step program familiarity and its in-session discussion on counselor credibility. *American Journal of Drug and Alcohol Abuse*. 2013. 39:298-303	Level 1, Form Title/abstract screen
4528	**L. Del Piccolo, C. Goss**. People-centred care: new research needs and methods in doctor-patient communication. Challenges in mental health. *Epidemiology and Psychiatric Sciences*. 2012. 21:145-149	Level 1, Form Title/abstract screen
4529	**F. K. Del Boca, J. Darkes**. Enhancing the validity and utility of randomized clinical trials in addictions treatment research: I. Treatment implementation and research design. *Addiction*. 2007. 102:1047-1056	Level 1, Form Title/abstract screen
4533	**L. de Visser, L. J. van der Knaap, Ajae van de Loo, C. M. M. van der Weerd, F. Ohl, R. van den Bos**. Trait anxiety affects decision-making differently in healthy men and women: Towards gender-specific endophenotypes of anxiety. *Neuropsychologia*. 2010. 48:1598-1606	Level 1, Form Title/abstract screen
4534	**H. A. de Haan, E. A. G. Joosten, A. G. M. Wijdeveld, P. B. Boswinkel, J. van der Palen, C. A. J. De Jong**. Cognitive Behavioural Treatment Is as Effective in High- as in Low-Scoring Alexithymic Patients with Substance-Related Disorders. *Psychotherapy and Psychosomatics*. 2011. 80:254-255	Level 1, Form Title/abstract screen
4537	**D. Davis, M. Hawk, D. Winkler**. Because the clients told us so. *Housing Care and Support*. 2017. 20:164-174	Level 1, Form Title/abstract

		screen
4538	**M. J. Davies, S. Heller, T. C. Skinner, M. J. Campbell, M. E. Carey, S. Cradock, H. M. Dallosso, H. Daly, Y. Doherty, S. Eaton, C. Fox, L. Oliver, K. Rantell, G. Rayman, K. Khunti, Ongoin Diabet Educ Self Management**. Effectiveness of the diabetes education and self management for ongoing and newly diagnosed (DESMOND) programme for people with newly diagnosed type 2 diabetes: cluster randomised controlled trial. *British Medical Journal*. 2008. 336:491-495	Level 1, Form Title/abstract screen
4542	**G. d'Ettorre, V. Pellicani**. Workplace Violence Toward Mental Healthcare Workers Employed in Psychiatric Wards. *Safety and Health at Work*. 2017. 8:337-342	Level 1, Form Title/abstract screen
4544	**W. D'Andrea, L. Bergholz, A. Fortunato, J. Spinazzola**. Play to the Whistle: A Pilot Investigation of a Sports-Based Intervention for Traumatized Girls in Residential Treatment. *Journal of Family Violence*. 2013. 28:739-749	Level 1, Form Title/abstract screen
4545	**P. Czobor, R. A. Van Dorn, L. Citrome, R. S. Kahn, W. W. Fleischhacker, J. Volavka**. Treatment adherence in schizophrenia: A patient-level meta-analysis of combined CATIE and EUFEST studies. *European Neuropsychopharmacology*. 2015. 25:1158-1166	Level 1, Form Title/abstract screen
4546	**P. A. Cushman, J. M. Liebschutz, B. J. Anderson, M. R. Moreau, M. D. Stein**. Buprenorphine Initiation and Linkage to Outpatient Buprenorphine do not Reduce Frequency of Injection Opiate Use Following Hospitalization. *Journal of Substance Abuse Treatment*. 2016. 68:68-73	Level 1, Form Title/abstract screen
4548	**L. Culpepper**. Primary care treatment of attention-deficit/hyperactivity disorder. *Journal of Clinical Psychiatry*. 2006. 67:51-58	Level 1, Form Title/abstract screen
4549	**R. A. Crowley, N. Kirschner, Phys Amer Coll**. The Integration of Care for Mental Health, Substance Abuse, and Other Behavioral Health Conditions into Primary Care: Executive Summary of an American College of Physicians Position Paper. *Annals of Internal Medicine*. 2015. 163:298-+	Level 1, Form Title/abstract screen
4550	**P. CritsChristoph, L. Siqueland, J. Blaine, A. Frank, L. Luborsky, L. S. Onken, L. Muenz, M. E. Thase, R. D. Weiss, D. R. Gastfriend, G. Woody, J. P. Barber, S. F. Butler, D. Daley, S. Bishop, L. M. Najavits, J. Lis, D. Mercer, M. L. Griffin, K. Moras, A. T. Beck**. The National Institute on Drug Abuse Collaborative Cocaine Treatment Study - Rationale and methods. *Archives of General Psychiatry*. 1997. 54:721-726	Level 1, Form Title/abstract screen
4555	**P. Crits-Christoph, J. E. Johnson, M. B. C. Gibbons, R. Gallop**. Process Predictors of the Outcome of Group Drug Counseling. *Journal of Consulting and Clinical Psychology*. 2013. 81:23-34	Level 1, Form Title/abstract screen
4559	**H. M. Crane, W. Lober, E. Webster, R. D. Harrington, P. K. Crane, T. E. Davis, M. M. Kitahata**. Routine collection of patient-reported outcomes in an HIV clinic setting: The first 100 patients. *Current Hiv Research*. 2007. 5:109-118	Level 1, Form Title/abstract screen
4566	**S. S. Coughlin, J. J. Prochaska, L. B. Williams, G. M. Besenyi, V. Heboyan, D. S. Goggans, W. Yoo, G. De Leo**. Patient web portals, disease management, and primary prevention. *Risk Management and Healthcare Policy*. 2017. 10:33-40	Level 1, Form Title/abstract screen
4568	**C. U. Correll, L. Citrome, P. M. Haddad, J. Lauriello, M. Olfson, S. M. Calloway, J. M. Kane**. The Use of Long-Acting Injectable Antipsychotics in Schizophrenia: Evaluating the Evidence. *Journal of Clinical Psychiatry*. 2016. 77:3-+	Level 1, Form Title/abstract screen

4570	**L. A. Copeland, A. L. Miller, D. E. Welsh, J. F. McCarthy, J. E. Zeber, A. M. Kilbourne**. Clinical and Demographic Factors Associated With Homelessness and Incarceration Among VA Patients With Bipolar Disorder. *American Journal of Public Health*. 2009. 99:871-877	Level 1, Form Title/abstract screen
4575	**J. M. Clem, T. E. Smith, K. V. Richards**. Effects of a Low-Element Challenge Course on Abstinence Self-Efficacy and Group Cohesion. *Research on Social Work Practice*. 2012. 22:151-158	Level 1, Form Title/abstract screen
4577	**K. Clarke, E. Mayo-Wilson, J. Kenny, S. Pilling**. Can non-pharmacological interventions prevent relapse in adults who have recovered from depression? A systematic review and meta-analysis of randomised controlled trials. *Clinical Psychology Review*. 2015. 39:58-70	Level 1, Form Title/abstract screen
4581	**B. Cichocki**. The alliance in psychiatric rehabilitation: Client characteristics associated with the initial alliance in a supported employment program. *Work-a Journal of Prevention Assessment & Rehabilitation*. 2015. 52:811-824	Level 1, Form Title/abstract screen
4586	**T. Cho, M. Toritsuka, Y. Saka, M. Morikawa, T. Kishimoto**. COLLABORATIVE MEDICAL CARE OF ALCOHOL USE DISORDER. *Alcohol and Alcoholism*. 2014. 49:1	Level 1, Form Title/abstract screen
4587	**T. Cho, M. Toritsuka, A. Ino, Y. Saka, M. Harada, T. Kishimoto**. COLLABORATIVE MEDICAL CARE FOR ALCOHOL DEPENDENCE FILL THE GAPS. *Alcoholism-Clinical and Experimental Research*. 2014. 38:69A-69A	Level 1, Form Title/abstract screen
4588	**M. Y. L. Chiu, W. W. N. Ho, W. T. L. Lo, M. G. C. Yiu**. Operationalization of the SAMHSA model of recovery: a quality of life perspective. *Quality of Life Research*. 2010. 19:1-13	Level 1, Form Title/abstract screen
4590	**T. C. Cheng, C. C. Lo**. A Longitudinal Causal Analysis of Impact Made by Collaborative Engagement and Service Receipt on Likelihood of Substantiated Re-Report. *Child Maltreatment*. 2015. 20:258-267	Level 1, Form Title/abstract screen
4591	**Y. F. Chen, J. Madan, N. Welton, I. Yahaya, P. Aveyard, L. Bauld, D. Wang, A. Fry-Smith, M. R. Munafo**. Effectiveness and cost-effectiveness of computer and other electronic aids for smoking cessation: a systematic review and network meta-analysis. *Health Technology Assessment*. 2012. 16:1-+	Level 1, Form Title/abstract screen
4592	**J. J. Chen, T. A. Caller, J. N. Mecchella, D. S. Thakur, K. Homa, C. T. Finn, E. J. Kobylarz, K. A. Bujarski, V. M. Thadani, B. C. Jobst**. Reducing severity of comorbid psychiatric symptoms in an epilepsy clinic using a colocation model: Results of a pilot intervention. *Epilepsy & Behavior*. 2014. 39:92-96	Level 1, Form Title/abstract screen
4594	**J. Charlton, A. Albanese, L. Brodie**. The challenges of type 1 diabetes and new psychoactive substance misuse. *Practical Diabetes*. 2018. 35:81-+	Level 1, Form Title/abstract screen
4598	**D. Casey**. Nurses' perceptions, understanding and experiences of health promotion. *Journal of Clinical Nursing*. 2007. 16:1039-1049	Level 1, Form Title/abstract screen
4599	**J. Cartwright, M. Asbridge**. Passengers' Decisions to Ride With a Driver Under the Influence of Either Alcohol or Cannabis. *Journal of Studies on Alcohol and Drugs*. 2011. 72:86-95	Level 1, Form Title/abstract screen
4600	**S. B. Carswell, M. S. Gordon, J. Gryczynski, S. A. Tangires**. The daily progress system: A proof of concept pilot study of a recovery support technology tool for outpatient substance abuse treatment. *American Journal of Drug and Alcohol Abuse*. 2018. 44:294-301	Level 1, Form Title/abstract screen

4601	**E. Carruzzo, G. Zimmermann, C. Zufferey, M. Monnat, A. Rougemont-Buecking, J. Besson, J. N. Despland**. Motivational interviewing, a new "panacea" in the treatment of substance use disorders? A review of literature. *Pratiques Psychologiques*. 2009. 15:405-413	Level 1, Form Title/abstract screen
4604	**K. M. Carpenter, W. Y. Cheng, J. L. Smith, A. C. Brooks, P. C. Amrhein, R. M. Wain, E. V. Nunes**. "Old Dogs" and New Skills: How Clinician Characteristics Relate to Motivational Interviewing Skills Before, During, and After Training. *Journal of Consulting and Clinical Psychology*. 2012. 80:560-573	Level 1, Form Title/abstract screen
4605	**R. M. Carney, K. E. Freedland, B. C. Steinmeyer, E. H. Rubin, G. Ewald**. Collaborative care for depression symptoms in an outpatient cardiology setting: A randomized clinical trial. *International Journal of Cardiology*. 2016. 219:164-171	Level 1, Form Title/abstract screen
4607	**D. Carise, O. Gurel, A. T. McLellan, K. Dugosh, C. Kendig**. Getting patients the services they need using a computer-assisted system for patient assessment and referral - CASPAR. *Drug and Alcohol Dependence*. 2005. 80:177-189	Level 2, Form Full Text Screening
4623	**R. I. Burton**. PRESIDENTIAL-ADDRESS - DISCOVERING THE RIGHT QUESTIONS - OUR CALL TO ACTION - WHO WILL DEFINE HEALTH-CARE RATIONING, AND HOW. *Journal of Hand Surgery-American Volume*. 1994. 19A:169-180	Level 1, Form Title/abstract screen
4624	**M. R. Burt, A. E. Duke, W. A. Hargreaves**. The program environment scale: Assessing client perceptions of community-based programs for the severely mentally ill. *American Journal of Community Psychology*. 1998. 26:853-879	Level 1, Form Title/abstract screen
4628	**N. L. Brown, V. Luna, M. H. Ramirez, K. A. Vail, C. A. Williams**. Developing an effective intervention for IDU women: A harm reduction approach to collaboration. *Aids Education and Prevention*. 2005. 17:317-333	Level 1, Form Title/abstract screen
4629	**L. D. Brown, G. Townley**. Determinants of Engagement in Mental Health Consumer-Run Organizations. *Psychiatric Services*. 2015. 66:411-417	Level 1, Form Title/abstract screen
4631	**K. M. Broome, P. M. Flynn, D. K. Knight, D. D. Simpson**. Program structure, staff perceptions, and client engagement in treatment. *Journal of Substance Abuse Treatment*. 2007. 33:149-158	Level 1, Form Title/abstract screen
4632	**M. A. Brondani, R. Alan, L. Donnelly**. Stigma of addiction and mental illness in healthcare: The case of patients' experiences in dental settings. *Plos One*. 2017. 12:13	Level 2, Form Full Text Screening
4634	**E. Bridges, M. M. McNeill, N. Munro**. URESEARCH IN REVIEW: ADVANCING CRITICAL CARE PRACTICE. *American Journal of Critical Care*. 2017. 26:77-88	Level 1, Form Title/abstract screen
4636	**L. Brener, W. von Hippel, C. von Hippel, I. Resnick, C. Treloar**. Perceptions of discriminatory treatment by staff as predictors of drug treatment completion: Utility of a mixed methods approach. *Drug and Alcohol Review*. 2010. 29:491-497	Level 1, Form Title/abstract screen
4637	**L. Brener, G. Rose, C. von Hippel, H. Wilson**. Implicit Attitudes, Emotions, and Helping Intentions of Mental Health Workers Toward Their Clients. *Journal of Nervous and Mental Disease*. 2013. 201:460-463	Level 1, Form Title/abstract screen
4643	**K. J. Bozic, E. Lau, K. Ong, V. Chan, S. Kurtz, T. P. Vail, H. E. Rubash, D. J. Berry**. Risk Factors for Early Revision After Primary TKA in Medicare Patients. *Clinical Orthopaedics and Related Research*. 2014. 472:232-237	Level 1, Form Title/abstract screen
4645	**G. Bottesi, M. Ghisi, A. J. Ouimet, M. D. Tira, E. Sanavio**. Compulsivity and	Level 1, Form

	Impulsivity in Pathological Gambling: Does a Dimensional-Transdiagnostic Approach Add Clinical Utility to DSM-5 Classification?. *Journal of Gambling Studies*. 2015. 31:825-847	Title/abstract screen
4646	**X. Bosch-Capblanch, K. Abba, M. Prictor, P. Garner**. Contracts between patients and healthcare practitioners for improving patients' adherence to treatment, prevention and health promotion activities. *Cochrane Database of Systematic Reviews*. 2007. #volume#:64	Level 1, Form Title/abstract screen
4648	**M. A. Bornovalova, S. B. Daughters**. How does dialectical behavior therapy facilitate treatment retention among individuals with comorbid borderline personality disorder and substance use disorders?. *Clinical Psychology Review*. 2007. 27:923-943	Level 1, Form Title/abstract screen
4652	**K. I. Bolla, D. A. Eldreth, J. A. Matochik, J. L. Cadet**. Neural substrates of faulty decision-making in abstinent marijuana users. *Neuroimage*. 2005. 26:480-492	Level 1, Form Title/abstract screen
4653	**M. P. Bogenschutz, C. M. A. Geppert, J. George**. The role of twelve-step approaches in dual diagnosis treatment and recovery. *American Journal on Addictions*. 2006. 15:50-60	Level 1, Form Title/abstract screen
4656	**A. W. Blume, L. V. Lovato**. Empowering the Disempowered: Harm Reduction with Racial/Ethnic Minority Clients. *Journal of Clinical Psychology*. 2010. 66:189-200	Level 1, Form Title/abstract screen
4658	**B. Biagianti, L. Grazzi, O. Gambini, S. Usai, R. Muffatti, S. Scarone, G. Bussone**. Decision-making deficit in chronic migraine patients with medication overuse. *Neurological Sciences*. 2012. 33:S151-S155	Level 1, Form Title/abstract screen
4659	**D. Bhugra, P. Dazzan**. Use of the Mental Health Act criteria in the decision-making process for compulsory admissions: A study of psychiatrists in South London. *Medicine Science and the Law*. 2000. 40:336-344	Level 1, Form Title/abstract screen
4669	**D. Ben-Zeev, S. M. Kaiser, I. Krzos**. SERVICES & POLICY. *Journal of Dual Diagnosis*. 2014. 10:197-203	Level 1, Form Title/abstract screen
4670	**M. F. Bellolio, W. I. Gilani, P. Barrionuevo, M. H. Murad, P. J. Erwin, J. R. Anderson, J. R. Miner, E. P. Hess**. Incidence of Adverse Events in Adults Undergoing Procedural Sedation in the Emergency Department: A Systematic Review and Meta-analysis. *Academic Emergency Medicine*. 2016. 23:119-134	Level 1, Form Title/abstract screen
4672	**C. Beeker, J. M. Kraft, R. Goldman, C. Jorgensen**. Strategies for increasing colorectal cancer screening among African Americans. *Journal of Psychosocial Oncology*. 2001. 19:113-132	Level 1, Form Title/abstract screen
4673	**F. Beauvais**. American Indians and alcohol. *Alcohol Health & Research World*. 1998. 22:253-259	Level 1, Form Title/abstract screen
4679	**L. Bartels, P. Easteal**. Women prisoners' sexual victimisation: ongoing vulnerabilities and possible responses. *Journal of Criminological Research Policy and Practice*. 2016. 2:206-216	Level 1, Form Title/abstract screen
4692	**G. Baranyi, M. Cassidy, S. Fazel, S. Priebe, A. P. Mundt**. Prevalence of Posttraumatic Stress Disorder in Prisoners. *Epidemiologic Reviews*. 2018. 40:134-145	Level 1, Form Title/abstract screen
4697	**J. S. Baer, S. A. Ball, B. K. Campbell, G. M. Miele, E. P. Schoener, K. Tracy**. Training and fidelity monitoring of behavioral interventions in multi-site addictions research. *Drug and Alcohol Dependence*. 2007. 87:107-118	Level 1, Form Title/abstract screen

4699	**M. H. Ayotte, N. Lanctot, M. Tourigny**. Pre-treatment profiles of adolescent girls as predictors of the strength of their working alliances with practitioners in residential care settings. *Children and Youth Services Review*. 2015. 53:61-69	Level 1, Form Title/abstract screen
4706	**L. T. Arciniega, I. Cuzmar, W. R. Miller, J. S. Tonigan**. Therapeutic alliance, treatment compliance, and satisfaction among hispanic female alcohol abusers. *Alcoholism-Clinical and Experimental Research*. 2005. 29:159A-159A	Level 2, Form Full Text Screening
4713	**P. C. G. Alves, C. M. D. Sales, M. Ashworth**. Enhancing the patient involvement in outcomes: a study protocol of personalised outcome measurement in the treatment of substance misuse. *Bmc Psychiatry*. 2013. 13:6	Level 2, Form Full Text Screening
4717	**R. A. Alingh, F. Hoekstra, C. P. van der Schans, F. J. Hettinga, R. Dekker, L. H. V. van der Woude**. Protocol of a longitudinal cohort study on physical activity behaviour in physically disabled patients participating in a rehabilitation counselling programme: ReSpAct. *Bmj Open*. 2015. 5:10	Level 1, Form Title/abstract screen
4720	**F. Alemi, M. R. Haack, A. Harge, R. Dill, L. Benson**. Engaging client's family and friends in online counseling. *Journal of Addictions Nursing*. 2005. 16:47-55	Level 1, Form Title/abstract screen
4725	**B. Adinoff, T. J. Carmody, R. Walker, D. M. Donovan, G. S. Brigham, T. M. Winhusen**. Decision-making processes as predictors of relapse and subsequent use in stimulant-dependent patients. *American Journal of Drug and Alcohol Abuse*. 2016. 42:88-97	Level 1, Form Title/abstract screen
4726	**S. Y. Adams, A. G. Crawford, R. N. Rimal, J. S. Lee, L. M. Janneck, C. N. Sciamanna**. The Effects of a Computer-Tailored Message on Secondary Prevention in Type 2 Diabetes: A Randomized Trial. *Population Health Management*. 2009. 12:197-204	Level 1, Form Title/abstract screen
4728	**S. J. Ackerman, M. J. Hilsenroth, E. S. Knowles**. Ratings of therapist dynamic activities and alliance early and late in psychotherapy. *Psychotherapy*. 2005. 42:225-231	Level 1, Form Title/abstract screen
4729	**S. J. Ackerman, M. J. Hilsenroth**. A review of therapist characteristics and techniques positively impacting the therapeutic alliance. *Clinical Psychology Review*. 2003. 23:1-33	Level 1, Form Title/abstract screen
4734	**BC Guidelines**. Problem Drinking-Screening and Assessment. *#journal#*. #year#. #volume#:#pages#	Level 1, Form Title/abstract screen
4735	**BCCSU**. A guideline for the clinical management of opioid use disorders. *#journal#*. #year#. #volume#:#pages#	Level 2, Form Full Text Screening
4736	**BCCSU**. Treatment of Opioid Use Disorder for Youth- Guideline Supplement. *#journal#*. #year#. #volume#:#pages#	Level 2, Form Full Text Screening
4738	**BC Guidelines**. Opioid Use Disorder: Diagnosis and Management in Primary Care. *#journal#*. #year#. #volume#:#pages#	Level 1, Form Title/abstract screen
4739	**College des Medicines du Quebec**. Low-risk drinking guidelines : a guide for physicians and health care professionals. *#journal#*. #year#. #volume#:#pages#	Level 1, Form Title/abstract screen
4740	**CRISM/CIHR**. CRISM National Guideline for the Clinical Management of Opioid Use Disorder. *#journal#*. #year#. #volume#:#pages#	Level 2, Form Full Text Screening

4741	**Ordean, A.; Wong, S.; Graves, L.**. Substance Use in Pregnancy. *#journal#*. #year#. #volume#:#pages#	Level 1, Form Title/abstract screen
4742	**Christina N. Grant, Richard E. Bélanger**. Position Statement: Cannabis and Canada's Children and Youth. *#journal#*. #year#. #volume#:#pages#	Level 1, Form Title/abstract screen
4743	**Canadian Task Force on Preventive Health Care***. Recommendations on behavioural interventions for the prevention and treatment of cigarette smoking among school-aged children and youth. *#journal#*. #year#. #volume#:#pages#	Level 1, Form Title/abstract screen
4745	**Ontario Association of Medical Laboratories**. Guidelines for Ordering Urine Testing for Drugs-of-Abuse: Targeted and Screening Tests (CLP013). *#journal#*. #year#. #volume#:#pages#	Level 1, Form Title/abstract screen
4747	**Canadian Pediatric Society**. Tobacco use and misuse among Indigenous children and youth in Canada. *#journal#*. #year#. #volume#:#pages#	Level 1, Form Title/abstract screen
4748	**Alberta Health Services**. Tobacco Screening and Treatment for Adult Cancer Patients. *#journal#*. #year#. #volume#:#pages#	Level 1, Form Title/abstract screen
4749	**College of Physicians and Surgeons of British Columbia**. Methadone and Buprenorphine: Clinical Practice Guideline for Opioid Use Disorder. *#journal#*. #year#. #volume#:#pages#	Level 1, Form Title/abstract screen
4750	**Linda Ritchie, PhD, RN; Marilyn K. Evans, PhD, RN; and Janet Matthews, MScN, RN**. Nursing students and clinical instructors' perceptions on the implementation of a best practice guideline.. *#journal#*. #year#. #volume#:#pages#	Level 1, Form Title/abstract screen
4754	**BC Patient Safety and Quality Council**. BC's Quality and Safety Community: Findings from an Environmental Scan and Stakeholder Consultation. *#journal#*. #year#. #volume#:#pages#	Level 1, Form Title/abstract screen
4755	**Health Quality Council**. Think Big, Start Small, Act Now: Tackling Indicator Chaos. *#journal#*. #year#. #volume#:#pages#	Level 1, Form Title/abstract screen
4756	**BC Ministry of Health**. Best Practice Guideline for Accommodating and Managing Behavioural and Psychological Symptoms of Dementia in Residential Care: A Person-Centered Interdisciplinary Approach. *#journal#*. #year#. #volume#:#pages#	Level 1, Form Title/abstract screen
4757	**KPMG**. The Need for More Effective Patient- and Family-centred Care Report on the patient experience component of the Saskatchewan Patient First Review. *#journal#*. #year#. #volume#:#pages#	Level 1, Form Title/abstract screen
4758	**KPMG**. The Need for More Effective Patient- and Family-centred Care Detailed research and findings of the patient experience component of the Saskatchewan Patient First Review. *#journal#*. #year#. #volume#:#pages#	Level 1, Form Title/abstract screen
4759	**Health Quality Ontario**. Care Coordination for Postacute Stroke, Chronic Obstructive Pulmonary Disease, and Heart Failure Clients: An Economic Rapid Review. *#journal#*. #year#. #volume#:#pages#	Level 1, Form Title/abstract screen
4760	**Ghazipura, M.** . Care Coordination on Postacute Stroke, Chronic Obstructive Pulmonary Disease, and Heart Failure Clients: A Rapid Review. *#journal#*. #year#. #volume#:#pages#	Level 1, Form Title/abstract screen
4761	**Canadian Pharmacists Association**. Our way forward Optimizing drug	Level 1, Form

	therapy outcomes for Canadians through patient-centred care. *#journal#*. #year#. #volume#:#pages#	Title/abstract screen
4762	**New Brunswick Health Council**. New Brunswickers' Experiences With Primary Health Care. *#journal#*. #year#. #volume#:#pages#	Level 1, Form Title/abstract screen
4764	**Canadian Life and Health Insurance Association Inc.** . CLHIA REPORT ON LONG-TERM CARE POLICY IMPROVING THE ACCESSIBILITY, QUALITY AND SUSTAINABILITY OF LONG-TERM CARE IN CANADA. *#journal#*. #year#. #volume#:#pages#	Level 1, Form Title/abstract screen
4765	**BC Patient Safety & Quality Council**. THE JOURNEY TOWARDS DIGNITY & RESIDENT-CENTERED CARE: SUMMARY RESULTS FROM THE CALL FOR LESS ANTIPSYCHOTICS IN RESIDENTIAL CARE. *#journal#*. #year#. #volume#:#pages#	Level 1, Form Title/abstract screen
4766	**Bernard Richard and Shirley Smallwood Task Force Co-Chairs**. Staying Connected A Report of the Task Force on a Centre of Excellence for Children and Youth with Complex Needs. *#journal#*. #year#. #volume#:#pages#	Level 1, Form Title/abstract screen
4767	**Rivers & Associates**. SUPPORTED CHILD DEVELOPMENT PROGRAM REVIEW. *#journal#*. #year#. #volume#:#pages#	Level 1, Form Title/abstract screen
4768	**Burntwood Regional Health Authority**. Burntwood Regional Health Authority 2009 Community Health Assessment. *#journal#*. #year#. #volume#:#pages#	Level 1, Form Title/abstract screen
4769	**Regional Municipality of Waterloo**. Best Practice in Housing Design for Seniors' Supportive Housing. *#journal#*. #year#. #volume#:#pages#	Level 1, Form Title/abstract screen
4770	**U.S. Department of Health and Human Services Public Health Service**. Helping Smokers Quit A Guide for Clinicians. *#journal#*. #year#. #volume#:#pages#	Level 1, Form Title/abstract screen
4771	**US Department of Health and Human Services**. Treating Tobacco Use and Dependence- Quick reference guide for clinicians. *#journal#*. #year#. #volume#:#pages#	Level 1, Form Title/abstract screen
4772	**U.S. Department of Health and Human Services Public Health Service**. Treating Tobacco Use and Dependence: 2008 Update. *#journal#*. #year#. #volume#:#pages#	Level 1, Form Title/abstract screen
4773	**Agency for Healthcare Research and Quality**. Implementing Medication-Assisted Treatment for Opioid Use Disorder in Rural Primary Care: Environmental Scan Volume 1. *#journal#*. #year#. #volume#:#pages#	Level 1, Form Title/abstract screen
4774	**Agency for Healthcare Research and Quality**. Implementing Medication-Assisted Treatment for Opioid Use Disorder in Rural Primary Care: Environmental Scan Volume 2 Tools and Resources. *#journal#*. #year#. #volume#:#pages#	Level 1, Form Title/abstract screen
4775	**NHS National Treatment Agency for Substance Misuse**. Improving services for substance misuse. *#journal#*. #year#. #volume#:#pages#	Level 2, Form Full Text Screening
4776	**World Health Organization**. mhGAP Intervention Guide for mental, neurological and substance use disorders in non-specialized health settings. *#journal#*. #year#. #volume#:#pages#	Level 1, Form Title/abstract screen
4777	**NHS National Treatment Agency for Substance Misuse**. Good practice in	Level 1, Form Title/abstract

	harm reduction. *#journal#*. #year#. #volume#:#pages#	screen
4778	**NHS National Treatment Agency for Substance Misuse**. Injectable heroin (and injectable methadone). Potential roles in drug treatment. *#journal#*. #year#. #volume#:#pages#	Level 1, Form Title/abstract screen
4779	**NHS National Prescribing Centre**. A guide to good practice in the management of controlled drugs in primary care (England). *#journal#*. #year#. #volume#:#pages#	Level 1, Form Title/abstract screen
4780	**The American College of Obstetricians and Gynecologists**. Alcohol Abuse and Other Substance Use Disorders: Ethical Issues in Obstetric and Gynecologic Practice. *#journal#*. #year#. #volume#:#pages#	Level 1, Form Title/abstract screen
4781	**Canadian Paediatric Society**. Position Statement: Harm reduction: An approach to reducing risky health behaviours in adolescents. *#journal#*. #year#. #volume#:#pages#	Level 1, Form Title/abstract screen
4782	**Committee on Obstetric Practice, American Society of Addiction Medicine**. Opioid Use and Opioid Use Disorder in Pregnancy. *#journal#*. #year#. #volume#:#pages#	Level 1, Form Title/abstract screen
4783	**Harvey, J; Chadi, N.**. Strategies to promote smoking cessation among adolescents. *#journal#*. #year#. #volume#:#pages#	Level 1, Form Title/abstract screen
